# Machine Learning-Based Cardiovascular Disease Prediction Model: A Cohort Study on the Korean National Health Insurance Service Health Screening Database

**DOI:** 10.3390/diagnostics11060943

**Published:** 2021-05-25

**Authors:** Joung Ouk (Ryan) Kim, Yong-Suk Jeong, Jin Ho Kim, Jong-Weon Lee, Dougho Park, Hyoung-Seop Kim

**Affiliations:** 1 Department of AI and Big Data, Swiss School of Management, 6500 Bellinzona, Switzerland; ryonkim@gmail.com (J.O.K.); jinhokim1314@gmail.com (J.H.K.); 2 Department of Cardiology, Brain and Vascular Center, Pohang Stroke and Spine Hospital, Pohang 37659, Korea; lukejungmd@gmail.com; 3 Department of Physical Medicine and Rehabilitation, National Health Insurance Service Ilsan Hospital, Goyang 10444, Korea; bickpjl@gmail.com; 4 Department of Rehabilitation Medicine, Brain and Vascular Center, Pohang Stroke and Spine Hospital, Pohang 37659, Korea

**Keywords:** cardiovascular disease, risk factors, algorithm, machine learning, artificial intelligence

## Abstract

Background: This study proposes a cardiovascular diseases (CVD) prediction model using machine learning (ML) algorithms based on the National Health Insurance Service-Health Screening datasets. Methods: We extracted 4699 patients aged over 45 as the CVD group, diagnosed according to the international classification of diseases system (I20–I25). In addition, 4699 random subjects without CVD diagnosis were enrolled as a non-CVD group. Both groups were matched by age and gender. Various ML algorithms were applied to perform CVD prediction; then, the performances of all the prediction models were compared. Results: The extreme gradient boosting, gradient boosting, and random forest algorithms exhibited the best average prediction accuracy (area under receiver operating characteristic curve (AUROC): 0.812, 0.812, and 0.811, respectively) among all algorithms validated in this study. Based on AUROC, the ML algorithms improved the CVD prediction performance, compared to previously proposed prediction models. Preexisting CVD history was the most important factor contributing to the accuracy of the prediction model, followed by total cholesterol, low-density lipoprotein cholesterol, waist-height ratio, and body mass index. Conclusions: Our results indicate that the proposed health screening dataset-based CVD prediction model using ML algorithms is readily applicable, produces validated results and outperforms the previous CVD prediction models.

## 1. Introduction

Cardiovascular disease (CVD) is the leading cause of death worldwide, accounting for approximately 17.9 million deaths annually which is about 30% of all global deaths [1,2]. In Korea, CVD is rapidly increasing, because people’s lifestyle has changed, and the average age of the population has increased significantly. Currently, CVD is one of the four major diseases in Korea, and ranks second in the leading causes of death, followed by cancer [3]. As of 2019, the mortality rate per 100 thousand people of CVD was 26.7 [4].

Extensive efforts have been made to analyze the causes of CVD [5]. In general, factors such as hypertension, diabetes, hyperlipidemia, and atherosclerosis have been noted as major factors causing CVD [6]. In addition to physiological and genetic risk factors, behavioral and psychosocial factors have also been known as CVD risk factors. Behavioral factors include smoking, food intake, and physical activity. Psychosocial factors include education, financial status, social support, stress, anxiety, and depression [7].

Identifying the association between risk factors and CVD has been a topic of significant research interest, and several CVD risk prediction models have been proposed [8]. Popular models include the American College of Cardiology/American Heart Association (ACC/AHA) risk model and the Framingham risk score [9,10]. The Korean heart study (KHS), one of the largest CVD prediction studies in Korea, introduced a novel 10-year CVD risk prediction model for the Korean population [3,11].

Previous studies on factors and predictive models for CVD have been mostly based on high-quality cohort data obtained by screening or from medical records and patient interviews [11,12]. However, establishing CVD predictive models using clinical data has several limitations. In particular, considerable time and effort is required to acquire and analyze the necessary data, also requiring considerable manpower. Additionally, the methods involved are usually difficult to implement and expensive. To overcome such limitations, it is critical to develop a novel and cost-effective tool for CVD risk prediction [13]. Using the existing National Health Insurance Service-Health Screening (NHIS-healS) database and applying machine learning (ML) algorithms for CVD prediction can provide a heuristic way to build a cost-effective and reliable tool.

This study aimed to present a CVD prediction model using ML algorithms based on nationwide health screening datasets. We also determined the importance of contributing factors related to the CVD prediction performance. Furthermore, we compared the performance of our CVD risk prediction model with that of previous models.

## 2. Materials and Methods

### 2.1. Data Source and Subject Inclusion

The data used for this study were taken from the dataset of the National Health Insurance Corporation (NHIC) for the years 2009 to 2013. The study used the following databases from the NHIC: the NHIS-healS database, examiner’s qualification database, and medical specification database. The study was reviewed and approved by the Institutional Review Board of the National Health Insurance Service Ilsan Hospital (Approval No. NHIS-2021-2-133). The study protocol was also approved by the National Health Insurance data sharing service office.

After extracting the databases, we took the following approaches. First, the disease code in the database was searched. Then, the value “1” was assigned to all users with CVD diagnosis codes. Other users were assigned the value “0.” The CVD diagnosis codes were specified using the International Classification of Diseases-10th system. The codes include angina pectoris (I20), acute myocardial infarction (I21), subsequent myocardial infarction (I22), certain current complications following acute myocardial infarction (I23), other acute ischemic heart diseases (I24), and chronic ischemic heart disease (I25).

From the datasets we used, the overall number of subjects was 234,478 in 2013, and the prevalence of CVD was 2.1% (4962 subjects). Because CVD prevalence differences exist between age groups, we focused on subjects over 45 years old. Consequently, 4699 CVD patients were extracted from the dataset. Then, we sampled the same number of non-CVD subjects assigned the value “0” from the datasets using a random sampling method. We confirmed the same statistical distribution, based on age groups and gender, as those of the CVD group for the sampling, because CVD prevalence is largely affected by age and gender [14]. Finally, a total of 9398 individual datasets were used for training and validating the ML algorithms of the prediction model.

### 2.2. Architecture of the Cardiovascular Disease Prediction Model

**Figure 1:**
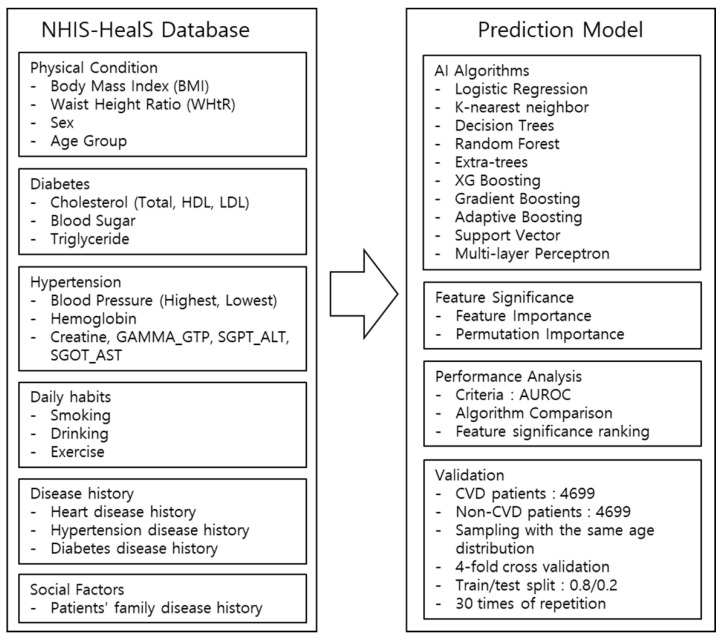
Study process and architecture of the CVD prediction model.

The architecture of the proposed CVD prediction model is shown in Figure 1. The entire code of this study investigation is available on the Online Supplementary Materials. The prediction model validated several ML algorithms and the best model was identified by comparing the performance results after applying the listed algorithms to the datasets. The performance was measured using the criteria of area under the receiver operating characteristic curve (AUROC) scoring mechanism, applying four-fold cross-validation to the datasets. To offset any random sampling effect, the model reiterated the same validation 30 times, the minimum number of iterations to produce the same result. Then, each model represented the final performance comparison results in the average score of AUROC values. Thirty-eight contributing factors were selected and used to build the CVD prediction model (Table S1), and also to measure the relative feature significance contributing to the performance of the model.

### 2.3. Comparison of Prediction Model Performances

The AUROC, accuracy, confusion matrix, classification report, and F1 score were calculated for performance comparison of the CVD prediction model using various ML algorithms. The calculated AUROC value was selected as the main metric to compare the performance of the prediction models. The algorithms used in this study were logistic regression, decision tree, extra tree, K-nearest neighbors, random forest, gradient boosting, adaptive boosting, extreme gradient (XG) boosting, support vector machine, and multi-layer perceptron. The grid search method, supported by the scikit-learn package, was applied to select the optimal hyperparameters of the predictive model algorithms.

### 2.4. Analysis of Contributing Factors Affecting the Prediction Performance

To enhance the prediction model’s performance, it is important to examine which factors contribute the most to the performance of the model. Tree based ML algorithms such as random forest support the feature importance method, which shows the relative ranking of the highest contributing factors impacting the performance of the prediction model. To reinforce the feature significance ranking analysis, another method called the permutation importance method, which can be used in all the aforementioned ML algorithms, is also used to compare the result of the feature importance method. In this study, these two methods running on the random forest algorithm were used simultaneously to examine how each factor contributed to the performance of the CVD prediction model.

### 2.5. Statistical Analysis of Demographic Factors

Continuous variables were expressed as mean ± standard deviation (SD), and categorical variables were expressed as frequencies and proportions. Independent *t*-tests were used to compare the continuous variables between the CVD and non-CVD groups. Chi-square test or Fisher’s exact test were performed to compare the categorical variables between the groups. All statistical analyses were performed using SPSS 22.0 (IBM Inc., Armonk, NY, USA).

## 3. Results

### 3.1. Baseline Characteristics of Subjects

The demographic features and CVD-related factors of both the non-CVD and CVD groups are summarized in Table 1. Both groups were matched for age distribution and gender. There were no significant differences in the heights and frequency of walking exercise between the two groups. In the non-CVD group, the serum levels of total cholesterol (TC) and low-density lipoprotein (LDL) cholesterol were significantly higher than those in the CVD group. The number of subjects who never smoked was higher in the non-CVD group. Current smoking subjects were also more commonly found in the non-CVD group than the CVD group (18.1% and 16.0%, respectively). Regarding alcohol consumption, the proportion of non-drinkers was higher in the CVD group than the non-CVD group (65.8% and 62.8%, respectively). Overall, the non-CVD group also had more subjects with more frequent alcohol consumption than subjects in the CVD group.

### 3.2. Comparisons of Prediction Model Performance

**Figure 2:**
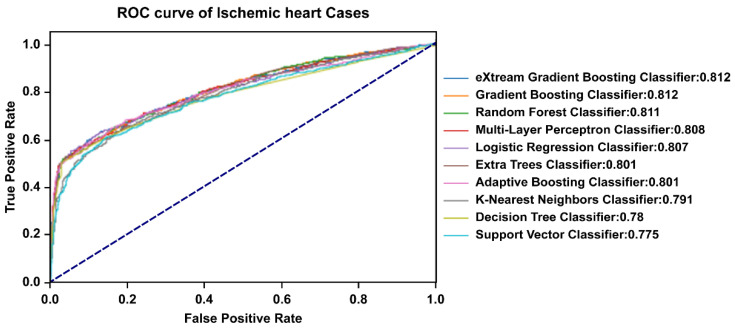
Receiver operating characteristics curve of the prediction performance for each algorithm. The XG boosting, gradient boosting, and random forest algorithms showed the best average prediction accuracy.

To compare the accuracy of the performance of the CVD prediction model, we validated the ML algorithms using 38 contributing factors based on AUROC values (Figure 2). The XG boosting, gradient boosting, and random forest algorithms exhibited the best average prediction accuracy among all utilized algorithms (AUROC: 0.812, 0.812, and 0.811, respectively). In addition, they also presented the best maximum prediction performance (AUROC: 0.822, 0.821, and 0.821, respectively). The results of the CVD prediction model performance for the algorithms are summarized in Table 2.

### 3.3. Contributing Factors for the Prediction Model Performance

**Figure 3:**
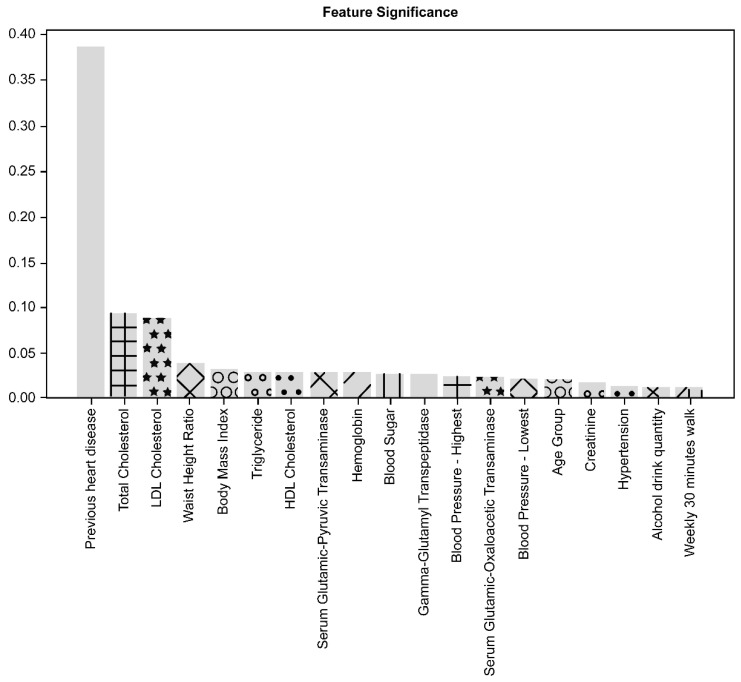
Importance analysis of contributing factors with the feature importance method. Previous CVD was the most important factor, followed by TC, LDL cholesterol, waist-to-height ratio, and body mass index.

**Figure 4:**
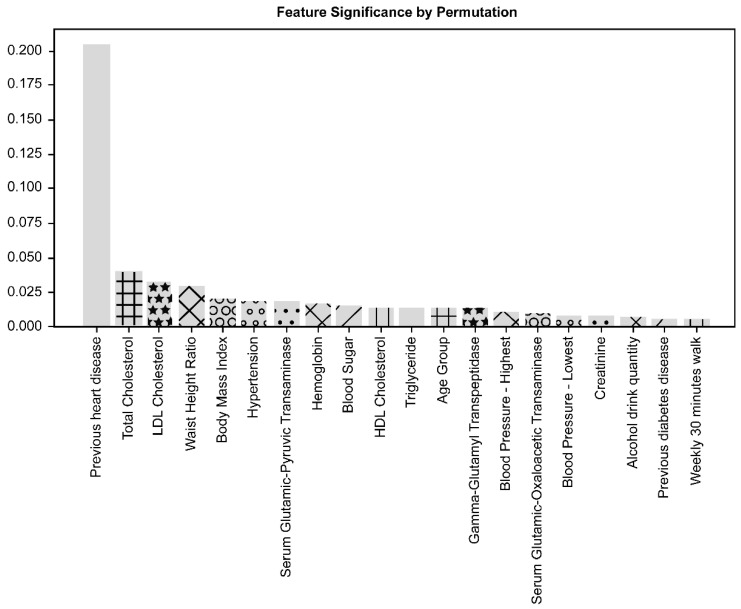
Importance analysis of contributing factors with the permutation importance method. As in the analysis of the feature importance method, previous CVD was the most important factor, followed by TC, LDL cholesterol, waist-to-height ratio, and body mass index.

The significance of contributing factors analyzed by the feature importance method is shown in Figure 3. The significance of contributing factors analyzed by the permutation importance method is shown in Figure 4.

Of the 38 variables considered in this study, previous CVD history was the most important factor contributing to the performance of the CVD prediction model, followed by TC, LDL cholesterol, waist-height ratio (WHtR), and body mass index (BMI). The top five contributing factors demonstrated the same ranking order when applying both methods of feature importance and permutation importance.

The other factors related to medical and behavioral features presented a lower significance of contribution to the prediction performance than the top five factors, representing a slightly different contribution ranking order upon applying both methods. In the analysis of the feature important method, triglyceride and high-density lipoprotein cholesterol followed the top five factors. Meanwhile, in the analysis of the permutation importance method, hypertension history and serum glutamic-pyruvic-transaminase level followed the top five factors.

Among laboratory findings, hemoglobin level, gamma-glutamyl transpeptidase, glutamic-oxaloacetic transaminase, sugar level, creatinine, and blood pressure level were found to be in the top 20 factors in both analyses. Alcohol consumption and walking for 30 min a week were also included in the top 20 factors, and their contributions were higher than other behavioral factors in both analysis methods. The age group was also included in the top 20 factors, but gender was not.

## 4. Discussion

In this study, we proposed a model that predicts the possibility of CVD occurrence by applying ML algorithms to the Korean NHIS-healS data. The accuracy of the proposed model was highest with the XG boosting, gradient boosting, and random forest algorithms. Both XG boosting and gradient boosting exhibited an average AUROC of 0.812, and random forest exhibited an average AUROC of 0.811.

The strong point of our study is, that because the prediction model was performed based only on existing NHIS-healS data, we can suggest that easier and more efficient CVD prediction is possible by using ML algorithms. As most Korean people undergo mandatory health screening every 1‒2 years under the regulation of NHIS, it is also easy to acquire the NHIS-healS data. Therefore, this method obviates additional costs and burdens in collecting baseline data compared to the traditional CVD prediction models.

Moreover, we also showed that the NHIS-healS-based ML algorithm prediction performances are superior to the validation results of previously introduced CVD prediction models—the ACC/AHA risk model, Framingham risk score, and KHS [3,9,10]. The ACC/AHA risk model assesses CVD risk by checking and scoring factors such as gender, age, race, TC, HDL, systolic blood pressure, hypertension treatment, diabetes, and smoking [10]. The Framingham risk score is a gender-specific algorithm that estimates the 10-year CVD risk of an individual. To assess the risk of CVD, the Framingham risk score refers to factors such as age, cholesterol levels, smoking, and blood pressure, as well as risk assessment methods for diseases such as cerebrovascular disease, peripheral artery disease, heart failure, and coronary heart disease [9]. The KHS model is based on 10 years of health screening data, including factors such as hypertension, hyperlipidemia, and diabetes. Moreover, lifestyle factors such as exercise, smoking, drinking, as well as social factors such as education and level of income were also considered [3,11]. Jung et al. [15] investigated the performance of the ACC/AHA risk model in the KHS population, and its AUROC was 0.73 to 0.74. Jee et al. [3] predicted CVD in the KHS population using Framingham risk score, and the result was 0.76 to 0.81 of AUROC. Further, Britton et al. [16] reported several cohort studies that validated the Framingham risk score, and their AUROC was 0.62 to 0.88. Overall, these outcomes are either on par with or lower than that of our model.

Recently, several studies have applied ML algorithms to predict CVD occurrence. Ward et al. [17] presented a 5-year CVD risk prediction framework using ML models for multi-ethnic patients. In their study, the gradient boosting model was the best performing model with AUROC 0.78−0.84. These results are similar to ours. In a study predicting CVD risk based on UK health care data, routine clinical data from 378,256 UK patients were used as cohort data, applying ML algorithms such as logistic regression, random forest, and gradient boosting. They analyzed 24,970 CVD cases with the algorithms, and compared the prediction model’s performance with that of the ACC/AHA risk model. Their results are similar to ours in that the performance of their predictive model was better than that of the existing model [18]. In a study attempting to predict CVD disease risk based on UK Biobank data, data from 423,604 participants without past CVD history were used in a predictive model by applying Auto Prognosis, which is an automated search system. They used 473 available variables and an ensemble technique that allows the model to automatically select an ML and DL algorithm to predict CVD risk. Linear support vector machine, random forest, gradient boosting, adaptive boosting, and neural network algorithms were used for the automated search system. The performance of the model was compared to that of the Framingham risk score, showing that the proposed predictive model outperformed the existing model [19]. Our model achieved a better AUROC value than that the UK Biobank prediction model with an AUROC of 0.77. Although the number of variables considered in our study was 38, which is much less than in their study, the AUROC value of our study is not inferior. Therefore, we have presented a CVD prediction model that is much simpler and easier, and at the same time provides better performance.

As mentioned above, the primary purpose of our study was to propose a simple and efficient CVD prediction model. Although we propose existing ML models with better accuracy, this can be a disadvantage of our study. In other words, the accuracy of our prediction model is lower than that of other studies using combined ML methods to maximize the disease prediction performance [20]. Battineni et al. [21] predicted the diagnosis and onset of Alzheimer disease with a sophisticated ML modeling. In their results, the manual feature selection-based artificial neural network model showed a 0.812 AUROC. However, hybrid modeling with four combined ML algorithms showed a much higher accuracy (AUROC of 0.991). The need for future studies utilizing more advanced and incorporated ML algorithms to maximize CVD prediction performance is also suggested.

CVD related factors have also been studied actively [22,23,24]. In many studies, not only disease-related factors such as diabetes, hypertension, and hyperlipidemia, but also the effects of social, behavioral, and environmental factors were considered as contributing factors for CVD [25,26]. We applied both the feature importance and permutation importance methods to identify the importance of 38 contribution factors. The feature importance method is useful and easy to deploy, native to tree-based algorithms such as random forest [27,28]. However, biased results may occur at times. The permutation importance method is used to overcome such potentially biased results [29,30]. Therefore, we decided to use both methods supported by the scikit-learn package for better explanatory analysis of factors contributing to the performance of the CVD prediction model.

Our results revealed that previous CVD history is the strongest factor contributing to CVD prediction. The dataset used in this study contained both primary and secondary events of CVD as part of medical examination check-up data. The primary CVD event has already been shown to be a strong risk factor for recurrence of CVD [2,31]. Among other medical causes, TC and LDL cholesterol are also important predictive factors for CVD prediction. Paradoxically, TC and LDL cholesterol were significantly higher in the non-CVD group than the CVD group, which was assumed to be due to the higher rate of active medication in the CVD group. Systolic and diastolic blood pressure, and fasting blood glucose had a smaller impact on predicting CVD. Another noteworthy finding was that in our CVD prediction model, factors associated with body shape—WHtR and BMI—showed higher importance than some of the CVD related diseases or laboratory findings related to vascular disease. This is consistent with a previous study by Ortega et al. [32], in which it was reported that obesity is strongly associated with the risk of CVD.

Behavioral factors such as smoking, alcohol consumption, and exercise also represented low contribution importance. Generally, social, behavioral, and environmental factors had a lower direct influence on CVD compared to medical causes [3]. Another notable finding is that age did not significantly affect the performance of the CVD prediction model, similar to other behavioral factors. Age has been known as an important risk factor for vascular diseases such as myocardial infarction and stroke, but our results are somewhat in disagreement with this [33]. In a previous epidemiologic cohort study, acute myocardial infarction exhibited a high incidence in the mid to late-50s, followed by a slight decrease in the occurrence rate during the 60s. Eventually, the incidence increased again after the 70s. The incidence rate was markedly lower than that of stroke from the mid-50s [34]. In this study, there was a high proportion of patients in their 60s and early-50s. Thus, our results reflect these epidemiologic features.

Previous studies conducted on cerebrovascular stroke, another major vascular disease, have revealed that several key causes are involved in stroke incidence. Age, gender, low weight, ethnicity, and genetic factors were risk factors that were either difficult to track, or with minimal medical impact. Medically known risk factors include hypertension, smoking, diabetes, atrial fibrillation, certain other heart diseases, dyslipidemia, carotid artery stenosis, sickle cell disease, postmenopausal hormone therapy, insufficient diet, lack of physical activity and obesity, and distribution of body fat [35,36]. These reports represented a similar tendency of factor importance to CVD prediction.

This study has some limitations. First, we should acknowledge the limitations of the datasets, because the NHIS-HealS datasets do not include detailed clinical data, that is, the details of individual hospital medical records, or clinical datasets, which represent more accurate medical conditions. In particular, in the case of recurring CVD patients, this difference in clinical severity may affect the results. Nevertheless, it is a limitation that this is not sufficiently considered in our study. If a method to link patient clinical data to the NHIS-HealS dataset is available as a consolidated dataset, additional key factors for CVD prediction can be included. This could lead to a more accurate CVD prediction model. Secondly, the fact that we performed CVD prediction model performances only in a single ethnic group can be a selection bias. Furthermore, even though over 9000 subjects were enrolled, our final sample size was relatively small compared to the other aforementioned CVD prediction studies. To build a CVD prediction model that can be applied globally, health screening and clinical datasets from various countries and different ethnicities need to be considered. Efficiently and effectively collecting, sharing, and analyzing research datasets is crucial for realizing more accurate CVD prediction models.

## 5. Conclusions

The CVD prediction model using ML algorithms exhibited superior validation performances compared to those of previously proposed prediction studies. It was also possible to predict CVD occurrence in individuals, readily using existing health screening data and ML algorithms. Therefore, our study verifies that a CVD prediction model using ML algorithms can predict CVD effectively. Among the factors considered in this study, preexisting history of CVD was the most important contributing factor to the prediction model performance. Furthermore, for more powerful and applicable prediction models, detailed patient data and clinical datasets across the globe may need to be considered in future studies.

## Figures and Tables

**Table 1:** Baseline characteristics and major contributing factors in the non-CVD and CVD groups.

	Non-CVD Group (*n* = 4699)	CVD Group (*n* = 4699)	*p* Value
Sex, *n* (%)			1.00 ^a^
Male	2789 (59.4%)	2789 (59.4%)	
Female	1910 (40.6%)	1910 (40.6%)	
Age group, *n* (%)			1.00 ^b^
45–49	248 (5.3%)	248 (5.3%)	
50–54	556 (11.8%)	556 (11.8%)	
55–59	658 (14.0%)	658 (14.0%)	
60–64	893 (19.0%)	893 (19.0%)	
65–69	730 (15.5%)	730 (15.5%)	
70–74	957 (20.4%)	957 (20.4%)	
75–79	411 (8.7%)	411 (8.7%)	
80–84	210 (4.5%)	210 (4.5%)	
≥85	36 (0.8%)	36 (0.8%)	
Height (cm)	161.1 ± 9.0	161.1 ± 8.8	0.82 ^c^
Weight (kg)	62.4 ± 10.4	64.1 ± 10.8	<0.001 ^c^
Waist (cm)	82.8 ± 8.5	84.5 ± 8.5	<0.001 ^c^
Total Cholesterol	196.63 ± 37.52	173.52 ± 40.06	<0.001 ^c^
LDL Cholesterol	116.44 ± 34.55	96.13 ± 35.44	<0.001 ^c^
HDL Cholesterol	50.85 ± 13.56	53.09 ± 16.71	<0.001 ^c^
Triglyceride	135.07 ± 82.23	137.40 ± 85.16	0.18 ^c^
Previous CVD, *n* (%)			<0.001 ^b^
No	4576 (97.4%)	2336 (49.7%)	
Yes	123 (2.6%)	2363 (50.3%)	
Previous Stroke, *n* (%)			0.008 ^b^
No	4607 (98.0%)	4568 (97.2%)	
Yes	92 (2.0%)	131 (2.8%)	
Previous Hypertension, *n* (%)			<0.001 ^a^
No	2949 (62.8%)	2364 (50.3%)	
Yes	1750 (37.2%)	2335 (49.7%)	
Previous Diabetes, *n* (%)			<0.001 ^a^
No	4047 (86.1%)	3680 (78.3%)	
Yes	652 (13.9%)	1019 (21.7%)	
Previous Hyperlipidemia, *n* (%)			<0.001 ^a^
No	4414 (93.9%)	4222 (89.8%)	
Yes	285 (6.1%)	477 (10.2%)	
FH of CVD, *n* (%)			<0.001 ^b^
No	4587 (97.6%)	4284 (91.2%)	
Yes	112 (2.4%)	415 (8.8%)	
FH of Stroke, *n* (%)			<0.001 ^a^
No	4409 (93.8%)	4273 (90.9%)	
Yes	290 (6.2%)	426 (9.1%)	
Smoking Type, *n* (%)			<0.001 ^a^
Never Smoking	2823 (60.1%)	2732 (58.1%)	
Past Smoking	1025 (21.8%)	1215 (25.9%)	
Current Smoking	851 (18.1%)	752 (16.0%)	
Drinking (days/week), *n* (%)			<0.001 ^b^
0	2953 (62.8%)	3219 (68.5%)	
1	634 (13.5%)	516 (11.0%)	
2	450 (9.6%)	395 (8.4%)	
3	290 (6.2%)	258 (5.5%)	
4	115 (2.4%)	91 (1.9%)	
5	70 (1.5%)	86 (1.8%)	
6	59 (1.3%)	44 (0.9%)	
7	128 (2.7%)	90 (1.9%)	
Walk 30 min (days/week), *n* (%)			0.27 ^a^
0	1503 (32.0%)	1482 (31.5%)	
1	337 (7.2%)	322 (6.9%)	
2	507 (10.8%)	506 (10.8%)	
3	584 (12.4%)	651 (13.9%)	
4	355 (7.6%)	328 (7.0%)	
5	423 (9.0%)	410 (8.7%)	
6	304 (6.5%)	270 (5.7%)	
7	686(14.6%)	730 (15.5%)	

CVD: cardiovascular disease, LDL: low-density lipoprotein, HDL: high-density lipoprotein, FH: family history, min: minutes. ^a^ Chi-square test. ^b^ Fisher’s exact test. ^c^ Independent *t*-tests.

**Table 2:** Results of the prediction performance for each algorithm.

	Trials	Mean	SD	Min	25%	50%	75%	Max
eXtream Gradient Boosting	30	0.812	0.005	0.803	0.810	0.812	0.815	0.822
Gradient Boosting	30	0.812	0.005	0.800	0.809	0.812	0.816	0.821
Random Forest	30	0.811	0.005	0.799	0.809	0.811	0.815	0.821
Multi-layer Perceptron	30	0.808	0.006	0.794	0.805	0.808	0.812	0.817
Adaptive Boosting	30	0.806	0.005	0.798	0.802	0.806	0.810	0.818
Logistic Regression	30	0.805	0.005	0.793	0.801	0.805	0.808	0.814
Extra-Trees	30	0.802	0.005	0.791	0.799	0.803	0.806	0.811
K-nearest Neighbors	30	0.792	0.005	0.779	0.789	0.794	0.796	0.802
Decision Tree	30	0.789	0.005	0.780	0.786	0.790	0.793	0.796
Support Vector Machine	30	0.782	0.006	0.766	0.779	0.783	0.786	0.793

SD: standard deviation, Min: minimum result, Max: maximum result.

## Data Availability

The data are not publicly available due to privacy and ethical restrictions of the Korean NHI data sharing system. The dataset used in this study can only be accessed through its own internal-networking system by an authorized researcher. The entire code for this study’s investigation is provided as Online Supplementary Material.

## Supplementary Materials

The following are available online at https://www.mdpi.com/article/10.3390/diagnostics11060943/s1. Table S1: Factors used in ML and DL algorithm-based CVD prediction model; Document S1: Cardiovascular prediction model test codes.
