# Supplementary material for: Machine Learning-Based Cardiovascular Disease Prediction Model: A Cohort Study on the Korean National Health Insurance Service Health Screening Database
Source: Diagnostics (Basel). 2021 May 25;11(6):943. doi: 10.3390/diagnostics11060943 (PMC8229422; doi:10.3390/diagnostics11060943)
Supplement: Supplementary file 1 [file diagnostics-11-00943-s001.zip › Document S1.pdf]

Cardiovascular prediction model test codes

- Dataset: National Health Insurance Service-Health Screening (NHIS-HealS) datasets from 2009 to 2013 provided by Korea National Health Insurance Corporation. 4,699 patients whose ages are over 45 in 2013 was selected as a CVD diagnosed group, where the same number of random patients in the same age and sex distribution was selected as a non-CVD group.
- Diagnosis of cardiovascular diseases (CVD): ICD-10 codes (I20-I25) were extracted to be ground truth database for CVD diagnosis.
- Sampling of Non-CVD dataset: retrived from the NHIS-HealS dataset in a random sampling way, confirming to the same statistical distribution of CVD dataset by considering the same year, age group and sex.
- Features for Prediction model : annual health checkup database including medical examination data related to diabetes, hyperlipidemia, hypertension and other health behavioral features were used to build the CVD prediction model.
- Algorithms of CVD prediction model: Logistic Regression, Decision Tree, Extra Tree, K-NN, Random Forest, Gradient Boosting, ADA Boosting, XG Boosting, Support Vector Machine, and Multi-Layer Perceptron.
- Feature importance: feature importance used for tress-based models and permutation importance supported by scikit-learn package are used.
- Performance comparison: ML algorithms improved the performance of CVD prediction, compared to the ACC/AHA risk model and the Framingham Risk Score.

Contact : JOUNG OUK (Ryan) Kim (ryonkim@gmail.com, ryonkim@naver.com)

In [1]:

```
# Definition of target test cases
target_case_dic = {
                   1:'dementia',
                   2:'ischemic heart',
                   3:'cerebrovascular',
                   4:'atrial_fibrillation'
                   }

# Select cardiovascular (ischemic heart) as target test case
target_case = 2

# Select the minimum target age group, which means the age groups from the selected age group to
the last are used for analysis
# 7: 30-34 years, 8: 35-39 years, 9:40-44 years, 10:45-49 years, 11 : 50-55 years, 12 : 55-59 ye
ars, 13: 60-65 years, 14 : 65-70 years
target_agegroup = 10

# Select target group by health checkup cadence
# 0: Whole dataset in the selected year
# 1: Whole dataset in the all years
# 2: Selected dataset who got health checkup each year repetitively in the seleted year
target_group = 0

# Set a flag variable to show plot during the analysis
# 0 :  Do not show the plots during the analysis
# 1 :  Shows the plots during the analysis
showplot_flag = 0

# Set sampling method by considering age groun and gender or not.
# 0: Do not consider age group, and gener when sampling
# 1: Condier age group, and gender when sampling
sample_method = 1

# Select sampling ration, which means how many times of random samples is selected for analysis
sampling_unit = 1

print("target case : {}, target agegroup : {}, sampling_unit : {}, showplot_flag : {} ".format(
target_case, target_agegroup, sampling_unit, showplot_flag))

# Select target year for analysis
target_year = '2013'

# Set the number of splits for input to kfold
kfold_split_number = 4
print('kfold_split_number:', kfold_split_number)

# Set maximum iteration for repetitive tests
max_iteration = 30
print('max_iteration:', max_iteration)

_DEBUG = False
#_DEBUG = True
print('_DEBUG:', _DEBUG)
```

```
target case : 2, target agegroup : 10, sampling_unit : 1, showplot_flag : 0
kfold_split_number: 4
max_iteration: 30
_DEBUG: False
```

In [2]:

```
#pip install xgboost
```

In [3]:

```
import pandas as pd
import numpy as np
import matplotlib.pyplot as plt
import sys

# from sklearn.preprocessing import LabelEncoder
from sklearn.model_selection import cross_val_score, train_test_split

import warnings
warnings.filterwarnings('ignore')
```

In [4]:

```
# ## Modeling

from sklearn.neural_network import MLPClassifier
from sklearn.gaussian_process import GaussianProcessClassifier
from sklearn.svm import SVC
from sklearn.tree import DecisionTreeClassifier
from sklearn import metrics
from sklearn.ensemble import ExtraTreesClassifier
from sklearn.tree import ExtraTreeClassifier
from sklearn.tree import DecisionTreeClassifier
from sklearn.ensemble import GradientBoostingClassifier
from sklearn.ensemble import BaggingClassifier
from sklearn.ensemble import AdaBoostClassifier
from sklearn.ensemble import RandomForestClassifier
from sklearn.model_selection import GridSearchCV, KFold
from sklearn.linear_model import LogisticRegression
from sklearn.naive_bayes import GaussianNB
from sklearn.neighbors import KNeighborsClassifier
from xgboost import XGBClassifier

from sklearn.metrics import accuracy_score, r2_score, classification_report, confusion_matri
x, roc_curve, auc
from sklearn import metrics
from datetime import datetime

# ### - minmaxscale, stardardscale

from sklearn.preprocessing import MinMaxScaler
from sklearn.preprocessing import StandardScaler

import sklearn.metrics as metrics
```

Fundtion definition for performance comparison of machine learning models

- Confusion Matrix
- TN, FP, FN, TP
- F1 Score
- Sensitivity (Recall)
- Precision
- Accuracy
- AUC-ROC

In [5]:

```
from sklearn.metrics import roc_auc_score

total_roc_auc = {}
total_fpr = {}
total_tpr = {}
total_accuracy = {}
```

In [6]:

```
# Confusion matrix : text

def report_performance(model):

    model_test = model.predict(X_test)

    print("₩nConfusion Matrix:")
    print("{0}".format(metrics.confusion_matrix(y_test, model_test)))
    print("₩nClassification Report: ")
    print(metrics.classification_report(y_test, model_test))
    plot_confusion_metrixs(y_test, model_test)
    print('₩nAUC Score : {}'.format(metrics.roc_auc_score(y_test, model_test)))
```

In [7]:

```
# Confusion matrix : plot

def plot_confusion_metrixs(y_test,model_test):
    cm = metrics.confusion_matrix(y_test, model_test)
    plt.figure(1)
    plt.clf()
    plt.imshow(cm, interpolation='nearest', cmap=plt.cm.Wistia)
    classNames = ['Group A','Group B']
    plt.title('Confusion Matrix')
    plt.ylabel('True label')
    plt.xlabel('Predicted label')
    tick_marks = np.arange(len(classNames))
    plt.xticks(tick_marks, classNames)
    plt.yticks(tick_marks, classNames)
    s = [['TN','FP'], ['FN', 'TP']]
    for i in range(2):
        for j in range(2):
            plt.text(j,i, str(s[i][j])+" = "+str(cm[i][j]))
    plt.show()
```

In [8]:

```
# ROC curve : text

def roc_scores(model, seperator=False):
# 예측
    predictions_test = model.predict_proba(X_test)
    predictions = predictions_test[:,1]
    fpr, tpr, thresholds = roc_curve(y_test, predictions)
    roc_auc = auc(fpr, tpr)

    if seperator == False:
        if _DEBUG:
            print('seperator == False:')
            print('model:', model)
            print("str((str(model).split('(')[0])):", str((str(model).split('(')[0])))

        total_roc_auc[str((str(model).split('(')[0]))] = roc_auc

        print("₩nROC_AUC Score of the model({}): {} ₩n".format( model, roc_auc))

    else:
        if _DEBUG:
            print('seperator == True:')
            print('model:', model)
            model_name = str((str(model).split('=')[5]))
            print('model_name:', model_name)

        model_name = str((str(model).split('=')[5])).split('(')[0]

        if _DEBUG:
            print('model_name:', model_name)

        total_roc_auc[model_name] = roc_auc

        print("₩nROC_AUC Score of the model({}): {} ₩n".format( model_name, roc_auc))
```

In [9]:

```
# ROC curve : plot

def roc_curves(model, seperator=False):
# 예측
    predictions_test = model.predict_proba(X_test)
    predictions = predictions_test[:,1]
    fpr, tpr, thresholds = roc_curve(y_test, predictions)
    roc_auc = auc(fpr, tpr)

# model 비교를 위하여 저장
    if seperator == False:
        total_fpr[str((str(model).split('(')[0]))] = fpr
        total_tpr[str((str(model).split('(')[0]))] = tpr
    else:
        model_name = str((str(model).split('=')[5]))
        model_name = str((str(model).split('=')[5])).split('(')[0]

        total_fpr[model_name] = fpr
        total_tpr[model_name] = tpr


# ROC curve
    plt.figure(figsize=(6,5))
    plt.plot(fpr, tpr, color='darkorange', lw=1, label='ROC curve (area = %0.2f)' % roc_auc)
    plt.plot([0, 1], [0, 1], color='navy', lw=2, linestyle='--', label='Random guess')
    plt.xlim([0.0, 1.0])
    plt.ylim([0.0, 1.05])
    plt.xlabel('False Positive Rate')
    plt.ylabel('True Positive Rate')
    plt.title('receiver operating characteristic (ROC) curve')
    plt.legend(loc="lower right")
    plt.show()
```

In [10]:

```
# Report accuracy

def report_accuracy(model, seperator=False):

    if _DEBUG:
        print('report_accuracy:')

    pred = model.predict(X_test)
    accu = metrics.accuracy_score(y_test,pred)

    if seperator == False:

        if _DEBUG:
            print('seperator == False:')
            print('model:', model)
            print("str((str(model).split('(')[0])):", str((str(model).split('(')[0])))

        total_accuracy[str((str(model).split('(')[0]))] = accu

        print("₩nAccuracy Of the Model({}): {}".format( model, accu))

    else:

        if _DEBUG:
            print('seperator == True:')
            model_name = str((str(model).split('=')[5]))
            print('model_name:', model_name)

        model_name = str((str(model).split('=')[5])).split('(')[0]

        if _DEBUG:
            print('model_name:', model_name)

        total_accuracy[model_name] = accu

        print("₩nAccuracy Of the Model({}): {}".format( model_name, accu))
```

In [11]:

```
# Modelling : optimal parameters are found by using grid search supported by scikit-learn packag
e

def gridsearch_cv_for_classifier(model, param, kfold, train_input, train_target, scoring='roc_au
c', n_jobs=-1, tracking=True):
    '''
    [Parameters]
    - model: A tuple like ('name', MODEL)
    - param
    - scoring: neg_mean_absolute_error, neg_mean_squared_error, neg_median_absolute_error, r2
               (http://scikit-learn.org/stable/modules/model_evaluation.html#scoring-parameter)
    - n_jobs: default as -1 (if it is -1, all CPU cores are used to train and validate models)
    - tracking: whether trained model's name and duration time are printed
    '''

    name = model[0]
    estimator = model[1]
    if tracking:
        start_time = datetime.now()
        print("[%s] Start parameter search for model '%s'" % (start_time, name))
        gridsearch = GridSearchCV(estimator=estimator, param_grid=param, cv=kfold, scoring=scori
ng, n_jobs=n_jobs)
        gridsearch.fit(train_input, train_target)
        end_time = datetime.now()
        duration_time = (end_time - start_time).seconds
        print("[%s] Finish parameter search for model '%s' (time: %d seconds)" % (end_time, nam
e, duration_time))
        print()
    else:
        gridsearch = GridSearchCV(estimator=estimator, param_grid=param, cv=kfold, scoring=scori
ng, n_jobs=n_jobs)
        gridsearch.fit(train_input, train_target)

    return gridsearch
```

Load dataset from the preprocessed CSV file

- Patients with main disease codes in the database were searched, and a label was assigned to all patients with CVD related codes according to International Classification of Diseases (ICD-10) system, including followings:
- Angina pectoris (I20)
- Acute myocardial infarction (I21)
- Subsequent myocardial infarction (I22)
- Certain current complications following acute myocardial infarction (I23)
- Other acute ischemic heart diseases (I24)
- Chronic ischemic heart disease (I25)

In [12]:

```
if target_group == 0 :
    if (target_case == 1):    # Demantia
        input_filename = 'data/data_dementia_' + target_year + '.csv'
    elif (target_case == 2):  # Ischaemic heart
        input_filename = 'data/data_ischaemic_heart_' + target_year + '.csv'
    elif (target_case == 3):  # Cerebravascular
        input_filename = 'data/data_cerebrovascular_' + target_year + '.csv'
    elif (target_case == 4):  # Atrial fibrillation
        input_filename = 'data/data_atrial_fibrillation_' + target_year + '.csv'
elif target_group == 2:
    if (target_case == 1):    # Demantia
        input_filename = 'data/data_dementia_diff_' + target_year + '.csv'
    elif (target_case == 2):  # Ischaemic heart
        input_filename = 'data/data_ischaemic_heart_diff_' + target_year + '.csv'
    elif (target_case == 3):  # Cerebravascular
        input_filename = 'data/data_cerebrovascular_diff_' + target_year + '.csv'
    elif (target_case == 4):  # Atrial fibrillation
        input_filename = 'data/data_atrial_fibrillation_diff_' + target_year + '.csv'

print("₩nInput filename : ", input_filename)

# Set random state value to represent the same test result when needed
random_state_val = 13250
print("₩nrandom state :  ", random_state_val)

# Load dataset from preprocessed CSV file
df = pd.read_csv(input_filename, header=0, index_col=0, encoding='utf-8')
print("₩nTotal number of data from input file : ", len(df))

# Check the simple distribution by age groun and sex
group_count = df.groupby(['GROUP', 'SEX','AGE_GROUP']).size()
print("₩nGroup count by 'SEX' and 'AGE_GROUP'")
print(group_count)

# Load and save the total number of CVD classified group
df_mainsick = df[(df['GROUP'] == 1) ]
print("₩nTotal number of Group 1 (main sick) : ", len(df_mainsick))

# Load and save the total number of CVD classified group based on the selected age group conditi
on
df_mainsick = df[(df['AGE_GROUP'] >= target_agegroup) & (df['GROUP'] == 1) ]
print("Total number of Group 1 (main sick) in target age group :" , len(df_mainsick))

# Load and save the total number of non-CVD classified group based on the selected age group con
dition
df_normal = df[(df['AGE_GROUP'] >= target_agegroup) & (df['GROUP'] == 0) ]
print("Total number of Group 0 (normal) in target age group :", len(df_normal))
```

```
Input filename :  data/data_ischaemic_heart_2013.csv

random state :   13250

Total number of data from input file :  234478

Group count by 'SEX' and 'AGE_GROUP'
GROUP  SEX  AGE_GROUP
0.0    1    2              1
            4            306
            5           2228
            6           8273
            7          14889
            8          13363
            9          18612
            10         14431
            11         15986
            12         11707
            13          9003
            14          5803
            15          5419
            16          2153
            17           993
            18           250
       2    4            292
            5           3444
            6           7279
            7           7263
            8           5476
            9          15701
            10         12246
            11         16310
            12         11238
            13         10038
            14          5768
            15          6363
            16          2659
            17          1536
            18           486
1.0    1    6             12
            7             18
            8             51
            9            127
            10           173
            11           367
            12           430
            13           541
            14           446
            15           498
            16           223
            17            95
            18            16
       2    5              2
            6              6
            7              8
            8              5
            9             34
            10            75
            11           189
            12           228
            13           352
```

```
            14          284
            15          459
            16          188
            17          115
            18           20
dtype: int64

Total number of Group 1 (main sick) :  4962
Total number of Group 1 (main sick) in target age group : 4699
Total number of Group 0 (normal) in target age group : 132389
```

Note for performance matics and ML model settings

1. AUC-ROC

- predict() is used to predict the actual class (In your case one of 0, 1 or 1).
- predict_proba() is used to predict the class probabilities
- The test in this code used predict_proba() when calculating AUC-ROC values.

2. SVC v.s. . LinearSVC

Support Vector machine Classifier needs to set probability=True when model is made, in order to use predict_proba function. LinearSVC does not support predict_proba so if users want to get probability for the class, addtional code like below needs to be cnsidered.

from sklearn.svm import LinearSVC model = LinearSVC() model.fit(X,Y) Y = model.decision_function(X) prob = (Y - Y.min()) / (Y.max() - Y.min()) print('Prob[0]: %.3f' % (1-prob[0])) print('Prob[1]: %.3f' % (prob[0]))

The above code can be used for only binary classification. Therefore, SVC is recommended in other cases than binary classification.

[Reference]

- https://m.blog.naver.com/PostView.nhn?blogId=cjh226&logNo=221358912619&proxyReferer=https:%2F%2Fwww.google.com%2F (https://m.blog.naver.com/PostView.nhn?blogId=cjh226&logNo=221358912619&proxyReferer=https:%2F%2Fwww.google.com%2F)

In [13]:

```
total_roc_aucs = pd.DataFrame(columns = ['result_train_filename', 'RandomState', 'LogisticRegres
sion', 'RandomForestClassifier', 'XGBClassifier',
                           'KNeighborsClassifier', 'DecisionTreeClassifier', 'ExtraTreesClassifie
r',
                          'GradientBoostingClassifier', 'AdaBoostClassifier', 'SVC'])


random_state_val = 12345

for i in range(max_iteration):
    random_state_val = random_state_val + i
    print("random state :  ", random_state_val)

    # Save CVD dataset into the dataframe
    df_sample = pd.DataFrame()
    df_sample = pd.concat([df_sample, df_mainsick])

    # Check group count distribution based on sex and age group target
    group_count = df_mainsick.groupby(['GROUP', 'SEX','AGE_GROUP']).size()
#     print("₩n=== mainsick tarin target ===")
#     print(group_count)

    # Total number of male in the age groups
    agegroup_male = group_count[1][1]
    agegroup_male = agegroup_male.reset_index(name='COUNT')
#     print("₩n=== mail agegroup === ₩n{}".format(agegroup_male))
#     print("mail agegroup sum : {}".format(agegroup_male.sum()))

    # # Total number of female in the age groups
    agegroup_female = group_count[1][2]
    agegroup_female = agegroup_female.reset_index(name='COUNT')
#     print("₩n=== femail agegroup === ₩n{}".format(agegroup_female))
#     print("femail agegroup sum : {}".format(agegroup_female.sum()))

    ### Sampling routine to select random samples of the non-CVD dataset conforming to sex and a
ge group distribution.
    for gender in range(1, 3):
        if gender == 1:
            agegroup_target = agegroup_male
        else :
            agegroup_target = agegroup_female

        for i in range(0, len(agegroup_target)  ):
            age_group, count = agegroup_target.iloc[i]

            # Sampling ration calcuation by setting on sampling_unit
            sampling_count = count * sampling_unit
#             print(gender, age_group, count, sampling_count)
            df_temp = df[(df['GROUP'] == 0) & (df['AGE_GROUP'] == age_group) & (df['SEX'] == gen
der)].sample(n=sampling_count, random_state=random_state_val)
            df_sample = pd.concat([df_sample, df_temp])

    print(len(df_sample))

    df = df_sample.copy()
#     df.info()
#     df.head()

    # Add new feature of WHtR (Waist Height Ratio)
```

```
    df['WHTR'] = df['WAIST']/df['HEIGHT']

    # Add new feature of BMI by calculation
    df['BMI'] = df['WEIGHT']/(df['HEIGHT']*df['HEIGHT']) * 10000

    # Save preprocessed dataset into CSV file
    date_time = datetime.now().strftime("%Y%m%d_%H%M%S")
    result_train_filename = "data/train_test_data_" + target_case_dic.get(target_case) + "_" + t
arget_year + "_" + date_time + ".csv"

    print('result_train_filename:', result_train_filename)
    df.to_csv(result_train_filename, encoding='utf-8')

    # Select feature variables for analysis
    feature_col_names = [
    # Sex
                          'SEX',
    # Age group
                          'AGE_GROUP',

    #                      'HEIGHT', 'WEIGHT',
    #                      'WAIST',
    # BMI
                          'BMI',
    # WHTR
                          'WHTR',
    # Blood pressure : Highest and Lowerst
                          'BP_HIGH', 'BP_LWST',
    # Sugar
                          'BLDS',
    # Cholesterols
                          'TOT_CHOLE', 'HDL_CHOLE','LDL_CHOLE',
    # Tryglyceride
                          'TRIGLYCERIDE',
    # Hemoglobin
                          'HMG',
    # Proteinuria
                          'OLIG_PROTE_CD',
    # Serum
                          'CREATININE', 'SGOT_AST', 'SGPT_ALT', 'GAMMA_GTP',
    # Self disease history
                          'HCHK_APOP_PMH_YN',   # Stroke
                          'HCHK_HDISE_PMH_YN',  # Heart
                          'HCHK_HPRTS_PMH_YN',  # Blood pressure
                          'HCHK_DIABML_PMH_YN', # Diabetes
                          'HCHK_HPLPDM_PMH_YN', # Hyperlipidemia
                          'HCHK_PHSS_PMH_YN',   # Tuberclosis
                          'HCHK_ETCDSE_PMH_YN', # Etc
    # Family disease history
                          'FMLY_APOP_PATIEN_YN',   # Stroke
                          'FMLY_HDISE_PATIEN_YN',  # Heart
                          'FMLY_HPRTS_PATIEN_YN',  # Blood presuure
                          'FMLY_DIABML_PATIEN_YN', # Diabetes
                          'FMLY_CANCER_PATIEN_YN', # Cancer
    #                       'SMK_STAT_TYPE_RSPS_CD', # 'SMK_TERM_RSPS_CD', 'DSQTY_RSPS_CD'
    # Smoking habit
                          'PAST_SMK_TERM_RSPS_CD', # Past smoking period
                          'PAST_DSQTY_RSPS_CD',    # Past smoking amount
                          'CUR_SMK_TERM_RSPS_CD',  # Current smoking period
                          'CUR_DSQTY_RSPS_CD',     # Current smoking amount
```

```
    # Drinking habit
                        'DRNK_HABIT_RSPS_CD',    # Drinking habit
                        'TM1_DRKQTY_RSPS_CD',    # Drinking amount at one time
    # Exercise habit
                        'MOV20_WEK_FREQ_ID',     # Intense exercise more than 20 minutes per week
                        'MOV30_WEK_FREQ_ID',     # Moderate exercise more than 30 minutes per wee
k
                        'WLK30_WEK_FREQ_ID',     # Walking more than 30 minuts per week
    ]   # End of feature_col_names

    # Select target name for classification
    predicted_class_names = ['GROUP']

    # Set feature and target variables
    X = df[feature_col_names].values
    y = df[predicted_class_names].values

    # Set train set and test set with the ratio of 4:1
    X_train, X_test, y_train, y_test = train_test_split(X, y, test_size=0.20, random_state=12345
6)
    print("total count : {}, df_train count : {}, df_test count : {}".format(len(df), len(X_tra
in), len(X_test)))

    print(X_train.shape)
    print(X_test.shape)

    # Standard scaling of dataset
    standardScaler = StandardScaler()
    X_train = standardScaler.fit_transform(X_train)
    X_test = standardScaler.fit_transform(X_test)


    # Set machine learning models to be used for grid search
    models = []
    params = []

    model = ('Logistic Regression', LogisticRegression())
    param = {
            'penalty' : ['l2'],
            'solver' : ['lbfgs'],
            'C' : [0.1, 1, 10, 1e2, 1e3]
    }
    models.append(model)
    params.append(param)

    model = ('Random Forest', RandomForestClassifier())
    param = {
        'n_estimators':[20, 50, 70, 90, 100],
        'max_features': ['auto'],
        'max_depth' : [4,  6,  8, 10],
        'criterion' :['gini']
    }

    models.append(model)
    params.append(param)

    model = ('Extreme Gradient Boosting)', XGBClassifier())
    param = {
        'max_depth' :[2, 5, 8 ],
        'n_estimators' :[20, 40, 50, 100],
        'learning_rate': [0.01, 0.05, 0.075, 0.1],
```

```
    'subsample' : [0.7, 0.8]
}

models.append(model)
params.append(param)


model = ('k-Nearest Neighbours)', KNeighborsClassifier())
param = {'n_neighbors': [4, 10, 20, 40, 60],
         'weights': ['uniform','distance']}

# k-NN is used only for the case of having large enough dataset is prepared
if len(df) >= 100 :
    models.append(model)
    params.append(param)

model = ('Decision Tree', DecisionTreeClassifier())
param = {
    'max_depth' :[3, 4, 5, 7, 9, 11, 15, 20],
    'criterion':['gini','entropy']

}
models.append(model)
params.append(param)

model = ('Extra Tree', ExtraTreesClassifier())
param = {
        'n_estimators':[20, 50, 100],
        'max_features' : ['auto', 'sqrt','log2']
}
models.append(model)
params.append(param)

model = ('Gradient Boosting', GradientBoostingClassifier())
param = {
        'n_estimators':[20, 50, 100],
        'learning_rate': [0.01, 0.075, 0.2],
        'max_features' : [2, 4, 6],
        'max_depth' :[2, 5, 8]
}
models.append(model)
params.append(param)

model = ('Adaptive Boosting', AdaBoostClassifier())
param = {
        'n_estimators':[20, 50, 100],
        'learning_rate': [0.01, 0.075, 0.2],
}

models.append(model)
params.append(param)

model = ('Suppport Vector Classfier', SVC(probability=True))
param = {
    'kernel' : ['linear', 'rbf'],
    'C':[0.1, 1, 10],
    'gamma': [0.1, 1, 10]
}

models.append(model)
params.append(param)
```

```
    model = ('Neural Network', MLPClassifier())
    param = {
        'hidden_layer_sizes': [(50,), (50,50)],
        'activation': ['logistic', 'tanh', 'relu'],
        'alpha': [0.0001, 0.005, 0.001, 0.05],
        'learning_rate': ['constant','adaptive'],
    }
    models.append(model)
    params.append(param)


#    from pprint import pprint
#    pprint(models)
#    print("=" * 60)
#    pprint(params)

    kfold = KFold(n_splits=kfold_split_number, shuffle=True, random_state=random_state_val)

    results = []
    for i in range(len(models)):
        model = models[i]
        param = params[i]
        print(model)
        result = gridsearch_cv_for_classifier(model=model, param=param, kfold=kfold, train_input
=X_train, train_target=y_train, scoring='roc_auc')
        print("=== train : best params ===")
        print(result.best_params_)
        print("=== train : best score : roc_auc ===")
        print(result.best_score_)
        print("₩n")
        results.append(result)

#    for row in results:
#        pprint(row)

    for i in range(len(results)):
        report_performance(results[i])
        report_accuracy(results[i], True)
        roc_curves(results[i], True)
        roc_scores(results[i], True)

    # Model Comparision

    # Accuracy : text
    sorted_total_accuracy= sorted(total_accuracy.items(), key=lambda x: x[1], reverse=True)
    sorted_total_accuracy
    print(sorted_total_accuracy)
    print('₩n')

    # AUC-ROC : text
    sorted_total_auc= sorted(total_roc_auc.items(), key=lambda x: x[1], reverse=True)
    print('sorted_total_auc:₩n', sorted_total_auc)

    # AUC-ROC : plot chart
    count = 0
    sorted_total_auc = dict( sorted_total_auc)
    for i in sorted_total_auc.keys():
#        label_desc = i + ":" + str(total_auc[i])

        model_name = ""
```

```
        if i == "RandomForestClassifier":
            model_name = "Random Forest Classifier"
        elif i == "GradientBoostingClassifier":
            model_name = "Gradient Boosting Classifier"
        elif i == "XGBClassifier":
            model_name = "eXtream Gradient Boosting Classifier"
        elif i == "DecisionTreeClassifier":
            model_name = "Decision Tree Classifier"
        elif i == "MLPClassifier":
            model_name = "Multi-Layer Perceptron Classifier"
        elif i == "ExtraTreesClassifier":
            model_name = "Extra Trees Classifier"
        elif i == "AdaBoostClassifier":
            model_name = "Adaptive Boosting Classifier"
        elif i == "LogisticRegression":
            model_name = "Logistic Regression Classifier"
        elif i == "SVC":
            model_name = "Support Vector Classifier"
        elif i == "KNeighborsClassifier":
            model_name = "K-Nearest Neighbors Classifier"
        else:
            print('Error')

        label_desc = model_name + ":" + str(total_roc_auc[i])

        plt.plot(total_fpr[i],total_tpr[i],lw=1, label=label_desc )
        count += 1

    plt.xlim([0.0, 1.0])
    plt.ylim([0.0, 1.05])
    plt.xlabel('False Positive Rate')
    plt.ylabel('True Positive Rate')
    plt.title('ROC curve of ' + target_case_dic.get(target_case).capitalize() + ' Cases')
    plt.plot([0, 1], [0, 1], color='navy', lw=2, linestyle='--')
    plt.legend(loc='center left', bbox_to_anchor=(1,0.5))
    plt.show()


    # AUC-ROC : plot chart
    count = 0
    sorted_total_auc = dict(sorted_total_auc)
    for i in sorted_total_auc.keys():

        if _DEBUG:
            label_desc = i + ":" + str(total_auc[i])

        model_name = ""

        if i == "RandomForestClassifier":
            model_name = "Random Forest Classifier"
        elif i == "GradientBoostingClassifier":
            model_name = "Gradient Boosting Classifier"
        elif i == "XGBClassifier":
            model_name = "eXtream Gradient Boosting Classifier"
        elif i == "DecisionTreeClassifier":
            model_name = "Decision Tree Classifier"
        elif i == "MLPClassifier":
            model_name = "Multi-Layer Perceptron Classifier"
        elif i == "ExtraTreesClassifier":
            model_name = "Extra Trees Classifier"
```

```
        elif i == "AdaBoostClassifier":
            model_name = "Adaptive Boosting Classifier"
        elif i == "LogisticRegression":
            model_name = "Logistic Regression Classifier"
        elif i == "SVC":
            model_name = "Support Vector Classifier"
        elif i == "KNeighborsClassifier":
            model_name = "K-Nearest Neighbors Classifier"
        else:
            print('Error')

        label_desc = model_name + ":" + str(total_roc_auc[i])

        plt.plot(total_fpr[i],total_tpr[i],lw=1, label=label_desc )
        count += 1
        if count == 3:
            break

    plt.xlim([0.0, 1.0])
    plt.ylim([0.0, 1.05])
    plt.xlabel('False Positive Rate')
    plt.ylabel('True Positive Rate')
    plt.title('ROC curve of ' + target_case_dic.get(target_case).capitalize() + ' Cases')
    plt.plot([0, 1], [0, 1], color='navy', lw=2, linestyle='--')
    plt.legend(loc='center left', bbox_to_anchor=(1,0.5))
    plt.show()


    total_roc_aucs = total_roc_aucs.append(total_roc_auc, ignore_index=True)
    total_roc_aucs['result_train_filename'][i] = result_train_filename
    total_roc_aucs['RandomState'][i] = random_state_val

    if _DEBUG:
        total_roc_aucs.head()
```

```
random state :   12345
9398
result_train_filename: data/train_test_data_ischemic heart_2013_20210519_105327.cs
v
total count : 9398, df_train count : 7518, df_test count : 1880
(7518, 38)
(1880, 38)
('Logistic Regression', LogisticRegression())
[2021-05-19 10:53:27.390393] Start parameter search for model 'Logistic Regressio
n'
[2021-05-19 10:53:29.517668] Finish parameter search for model 'Logistic Regressio
n' (time: 2 seconds)

=== train : best params ===
{'C': 100.0, 'penalty': 'l2', 'solver': 'lbfgs'}
=== train : best score : roc_auc ===
0.8013898864244229



('Random Forest', RandomForestClassifier())
[2021-05-19 10:53:29.518666] Start parameter search for model 'Random Forest'
[2021-05-19 10:53:34.390646] Finish parameter search for model 'Random Forest' (ti
me: 4 seconds)

=== train : best params ===
{'criterion': 'gini', 'max_depth': 10, 'max_features': 'auto', 'n_estimators': 10
0}
=== train : best score : roc_auc ===
0.8091220662931374



('Extreme Gradient Boosting)', XGBClassifier(base_score=None, booster=None, colsam
ple_bylevel=None,
              colsample_bynode=None, colsample_bytree=None, gamma=None,
              gpu_id=None, importance_type='gain', interaction_constraints=None,
              learning_rate=None, max_delta_step=None, max_depth=None,
              min_child_weight=None, missing=nan, monotone_constraints=None,
              n_estimators=100, n_jobs=None, num_parallel_tree=None,
              random_state=None, reg_alpha=None, reg_lambda=None,
              scale_pos_weight=None, subsample=None, tree_method=None,
              validate_parameters=None, verbosity=None))
[2021-05-19 10:53:34.391644] Start parameter search for model 'Extreme Gradient Bo
osting)'
[2021-05-19 10:54:01.929936] Finish parameter search for model 'Extreme Gradient B
oosting)' (time: 27 seconds)

=== train : best params ===
{'learning_rate': 0.1, 'max_depth': 2, 'n_estimators': 50, 'subsample': 0.7}
=== train : best score : roc_auc ===
0.8077747989852403



('k-Nearest Neighbours)', KNeighborsClassifier())
[2021-05-19 10:54:01.930933] Start parameter search for model 'k-Nearest Neighbour
s)'
[2021-05-19 10:54:09.943460] Finish parameter search for model 'k-Nearest Neighbou
rs)' (time: 8 seconds)

=== train : best params ===
{'n_neighbors': 60, 'weights': 'distance'}
=== train : best score : roc_auc ===
```

```
0.7845593979804786


('Decision Tree', DecisionTreeClassifier())
[2021-05-19 10:54:09.943460] Start parameter search for model 'Decision Tree'
[2021-05-19 10:54:10.481329] Finish parameter search for model 'Decision Tree' (ti
me: 0 seconds)

=== train : best params ===
{'criterion': 'entropy', 'max_depth': 5}
=== train : best score : roc_auc ===
0.793042542727846


('Extra Tree', ExtraTreesClassifier())
[2021-05-19 10:54:10.482290] Start parameter search for model 'Extra Tree'
[2021-05-19 10:54:13.529846] Finish parameter search for model 'Extra Tree' (time:
3 seconds)

=== train : best params ===
{'max_features': 'sqrt', 'n_estimators': 100}
=== train : best score : roc_auc ===
0.8002751428844368


('Gradient Boosting', GradientBoostingClassifier())
[2021-05-19 10:54:13.530843] Start parameter search for model 'Gradient Boosting'
[2021-05-19 10:54:27.921353] Finish parameter search for model 'Gradient Boosting'
(time: 14 seconds)

=== train : best params ===
{'learning_rate': 0.075, 'max_depth': 5, 'max_features': 6, 'n_estimators': 100}
=== train : best score : roc_auc ===
0.8094511283531317


('Adaptive Boosting', AdaBoostClassifier())
[2021-05-19 10:54:27.921353] Start parameter search for model 'Adaptive Boosting'
[2021-05-19 10:54:31.379331] Finish parameter search for model 'Adaptive Boosting'
(time: 3 seconds)

=== train : best params ===
{'learning_rate': 0.2, 'n_estimators': 50}
=== train : best score : roc_auc ===
0.8013483468336091


('Suppport Vector Classfier', SVC(probability=True))
[2021-05-19 10:54:31.380328] Start parameter search for model 'Suppport Vector Cla
ssfier'
[2021-05-19 10:58:39.855225] Finish parameter search for model 'Suppport Vector Cl
assfier' (time: 248 seconds)

=== train : best params ===
{'C': 1, 'gamma': 0.1, 'kernel': 'rbf'}
=== train : best score : roc_auc ===
0.7830575299111913


('Neural Network', MLPClassifier())
[2021-05-19 10:58:39.855225] Start parameter search for model 'Neural Network'
```

```
[2021-05-19 11:00:43.546412] Finish parameter search for model 'Neural Network' (t
ime: 123 seconds)

=== train : best params ===
{'activation': 'logistic', 'alpha': 0.05, 'hidden_layer_sizes': (50,), 'learning_r
ate': 'constant'}
=== train : best score : roc_auc ===
0.8067661104260182



Confusion Matrix:
[[824  57]
 [425 574]]

Classification Report:
              precision    recall  f1-score   support

         0.0       0.66      0.94      0.77       881
         1.0       0.91      0.57      0.70       999

    accuracy                           0.74      1880
   macro avg       0.78      0.75      0.74      1880
weighted avg       0.79      0.74      0.74      1880
```

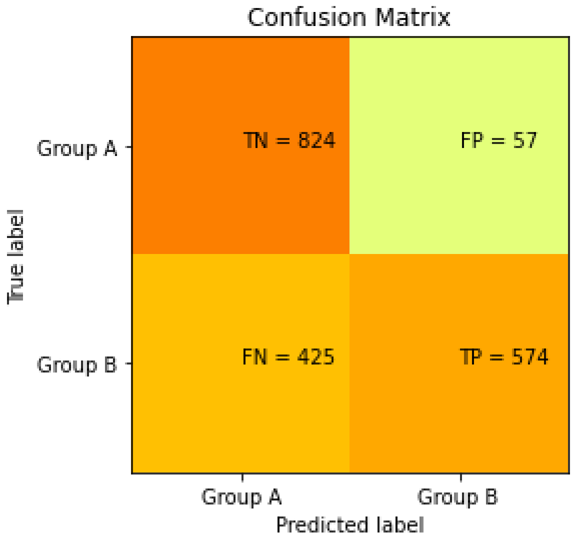


```
AUC Score : 0.7549376845631103

Accuracy Of the Model(LogisticRegression): 0.7436170212765958
```

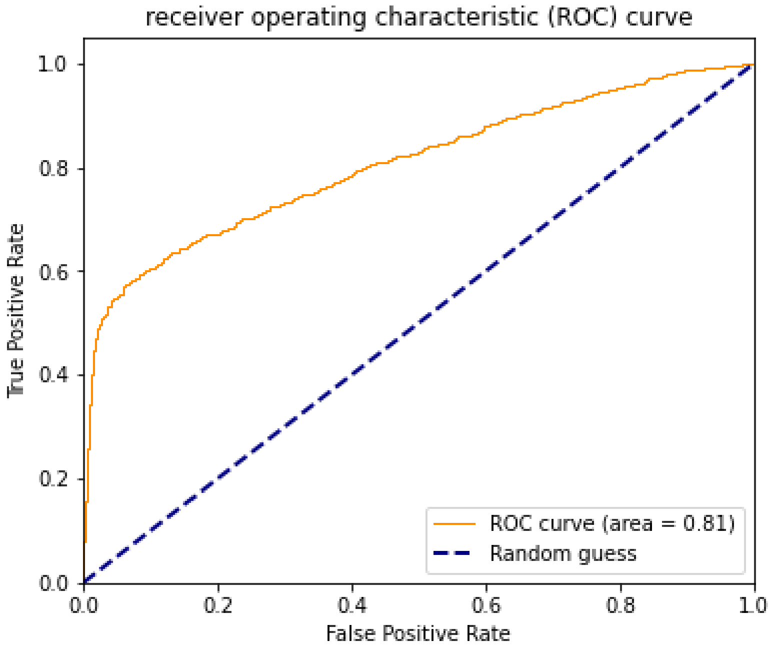

ROC_AUC Score of the model(LogisticRegression): 0.8077669042481755

Confusion Matrix:
[[811 70]
 [413 586]]

Classification Report:

	precision	recall	f1-score	support
0.0	0.66	0.92	0.77	881
1.0	0.89	0.59	0.71	999
accuracy			0.74	1880
macro avg	0.78	0.75	0.74	1880
weighted avg	0.79	0.74	0.74	1880

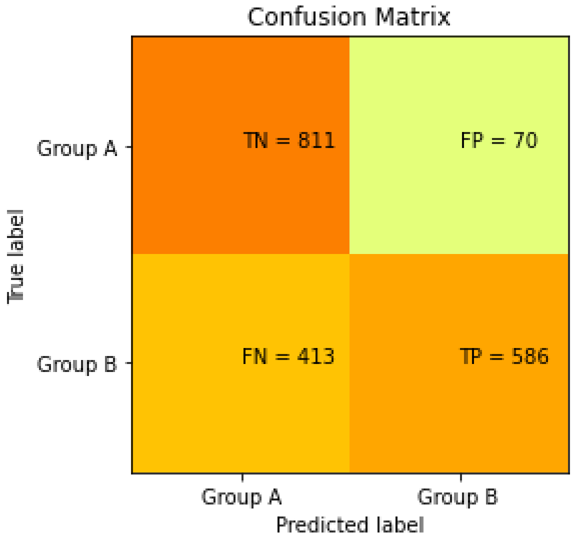

AUC Score : 0.7535657110004442

Accuracy Of the Model(RandomForestClassifier): 0.7430851063829788

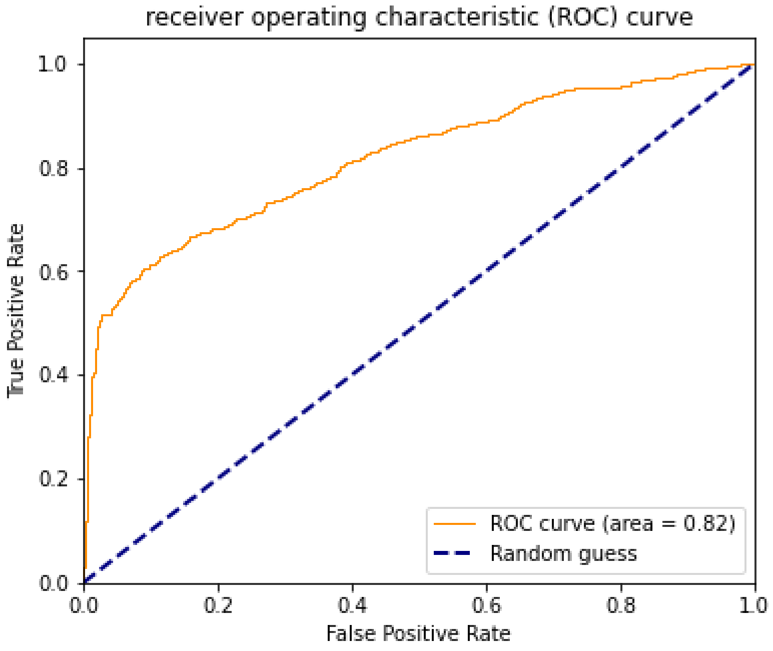

ROC_AUC Score of the model(RandomForestClassifier): 0.818490454131771

```
Confusion Matrix:
[[817  64]
 [440 559]]

Classification Report:
              precision    recall  f1-score   support

         0.0       0.65      0.93      0.76       881
         1.0       0.90      0.56      0.69       999

    accuracy                           0.73      1880
   macro avg       0.77      0.74      0.73      1880
weighted avg       0.78      0.73      0.72      1880
```

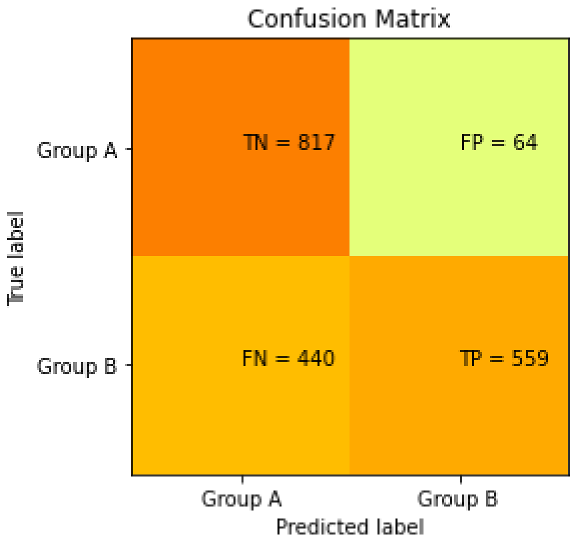

AUC Score : 0.7434574188263179

Accuracy Of the Model(XGBClassifier): 0.7319148936170212

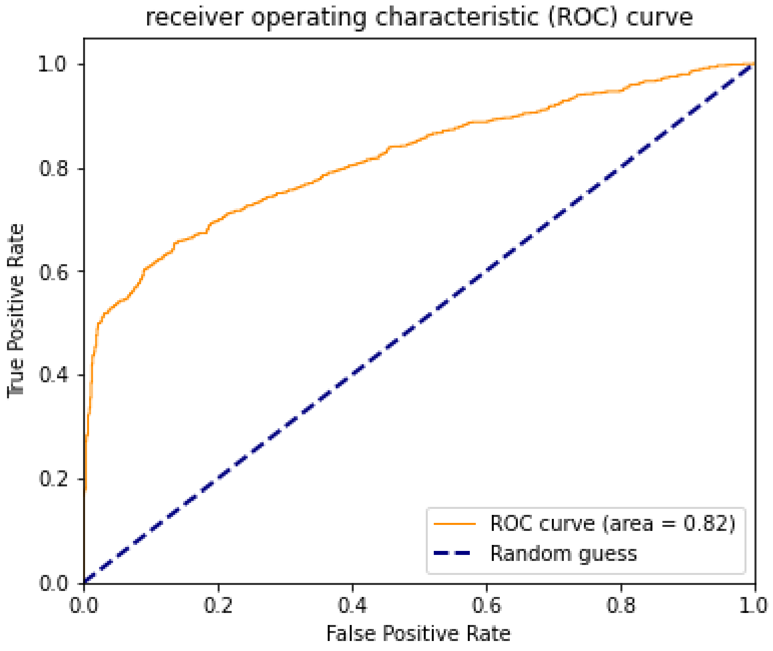


```
ROC_AUC Score of the model(XGBClassifier): 0.8169003282510661


Confusion Matrix:
[[795  86]
 [456 543]]

Classification Report:
              precision    recall  f1-score   support

         0.0       0.64      0.90      0.75       881
         1.0       0.86      0.54      0.67       999

    accuracy                           0.71      1880
   macro avg       0.75      0.72      0.71      1880
weighted avg       0.76      0.71      0.70      1880
```

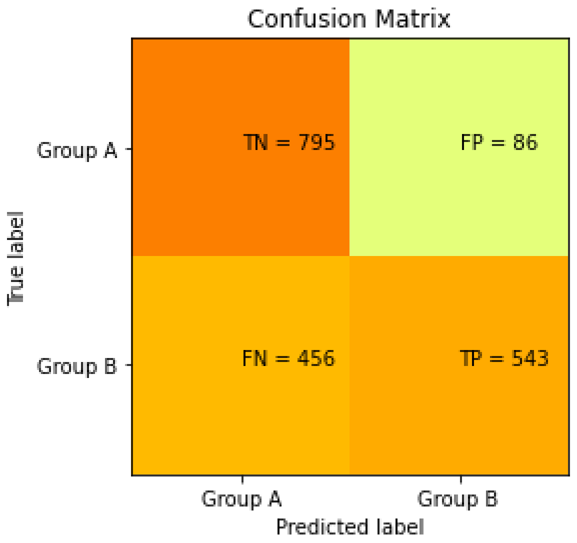


```
AUC Score : 0.7229635992405573

Accuracy Of the Model(KNeighborsClassifier): 0.7117021276595744
```

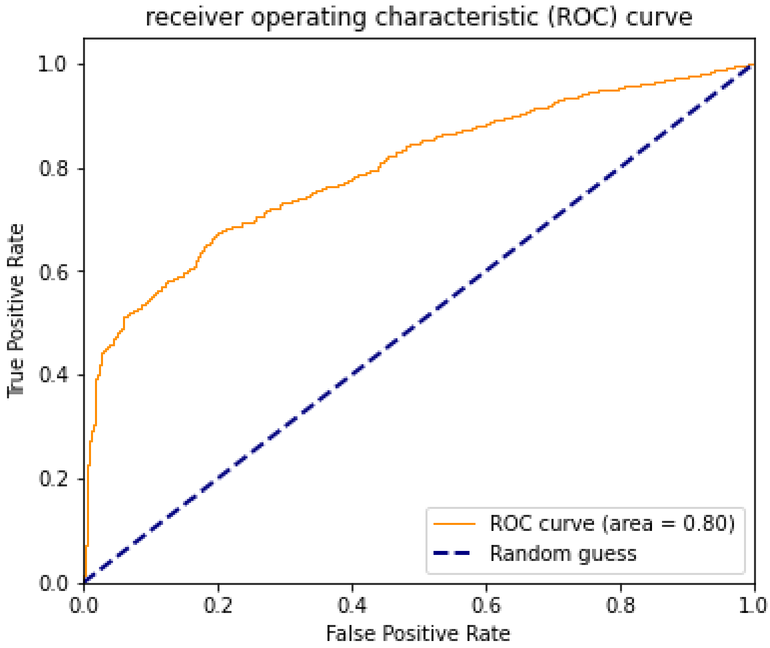


```
ROC_AUC Score of the model(KNeighborsClassifier): 0.7975535126499994


Confusion Matrix:
[[778 103]
 [377 622]]

Classification Report:
              precision    recall  f1-score   support

         0.0       0.67      0.88      0.76       881
         1.0       0.86      0.62      0.72       999

    accuracy                           0.74      1880
   macro avg       0.77      0.75      0.74      1880
weighted avg       0.77      0.74      0.74      1880
```

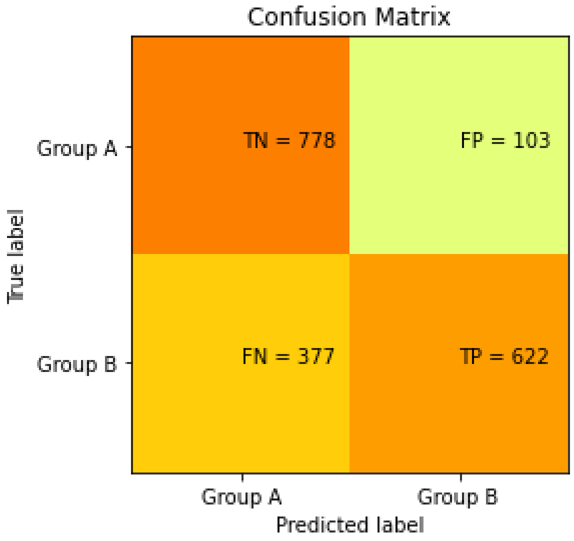


```
AUC Score : 0.7528550116518334

Accuracy Of the Model(DecisionTreeClassifier): 0.7446808510638298
```

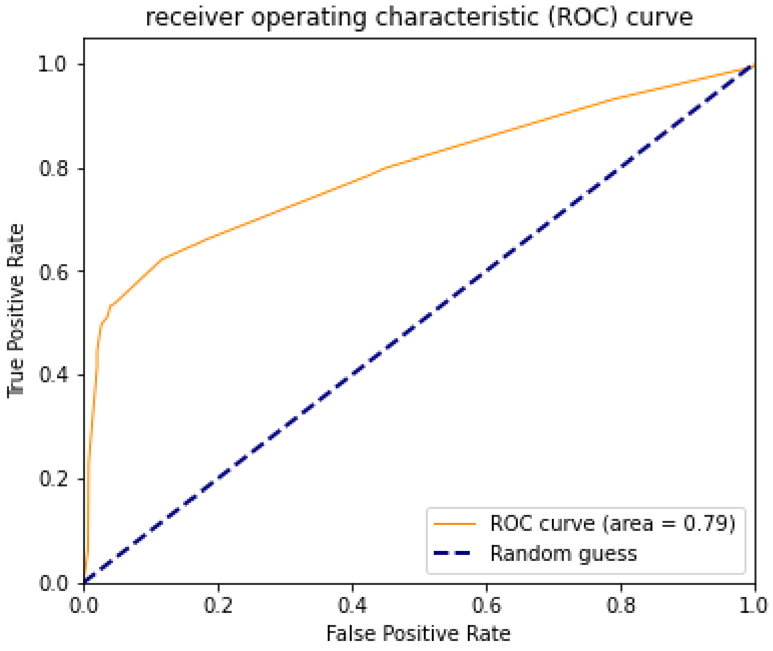

ROC_AUC Score of the model(DecisionTreeClassifier): 0.7919571103453056

```
Confusion Matrix:
[[797  84]
 [416 583]]

Classification Report:
              precision    recall  f1-score   support

         0.0       0.66      0.90      0.76       881
         1.0       0.87      0.58      0.70       999

    accuracy                           0.73      1880
   macro avg       0.77      0.74      0.73      1880
weighted avg       0.77      0.73      0.73      1880
```

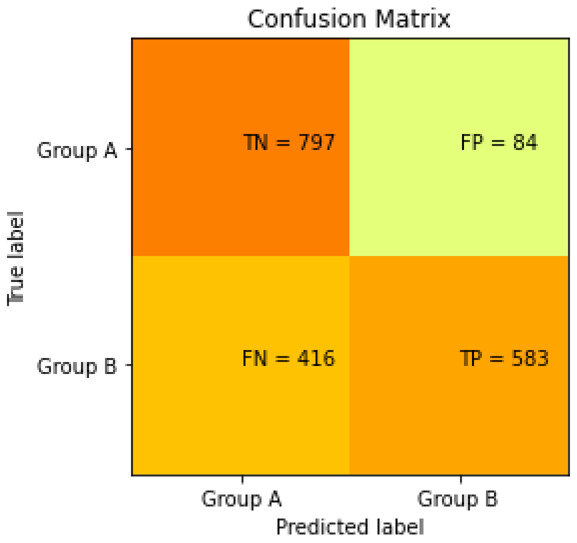

AUC Score : 0.7441186930403731

Accuracy Of the Model(ExtraTreesClassifier): 0.7340425531914894

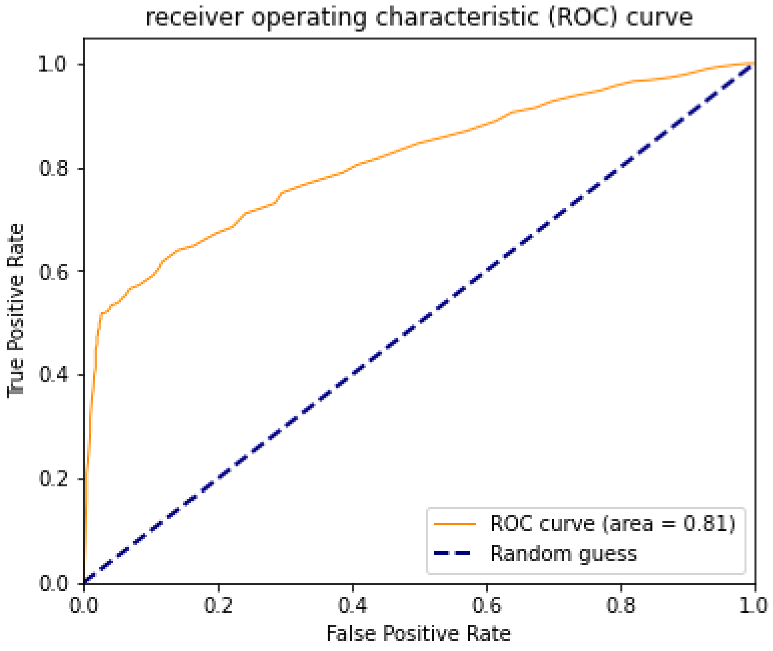


```
ROC_AUC Score of the model(ExtraTreesClassifier): 0.8117021675477976


Confusion Matrix:
[[793  88]
 [382 617]]

Classification Report:
              precision    recall  f1-score   support

         0.0       0.67      0.90      0.77       881
         1.0       0.88      0.62      0.72       999

    accuracy                           0.75      1880
   macro avg       0.78      0.76      0.75      1880
weighted avg       0.78      0.75      0.75      1880
```

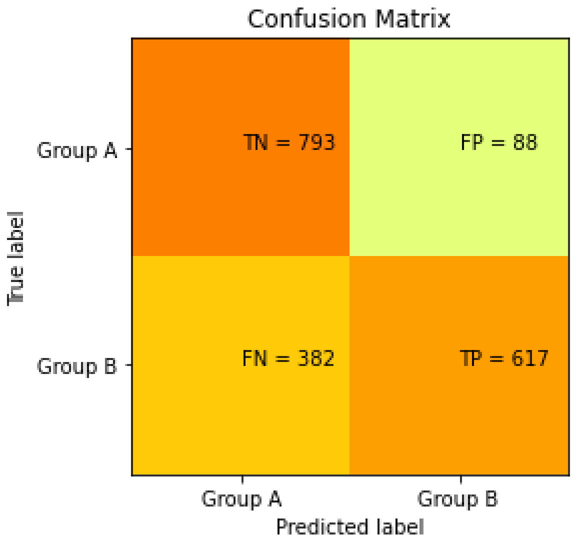


```
AUC Score : 0.7588655624977986

Accuracy Of the Model(GradientBoostingClassifier): 0.75
```

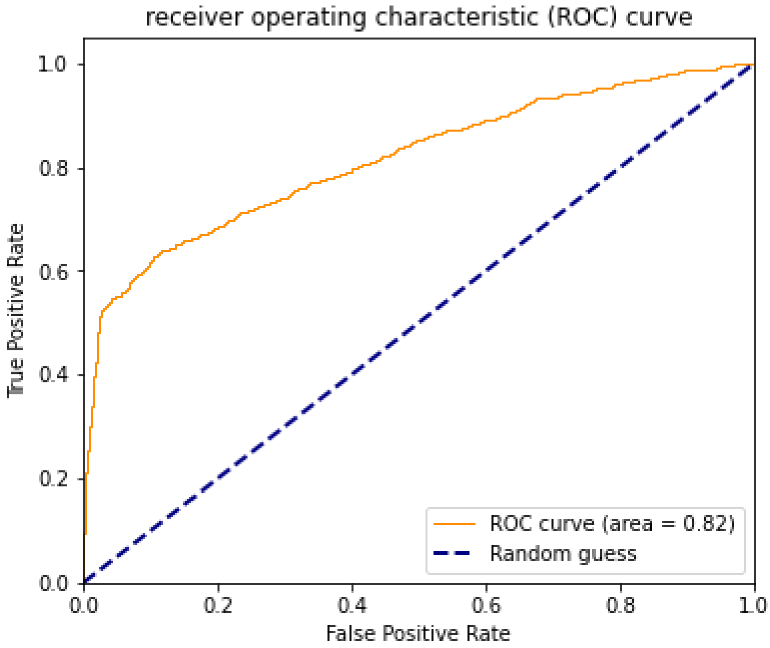

ROC_AUC Score of the model(GradientBoostingClassifier): 0.8172303972530988

```
Confusion Matrix:
[[827  54]
 [453 546]]

Classification Report:
              precision    recall  f1-score   support

         0.0       0.65      0.94      0.77       881
         1.0       0.91      0.55      0.68       999

    accuracy                           0.73      1880
   macro avg       0.78      0.74      0.72      1880
weighted avg       0.79      0.73      0.72      1880
```

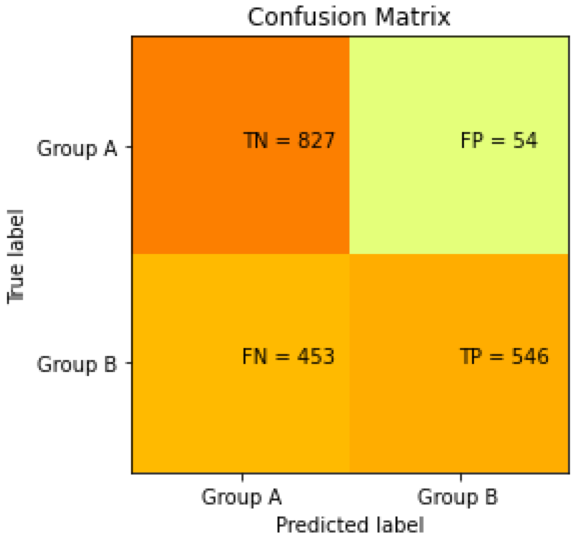

AUC Score : 0.7426262812187898

Accuracy Of the Model(AdaBoostClassifier): 0.7303191489361702

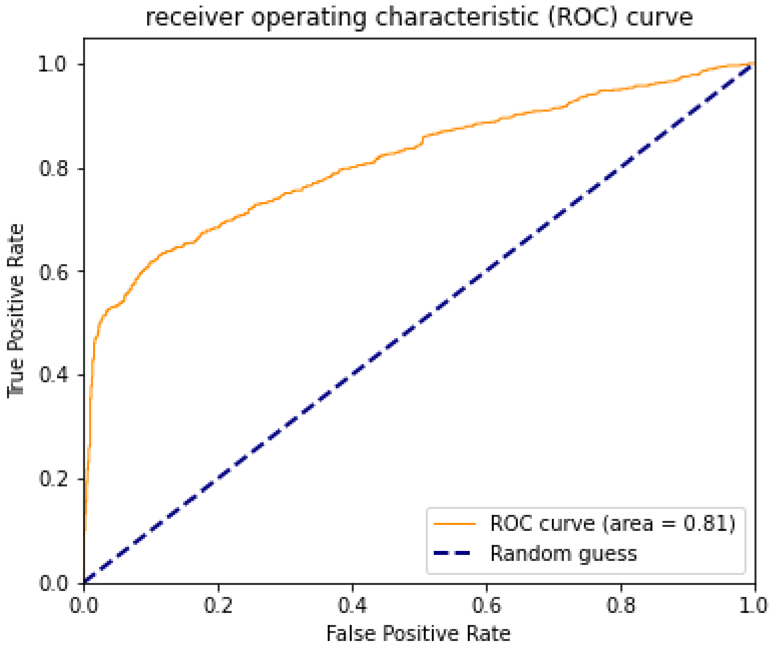

ROC_AUC Score of the model(AdaBoostClassifier): 0.8124441126711275

Confusion Matrix:
[[702 179]
 [348 651]]

Classification Report:

	precision	recall	f1-score	support
0.0	0.67	0.80	0.73	881
1.0	0.78	0.65	0.71	999
accuracy			0.72	1880
macro avg	0.73	0.72	0.72	1880
weighted avg	0.73	0.72	0.72	1880

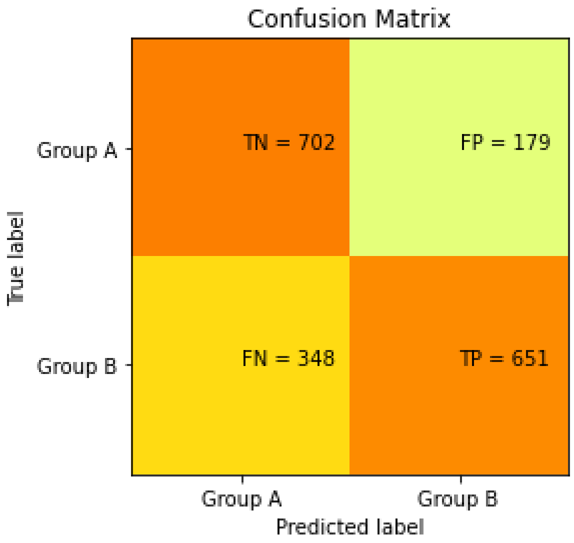

AUC Score : 0.7242367225341118

Accuracy Of the Model(SVC): 0.7196808510638298

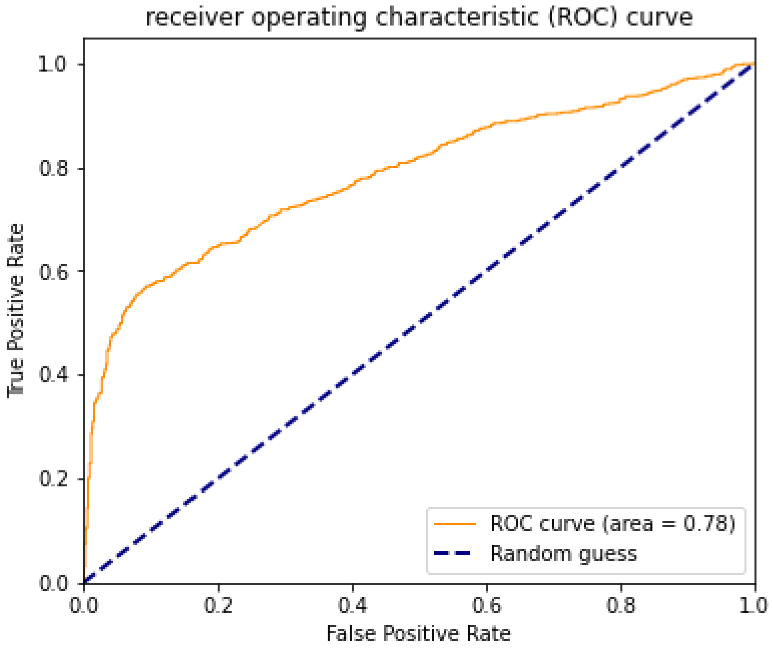


```
ROC_AUC Score of the model(SVC): 0.7848427314942639


Confusion Matrix:
[[801  80]
 [404 595]]

Classification Report:
              precision    recall  f1-score   support

         0.0       0.66      0.91      0.77       881
         1.0       0.88      0.60      0.71       999

    accuracy                           0.74      1880
   macro avg       0.77      0.75      0.74      1880
weighted avg       0.78      0.74      0.74      1880
```

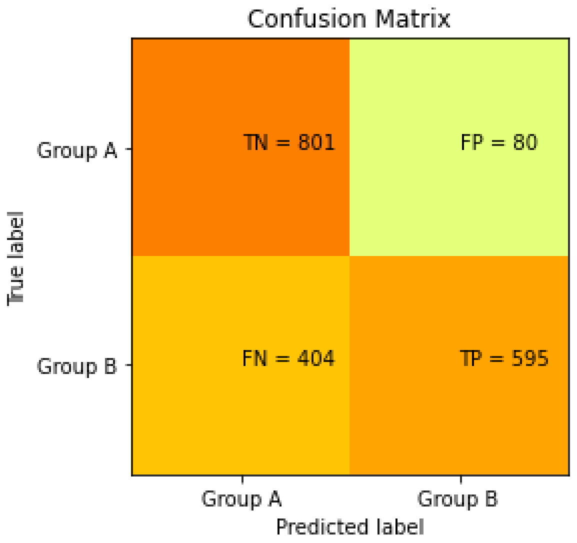


```
AUC Score : 0.7523948466059703

Accuracy Of the Model(MLPClassifier): 0.7425531914893617
```

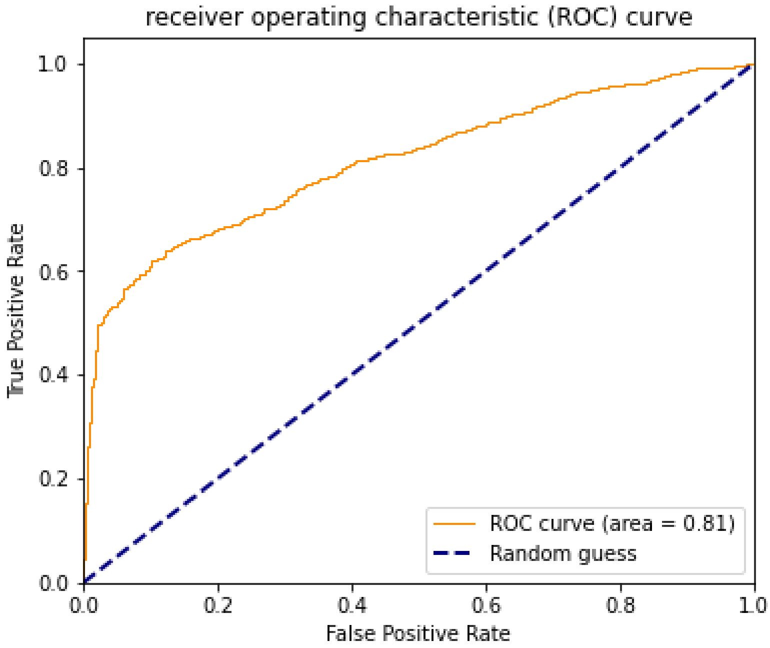

ROC_AUC Score of the model(MLPClassifier): 0.8131468585498098

[('GradientBoostingClassifier', 0.75), ('DecisionTreeClassifier', 0.7446808510638298), ('LogisticRegression', 0.7436170212765958), ('RandomForestClassifier', 0.7430851063829788), ('MLPClassifier', 0.7425531914893617), ('ExtraTreesClassifier', 0.7340425531914894), ('XGBClassifier', 0.7319148936170212), ('AdaBoostClassifier', 0.7303191489361702), ('SVC', 0.7196808510638298), ('KNeighborsClassifier', 0.7117021276595744)]

sorted_total_auc:
[('RandomForestClassifier', 0.818490454131771), ('GradientBoostingClassifier', 0.8172303972530988), ('XGBClassifier', 0.8169003282510661), ('MLPClassifier', 0.8131468585498098), ('AdaBoostClassifier', 0.8124441126711275), ('ExtraTreesClassifier', 0.8117021675477976), ('LogisticRegression', 0.8077669042481755), ('KNeighborsClassifier', 0.797553512649994), ('DecisionTreeClassifier', 0.7919571103453056), ('SVC', 0.7848427314942639)]

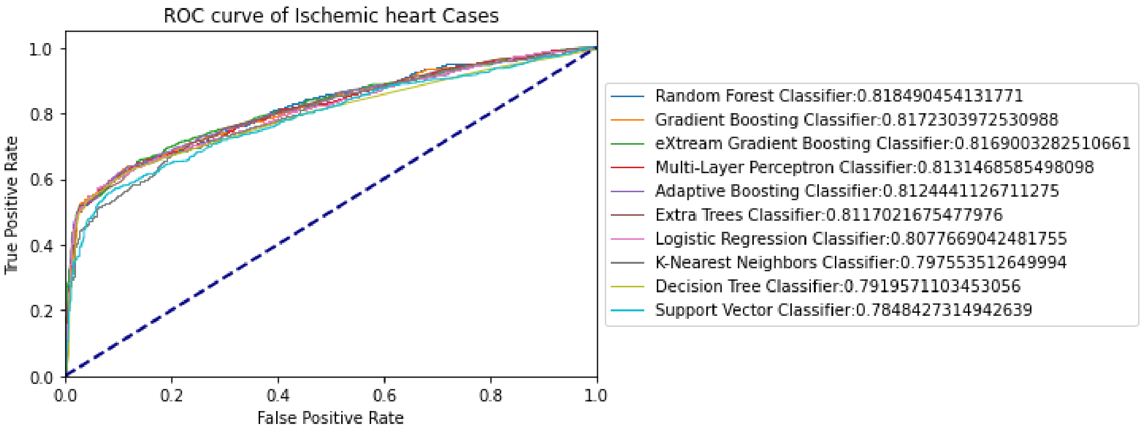

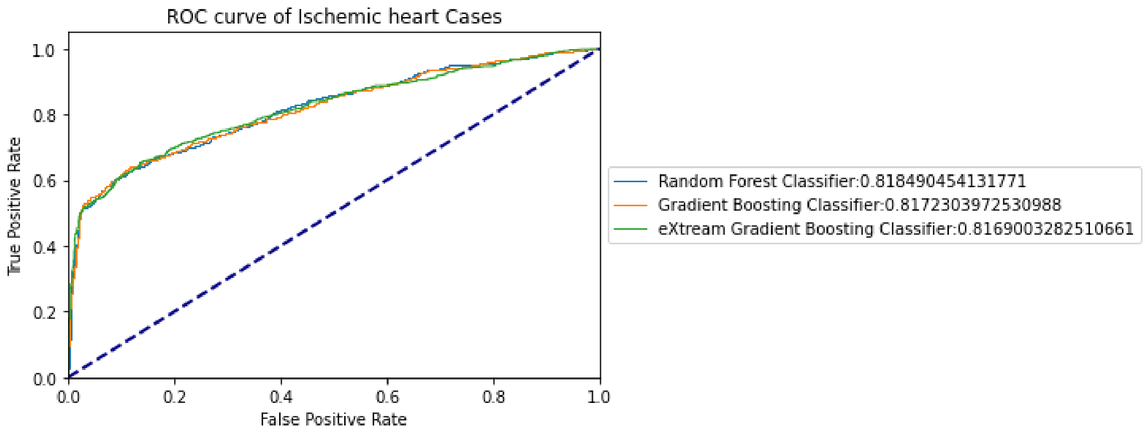
ROC curve of Ischemic heart Cases
True Positive Rate
False Positive Rate
0.0
0.2
0.4
0.6
0.8
1.0
Random Forest Classifier:0.818490454131771
Gradient Boosting Classifier:0.8172303972530988
eXtream Gradient Boosting Classifier:0.8169003282510661

```
random state :   12346
9398
result_train_filename: data/train_test_data_ischemic heart_2013_20210519_110051.cs
v
total count : 9398, df_train count : 7518, df_test count : 1880
(7518, 38)
(1880, 38)
('Logistic Regression', LogisticRegression())
[2021-05-19 11:00:51.348752] Start parameter search for model 'Logistic Regressio
n'
[2021-05-19 11:00:51.726190] Finish parameter search for model 'Logistic Regressio
n' (time: 0 seconds)

=== train : best params ===
{'C': 1000.0, 'penalty': 'l2', 'solver': 'lbfgs'}
=== train : best score : roc_auc ===
0.8032183070908039


('Random Forest', RandomForestClassifier())
[2021-05-19 11:00:51.727193] Start parameter search for model 'Random Forest'
[2021-05-19 11:00:56.036221] Finish parameter search for model 'Random Forest' (ti
me: 4 seconds)

=== train : best params ===
{'criterion': 'gini', 'max_depth': 8, 'max_features': 'auto', 'n_estimators': 70}
=== train : best score : roc_auc ===
0.8103526354470634


('Extreme Gradient Boosting)', XGBClassifier(base_score=None, booster=None, colsam
ple_bylevel=None,
              colsample_bynode=None, colsample_bytree=None, gamma=None,
              gpu_id=None, importance_type='gain', interaction_constraints=None,
              learning_rate=None, max_delta_step=None, max_depth=None,
              min_child_weight=None, missing=nan, monotone_constraints=None,
              n_estimators=100, n_jobs=None, num_parallel_tree=None,
              random_state=None, reg_alpha=None, reg_lambda=None,
              scale_pos_weight=None, subsample=None, tree_method=None,
              validate_parameters=None, verbosity=None))
[2021-05-19 11:00:56.036221] Start parameter search for model 'Extreme Gradient Bo
osting)'
[2021-05-19 11:01:23.200561] Finish parameter search for model 'Extreme Gradient B
oosting)' (time: 27 seconds)

=== train : best params ===
{'learning_rate': 0.05, 'max_depth': 5, 'n_estimators': 40, 'subsample': 0.7}
=== train : best score : roc_auc ===
0.8119026356707519


('k-Nearest Neighbours)', KNeighborsClassifier())
[2021-05-19 11:01:23.201558] Start parameter search for model 'k-Nearest Neighbour
s)'
[2021-05-19 11:01:31.160852] Finish parameter search for model 'k-Nearest Neighbou
rs)' (time: 7 seconds)

=== train : best params ===
{'n_neighbors': 60, 'weights': 'distance'}
=== train : best score : roc_auc ===
0.790066638727071
```

```
('Decision Tree', DecisionTreeClassifier())
[2021-05-19 11:01:31.161853] Start parameter search for model 'Decision Tree'
[2021-05-19 11:01:31.720567] Finish parameter search for model 'Decision Tree' (ti
me: 0 seconds)

=== train : best params ===
{'criterion': 'gini', 'max_depth': 4}
=== train : best score : roc_auc ===
0.7928815624161083


('Extra Tree', ExtraTreesClassifier())
[2021-05-19 11:01:31.720567] Start parameter search for model 'Extra Tree'
[2021-05-19 11:01:34.725010] Finish parameter search for model 'Extra Tree' (time:
3 seconds)

=== train : best params ===
{'max_features': 'auto', 'n_estimators': 100}
=== train : best score : roc_auc ===
0.8036321024363333


('Gradient Boosting', GradientBoostingClassifier())
[2021-05-19 11:01:34.725010] Start parameter search for model 'Gradient Boosting'
[2021-05-19 11:01:49.721747] Finish parameter search for model 'Gradient Boosting'
(time: 14 seconds)

=== train : best params ===
{'learning_rate': 0.075, 'max_depth': 5, 'max_features': 6, 'n_estimators': 50}
=== train : best score : roc_auc ===
0.8114527832159587


('Adaptive Boosting', AdaBoostClassifier())
[2021-05-19 11:01:49.722743] Start parameter search for model 'Adaptive Boosting'
[2021-05-19 11:01:53.305133] Finish parameter search for model 'Adaptive Boosting'
(time: 3 seconds)

=== train : best params ===
{'learning_rate': 0.2, 'n_estimators': 100}
=== train : best score : roc_auc ===
0.8034488701776312


('Suppport Vector Classfier', SVC(probability=True))
[2021-05-19 11:01:53.305133] Start parameter search for model 'Suppport Vector Cla
ssfier'
[2021-05-19 11:05:55.956187] Finish parameter search for model 'Suppport Vector Cl
assfier' (time: 242 seconds)

=== train : best params ===
{'C': 1, 'gamma': 0.1, 'kernel': 'rbf'}
=== train : best score : roc_auc ===
0.7831378982598766


('Neural Network', MLPClassifier())
[2021-05-19 11:05:55.958183] Start parameter search for model 'Neural Network'
[2021-05-19 11:08:25.745636] Finish parameter search for model 'Neural Network' (t
```

```
ime: 149 seconds)

=== train : best params ===
{'activation': 'logistic', 'alpha': 0.05, 'hidden_layer_sizes': (50,), 'learning_r
ate': 'adaptive'}
=== train : best score : roc_auc ===
0.8073429733094666



Confusion Matrix:
[[813  68]
 [429 570]]

Classification Report:
              precision    recall  f1-score   support

         0.0       0.65      0.92      0.77       881
         1.0       0.89      0.57      0.70       999

    accuracy                           0.74      1880
   macro avg       0.77      0.75      0.73      1880
weighted avg       0.78      0.74      0.73      1880
```

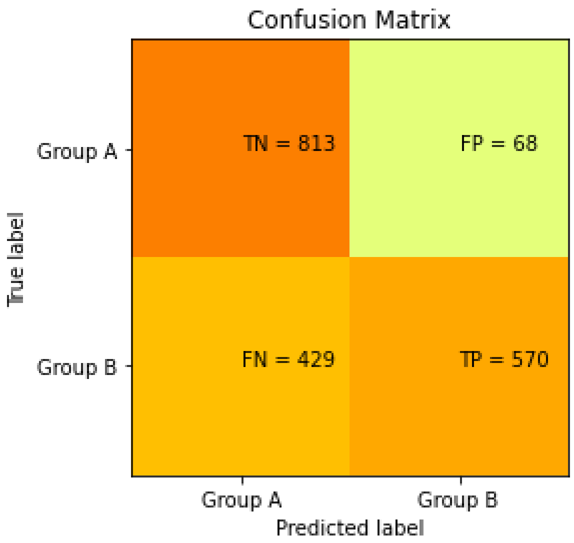


```
AUC Score : 0.7466927767722319

Accuracy Of the Model(LogisticRegression): 0.7356382978723405
```

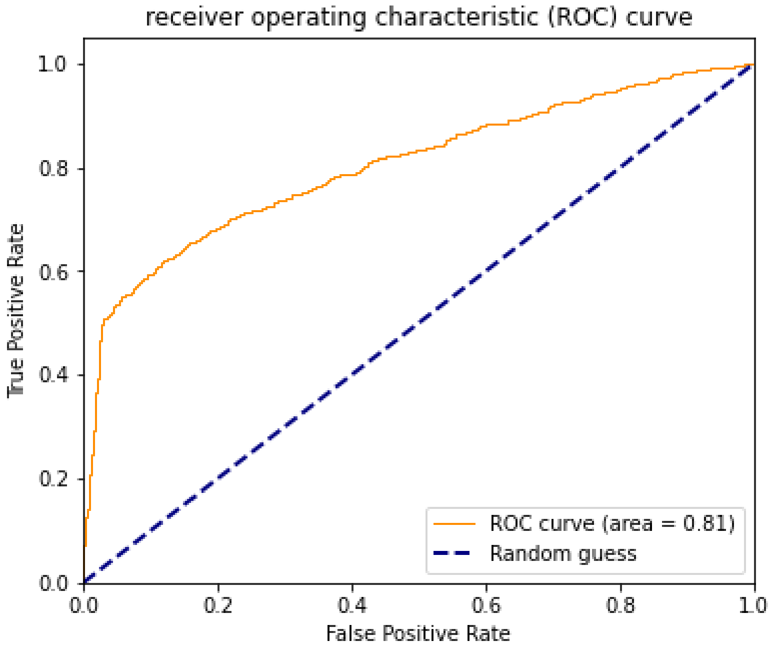


```
ROC_AUC Score of the model(LogisticRegression): 0.8056296932573891


Confusion Matrix:
[[791  90]
 [402 597]]

Classification Report:
              precision    recall  f1-score   support

         0.0       0.66      0.90      0.76       881
         1.0       0.87      0.60      0.71       999

    accuracy                           0.74      1880
   macro avg       0.77      0.75      0.74      1880
weighted avg       0.77      0.74      0.73      1880
```

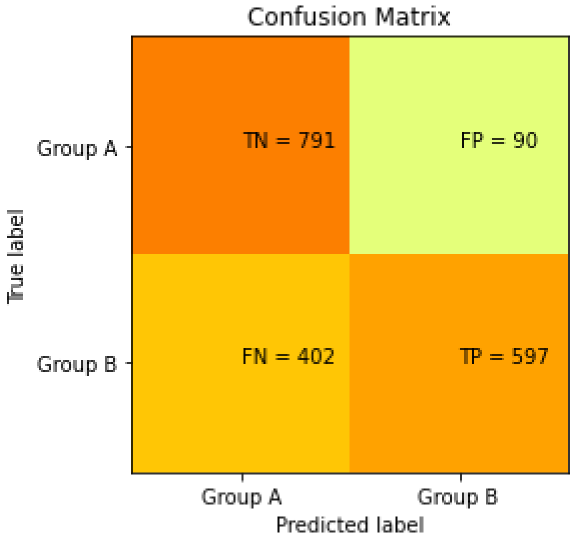

AUC Score : 0.7477204787079929

Accuracy Of the Model(RandomForestClassifier): 0.7382978723404255

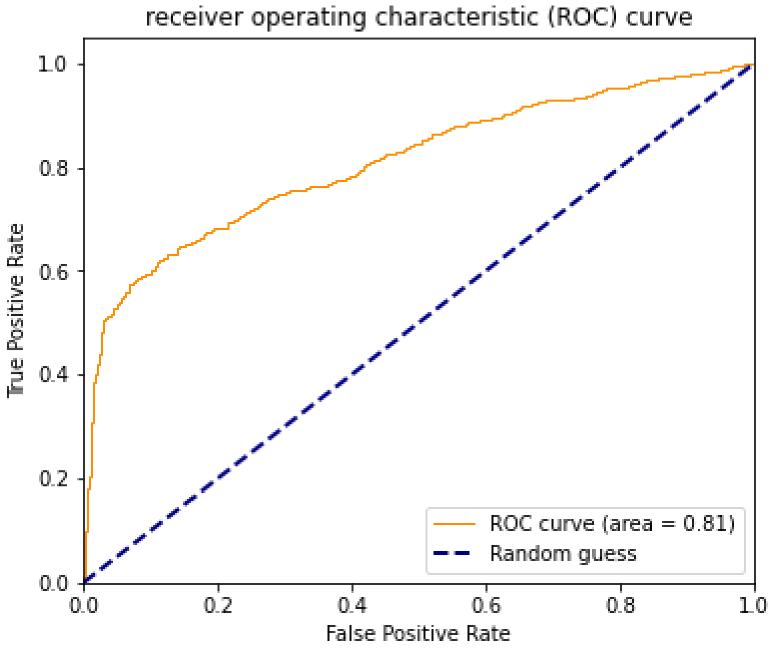

```
ROC_AUC Score of the model(RandomForestClassifier): 0.8108608040503613


Confusion Matrix:
[[822  59]
 [424 575]]

Classification Report:
              precision    recall  f1-score   support

         0.0       0.66      0.93      0.77       881
         1.0       0.91      0.58      0.70       999

    accuracy                           0.74      1880
   macro avg       0.78      0.75      0.74      1880
weighted avg       0.79      0.74      0.74      1880
```

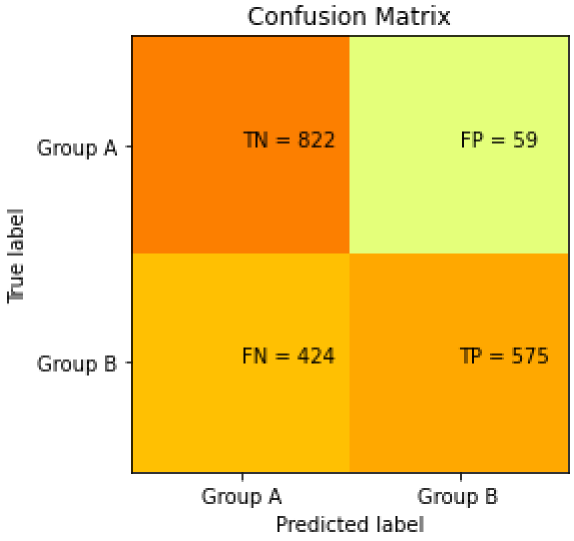


```
AUC Score : 0.7543031112838151

Accuracy Of the Model(XGBClassifier): 0.7430851063829788
```

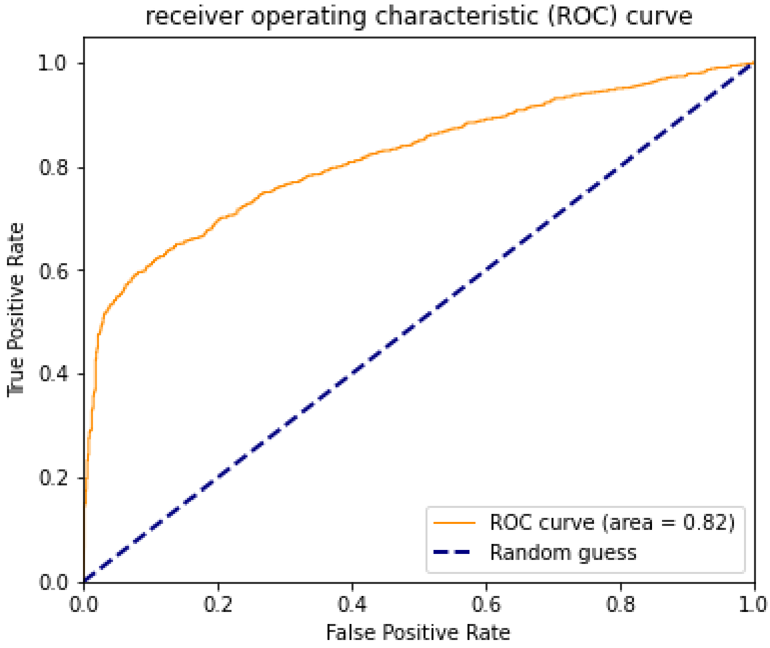

```
ROC_AUC Score of the model(XGBClassifier): 0.8165748040889925


Confusion Matrix:
[[807  74]
 [462 537]]

Classification Report:
              precision    recall  f1-score   support

         0.0       0.64      0.92      0.75       881
         1.0       0.88      0.54      0.67       999

    accuracy                           0.71      1880
   macro avg       0.76      0.73      0.71      1880
weighted avg       0.77      0.71      0.71      1880
```

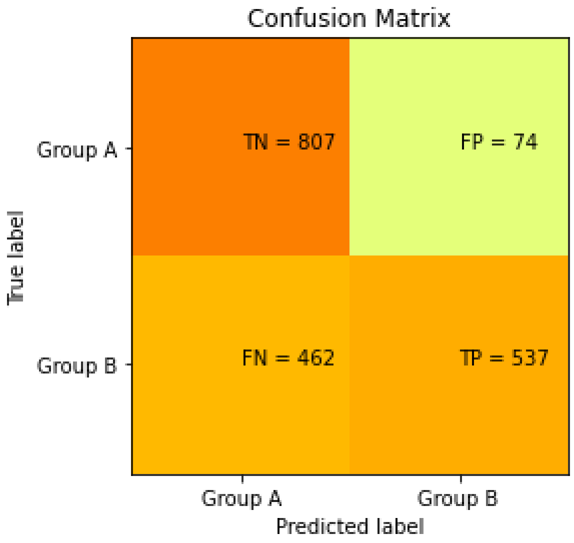


```
AUC Score : 0.7267710389163283

Accuracy Of the Model(KNeighborsClassifier): 0.7148936170212766
```

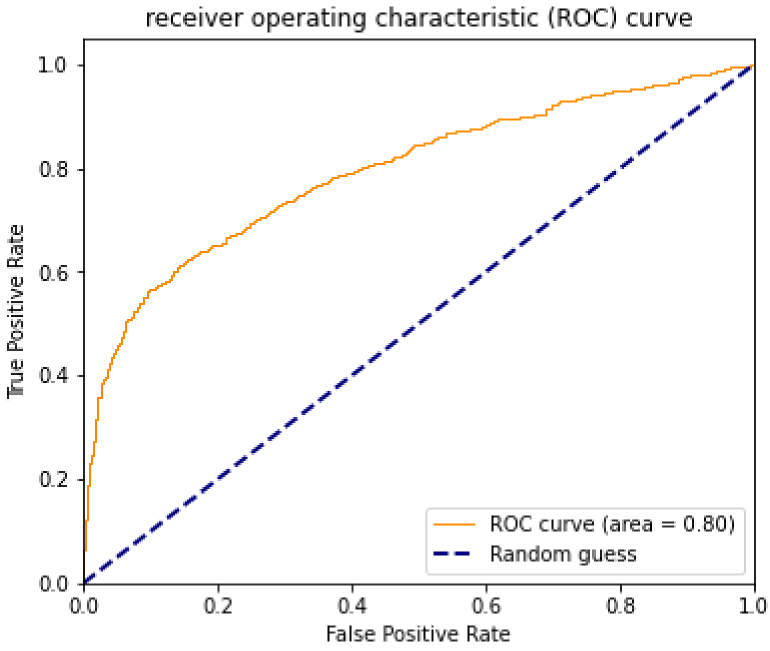

```
ROC_AUC Score of the model(KNeighborsClassifier): 0.7959503203544066


Confusion Matrix:
[[810  71]
 [423 576]]

Classification Report:
              precision    recall  f1-score   support

         0.0       0.66      0.92      0.77       881
         1.0       0.89      0.58      0.70       999

    accuracy                           0.74      1880
   macro avg       0.77      0.75      0.73      1880
weighted avg       0.78      0.74      0.73      1880
```

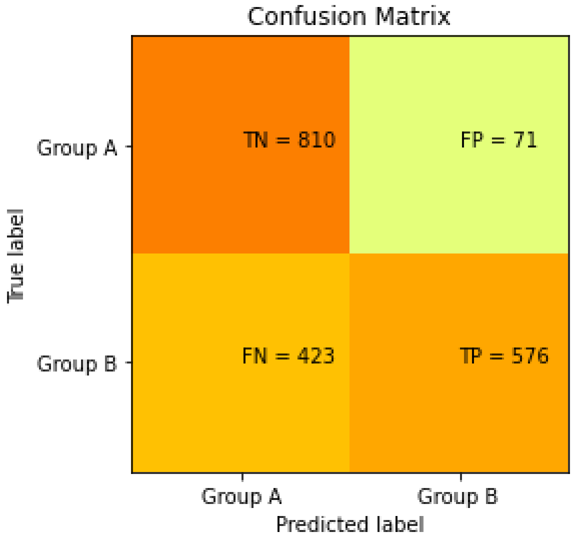


```
AUC Score : 0.7479931691055414

Accuracy Of the Model(DecisionTreeClassifier): 0.7372340425531915
```

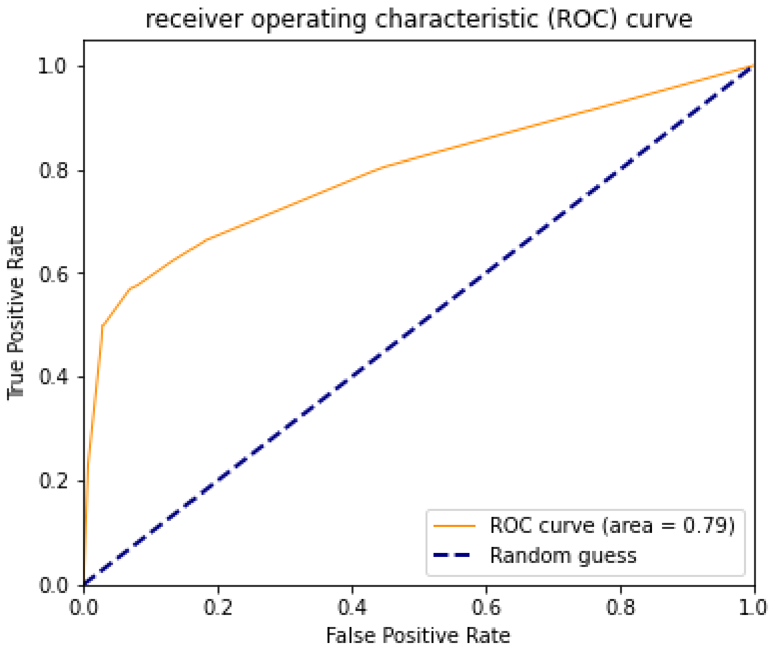

```
ROC_AUC Score of the model(DecisionTreeClassifier): 0.7932495492086866


Confusion Matrix:
[[794  87]
 [418 581]]

Classification Report:
              precision    recall  f1-score   support

         0.0       0.66      0.90      0.76       881
         1.0       0.87      0.58      0.70       999

    accuracy                           0.73      1880
   macro avg       0.76      0.74      0.73      1880
weighted avg       0.77      0.73      0.73      1880
```

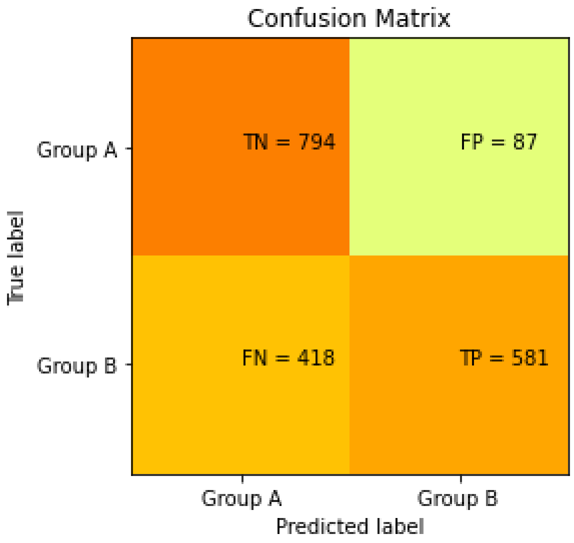


```
AUC Score : 0.7414150813696784

Accuracy Of the Model(ExtraTreesClassifier): 0.7313829787234043
```

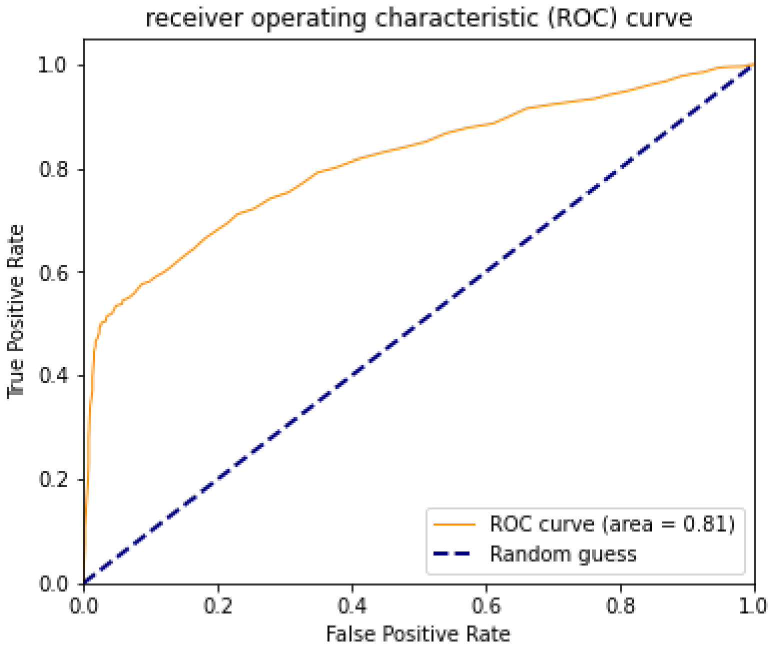

```
ROC_AUC Score of the model(ExtraTreesClassifier): 0.8120731401094624


Confusion Matrix:
[[804  77]
 [411 588]]

Classification Report:
              precision    recall  f1-score   support

         0.0       0.66      0.91      0.77       881
         1.0       0.88      0.59      0.71       999

    accuracy                           0.74      1880
   macro avg       0.77      0.75      0.74      1880
weighted avg       0.78      0.74      0.74      1880
```

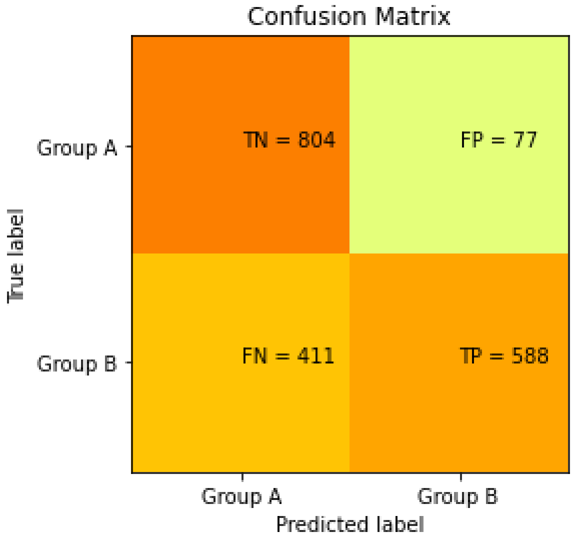


```
AUC Score : 0.7505939537721603

Accuracy Of the Model(GradientBoostingClassifier): 0.7404255319148936
```

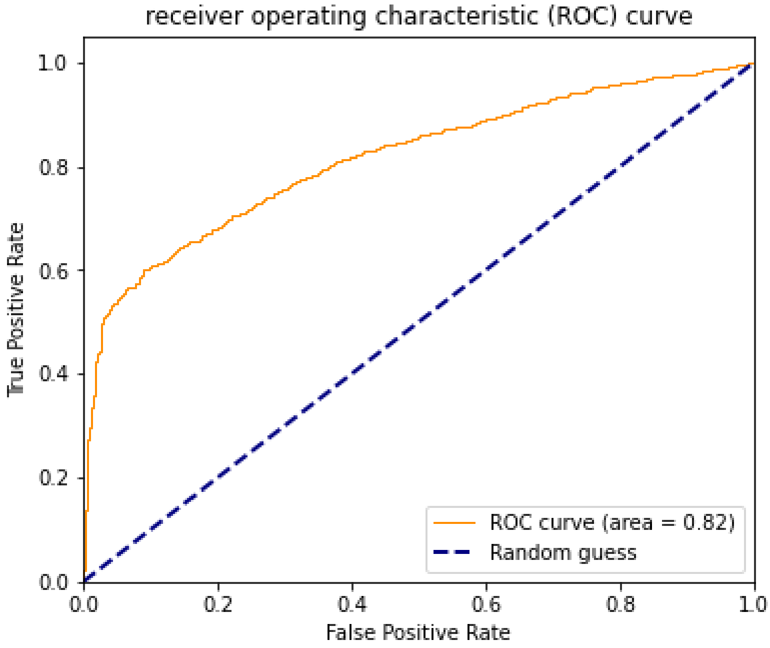

```
ROC_AUC Score of the model(GradientBoostingClassifier): 0.8169702051654377


Confusion Matrix:
[[817  64]
 [425 574]]

Classification Report:
              precision    recall  f1-score   support

         0.0       0.66      0.93      0.77       881
         1.0       0.90      0.57      0.70       999

    accuracy                           0.74      1880
   macro avg       0.78      0.75      0.74      1880
weighted avg       0.79      0.74      0.73      1880
```

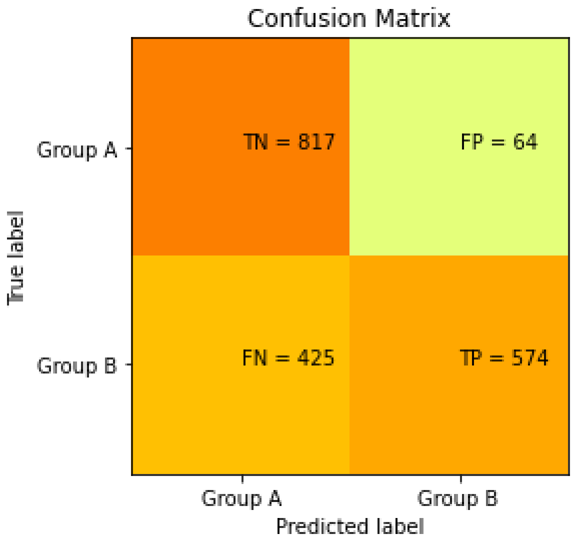


```
AUC Score : 0.7509649263338254

Accuracy Of the Model(AdaBoostClassifier): 0.7398936170212767
```

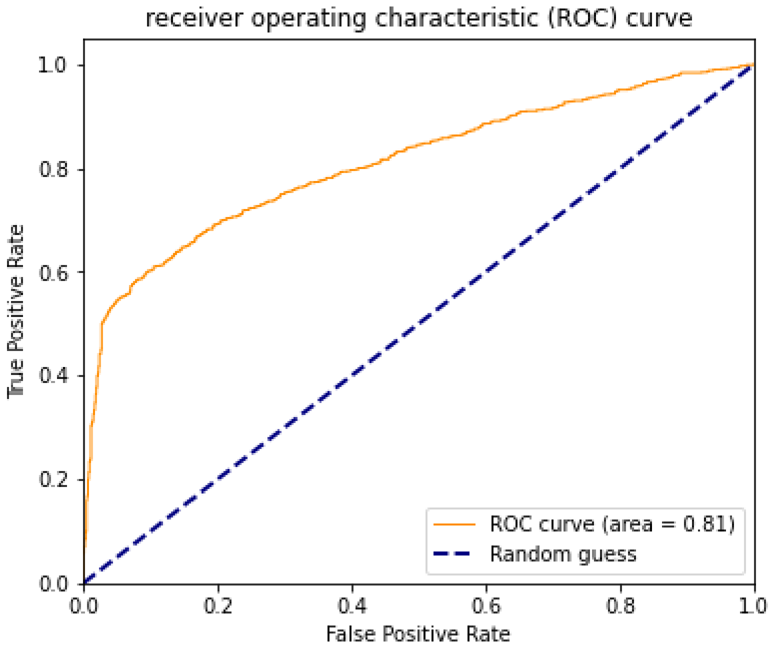

```
ROC_AUC Score of the model(AdaBoostClassifier): 0.8100012611930887


Confusion Matrix:
[[712 169]
 [352 647]]

Classification Report:
              precision    recall  f1-score   support

         0.0       0.67      0.81      0.73       881
         1.0       0.79      0.65      0.71       999

    accuracy                           0.72      1880
   macro avg       0.73      0.73      0.72      1880
weighted avg       0.73      0.72      0.72      1880
```

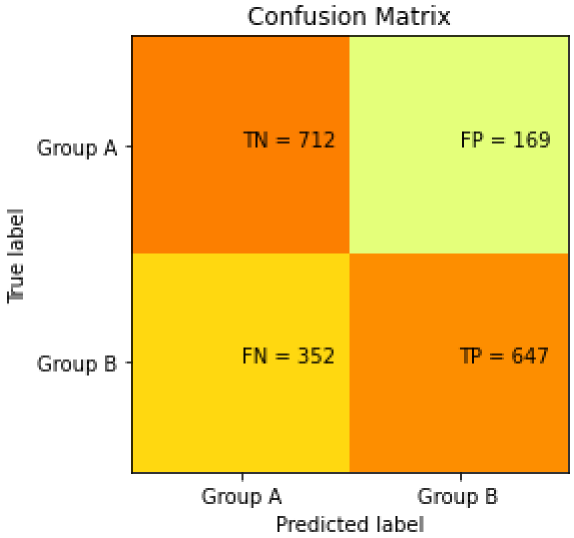


```
AUC Score : 0.7279100894310883

Accuracy Of the Model(SVC): 0.722872340425532
```

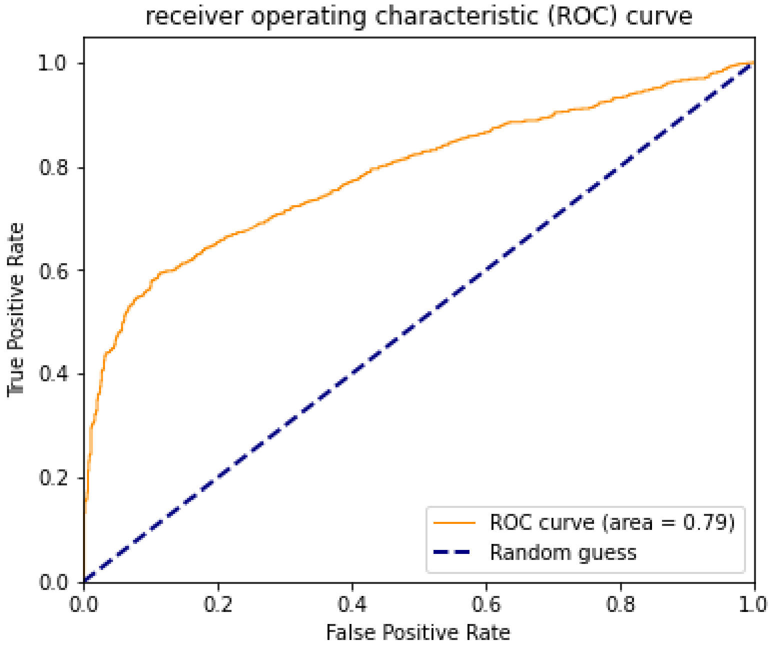

ROC_AUC Score of the model(SVC): 0.7853091456950707

Confusion Matrix:
[[800 81]
 [409 590]]

Classification Report:

	precision	recall	f1-score	support
0.0	0.66	0.91	0.77	881
1.0	0.88	0.59	0.71	999
accuracy			0.74	1880
macro avg	0.77	0.75	0.74	1880
weighted avg	0.78	0.74	0.73	1880

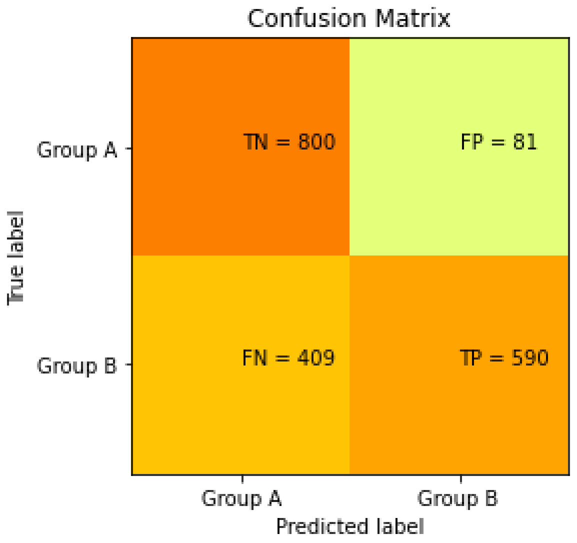

AUC Score : 0.74932480721357

Accuracy Of the Model(MLPClassifier): 0.7393617021276596

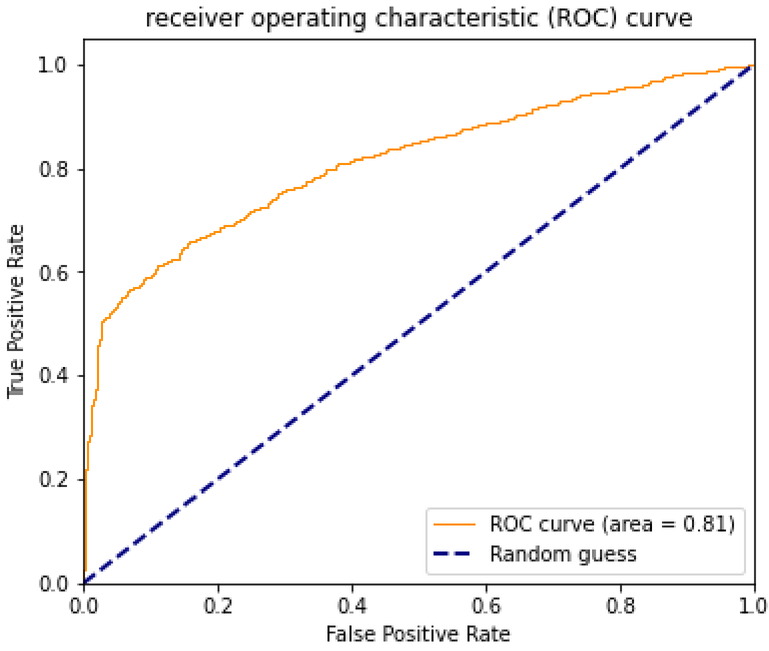

```
ROC_AUC Score of the model(MLPClassifier): 0.8134888577567352

[('XGBClassifier', 0.7430851063829788), ('GradientBoostingClassifier', 0.740425531
9148936), ('AdaBoostClassifier', 0.7398936170212767), ('MLPClassifier', 0.73936170
21276596), ('RandomForestClassifier', 0.7382978723404255), ('DecisionTreeClassifie
r', 0.7372340425531915), ('LogisticRegression', 0.7356382978723405), ('ExtraTreesC
lassifier', 0.7313829787234043), ('SVC', 0.722872340425532), ('KNeighborsClassifie
r', 0.7148936170212766)]


sorted_total_auc:
 [('GradientBoostingClassifier', 0.8169702051654377), ('XGBClassifier', 0.81657480
40889925), ('MLPClassifier', 0.8134888577567352), ('ExtraTreesClassifier', 0.81207
31401094624), ('RandomForestClassifier', 0.8108608040503613), ('AdaBoostClassifie
r', 0.8100012611930887), ('LogisticRegression', 0.8056296932573891), ('KNeighborsC
lassifier', 0.7959503203544066), ('DecisionTreeClassifier', 0.7932495492086866),
('SVC', 0.7853091456950707)]
```

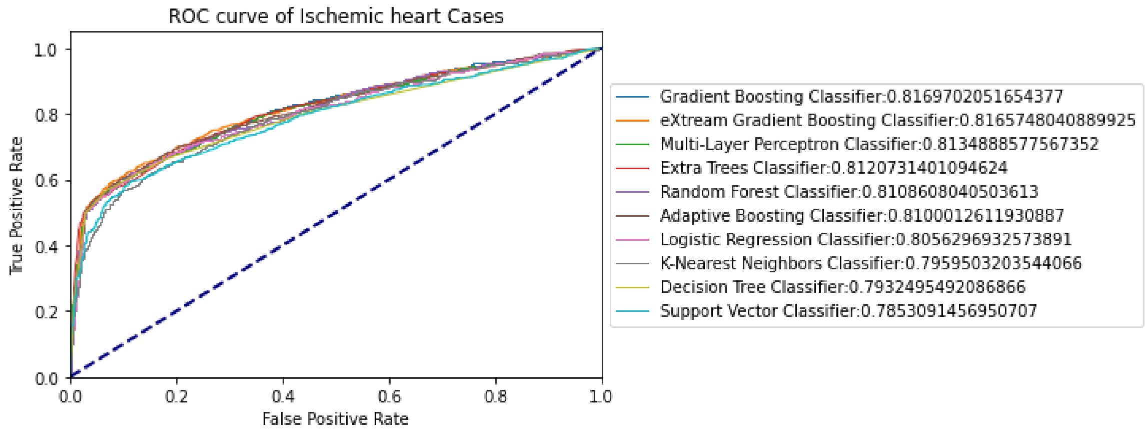

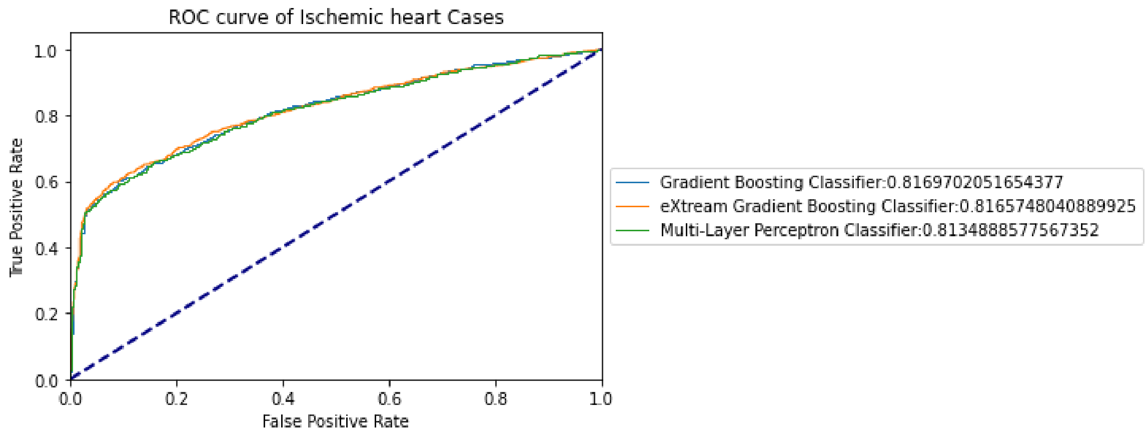

```
random state :   12348
9398
result_train_filename: data/train_test_data_ischemic heart_2013_20210519_110835.cs
v
total count : 9398, df_train count : 7518, df_test count : 1880
(7518, 38)
(1880, 38)
('Logistic Regression', LogisticRegression())
[2021-05-19 11:08:36.186760] Start parameter search for model 'Logistic Regressio
n'
[2021-05-19 11:08:36.535799] Finish parameter search for model 'Logistic Regressio
n' (time: 0 seconds)

=== train : best params ===
{'C': 1, 'penalty': 'l2', 'solver': 'lbfgs'}
=== train : best score : roc_auc ===
0.8041621815804296



('Random Forest', RandomForestClassifier())
[2021-05-19 11:08:36.536796] Start parameter search for model 'Random Forest'
[2021-05-19 11:08:43.531705] Finish parameter search for model 'Random Forest' (ti
me: 6 seconds)

=== train : best params ===
{'criterion': 'gini', 'max_depth': 10, 'max_features': 'auto', 'n_estimators': 90}
=== train : best score : roc_auc ===
0.8096836425943453



('Extreme Gradient Boosting)', XGBClassifier(base_score=None, booster=None, colsam
ple_bylevel=None,
              colsample_bynode=None, colsample_bytree=None, gamma=None,
              gpu_id=None, importance_type='gain', interaction_constraints=None,
              learning_rate=None, max_delta_step=None, max_depth=None,
              min_child_weight=None, missing=nan, monotone_constraints=None,
              n_estimators=100, n_jobs=None, num_parallel_tree=None,
              random_state=None, reg_alpha=None, reg_lambda=None,
              scale_pos_weight=None, subsample=None, tree_method=None,
              validate_parameters=None, verbosity=None))
[2021-05-19 11:08:43.532703] Start parameter search for model 'Extreme Gradient Bo
osting)'
[2021-05-19 11:09:22.744500] Finish parameter search for model 'Extreme Gradient B
oosting)' (time: 39 seconds)

=== train : best params ===
{'learning_rate': 0.05, 'max_depth': 5, 'n_estimators': 50, 'subsample': 0.7}
=== train : best score : roc_auc ===
0.8090344430234235



('k-Nearest Neighbours)', KNeighborsClassifier())
[2021-05-19 11:09:22.746494] Start parameter search for model 'k-Nearest Neighbour
s)'
[2021-05-19 11:09:31.695348] Finish parameter search for model 'k-Nearest Neighbou
rs)' (time: 8 seconds)

=== train : best params ===
{'n_neighbors': 60, 'weights': 'distance'}
=== train : best score : roc_auc ===
0.7868951796500541
```

```
('Decision Tree', DecisionTreeClassifier())
[2021-05-19 11:09:31.695348] Start parameter search for model 'Decision Tree'
[2021-05-19 11:09:32.424041] Finish parameter search for model 'Decision Tree' (ti
me: 0 seconds)

=== train : best params ===
{'criterion': 'entropy', 'max_depth': 4}
=== train : best score : roc_auc ===
0.7936942130477878


('Extra Tree', ExtraTreesClassifier())
[2021-05-19 11:09:32.424041] Start parameter search for model 'Extra Tree'
[2021-05-19 11:09:36.558097] Finish parameter search for model 'Extra Tree' (time:
4 seconds)

=== train : best params ===
{'max_features': 'auto', 'n_estimators': 100}
=== train : best score : roc_auc ===
0.8020707725744347


('Gradient Boosting', GradientBoostingClassifier())
[2021-05-19 11:09:36.560060] Start parameter search for model 'Gradient Boosting'
[2021-05-19 11:09:56.177256] Finish parameter search for model 'Gradient Boosting'
(time: 19 seconds)

=== train : best params ===
{'learning_rate': 0.01, 'max_depth': 8, 'max_features': 6, 'n_estimators': 100}
=== train : best score : roc_auc ===
0.8113414634885574


('Adaptive Boosting', AdaBoostClassifier())
[2021-05-19 11:09:56.177256] Start parameter search for model 'Adaptive Boosting'
[2021-05-19 11:10:00.092865] Finish parameter search for model 'Adaptive Boosting'
(time: 3 seconds)

=== train : best params ===
{'learning_rate': 0.2, 'n_estimators': 50}
=== train : best score : roc_auc ===
0.8020639416400924


('Suppport Vector Classfier', SVC(probability=True))
[2021-05-19 11:10:00.092865] Start parameter search for model 'Suppport Vector Cla
ssfier'
[2021-05-19 11:17:01.484600] Finish parameter search for model 'Suppport Vector Cl
assfier' (time: 421 seconds)

=== train : best params ===
{'C': 1, 'gamma': 0.1, 'kernel': 'rbf'}
=== train : best score : roc_auc ===
0.7817716580827507


('Neural Network', MLPClassifier())
[2021-05-19 11:17:01.484600] Start parameter search for model 'Neural Network'
[2021-05-19 11:19:09.188843] Finish parameter search for model 'Neural Network' (t
```

```
ime: 127 seconds)

=== train : best params ===
{'activation': 'logistic', 'alpha': 0.05, 'hidden_layer_sizes': (50,), 'learning_r
ate': 'adaptive'}
=== train : best score : roc_auc ===
0.8074270924435595



Confusion Matrix:
[[812  69]
 [425 574]]

Classification Report:
              precision    recall  f1-score   support

         0.0       0.66      0.92      0.77       881
         1.0       0.89      0.57      0.70       999

    accuracy                           0.74      1880
   macro avg       0.77      0.75      0.73      1880
weighted avg       0.78      0.74      0.73      1880
```

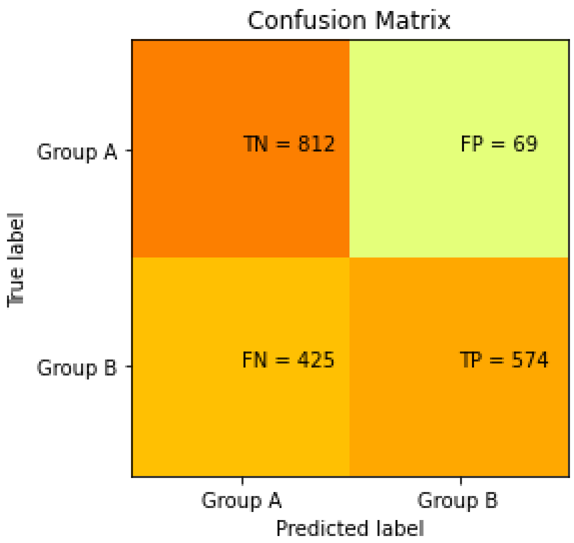


```
AUC Score : 0.7481272418843361

Accuracy Of the Model(LogisticRegression): 0.7372340425531915
```

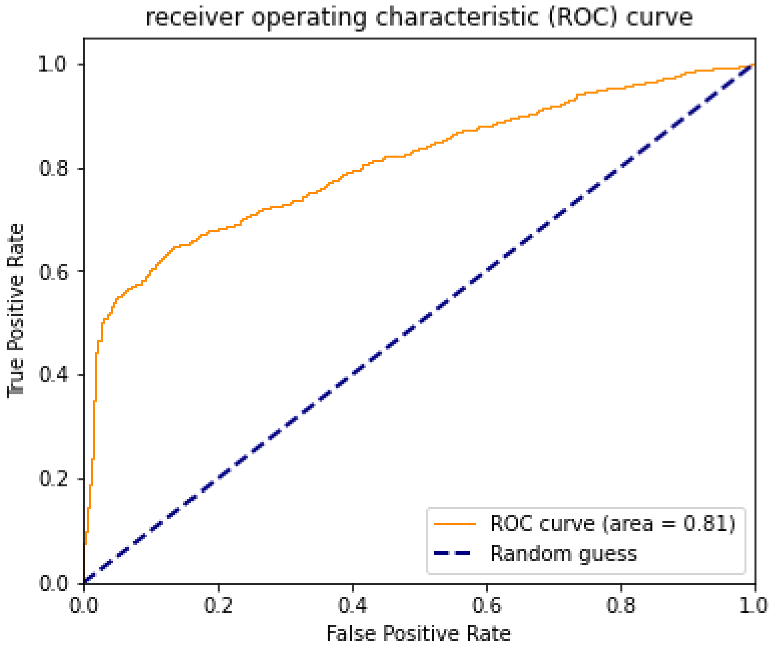


```
ROC_AUC Score of the model(LogisticRegression): 0.807483987960719


Confusion Matrix:
[[803  78]
 [402 597]]

Classification Report:
              precision    recall  f1-score   support

         0.0       0.67      0.91      0.77       881
         1.0       0.88      0.60      0.71       999

    accuracy                           0.74      1880
   macro avg       0.78      0.75      0.74      1880
weighted avg       0.78      0.74      0.74      1880
```

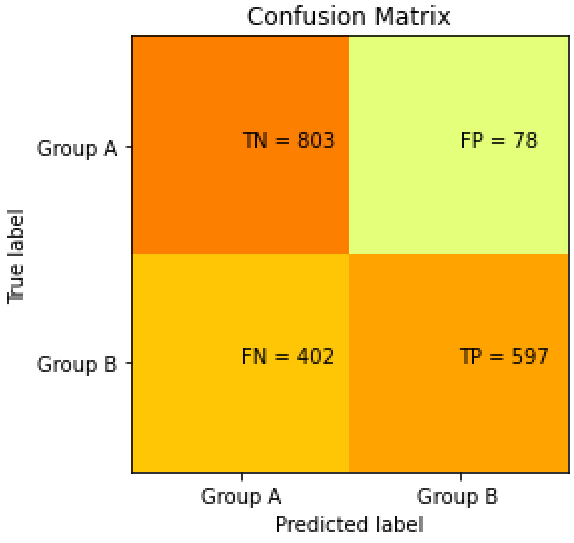

AUC Score : 0.754530921386767

Accuracy Of the Model(RandomForestClassifier): 0.7446808510638298

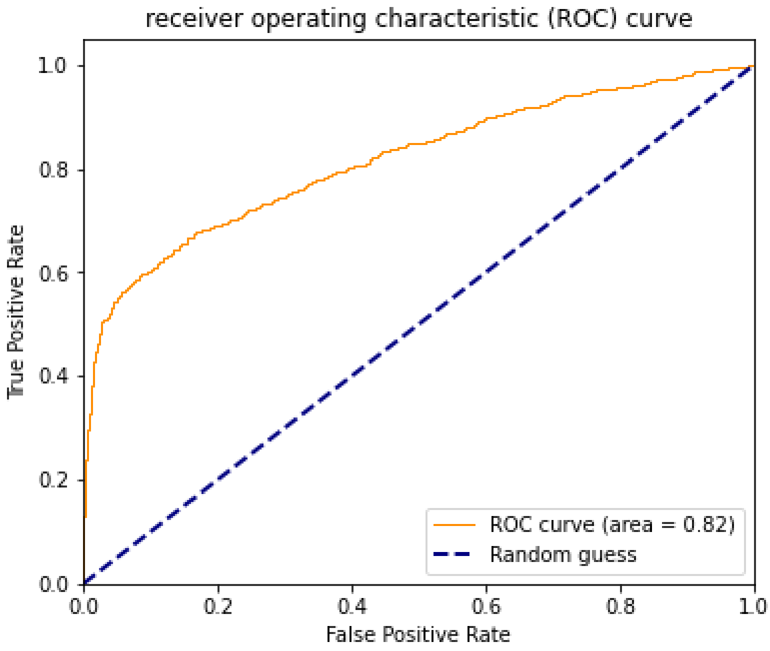

```
ROC_AUC Score of the model(RandomForestClassifier): 0.8176451138993704


Confusion Matrix:
[[818  63]
 [423 576]]

Classification Report:
              precision    recall  f1-score   support

         0.0       0.66      0.93      0.77       881
         1.0       0.90      0.58      0.70       999

    accuracy                           0.74      1880
   macro avg       0.78      0.75      0.74      1880
weighted avg       0.79      0.74      0.74      1880
```

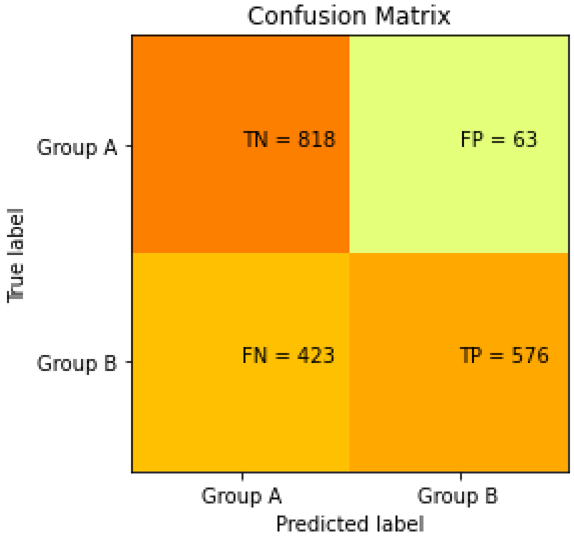


```
AUC Score : 0.7525334642247241

Accuracy Of the Model(XGBClassifier): 0.7414893617021276
```

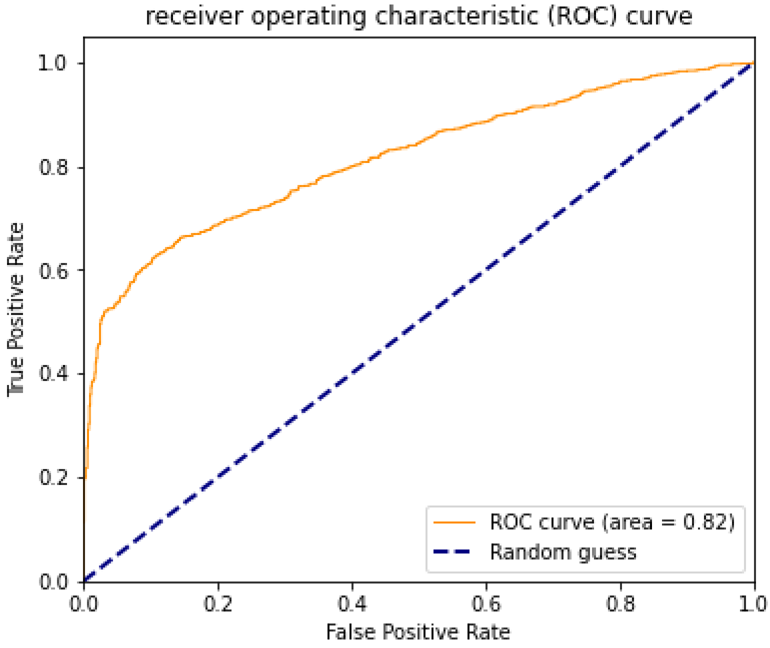

```
ROC_AUC Score of the model(XGBClassifier): 0.8151676080166432


Confusion Matrix:
[[802  79]
 [464 535]]

Classification Report:
              precision    recall  f1-score   support

         0.0       0.63      0.91      0.75       881
         1.0       0.87      0.54      0.66       999

    accuracy                           0.71      1880
   macro avg       0.75      0.72      0.71      1880
weighted avg       0.76      0.71      0.70      1880
```

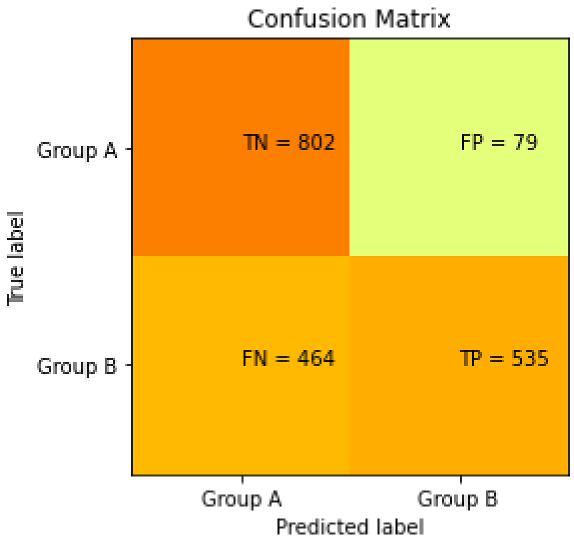


```
AUC Score : 0.7229323534658382

Accuracy Of the Model(KNeighborsClassifier): 0.7111702127659575
```

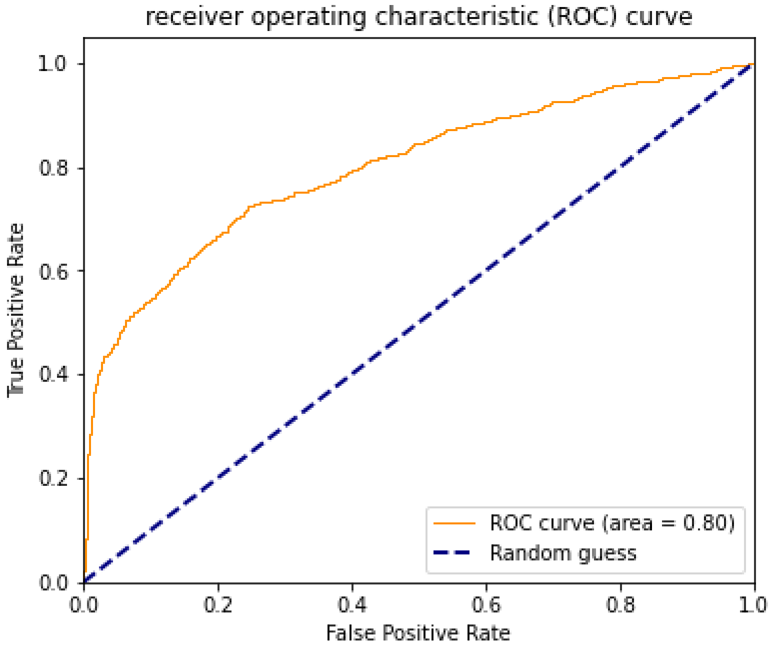

ROC_AUC Score of the model(KNeighborsClassifier): 0.8010871257182267

```
Confusion Matrix:
[[802  79]
 [419 580]]

Classification Report:
              precision    recall  f1-score   support

         0.0       0.66      0.91      0.76       881
         1.0       0.88      0.58      0.70       999

    accuracy                           0.74      1880
   macro avg       0.77      0.75      0.73      1880
weighted avg       0.78      0.74      0.73      1880
```

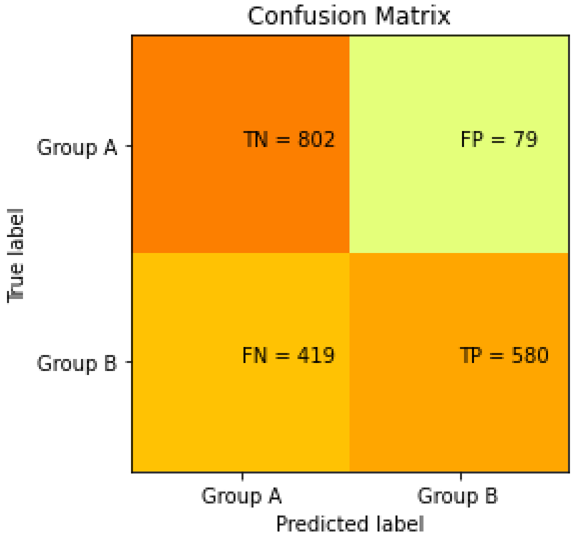

AUC Score : 0.7454548759883607

Accuracy Of the Model(DecisionTreeClassifier): 0.7351063829787234

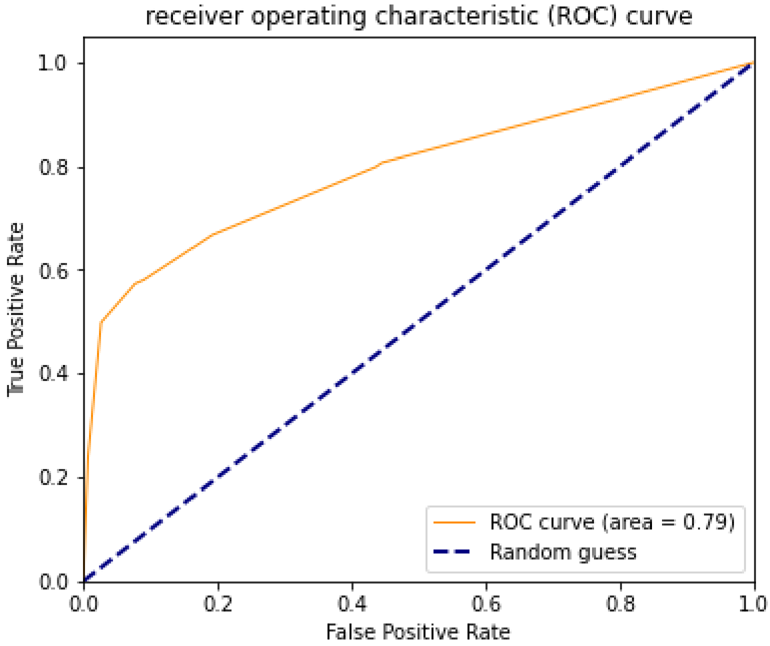

```
ROC_AUC Score of the model(DecisionTreeClassifier): 0.7938699198631094


Confusion Matrix:
[[802  79]
 [415 584]]

Classification Report:
              precision    recall  f1-score   support

         0.0       0.66      0.91      0.76       881
         1.0       0.88      0.58      0.70       999

    accuracy                           0.74      1880
   macro avg       0.77      0.75      0.73      1880
weighted avg       0.78      0.74      0.73      1880
```

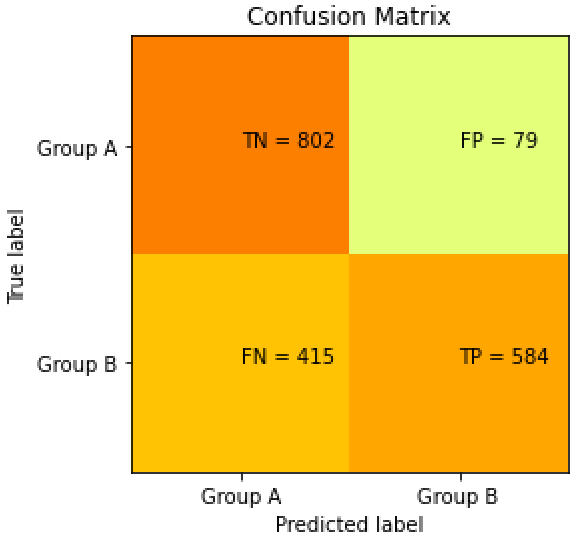


```
AUC Score : 0.7474568779903628

Accuracy Of the Model(ExtraTreesClassifier): 0.7372340425531915
```

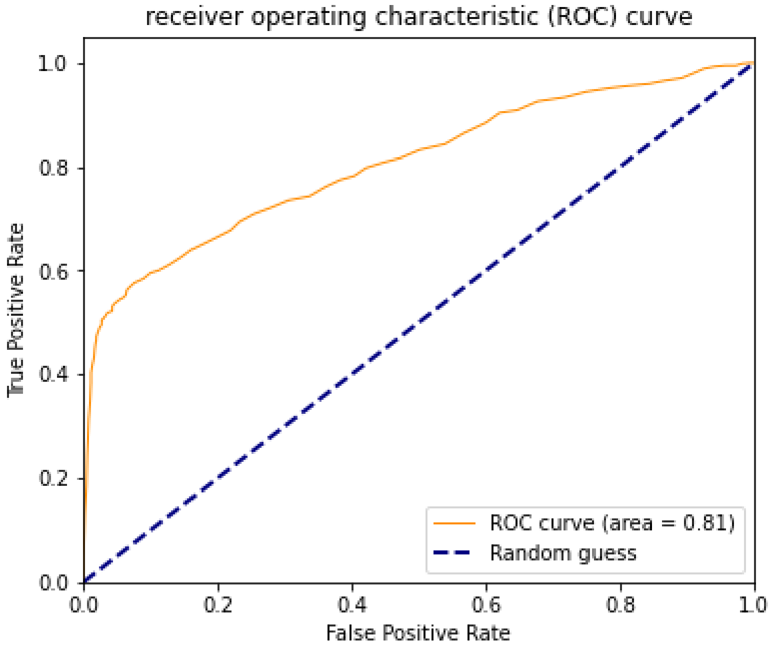

```
ROC_AUC Score of the model(ExtraTreesClassifier): 0.8063415288159896


Confusion Matrix:
[[803  78]
 [398 601]]

Classification Report:
              precision    recall  f1-score   support

         0.0       0.67      0.91      0.77       881
         1.0       0.89      0.60      0.72       999

    accuracy                           0.75      1880
   macro avg       0.78      0.76      0.74      1880
weighted avg       0.78      0.75      0.74      1880
```

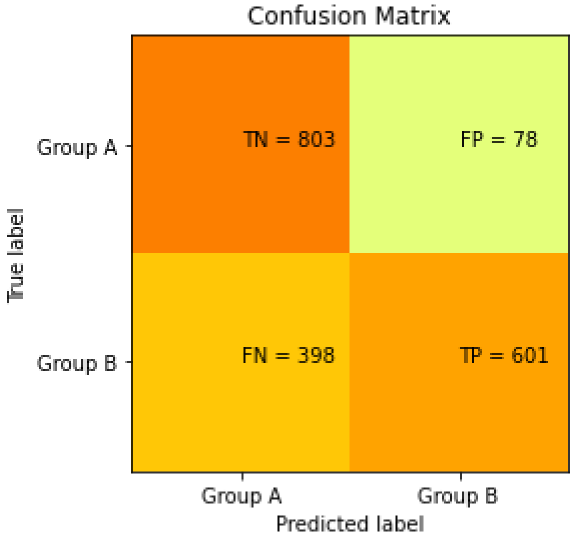


```
AUC Score : 0.756532923388769

Accuracy Of the Model(GradientBoostingClassifier): 0.7468085106382979
```

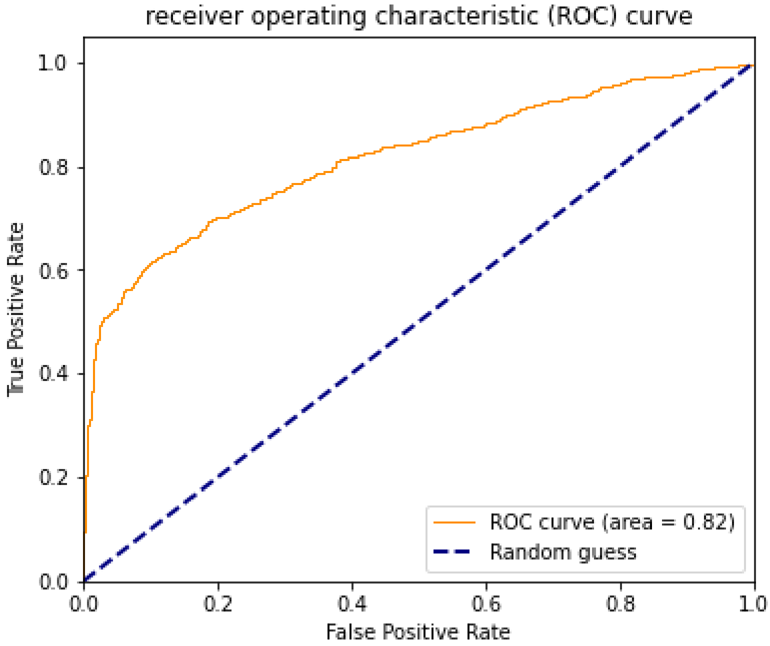

```
ROC_AUC Score of the model(GradientBoostingClassifier): 0.8180882357953867


Confusion Matrix:
[[833  48]
 [453 546]]

Classification Report:
              precision    recall  f1-score   support

         0.0       0.65      0.95      0.77       881
         1.0       0.92      0.55      0.69       999

    accuracy                           0.73      1880
   macro avg       0.78      0.75      0.73      1880
weighted avg       0.79      0.73      0.72      1880
```

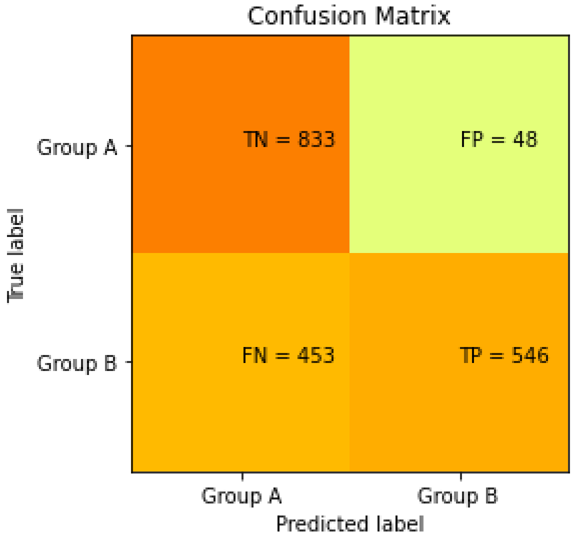


```
AUC Score : 0.7460315025581767

Accuracy Of the Model(AdaBoostClassifier): 0.7335106382978723
```

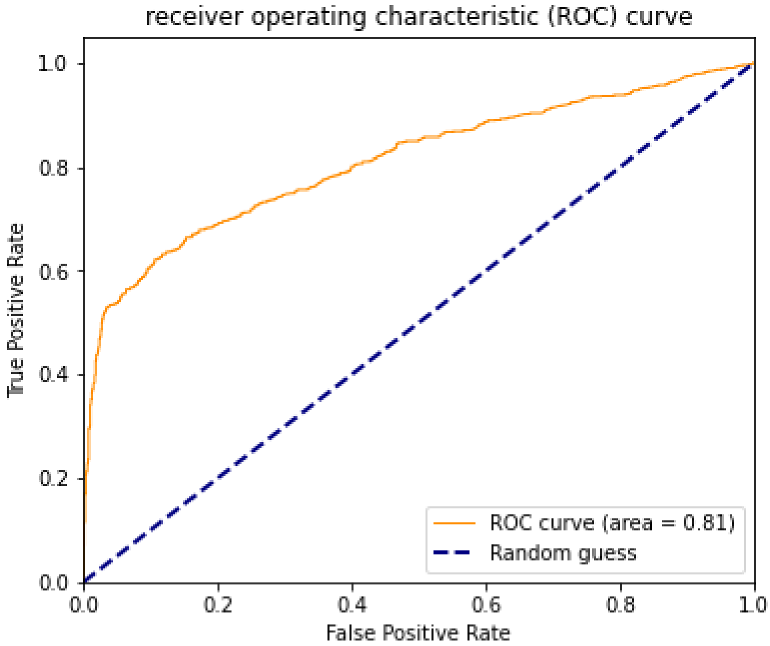

```
ROC_AUC Score of the model(AdaBoostClassifier): 0.8101699883765718


Confusion Matrix:
[[726 155]
 [354 645]]

Classification Report:
              precision    recall  f1-score   support

         0.0       0.67      0.82      0.74       881
         1.0       0.81      0.65      0.72       999

    accuracy                           0.73      1880
   macro avg       0.74      0.73      0.73      1880
weighted avg       0.74      0.73      0.73      1880
```

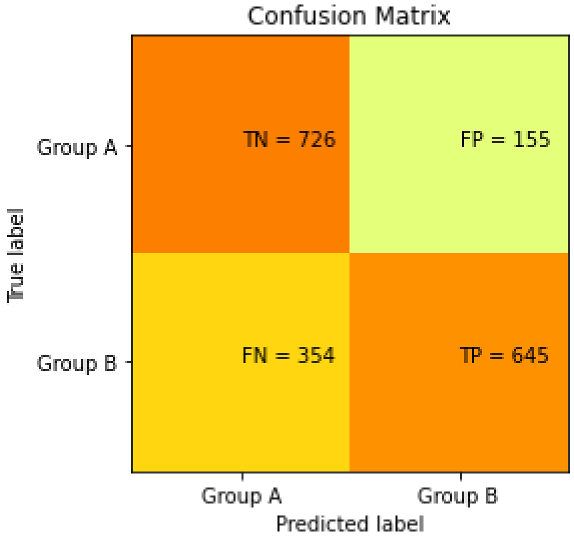


```
AUC Score : 0.7348546048886571

Accuracy Of the Model(SVC): 0.7292553191489362
```

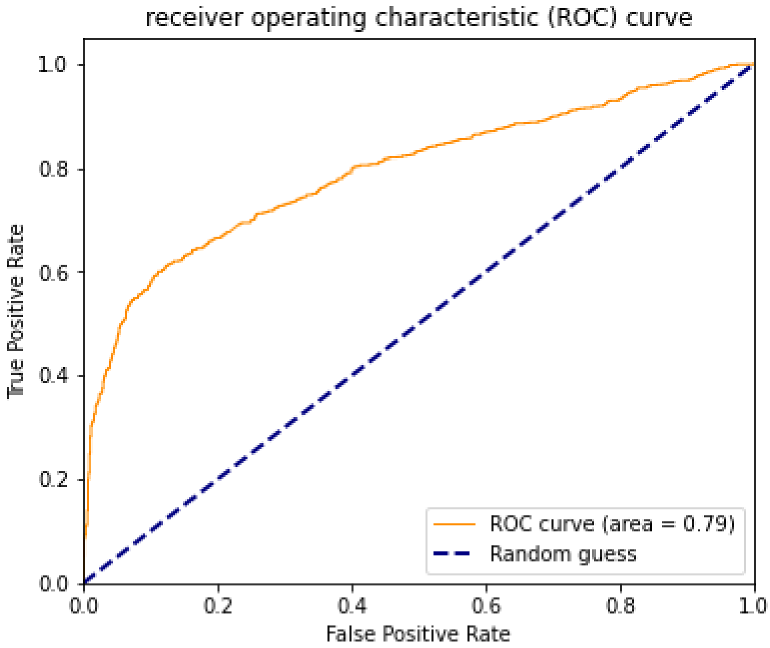

```
ROC_AUC Score of the model(SVC): 0.7928297196174607


Confusion Matrix:
[[801  80]
 [405 594]]

Classification Report:
              precision    recall  f1-score   support

         0.0       0.66      0.91      0.77       881
         1.0       0.88      0.59      0.71       999

    accuracy                           0.74      1880
   macro avg       0.77      0.75      0.74      1880
weighted avg       0.78      0.74      0.74      1880
```

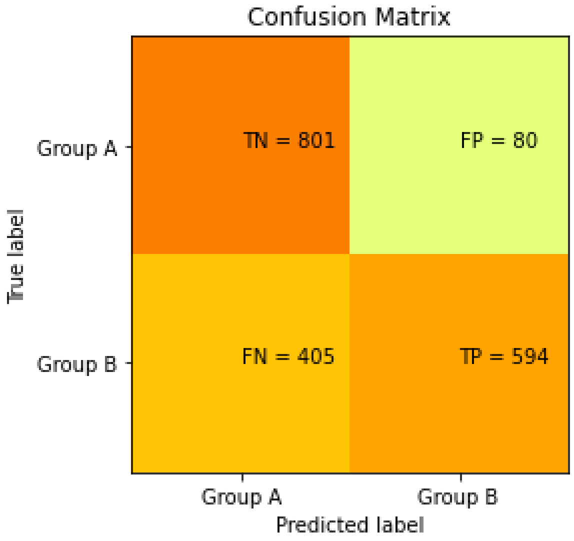


```
AUC Score : 0.7518943461054699

Accuracy Of the Model(MLPClassifier): 0.7420212765957447
```

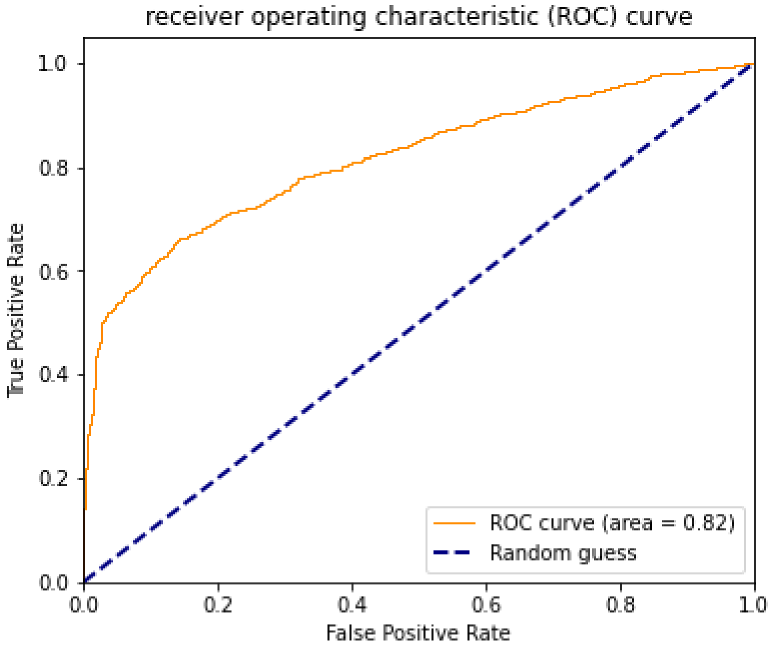

```
ROC_AUC Score of the model(MLPClassifier): 0.8179893855262754

[('GradientBoostingClassifier', 0.7468085106382979), ('RandomForestClassifier', 0.7446808510638298), ('MLPClassifier', 0.7420212765957447), ('XGBClassifier', 0.7414893617021276), ('LogisticRegression', 0.7372340425531915), ('ExtraTreesClassifier', 0.7372340425531915), ('DecisionTreeClassifier', 0.7351063829787234), ('AdaBoostClassifier', 0.7335106382978723), ('SVC', 0.7292553191489362), ('KNeighborsClassifier', 0.7111702127659575)]


sorted_total_auc:
 [('GradientBoostingClassifier', 0.8180882357953867), ('MLPClassifier', 0.8179893855262754), ('RandomForestClassifier', 0.8176451138993704), ('XGBClassifier', 0.8151676080166432), ('AdaBoostClassifier', 0.8101699883765718), ('LogisticRegression', 0.807483987960719), ('ExtraTreesClassifier', 0.8063415288159896), ('KNeighborsClassifier', 0.8010871257182267), ('DecisionTreeClassifier', 0.7938699198631094), ('SVC', 0.7928297196174607)]
```

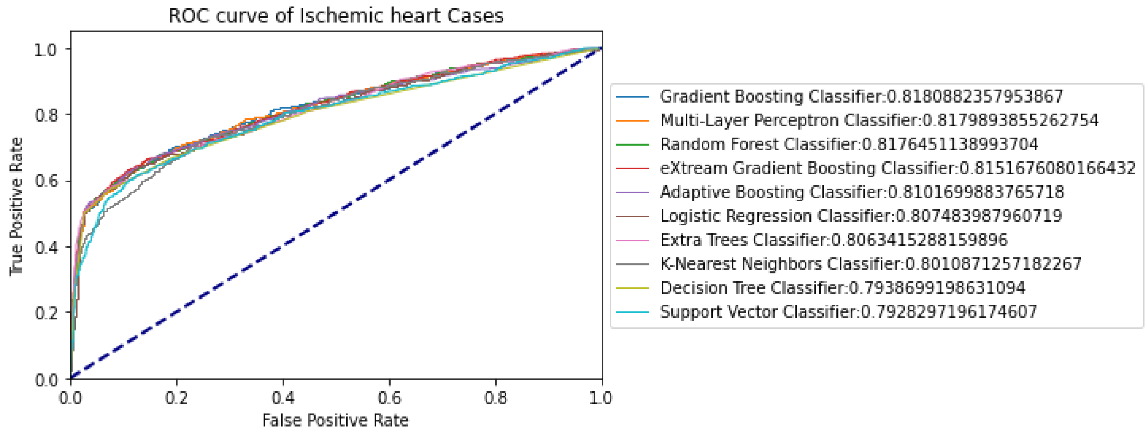

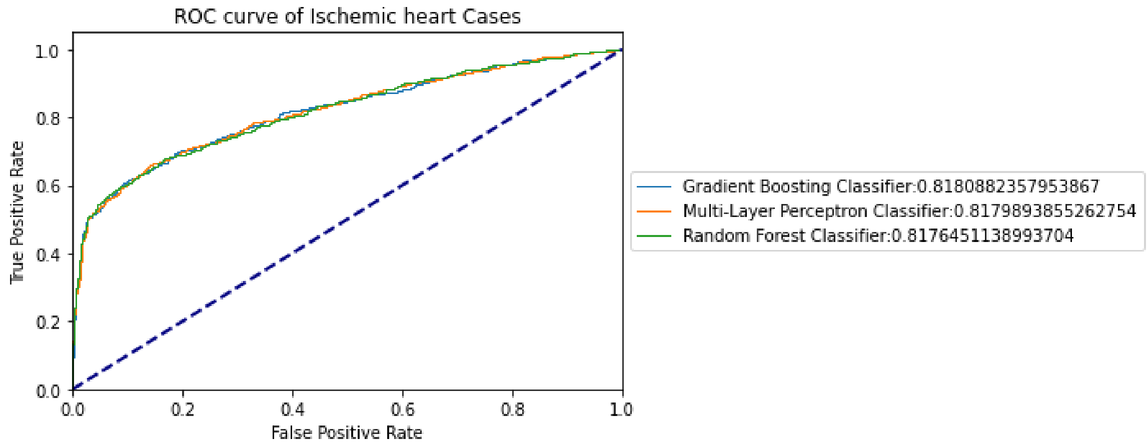

```
random state :   12351
9398
result_train_filename: data/train_test_data_ischemic heart_2013_20210519_111916.cs
v
total count : 9398, df_train count : 7518, df_test count : 1880
(7518, 38)
(1880, 38)
('Logistic Regression', LogisticRegression())
[2021-05-19 11:19:16.990572] Start parameter search for model 'Logistic Regressio
n'
[2021-05-19 11:19:17.304190] Finish parameter search for model 'Logistic Regressio
n' (time: 0 seconds)

=== train : best params ===
{'C': 0.1, 'penalty': 'l2', 'solver': 'lbfgs'}
=== train : best score : roc_auc ===
0.8059064532177408


('Random Forest', RandomForestClassifier())
[2021-05-19 11:19:17.305182] Start parameter search for model 'Random Forest'
[2021-05-19 11:19:21.230524] Finish parameter search for model 'Random Forest' (ti
me: 3 seconds)

=== train : best params ===
{'criterion': 'gini', 'max_depth': 8, 'max_features': 'auto', 'n_estimators': 90}
=== train : best score : roc_auc ===
0.8123035893703558


('Extreme Gradient Boosting)', XGBClassifier(base_score=None, booster=None, colsam
ple_bylevel=None,
              colsample_bynode=None, colsample_bytree=None, gamma=None,
              gpu_id=None, importance_type='gain', interaction_constraints=None,
              learning_rate=None, max_delta_step=None, max_depth=None,
              min_child_weight=None, missing=nan, monotone_constraints=None,
              n_estimators=100, n_jobs=None, num_parallel_tree=None,
              random_state=None, reg_alpha=None, reg_lambda=None,
              scale_pos_weight=None, subsample=None, tree_method=None,
              validate_parameters=None, verbosity=None))
[2021-05-19 11:19:21.230524] Start parameter search for model 'Extreme Gradient Bo
osting)'
[2021-05-19 11:19:44.521803] Finish parameter search for model 'Extreme Gradient B
oosting)' (time: 23 seconds)

=== train : best params ===
{'learning_rate': 0.075, 'max_depth': 2, 'n_estimators': 100, 'subsample': 0.7}
=== train : best score : roc_auc ===
0.8125871947286945


('k-Nearest Neighbours)', KNeighborsClassifier())
[2021-05-19 11:19:44.523797] Start parameter search for model 'k-Nearest Neighbour
s)'
[2021-05-19 11:19:52.446568] Finish parameter search for model 'k-Nearest Neighbou
rs)' (time: 7 seconds)

=== train : best params ===
{'n_neighbors': 60, 'weights': 'distance'}
=== train : best score : roc_auc ===
0.7892111961436032
```

```
('Decision Tree', DecisionTreeClassifier())
[2021-05-19 11:19:52.446568] Start parameter search for model 'Decision Tree'
[2021-05-19 11:19:53.028578] Finish parameter search for model 'Decision Tree' (ti
me: 0 seconds)

=== train : best params ===
{'criterion': 'gini', 'max_depth': 5}
=== train : best score : roc_auc ===
0.7965325445374986


('Extra Tree', ExtraTreesClassifier())
[2021-05-19 11:19:53.029576] Start parameter search for model 'Extra Tree'
[2021-05-19 11:19:55.758351] Finish parameter search for model 'Extra Tree' (time:
2 seconds)

=== train : best params ===
{'max_features': 'log2', 'n_estimators': 100}
=== train : best score : roc_auc ===
0.8054399606761388


('Gradient Boosting', GradientBoostingClassifier())
[2021-05-19 11:19:55.758351] Start parameter search for model 'Gradient Boosting'
[2021-05-19 11:20:11.399936] Finish parameter search for model 'Gradient Boosting'
(time: 15 seconds)

=== train : best params ===
{'learning_rate': 0.01, 'max_depth': 8, 'max_features': 6, 'n_estimators': 100}
=== train : best score : roc_auc ===
0.8121175867170634


('Adaptive Boosting', AdaBoostClassifier())
[2021-05-19 11:20:11.400964] Start parameter search for model 'Adaptive Boosting'
[2021-05-19 11:20:15.108670] Finish parameter search for model 'Adaptive Boosting'
(time: 3 seconds)

=== train : best params ===
{'learning_rate': 0.2, 'n_estimators': 100}
=== train : best score : roc_auc ===
0.8061379802689976


('Suppport Vector Classfier', SVC(probability=True))
[2021-05-19 11:20:15.109660] Start parameter search for model 'Suppport Vector Cla
ssfier'
[2021-05-19 11:24:17.961723] Finish parameter search for model 'Suppport Vector Cl
assfier' (time: 242 seconds)

=== train : best params ===
{'C': 1, 'gamma': 0.1, 'kernel': 'rbf'}
=== train : best score : roc_auc ===
0.786059072340515


('Neural Network', MLPClassifier())
[2021-05-19 11:24:17.961723] Start parameter search for model 'Neural Network'
[2021-05-19 11:26:33.675103] Finish parameter search for model 'Neural Network' (t
```

```
ime: 135 seconds)

=== train : best params ===
{'activation': 'logistic', 'alpha': 0.05, 'hidden_layer_sizes': (50, 50), 'learnin
g_rate': 'adaptive'}
=== train : best score : roc_auc ===
0.8115142413720249



Confusion Matrix:
[[810  71]
 [426 573]]

Classification Report:
              precision    recall  f1-score   support

         0.0       0.66      0.92      0.77       881
         1.0       0.89      0.57      0.70       999

    accuracy                           0.74      1880
   macro avg       0.77      0.75      0.73      1880
weighted avg       0.78      0.74      0.73      1880
```

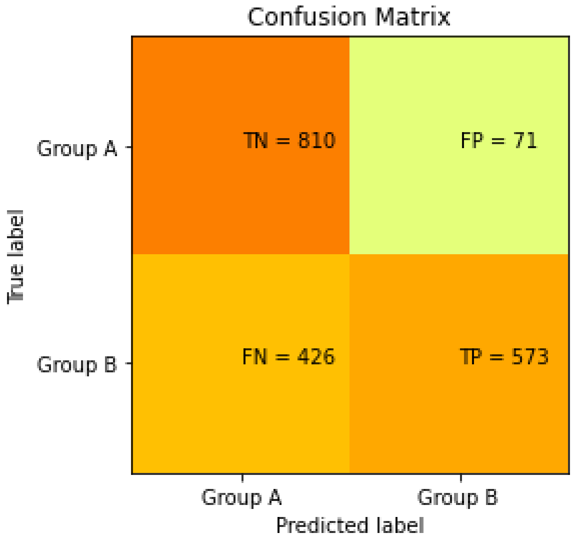


```
AUC Score : 0.746491667604O4

Accuracy Of the Model(LogisticRegression): 0.7356382978723405
```

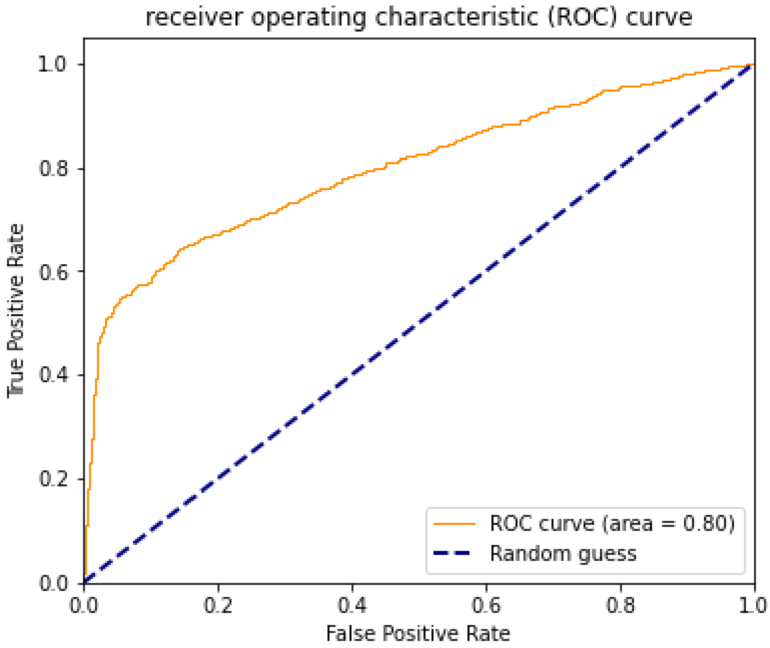


```
ROC_AUC Score of the model(LogisticRegression): 0.8007746679710357


Confusion Matrix:
[[784  97]
 [395 604]]

Classification Report:
              precision    recall  f1-score   support

         0.0       0.66      0.89      0.76       881
         1.0       0.86      0.60      0.71       999

    accuracy                           0.74      1880
   macro avg       0.76      0.75      0.74      1880
weighted avg       0.77      0.74      0.73      1880
```

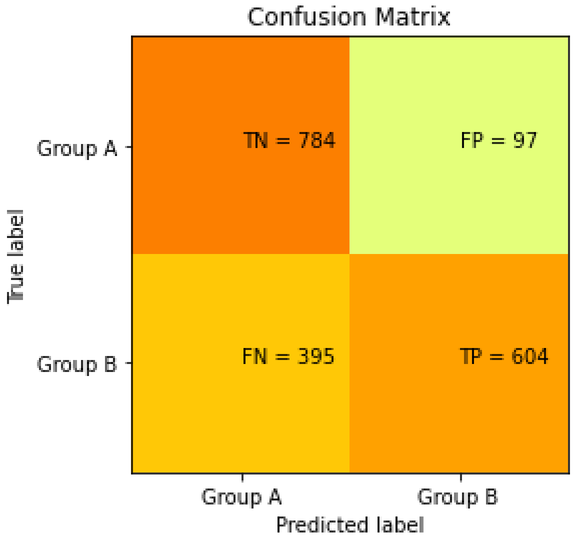

AUC Score : 0.7472512239822114

Accuracy Of the Model(RandomForestClassifier): 0.7382978723404255

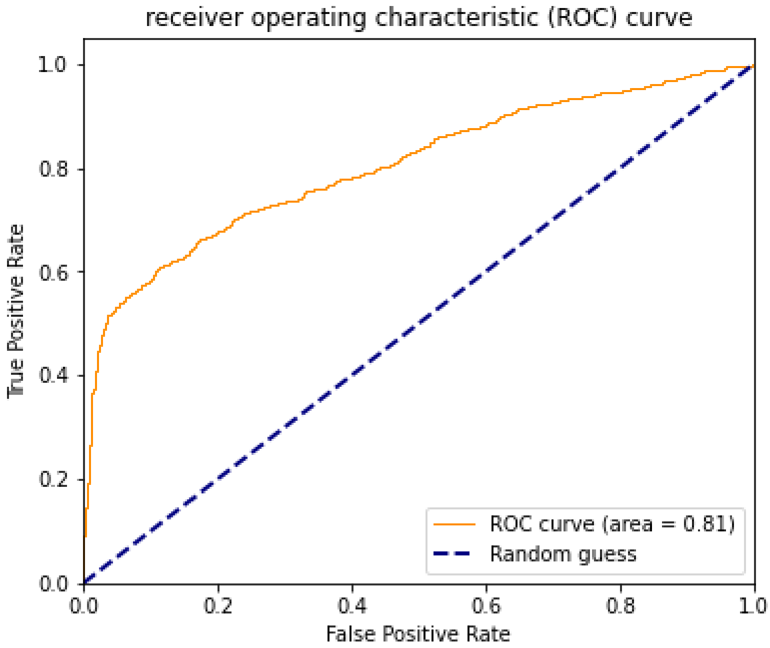

```
ROC_AUC Score of the model(RandomForestClassifier): 0.8053274614001061


Confusion Matrix:
[[798  83]
 [418 581]]

Classification Report:
              precision    recall  f1-score   support

         0.0       0.66      0.91      0.76       881
         1.0       0.88      0.58      0.70       999

    accuracy                           0.73      1880
   macro avg       0.77      0.74      0.73      1880
weighted avg       0.77      0.73      0.73      1880
```

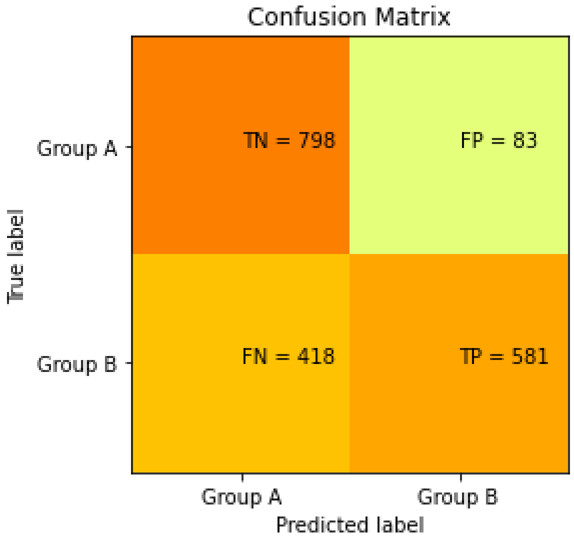


```
AUC Score : 0.7436852289292698

Accuracy Of the Model(XGBClassifier): 0.7335106382978723
```

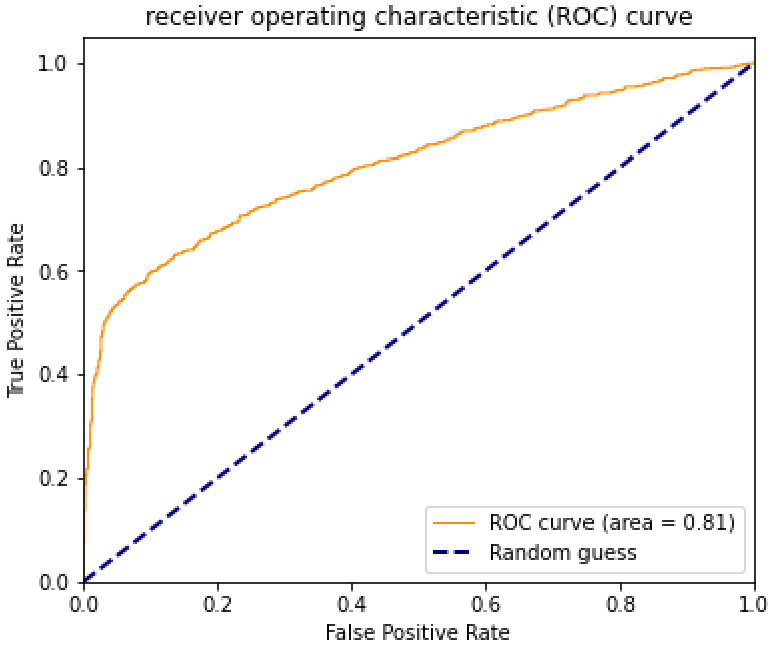

```
ROC_AUC Score of the model(XGBClassifier): 0.8052484948058161


Confusion Matrix:
[[791  90]
 [463 536]]

Classification Report:
              precision    recall  f1-score   support

         0.0       0.63      0.90      0.74       881
         1.0       0.86      0.54      0.66       999

    accuracy                           0.71      1880
   macro avg       0.74      0.72      0.70      1880
weighted avg       0.75      0.71      0.70      1880
```

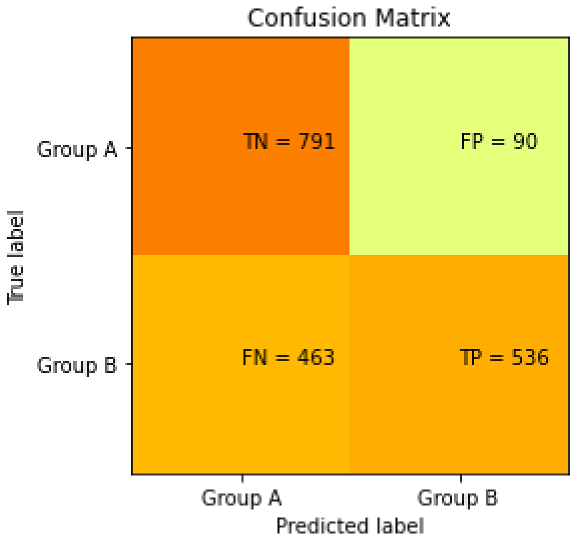


```
AUC Score : 0.7171899481774623

Accuracy Of the Model(KNeighborsClassifier): 0.7058510638297872
```

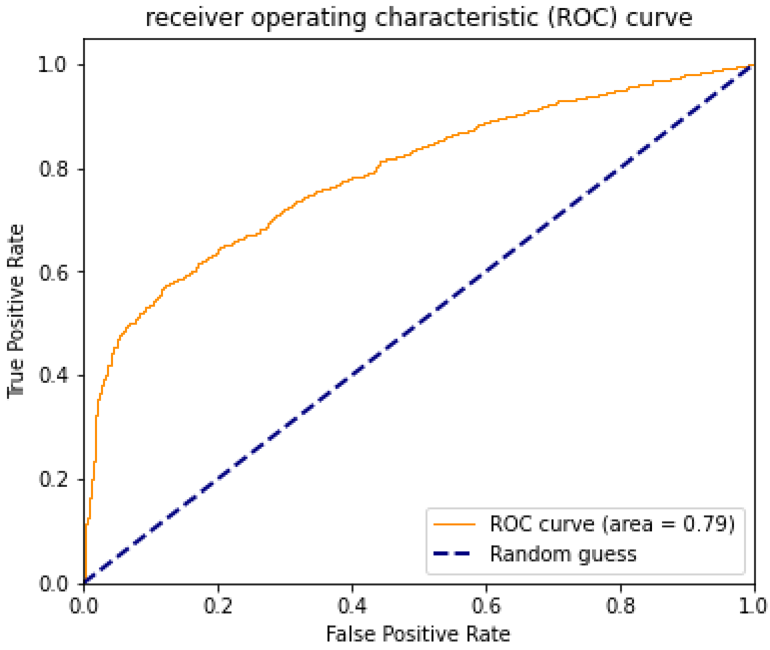

```
ROC_AUC Score of the model(KNeighborsClassifier): 0.7896329928111995


Confusion Matrix:
[[790  91]
 [409 590]]

Classification Report:
              precision    recall  f1-score   support

         0.0       0.66      0.90      0.76       881
         1.0       0.87      0.59      0.70       999

    accuracy                           0.73      1880
   macro avg       0.76      0.74      0.73      1880
weighted avg       0.77      0.73      0.73      1880
```

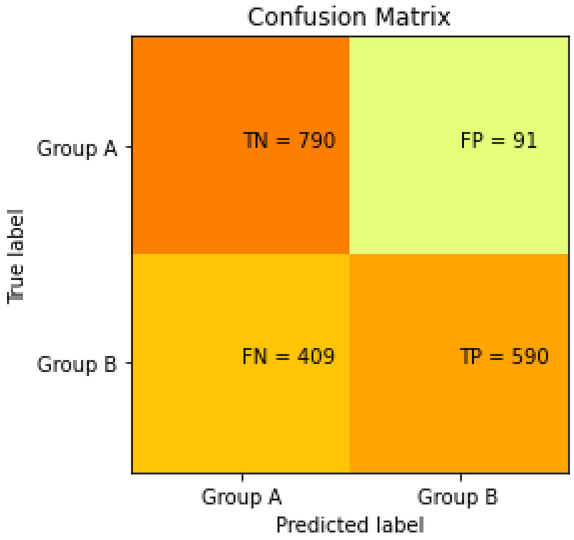


```
AUC Score : 0.7436494383145915

Accuracy Of the Model(DecisionTreeClassifier): 0.7340425531914894
```

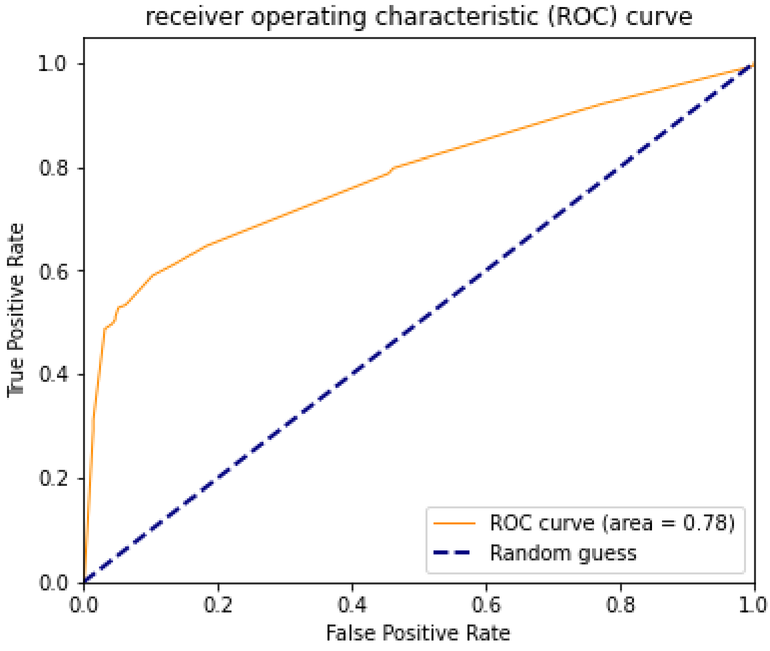

```
ROC_AUC Score of the model(DecisionTreeClassifier): 0.7820851498490545


Confusion Matrix:
[[783  98]
 [416 583]]

Classification Report:
              precision    recall  f1-score   support

         0.0       0.65      0.89      0.75       881
         1.0       0.86      0.58      0.69       999

    accuracy                           0.73      1880
   macro avg       0.75      0.74      0.72      1880
weighted avg       0.76      0.73      0.72      1880
```

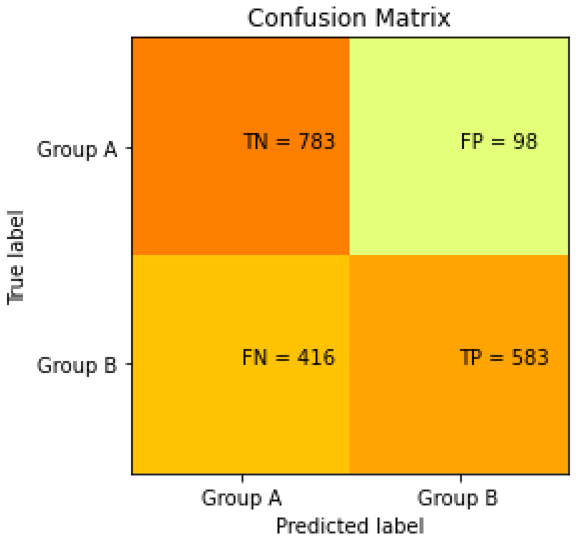


```
AUC Score : 0.7361731765818031

Accuracy Of the Model(ExtraTreesClassifier): 0.7265957446808511
```

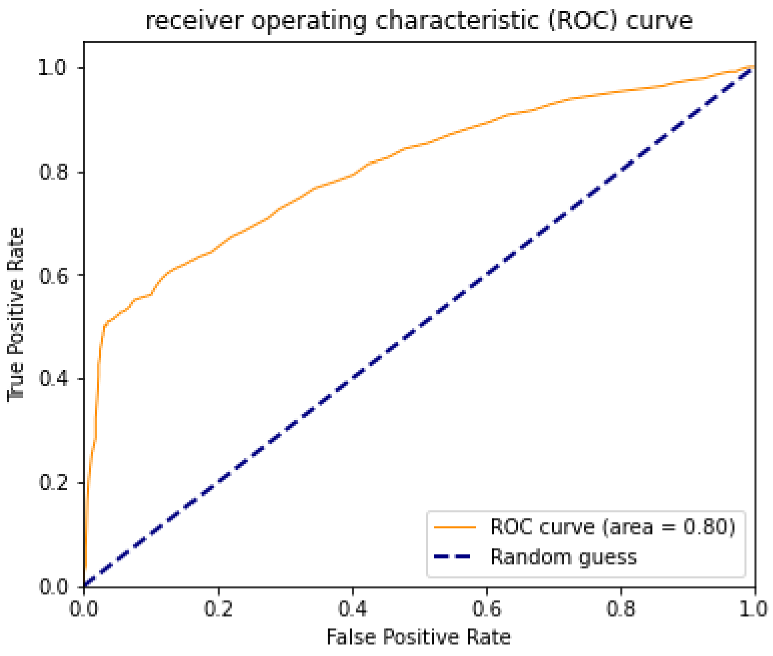

```
ROC_AUC Score of the model(ExtraTreesClassifier): 0.8031249183349071


Confusion Matrix:
[[784  97]
 [400 599]]

Classification Report:
              precision    recall  f1-score   support

         0.0       0.66      0.89      0.76       881
         1.0       0.86      0.60      0.71       999

    accuracy                           0.74      1880
   macro avg       0.76      0.74      0.73      1880
weighted avg       0.77      0.74      0.73      1880
```

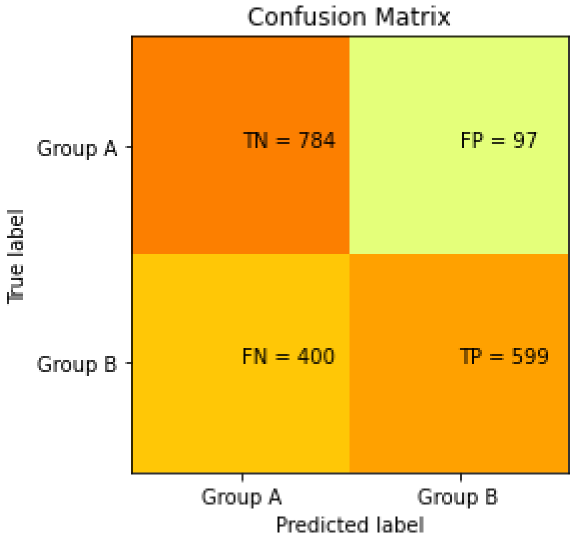


```
AUC Score : 0.7447487214797089

Accuracy Of the Model(GradientBoostingClassifier): 0.7356382978723405
```

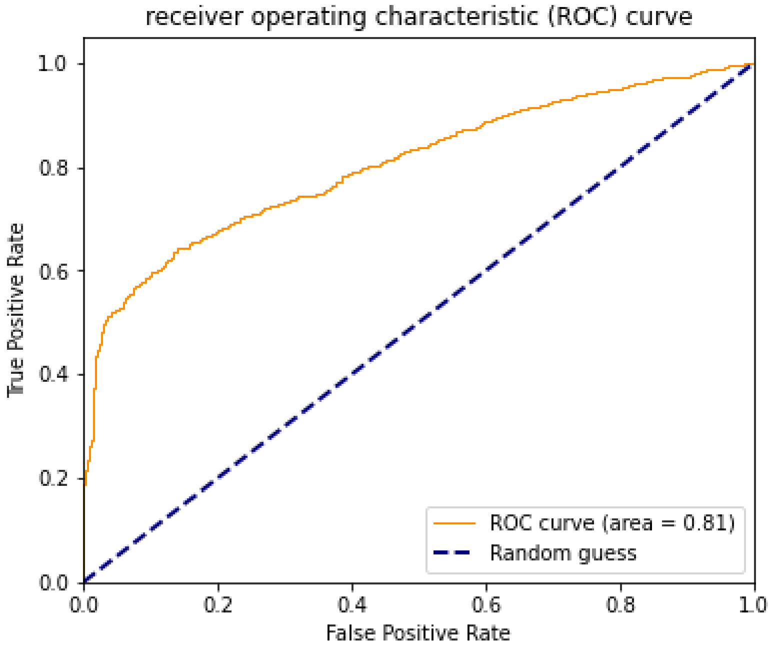

```
ROC_AUC Score of the model(GradientBoostingClassifier): 0.8060023701340387


Confusion Matrix:
[[810  71]
 [430 569]]

Classification Report:
              precision    recall  f1-score   support

         0.0       0.65      0.92      0.76       881
         1.0       0.89      0.57      0.69       999

    accuracy                           0.73      1880
   macro avg       0.77      0.74      0.73      1880
weighted avg       0.78      0.73      0.73      1880
```

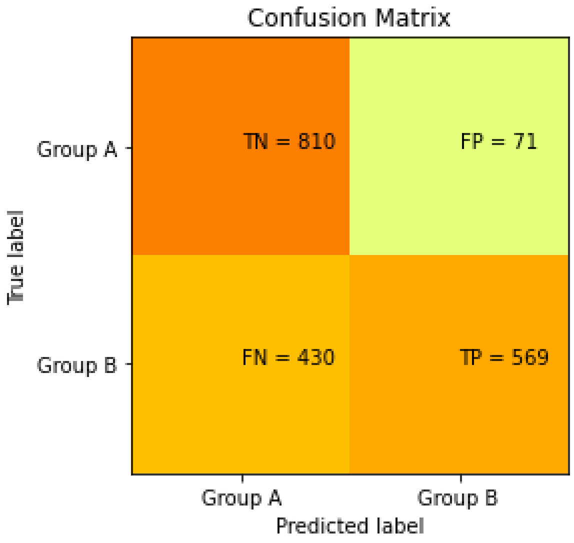


```
AUC Score : 0.7444489665602038

Accuracy Of the Model(AdaBoostClassifier): 0.7335106382978723
```

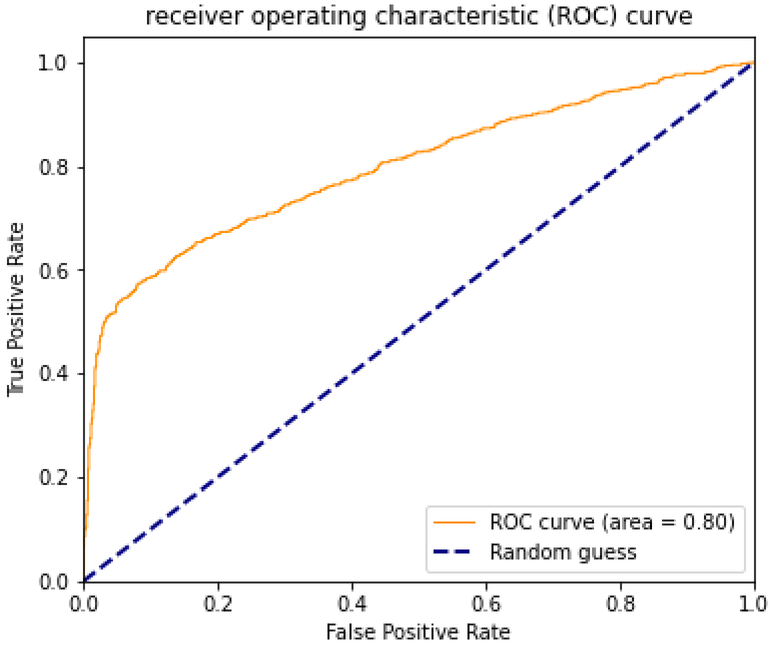

```
ROC_AUC Score of the model(AdaBoostClassifier): 0.7978148409476447


Confusion Matrix:
[[689 192]
 [343 656]]

Classification Report:
              precision    recall  f1-score   support

         0.0       0.67      0.78      0.72       881
         1.0       0.77      0.66      0.71       999

    accuracy                           0.72      1880
   macro avg       0.72      0.72      0.72      1880
weighted avg       0.72      0.72      0.72      1880
```

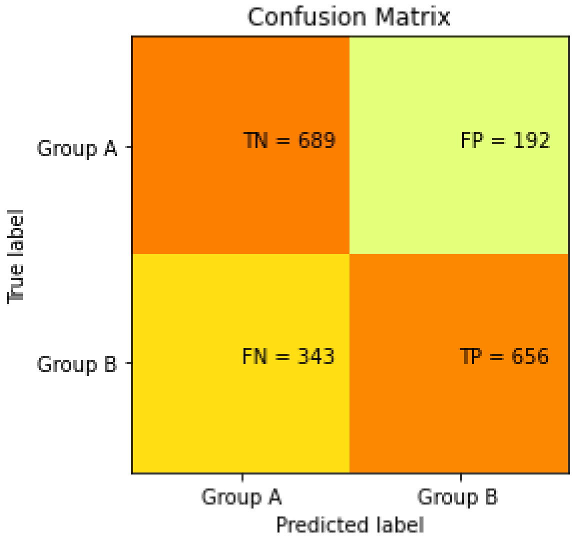


```
AUC Score : 0.7193612454679423

Accuracy Of the Model(SVC): 0.7154255319148937
```

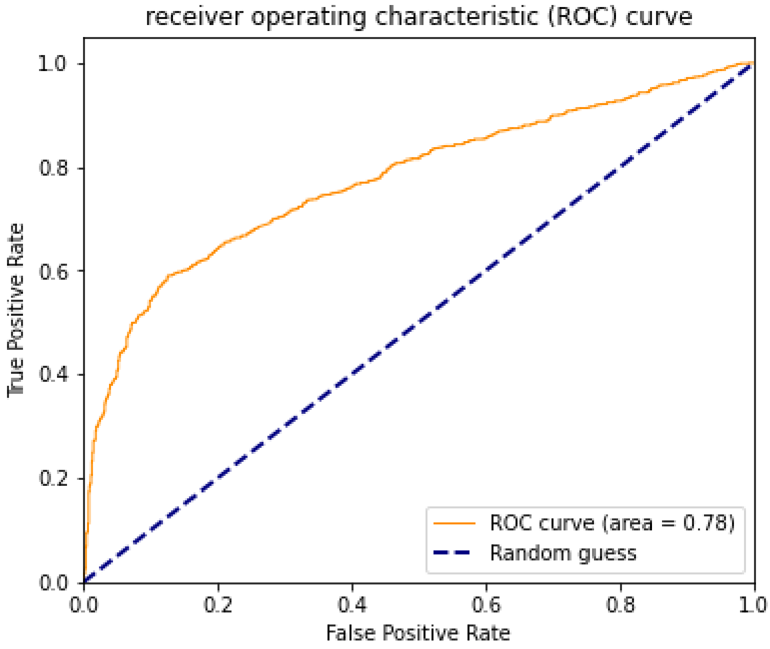

```
ROC_AUC Score of the model(SVC): 0.7750934816769095


Confusion Matrix:
[[776 105]
 [401 598]]

Classification Report:
              precision    recall  f1-score   support

         0.0       0.66      0.88      0.75       881
         1.0       0.85      0.60      0.70       999

    accuracy                           0.73      1880
   macro avg       0.75      0.74      0.73      1880
weighted avg       0.76      0.73      0.73      1880
```

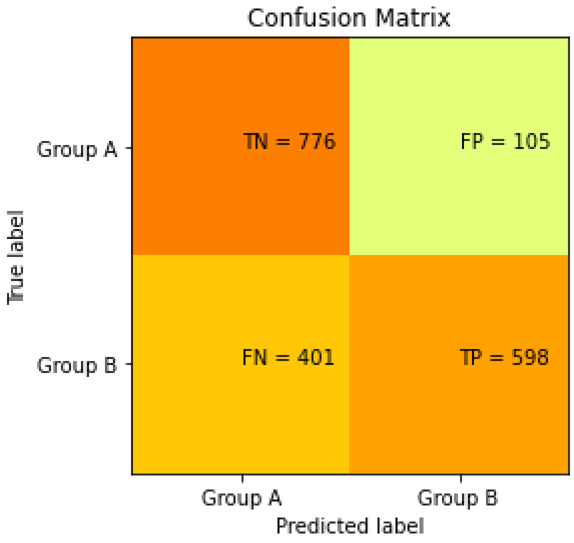


```
AUC Score : 0.7397079258600258

Accuracy Of the Model(MLPClassifier): 0.7308510638297873
```

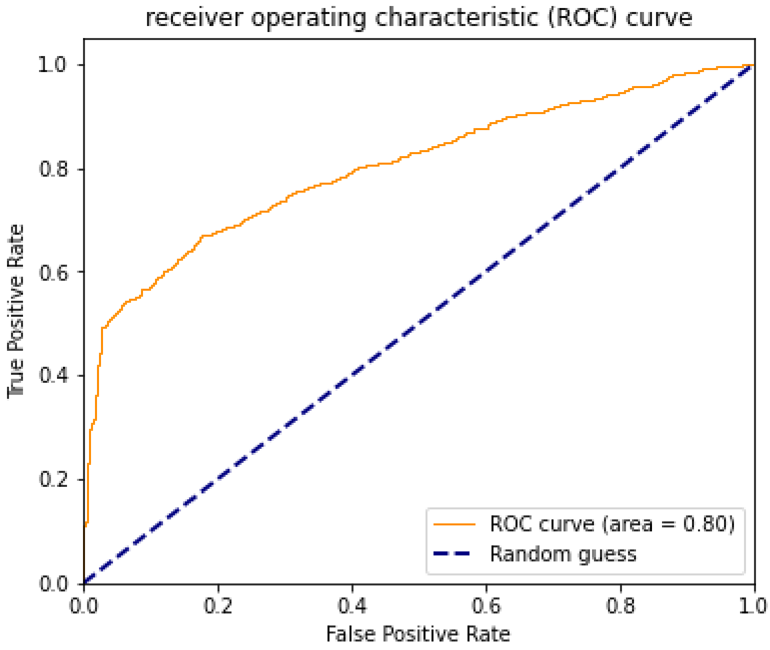

```
ROC_AUC Score of the model(MLPClassifier): 0.8034390803970828

[('RandomForestClassifier', 0.7382978723404255), ('LogisticRegression', 0.73563829
78723405), ('GradientBoostingClassifier', 0.7356382978723405), ('DecisionTreeClass
ifier', 0.7340425531914894), ('XGBClassifier', 0.7335106382978723), ('AdaBoostClas
sifier', 0.7335106382978723), ('MLPClassifier', 0.7308510638297873), ('ExtraTreesC
lassifier', 0.7265957446808511), ('SVC', 0.7154255319148937), ('KNeighborsClassifi
er', 0.7058510638297872)]


sorted_total_auc:
 [('GradientBoostingClassifier', 0.8060023701340387), ('RandomForestClassifier',
0.8053274614001061), ('XGBClassifier', 0.8052484948058161), ('MLPClassifier', 0.80
34390803970828), ('ExtraTreesClassifier', 0.8031249183349071), ('LogisticRegressio
n', 0.8007746679710357), ('AdaBoostClassifier', 0.7978148409476447), ('KNeighborsC
lassifier', 0.7896329928111995), ('DecisionTreeClassifier', 0.7820851498490545),
('SVC', 0.7750934816769095)]
```

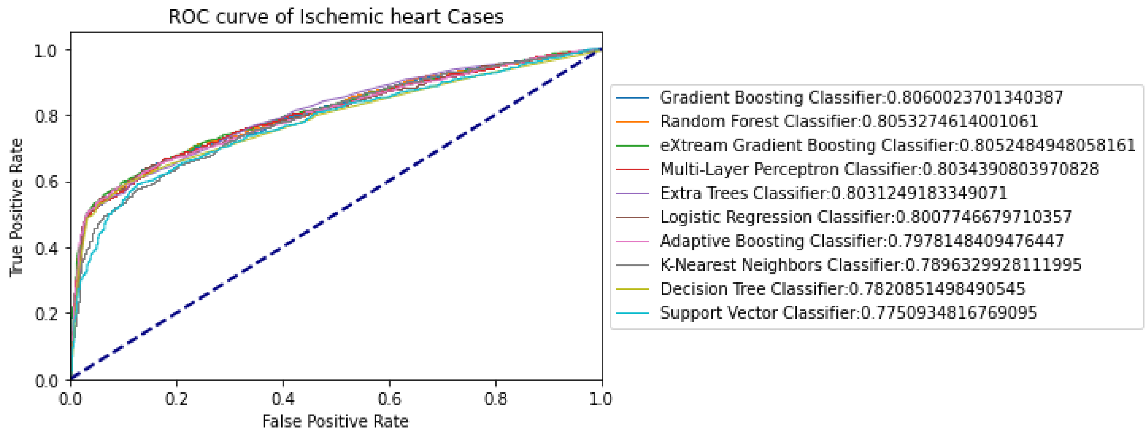

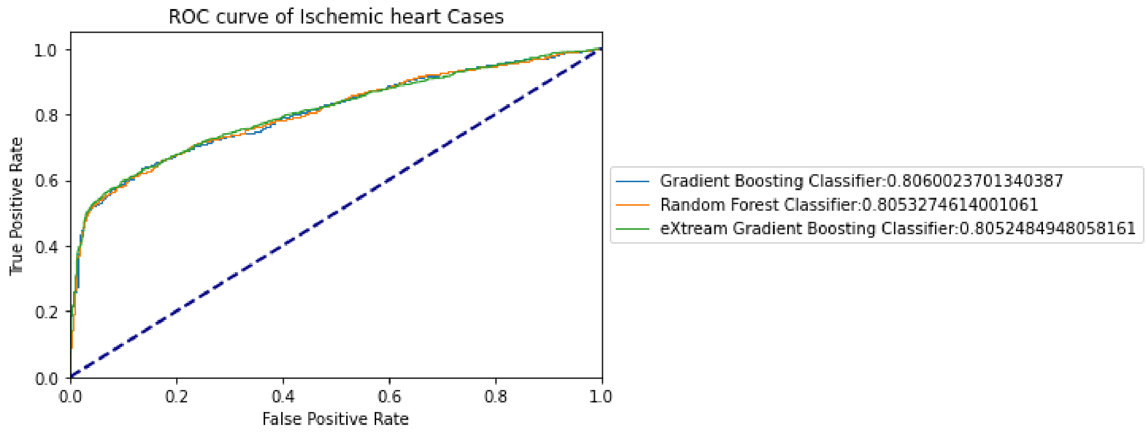

```
random state :   12355
9398
result_train_filename: data/train_test_data_ischemic heart_2013_20210519_112641.cs
v
total count : 9398, df_train count : 7518, df_test count : 1880
(7518, 38)
(1880, 38)
('Logistic Regression', LogisticRegression())
[2021-05-19 11:26:41.427605] Start parameter search for model 'Logistic Regressio
n'
[2021-05-19 11:26:41.751101] Finish parameter search for model 'Logistic Regressio
n' (time: 0 seconds)

=== train : best params ===
{'C': 0.1, 'penalty': 'l2', 'solver': 'lbfgs'}
=== train : best score : roc_auc ===
0.8079212284702747



('Random Forest', RandomForestClassifier())
[2021-05-19 11:26:41.752098] Start parameter search for model 'Random Forest'
[2021-05-19 11:26:46.109660] Finish parameter search for model 'Random Forest' (ti
me: 4 seconds)

=== train : best params ===
{'criterion': 'gini', 'max_depth': 10, 'max_features': 'auto', 'n_estimators': 10
0}
=== train : best score : roc_auc ===
0.8132625452592446



('Extreme Gradient Boosting)', XGBClassifier(base_score=None, booster=None, colsam
ple_bylevel=None,
              colsample_bynode=None, colsample_bytree=None, gamma=None,
              gpu_id=None, importance_type='gain', interaction_constraints=None,
              learning_rate=None, max_delta_step=None, max_depth=None,
              min_child_weight=None, missing=nan, monotone_constraints=None,
              n_estimators=100, n_jobs=None, num_parallel_tree=None,
              random_state=None, reg_alpha=None, reg_lambda=None,
              scale_pos_weight=None, subsample=None, tree_method=None,
              validate_parameters=None, verbosity=None))
[2021-05-19 11:26:46.110669] Start parameter search for model 'Extreme Gradient Bo
osting)'
[2021-05-19 11:27:12.986262] Finish parameter search for model 'Extreme Gradient B
oosting)' (time: 26 seconds)

=== train : best params ===
{'learning_rate': 0.075, 'max_depth': 5, 'n_estimators': 50, 'subsample': 0.7}
=== train : best score : roc_auc ===
0.8144342257715105



('k-Nearest Neighbours)', KNeighborsClassifier())
[2021-05-19 11:27:12.988257] Start parameter search for model 'k-Nearest Neighbour
s)'
[2021-05-19 11:27:21.314966] Finish parameter search for model 'k-Nearest Neighbou
rs)' (time: 8 seconds)

=== train : best params ===
{'n_neighbors': 60, 'weights': 'distance'}
=== train : best score : roc_auc ===
```

```
0.792729244896867


('Decision Tree', DecisionTreeClassifier())
[2021-05-19 11:27:21.314966] Start parameter search for model 'Decision Tree'
[2021-05-19 11:27:21.926945] Finish parameter search for model 'Decision Tree' (ti
me: 0 seconds)

=== train : best params ===
{'criterion': 'entropy', 'max_depth': 5}
=== train : best score : roc_auc ===
0.7977663159694495


('Extra Tree', ExtraTreesClassifier())
[2021-05-19 11:27:21.927911] Start parameter search for model 'Extra Tree'
[2021-05-19 11:27:25.129397] Finish parameter search for model 'Extra Tree' (time:
3 seconds)

=== train : best params ===
{'max_features': 'auto', 'n_estimators': 100}
=== train : best score : roc_auc ===
0.8087055266135608


('Gradient Boosting', GradientBoostingClassifier())
[2021-05-19 11:27:25.129397] Start parameter search for model 'Gradient Boosting'
[2021-05-19 11:27:42.466574] Finish parameter search for model 'Gradient Boosting'
(time: 17 seconds)

=== train : best params ===
{'learning_rate': 0.01, 'max_depth': 8, 'max_features': 6, 'n_estimators': 100}
=== train : best score : roc_auc ===
0.8135628779363706


('Adaptive Boosting', AdaBoostClassifier())
[2021-05-19 11:27:42.467570] Start parameter search for model 'Adaptive Boosting'
[2021-05-19 11:27:46.274365] Finish parameter search for model 'Adaptive Boosting'
(time: 3 seconds)

=== train : best params ===
{'learning_rate': 0.2, 'n_estimators': 100}
=== train : best score : roc_auc ===
0.8081015102680386


('Suppport Vector Classfier', SVC(probability=True))
[2021-05-19 11:27:46.275331] Start parameter search for model 'Suppport Vector Cla
ssfier'
[2021-05-19 11:31:34.541045] Finish parameter search for model 'Suppport Vector Cl
assfier' (time: 228 seconds)

=== train : best params ===
{'C': 1, 'gamma': 0.1, 'kernel': 'rbf'}
=== train : best score : roc_auc ===
0.788727440436914


('Neural Network', MLPClassifier())
[2021-05-19 11:31:34.542009] Start parameter search for model 'Neural Network'
```

```
[2021-05-19 11:33:58.605657] Finish parameter search for model 'Neural Network' (t
ime: 144 seconds)

=== train : best params ===
{'activation': 'logistic', 'alpha': 0.05, 'hidden_layer_sizes': (50,), 'learning_r
ate': 'constant'}
=== train : best score : roc_auc ===
0.8106889646177967



Confusion Matrix:
[[806  75]
 [428 571]]

Classification Report:
              precision    recall  f1-score   support

         0.0       0.65      0.91      0.76       881
         1.0       0.88      0.57      0.69       999

    accuracy                           0.73      1880
   macro avg       0.77      0.74      0.73      1880
weighted avg       0.78      0.73      0.73      1880
```

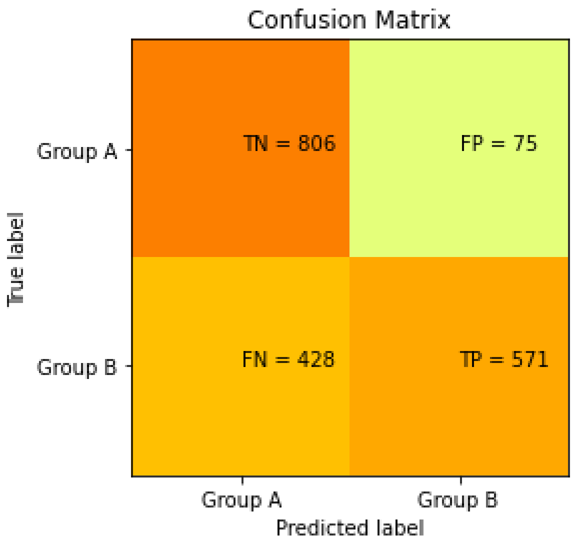


```
AUC Score : 0.7432205190434475

Accuracy Of the Model(LogisticRegression): 0.7324468085106383
```

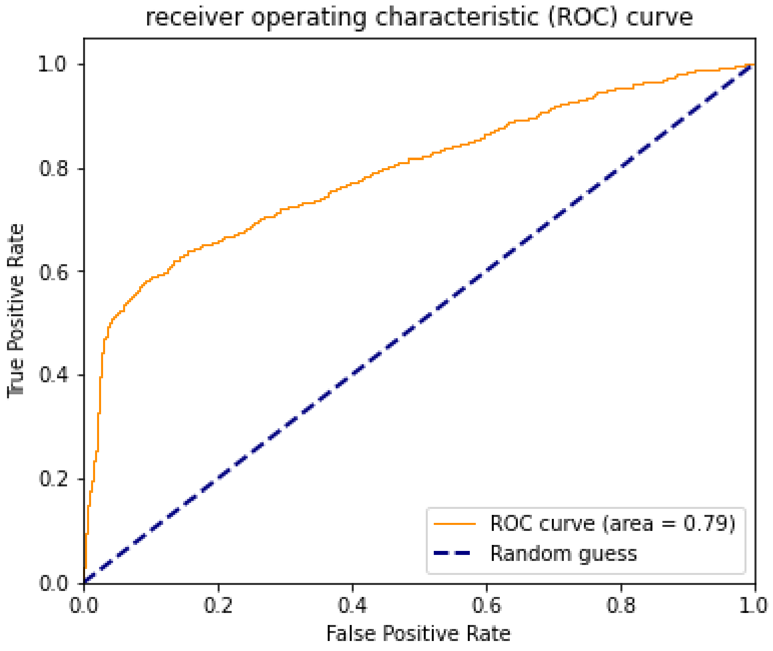


```
ROC_AUC Score of the model(LogisticRegression): 0.7928359687724046


Confusion Matrix:
[[779 102]
 [399 600]]

Classification Report:
              precision    recall  f1-score   support

         0.0       0.66      0.88      0.76       881
         1.0       0.85      0.60      0.71       999

    accuracy                           0.73      1880
   macro avg       0.76      0.74      0.73      1880
weighted avg       0.76      0.73      0.73      1880
```

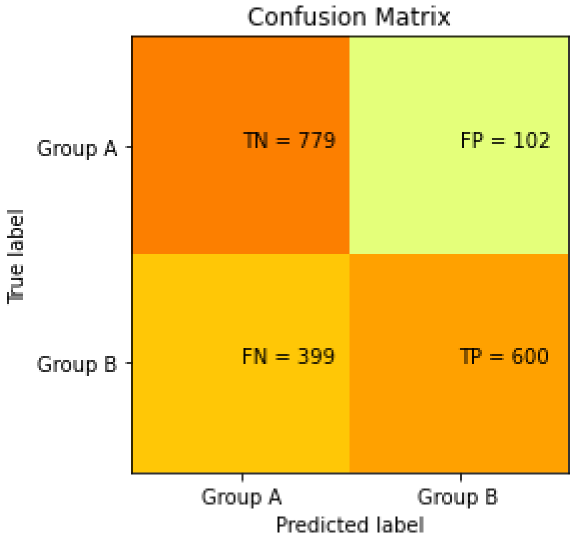

AUC Score : 0.7424115375307203

Accuracy Of the Model(RandomForestClassifier): 0.7335106382978723

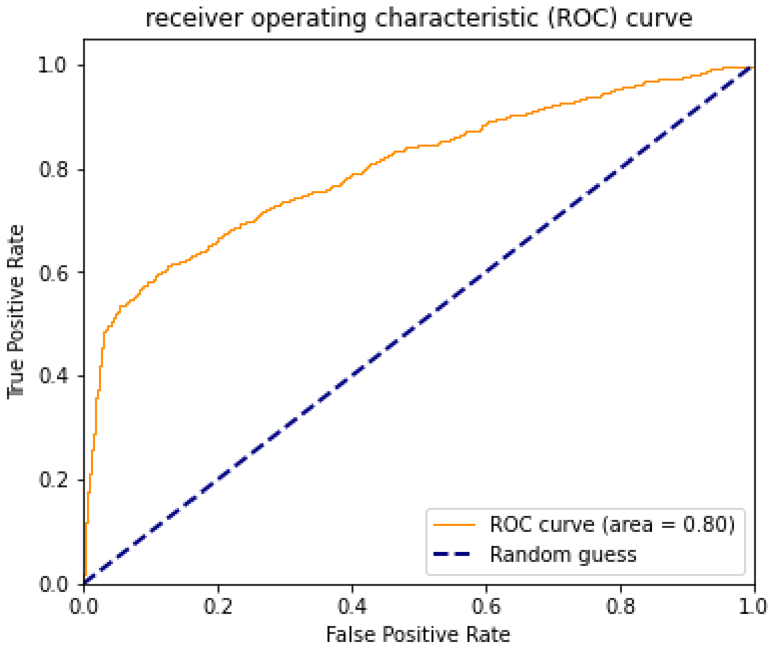

```
ROC_AUC Score of the model(RandomForestClassifier): 0.8022301529679509


Confusion Matrix:
[[787  94]
 [403 596]]

Classification Report:
              precision    recall  f1-score   support

         0.0       0.66      0.89      0.76       881
         1.0       0.86      0.60      0.71       999

    accuracy                           0.74      1880
   macro avg       0.76      0.74      0.73      1880
weighted avg       0.77      0.74      0.73      1880
```

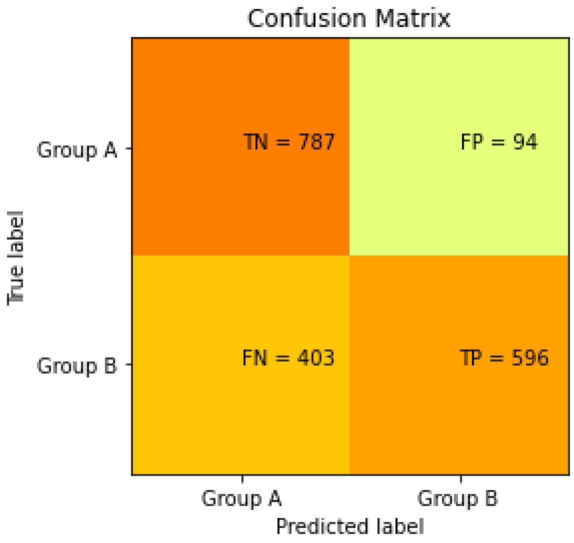


```
AUC Score : 0.744949830647901

Accuracy Of the Model(XGBClassifier): 0.7356382978723405
```

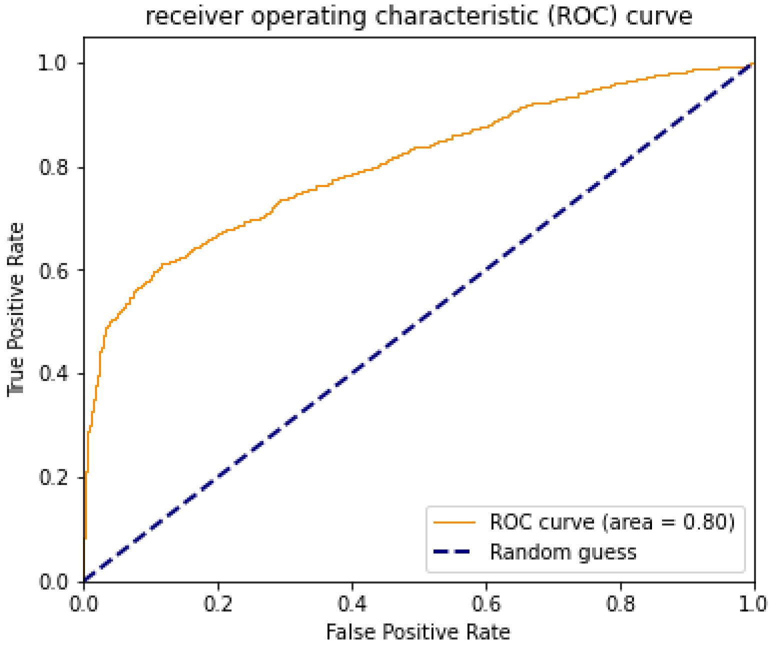

```
ROC_AUC Score of the model(XGBClassifier): 0.8048741136141817


Confusion Matrix:
[[788  93]
 [474 525]]

Classification Report:
              precision    recall  f1-score   support

         0.0       0.62      0.89      0.74       881
         1.0       0.85      0.53      0.65       999

    accuracy                           0.70      1880
   macro avg       0.74      0.71      0.69      1880
weighted avg       0.74      0.70      0.69      1880
```

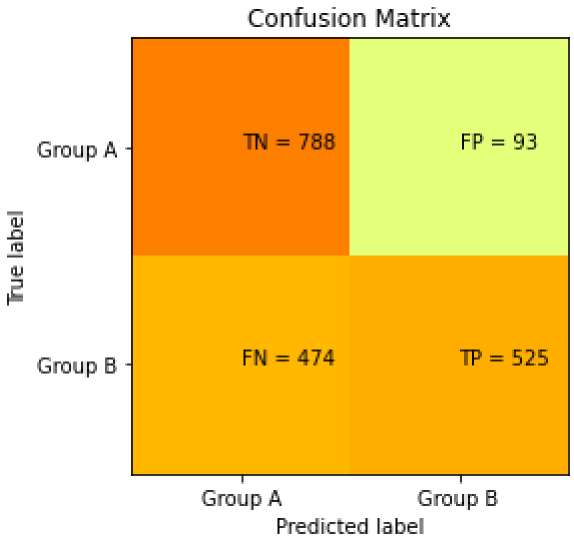


```
AUC Score : 0.7099818320022634

Accuracy Of the Model(KNeighborsClassifier): 0.698404255319149
```

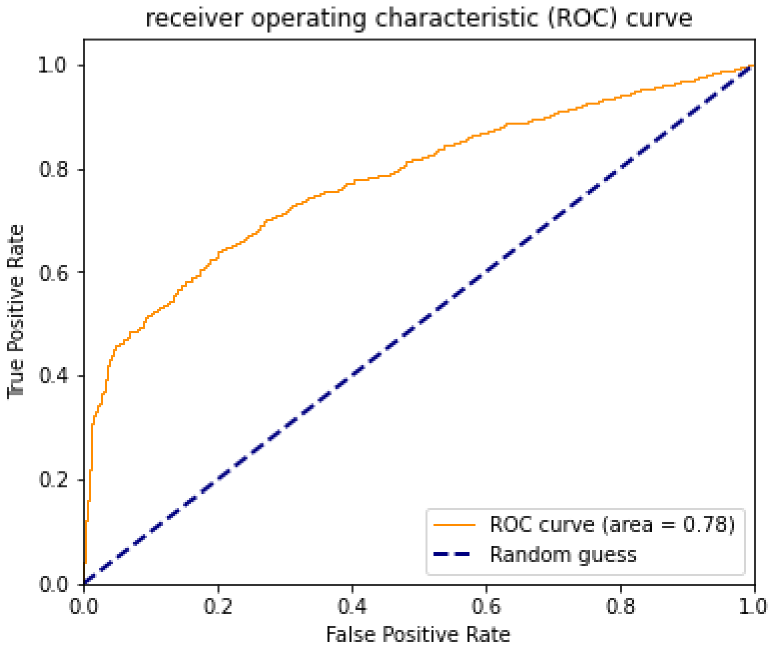

ROC_AUC Score of the model(KNeighborsClassifier): 0.7794389167828442

Confusion Matrix:
[[779 102]
 [407 592]]

Classification Report:

	precision	recall	f1-score	support
0.0	0.66	0.88	0.75	881
1.0	0.85	0.59	0.70	999
accuracy			0.73	1880
macro avg	0.75	0.74	0.73	1880
weighted avg	0.76	0.73	0.72	1880

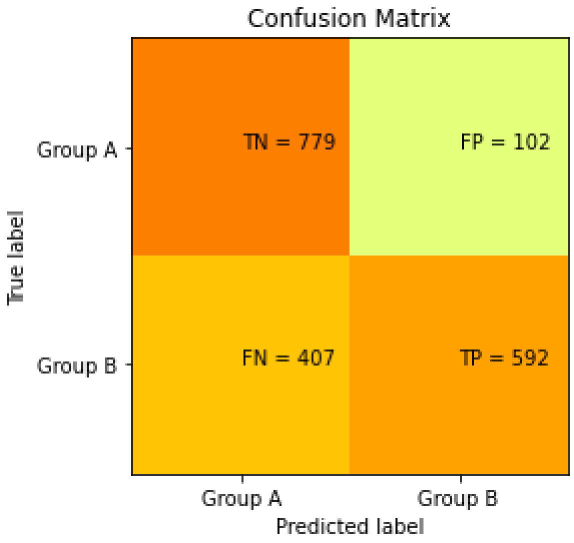

AUC Score : 0.7384075335267163

Accuracy Of the Model(DecisionTreeClassifier): 0.7292553191489362

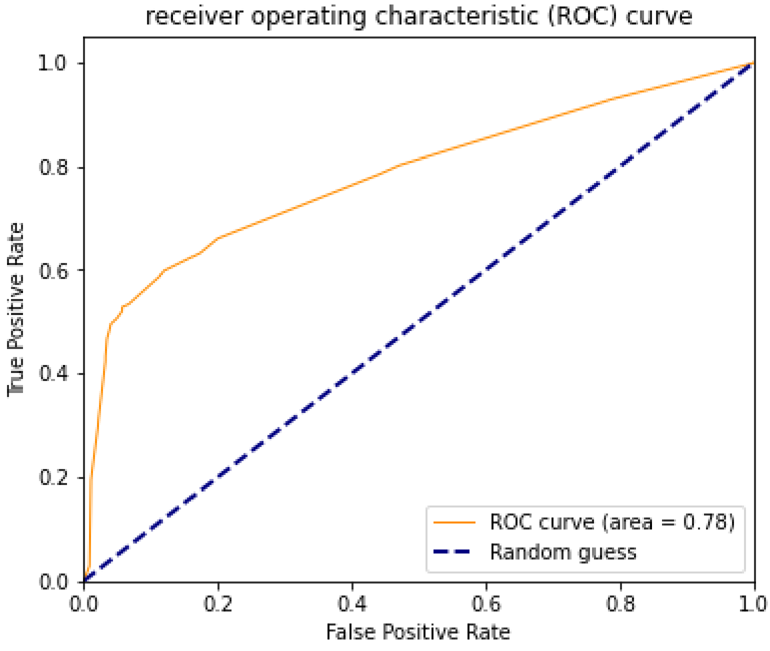

ROC_AUC Score of the model(DecisionTreeClassifier): 0.7808637241100352

```
Confusion Matrix:
[[776 105]
 [414 585]]

Classification Report:
              precision    recall  f1-score   support

         0.0       0.65      0.88      0.75       881
         1.0       0.85      0.59      0.69       999

    accuracy                           0.72      1880
   macro avg       0.75      0.73      0.72      1880
weighted avg       0.76      0.72      0.72      1880
```

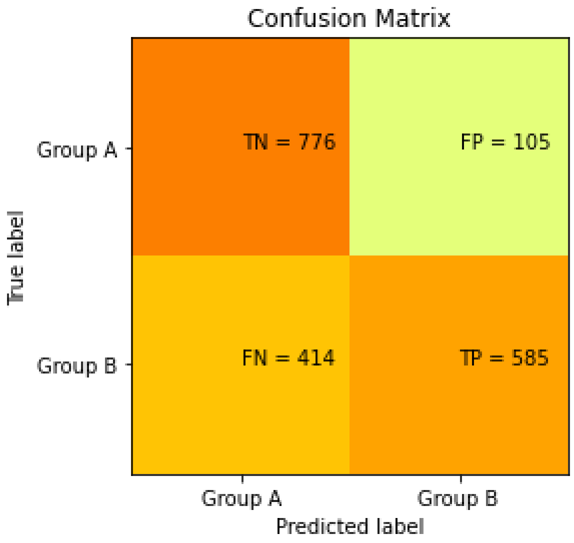

AUC Score : 0.7332014193535192

Accuracy Of the Model(ExtraTreesClassifier): 0.7239361702127659

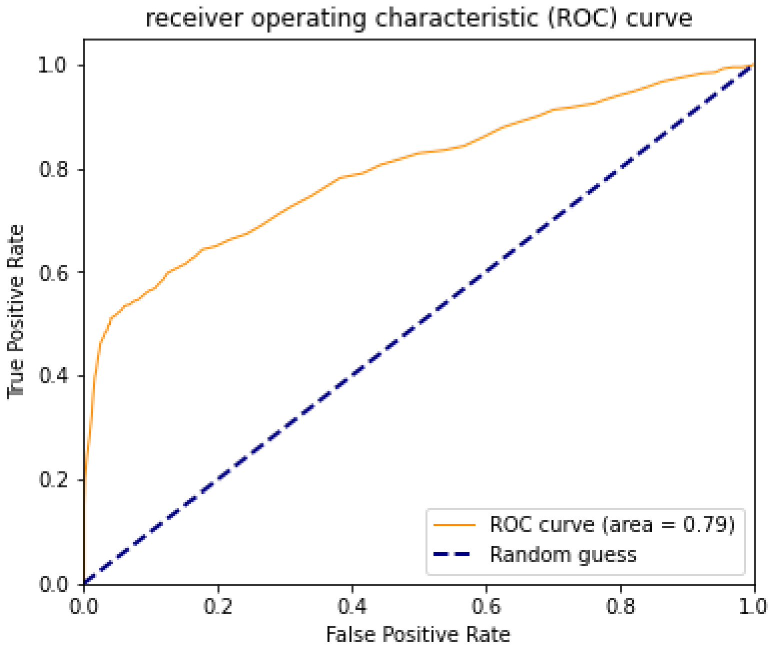

```
ROC_AUC Score of the model(ExtraTreesClassifier): 0.7937341427693301


Confusion Matrix:
[[776 105]
 [399 600]]

Classification Report:
              precision    recall  f1-score   support

         0.0       0.66      0.88      0.75       881
         1.0       0.85      0.60      0.70       999

    accuracy                           0.73      1880
   macro avg       0.76      0.74      0.73      1880
weighted avg       0.76      0.73      0.73      1880
```

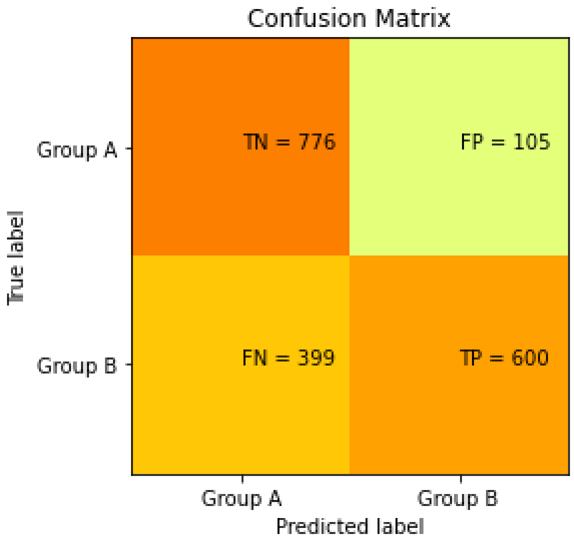


```
AUC Score : 0.7407089268610267

Accuracy Of the Model(GradientBoostingClassifier): 0.7319148936170212
```

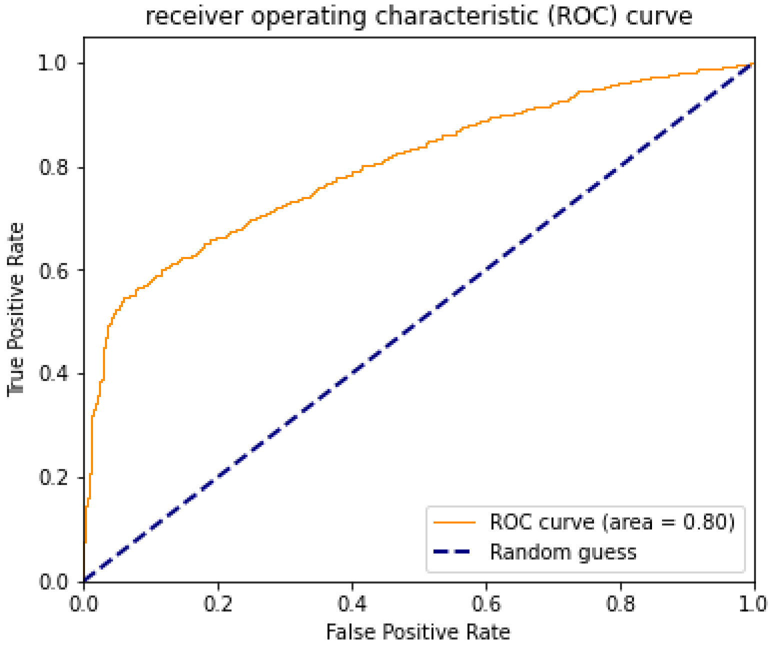

```
ROC_AUC Score of the model(GradientBoostingClassifier): 0.8023551360668273


Confusion Matrix:
[[797  84]
 [427 572]]

Classification Report:
              precision    recall  f1-score   support

         0.0       0.65      0.90      0.76       881
         1.0       0.87      0.57      0.69       999

    accuracy                           0.73      1880
   macro avg       0.76      0.74      0.72      1880
weighted avg       0.77      0.73      0.72      1880
```

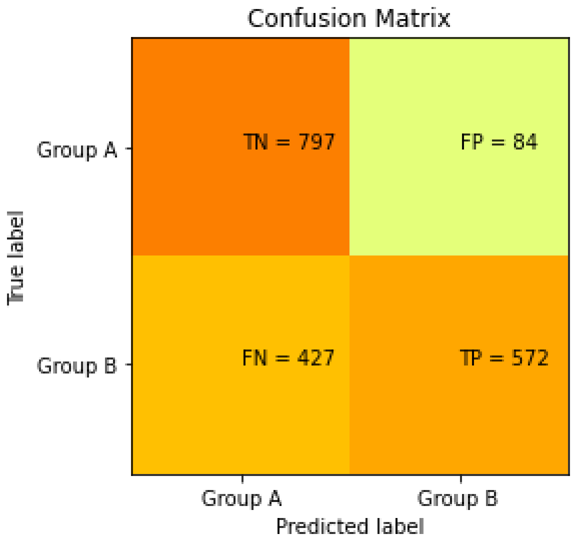


```
AUC Score : 0.7386131875348675

Accuracy Of the Model(AdaBoostClassifier): 0.7281914893617021
```

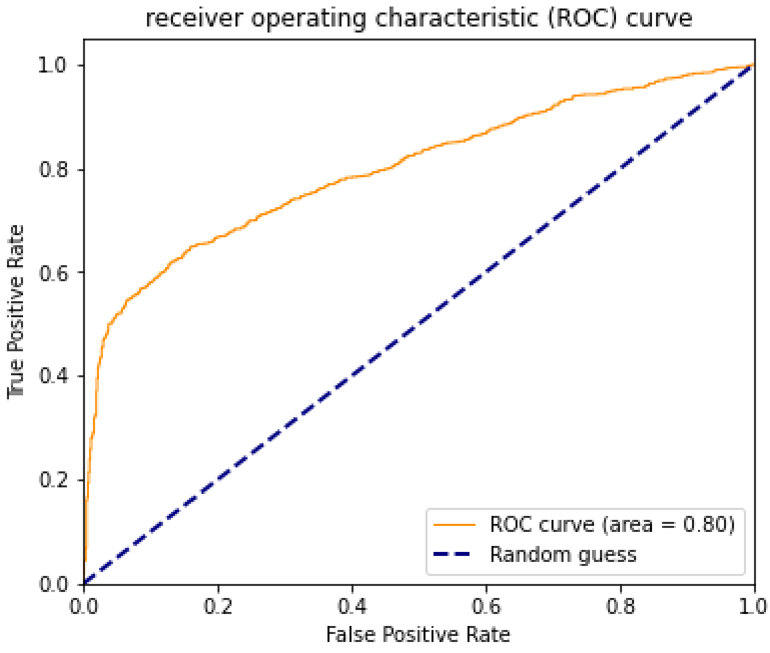

ROC_AUC Score of the model(AdaBoostClassifier): 0.7984556633818836

```
Confusion Matrix:
[[682 199]
 [349 650]]

Classification Report:
              precision    recall  f1-score   support

         0.0       0.66      0.77      0.71       881
         1.0       0.77      0.65      0.70       999

    accuracy                           0.71      1880
   macro avg       0.71      0.71      0.71      1880
weighted avg       0.72      0.71      0.71      1880
```

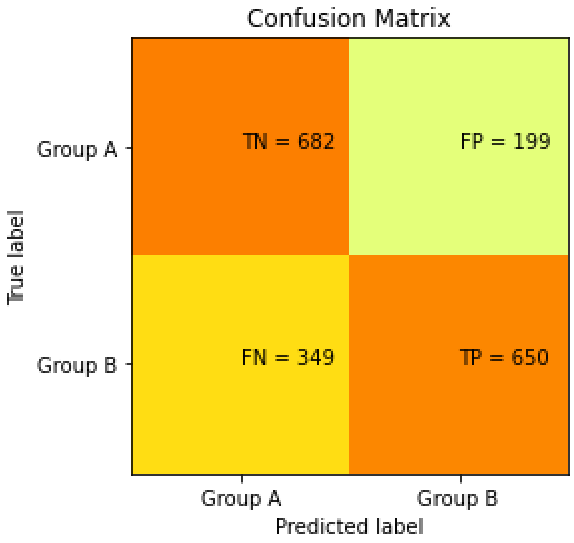

AUC Score : 0.7123854842356545

Accuracy Of the Model(SVC): 0.7085106382978723

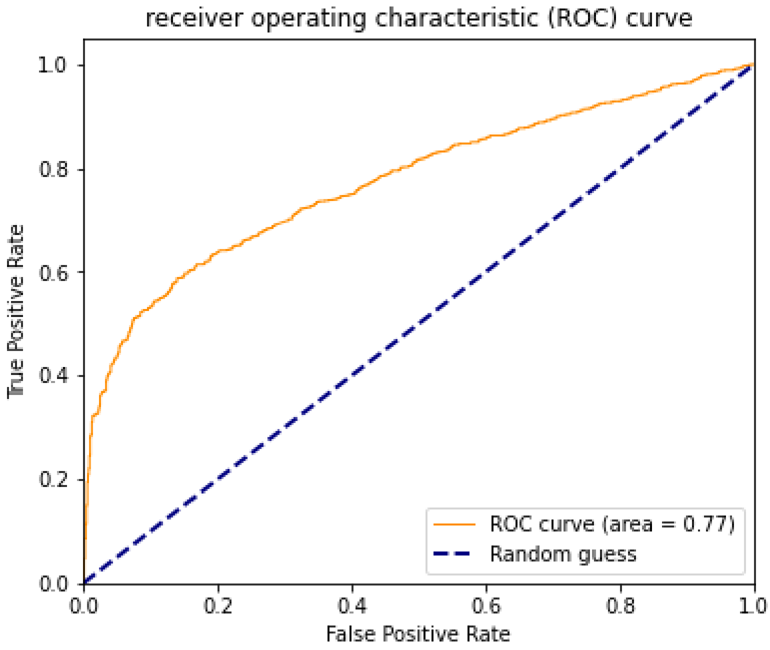

```
ROC_AUC Score of the model(SVC): 0.7739135276025175


Confusion Matrix:
[[783  98]
 [401 598]]

Classification Report:
              precision    recall  f1-score   support

         0.0       0.66      0.89      0.76       881
         1.0       0.86      0.60      0.71       999

    accuracy                           0.73      1880
   macro avg       0.76      0.74      0.73      1880
weighted avg       0.77      0.73      0.73      1880
```

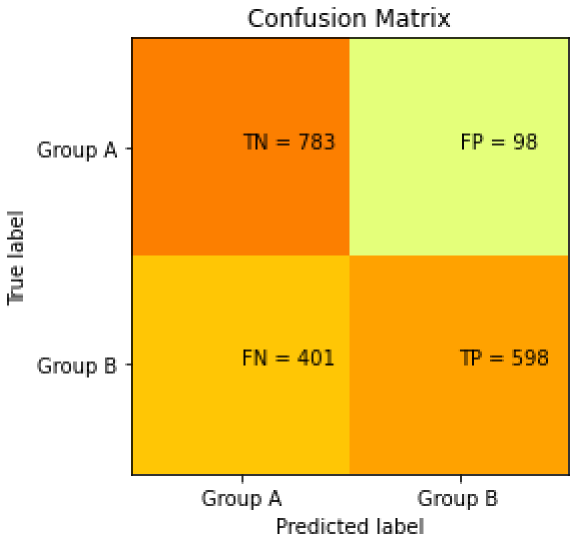


```
AUC Score : 0.7436806840893107

Accuracy Of the Model(MLPClassifier): 0.7345744680851064
```

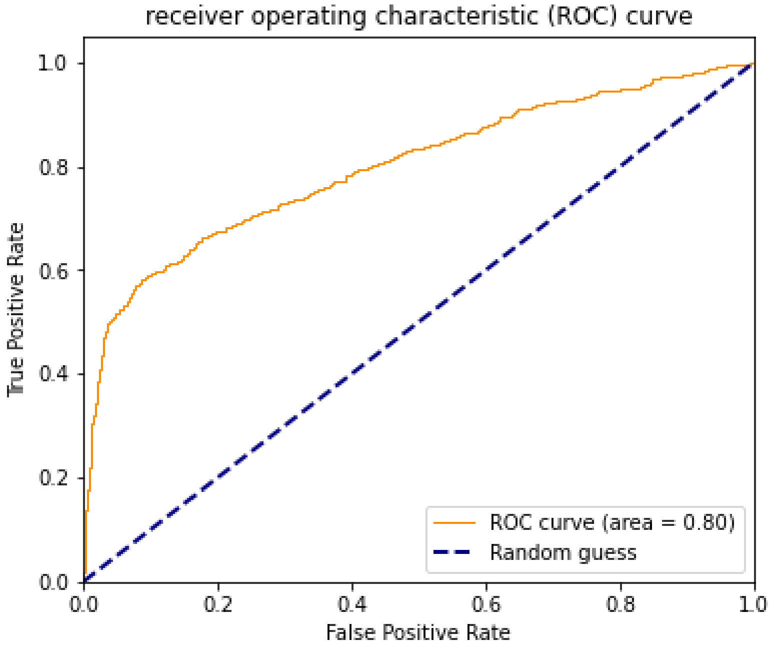

```
ROC_AUC Score of the model(MLPClassifier): 0.8007053591616589

[('XGBClassifier', 0.7356382978723405), ('MLPClassifier', 0.7345744680851064), ('RandomForestClassifier', 0.7335106382978723), ('LogisticRegression', 0.7324468085106383), ('GradientBoostingClassifier', 0.7319148936170212), ('DecisionTreeClassifier', 0.7292553191489362), ('AdaBoostClassifier', 0.7281914893617021), ('ExtraTreesClassifier', 0.7239361702127659), ('SVC', 0.7085106382978723), ('KNeighborsClassifier', 0.698404255319149)]


sorted_total_auc:
 [('XGBClassifier', 0.8048741136141817), ('GradientBoostingClassifier', 0.8023551360668273), ('RandomForestClassifier', 0.8022301529679509), ('MLPClassifier', 0.8007053591616589), ('AdaBoostClassifier', 0.7984556633818836), ('ExtraTreesClassifier', 0.7937341427693301), ('LogisticRegression', 0.7928359687724046), ('DecisionTreeClassifier', 0.7808637241100352), ('KNeighborsClassifier', 0.7794389167828442), ('SVC', 0.7739135276025175)]
```

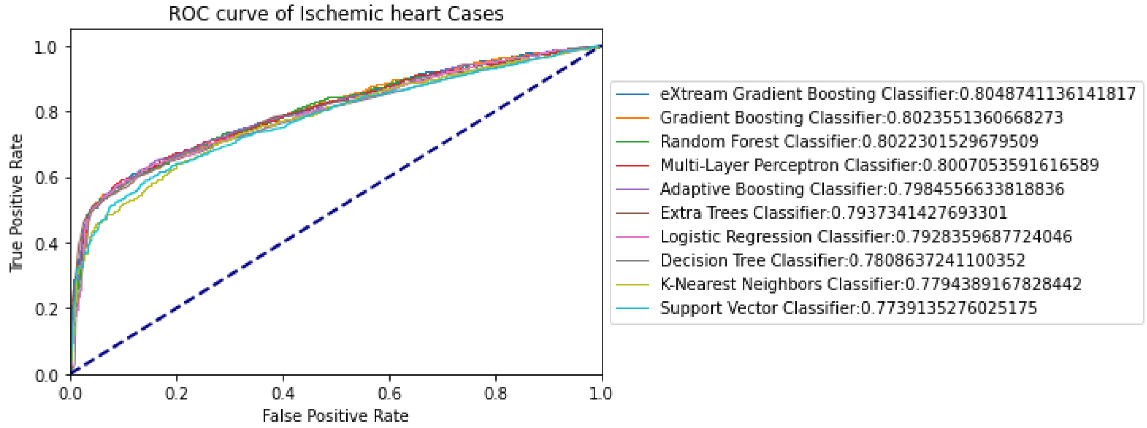

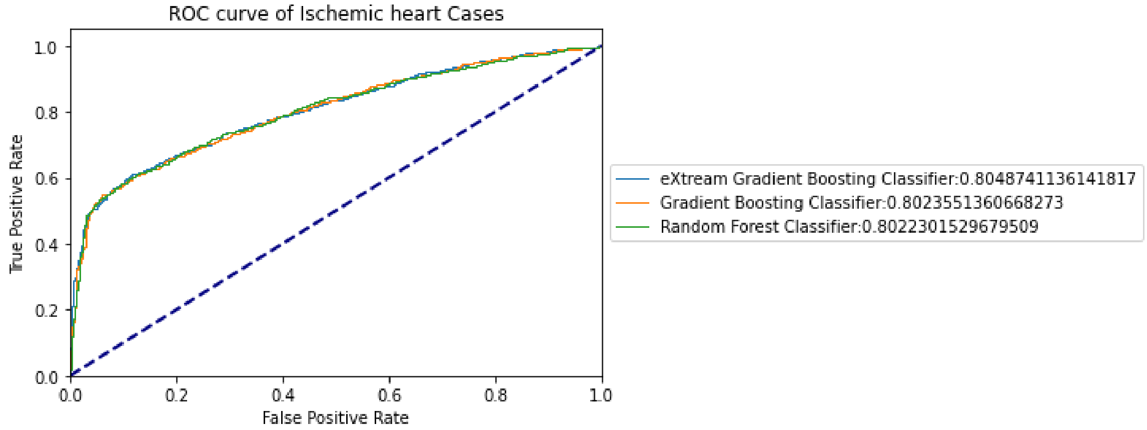

```
random state :   12360
9398
result_train_filename: data/train_test_data_ischemic heart_2013_20210519_113406.cs
v
total count : 9398, df_train count : 7518, df_test count : 1880
(7518, 38)
(1880, 38)
('Logistic Regression', LogisticRegression())
[2021-05-19 11:34:06.440192] Start parameter search for model 'Logistic Regressio
n'
[2021-05-19 11:34:06.751961] Finish parameter search for model 'Logistic Regressio
n' (time: 0 seconds)

=== train : best params ===
{'C': 100.0, 'penalty': 'l2', 'solver': 'lbfgs'}
=== train : best score : roc_auc ===
0.8019801938389309



('Random Forest', RandomForestClassifier())
[2021-05-19 11:34:06.751961] Start parameter search for model 'Random Forest'
[2021-05-19 11:34:11.001168] Finish parameter search for model 'Random Forest' (ti
me: 4 seconds)

=== train : best params ===
{'criterion': 'gini', 'max_depth': 10, 'max_features': 'auto', 'n_estimators': 10
0}
=== train : best score : roc_auc ===
0.8099224836502388



('Extreme Gradient Boosting)', XGBClassifier(base_score=None, booster=None, colsam
ple_bylevel=None,
              colsample_bynode=None, colsample_bytree=None, gamma=None,
              gpu_id=None, importance_type='gain', interaction_constraints=None,
              learning_rate=None, max_delta_step=None, max_depth=None,
              min_child_weight=None, missing=nan, monotone_constraints=None,
              n_estimators=100, n_jobs=None, num_parallel_tree=None,
              random_state=None, reg_alpha=None, reg_lambda=None,
              scale_pos_weight=None, subsample=None, tree_method=None,
              validate_parameters=None, verbosity=None))
[2021-05-19 11:34:11.002165] Start parameter search for model 'Extreme Gradient Bo
osting)'
[2021-05-19 11:34:36.075789] Finish parameter search for model 'Extreme Gradient B
oosting)' (time: 25 seconds)

=== train : best params ===
{'learning_rate': 0.075, 'max_depth': 5, 'n_estimators': 50, 'subsample': 0.8}
=== train : best score : roc_auc ===
0.8111640582463784



('k-Nearest Neighbours)', KNeighborsClassifier())
[2021-05-19 11:34:36.076786] Start parameter search for model 'k-Nearest Neighbour
s)'
[2021-05-19 11:34:43.960111] Finish parameter search for model 'k-Nearest Neighbou
rs)' (time: 7 seconds)

=== train : best params ===
{'n_neighbors': 60, 'weights': 'distance'}
=== train : best score : roc_auc ===
```

```
0.7885933272647379


('Decision Tree', DecisionTreeClassifier())
[2021-05-19 11:34:43.960111] Start parameter search for model 'Decision Tree'
[2021-05-19 11:34:44.528170] Finish parameter search for model 'Decision Tree' (ti
me: 0 seconds)

=== train : best params ===
{'criterion': 'gini', 'max_depth': 5}
=== train : best score : roc_auc ===
0.7918095393922406


('Extra Tree', ExtraTreesClassifier())
[2021-05-19 11:34:44.528170] Start parameter search for model 'Extra Tree'
[2021-05-19 11:34:47.352901] Finish parameter search for model 'Extra Tree' (time:
2 seconds)

=== train : best params ===
{'max_features': 'log2', 'n_estimators': 100}
=== train : best score : roc_auc ===
0.8040807661364562


('Gradient Boosting', GradientBoostingClassifier())
[2021-05-19 11:34:47.352901] Start parameter search for model 'Gradient Boosting'
[2021-05-19 11:35:03.227586] Finish parameter search for model 'Gradient Boosting'
(time: 15 seconds)

=== train : best params ===
{'learning_rate': 0.01, 'max_depth': 8, 'max_features': 6, 'n_estimators': 100}
=== train : best score : roc_auc ===
0.80974123288884


('Adaptive Boosting', AdaBoostClassifier())
[2021-05-19 11:35:03.227586] Start parameter search for model 'Adaptive Boosting'
[2021-05-19 11:35:06.818652] Finish parameter search for model 'Adaptive Boosting'
(time: 3 seconds)

=== train : best params ===
{'learning_rate': 0.2, 'n_estimators': 100}
=== train : best score : roc_auc ===
0.803995475821993


('Suppport Vector Classfier', SVC(probability=True))
[2021-05-19 11:35:06.819650] Start parameter search for model 'Suppport Vector Cla
ssfier'
[2021-05-19 11:39:12.526691] Finish parameter search for model 'Suppport Vector Cl
assfier' (time: 245 seconds)

=== train : best params ===
{'C': 1, 'gamma': 0.1, 'kernel': 'rbf'}
=== train : best score : roc_auc ===
0.7844347317002673


('Neural Network', MLPClassifier())
[2021-05-19 11:39:12.527689] Start parameter search for model 'Neural Network'
```

```
[2021-05-19 11:41:41.924370] Finish parameter search for model 'Neural Network' (t
ime: 149 seconds)

=== train : best params ===
{'activation': 'logistic', 'alpha': 0.05, 'hidden_layer_sizes': (50,), 'learning_r
ate': 'adaptive'}
=== train : best score : roc_auc ===
0.8064316712726263



Confusion Matrix:
[[818  63]
 [430 569]]

Classification Report:
              precision    recall  f1-score   support

         0.0       0.66      0.93      0.77       881
         1.0       0.90      0.57      0.70       999

    accuracy                           0.74      1880
   macro avg       0.78      0.75      0.73      1880
weighted avg       0.79      0.74      0.73      1880
```

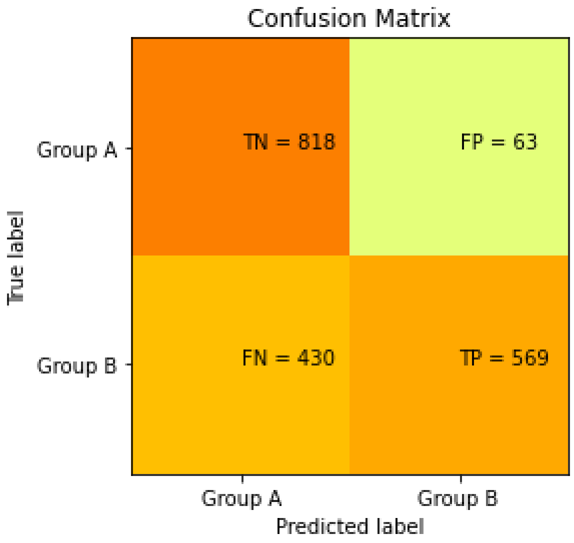


```
AUC Score : 0.7490299607212207

Accuracy Of the Model(LogisticRegression): 0.7377659574468085
```

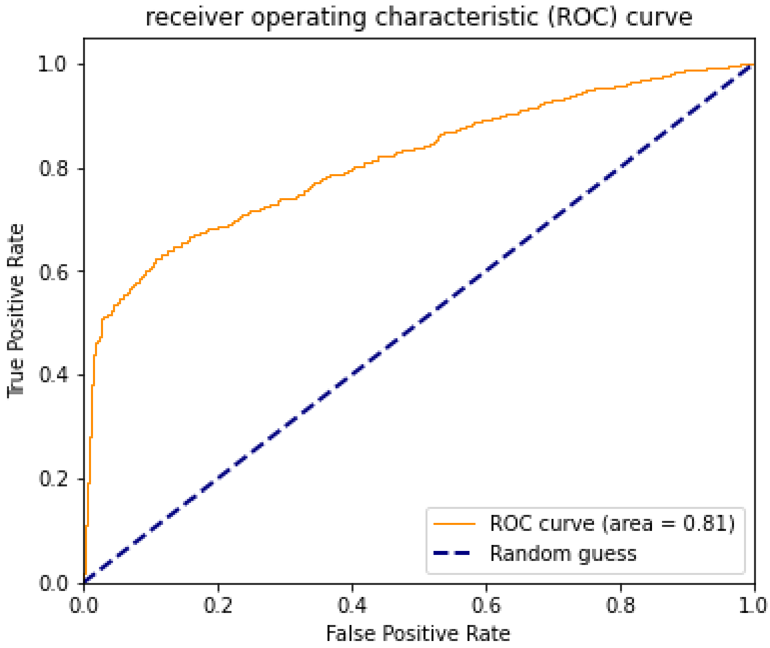


```
ROC_AUC Score of the model(LogisticRegression): 0.8139797004723226


Confusion Matrix:
[[802  79]
 [399 600]]

Classification Report:
              precision    recall  f1-score   support

         0.0       0.67      0.91      0.77       881
         1.0       0.88      0.60      0.72       999

    accuracy                           0.75      1880
   macro avg       0.78      0.76      0.74      1880
weighted avg       0.78      0.75      0.74      1880
```

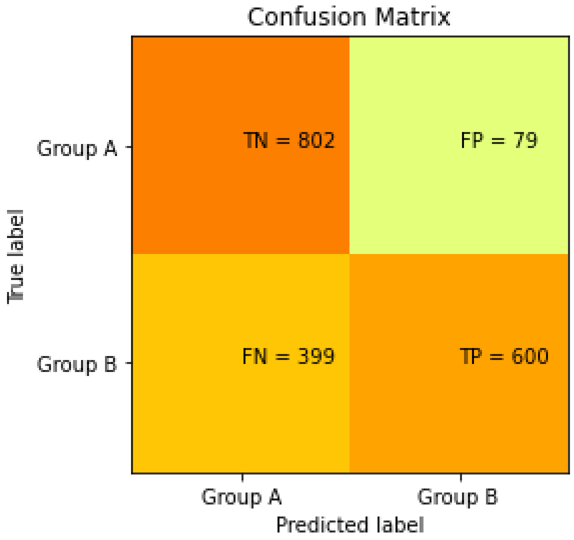

AUC Score : 0.7554648859983707

Accuracy Of the Model(RandomForestClassifier): 0.7457446808510638

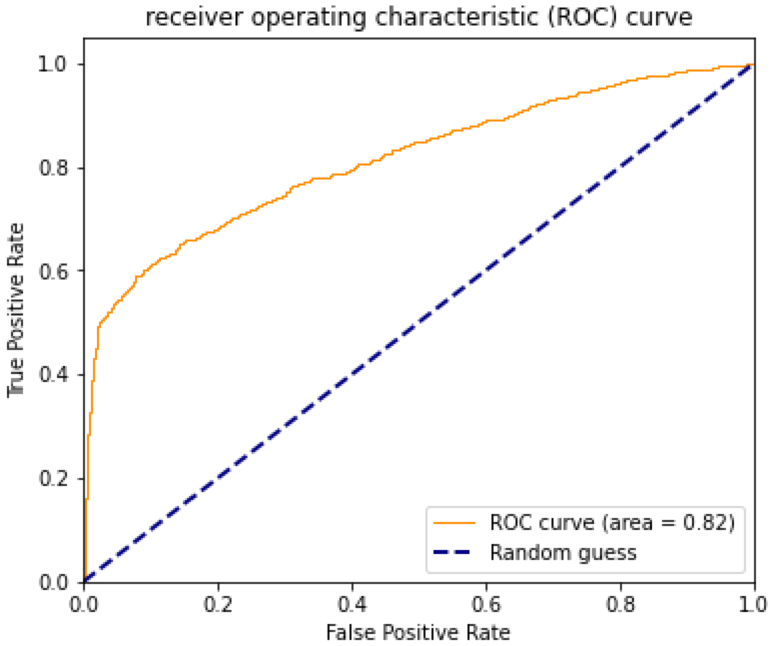

```
ROC_AUC Score of the model(RandomForestClassifier): 0.8160385129738137


Confusion Matrix:
[[806  75]
 [415 584]]

Classification Report:
              precision    recall  f1-score   support

         0.0       0.66      0.91      0.77       881
         1.0       0.89      0.58      0.70       999

    accuracy                           0.74      1880
   macro avg       0.77      0.75      0.74      1880
weighted avg       0.78      0.74      0.73      1880
```

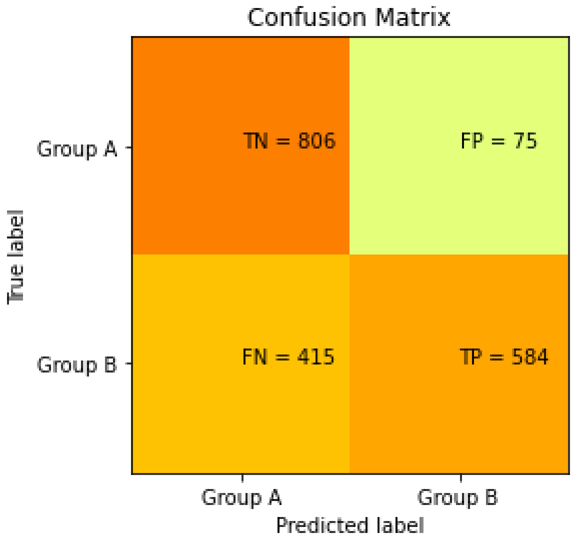


```
AUC Score : 0.7497270255499542

Accuracy Of the Model(XGBClassifier): 0.7393617021276596
```

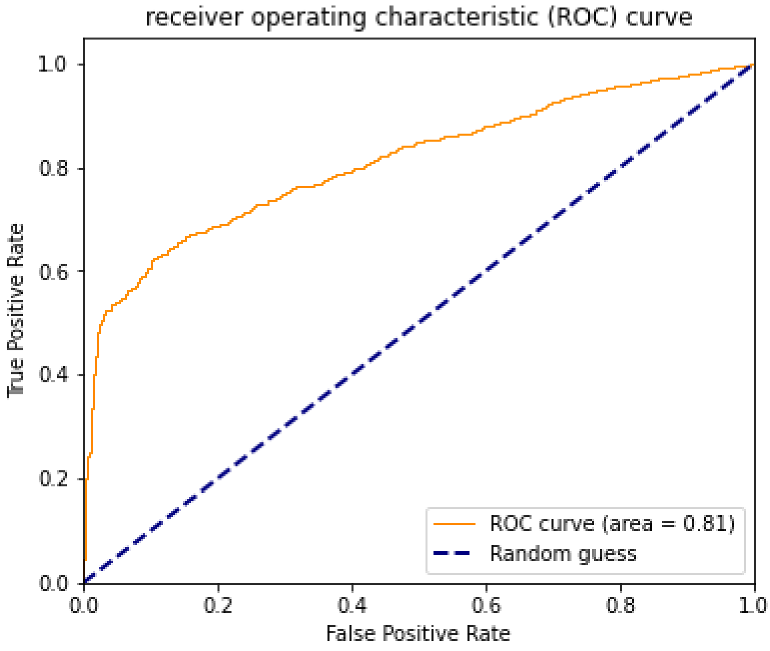

```
ROC_AUC Score of the model(XGBClassifier): 0.8126412451043552


Confusion Matrix:
[[796  85]
 [466 533]]

Classification Report:
              precision    recall  f1-score   support

         0.0       0.63      0.90      0.74       881
         1.0       0.86      0.53      0.66       999

    accuracy                           0.71      1880
   macro avg       0.75      0.72      0.70      1880
weighted avg       0.75      0.71      0.70      1880
```

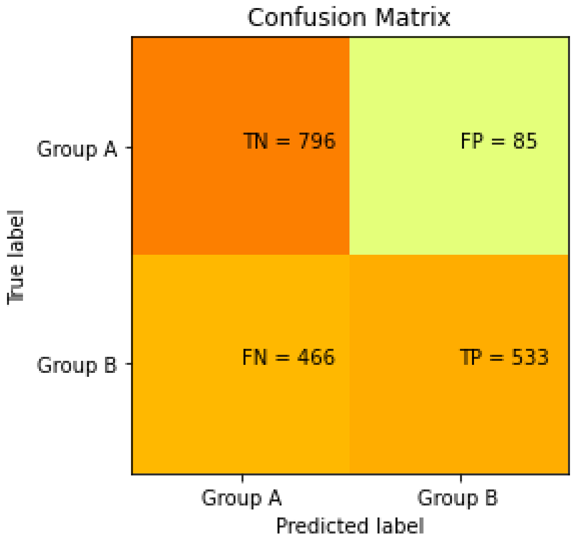


```
AUC Score : 0.7185261311254502

Accuracy Of the Model(KNeighborsClassifier): 0.7069148936170213
```

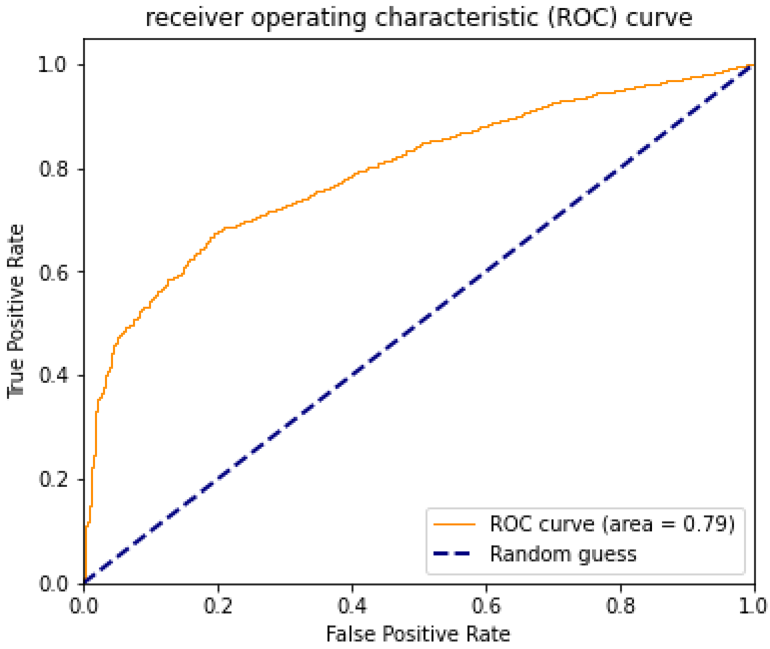

```
ROC_AUC Score of the model(KNeighborsClassifier): 0.7938971889028642


Confusion Matrix:
[[773 108]
 [386 613]]

Classification Report:
              precision    recall  f1-score   support

         0.0       0.67      0.88      0.76       881
         1.0       0.85      0.61      0.71       999

    accuracy                           0.74      1880
   macro avg       0.76      0.75      0.74      1880
weighted avg       0.76      0.74      0.73      1880
```

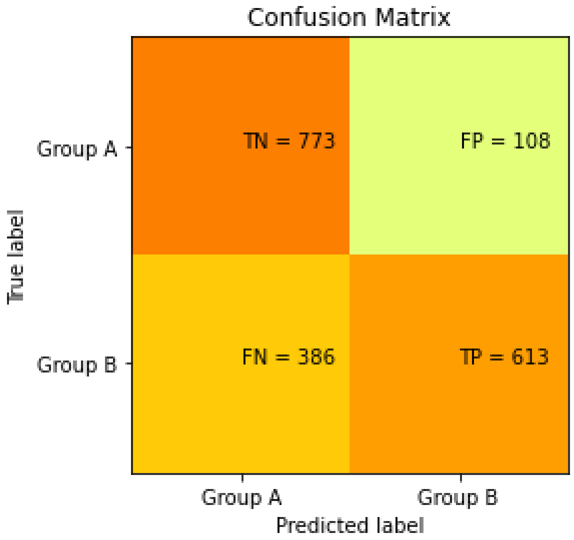


```
AUC Score : 0.7455128226978397

Accuracy Of the Model(DecisionTreeClassifier): 0.7372340425531915
```

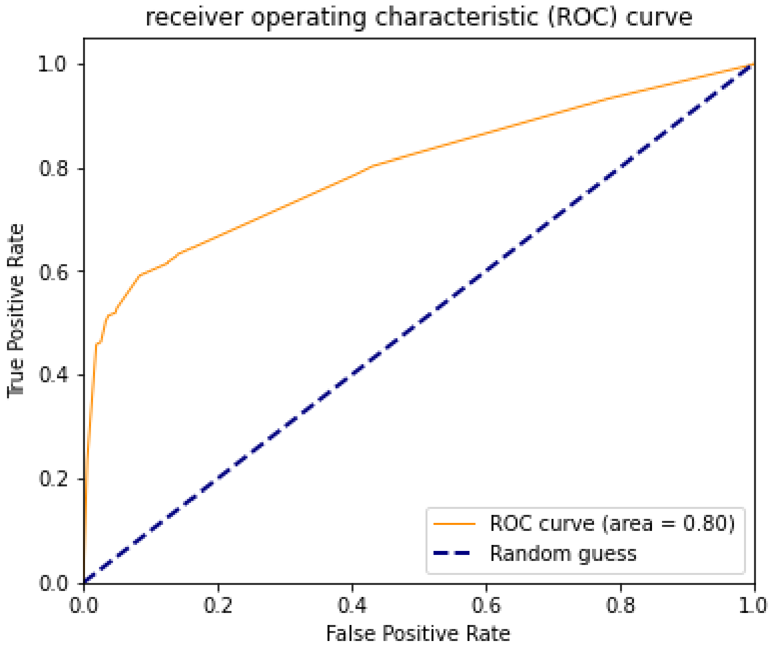

ROC_AUC Score of the model(DecisionTreeClassifier): 0.7971541348385844

```
Confusion Matrix:
[[789  92]
 [418 581]]

Classification Report:
              precision    recall  f1-score   support

         0.0       0.65      0.90      0.76       881
         1.0       0.86      0.58      0.69       999

    accuracy                           0.73      1880
   macro avg       0.76      0.74      0.73      1880
weighted avg       0.77      0.73      0.72      1880
```

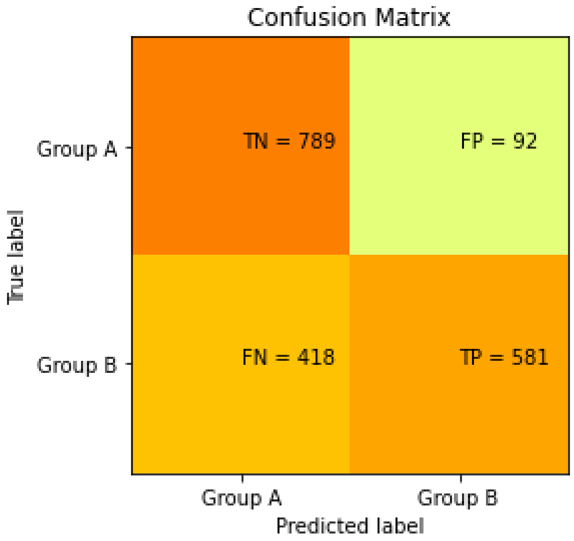

AUC Score : 0.7385773969201892

Accuracy Of the Model(ExtraTreesClassifier): 0.7287234042553191

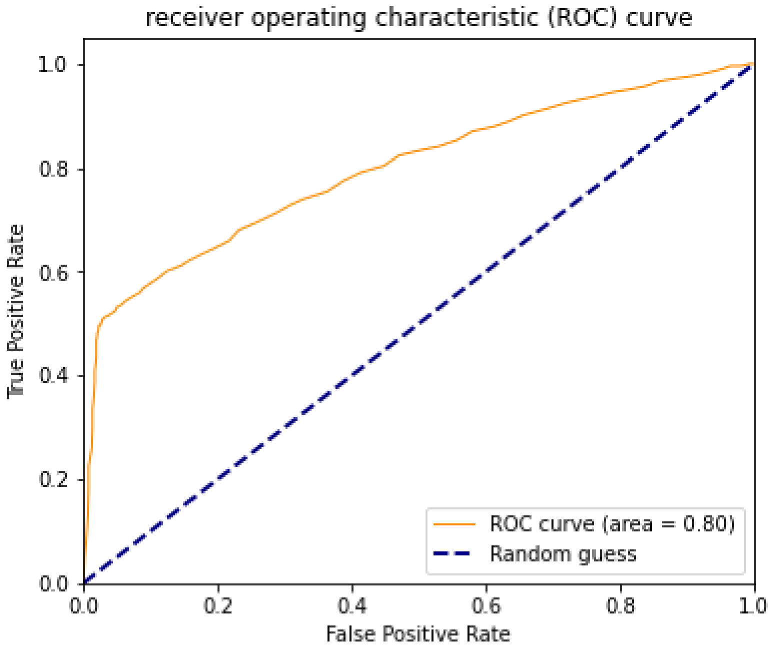

```
ROC_AUC Score of the model(ExtraTreesClassifier): 0.7973012740322615


Confusion Matrix:
[[804  77]
 [405 594]]

Classification Report:
              precision    recall  f1-score   support

         0.0       0.67      0.91      0.77       881
         1.0       0.89      0.59      0.71       999

    accuracy                           0.74      1880
   macro avg       0.78      0.75      0.74      1880
weighted avg       0.78      0.74      0.74      1880
```

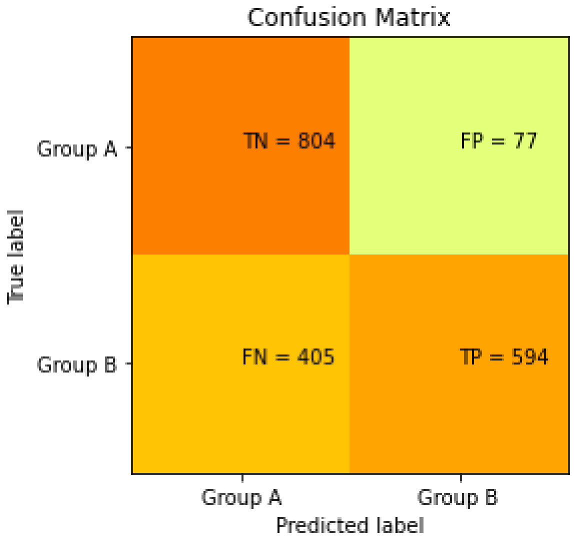


```
AUC Score : 0.7535969567751634

Accuracy Of the Model(GradientBoostingClassifier): 0.7436170212765958
```

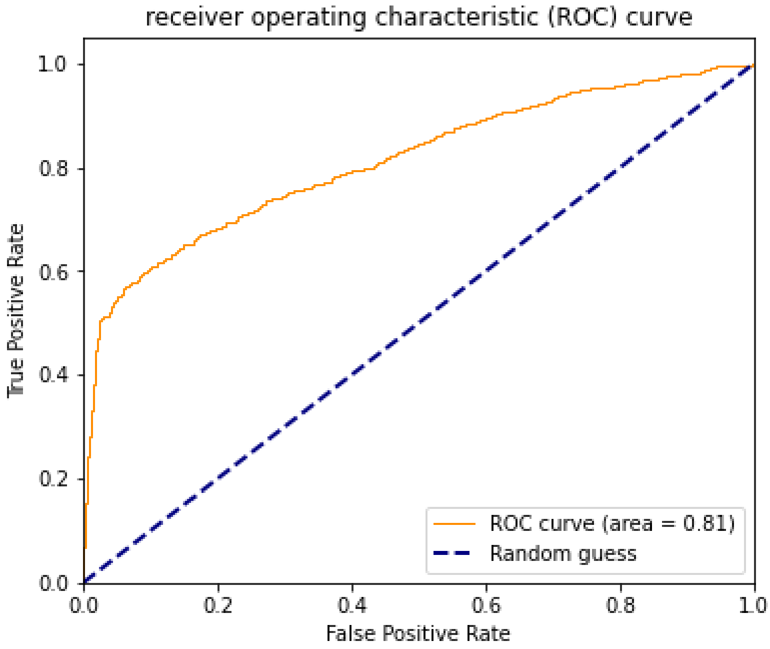

```
ROC_AUC Score of the model(GradientBoostingClassifier): 0.8138035879239057


Confusion Matrix:
[[810  71]
 [429 570]]

Classification Report:
              precision    recall  f1-score   support

         0.0       0.65      0.92      0.76       881
         1.0       0.89      0.57      0.70       999

    accuracy                           0.73      1880
   macro avg       0.77      0.74      0.73      1880
weighted avg       0.78      0.73      0.73      1880
```

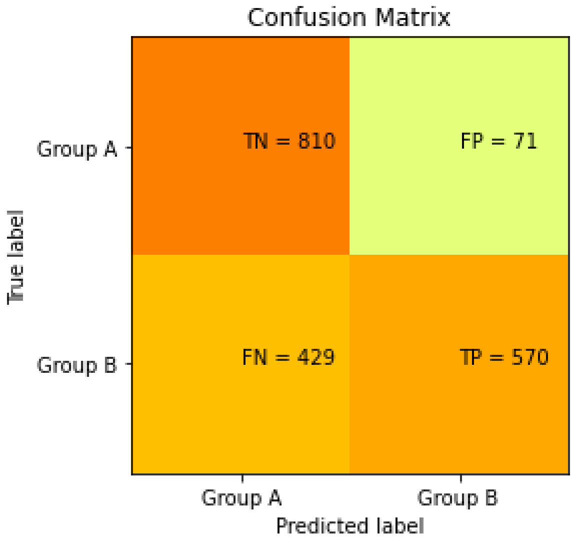


```
AUC Score : 0.7449901661025384

Accuracy Of the Model(AdaBoostClassifier): 0.7340425531914894
```

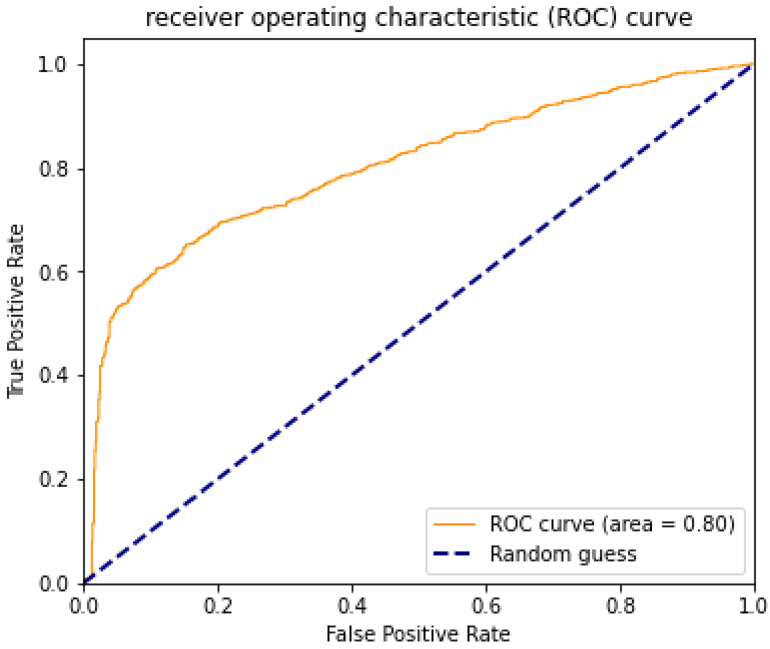

```
ROC_AUC Score of the model(AdaBoostClassifier): 0.801191657037287


Confusion Matrix:
[[702 179]
 [353 646]]

Classification Report:
              precision    recall  f1-score   support

         0.0       0.67      0.80      0.73       881
         1.0       0.78      0.65      0.71       999

    accuracy                           0.72      1880
   macro avg       0.72      0.72      0.72      1880
weighted avg       0.73      0.72      0.72      1880
```

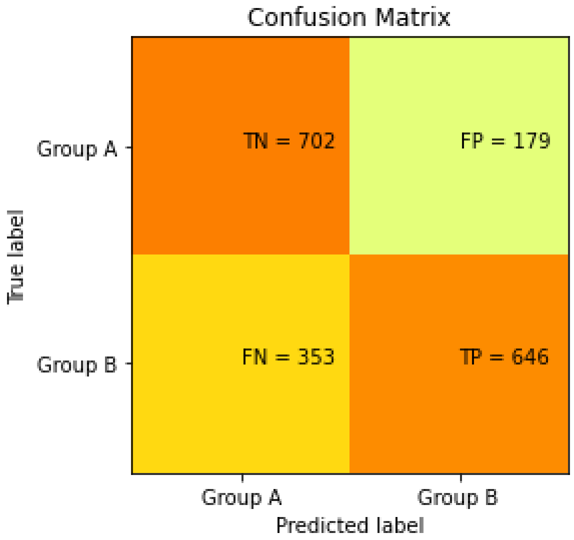


```
AUC Score : 0.7217342200316093

Accuracy Of the Model(SVC): 0.7170212765957447
```

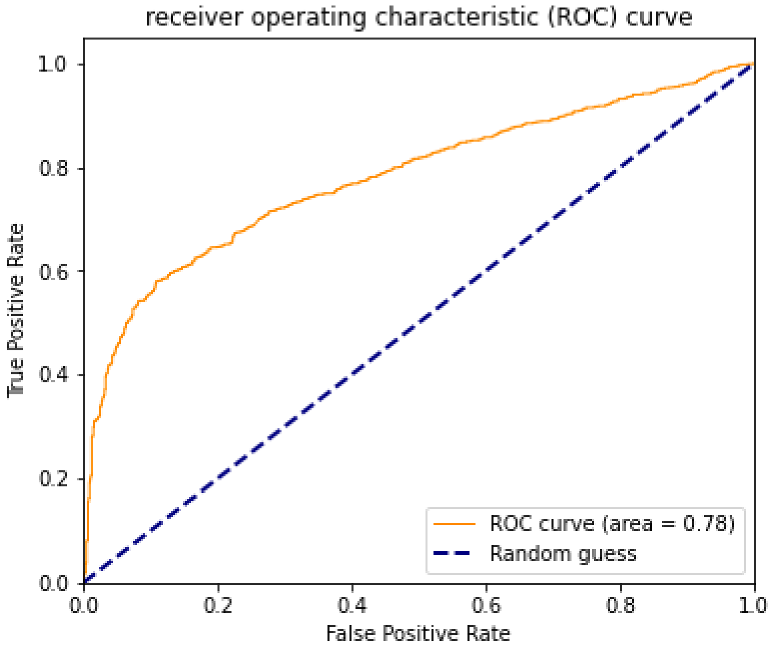

```
ROC_AUC Score of the model(SVC): 0.7798138660794733


Confusion Matrix:
[[790  91]
 [396 603]]

Classification Report:
              precision    recall  f1-score   support

         0.0       0.67      0.90      0.76       881
         1.0       0.87      0.60      0.71       999

    accuracy                           0.74      1880
   macro avg       0.77      0.75      0.74      1880
weighted avg       0.77      0.74      0.74      1880
```

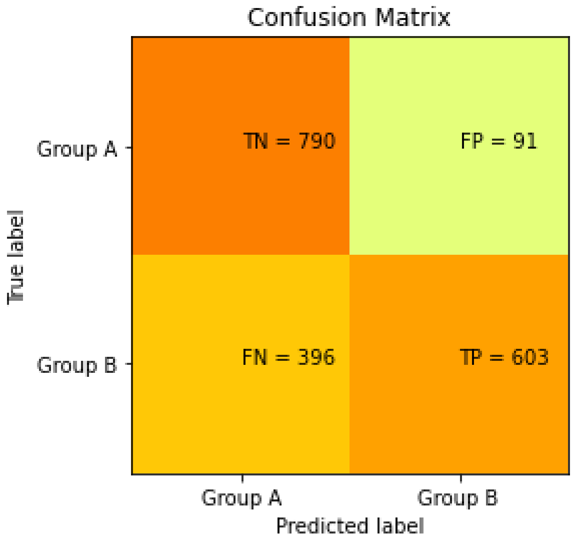


```
AUC Score : 0.7501559448210982

Accuracy Of the Model(MLPClassifier): 0.7409574468085106
```

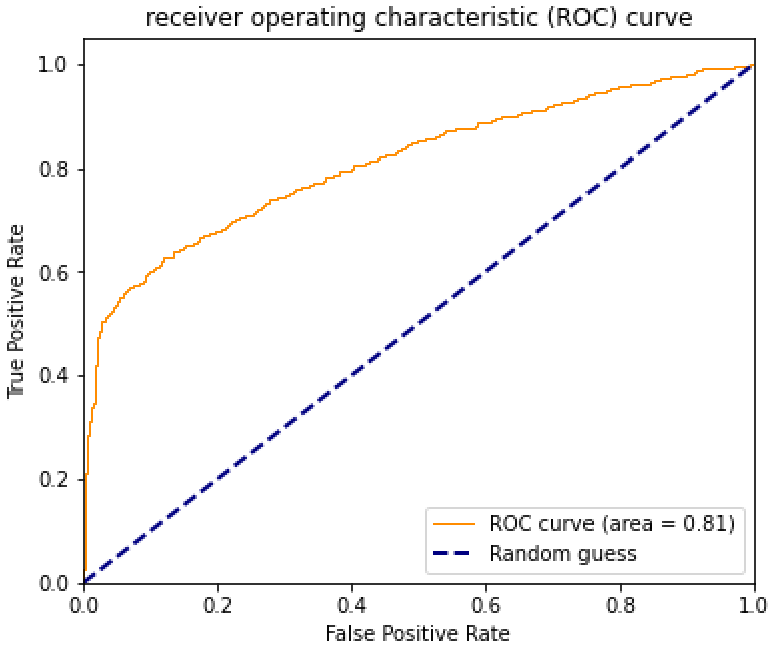

```
ROC_AUC Score of the model(MLPClassifier): 0.8121299506089517

[('RandomForestClassifier', 0.7457446808510638), ('GradientBoostingClassifier', 0.7436170212765958), ('MLPClassifier', 0.7409574468085106), ('XGBClassifier', 0.7393617021276596), ('LogisticRegression', 0.7377659574468085), ('DecisionTreeClassifier', 0.7372340425531915), ('AdaBoostClassifier', 0.7340425531914894), ('ExtraTreesClassifier', 0.7287234042553191), ('SVC', 0.7170212765957447), ('KNeighborsClassifier', 0.7069148936170213)]


sorted_total_auc:
 [('RandomForestClassifier', 0.8160385129738137), ('LogisticRegression', 0.8139797004723226), ('GradientBoostingClassifier', 0.8138035879239057), ('XGBClassifier', 0.8126412451043552), ('MLPClassifier', 0.8121299506089517), ('AdaBoostClassifier', 0.801191657037287), ('ExtraTreesClassifier', 0.7973012740322615), ('DecisionTreeClassifier', 0.7971541348385844), ('KNeighborsClassifier', 0.7938971889028642), ('SVC', 0.7798138660794733)]
```

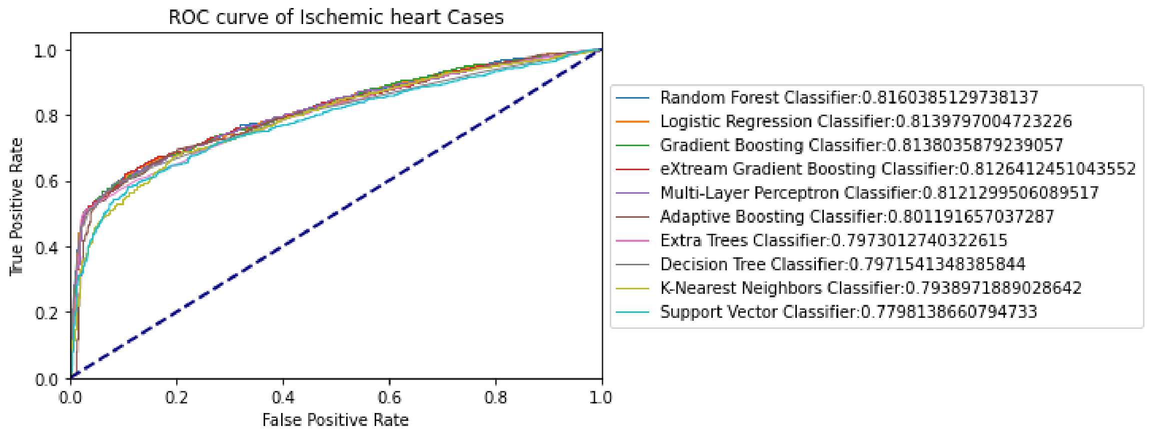

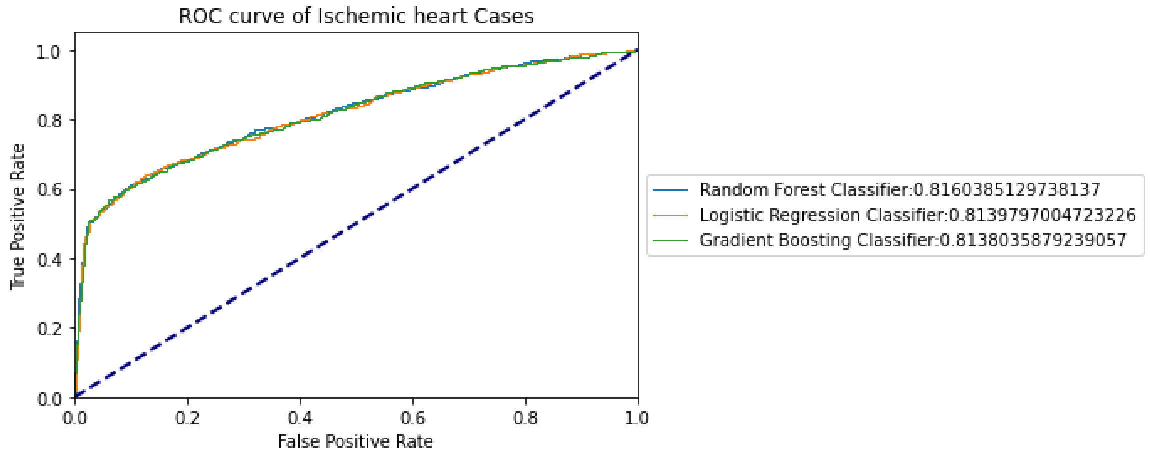

```
random state :   12366
9398
result_train_filename: data/train_test_data_ischemic heart_2013_20210519_114149.cs
v
total count : 9398, df_train count : 7518, df_test count : 1880
(7518, 38)
(1880, 38)
('Logistic Regression', LogisticRegression())
[2021-05-19 11:41:49.623205] Start parameter search for model 'Logistic Regressio
n'
[2021-05-19 11:41:49.959105] Finish parameter search for model 'Logistic Regressio
n' (time: 0 seconds)

=== train : best params ===
{'C': 0.1, 'penalty': 'l2', 'solver': 'lbfgs'}
=== train : best score : roc_auc ===
0.8043091418276458



('Random Forest', RandomForestClassifier())
[2021-05-19 11:41:49.959105] Start parameter search for model 'Random Forest'
[2021-05-19 11:41:54.134899] Finish parameter search for model 'Random Forest' (ti
me: 4 seconds)

=== train : best params ===
{'criterion': 'gini', 'max_depth': 8, 'max_features': 'auto', 'n_estimators': 50}
=== train : best score : roc_auc ===
0.8107485829077042



('Extreme Gradient Boosting)', XGBClassifier(base_score=None, booster=None, colsam
ple_bylevel=None,
              colsample_bynode=None, colsample_bytree=None, gamma=None,
              gpu_id=None, importance_type='gain', interaction_constraints=None,
              learning_rate=None, max_delta_step=None, max_depth=None,
              min_child_weight=None, missing=nan, monotone_constraints=None,
              n_estimators=100, n_jobs=None, num_parallel_tree=None,
              random_state=None, reg_alpha=None, reg_lambda=None,
              scale_pos_weight=None, subsample=None, tree_method=None,
              validate_parameters=None, verbosity=None))
[2021-05-19 11:41:54.134899] Start parameter search for model 'Extreme Gradient Bo
osting)'
[2021-05-19 11:42:20.202559] Finish parameter search for model 'Extreme Gradient B
oosting)' (time: 26 seconds)

=== train : best params ===
{'learning_rate': 0.1, 'max_depth': 5, 'n_estimators': 50, 'subsample': 0.7}
=== train : best score : roc_auc ===
0.8108100442643819



('k-Nearest Neighbours)', KNeighborsClassifier())
[2021-05-19 11:42:20.202559] Start parameter search for model 'k-Nearest Neighbour
s)'
[2021-05-19 11:42:28.093592] Finish parameter search for model 'k-Nearest Neighbou
rs)' (time: 7 seconds)

=== train : best params ===
{'n_neighbors': 60, 'weights': 'distance'}
=== train : best score : roc_auc ===
0.7876972847842572
```

```
('Decision Tree', DecisionTreeClassifier())
[2021-05-19 11:42:28.093592] Start parameter search for model 'Decision Tree'
[2021-05-19 11:42:28.650786] Finish parameter search for model 'Decision Tree' (ti
me: 0 seconds)

=== train : best params ===
{'criterion': 'entropy', 'max_depth': 4}
=== train : best score : roc_auc ===
0.7910542347063911


('Extra Tree', ExtraTreesClassifier())
[2021-05-19 11:42:28.650786] Start parameter search for model 'Extra Tree'
[2021-05-19 11:42:31.551592] Finish parameter search for model 'Extra Tree' (time:
2 seconds)

=== train : best params ===
{'max_features': 'auto', 'n_estimators': 100}
=== train : best score : roc_auc ===
0.8013083580076501


('Gradient Boosting', GradientBoostingClassifier())
[2021-05-19 11:42:31.552589] Start parameter search for model 'Gradient Boosting'
[2021-05-19 11:42:46.196990] Finish parameter search for model 'Gradient Boosting'
(time: 14 seconds)

=== train : best params ===
{'learning_rate': 0.075, 'max_depth': 5, 'max_features': 2, 'n_estimators': 100}
=== train : best score : roc_auc ===
0.8102308078374505


('Adaptive Boosting', AdaBoostClassifier())
[2021-05-19 11:42:46.196990] Start parameter search for model 'Adaptive Boosting'
[2021-05-19 11:42:49.747145] Finish parameter search for model 'Adaptive Boosting'
(time: 3 seconds)

=== train : best params ===
{'learning_rate': 0.2, 'n_estimators': 100}
=== train : best score : roc_auc ===
0.8042088427027598


('Suppport Vector Classfier', SVC(probability=True))
[2021-05-19 11:42:49.748143] Start parameter search for model 'Suppport Vector Cla
ssfier'
[2021-05-19 11:46:37.789402] Finish parameter search for model 'Suppport Vector Cl
assfier' (time: 228 seconds)

=== train : best params ===
{'C': 1, 'gamma': 0.1, 'kernel': 'rbf'}
=== train : best score : roc_auc ===
0.7816477257398535


('Neural Network', MLPClassifier())
[2021-05-19 11:46:37.790400] Start parameter search for model 'Neural Network'
[2021-05-19 11:49:14.588899] Finish parameter search for model 'Neural Network' (t
```

```
ime: 156 seconds)

=== train : best params ===
{'activation': 'logistic', 'alpha': 0.05, 'hidden_layer_sizes': (50,), 'learning_r
ate': 'constant'}
=== train : best score : roc_auc ===
0.8094300898701073



Confusion Matrix:
[[815  66]
 [426 573]]

Classification Report:
              precision    recall  f1-score   support

         0.0       0.66      0.93      0.77       881
         1.0       0.90      0.57      0.70       999

    accuracy                           0.74      1880
   macro avg       0.78      0.75      0.73      1880
weighted avg       0.78      0.74      0.73      1880
```

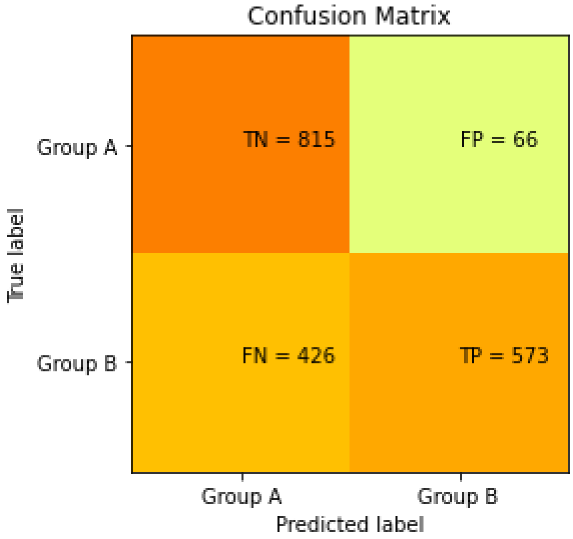


```
AUC Score : 0.7493293520535291

Accuracy Of the Model(LogisticRegression): 0.7382978723404255
```

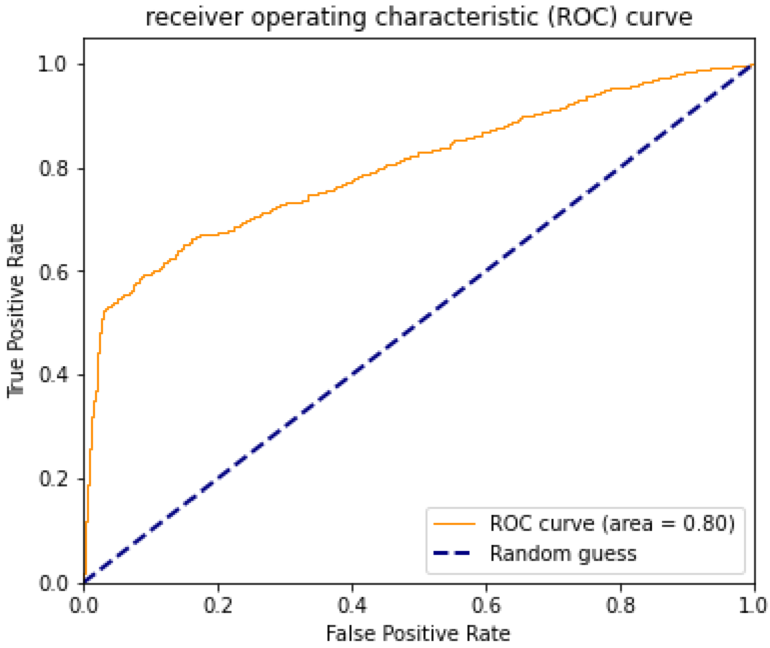


```
ROC_AUC Score of the model(LogisticRegression): 0.8012655106866231


Confusion Matrix:
[[797  84]
 [401 598]]

Classification Report:
              precision    recall  f1-score   support

         0.0       0.67      0.90      0.77       881
         1.0       0.88      0.60      0.71       999

    accuracy                           0.74      1880
   macro avg       0.77      0.75      0.74      1880
weighted avg       0.78      0.74      0.74      1880
```

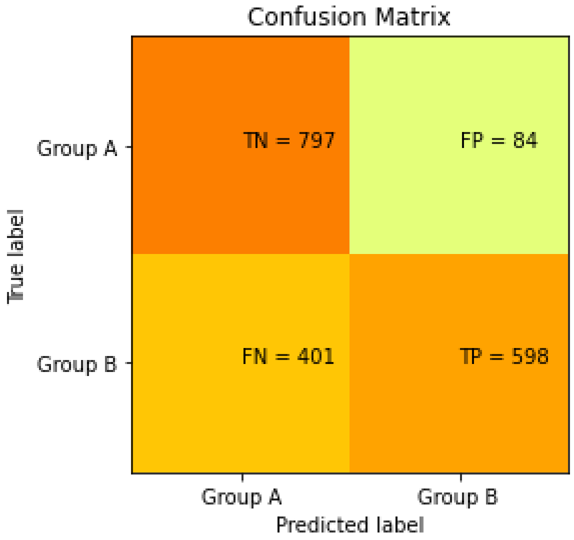


```
AUC Score : 0.7516262005478804

Accuracy Of the Model(RandomForestClassifier): 0.7420212765957447
```

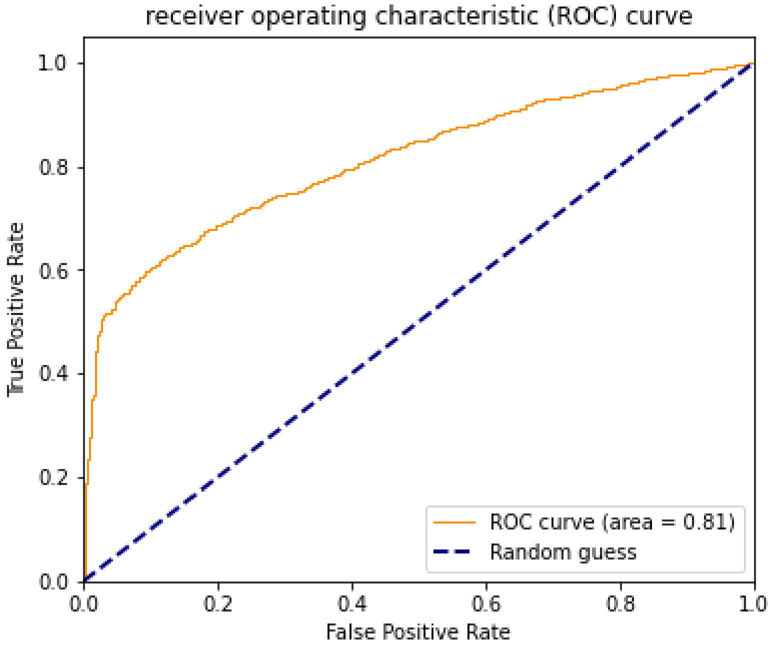

```
ROC_AUC Score of the model(RandomForestClassifier): 0.8133752367577567


Confusion Matrix:
[[793  88]
 [401 598]]

Classification Report:
              precision    recall  f1-score   support

         0.0       0.66      0.90      0.76       881
         1.0       0.87      0.60      0.71       999

    accuracy                           0.74      1880
   macro avg       0.77      0.75      0.74      1880
weighted avg       0.77      0.74      0.74      1880
```

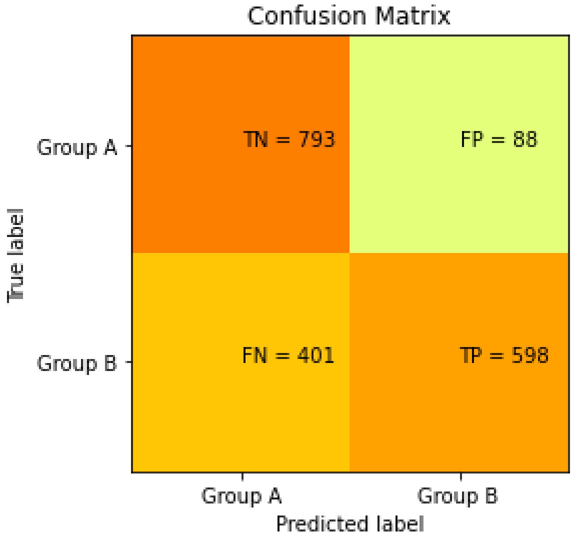


```
AUC Score : 0.7493560529882891

Accuracy Of the Model(XGBClassifier): 0.7398936170212767
```

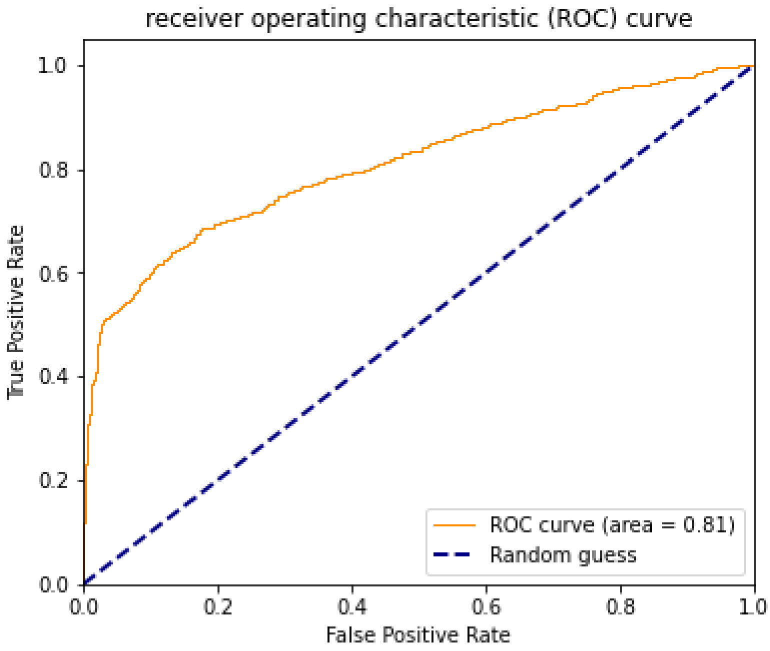

```
ROC_AUC Score of the model(XGBClassifier): 0.8106903725518935


Confusion Matrix:
[[781 100]
 [463 536]]

Classification Report:
              precision    recall  f1-score   support

         0.0       0.63      0.89      0.74       881
         1.0       0.84      0.54      0.66       999

    accuracy                           0.70      1880
   macro avg       0.74      0.71      0.70      1880
weighted avg       0.74      0.70      0.69      1880
```

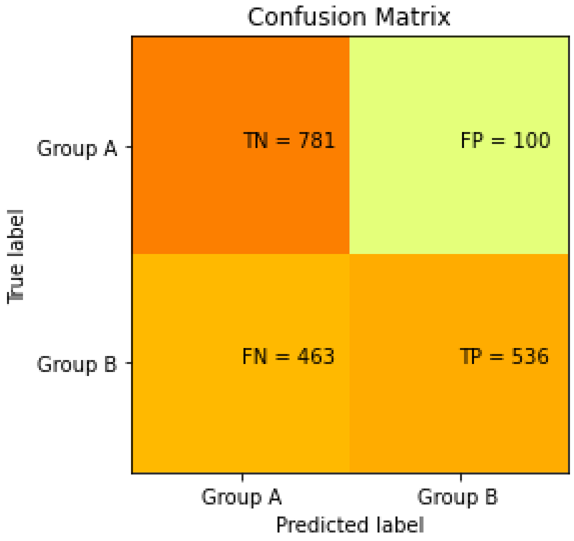


```
AUC Score : 0.7115145792784838

Accuracy Of the Model(KNeighborsClassifier): 0.700531914893617
```

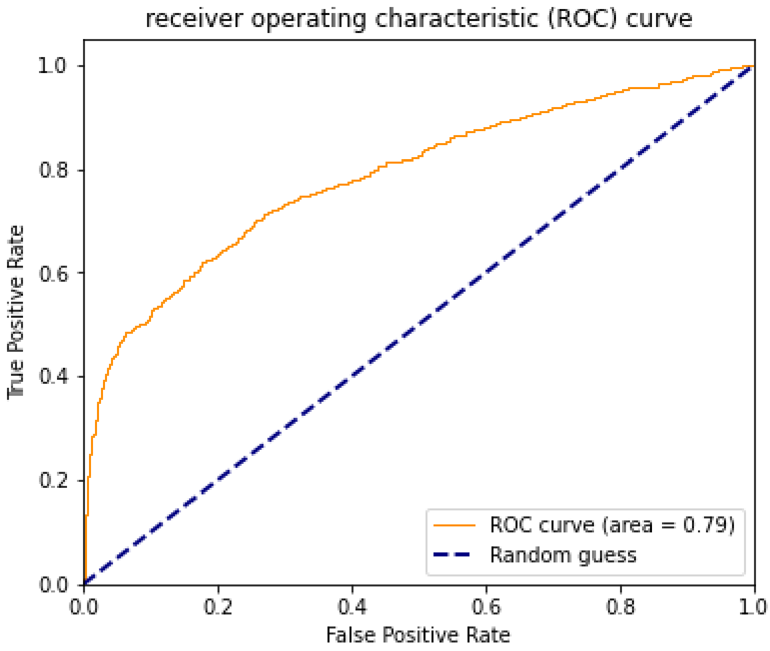

```
ROC_AUC Score of the model(KNeighborsClassifier): 0.787909362256695


Confusion Matrix:
[[804  77]
 [427 572]]

Classification Report:
              precision    recall  f1-score   support

         0.0       0.65      0.91      0.76       881
         1.0       0.88      0.57      0.69       999

    accuracy                           0.73      1880
   macro avg       0.77      0.74      0.73      1880
weighted avg       0.77      0.73      0.73      1880
```

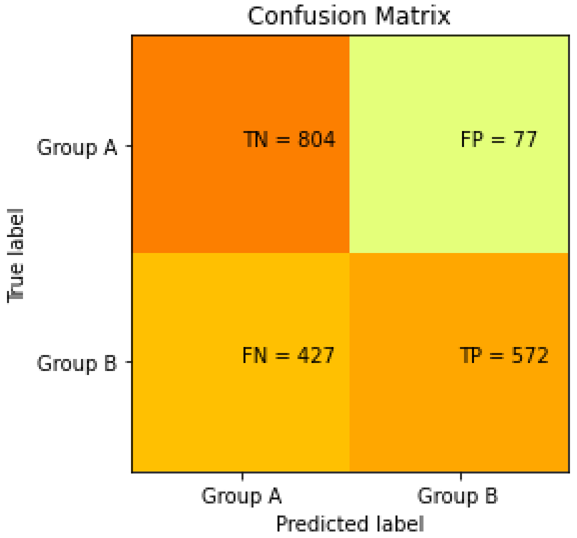


```
AUC Score : 0.7425859457641523

Accuracy Of the Model(DecisionTreeClassifier): 0.7319148936170212
```

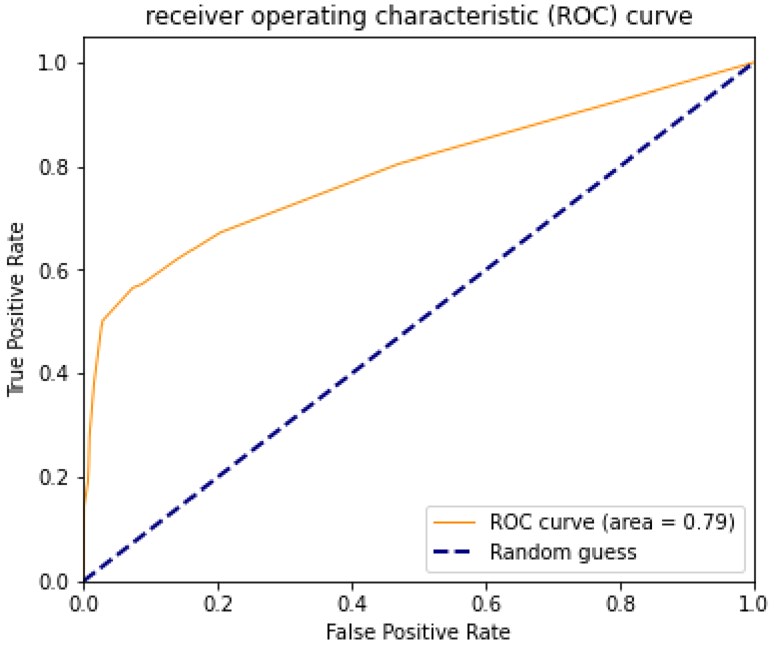

```
ROC_AUC Score of the model(DecisionTreeClassifier): 0.7888529846532117


Confusion Matrix:
[[785  96]
 [410 589]]

Classification Report:
              precision    recall  f1-score   support

         0.0       0.66      0.89      0.76       881
         1.0       0.86      0.59      0.70       999

    accuracy                           0.73      1880
   macro avg       0.76      0.74      0.73      1880
weighted avg       0.76      0.73      0.73      1880
```

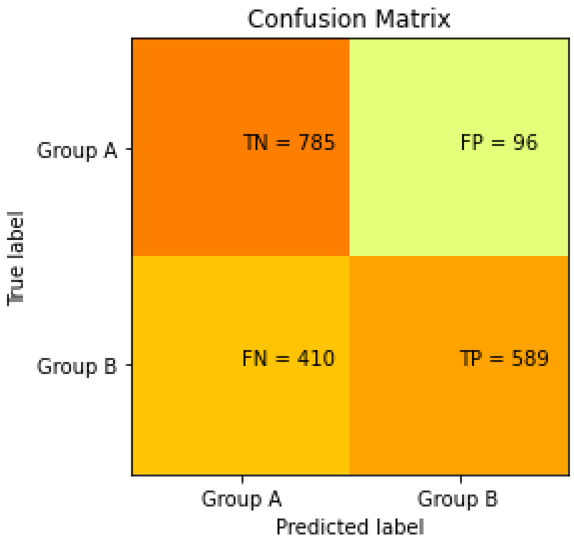


```
AUC Score : 0.7403112533646019

Accuracy Of the Model(ExtraTreesClassifier): 0.7308510638297873
```

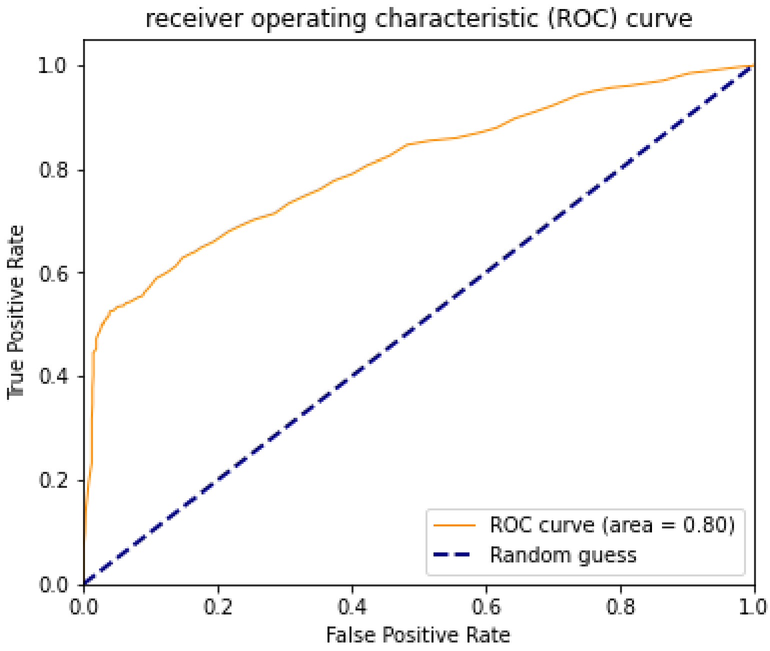

```
ROC_AUC Score of the model(ExtraTreesClassifier): 0.8048485488894117


Confusion Matrix:
[[769 112]
 [377 622]]

Classification Report:
              precision    recall  f1-score   support

         0.0       0.67      0.87      0.76       881
         1.0       0.85      0.62      0.72       999

    accuracy                           0.74      1880
   macro avg       0.76      0.75      0.74      1880
weighted avg       0.76      0.74      0.74      1880
```

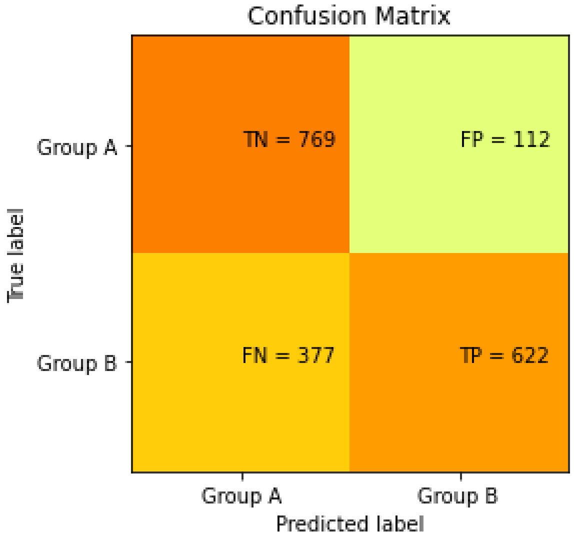


```
AUC Score : 0.7477471796427528

Accuracy Of the Model(GradientBoostingClassifier): 0.7398936170212767
```

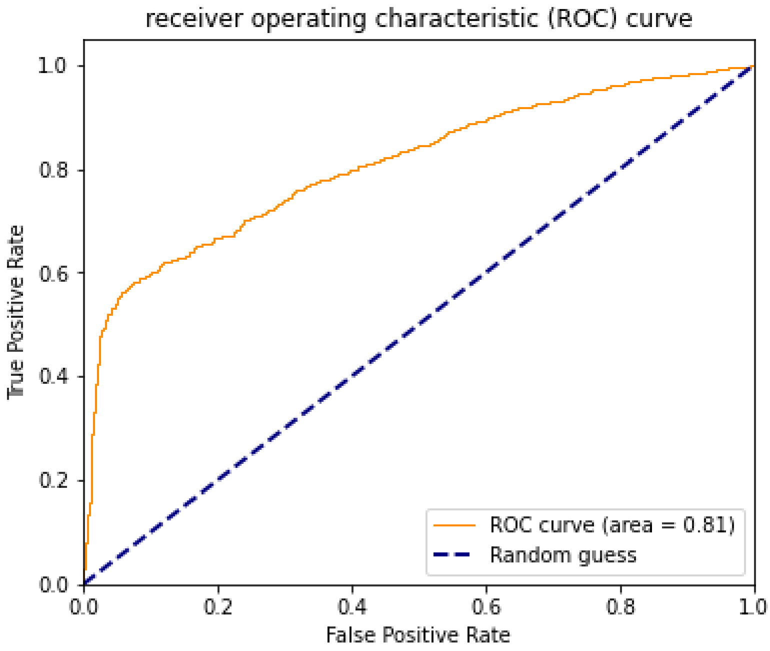

ROC_AUC Score of the model(GradientBoostingClassifier): 0.8107778607211071

```
Confusion Matrix:
[[817  64]
 [426 573]]

Classification Report:
              precision    recall  f1-score   support

         0.0       0.66      0.93      0.77       881
         1.0       0.90      0.57      0.70       999

    accuracy                           0.74      1880
   macro avg       0.78      0.75      0.73      1880
weighted avg       0.79      0.74      0.73      1880
```

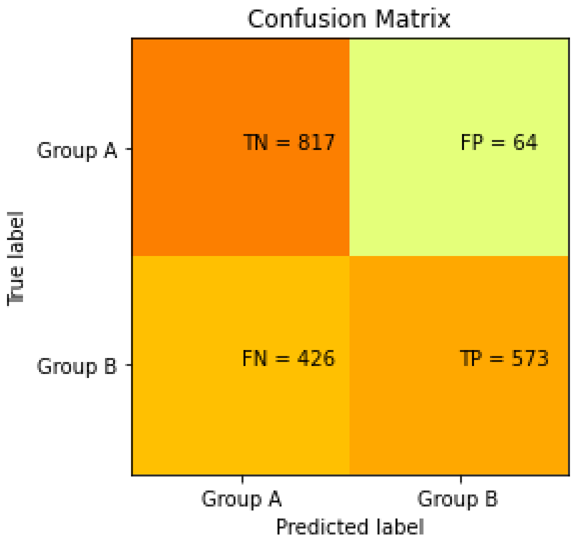

AUC Score : 0.7504644258333248

Accuracy Of the Model(AdaBoostClassifier): 0.7393617021276596

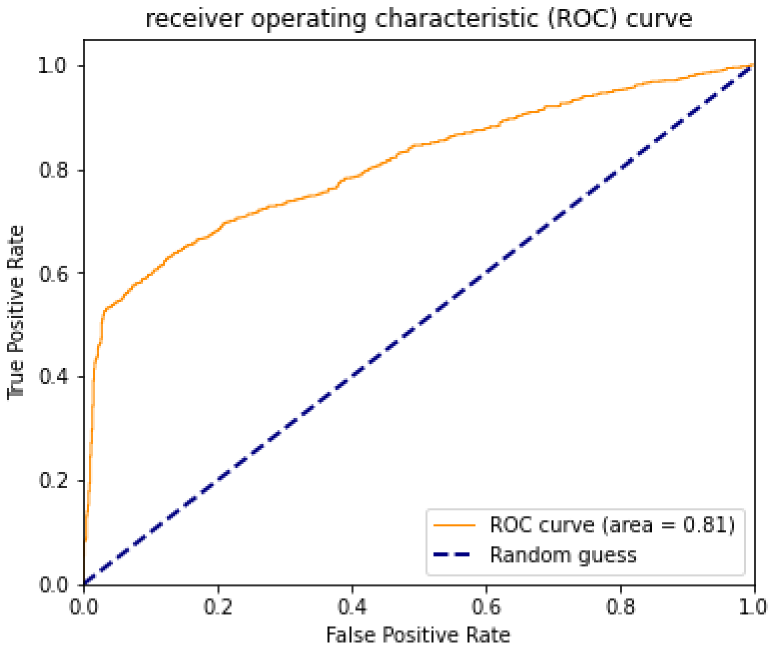

```
ROC_AUC Score of the model(AdaBoostClassifier): 0.8066233088934565


Confusion Matrix:
[[687 194]
 [349 650]]

Classification Report:
              precision    recall  f1-score   support

         0.0       0.66      0.78      0.72       881
         1.0       0.77      0.65      0.71       999

    accuracy                           0.71      1880
   macro avg       0.72      0.72      0.71      1880
weighted avg       0.72      0.71      0.71      1880
```

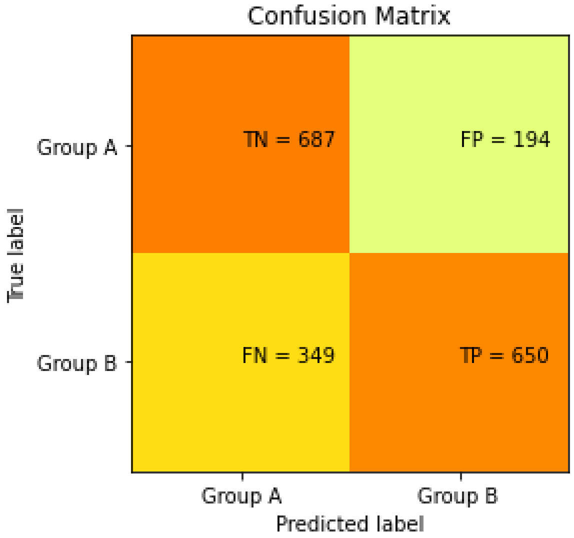


```
AUC Score : 0.7152231686851437

Accuracy Of the Model(SVC): 0.7111702127659575
```

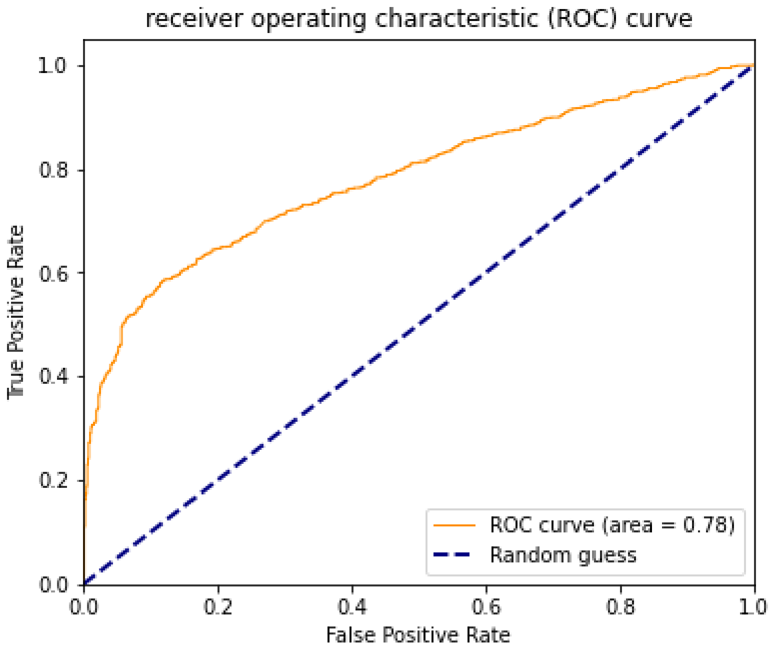

```
ROC_AUC Score of the model(SVC): 0.7818363198612914


Confusion Matrix:
[[801  80]
 [425 574]]

Classification Report:
              precision    recall  f1-score   support

         0.0       0.65      0.91      0.76       881
         1.0       0.88      0.57      0.69       999

    accuracy                           0.73      1880
   macro avg       0.77      0.74      0.73      1880
weighted avg       0.77      0.73      0.73      1880
```

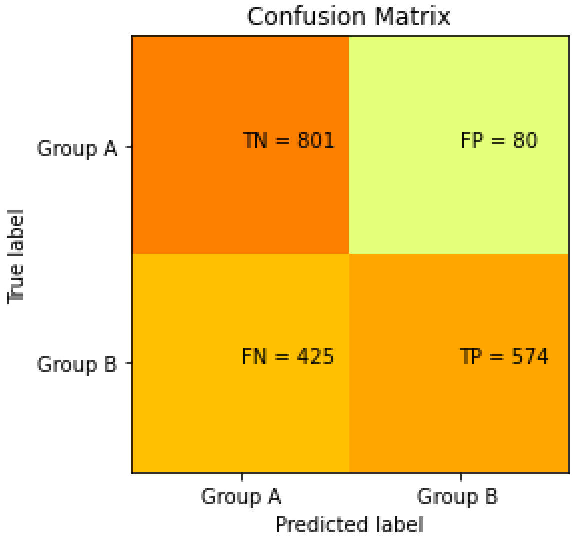


```
AUC Score : 0.7418843360954599

Accuracy Of the Model(MLPClassifier): 0.7313829787234043
```

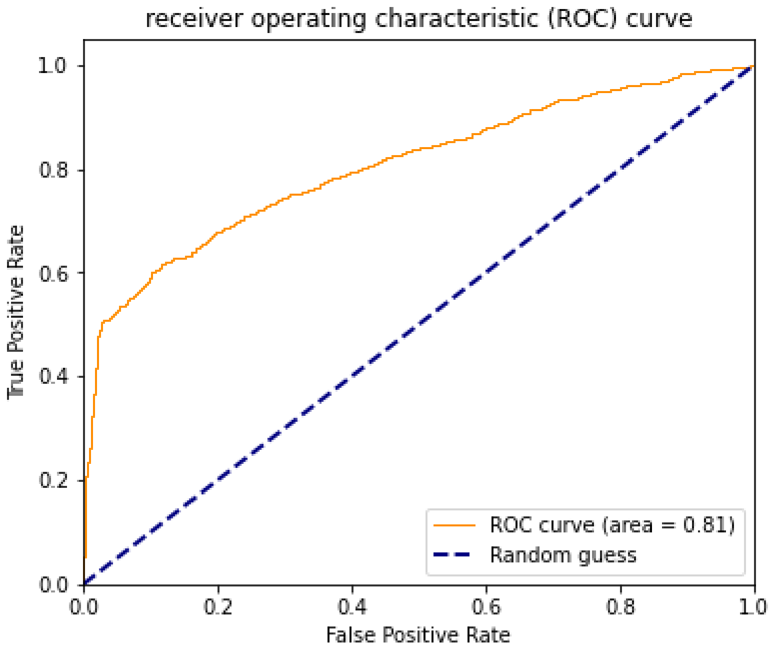

```
ROC_AUC Score of the model(MLPClassifier): 0.8075862468597997

[('RandomForestClassifier', 0.7420212765957447), ('XGBClassifier', 0.7398936170212767), ('GradientBoostingClassifier', 0.7398936170212767), ('AdaBoostClassifier', 0.7393617021276596), ('LogisticRegression', 0.7382978723404255), ('DecisionTreeClassifier', 0.7319148936170212), ('MLPClassifier', 0.7313829787234043), ('ExtraTreesClassifier', 0.7308510638297873), ('SVC', 0.7111702127659575), ('KNeighborsClassifier', 0.700531914893617)]


sorted_total_auc:
 [('RandomForestClassifier', 0.8133752367577567), ('GradientBoostingClassifier', 0.8107778607211071), ('XGBClassifier', 0.8106903725518935), ('MLPClassifier', 0.8075862468597997), ('AdaBoostClassifier', 0.8066233088934565), ('ExtraTreesClassifier', 0.8048485488894117), ('LogisticRegression', 0.8012655106866231), ('DecisionTreeClassifier', 0.7888529846532117), ('KNeighborsClassifier', 0.787909362256695), ('SVC', 0.7818363198612914)]
```

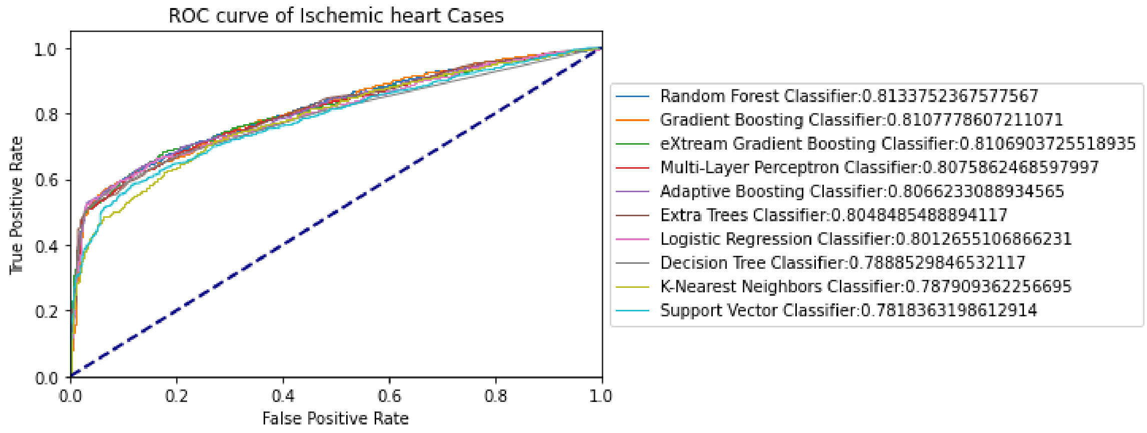

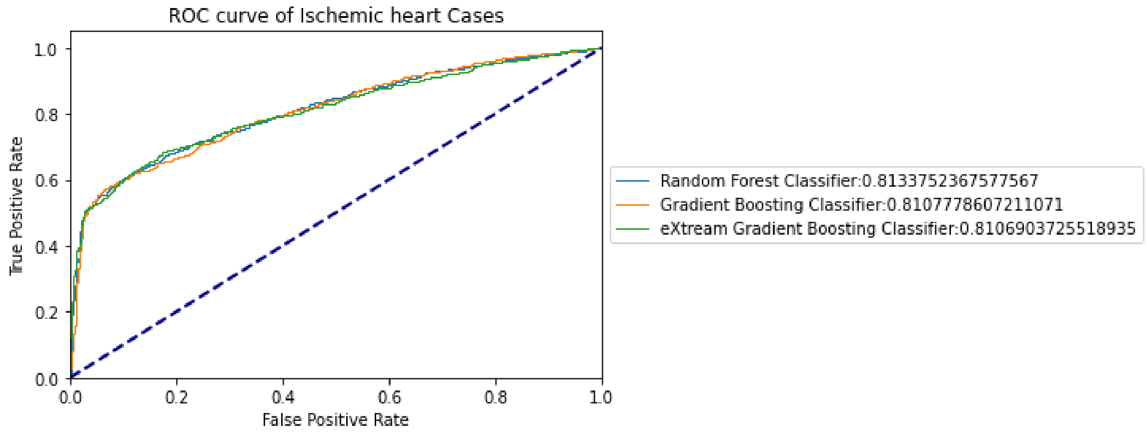

```
random state :   12373
9398
result_train_filename: data/train_test_data_ischemic heart_2013_20210519_114922.cs
v
total count : 9398, df_train count : 7518, df_test count : 1880
(7518, 38)
(1880, 38)
('Logistic Regression', LogisticRegression())
[2021-05-19 11:49:22.373813] Start parameter search for model 'Logistic Regressio
n'
[2021-05-19 11:49:22.770243] Finish parameter search for model 'Logistic Regressio
n' (time: 0 seconds)

=== train : best params ===
{'C': 0.1, 'penalty': 'l2', 'solver': 'lbfgs'}
=== train : best score : roc_auc ===
0.8077884210512253



('Random Forest', RandomForestClassifier())
[2021-05-19 11:49:22.772237] Start parameter search for model 'Random Forest'
[2021-05-19 11:49:27.567970] Finish parameter search for model 'Random Forest' (ti
me: 4 seconds)

=== train : best params ===
{'criterion': 'gini', 'max_depth': 10, 'max_features': 'auto', 'n_estimators': 10
0}
=== train : best score : roc_auc ===
0.811917475780289



('Extreme Gradient Boosting)', XGBClassifier(base_score=None, booster=None, colsam
ple_bylevel=None,
              colsample_bynode=None, colsample_bytree=None, gamma=None,
              gpu_id=None, importance_type='gain', interaction_constraints=None,
              learning_rate=None, max_delta_step=None, max_depth=None,
              min_child_weight=None, missing=nan, monotone_constraints=None,
              n_estimators=100, n_jobs=None, num_parallel_tree=None,
              random_state=None, reg_alpha=None, reg_lambda=None,
              scale_pos_weight=None, subsample=None, tree_method=None,
              validate_parameters=None, verbosity=None))
[2021-05-19 11:49:27.568969] Start parameter search for model 'Extreme Gradient Bo
osting)'
[2021-05-19 11:49:54.421299] Finish parameter search for model 'Extreme Gradient B
oosting)' (time: 26 seconds)

=== train : best params ===
{'learning_rate': 0.1, 'max_depth': 5, 'n_estimators': 50, 'subsample': 0.7}
=== train : best score : roc_auc ===
0.81187510835531



('k-Nearest Neighbours)', KNeighborsClassifier())
[2021-05-19 11:49:54.422296] Start parameter search for model 'k-Nearest Neighbour
s)'
[2021-05-19 11:50:02.316202] Finish parameter search for model 'k-Nearest Neighbou
rs)' (time: 7 seconds)

=== train : best params ===
{'n_neighbors': 60, 'weights': 'distance'}
=== train : best score : roc_auc ===
```

```
0.7852852412652668


('Decision Tree', DecisionTreeClassifier())
[2021-05-19 11:50:02.316202] Start parameter search for model 'Decision Tree'
[2021-05-19 11:50:02.894423] Finish parameter search for model 'Decision Tree' (ti
me: 0 seconds)

=== train : best params ===
{'criterion': 'gini', 'max_depth': 5}
=== train : best score : roc_auc ===
0.7963397970864502


('Extra Tree', ExtraTreesClassifier())
[2021-05-19 11:50:02.894423] Start parameter search for model 'Extra Tree'
[2021-05-19 11:50:05.942690] Finish parameter search for model 'Extra Tree' (time:
3 seconds)

=== train : best params ===
{'max_features': 'sqrt', 'n_estimators': 100}
=== train : best score : roc_auc ===
0.804065535710632


('Gradient Boosting', GradientBoostingClassifier())
[2021-05-19 11:50:05.943692] Start parameter search for model 'Gradient Boosting'
[2021-05-19 11:50:20.731695] Finish parameter search for model 'Gradient Boosting'
(time: 14 seconds)

=== train : best params ===
{'learning_rate': 0.075, 'max_depth': 5, 'max_features': 6, 'n_estimators': 50}
=== train : best score : roc_auc ===
0.8122702283346402


('Adaptive Boosting', AdaBoostClassifier())
[2021-05-19 11:50:20.731695] Start parameter search for model 'Adaptive Boosting'
[2021-05-19 11:50:24.352085] Finish parameter search for model 'Adaptive Boosting'
(time: 3 seconds)

=== train : best params ===
{'learning_rate': 0.2, 'n_estimators': 100}
=== train : best score : roc_auc ===
0.8055639430747379


('Suppport Vector Classfier', SVC(probability=True))
[2021-05-19 11:50:24.353061] Start parameter search for model 'Suppport Vector Cla
ssfier'
[2021-05-19 11:55:04.088029] Finish parameter search for model 'Suppport Vector Cl
assfier' (time: 279 seconds)

=== train : best params ===
{'C': 1, 'gamma': 0.1, 'kernel': 'rbf'}
=== train : best score : roc_auc ===
0.7859387084131231


('Neural Network', MLPClassifier())
[2021-05-19 11:55:04.088029] Start parameter search for model 'Neural Network'
```

```
[2021-05-19 11:57:42.221196] Finish parameter search for model 'Neural Network' (t
ime: 158 seconds)

=== train : best params ===
{'activation': 'logistic', 'alpha': 0.05, 'hidden_layer_sizes': (50,), 'learning_r
ate': 'adaptive'}
=== train : best score : roc_auc ===
0.8132964800209821



Confusion Matrix:
[[815  66]
 [429 570]]

Classification Report:
              precision    recall  f1-score   support

         0.0       0.66      0.93      0.77       881
         1.0       0.90      0.57      0.70       999

    accuracy                           0.74      1880
   macro avg       0.78      0.75      0.73      1880
weighted avg       0.78      0.74      0.73      1880
```

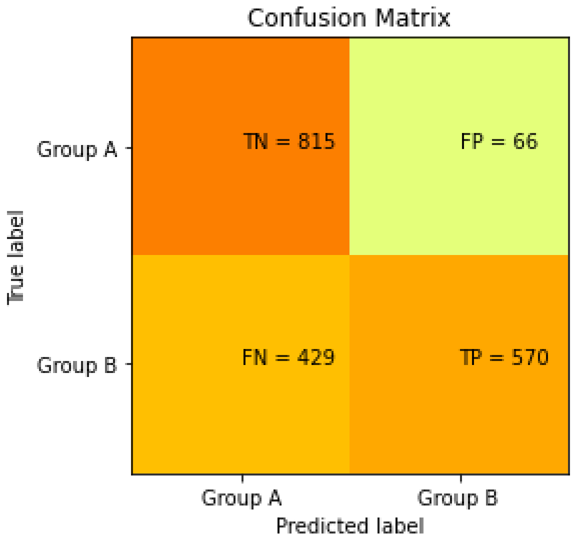


```
AUC Score : 0.7478278505520276

Accuracy Of the Model(LogisticRegression): 0.7367021276595744
```

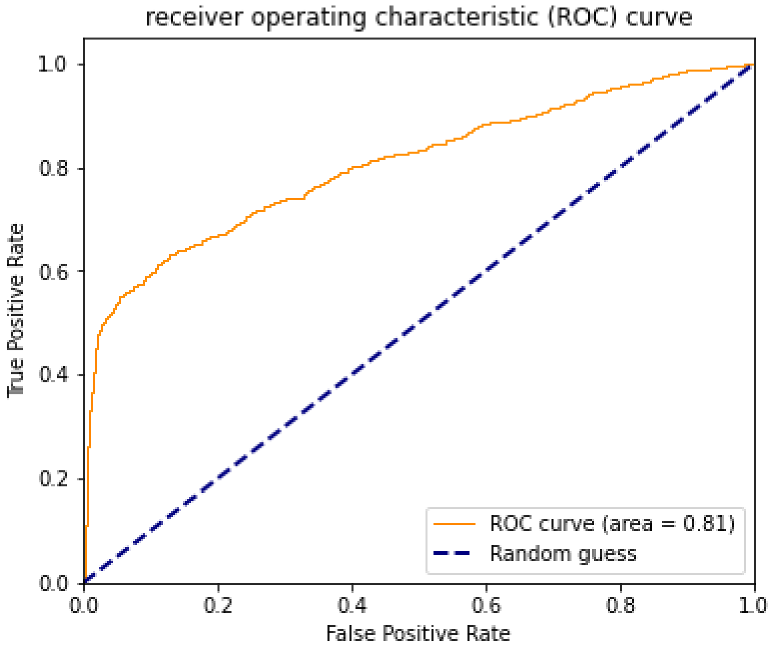


```
ROC_AUC Score of the model(LogisticRegression): 0.8073612772818222


Confusion Matrix:
[[787  94]
 [402 597]]

Classification Report:
              precision    recall  f1-score   support

         0.0       0.66      0.89      0.76       881
         1.0       0.86      0.60      0.71       999

    accuracy                           0.74      1880
   macro avg       0.76      0.75      0.73      1880
weighted avg       0.77      0.74      0.73      1880
```

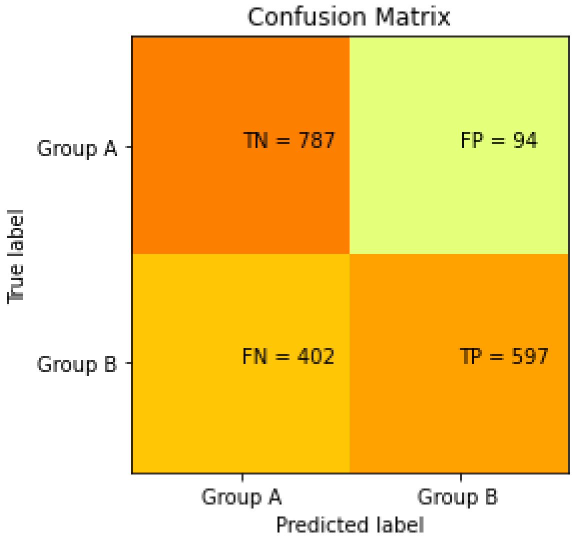


```
AUC Score : 0.7454503311484016

Accuracy Of the Model(RandomForestClassifier): 0.7361702127659574
```

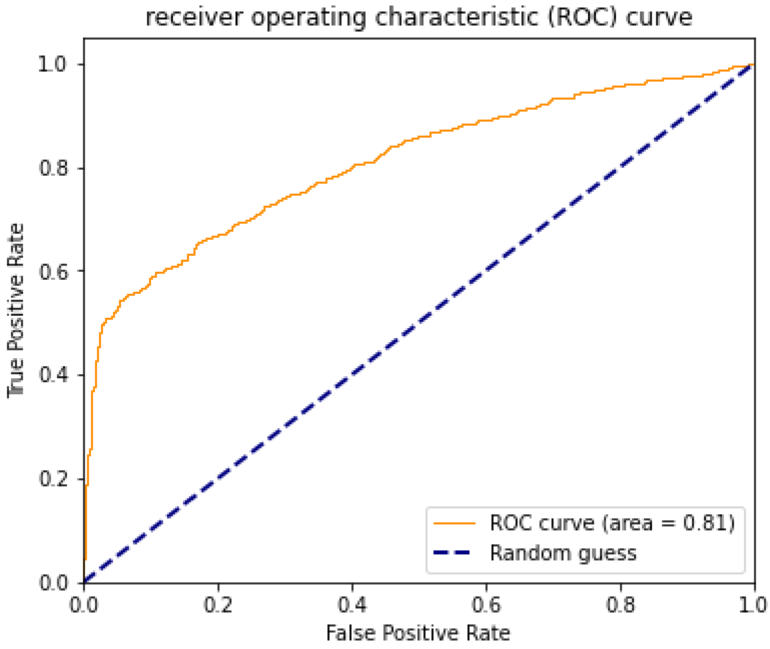

```
ROC_AUC Score of the model(RandomForestClassifier): 0.8103199680952234


Confusion Matrix:
[[785  96]
 [391 608]]

Classification Report:
              precision    recall  f1-score   support

         0.0       0.67      0.89      0.76       881
         1.0       0.86      0.61      0.71       999

    accuracy                           0.74      1880
   macro avg       0.77      0.75      0.74      1880
weighted avg       0.77      0.74      0.74      1880
```

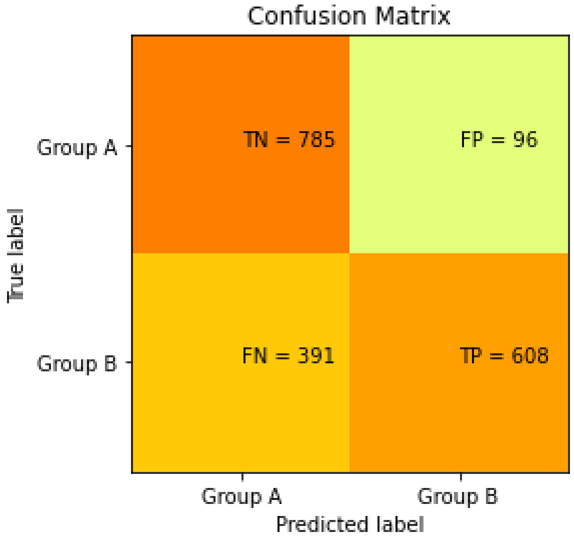


```
AUC Score : 0.7498207628741114

Accuracy Of the Model(XGBClassifier): 0.7409574468085106
```

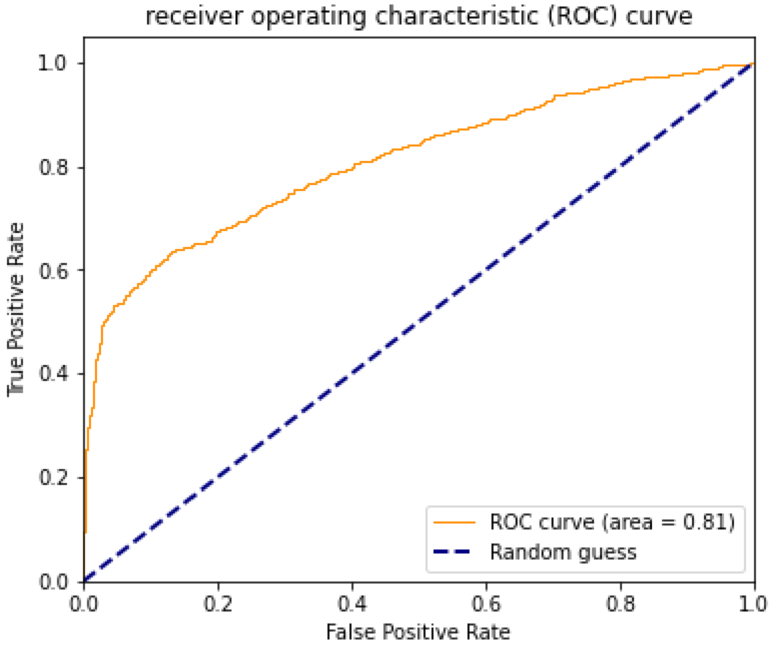

```
ROC_AUC Score of the model(XGBClassifier): 0.8118845292511581


Confusion Matrix:
[[799  82]
 [461 538]]

Classification Report:
              precision    recall  f1-score   support

         0.0       0.63      0.91      0.75       881
         1.0       0.87      0.54      0.66       999

    accuracy                           0.71      1880
   macro avg       0.75      0.72      0.71      1880
weighted avg       0.76      0.71      0.70      1880
```

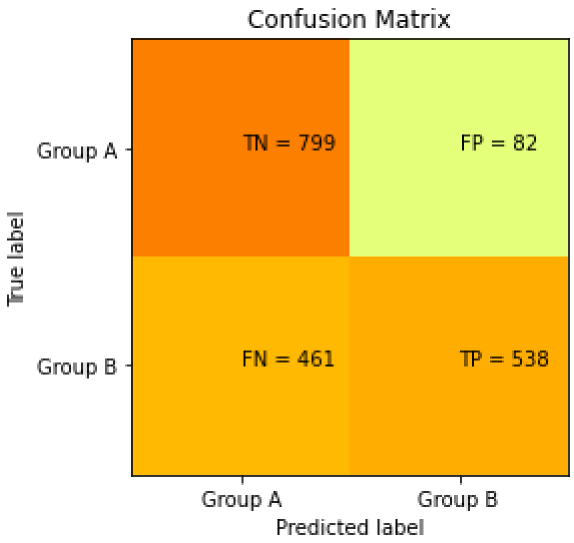


```
AUC Score : 0.7227312244297646

Accuracy Of the Model(KNeighborsClassifier): 0.7111702127659575
```

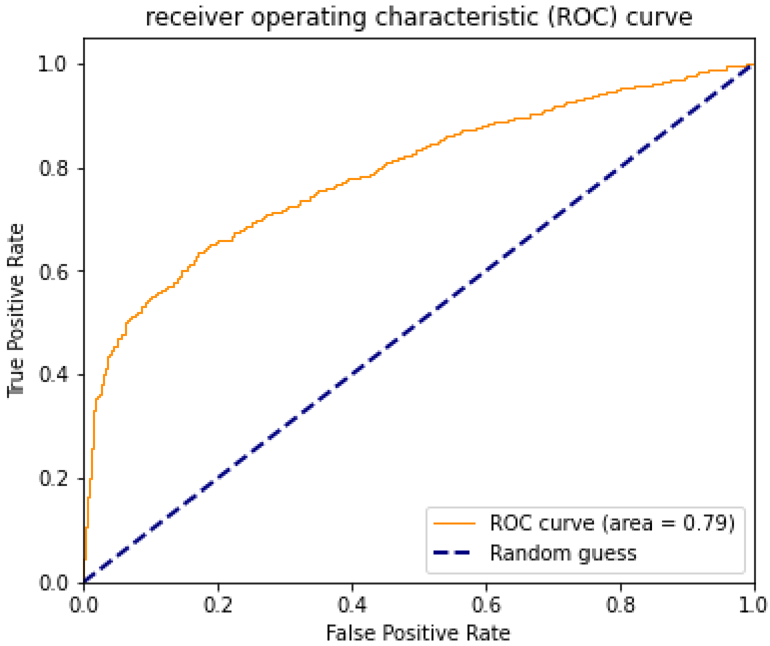

```
ROC_AUC Score of the model(KNeighborsClassifier): 0.7907726114309541


Confusion Matrix:
[[789  92]
 [415 584]]

Classification Report:
              precision    recall  f1-score   support

         0.0       0.66      0.90      0.76       881
         1.0       0.86      0.58      0.70       999

    accuracy                           0.73      1880
   macro avg       0.76      0.74      0.73      1880
weighted avg       0.77      0.73      0.73      1880
```

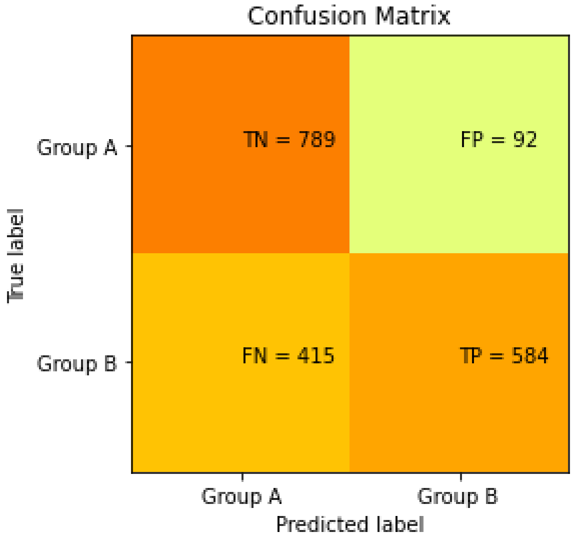


```
AUC Score : 0.7400788984216908

Accuracy Of the Model(DecisionTreeClassifier): 0.7303191489361702
```

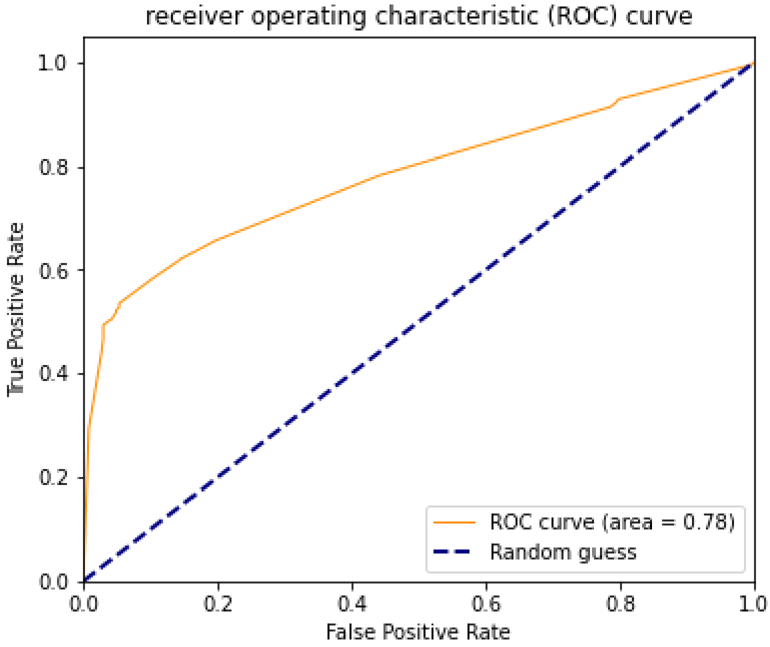

```
ROC_AUC Score of the model(DecisionTreeClassifier): 0.7815420414739371


Confusion Matrix:
[[785  96]
 [416 583]]

Classification Report:
              precision    recall  f1-score   support

         0.0       0.65      0.89      0.75       881
         1.0       0.86      0.58      0.69       999

    accuracy                           0.73      1880
   macro avg       0.76      0.74      0.72      1880
weighted avg       0.76      0.73      0.72      1880
```

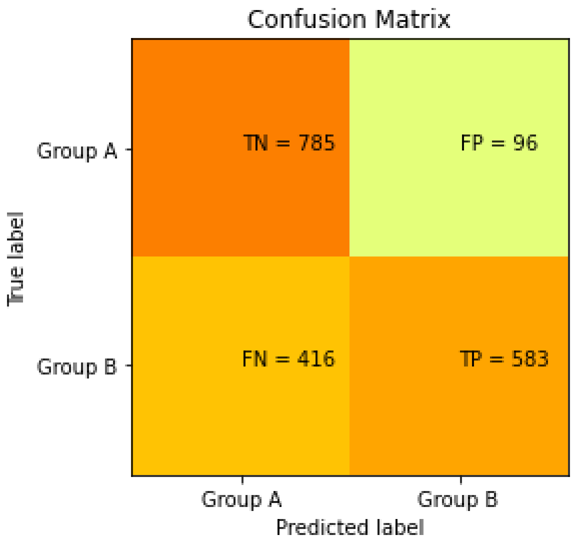


```
AUC Score : 0.7373082503615989

Accuracy Of the Model(ExtraTreesClassifier): 0.7276595744680852
```

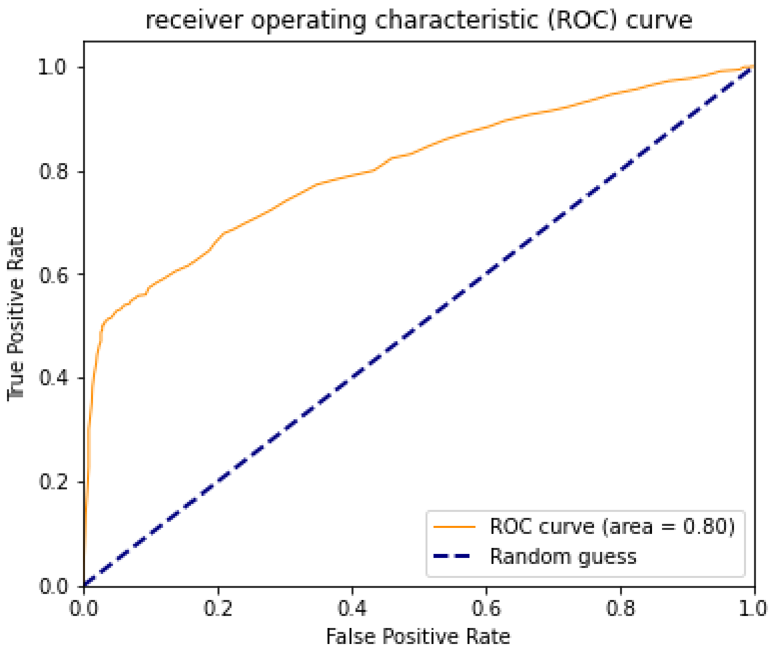

```
ROC_AUC Score of the model(ExtraTreesClassifier): 0.8033067119332726


Confusion Matrix:
[[777 104]
 [402 597]]

Classification Report:
              precision    recall  f1-score   support

         0.0       0.66      0.88      0.75       881
         1.0       0.85      0.60      0.70       999

    accuracy                           0.73      1880
   macro avg       0.76      0.74      0.73      1880
weighted avg       0.76      0.73      0.73      1880
```

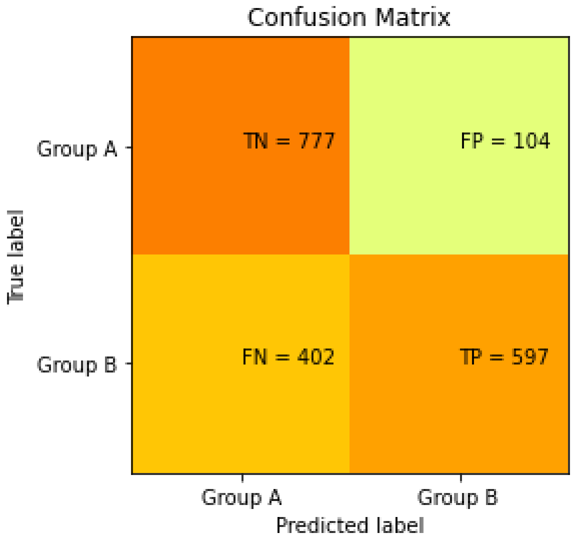


```
AUC Score : 0.7397749622494231

Accuracy Of the Model(GradientBoostingClassifier): 0.7308510638297873
```

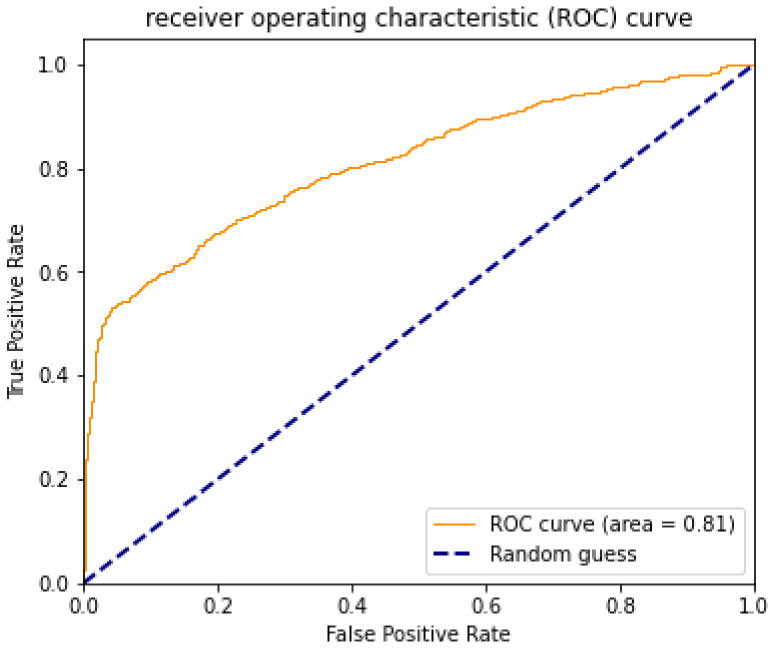

```
ROC_AUC Score of the model(GradientBoostingClassifier): 0.8118765757812296


Confusion Matrix:
[[799  82]
 [430 569]]

Classification Report:
              precision    recall  f1-score   support

         0.0       0.65      0.91      0.76       881
         1.0       0.87      0.57      0.69       999

    accuracy                           0.73      1880
   macro avg       0.76      0.74      0.72      1880
weighted avg       0.77      0.73      0.72      1880
```

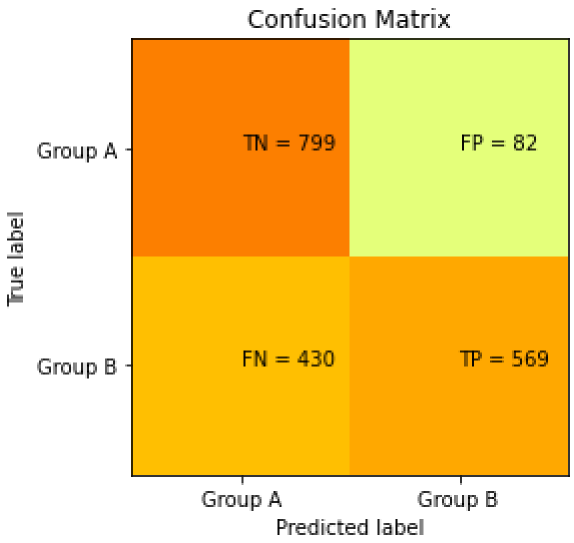


```
AUC Score : 0.7382467598131616

Accuracy Of the Model(AdaBoostClassifier): 0.7276595744680852
```

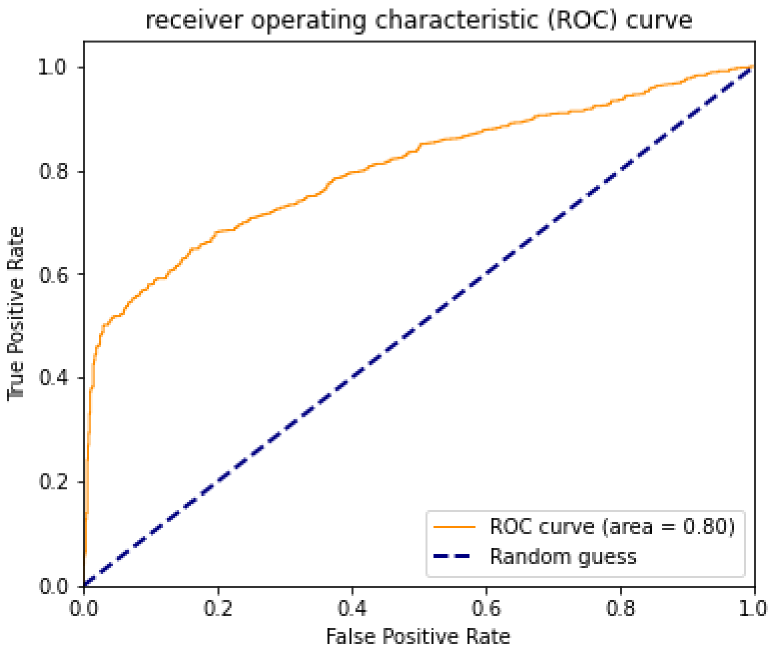

ROC_AUC Score of the model(AdaBoostClassifier): 0.8010041823889724

```
Confusion Matrix:
[[692 189]
 [352 647]]

Classification Report:
              precision    recall  f1-score   support

         0.0       0.66      0.79      0.72       881
         1.0       0.77      0.65      0.71       999

    accuracy                           0.71      1880
   macro avg       0.72      0.72      0.71      1880
weighted avg       0.72      0.71      0.71      1880
```

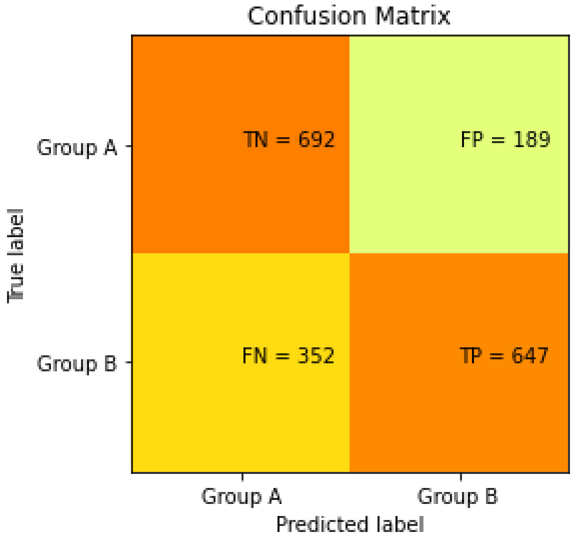

AUC Score : 0.7165593516331314

Accuracy Of the Model(SVC): 0.7122340425531914

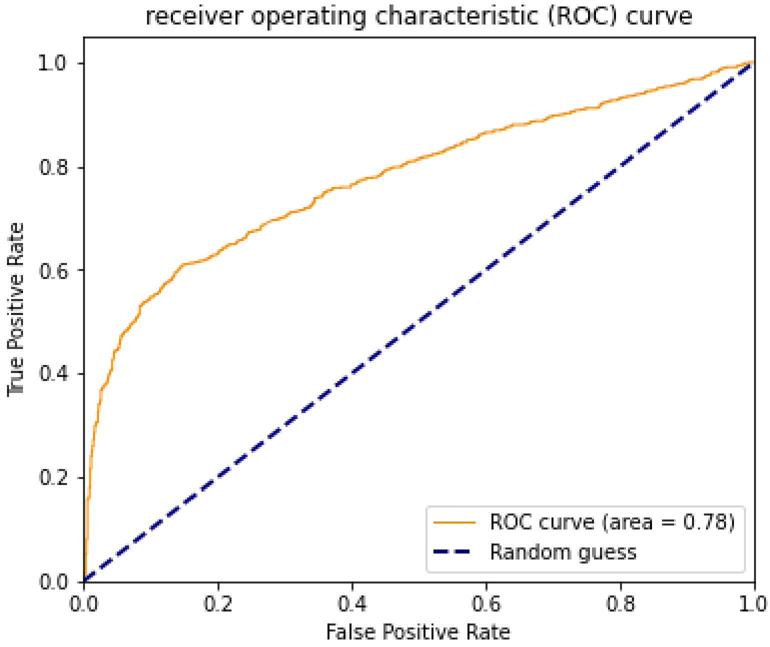

```
ROC_AUC Score of the model(SVC): 0.7754939956983089


Confusion Matrix:
[[789  92]
 [410 589]]

Classification Report:
              precision    recall  f1-score   support

         0.0       0.66      0.90      0.76       881
         1.0       0.86      0.59      0.70       999

    accuracy                           0.73      1880
   macro avg       0.76      0.74      0.73      1880
weighted avg       0.77      0.73      0.73      1880
```

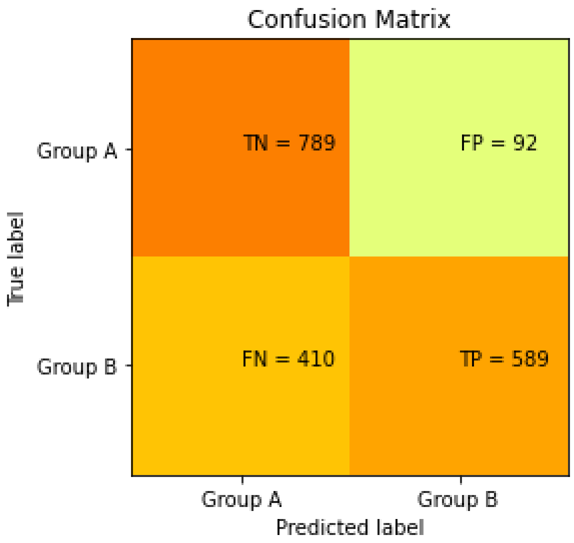


```
AUC Score : 0.7425814009241933

Accuracy Of the Model(MLPClassifier): 0.7329787234042553
```

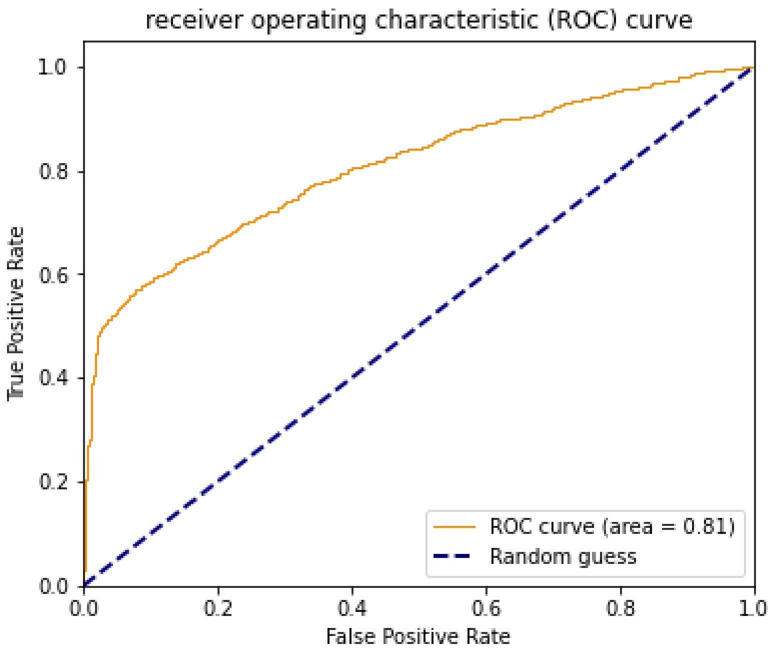

```
ROC_AUC Score of the model(MLPClassifier): 0.8083293281931193

[('XGBClassifier', 0.7409574468085106), ('LogisticRegression', 0.736702127659574
4), ('RandomForestClassifier', 0.7361702127659574), ('MLPClassifier', 0.7329787234
042553), ('GradientBoostingClassifier', 0.7308510638297873), ('DecisionTreeClassif
ier', 0.7303191489361702), ('ExtraTreesClassifier', 0.7276595744680852), ('AdaBoos
tClassifier', 0.7276595744680852), ('SVC', 0.7122340425531914), ('KNeighborsClassi
fier', 0.7111702127659575)]


sorted_total_auc:
 [('XGBClassifier', 0.8118845292511581), ('GradientBoostingClassifier', 0.81187657
57812296), ('RandomForestClassifier', 0.8103199680952234), ('MLPClassifier', 0.808
3293281931193), ('LogisticRegression', 0.8073612772818222), ('ExtraTreesClassifie
r', 0.8033067119332726), ('AdaBoostClassifier', 0.8010041823889724), ('KNeighborsC
lassifier', 0.7907726114309541), ('DecisionTreeClassifier', 0.7815420414739371),
('SVC', 0.7754939956983089)]
```

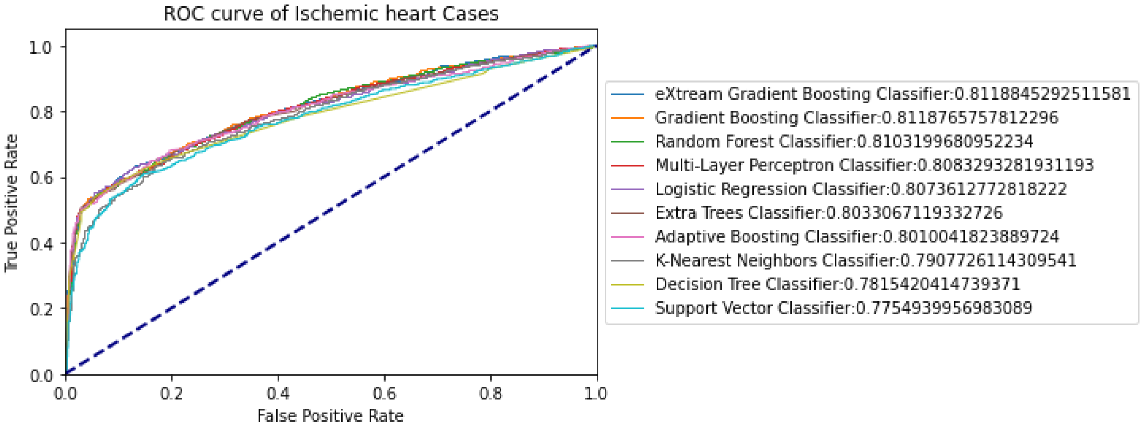

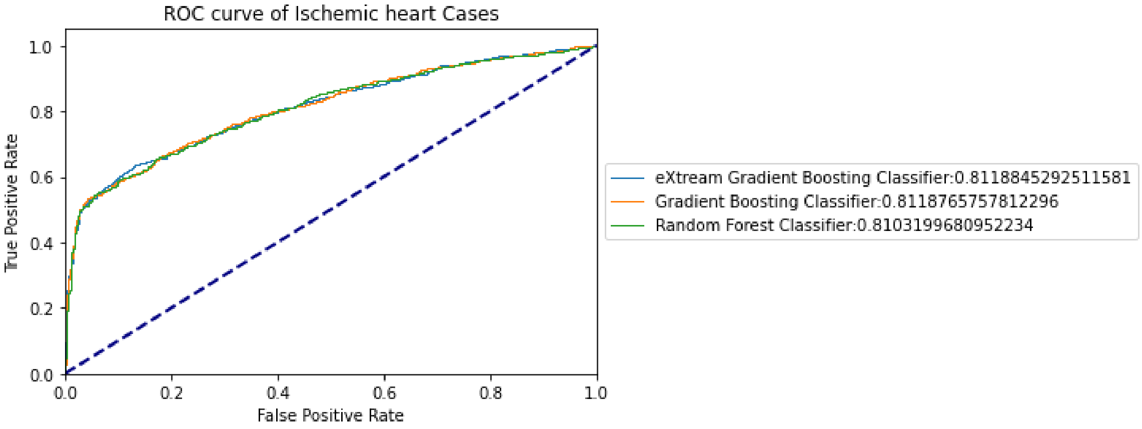

```
random state :   12381
9398
result_train_filename: data/train_test_data_ischemic heart_2013_20210519_115749.cs
v
total count : 9398, df_train count : 7518, df_test count : 1880
(7518, 38)
(1880, 38)
('Logistic Regression', LogisticRegression())
[2021-05-19 11:57:50.026221] Start parameter search for model 'Logistic Regressio
n'
[2021-05-19 11:57:50.370752] Finish parameter search for model 'Logistic Regressio
n' (time: 0 seconds)

=== train : best params ===
{'C': 0.1, 'penalty': 'l2', 'solver': 'lbfgs'}
=== train : best score : roc_auc ===
0.8050376217987035



('Random Forest', RandomForestClassifier())
[2021-05-19 11:57:50.371749] Start parameter search for model 'Random Forest'
[2021-05-19 11:57:54.647304] Finish parameter search for model 'Random Forest' (ti
me: 4 seconds)

=== train : best params ===
{'criterion': 'gini', 'max_depth': 10, 'max_features': 'auto', 'n_estimators': 90}
=== train : best score : roc_auc ===
0.8114728390393611



('Extreme Gradient Boosting)', XGBClassifier(base_score=None, booster=None, colsam
ple_bylevel=None,
              colsample_bynode=None, colsample_bytree=None, gamma=None,
              gpu_id=None, importance_type='gain', interaction_constraints=None,
              learning_rate=None, max_delta_step=None, max_depth=None,
              min_child_weight=None, missing=nan, monotone_constraints=None,
              n_estimators=100, n_jobs=None, num_parallel_tree=None,
              random_state=None, reg_alpha=None, reg_lambda=None,
              scale_pos_weight=None, subsample=None, tree_method=None,
              validate_parameters=None, verbosity=None))
[2021-05-19 11:57:54.647304] Start parameter search for model 'Extreme Gradient Bo
osting)'
[2021-05-19 11:58:20.833356] Finish parameter search for model 'Extreme Gradient B
oosting)' (time: 26 seconds)

=== train : best params ===
{'learning_rate': 0.05, 'max_depth': 5, 'n_estimators': 100, 'subsample': 0.8}
=== train : best score : roc_auc ===
0.813223549696321



('k-Nearest Neighbours)', KNeighborsClassifier())
[2021-05-19 11:58:20.834353] Start parameter search for model 'k-Nearest Neighbour
s)'
[2021-05-19 11:58:28.707184] Finish parameter search for model 'k-Nearest Neighbou
rs)' (time: 7 seconds)

=== train : best params ===
{'n_neighbors': 60, 'weights': 'distance'}
=== train : best score : roc_auc ===
0.7882993141993747
```

```
('Decision Tree', DecisionTreeClassifier())
[2021-05-19 11:58:28.708182] Start parameter search for model 'Decision Tree'
[2021-05-19 11:58:29.273135] Finish parameter search for model 'Decision Tree' (ti
me: 0 seconds)

=== train : best params ===
{'criterion': 'entropy', 'max_depth': 5}
=== train : best score : roc_auc ===
0.7959138566837142


('Extra Tree', ExtraTreesClassifier())
[2021-05-19 11:58:29.273135] Start parameter search for model 'Extra Tree'
[2021-05-19 11:58:32.103962] Finish parameter search for model 'Extra Tree' (time:
2 seconds)

=== train : best params ===
{'max_features': 'log2', 'n_estimators': 100}
=== train : best score : roc_auc ===
0.805058837428837


('Gradient Boosting', GradientBoostingClassifier())
[2021-05-19 11:58:32.104959] Start parameter search for model 'Gradient Boosting'
[2021-05-19 11:58:48.065719] Finish parameter search for model 'Gradient Boosting'
(time: 15 seconds)

=== train : best params ===
{'learning_rate': 0.01, 'max_depth': 8, 'max_features': 6, 'n_estimators': 100}
=== train : best score : roc_auc ===
0.8120397073127112


('Adaptive Boosting', AdaBoostClassifier())
[2021-05-19 11:58:48.065719] Start parameter search for model 'Adaptive Boosting'
[2021-05-19 11:58:51.628933] Finish parameter search for model 'Adaptive Boosting'
(time: 3 seconds)

=== train : best params ===
{'learning_rate': 0.2, 'n_estimators': 100}
=== train : best score : roc_auc ===
0.80759606485172


('Suppport Vector Classfier', SVC(probability=True))
[2021-05-19 11:58:51.628933] Start parameter search for model 'Suppport Vector Cla
ssfier'
[2021-05-19 12:02:42.000660] Finish parameter search for model 'Suppport Vector Cl
assfier' (time: 230 seconds)

=== train : best params ===
{'C': 1, 'gamma': 0.1, 'kernel': 'rbf'}
=== train : best score : roc_auc ===
0.7846080554772559


('Neural Network', MLPClassifier())
[2021-05-19 12:02:42.001661] Start parameter search for model 'Neural Network'
[2021-05-19 12:05:05.142520] Finish parameter search for model 'Neural Network' (t
```

```
ime: 143 seconds)

=== train : best params ===
{'activation': 'logistic', 'alpha': 0.05, 'hidden_layer_sizes': (50,), 'learning_r
ate': 'constant'}
=== train : best score : roc_auc ===
0.8084375886492493



Confusion Matrix:
[[817  64]
 [427 572]]

Classification Report:
              precision    recall  f1-score   support

         0.0       0.66      0.93      0.77       881
         1.0       0.90      0.57      0.70       999

    accuracy                           0.74      1880
   macro avg       0.78      0.75      0.73      1880
weighted avg       0.79      0.74      0.73      1880
```

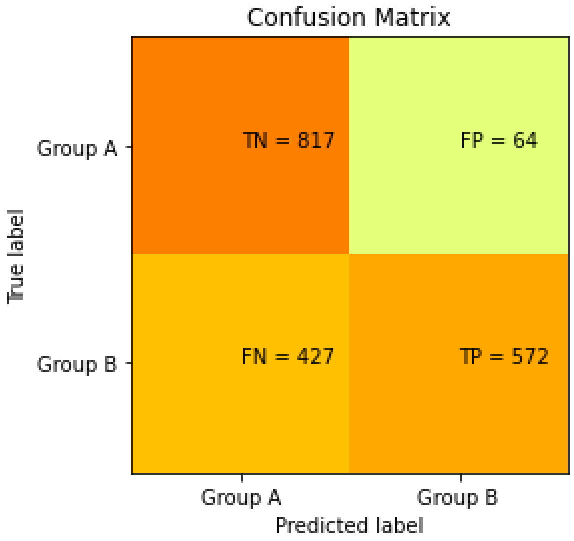


```
AUC Score : 0.7499639253328243

Accuracy Of the Model(LogisticRegression): 0.7388297872340426
```

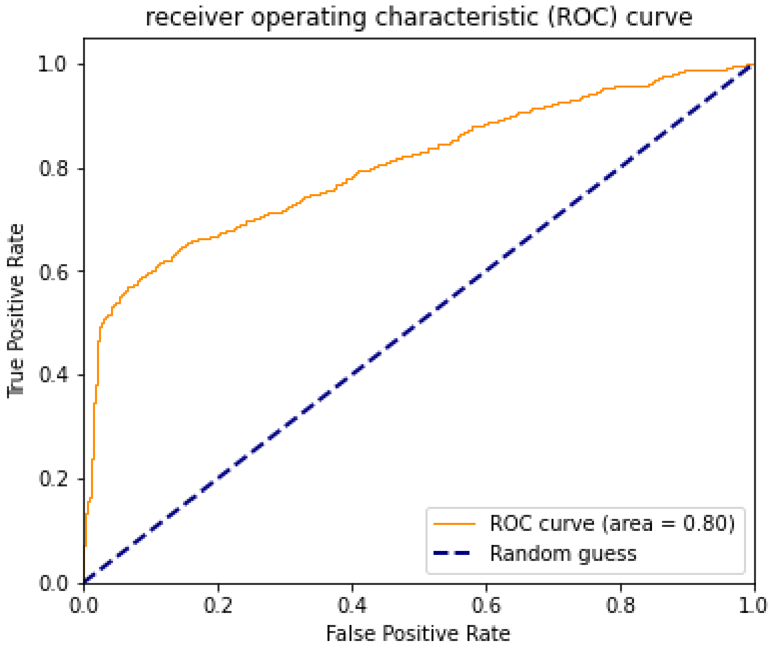

ROC_AUC Score of the model(LogisticRegression): 0.8042764671595546

```
Confusion Matrix:
[[778 103]
 [394 605]]

Classification Report:
              precision    recall  f1-score   support

         0.0       0.66      0.88      0.76       881
         1.0       0.85      0.61      0.71       999

    accuracy                           0.74      1880
   macro avg       0.76      0.74      0.73      1880
weighted avg       0.77      0.74      0.73      1880
```

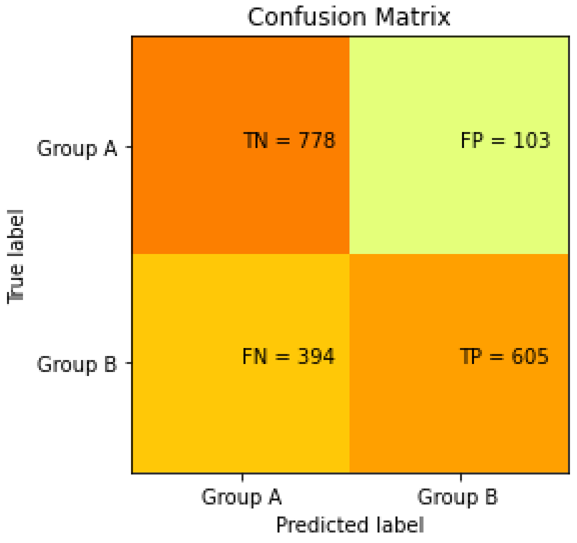


```
AUC Score : 0.7443465031433325

Accuracy Of the Model(RandomForestClassifier): 0.7356382978723405
```

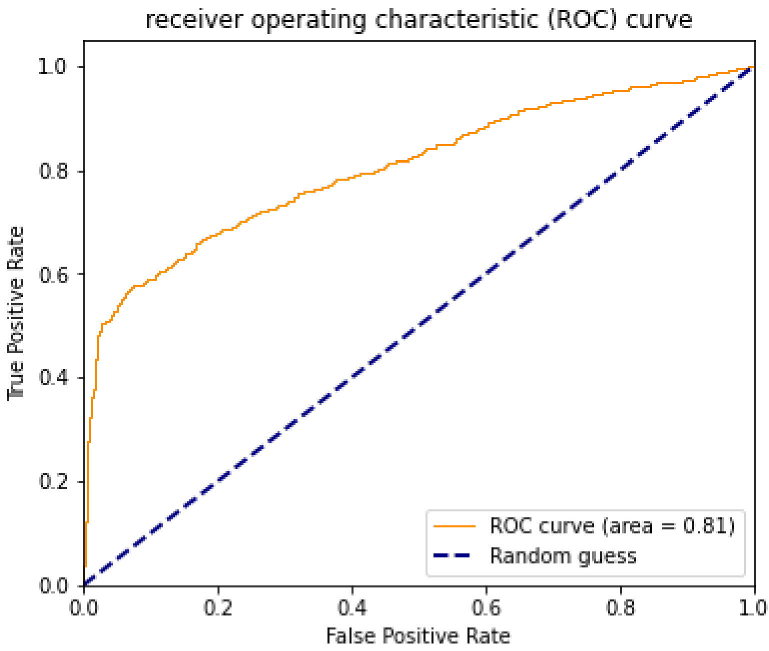

```
ROC_AUC Score of the model(RandomForestClassifier): 0.8072340217629661


Confusion Matrix:
[[796  85]
 [400 599]]

Classification Report:
              precision    recall  f1-score   support

         0.0       0.67      0.90      0.77       881
         1.0       0.88      0.60      0.71       999

    accuracy                           0.74      1880
   macro avg       0.77      0.75      0.74      1880
weighted avg       0.78      0.74      0.74      1880
```

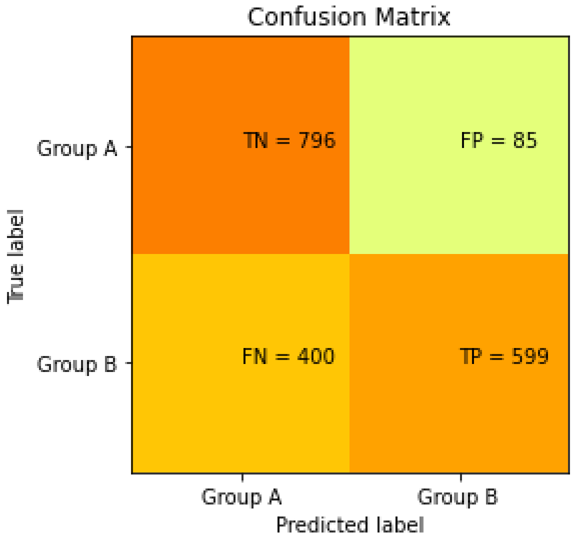


```
AUC Score : 0.7515591641584832

Accuracy Of the Model(XGBClassifier): 0.7420212765957447
```

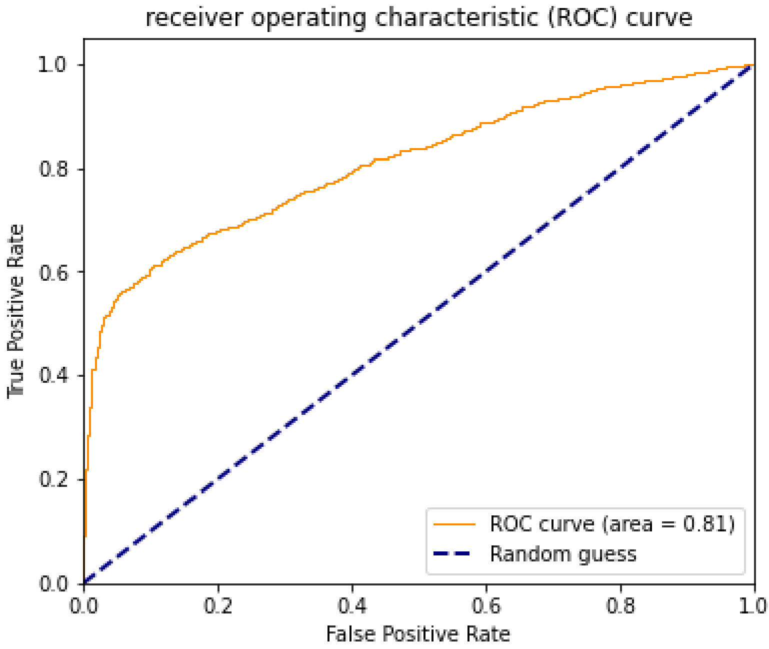

```
ROC_AUC Score of the model(XGBClassifier): 0.8114368625151827


Confusion Matrix:
[[802  79]
 [460 539]]

Classification Report:
              precision    recall  f1-score   support

         0.0       0.64      0.91      0.75       881
         1.0       0.87      0.54      0.67       999

    accuracy                           0.71      1880
   macro avg       0.75      0.72      0.71      1880
weighted avg       0.76      0.71      0.71      1880
```

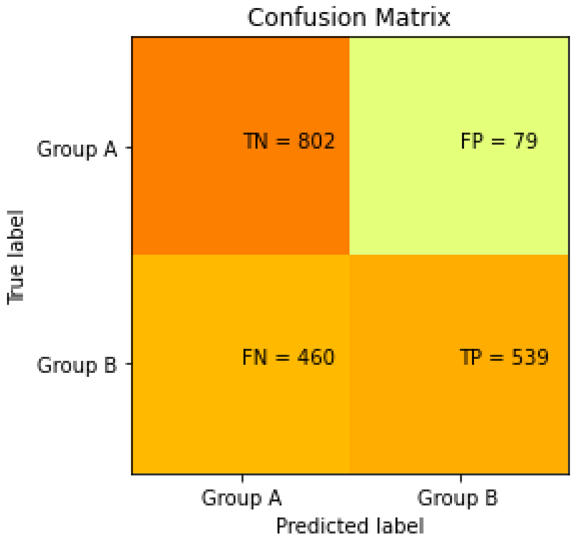


```
AUC Score : 0.7249343554678402

Accuracy Of the Model(KNeighborsClassifier): 0.7132978723404255
```

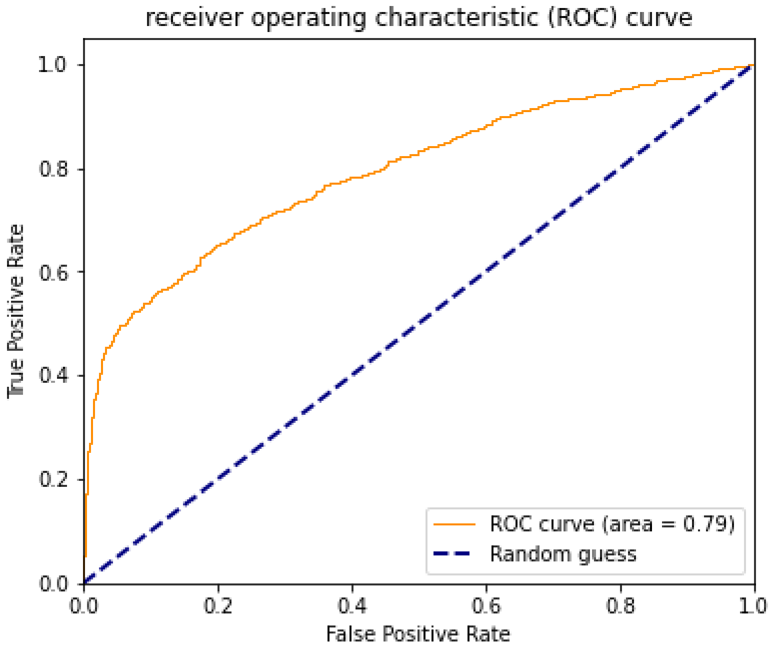

```
ROC_AUC Score of the model(KNeighborsClassifier): 0.7944289351780838


Confusion Matrix:
[[757 124]
 [371 628]]

Classification Report:
              precision    recall  f1-score   support

         0.0       0.67      0.86      0.75       881
         1.0       0.84      0.63      0.72       999

    accuracy                           0.74      1880
   macro avg       0.75      0.74      0.74      1880
weighted avg       0.76      0.74      0.73      1880
```

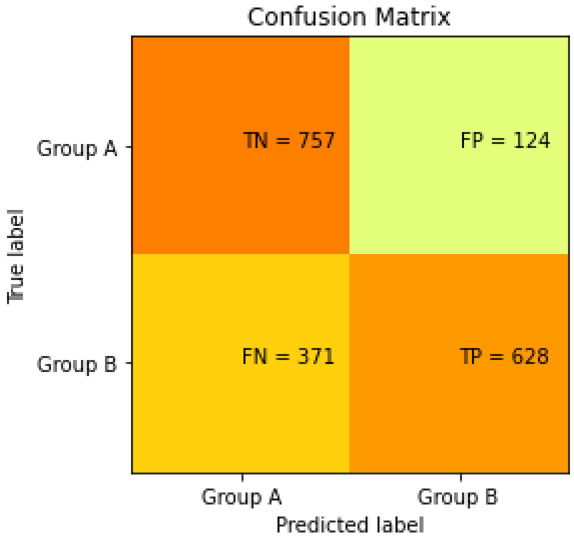


```
AUC Score : 0.7439397399669818

Accuracy Of the Model(DecisionTreeClassifier): 0.7367021276595744
```

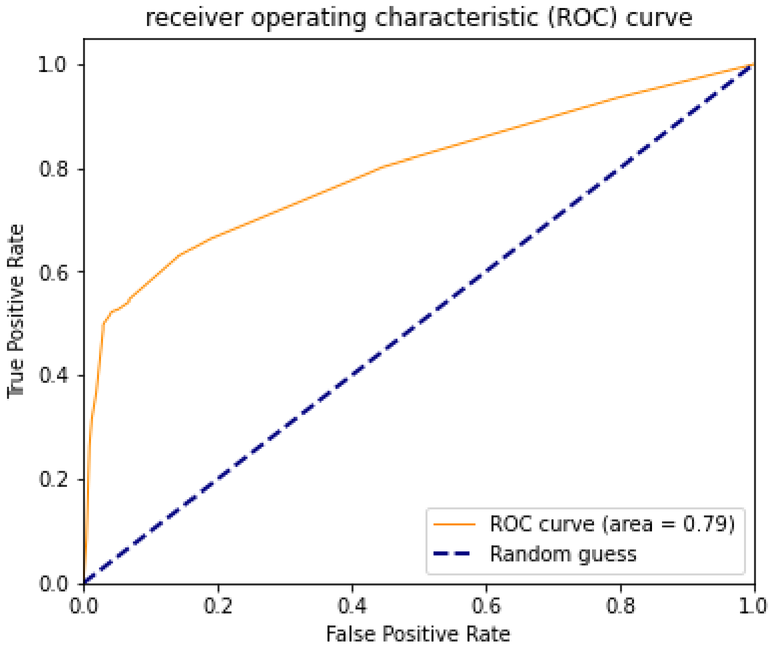

```
ROC_AUC Score of the model(DecisionTreeClassifier): 0.7921218607938244


Confusion Matrix:
[[788  93]
 [417 582]]

Classification Report:
              precision    recall  f1-score   support

         0.0       0.65      0.89      0.76       881
         1.0       0.86      0.58      0.70       999

    accuracy                           0.73      1880
   macro avg       0.76      0.74      0.73      1880
weighted avg       0.76      0.73      0.72      1880
```

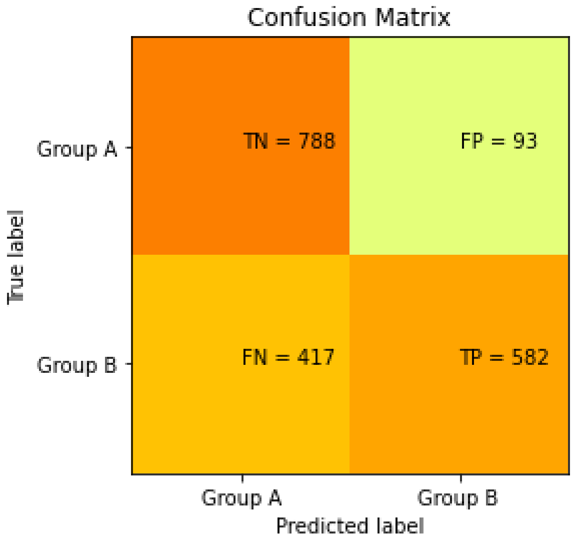


```
AUC Score : 0.7385103605307918

Accuracy Of the Model(ExtraTreesClassifier): 0.7287234042553191
```

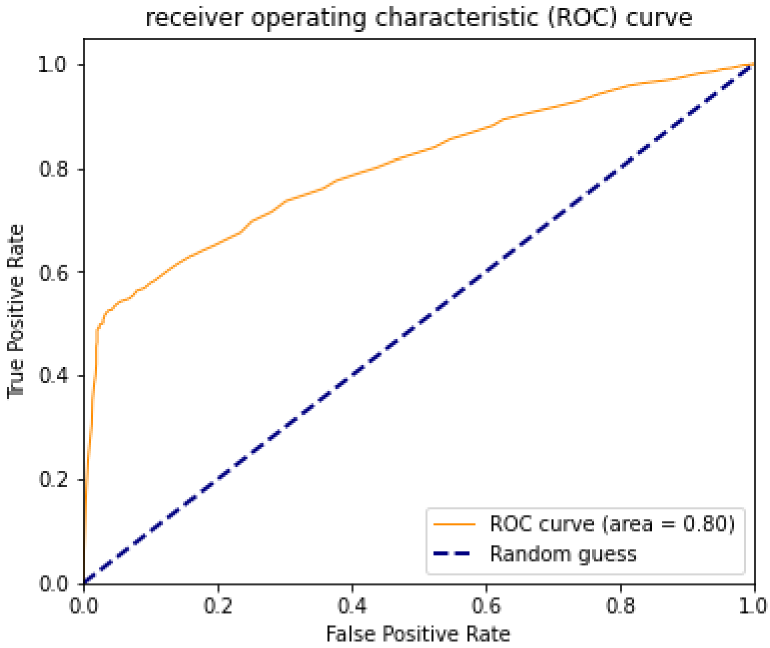

```
ROC_AUC Score of the model(ExtraTreesClassifier): 0.8008127310056935


Confusion Matrix:
[[798  83]
 [401 598]]

Classification Report:
              precision    recall  f1-score   support

         0.0       0.67      0.91      0.77       881
         1.0       0.88      0.60      0.71       999

    accuracy                           0.74      1880
   macro avg       0.77      0.75      0.74      1880
weighted avg       0.78      0.74      0.74      1880
```

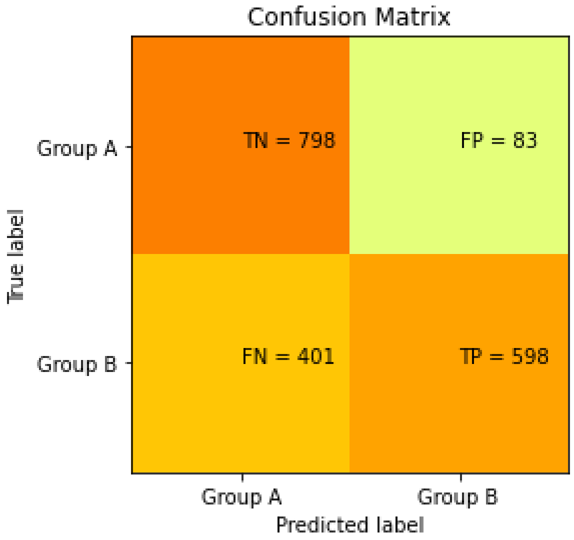


```
AUC Score : 0.7521937374377783

Accuracy Of the Model(GradientBoostingClassifier): 0.7425531914893617
```

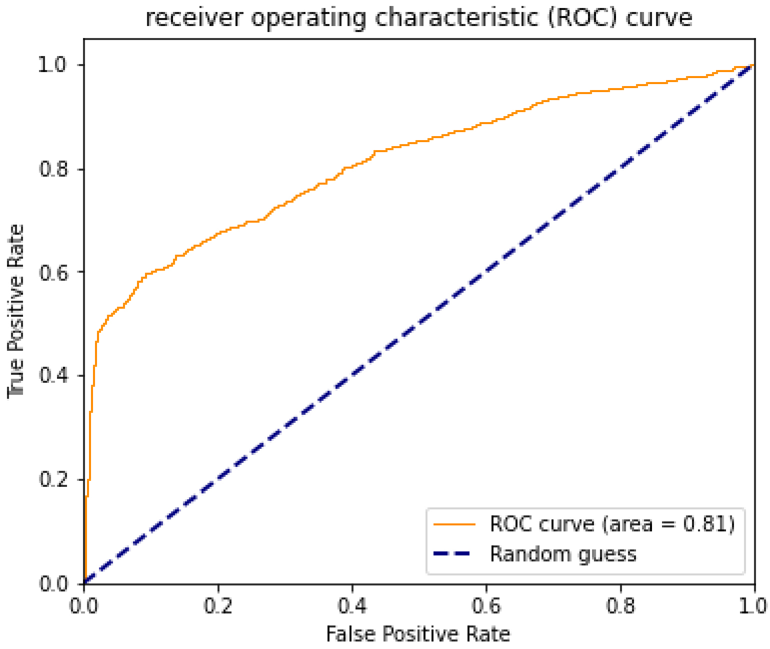

```
ROC_AUC Score of the model(GradientBoostingClassifier): 0.8109403387496463


Confusion Matrix:
[[814  67]
 [420 579]]

Classification Report:
              precision    recall  f1-score   support

         0.0       0.66      0.92      0.77       881
         1.0       0.90      0.58      0.70       999

    accuracy                           0.74      1880
   macro avg       0.78      0.75      0.74      1880
weighted avg       0.79      0.74      0.73      1880
```

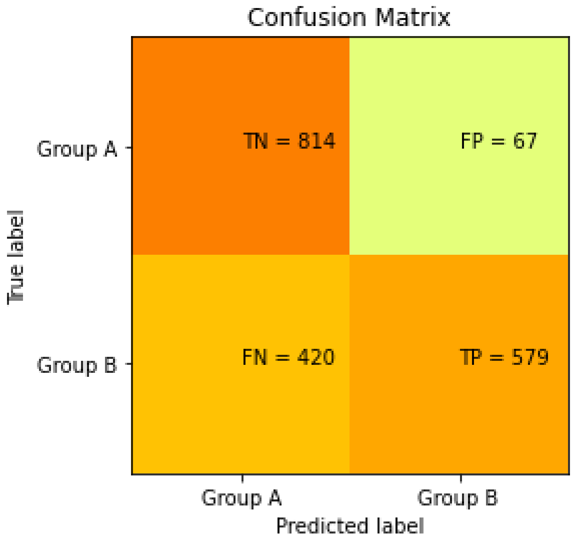


```
AUC Score : 0.7517648181666343

Accuracy Of the Model(AdaBoostClassifier): 0.7409574468085106
```

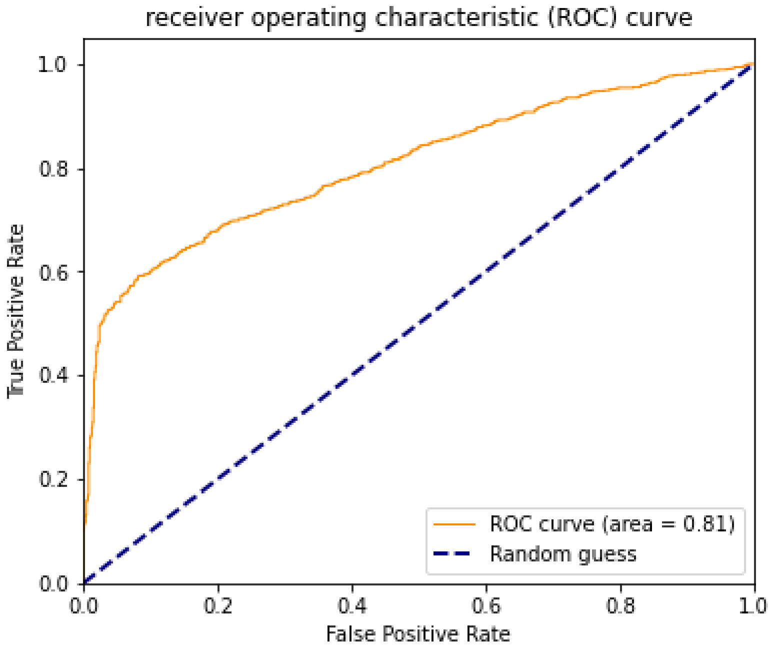

```
ROC_AUC Score of the model(AdaBoostClassifier): 0.8061267851279202


Confusion Matrix:
[[703 178]
 [342 657]]

Classification Report:
              precision    recall  f1-score   support

         0.0       0.67      0.80      0.73       881
         1.0       0.79      0.66      0.72       999

    accuracy                           0.72      1880
   macro avg       0.73      0.73      0.72      1880
weighted avg       0.73      0.72      0.72      1880
```

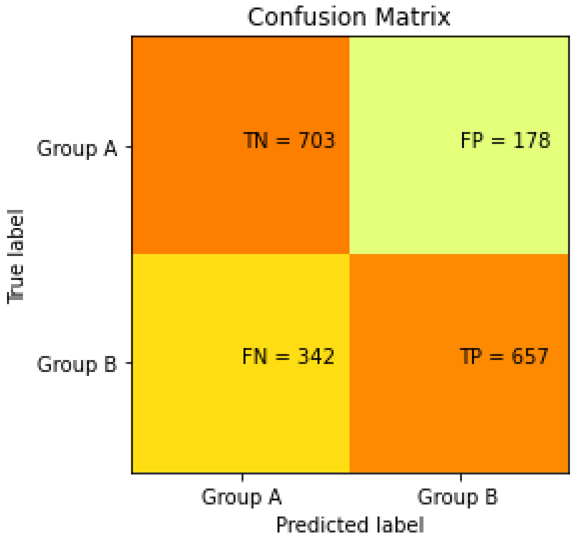


```
AUC Score : 0.7278072624270127

Accuracy Of the Model(SVC): 0.723404255319149
```

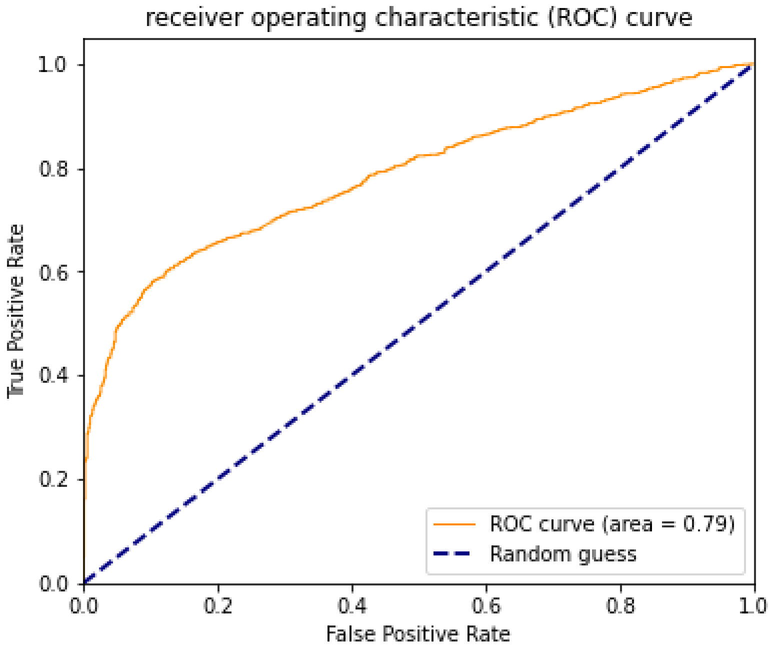

```
ROC_AUC Score of the model(SVC): 0.7858863403698819


Confusion Matrix:
[[788  93]
 [398 601]]

Classification Report:
              precision    recall  f1-score   support

         0.0       0.66      0.89      0.76       881
         1.0       0.87      0.60      0.71       999

    accuracy                           0.74      1880
   macro avg       0.77      0.75      0.74      1880
weighted avg       0.77      0.74      0.73      1880
```

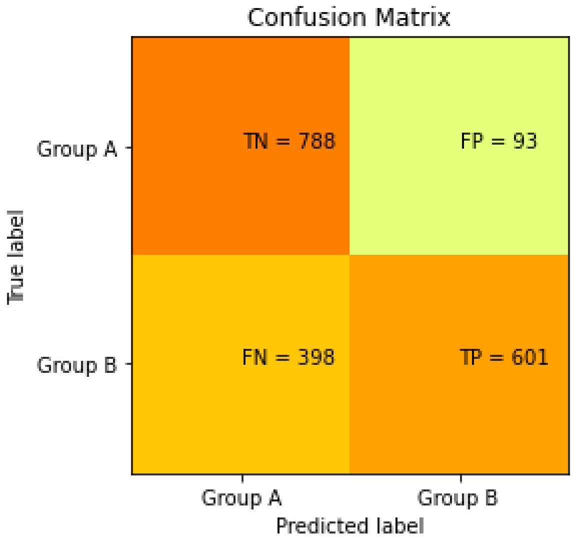


```
AUC Score : 0.7480198700403013

Accuracy Of the Model(MLPClassifier): 0.7388297872340426
```

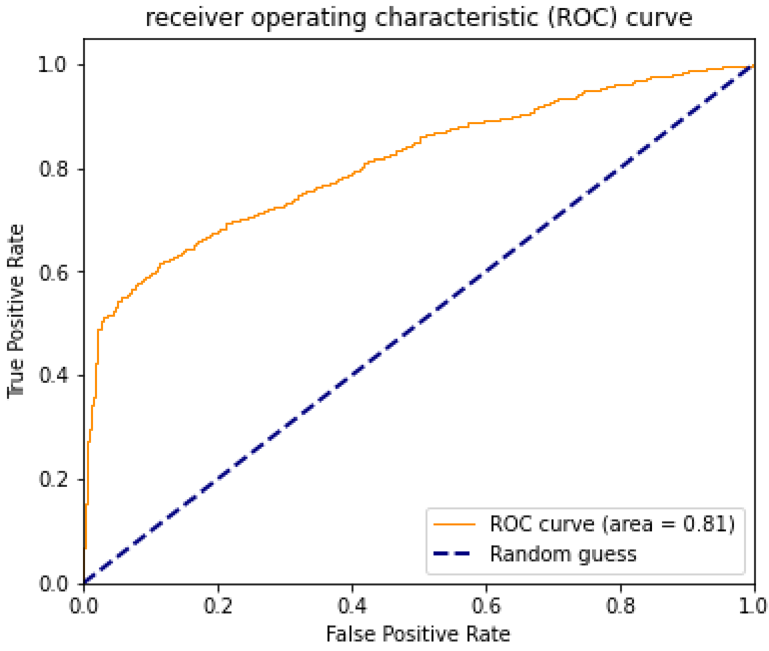

```
ROC_AUC Score of the model(MLPClassifier): 0.8115561645641101

[('GradientBoostingClassifier', 0.7425531914893617), ('XGBClassifier', 0.7420212765957447), ('AdaBoostClassifier', 0.7409574468085106), ('LogisticRegression', 0.7388297872340426), ('MLPClassifier', 0.7388297872340426), ('DecisionTreeClassifier', 0.7367021276595744), ('RandomForestClassifier', 0.7356382978723405), ('ExtraTreesClassifier', 0.7287234042553191), ('SVC', 0.723404255319149), ('KNeighborsClassifier', 0.7132978723404255)]


sorted_total_auc:
 [('MLPClassifier', 0.8115561645641101), ('XGBClassifier', 0.8114368625151827), ('GradientBoostingClassifier', 0.8109403387496463), ('RandomForestClassifier', 0.8072340217629661), ('AdaBoostClassifier', 0.8061267851279202), ('LogisticRegression', 0.8042764671595546), ('ExtraTreesClassifier', 0.8008127310056935), ('KNeighborsClassifier', 0.7944289351780838), ('DecisionTreeClassifier', 0.7921218607938244), ('SVC', 0.7858863403698819)]
```

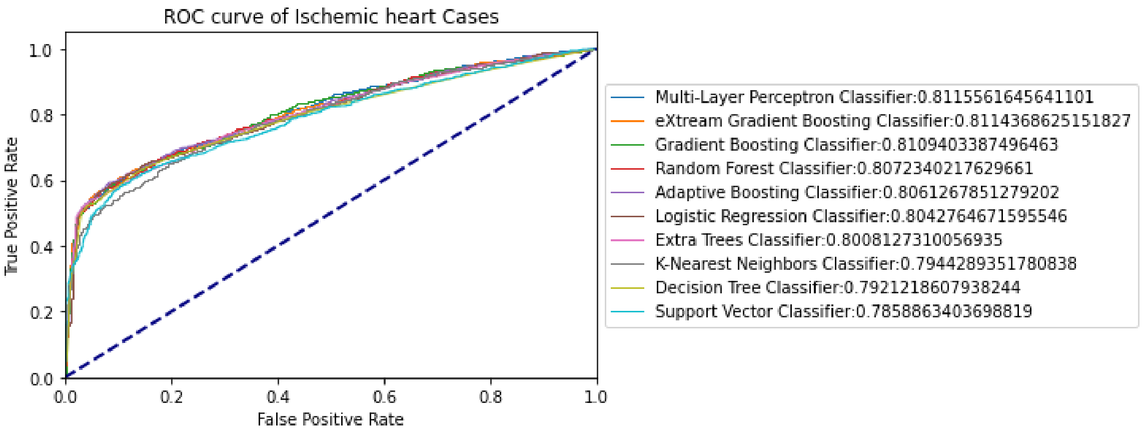

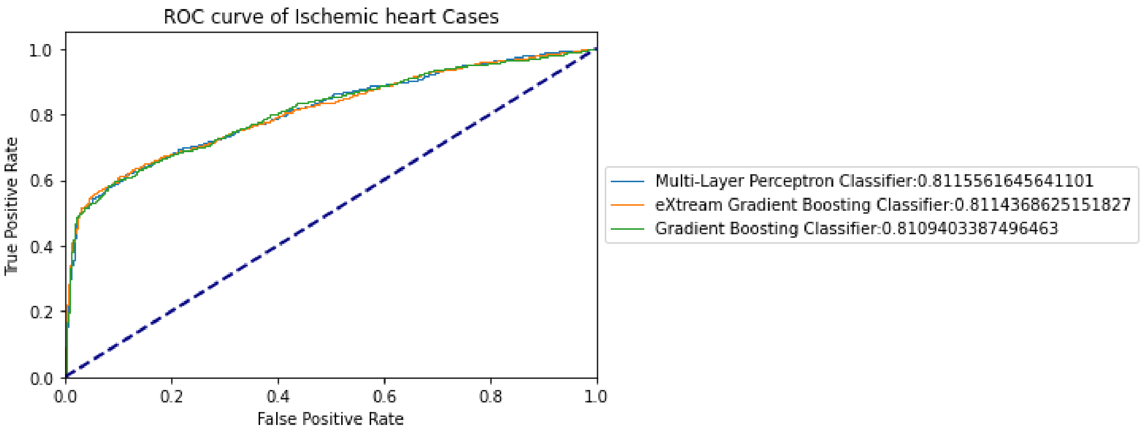

```
random state :   12390
9398
result_train_filename: data/train_test_data_ischemic heart_2013_20210519_120512.cs
v
total count : 9398, df_train count : 7518, df_test count : 1880
(7518, 38)
(1880, 38)
('Logistic Regression', LogisticRegression())
[2021-05-19 12:05:12.914339] Start parameter search for model 'Logistic Regressio
n'
[2021-05-19 12:05:13.274283] Finish parameter search for model 'Logistic Regressio
n' (time: 0 seconds)

=== train : best params ===
{'C': 0.1, 'penalty': 'l2', 'solver': 'lbfgs'}
=== train : best score : roc_auc ===
0.8011007495350629



('Random Forest', RandomForestClassifier())
[2021-05-19 12:05:13.276232] Start parameter search for model 'Random Forest'
[2021-05-19 12:05:17.589416] Finish parameter search for model 'Random Forest' (ti
me: 4 seconds)

=== train : best params ===
{'criterion': 'gini', 'max_depth': 8, 'max_features': 'auto', 'n_estimators': 90}
=== train : best score : roc_auc ===
0.808537504050152



('Extreme Gradient Boosting)', XGBClassifier(base_score=None, booster=None, colsam
ple_bylevel=None,
              colsample_bynode=None, colsample_bytree=None, gamma=None,
              gpu_id=None, importance_type='gain', interaction_constraints=None,
              learning_rate=None, max_delta_step=None, max_depth=None,
              min_child_weight=None, missing=nan, monotone_constraints=None,
              n_estimators=100, n_jobs=None, num_parallel_tree=None,
              random_state=None, reg_alpha=None, reg_lambda=None,
              scale_pos_weight=None, subsample=None, tree_method=None,
              validate_parameters=None, verbosity=None))
[2021-05-19 12:05:17.589416] Start parameter search for model 'Extreme Gradient Bo
osting)'
[2021-05-19 12:05:43.736905] Finish parameter search for model 'Extreme Gradient B
oosting)' (time: 26 seconds)

=== train : best params ===
{'learning_rate': 0.05, 'max_depth': 5, 'n_estimators': 50, 'subsample': 0.7}
=== train : best score : roc_auc ===
0.8103318852719299



('k-Nearest Neighbours)', KNeighborsClassifier())
[2021-05-19 12:05:43.738899] Start parameter search for model 'k-Nearest Neighbour
s)'
[2021-05-19 12:05:51.695829] Finish parameter search for model 'k-Nearest Neighbou
rs)' (time: 7 seconds)

=== train : best params ===
{'n_neighbors': 60, 'weights': 'distance'}
=== train : best score : roc_auc ===
0.7848731556700965
```

```
('Decision Tree', DecisionTreeClassifier())
[2021-05-19 12:05:51.696825] Start parameter search for model 'Decision Tree'
[2021-05-19 12:05:52.302595] Finish parameter search for model 'Decision Tree' (ti
me: 0 seconds)

=== train : best params ===
{'criterion': 'entropy', 'max_depth': 4}
=== train : best score : roc_auc ===
0.7910151370782861


('Extra Tree', ExtraTreesClassifier())
[2021-05-19 12:05:52.302595] Start parameter search for model 'Extra Tree'
[2021-05-19 12:05:55.357804] Finish parameter search for model 'Extra Tree' (time:
3 seconds)

=== train : best params ===
{'max_features': 'sqrt', 'n_estimators': 100}
=== train : best score : roc_auc ===
0.8005645182533307


('Gradient Boosting', GradientBoostingClassifier())
[2021-05-19 12:05:55.358802] Start parameter search for model 'Gradient Boosting'
[2021-05-19 12:06:12.032631] Finish parameter search for model 'Gradient Boosting'
(time: 16 seconds)

=== train : best params ===
{'learning_rate': 0.01, 'max_depth': 8, 'max_features': 6, 'n_estimators': 100}
=== train : best score : roc_auc ===
0.8093265284208802


('Adaptive Boosting', AdaBoostClassifier())
[2021-05-19 12:06:12.032631] Start parameter search for model 'Adaptive Boosting'
[2021-05-19 12:06:15.578548] Finish parameter search for model 'Adaptive Boosting'
(time: 3 seconds)

=== train : best params ===
{'learning_rate': 0.2, 'n_estimators': 100}
=== train : best score : roc_auc ===
0.8021102991210665


('Suppport Vector Classfier', SVC(probability=True))
[2021-05-19 12:06:15.578548] Start parameter search for model 'Suppport Vector Cla
ssfier'
[2021-05-19 12:10:15.420873] Finish parameter search for model 'Suppport Vector Cl
assfier' (time: 239 seconds)

=== train : best params ===
{'C': 1, 'gamma': 0.1, 'kernel': 'rbf'}
=== train : best score : roc_auc ===
0.7805807414841208


('Neural Network', MLPClassifier())
[2021-05-19 12:10:15.420873] Start parameter search for model 'Neural Network'
[2021-05-19 12:12:58.572635] Finish parameter search for model 'Neural Network' (t
```

```
ime: 163 seconds)

=== train : best params ===
{'activation': 'logistic', 'alpha': 0.05, 'hidden_layer_sizes': (50,), 'learning_r
ate': 'adaptive'}
=== train : best score : roc_auc ===
0.805905439841877



Confusion Matrix:
[[827  54]
 [422 577]]

Classification Report:
              precision    recall  f1-score   support

         0.0       0.66      0.94      0.78       881
         1.0       0.91      0.58      0.71       999

    accuracy                           0.75      1880
   macro avg       0.79      0.76      0.74      1880
weighted avg       0.80      0.75      0.74      1880
```

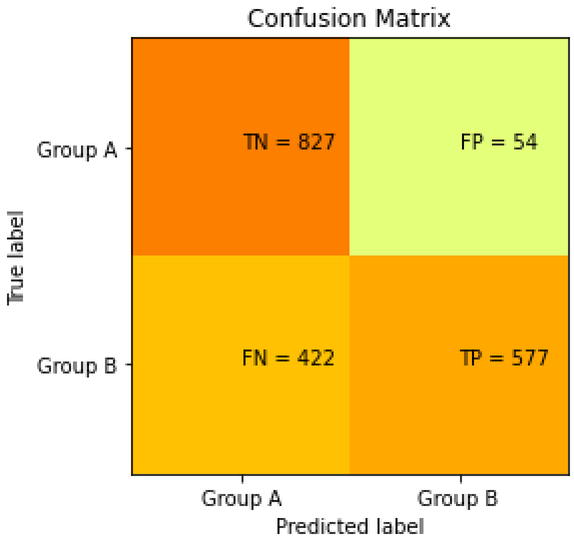


```
AUC Score : 0.7581417967343053

Accuracy Of the Model(LogisticRegression): 0.7468085106382979
```

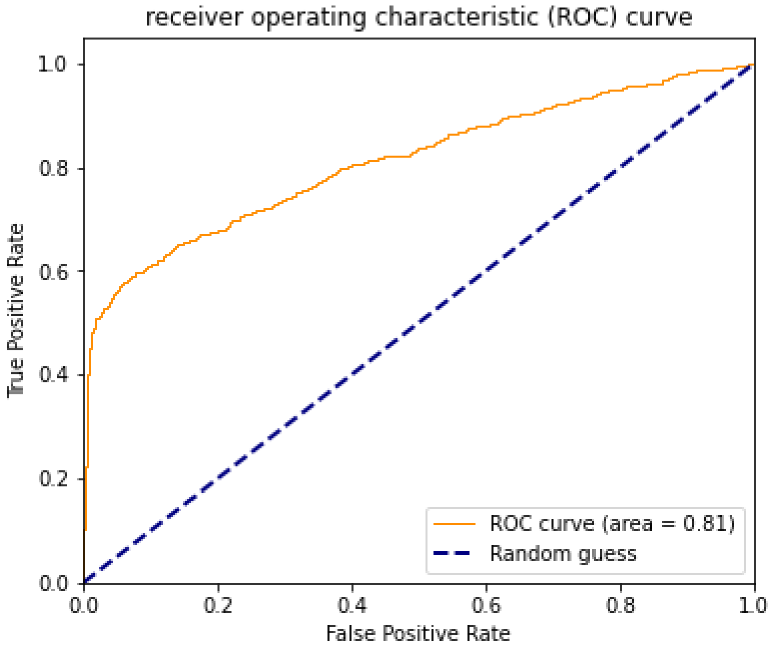


```
ROC_AUC Score of the model(LogisticRegression): 0.8134354558872152


Confusion Matrix:
[[806  75]
 [396 603]]

Classification Report:
              precision    recall  f1-score   support

         0.0       0.67      0.91      0.77       881
         1.0       0.89      0.60      0.72       999

    accuracy                           0.75      1880
   macro avg       0.78      0.76      0.75      1880
weighted avg       0.79      0.75      0.74      1880
```

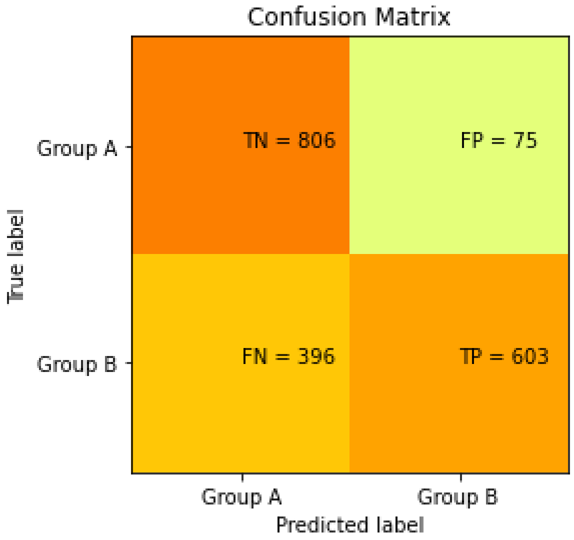


```
AUC Score : 0.7592365350594636

Accuracy Of the Model(RandomForestClassifier): 0.749468085106383
```

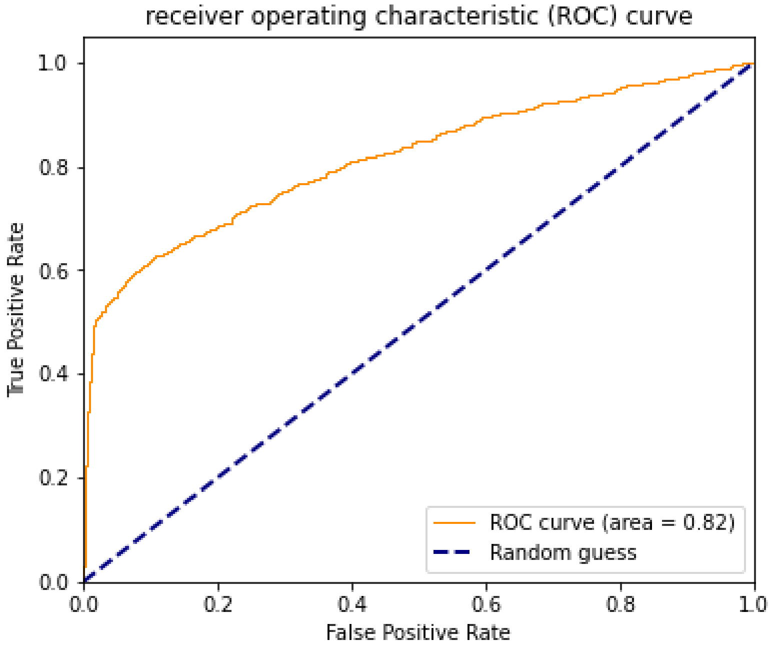

```
ROC_AUC Score of the model(RandomForestClassifier): 0.817022470824968


Confusion Matrix:
[[825  56]
 [412 587]]

Classification Report:
              precision    recall  f1-score   support

         0.0       0.67      0.94      0.78       881
         1.0       0.91      0.59      0.71       999

    accuracy                           0.75      1880
   macro avg       0.79      0.76      0.75      1880
weighted avg       0.80      0.75      0.74      1880
```

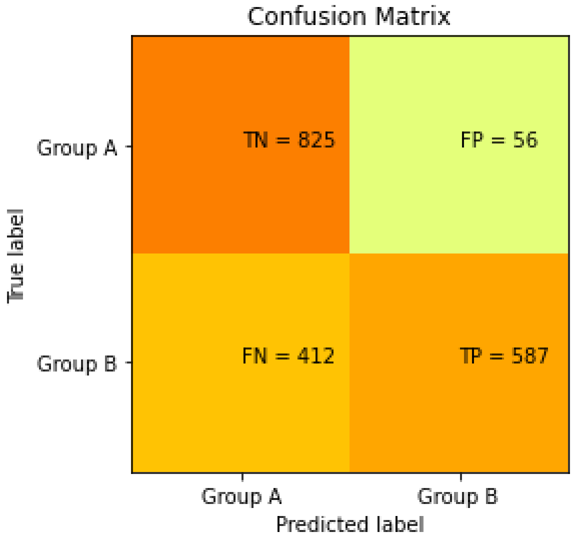


```
AUC Score : 0.7620117279595146

Accuracy Of the Model(XGBClassifier): 0.7510638297872341
```

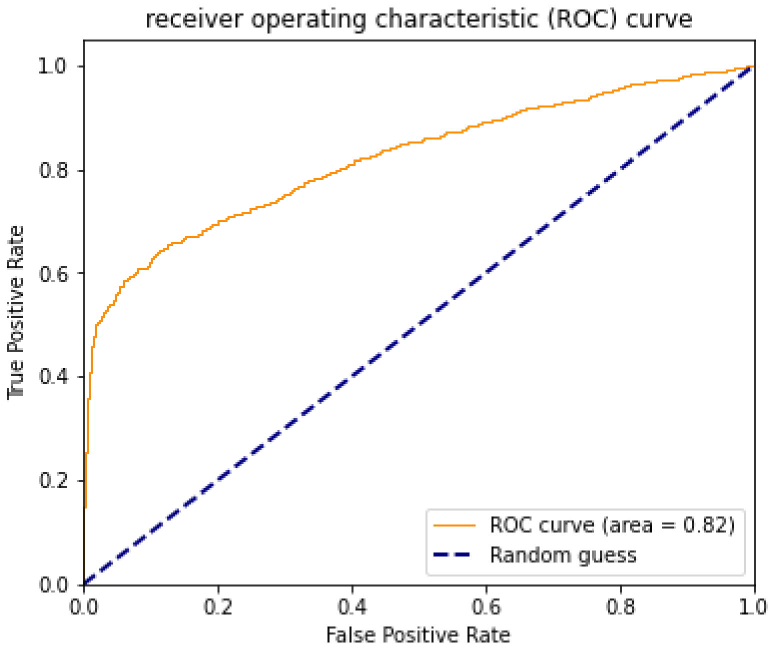

```
ROC_AUC Score of the model(XGBClassifier): 0.8221297347290536


Confusion Matrix:
[[804  77]
 [446 553]]

Classification Report:
              precision    recall  f1-score   support

         0.0       0.64      0.91      0.75       881
         1.0       0.88      0.55      0.68       999

    accuracy                           0.72      1880
   macro avg       0.76      0.73      0.72      1880
weighted avg       0.77      0.72      0.71      1880
```

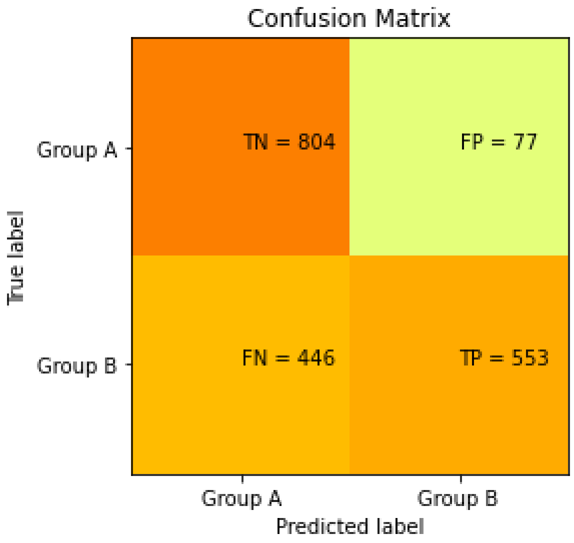


```
AUC Score : 0.7330764362546428

Accuracy Of the Model(KNeighborsClassifier): 0.7218085106382979
```

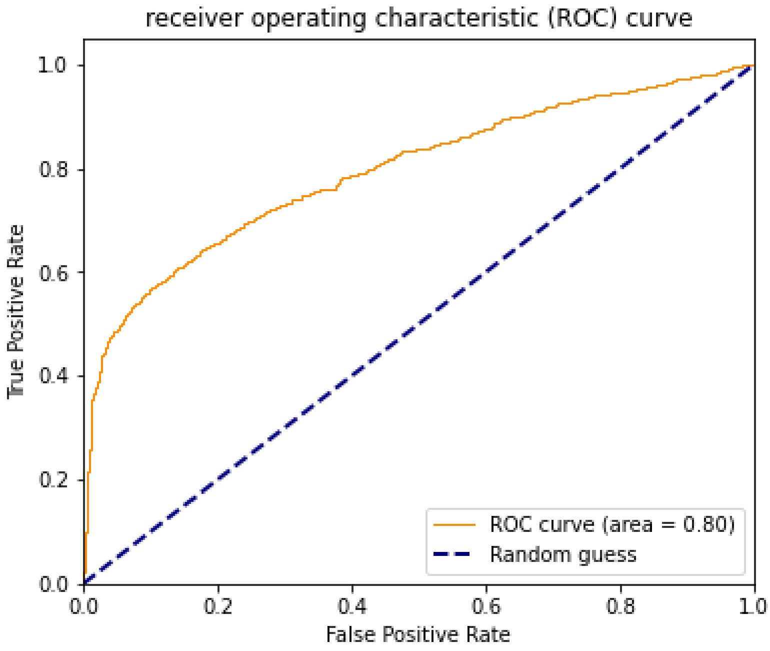

```
ROC_AUC Score of the model(KNeighborsClassifier): 0.797028583634713


Confusion Matrix:
[[798  83]
 [415 584]]

Classification Report:
              precision    recall  f1-score   support

         0.0       0.66      0.91      0.76       881
         1.0       0.88      0.58      0.70       999

    accuracy                           0.74      1880
   macro avg       0.77      0.75      0.73      1880
weighted avg       0.77      0.74      0.73      1880
```

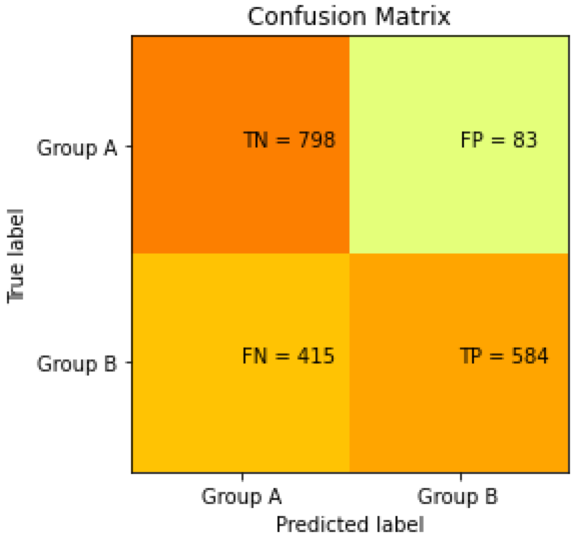


```
AUC Score : 0.7451867304307713

Accuracy Of the Model(DecisionTreeClassifier): 0.7351063829787234
```

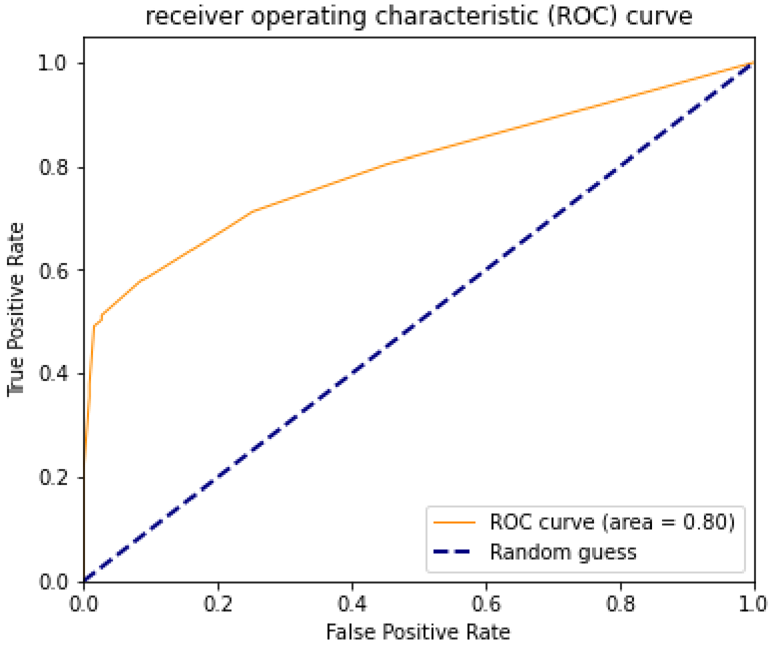

```
ROC_AUC Score of the model(DecisionTreeClassifier): 0.7962866385113831


Confusion Matrix:
[[807  74]
 [416 583]]

Classification Report:
              precision    recall  f1-score   support

         0.0       0.66      0.92      0.77       881
         1.0       0.89      0.58      0.70       999

    accuracy                           0.74      1880
   macro avg       0.77      0.75      0.74      1880
weighted avg       0.78      0.74      0.73      1880
```

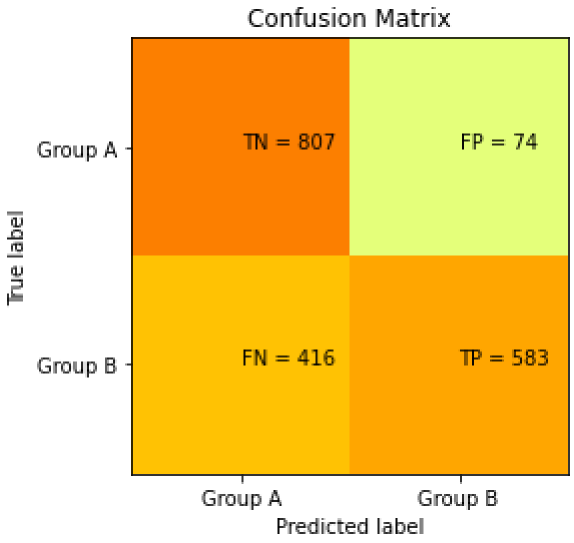


```
AUC Score : 0.7497940619393514

Accuracy Of the Model(ExtraTreesClassifier): 0.7393617021276596
```

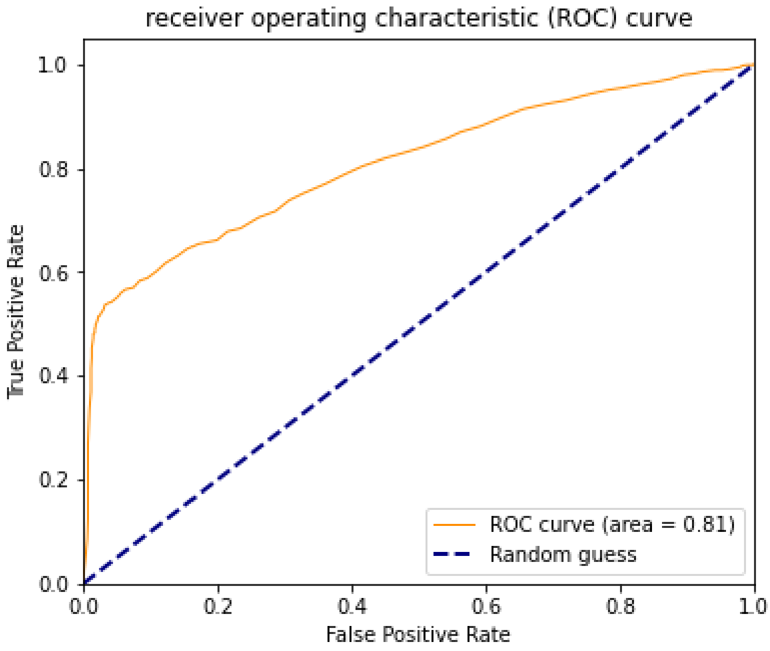

```
ROC_AUC Score of the model(ExtraTreesClassifier): 0.809057070691577


Confusion Matrix:
[[811  70]
 [404 595]]

Classification Report:
              precision    recall  f1-score   support

         0.0       0.67      0.92      0.77       881
         1.0       0.89      0.60      0.72       999

    accuracy                           0.75      1880
   macro avg       0.78      0.76      0.74      1880
weighted avg       0.79      0.75      0.74      1880
```

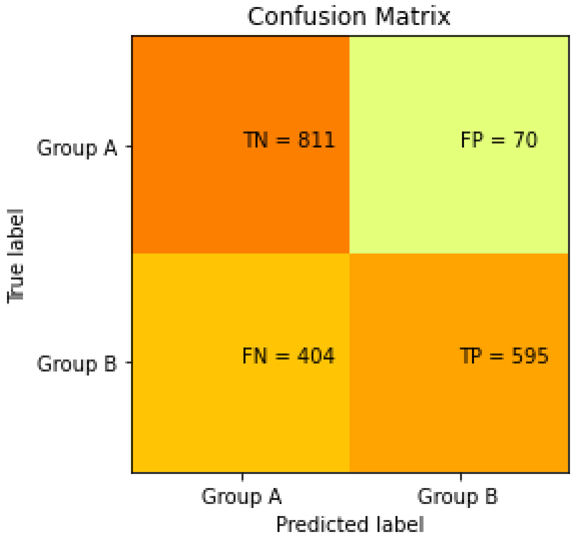


```
AUC Score : 0.7580702155049488

Accuracy Of the Model(GradientBoostingClassifier): 0.747872340425532
```

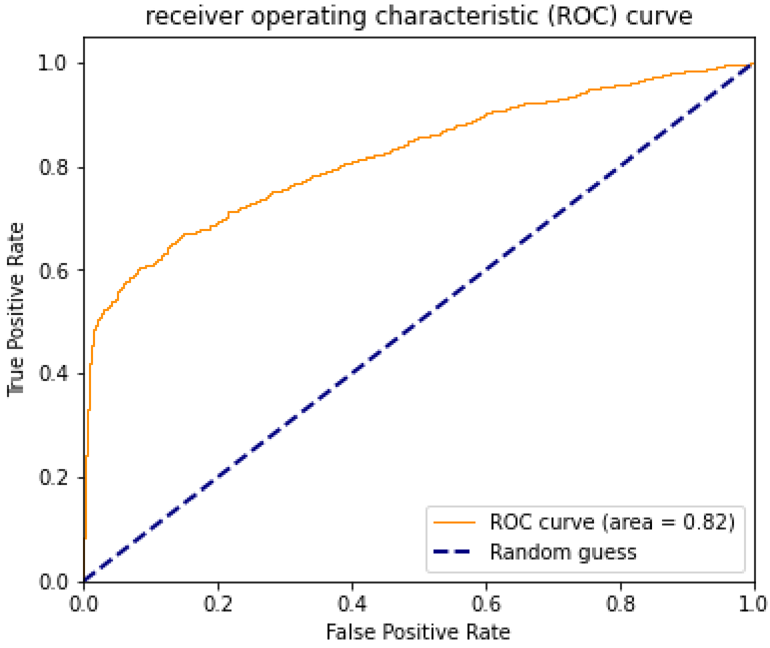

```
ROC_AUC Score of the model(GradientBoostingClassifier): 0.8220263396199832


Confusion Matrix:
[[822  59]
 [428 571]]

Classification Report:
              precision    recall  f1-score   support

         0.0       0.66      0.93      0.77       881
         1.0       0.91      0.57      0.70       999

    accuracy                           0.74      1880
   macro avg       0.78      0.75      0.74      1880
weighted avg       0.79      0.74      0.73      1880
```

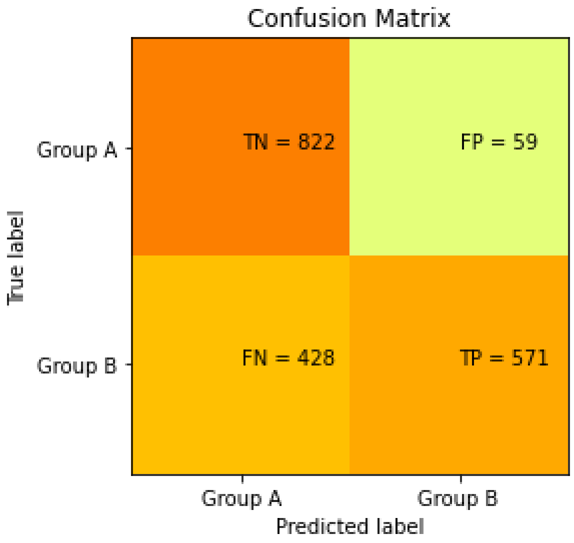


```
AUC Score : 0.752301109281813

Accuracy Of the Model(AdaBoostClassifier): 0.7409574468085106
```

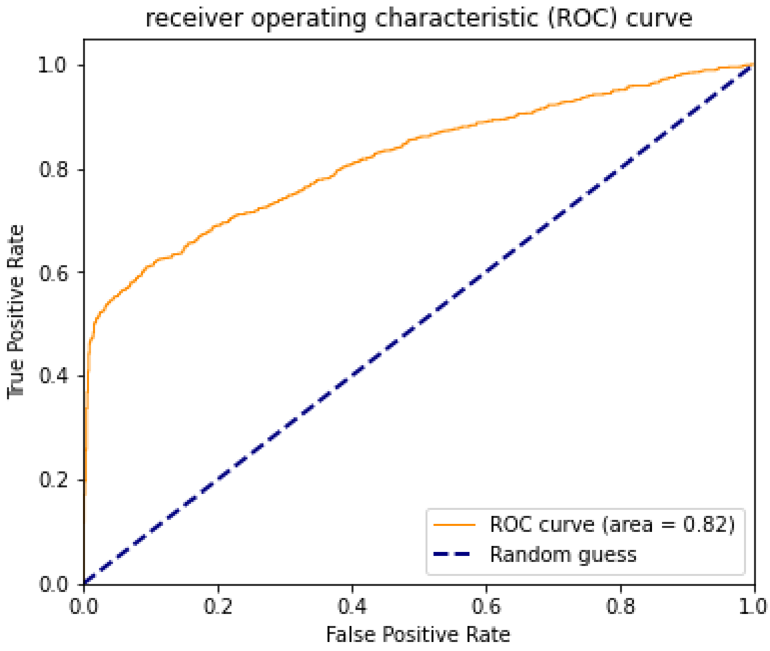

```
ROC_AUC Score of the model(AdaBoostClassifier): 0.8175286523754174


Confusion Matrix:
[[717 164]
 [345 654]]

Classification Report:
              precision    recall  f1-score   support

         0.0       0.68      0.81      0.74       881
         1.0       0.80      0.65      0.72       999

    accuracy                           0.73      1880
   macro avg       0.74      0.73      0.73      1880
weighted avg       0.74      0.73      0.73      1880
```

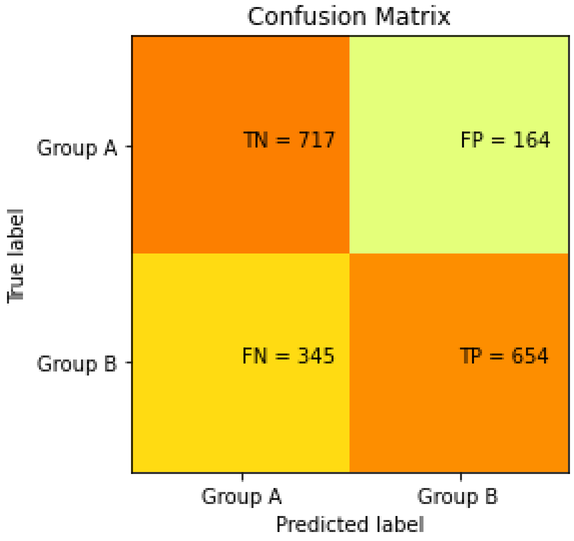


```
AUC Score : 0.734251277384081

Accuracy Of the Model(SVC): 0.7292553191489362
```

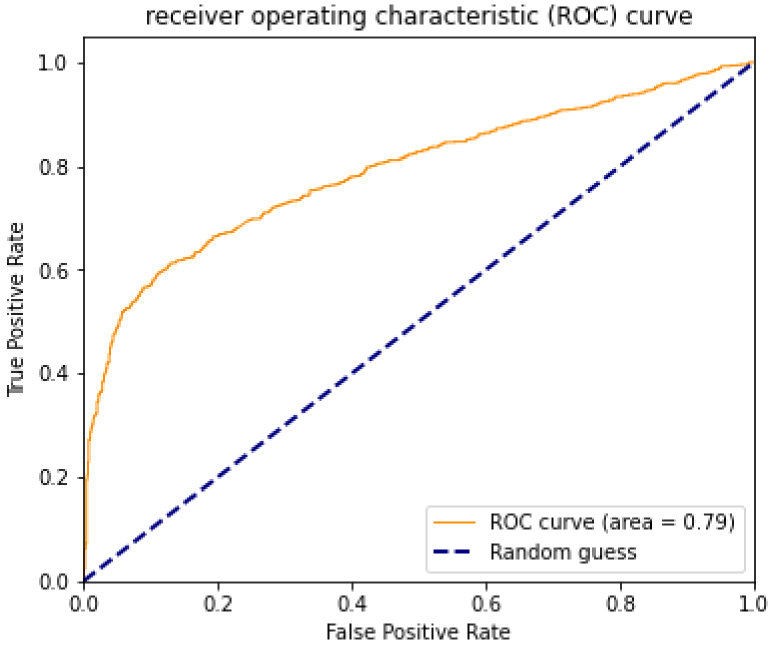

```
ROC_AUC Score of the model(SVC): 0.7895773185216999


Confusion Matrix:
[[816  65]
 [418 581]]

Classification Report:
              precision    recall  f1-score   support

         0.0       0.66      0.93      0.77       881
         1.0       0.90      0.58      0.71       999

    accuracy                           0.74      1880
   macro avg       0.78      0.75      0.74      1880
weighted avg       0.79      0.74      0.74      1880
```

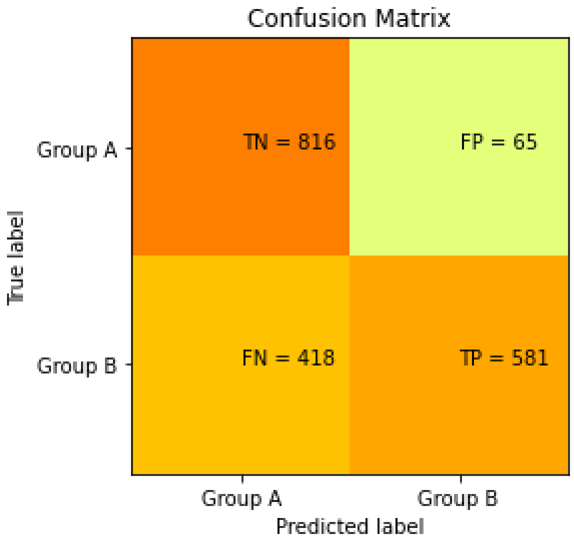


```
AUC Score : 0.7539008929474309

Accuracy Of the Model(MLPClassifier): 0.7430851063829788
```

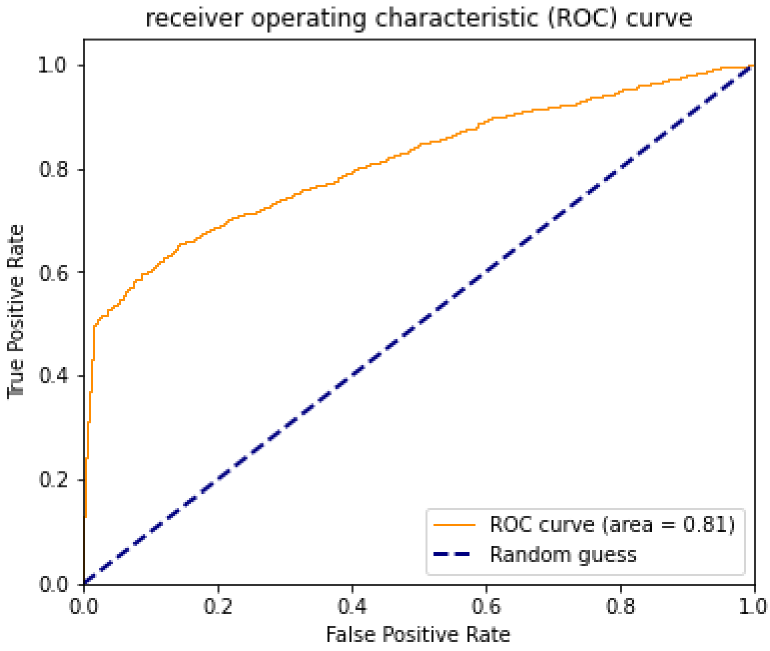

```
ROC_AUC Score of the model(MLPClassifier): 0.813034373760821

[('XGBClassifier', 0.7510638297872341), ('RandomForestClassifier', 0.7494680851063
83), ('GradientBoostingClassifier', 0.747872340425532), ('LogisticRegression', 0.7
468085106382979), ('MLPClassifier', 0.7430851063829788), ('AdaBoostClassifier', 0.
7409574468085106), ('ExtraTreesClassifier', 0.7393617021276596), ('DecisionTreeCla
ssifier', 0.7351063829787234), ('SVC', 0.7292553191489362), ('KNeighborsClassifie
r', 0.7218085106382979)]


sorted_total_auc:
 [('XGBClassifier', 0.8221297347290536), ('GradientBoostingClassifier', 0.82202633
96199832), ('AdaBoostClassifier', 0.8175286523754174), ('RandomForestClassifier',
0.817022470824968), ('LogisticRegression', 0.8134354558872152), ('MLPClassifier',
0.813034373760821), ('ExtraTreesClassifier', 0.809057070691577), ('KNeighborsClass
ifier', 0.797028583634713), ('DecisionTreeClassifier', 0.7962866385113831), ('SV
C', 0.7895773185216999)]
```

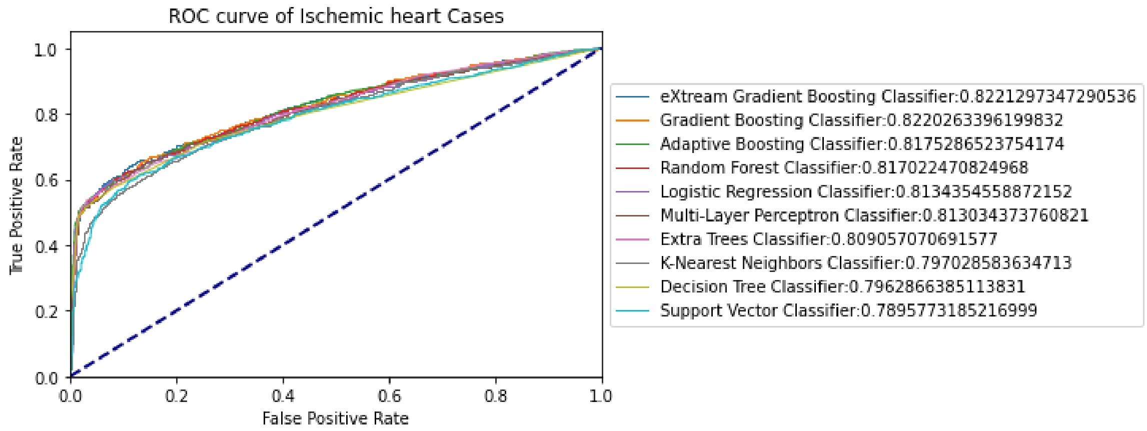

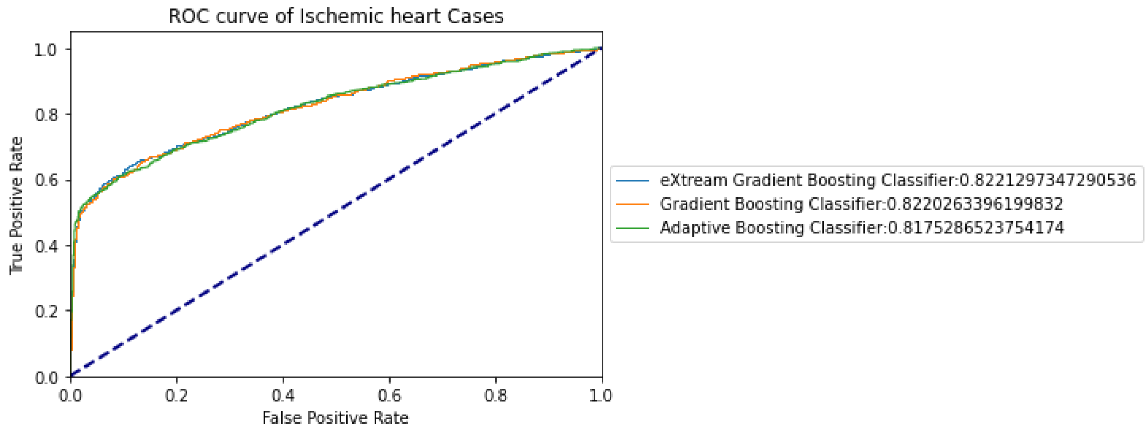

```
random state :   12400
9398
result_train_filename: data/train_test_data_ischemic heart_2013_20210519_121306.cs
v
total count : 9398, df_train count : 7518, df_test count : 1880
(7518, 38)
(1880, 38)
('Logistic Regression', LogisticRegression())
[2021-05-19 12:13:06.334064] Start parameter search for model 'Logistic Regressio
n'
[2021-05-19 12:13:06.658582] Finish parameter search for model 'Logistic Regressio
n' (time: 0 seconds)

=== train : best params ===
{'C': 0.1, 'penalty': 'l2', 'solver': 'lbfgs'}
=== train : best score : roc_auc ===
0.806402001473255



('Random Forest', RandomForestClassifier())
[2021-05-19 12:13:06.660605] Start parameter search for model 'Random Forest'
[2021-05-19 12:13:11.230437] Finish parameter search for model 'Random Forest' (ti
me: 4 seconds)

=== train : best params ===
{'criterion': 'gini', 'max_depth': 8, 'max_features': 'auto', 'n_estimators': 90}
=== train : best score : roc_auc ===
0.8111104062216886



('Extreme Gradient Boosting)', XGBClassifier(base_score=None, booster=None, colsam
ple_bylevel=None,
              colsample_bynode=None, colsample_bytree=None, gamma=None,
              gpu_id=None, importance_type='gain', interaction_constraints=None,
              learning_rate=None, max_delta_step=None, max_depth=None,
              min_child_weight=None, missing=nan, monotone_constraints=None,
              n_estimators=100, n_jobs=None, num_parallel_tree=None,
              random_state=None, reg_alpha=None, reg_lambda=None,
              scale_pos_weight=None, subsample=None, tree_method=None,
              validate_parameters=None, verbosity=None))
[2021-05-19 12:13:11.231435] Start parameter search for model 'Extreme Gradient Bo
osting)'
[2021-05-19 12:13:37.618555] Finish parameter search for model 'Extreme Gradient B
oosting)' (time: 26 seconds)

=== train : best params ===
{'learning_rate': 0.05, 'max_depth': 5, 'n_estimators': 50, 'subsample': 0.8}
=== train : best score : roc_auc ===
0.8135083079792245



('k-Nearest Neighbours)', KNeighborsClassifier())
[2021-05-19 12:13:37.619552] Start parameter search for model 'k-Nearest Neighbour
s)'
[2021-05-19 12:13:45.559876] Finish parameter search for model 'k-Nearest Neighbou
rs)' (time: 7 seconds)

=== train : best params ===
{'n_neighbors': 60, 'weights': 'distance'}
=== train : best score : roc_auc ===
0.7889054763607457
```

```
('Decision Tree', DecisionTreeClassifier())
[2021-05-19 12:13:45.560875] Start parameter search for model 'Decision Tree'
[2021-05-19 12:13:46.111118] Finish parameter search for model 'Decision Tree' (ti
me: 0 seconds)

=== train : best params ===
{'criterion': 'gini', 'max_depth': 4}
=== train : best score : roc_auc ===
0.7947623783344081


('Extra Tree', ExtraTreesClassifier())
[2021-05-19 12:13:46.111118] Start parameter search for model 'Extra Tree'
[2021-05-19 12:13:48.943088] Finish parameter search for model 'Extra Tree' (time:
2 seconds)

=== train : best params ===
{'max_features': 'log2', 'n_estimators': 100}
=== train : best score : roc_auc ===
0.8054440811020318


('Gradient Boosting', GradientBoostingClassifier())
[2021-05-19 12:13:48.943088] Start parameter search for model 'Gradient Boosting'
[2021-05-19 12:14:04.621096] Finish parameter search for model 'Gradient Boosting'
(time: 15 seconds)

=== train : best params ===
{'learning_rate': 0.01, 'max_depth': 8, 'max_features': 6, 'n_estimators': 100}
=== train : best score : roc_auc ===
0.8132026712113816


('Adaptive Boosting', AdaBoostClassifier())
[2021-05-19 12:14:04.622091] Start parameter search for model 'Adaptive Boosting'
[2021-05-19 12:14:08.158235] Finish parameter search for model 'Adaptive Boosting'
(time: 3 seconds)

=== train : best params ===
{'learning_rate': 0.2, 'n_estimators': 100}
=== train : best score : roc_auc ===
0.8082715352291686


('Suppport Vector Classfier', SVC(probability=True))
[2021-05-19 12:14:08.159233] Start parameter search for model 'Suppport Vector Cla
ssfier'
[2021-05-19 12:17:56.494131] Finish parameter search for model 'Suppport Vector Cl
assfier' (time: 228 seconds)

=== train : best params ===
{'C': 1, 'gamma': 0.1, 'kernel': 'rbf'}
=== train : best score : roc_auc ===
0.782533361152354


('Neural Network', MLPClassifier())
[2021-05-19 12:17:56.495124] Start parameter search for model 'Neural Network'
[2021-05-19 12:20:38.519001] Finish parameter search for model 'Neural Network' (t
```

```
ime: 162 seconds)

=== train : best params ===
{'activation': 'logistic', 'alpha': 0.05, 'hidden_layer_sizes': (50,), 'learning_r
ate': 'constant'}
=== train : best score : roc_auc ===
0.8110306968396932



Confusion Matrix:
[[815  66]
 [427 572]]

Classification Report:
              precision    recall  f1-score   support

         0.0       0.66      0.93      0.77       881
         1.0       0.90      0.57      0.70       999

    accuracy                           0.74      1880
   macro avg       0.78      0.75      0.73      1880
weighted avg       0.78      0.74      0.73      1880
```

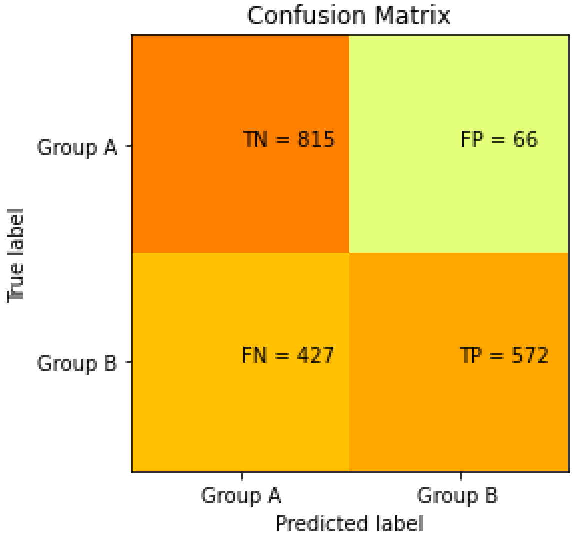


```
AUC Score : 0.7488288515530286

Accuracy Of the Model(LogisticRegression): 0.7377659574468085
```

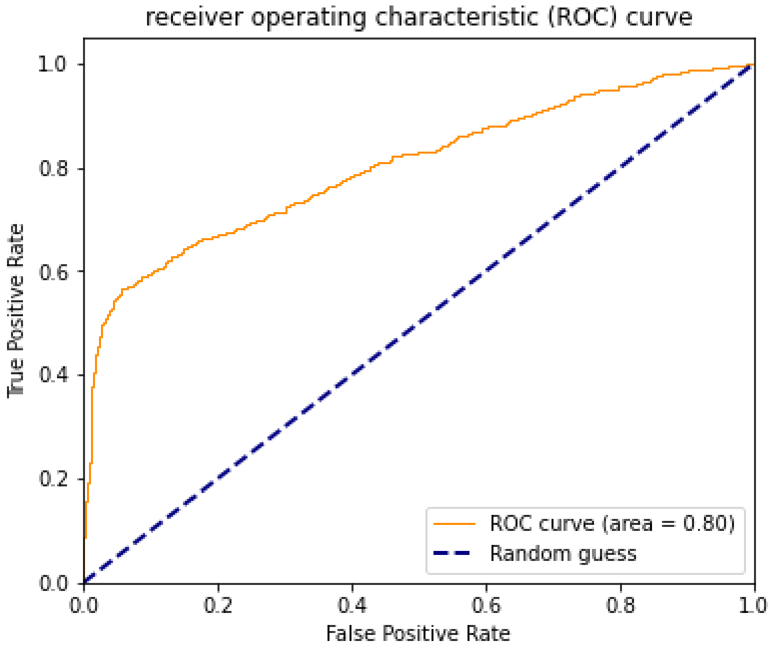

ROC_AUC Score of the model(LogisticRegression): 0.8032538781687477

```
Confusion Matrix:
[[785  96]
 [395 604]]

Classification Report:
              precision    recall  f1-score   support

         0.0       0.67      0.89      0.76       881
         1.0       0.86      0.60      0.71       999

    accuracy                           0.74      1880
   macro avg       0.76      0.75      0.74      1880
weighted avg       0.77      0.74      0.73      1880
```

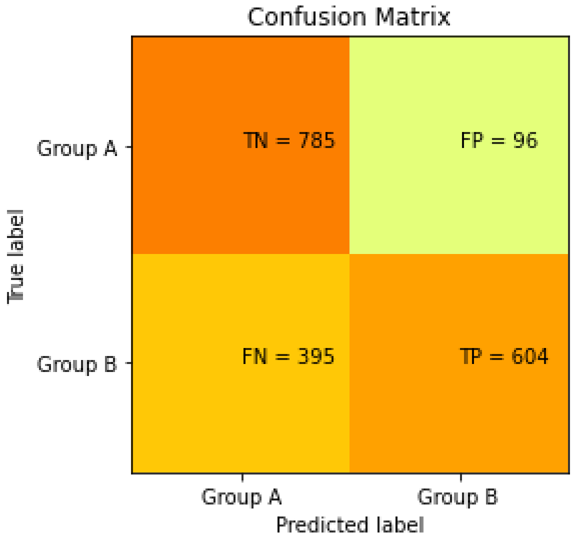

AUC Score : 0.7478187608721094

Accuracy Of the Model(RandomForestClassifier): 0.7388297872340426

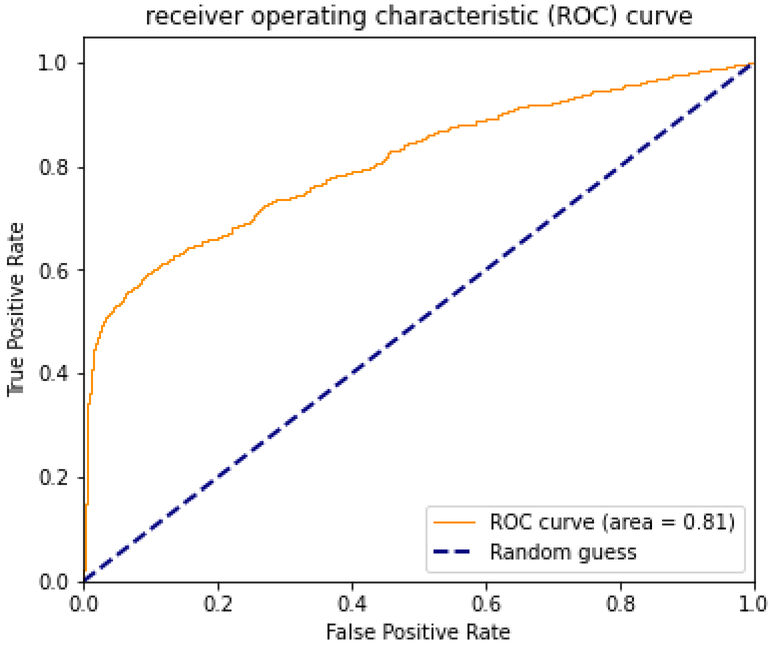

ROC_AUC Score of the model(RandomForestClassifier): 0.8092621565947332

Confusion Matrix:
[[812 69]
 [422 577]]

Classification Report:

	precision	recall	f1-score	support
0.0	0.66	0.92	0.77	881
1.0	0.89	0.58	0.70	999
accuracy			0.74	1880
macro avg	0.78	0.75	0.73	1880
weighted avg	0.78	0.74	0.73	1880

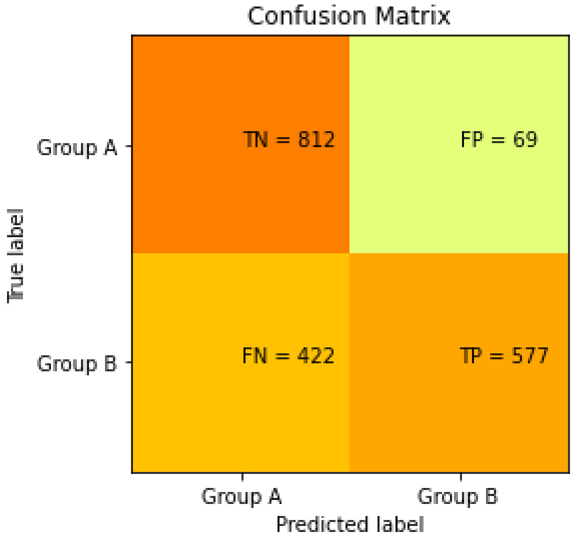

AUC Score : 0.7496287433858375

Accuracy Of the Model(XGBClassifier): 0.7388297872340426

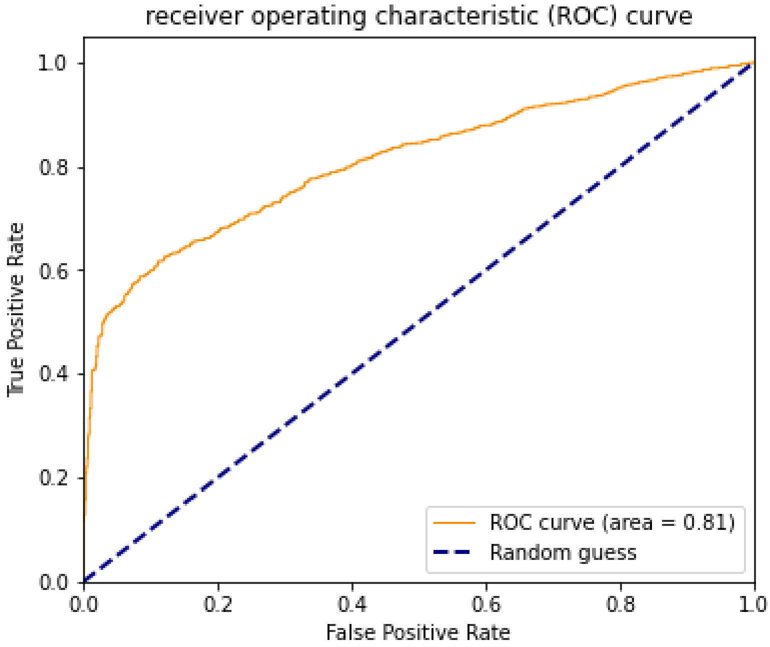

```
ROC_AUC Score of the model(XGBClassifier): 0.8096956207058364


Confusion Matrix:
[[795  86]
 [454 545]]

Classification Report:
              precision    recall  f1-score   support

         0.0       0.64      0.90      0.75       881
         1.0       0.86      0.55      0.67       999

    accuracy                           0.71      1880
   macro avg       0.75      0.72      0.71      1880
weighted avg       0.76      0.71      0.71      1880
```

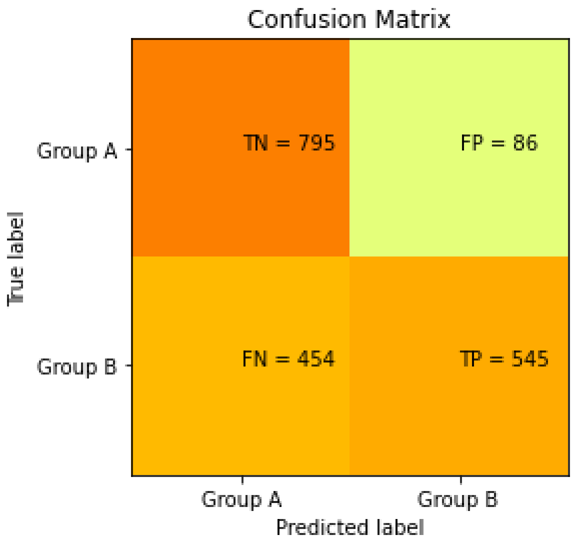


```
AUC Score : 0.7239646002415583

Accuracy Of the Model(KNeighborsClassifier): 0.7127659574468085
```

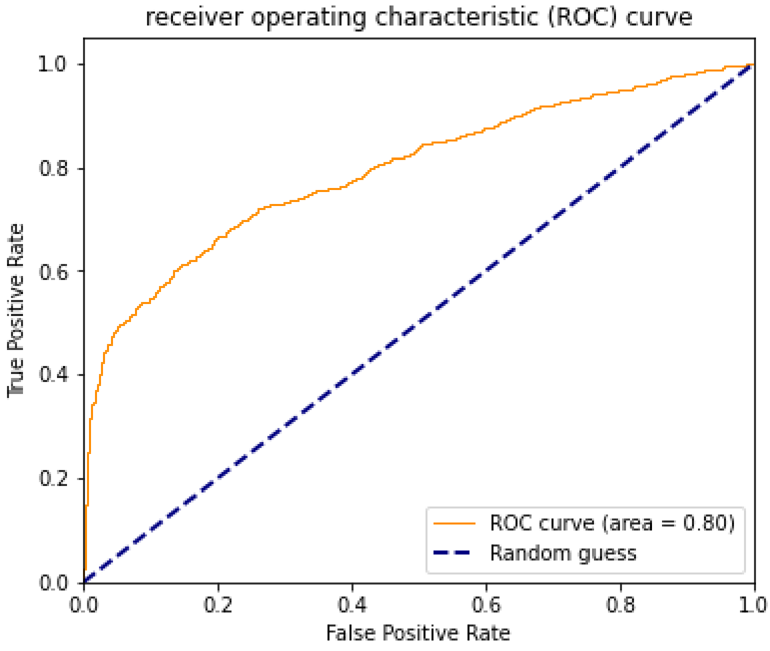

```
ROC_AUC Score of the model(KNeighborsClassifier): 0.7965979600485843


Confusion Matrix:
[[770 111]
 [411 588]]

Classification Report:
              precision    recall  f1-score   support

         0.0       0.65      0.87      0.75       881
         1.0       0.84      0.59      0.69       999

    accuracy                           0.72      1880
   macro avg       0.75      0.73      0.72      1880
weighted avg       0.75      0.72      0.72      1880
```

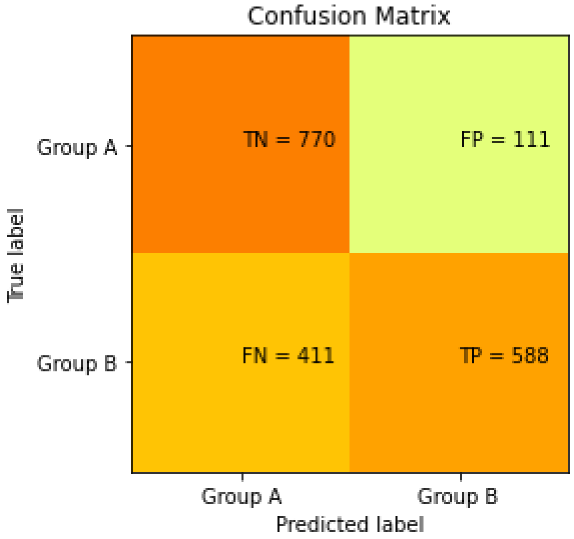


```
AUC Score : 0.7312976995156337

Accuracy Of the Model(DecisionTreeClassifier): 0.7223404255319149
```

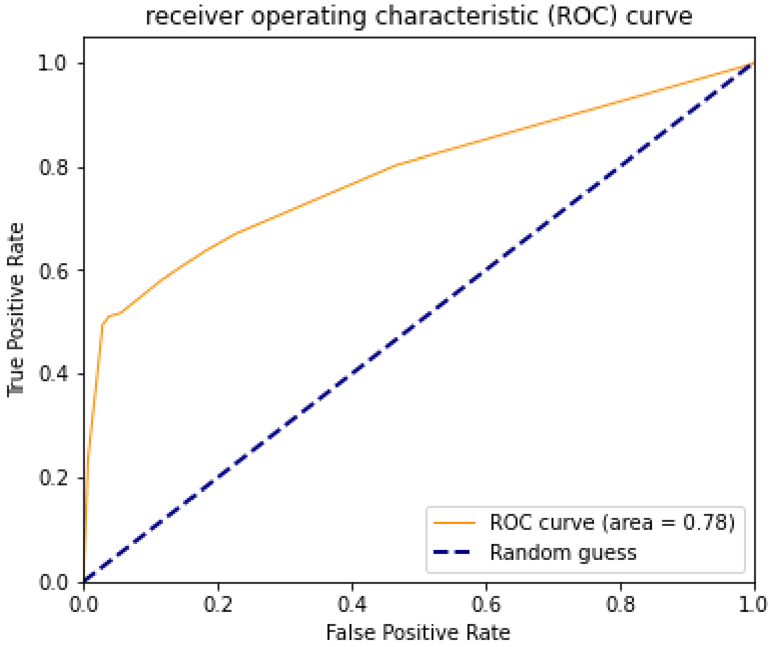

ROC_AUC Score of the model(DecisionTreeClassifier): 0.7821430965585336

```
Confusion Matrix:
[[786  95]
 [411 588]]

Classification Report:
              precision    recall  f1-score   support

         0.0       0.66      0.89      0.76       881
         1.0       0.86      0.59      0.70       999

    accuracy                           0.73      1880
   macro avg       0.76      0.74      0.73      1880
weighted avg       0.77      0.73      0.73      1880
```

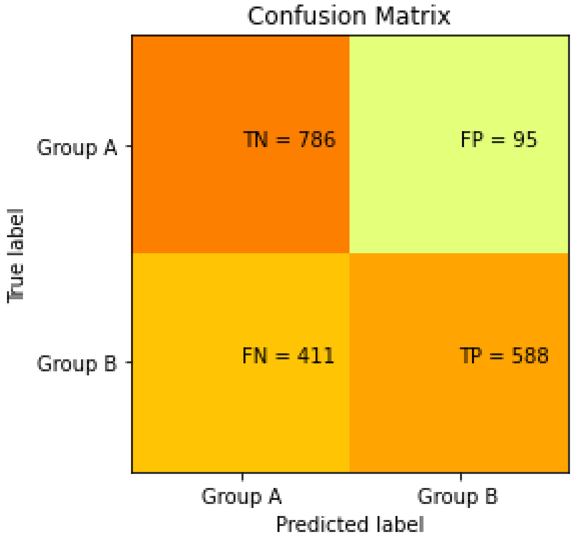

AUC Score : 0.7403782897539992

Accuracy Of the Model(ExtraTreesClassifier): 0.7308510638297873

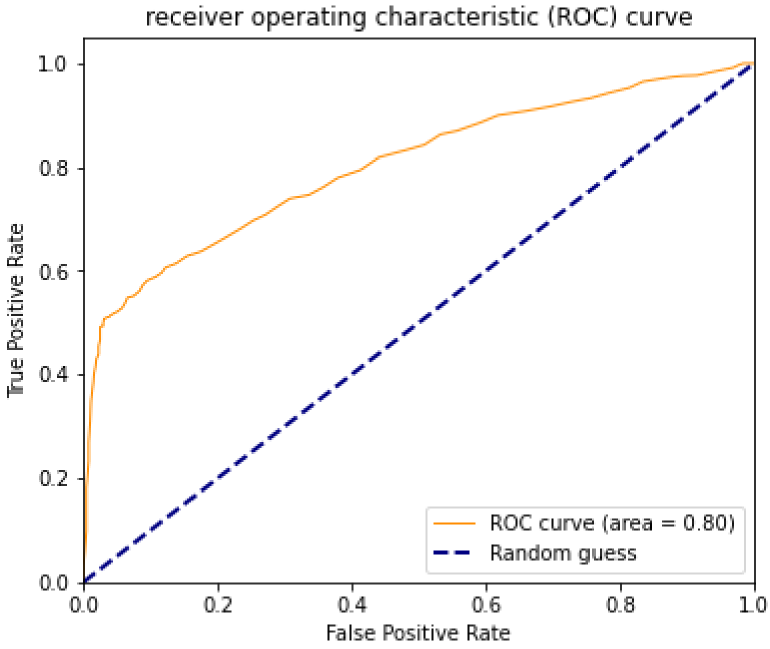

```
ROC_AUC Score of the model(ExtraTreesClassifier): 0.8033175059281756


Confusion Matrix:
[[788  93]
 [405 594]]

Classification Report:
              precision    recall  f1-score   support

         0.0       0.66      0.89      0.76       881
         1.0       0.86      0.59      0.70       999

    accuracy                           0.74      1880
   macro avg       0.76      0.74      0.73      1880
weighted avg       0.77      0.74      0.73      1880
```

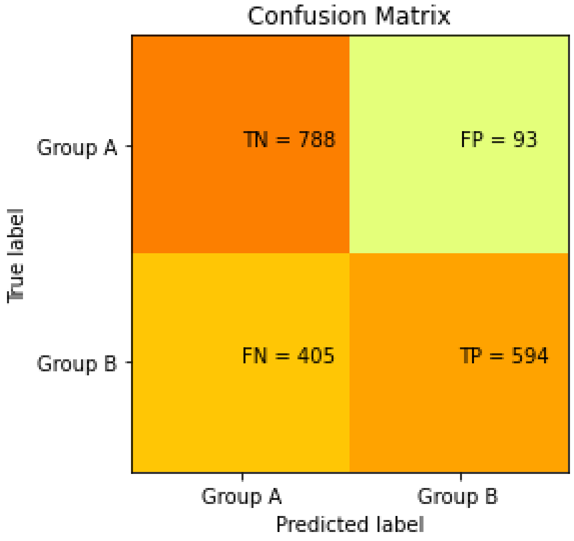


```
AUC Score : 0.7445163665367979

Accuracy Of the Model(GradientBoostingClassifier): 0.7351063829787234
```

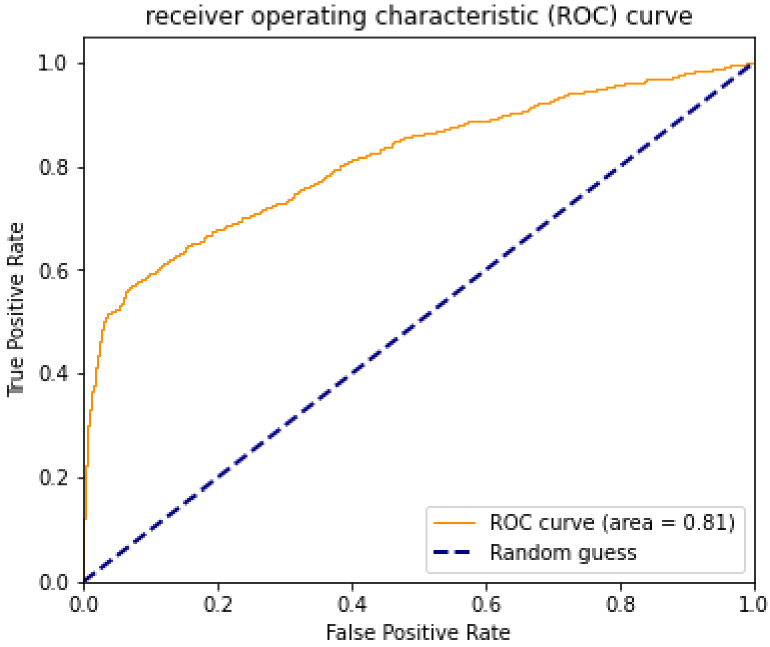

```
ROC_AUC Score of the model(GradientBoostingClassifier): 0.8132979744784512


Confusion Matrix:
[[820  61]
 [439 560]]

Classification Report:
              precision    recall  f1-score   support

         0.0       0.65      0.93      0.77       881
         1.0       0.90      0.56      0.69       999

    accuracy                           0.73      1880
   macro avg       0.78      0.75      0.73      1880
weighted avg       0.78      0.73      0.73      1880
```

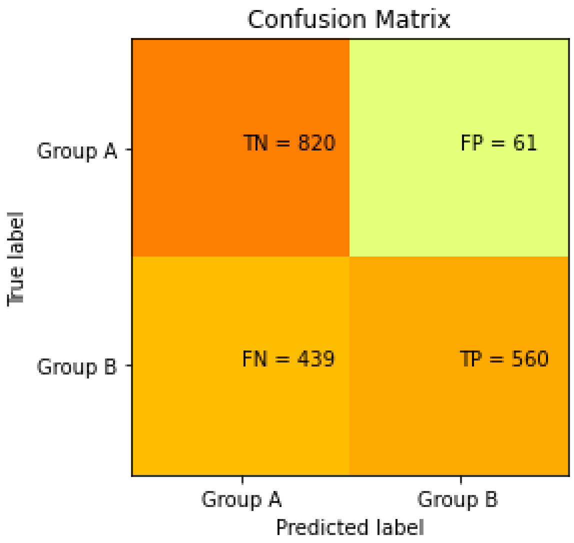


```
AUC Score : 0.7456605299965119

Accuracy Of the Model(AdaBoostClassifier): 0.7340425531914894
```

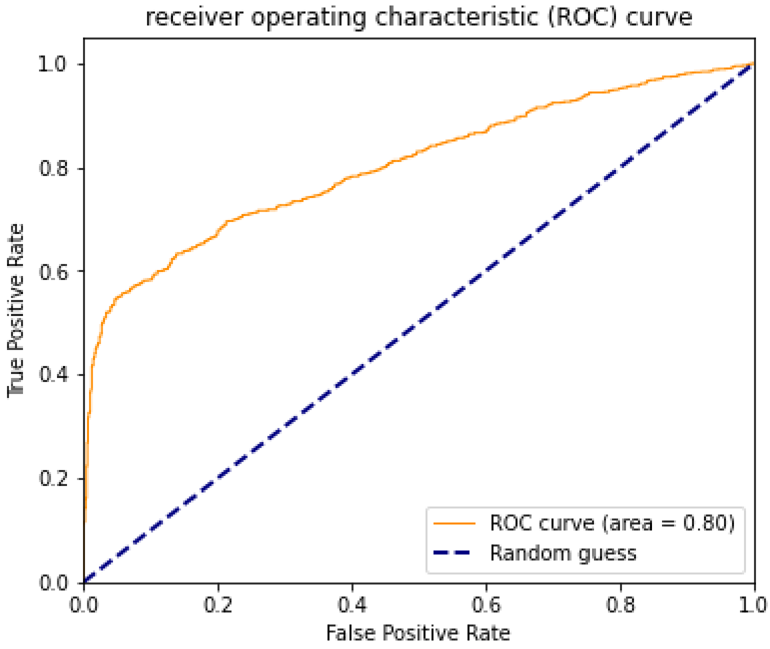

```
ROC_AUC Score of the model(AdaBoostClassifier): 0.8034209010372462


Confusion Matrix:
[[703 178]
 [346 653]]

Classification Report:
              precision    recall  f1-score   support

         0.0       0.67      0.80      0.73       881
         1.0       0.79      0.65      0.71       999

    accuracy                           0.72      1880
   macro avg       0.73      0.73      0.72      1880
weighted avg       0.73      0.72      0.72      1880
```

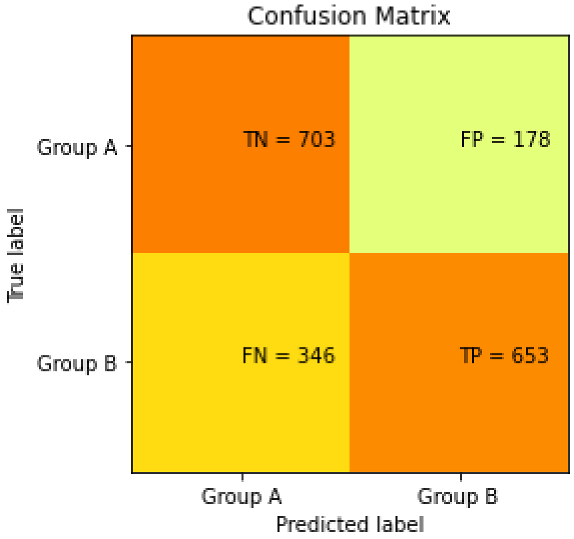


```
AUC Score : 0.7258052604250107

Accuracy Of the Model(SVC): 0.7212765957446808
```

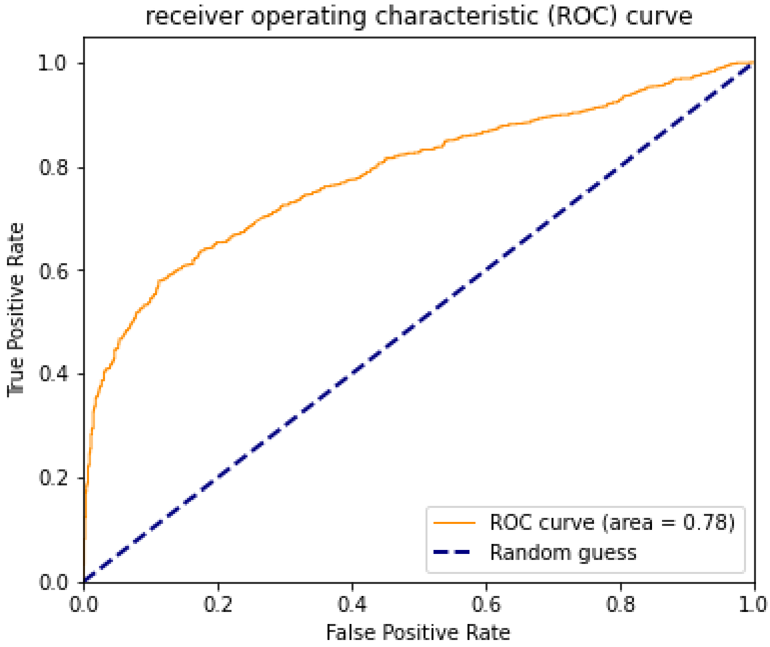

```
ROC_AUC Score of the model(SVC): 0.7848847712638859


Confusion Matrix:
[[797  84]
 [417 582]]

Classification Report:
              precision    recall  f1-score   support

         0.0       0.66      0.90      0.76       881
         1.0       0.87      0.58      0.70       999

    accuracy                           0.73      1880
   macro avg       0.77      0.74      0.73      1880
weighted avg       0.77      0.73      0.73      1880
```

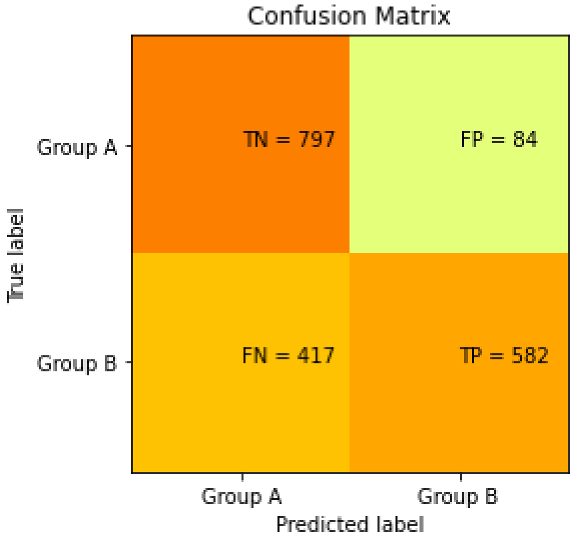


```
AUC Score : 0.7436181925398724

Accuracy Of the Model(MLPClassifier): 0.7335106382978723
```

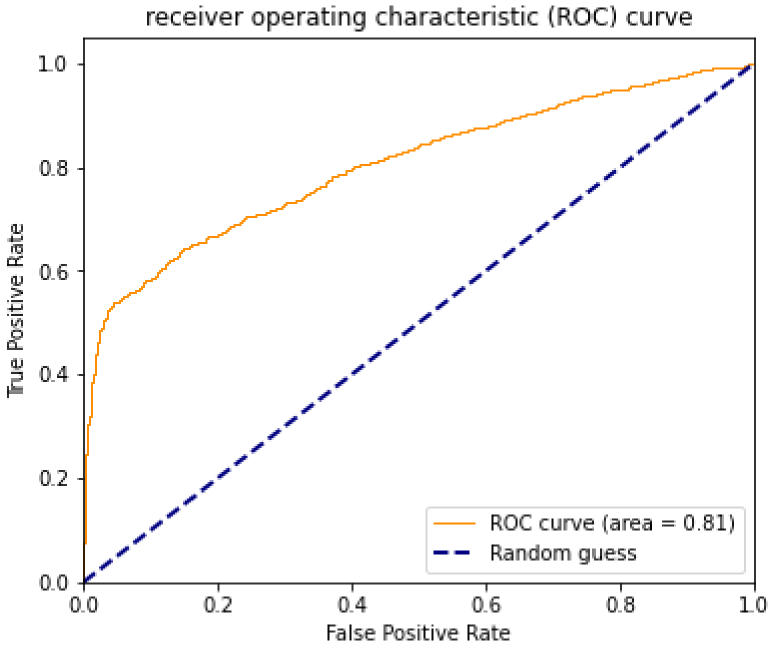

```
ROC_AUC Score of the model(MLPClassifier): 0.8064829869597179

[('RandomForestClassifier', 0.7388297872340426), ('XGBClassifier', 0.7388297872340
426), ('LogisticRegression', 0.7377659574468085), ('GradientBoostingClassifier',
0.7351063829787234), ('AdaBoostClassifier', 0.7340425531914894), ('MLPClassifier',
0.7335106382978723), ('ExtraTreesClassifier', 0.7308510638297873), ('DecisionTreeC
lassifier', 0.7223404255319149), ('SVC', 0.7212765957446808), ('KNeighborsClassifi
er', 0.7127659574468085)]


sorted_total_auc:
 [('GradientBoostingClassifier', 0.8132979744784512), ('XGBClassifier', 0.80969562
07058364), ('RandomForestClassifier', 0.8092621565947332), ('MLPClassifier', 0.806
4829869597179), ('AdaBoostClassifier', 0.8034209010372462), ('ExtraTreesClassifie
r', 0.8033175059281756), ('LogisticRegression', 0.8032538781687477), ('KNeighborsC
lassifier', 0.7965979600485843), ('SVC', 0.7848847712638859), ('DecisionTreeClassi
fier', 0.7821430965585336)]
```

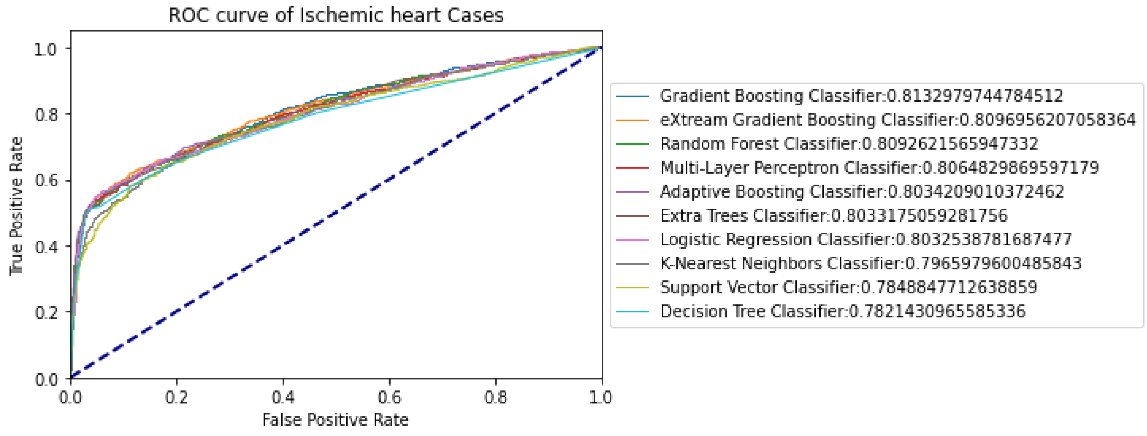

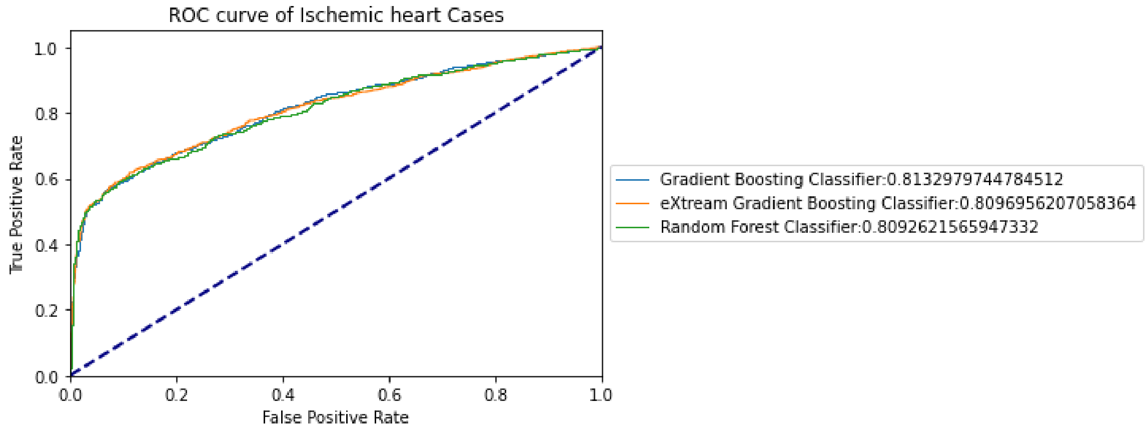

```
random state :   12411
9398
result_train_filename: data/train_test_data_ischemic heart_2013_20210519_122046.cs
v
total count : 9398, df_train count : 7518, df_test count : 1880
(7518, 38)
(1880, 38)
('Logistic Regression', LogisticRegression())
[2021-05-19 12:20:46.475727] Start parameter search for model 'Logistic Regressio
n'
[2021-05-19 12:20:46.824360] Finish parameter search for model 'Logistic Regressio
n' (time: 0 seconds)

=== train : best params ===
{'C': 0.1, 'penalty': 'l2', 'solver': 'lbfgs'}
=== train : best score : roc_auc ===
0.8049849133797079



('Random Forest', RandomForestClassifier())
[2021-05-19 12:20:46.824360] Start parameter search for model 'Random Forest'
[2021-05-19 12:20:51.896621] Finish parameter search for model 'Random Forest' (ti
me: 5 seconds)

=== train : best params ===
{'criterion': 'gini', 'max_depth': 8, 'max_features': 'auto', 'n_estimators': 100}
=== train : best score : roc_auc ===
0.8102999406580125



('Extreme Gradient Boosting)', XGBClassifier(base_score=None, booster=None, colsam
ple_bylevel=None,
              colsample_bynode=None, colsample_bytree=None, gamma=None,
              gpu_id=None, importance_type='gain', interaction_constraints=None,
              learning_rate=None, max_delta_step=None, max_depth=None,
              min_child_weight=None, missing=nan, monotone_constraints=None,
              n_estimators=100, n_jobs=None, num_parallel_tree=None,
              random_state=None, reg_alpha=None, reg_lambda=None,
              scale_pos_weight=None, subsample=None, tree_method=None,
              validate_parameters=None, verbosity=None))
[2021-05-19 12:20:51.896621] Start parameter search for model 'Extreme Gradient Bo
osting)'
[2021-05-19 12:21:19.915412] Finish parameter search for model 'Extreme Gradient B
oosting)' (time: 28 seconds)

=== train : best params ===
{'learning_rate': 0.075, 'max_depth': 5, 'n_estimators': 50, 'subsample': 0.7}
=== train : best score : roc_auc ===
0.8120306659066735



('k-Nearest Neighbours)', KNeighborsClassifier())
[2021-05-19 12:21:19.917407] Start parameter search for model 'k-Nearest Neighbour
s)'
[2021-05-19 12:21:27.823159] Finish parameter search for model 'k-Nearest Neighbou
rs)' (time: 7 seconds)

=== train : best params ===
{'n_neighbors': 60, 'weights': 'distance'}
=== train : best score : roc_auc ===
0.7916162923775372
```

```
('Decision Tree', DecisionTreeClassifier())
[2021-05-19 12:21:27.823159] Start parameter search for model 'Decision Tree'
[2021-05-19 12:21:28.413021] Finish parameter search for model 'Decision Tree' (ti
me: 0 seconds)

=== train : best params ===
{'criterion': 'entropy', 'max_depth': 5}
=== train : best score : roc_auc ===
0.7942205061488989


('Extra Tree', ExtraTreesClassifier())
[2021-05-19 12:21:28.413021] Start parameter search for model 'Extra Tree'
[2021-05-19 12:21:31.463213] Finish parameter search for model 'Extra Tree' (time:
3 seconds)

=== train : best params ===
{'max_features': 'auto', 'n_estimators': 100}
=== train : best score : roc_auc ===
0.8051512416857243


('Gradient Boosting', GradientBoostingClassifier())
[2021-05-19 12:21:31.464211] Start parameter search for model 'Gradient Boosting'
[2021-05-19 12:21:47.397862] Finish parameter search for model 'Gradient Boosting'
(time: 15 seconds)

=== train : best params ===
{'learning_rate': 0.01, 'max_depth': 8, 'max_features': 6, 'n_estimators': 100}
=== train : best score : roc_auc ===
0.8117732954394354


('Adaptive Boosting', AdaBoostClassifier())
[2021-05-19 12:21:47.398861] Start parameter search for model 'Adaptive Boosting'
[2021-05-19 12:21:50.543796] Finish parameter search for model 'Adaptive Boosting'
(time: 3 seconds)

=== train : best params ===
{'learning_rate': 0.2, 'n_estimators': 50}
=== train : best score : roc_auc ===
0.801771318637286


('Suppport Vector Classfier', SVC(probability=True))
[2021-05-19 12:21:50.544793] Start parameter search for model 'Suppport Vector Cla
ssfier'
[2021-05-19 12:25:33.684624] Finish parameter search for model 'Suppport Vector Cl
assfier' (time: 223 seconds)

=== train : best params ===
{'C': 1, 'gamma': 0.1, 'kernel': 'rbf'}
=== train : best score : roc_auc ===
0.7878740339782728


('Neural Network', MLPClassifier())
[2021-05-19 12:25:33.685625] Start parameter search for model 'Neural Network'
[2021-05-19 12:28:05.635944] Finish parameter search for model 'Neural Network' (t
```

```
ime: 151 seconds)

=== train : best params ===
{'activation': 'logistic', 'alpha': 0.05, 'hidden_layer_sizes': (50,), 'learning_r
ate': 'adaptive'}
=== train : best score : roc_auc ===
0.8090327480734528



Confusion Matrix:
[[808  73]
 [425 574]]

Classification Report:
              precision    recall  f1-score   support

         0.0       0.66      0.92      0.76       881
         1.0       0.89      0.57      0.70       999

    accuracy                           0.74      1880
   macro avg       0.77      0.75      0.73      1880
weighted avg       0.78      0.74      0.73      1880
```

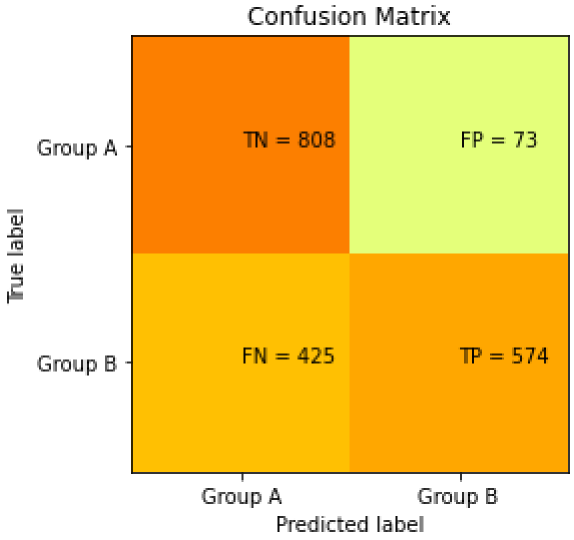


```
AUC Score : 0.7458570943247447

Accuracy Of the Model(LogisticRegression): 0.7351063829787234
```

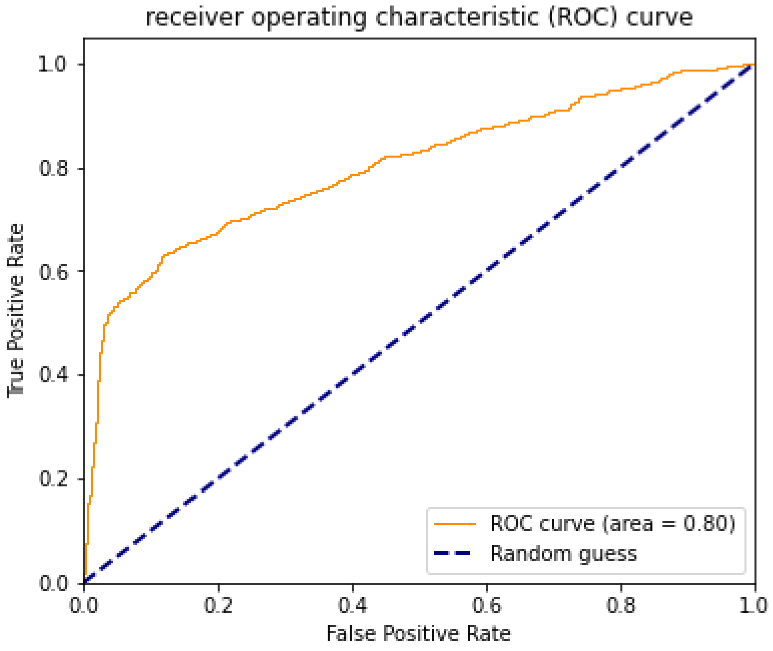


```
ROC_AUC Score of the model(LogisticRegression): 0.8029891412411275


Confusion Matrix:
[[786  95]
 [400 599]]

Classification Report:
              precision    recall  f1-score   support

         0.0       0.66      0.89      0.76       881
         1.0       0.86      0.60      0.71       999

    accuracy                           0.74      1880
   macro avg       0.76      0.75      0.73      1880
weighted avg       0.77      0.74      0.73      1880
```

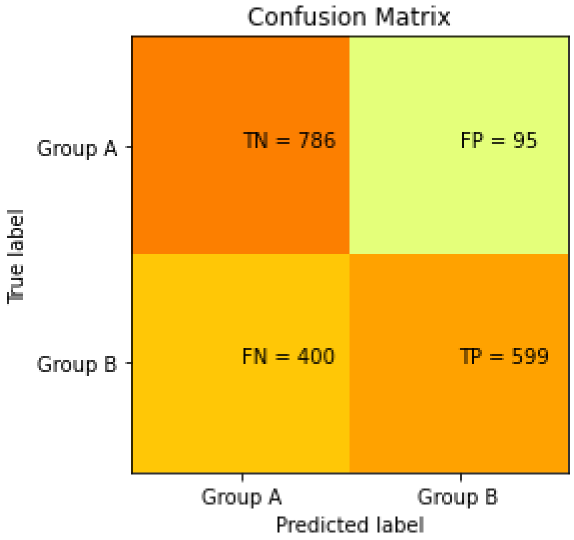


```
AUC Score : 0.7458837952595048

Accuracy Of the Model(RandomForestClassifier): 0.7367021276595744
```

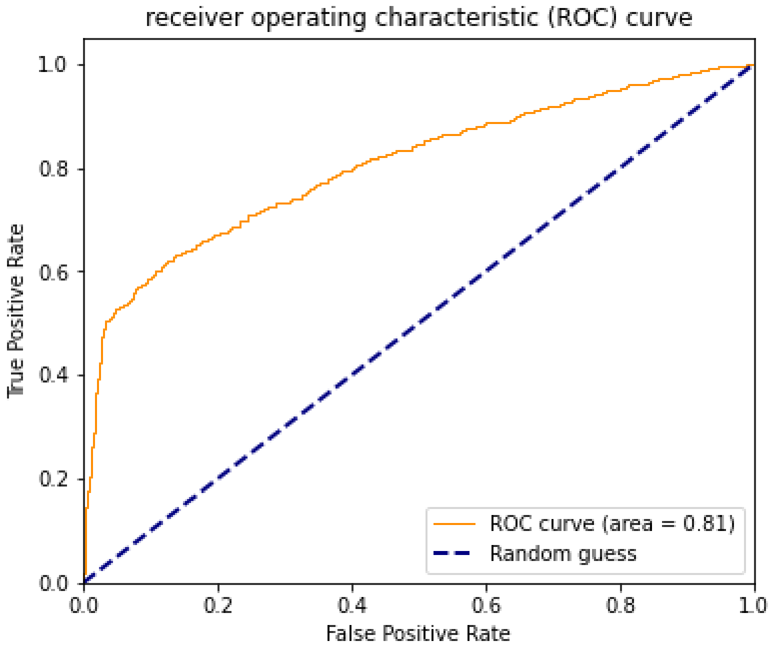

```
ROC_AUC Score of the model(RandomForestClassifier): 0.8061375791228232


Confusion Matrix:
[[793  88]
 [403 596]]

Classification Report:
              precision    recall  f1-score   support

         0.0       0.66      0.90      0.76       881
         1.0       0.87      0.60      0.71       999

    accuracy                           0.74      1880
   macro avg       0.77      0.75      0.74      1880
weighted avg       0.77      0.74      0.73      1880
```

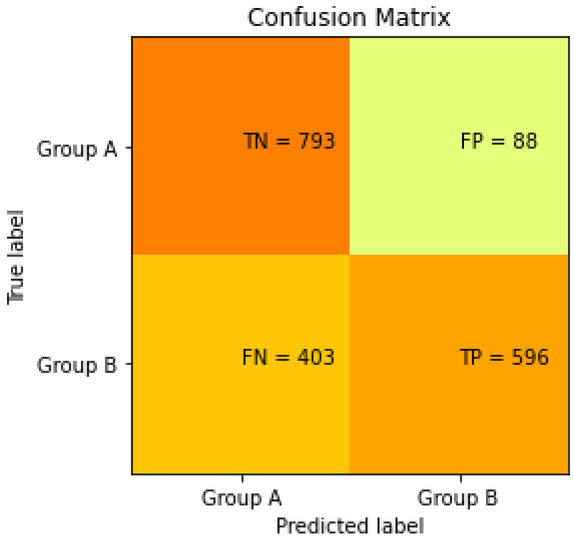


```
AUC Score : 0.748355051987288

Accuracy Of the Model(XGBClassifier): 0.7388297872340426
```

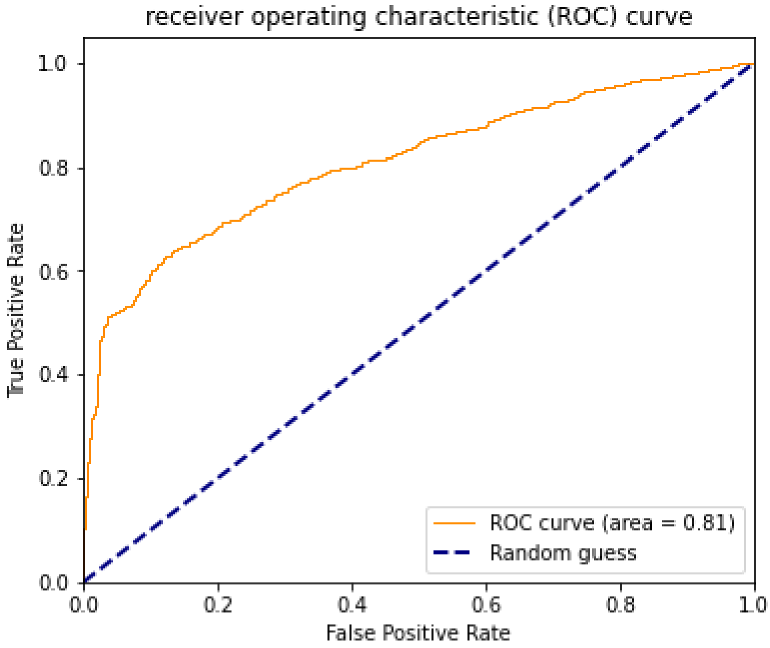

```
ROC_AUC Score of the model(XGBClassifier): 0.8108108108108109


Confusion Matrix:
[[786  95]
 [463 536]]

Classification Report:
              precision    recall  f1-score   support

         0.0       0.63      0.89      0.74       881
         1.0       0.85      0.54      0.66       999

    accuracy                           0.70      1880
   macro avg       0.74      0.71      0.70      1880
weighted avg       0.75      0.70      0.70      1880
```

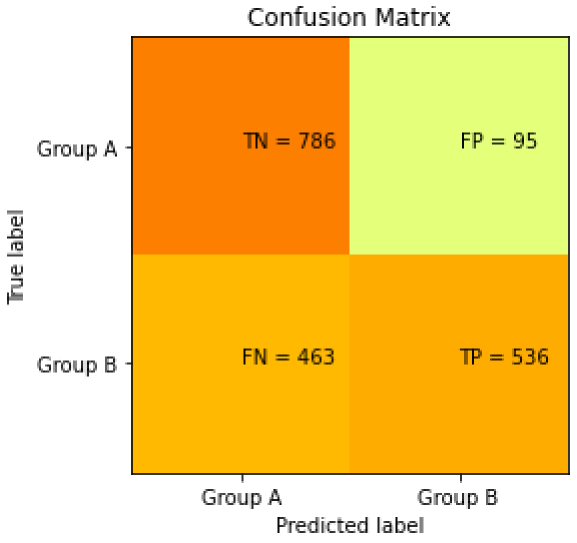


```
AUC Score : 0.7143522637279731

Accuracy Of the Model(KNeighborsClassifier): 0.7031914893617022
```

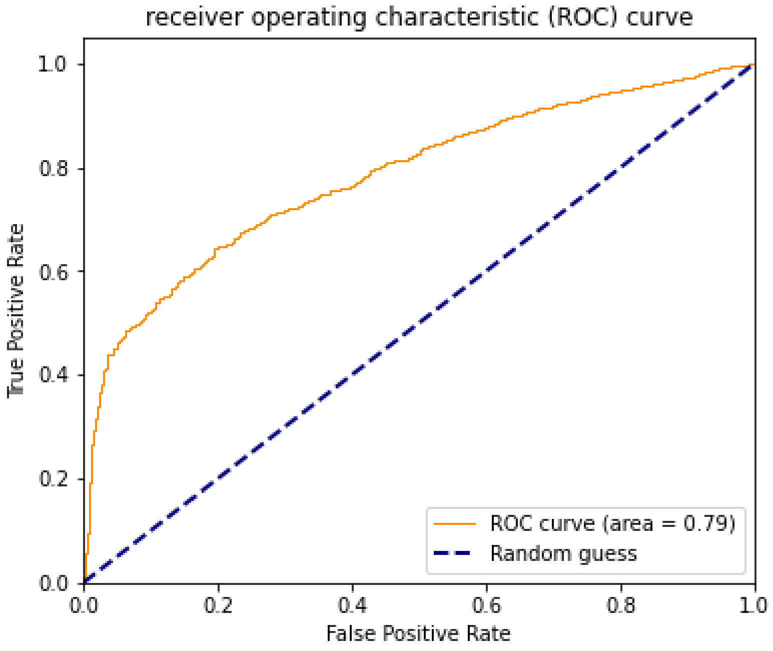

```
ROC_AUC Score of the model(KNeighborsClassifier): 0.7853903847093404


Confusion Matrix:
[[792  89]
 [413 586]]

Classification Report:
              precision    recall  f1-score   support

         0.0       0.66      0.90      0.76       881
         1.0       0.87      0.59      0.70       999

    accuracy                           0.73      1880
   macro avg       0.76      0.74      0.73      1880
weighted avg       0.77      0.73      0.73      1880
```

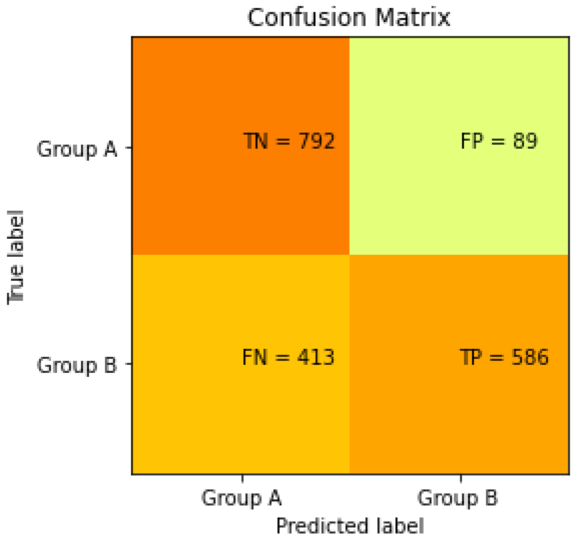


```
AUC Score : 0.7427825100923852

Accuracy Of the Model(DecisionTreeClassifier): 0.7329787234042553
```

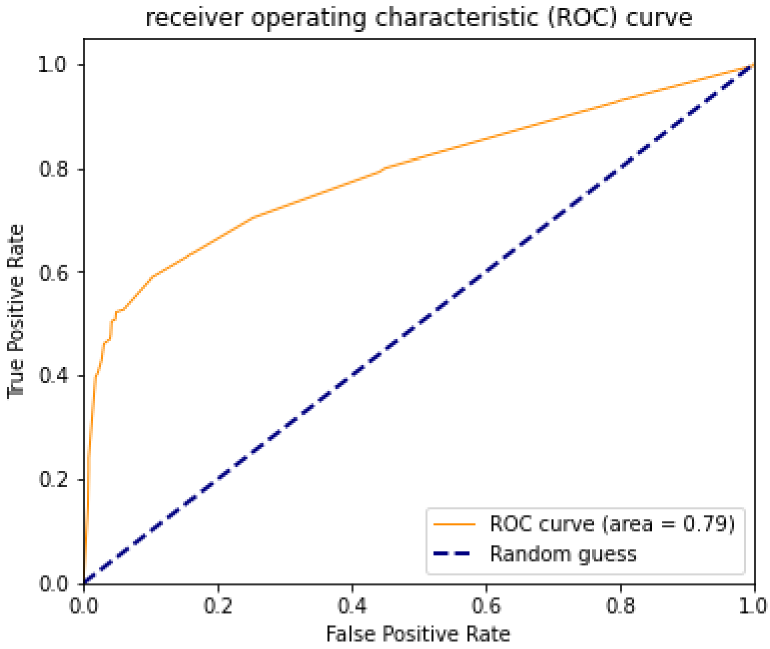

```
ROC_AUC Score of the model(DecisionTreeClassifier): 0.7880320729355916


Confusion Matrix:
[[769 112]
 [412 587]]

Classification Report:
              precision    recall  f1-score   support

         0.0       0.65      0.87      0.75       881
         1.0       0.84      0.59      0.69       999

    accuracy                           0.72      1880
   macro avg       0.75      0.73      0.72      1880
weighted avg       0.75      0.72      0.72      1880
```

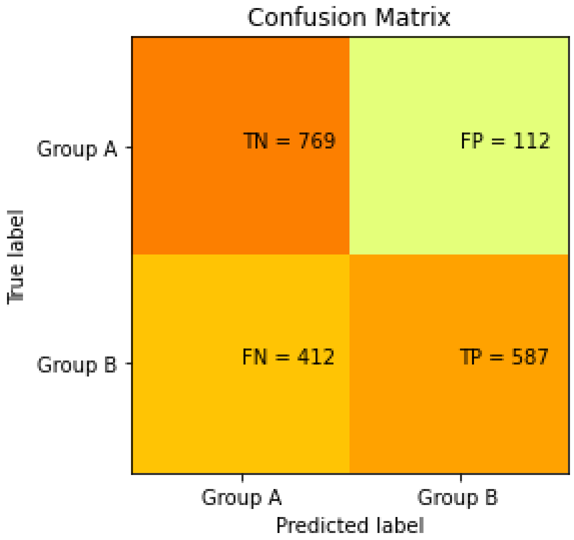


```
AUC Score : 0.7302296621252353

Accuracy Of the Model(ExtraTreesClassifier): 0.7212765957446808
```

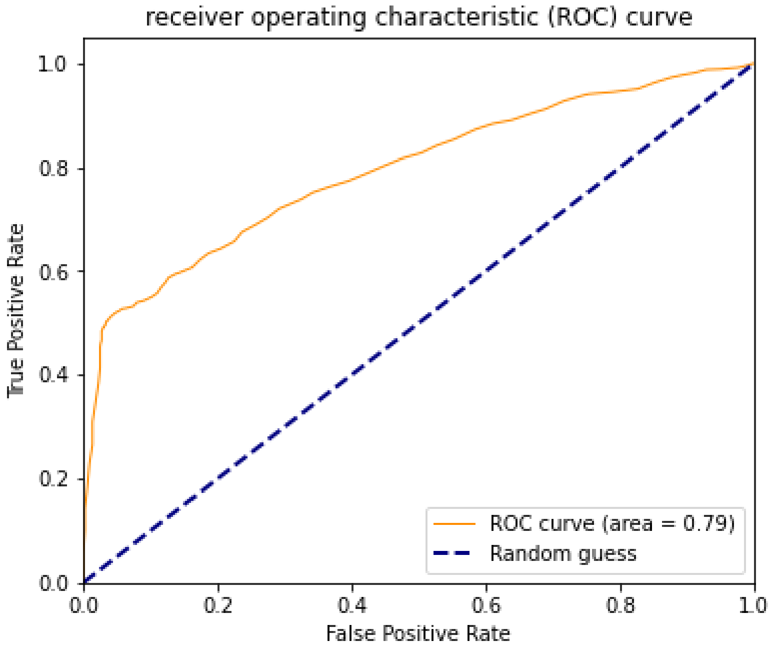

```
ROC_AUC Score of the model(ExtraTreesClassifier): 0.7938114050486356


Confusion Matrix:
[[791  90]
 [404 595]]

Classification Report:
              precision    recall  f1-score   support

         0.0       0.66      0.90      0.76       881
         1.0       0.87      0.60      0.71       999

    accuracy                           0.74      1880
   macro avg       0.77      0.75      0.73      1880
weighted avg       0.77      0.74      0.73      1880
```

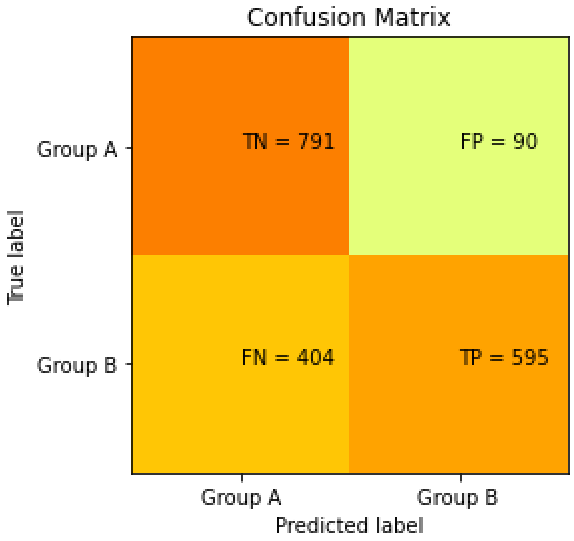


```
AUC Score : 0.7467194777069919

Accuracy Of the Model(GradientBoostingClassifier): 0.7372340425531915
```

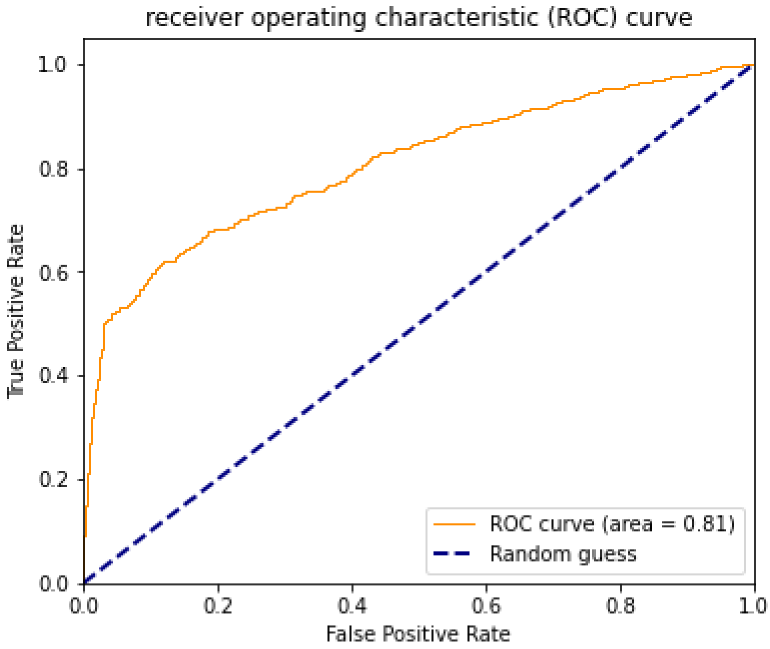

ROC_AUC Score of the model(GradientBoostingClassifier): 0.8079361995366535

```
Confusion Matrix:
[[818  63]
 [452 547]]

Classification Report:
              precision    recall  f1-score   support

         0.0       0.64      0.93      0.76       881
         1.0       0.90      0.55      0.68       999

    accuracy                           0.73      1880
   macro avg       0.77      0.74      0.72      1880
weighted avg       0.78      0.73      0.72      1880
```

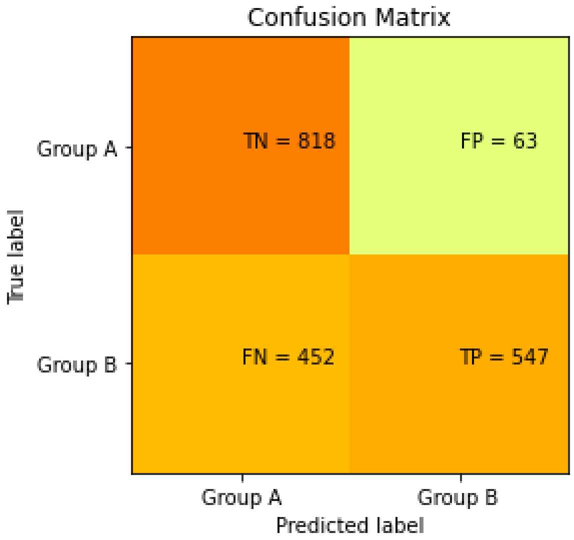

AUC Score : 0.7380189497102096

Accuracy Of the Model(AdaBoostClassifier): 0.726063829787234

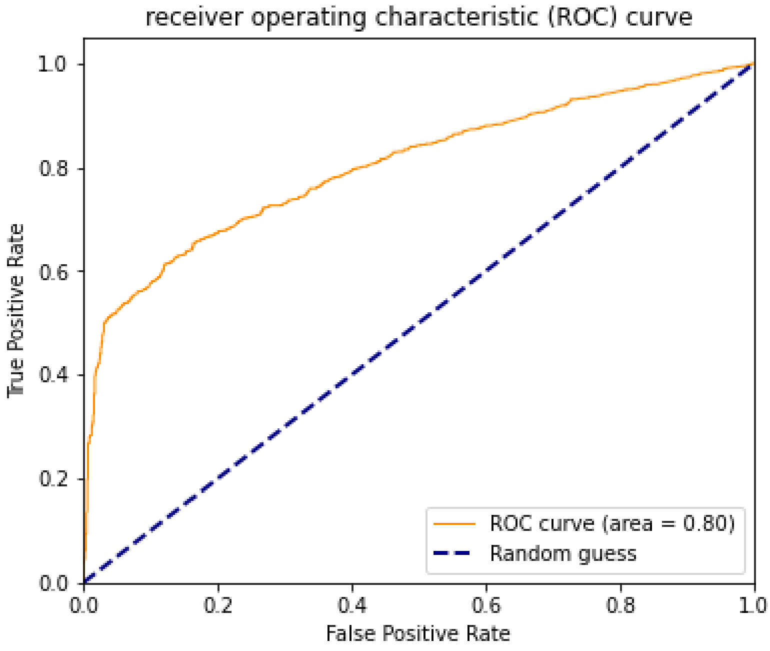

```
ROC_AUC Score of the model(AdaBoostClassifier): 0.8021523225836507


Confusion Matrix:
[[690 191]
 [350 649]]

Classification Report:
              precision    recall  f1-score   support

         0.0       0.66      0.78      0.72       881
         1.0       0.77      0.65      0.71       999

    accuracy                           0.71      1880
   macro avg       0.72      0.72      0.71      1880
weighted avg       0.72      0.71      0.71      1880
```

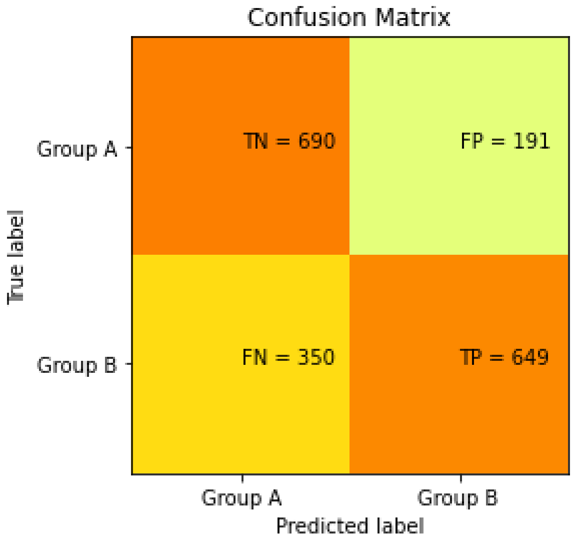


```
AUC Score : 0.7164252788543368

Accuracy Of the Model(SVC): 0.7122340425531914
```

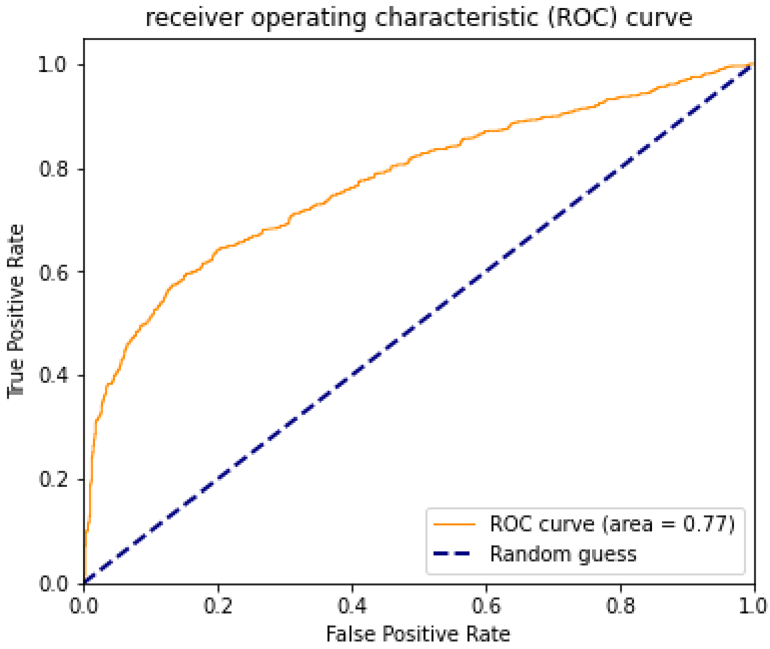

ROC_AUC Score of the model(SVC): 0.7732102136188402

Confusion Matrix:
[[779 102]
 [405 594]]

Classification Report:

	precision	recall	f1-score	support
0.0	0.66	0.88	0.75	881
1.0	0.85	0.59	0.70	999
accuracy			0.73	1880
macro avg	0.76	0.74	0.73	1880
weighted avg	0.76	0.73	0.73	1880

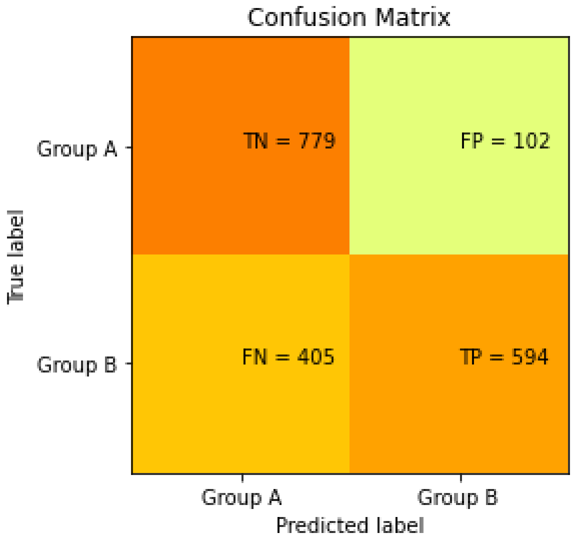

AUC Score : 0.7394085345277174

Accuracy Of the Model(MLPClassifier): 0.7303191489361702

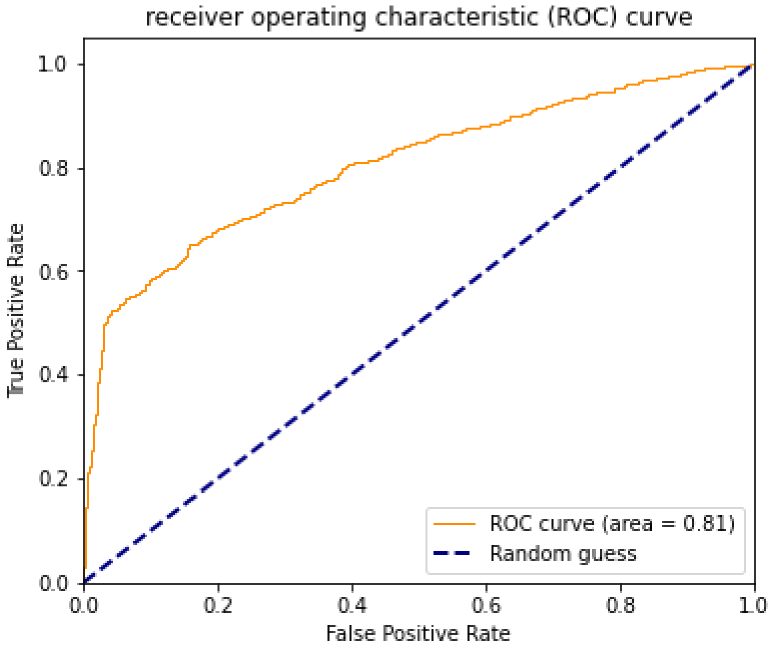

```
ROC_AUC Score of the model(MLPClassifier): 0.8062807415815361

[('XGBClassifier', 0.7388297872340426), ('GradientBoostingClassifier', 0.737234042
5531915), ('RandomForestClassifier', 0.7367021276595744), ('LogisticRegression',
0.7351063829787234), ('DecisionTreeClassifier', 0.7329787234042553), ('MLPClassifi
er', 0.7303191489361702), ('AdaBoostClassifier', 0.726063829787234), ('ExtraTreesC
lassifier', 0.7212765957446808), ('SVC', 0.7122340425531914), ('KNeighborsClassifi
er', 0.7031914893617022)]


sorted_total_auc:
 [('XGBClassifier', 0.8108108108108109), ('GradientBoostingClassifier', 0.80793619
95366535), ('MLPClassifier', 0.8062807415815361), ('RandomForestClassifier', 0.806
1375791228232), ('LogisticRegression', 0.8029891412411275), ('AdaBoostClassifier',
0.8021523225836507), ('ExtraTreesClassifier', 0.7938114050486356), ('DecisionTreeC
lassifier', 0.7880320729355916), ('KNeighborsClassifier', 0.7853903847093404), ('S
VC', 0.7732102136188402)]
```

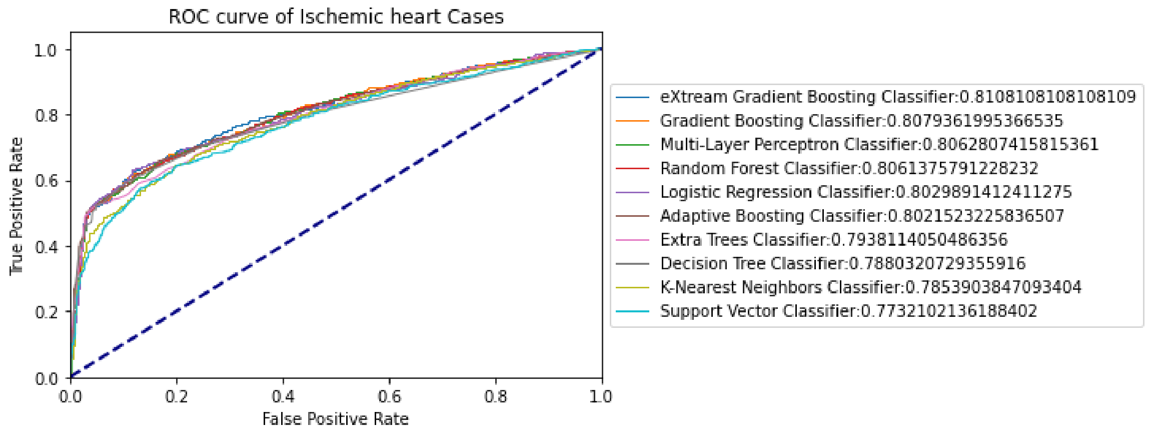

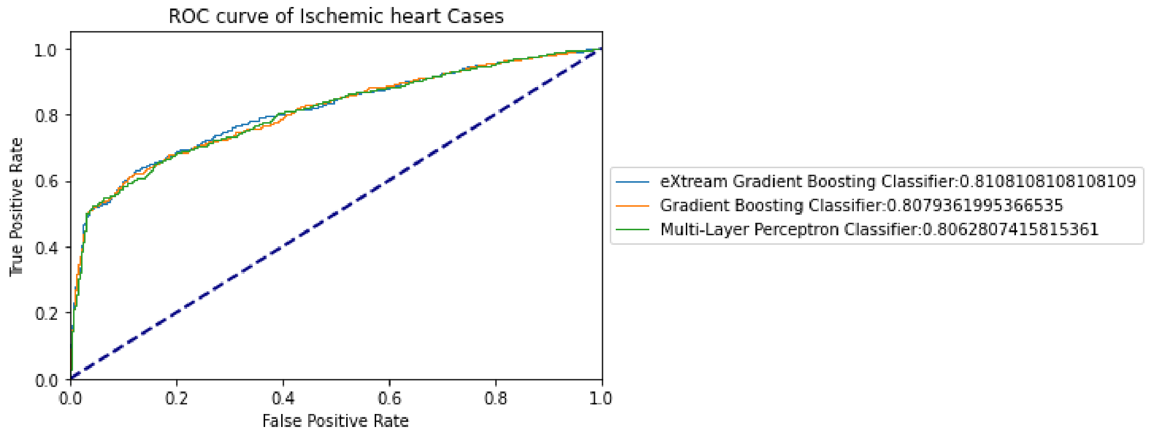

```
random state :   12423
9398
result_train_filename: data/train_test_data_ischemic heart_2013_20210519_122813.cs
v
total count : 9398, df_train count : 7518, df_test count : 1880
(7518, 38)
(1880, 38)
('Logistic Regression', LogisticRegression())
[2021-05-19 12:28:14.094042] Start parameter search for model 'Logistic Regressio
n'
[2021-05-19 12:28:14.448503] Finish parameter search for model 'Logistic Regressio
n' (time: 0 seconds)

=== train : best params ===
{'C': 0.1, 'penalty': 'l2', 'solver': 'lbfgs'}
=== train : best score : roc_auc ===
0.8049649740821501


('Random Forest', RandomForestClassifier())
[2021-05-19 12:28:14.448503] Start parameter search for model 'Random Forest'
[2021-05-19 12:28:20.511013] Finish parameter search for model 'Random Forest' (ti
me: 6 seconds)

=== train : best params ===
{'criterion': 'gini', 'max_depth': 8, 'max_features': 'auto', 'n_estimators': 90}
=== train : best score : roc_auc ===
0.8116766109833261


('Extreme Gradient Boosting)', XGBClassifier(base_score=None, booster=None, colsam
ple_bylevel=None,
              colsample_bynode=None, colsample_bytree=None, gamma=None,
              gpu_id=None, importance_type='gain', interaction_constraints=None,
              learning_rate=None, max_delta_step=None, max_depth=None,
              min_child_weight=None, missing=nan, monotone_constraints=None,
              n_estimators=100, n_jobs=None, num_parallel_tree=None,
              random_state=None, reg_alpha=None, reg_lambda=None,
              scale_pos_weight=None, subsample=None, tree_method=None,
              validate_parameters=None, verbosity=None))
[2021-05-19 12:28:20.511013] Start parameter search for model 'Extreme Gradient Bo
osting)'
[2021-05-19 12:28:53.688475] Finish parameter search for model 'Extreme Gradient B
oosting)' (time: 33 seconds)

=== train : best params ===
{'learning_rate': 0.075, 'max_depth': 5, 'n_estimators': 40, 'subsample': 0.7}
=== train : best score : roc_auc ===
0.8120787077772228


('k-Nearest Neighbours)', KNeighborsClassifier())
[2021-05-19 12:28:53.689472] Start parameter search for model 'k-Nearest Neighbour
s)'
[2021-05-19 12:29:01.921214] Finish parameter search for model 'k-Nearest Neighbou
rs)' (time: 8 seconds)

=== train : best params ===
{'n_neighbors': 60, 'weights': 'distance'}
=== train : best score : roc_auc ===
0.7910867771758351
```

```
('Decision Tree', DecisionTreeClassifier())
[2021-05-19 12:29:01.922211] Start parameter search for model 'Decision Tree'
[2021-05-19 12:29:02.572061] Finish parameter search for model 'Decision Tree' (ti
me: 0 seconds)

=== train : best params ===
{'criterion': 'entropy', 'max_depth': 4}
=== train : best score : roc_auc ===
0.7951480701525643


('Extra Tree', ExtraTreesClassifier())
[2021-05-19 12:29:02.573057] Start parameter search for model 'Extra Tree'
[2021-05-19 12:29:05.856729] Finish parameter search for model 'Extra Tree' (time:
3 seconds)

=== train : best params ===
{'max_features': 'sqrt', 'n_estimators': 100}
=== train : best score : roc_auc ===
0.8038630651533564


('Gradient Boosting', GradientBoostingClassifier())
[2021-05-19 12:29:05.857724] Start parameter search for model 'Gradient Boosting'
[2021-05-19 12:29:23.122465] Finish parameter search for model 'Gradient Boosting'
(time: 17 seconds)

=== train : best params ===
{'learning_rate': 0.01, 'max_depth': 8, 'max_features': 4, 'n_estimators': 100}
=== train : best score : roc_auc ===
0.8123925827029915


('Adaptive Boosting', AdaBoostClassifier())
[2021-05-19 12:29:23.123455] Start parameter search for model 'Adaptive Boosting'
[2021-05-19 12:29:26.866003] Finish parameter search for model 'Adaptive Boosting'
(time: 3 seconds)

=== train : best params ===
{'learning_rate': 0.2, 'n_estimators': 100}
=== train : best score : roc_auc ===
0.8052003500588898


('Suppport Vector Classfier', SVC(probability=True))
[2021-05-19 12:29:26.867000] Start parameter search for model 'Suppport Vector Cla
ssfier'
[2021-05-19 12:33:34.756410] Finish parameter search for model 'Suppport Vector Cl
assfier' (time: 247 seconds)

=== train : best params ===
{'C': 1, 'gamma': 0.1, 'kernel': 'rbf'}
=== train : best score : roc_auc ===
0.7865132098641778


('Neural Network', MLPClassifier())
[2021-05-19 12:33:34.757411] Start parameter search for model 'Neural Network'
[2021-05-19 12:36:08.945989] Finish parameter search for model 'Neural Network' (t
```

```
ime: 154 seconds)

=== train : best params ===
{'activation': 'logistic', 'alpha': 0.05, 'hidden_layer_sizes': (50,), 'learning_r
ate': 'adaptive'}
=== train : best score : roc_auc ===
0.8108015654230086



Confusion Matrix:
[[819  62]
 [426 573]]

Classification Report:
              precision    recall  f1-score   support

         0.0       0.66      0.93      0.77       881
         1.0       0.90      0.57      0.70       999

    accuracy                           0.74      1880
   macro avg       0.78      0.75      0.74      1880
weighted avg       0.79      0.74      0.73      1880
```

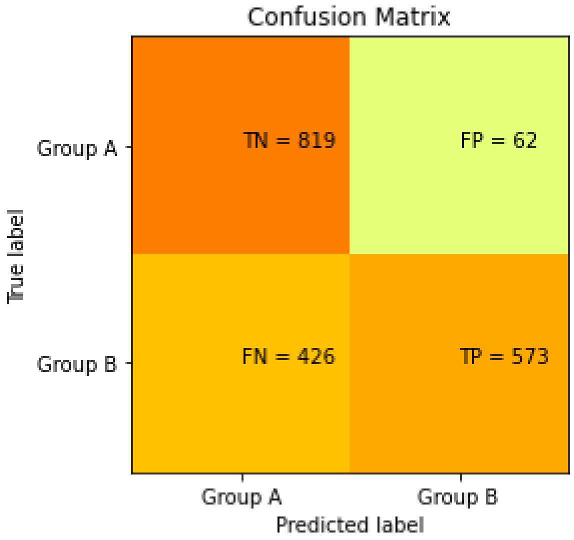


```
AUC Score : 0.7515994996131206

Accuracy Of the Model(LogisticRegression): 0.7404255319148936
```

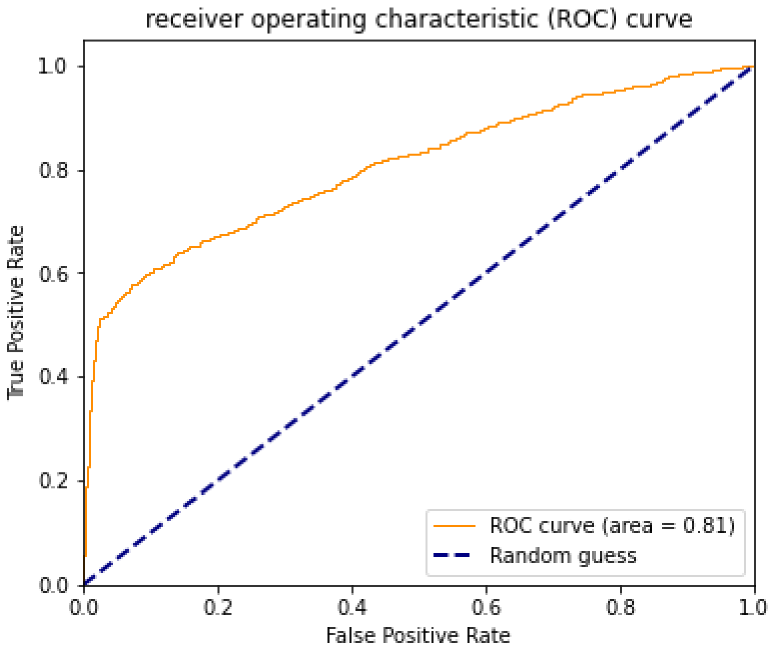


```
ROC_AUC Score of the model(LogisticRegression): 0.8071522146437016


Confusion Matrix:
[[791  90]
 [401 598]]

Classification Report:
              precision    recall  f1-score   support

         0.0       0.66      0.90      0.76       881
         1.0       0.87      0.60      0.71       999

    accuracy                           0.74      1880
   macro avg       0.77      0.75      0.74      1880
weighted avg       0.77      0.74      0.73      1880
```

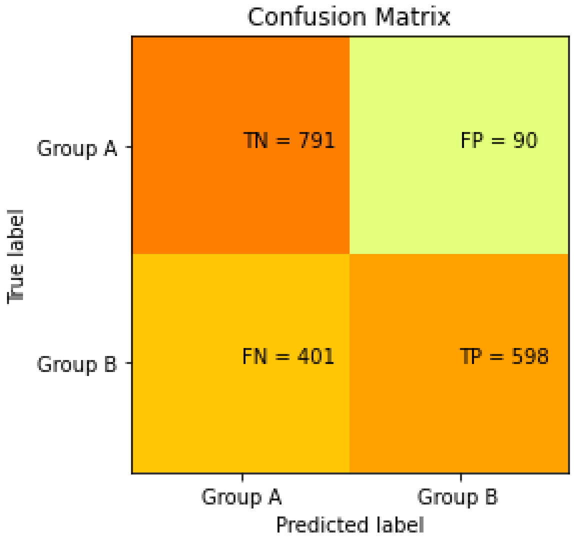


```
AUC Score : 0.7482209792084934

Accuracy Of the Model(RandomForestClassifier): 0.7388297872340426
```

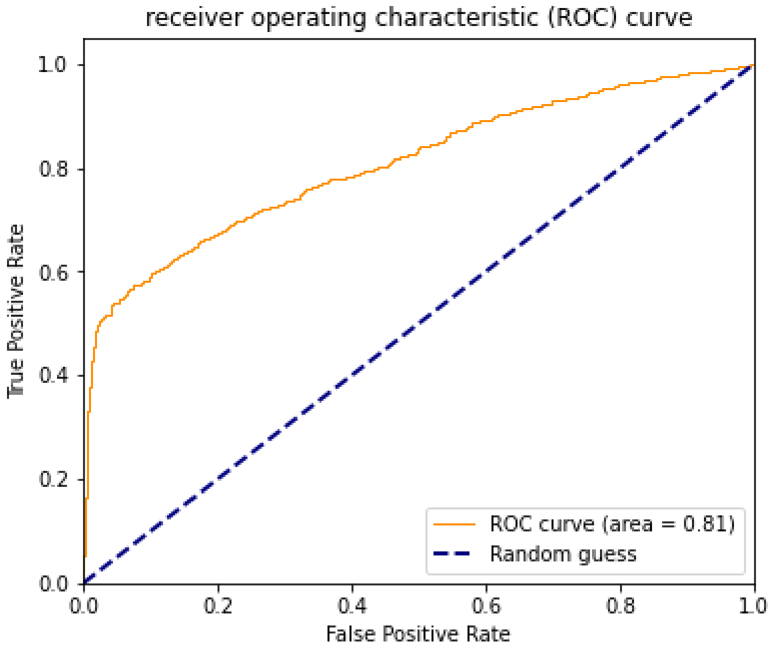

```
ROC_AUC Score of the model(RandomForestClassifier): 0.8097473182603716


Confusion Matrix:
[[804  77]
 [409 590]]

Classification Report:
              precision    recall  f1-score   support

         0.0       0.66      0.91      0.77       881
         1.0       0.88      0.59      0.71       999

    accuracy                           0.74      1880
   macro avg       0.77      0.75      0.74      1880
weighted avg       0.78      0.74      0.74      1880
```

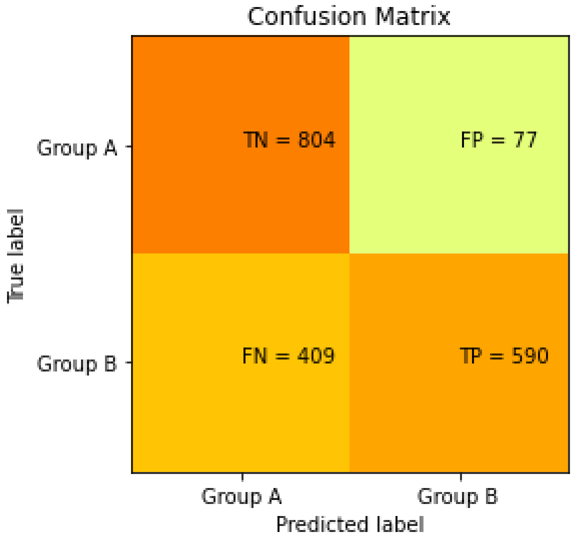


```
AUC Score : 0.7515949547731613

Accuracy Of the Model(XGBClassifier): 0.7414893617021276
```

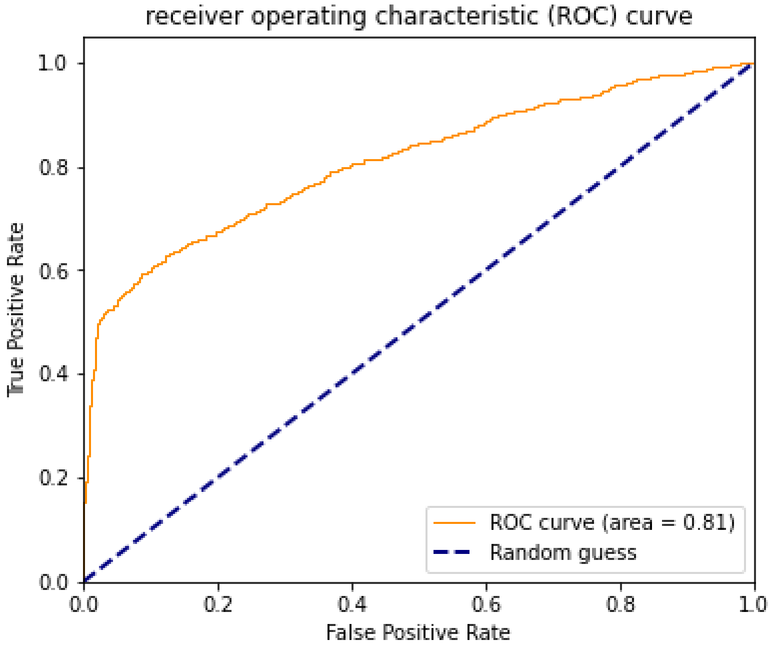

ROC_AUC Score of the model(XGBClassifier): 0.8118572602114031

```
Confusion Matrix:
[[790  91]
 [452 547]]

Classification Report:
              precision    recall  f1-score   support

         0.0       0.64      0.90      0.74       881
         1.0       0.86      0.55      0.67       999

    accuracy                           0.71      1880
   macro avg       0.75      0.72      0.71      1880
weighted avg       0.75      0.71      0.70      1880
```

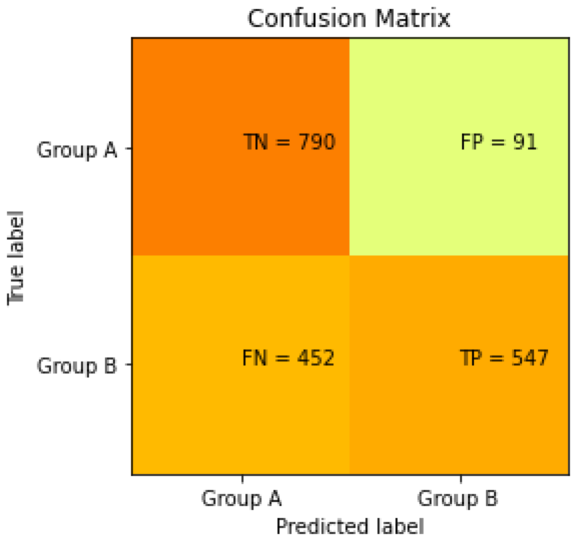

AUC Score : 0.7221279167930701

Accuracy Of the Model(KNeighborsClassifier): 0.7111702127659575

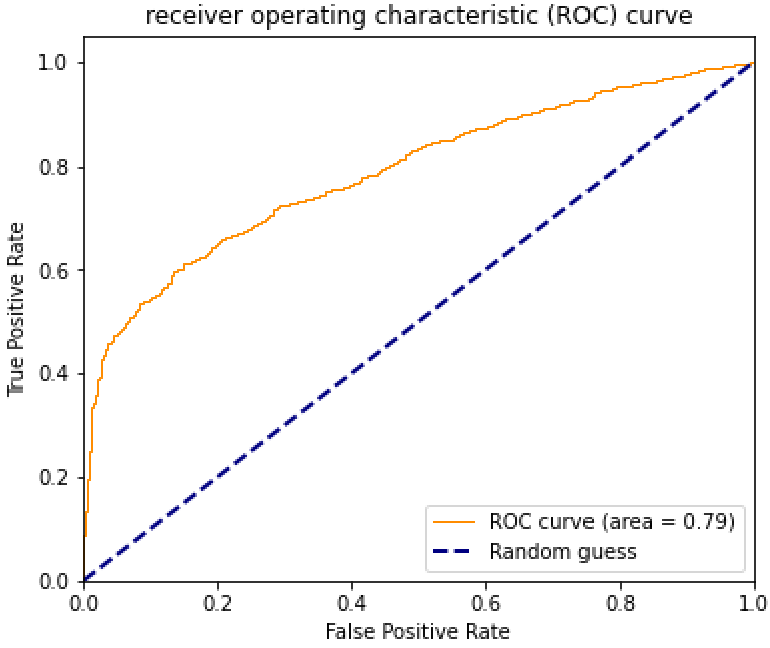

```
ROC_AUC Score of the model(KNeighborsClassifier): 0.7899965800079307


Confusion Matrix:
[[791  90]
 [425 574]]

Classification Report:
              precision    recall  f1-score   support

         0.0       0.65      0.90      0.75       881
         1.0       0.86      0.57      0.69       999

    accuracy                           0.73      1880
   macro avg       0.76      0.74      0.72      1880
weighted avg       0.76      0.73      0.72      1880
```

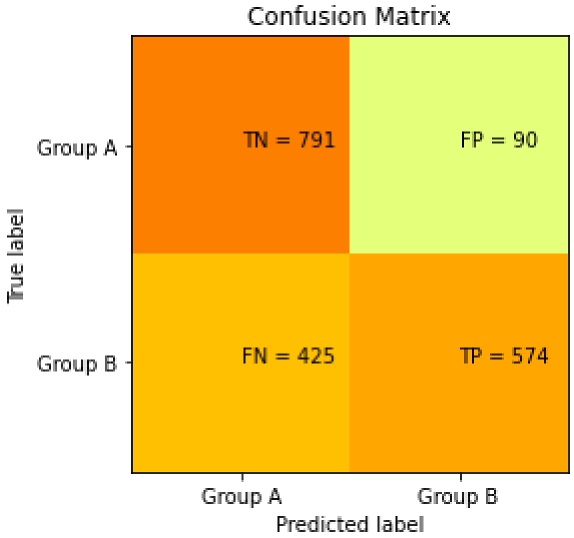


```
AUC Score : 0.7362089671964814

Accuracy Of the Model(DecisionTreeClassifier): 0.726063829787234
```

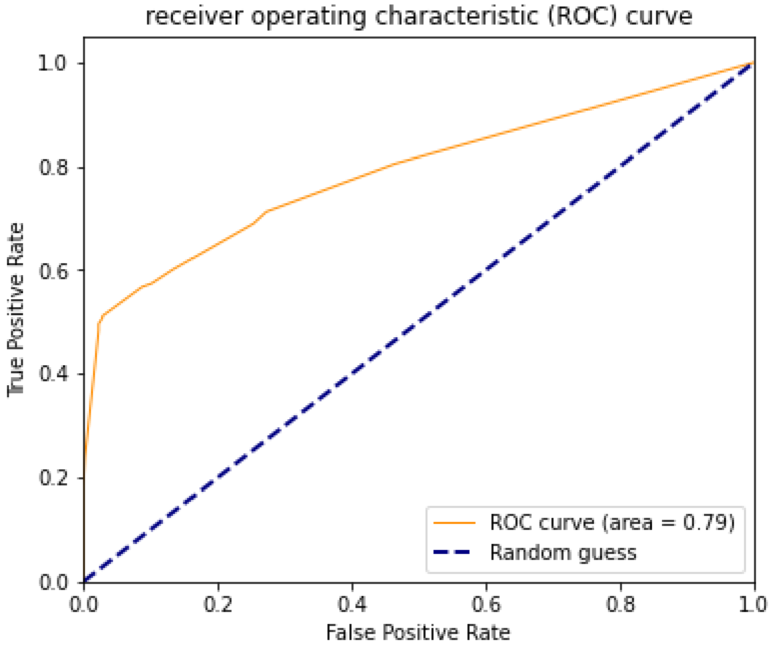

```
ROC_AUC Score of the model(DecisionTreeClassifier): 0.7891540803005047


Confusion Matrix:
[[776 105]
 [424 575]]

Classification Report:
              precision    recall  f1-score   support

         0.0       0.65      0.88      0.75       881
         1.0       0.85      0.58      0.68       999

    accuracy                           0.72      1880
   macro avg       0.75      0.73      0.72      1880
weighted avg       0.75      0.72      0.71      1880
```

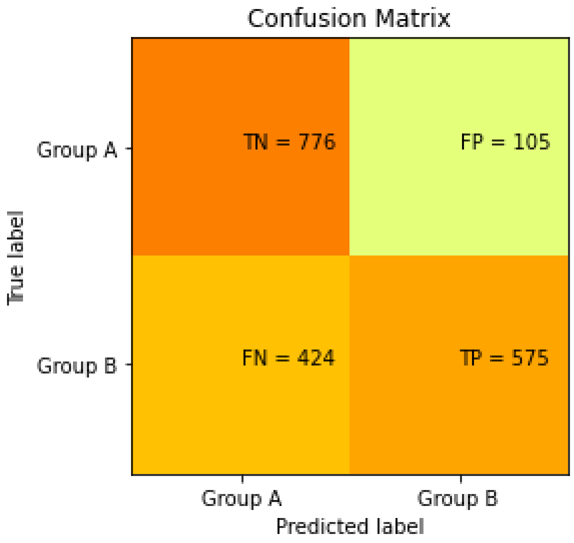


```
AUC Score : 0.7281964143485142

Accuracy Of the Model(ExtraTreesClassifier): 0.7186170212765958
```

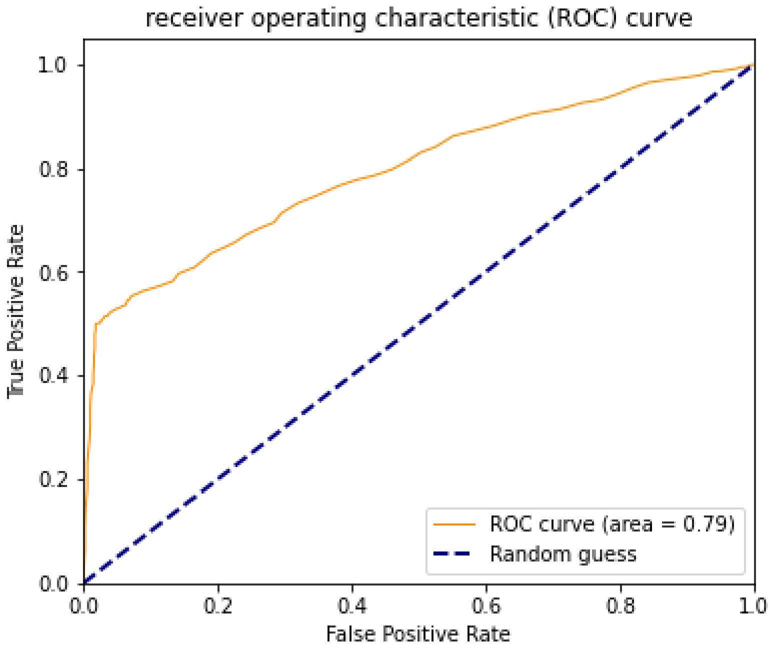

```
ROC_AUC Score of the model(ExtraTreesClassifier): 0.7940596669314036


Confusion Matrix:
[[771 110]
 [390 609]]

Classification Report:
              precision    recall  f1-score   support

         0.0       0.66      0.88      0.76       881
         1.0       0.85      0.61      0.71       999

    accuracy                           0.73      1880
   macro avg       0.76      0.74      0.73      1880
weighted avg       0.76      0.73      0.73      1880
```

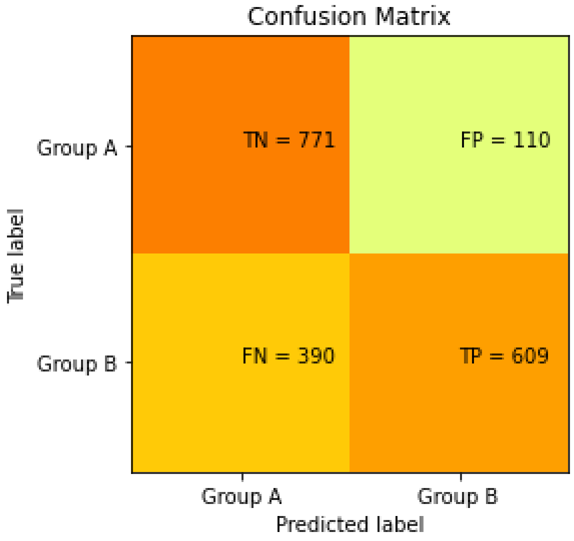


```
AUC Score : 0.7423757469160422

Accuracy Of the Model(GradientBoostingClassifier): 0.7340425531914894
```

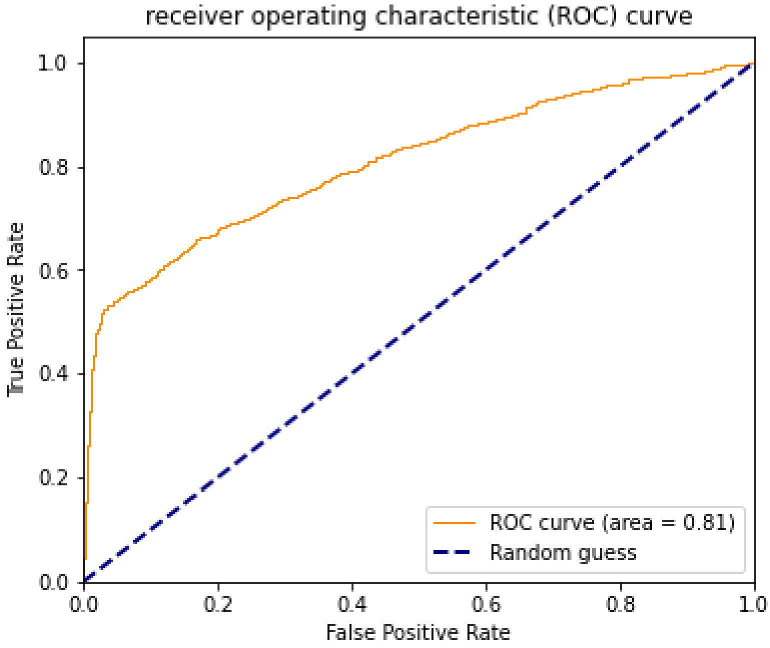

```
ROC_AUC Score of the model(GradientBoostingClassifier): 0.8100200086579201


Confusion Matrix:
[[804  77]
 [428 571]]

Classification Report:
              precision    recall  f1-score   support

         0.0       0.65      0.91      0.76       881
         1.0       0.88      0.57      0.69       999

    accuracy                           0.73      1880
   macro avg       0.77      0.74      0.73      1880
weighted avg       0.77      0.73      0.73      1880
```

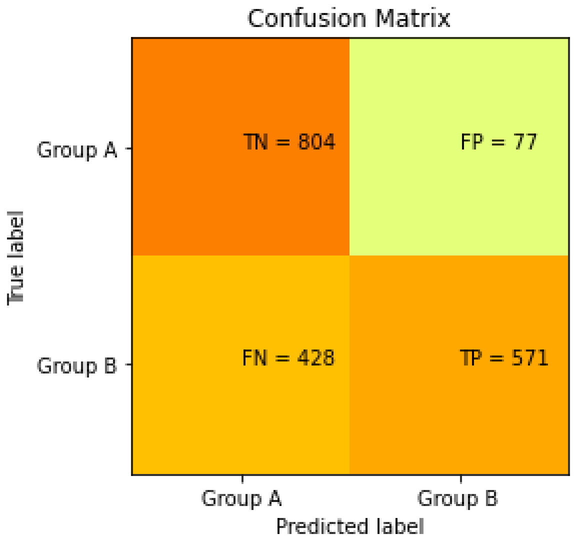


```
AUC Score : 0.7420854452636517

Accuracy Of the Model(AdaBoostClassifier): 0.7313829787234043
```

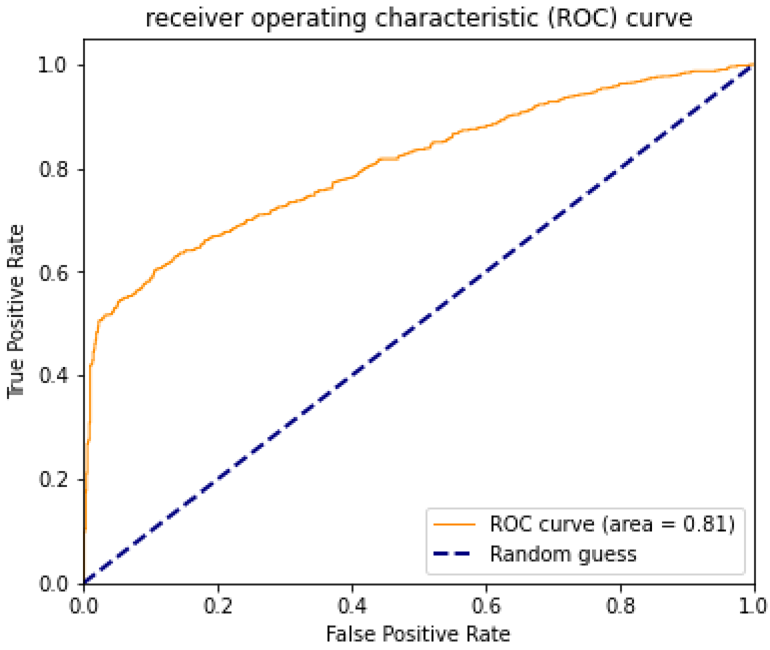

ROC_AUC Score of the model(AdaBoostClassifier): 0.8073385530820264

Confusion Matrix:
[[701 180]
 [348 651]]

Classification Report:

	precision	recall	f1-score	support
0.0	0.67	0.80	0.73	881
1.0	0.78	0.65	0.71	999
accuracy			0.72	1880
macro avg	0.73	0.72	0.72	1880
weighted avg	0.73	0.72	0.72	1880

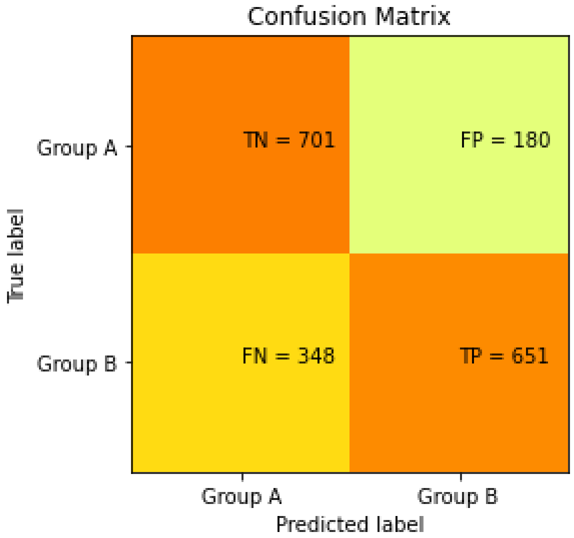

AUC Score : 0.7236691856442139

Accuracy Of the Model(SVC): 0.7191489361702128

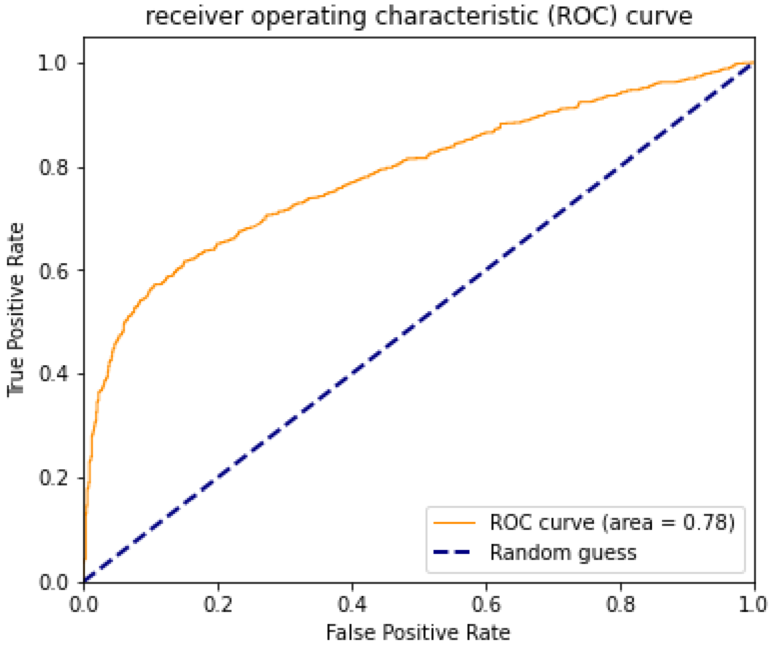

```
ROC_AUC Score of the model(SVC): 0.7835997177654385


Confusion Matrix:
[[783  98]
 [409 590]]

Classification Report:
              precision    recall  f1-score   support

         0.0       0.66      0.89      0.76       881
         1.0       0.86      0.59      0.70       999

    accuracy                           0.73      1880
   macro avg       0.76      0.74      0.73      1880
weighted avg       0.76      0.73      0.73      1880
```

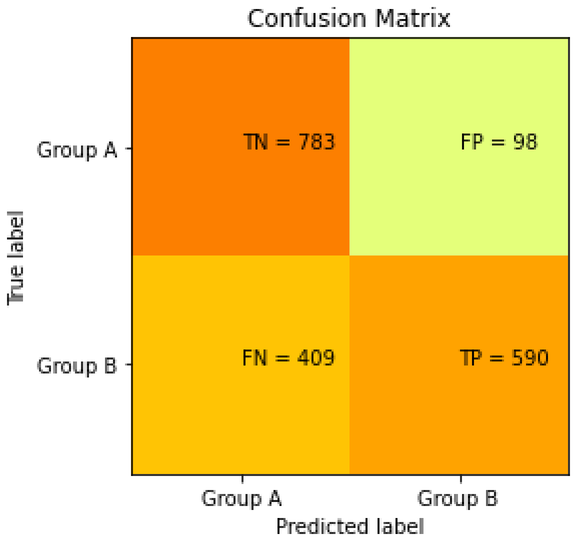


```
AUC Score : 0.7396766800853066

Accuracy Of the Model(MLPClassifier): 0.7303191489361702
```

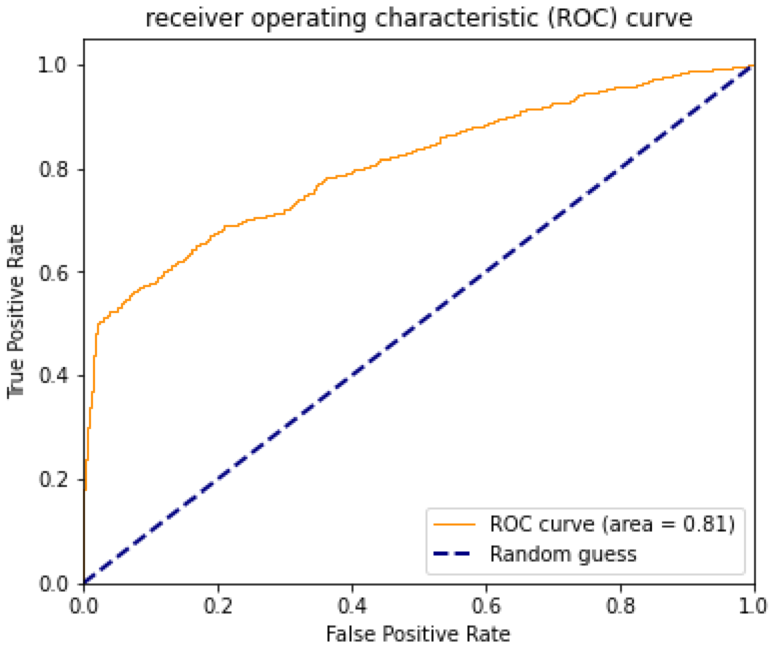

```
ROC_AUC Score of the model(MLPClassifier): 0.8083122850432726

[('XGBClassifier', 0.7414893617021276), ('LogisticRegression', 0.740425531914893
6), ('RandomForestClassifier', 0.7388297872340426), ('GradientBoostingClassifier',
0.7340425531914894), ('AdaBoostClassifier', 0.7313829787234043), ('MLPClassifier',
0.7303191489361702), ('DecisionTreeClassifier', 0.726063829787234), ('SVC', 0.7191
489361702128), ('ExtraTreesClassifier', 0.7186170212765958), ('KNeighborsClassifie
r', 0.7111702127659575)]


sorted_total_auc:
 [('XGBClassifier', 0.8118572602114031), ('GradientBoostingClassifier', 0.81002000
86579201), ('RandomForestClassifier', 0.8097473182603716), ('MLPClassifier', 0.808
3122850432726), ('AdaBoostClassifier', 0.8073385530820264), ('LogisticRegression',
0.8071522146437016), ('ExtraTreesClassifier', 0.7940596669314036), ('KNeighborsCla
ssifier', 0.7899965800079307), ('DecisionTreeClassifier', 0.7891540803005047), ('S
VC', 0.7835997177654385)]
```

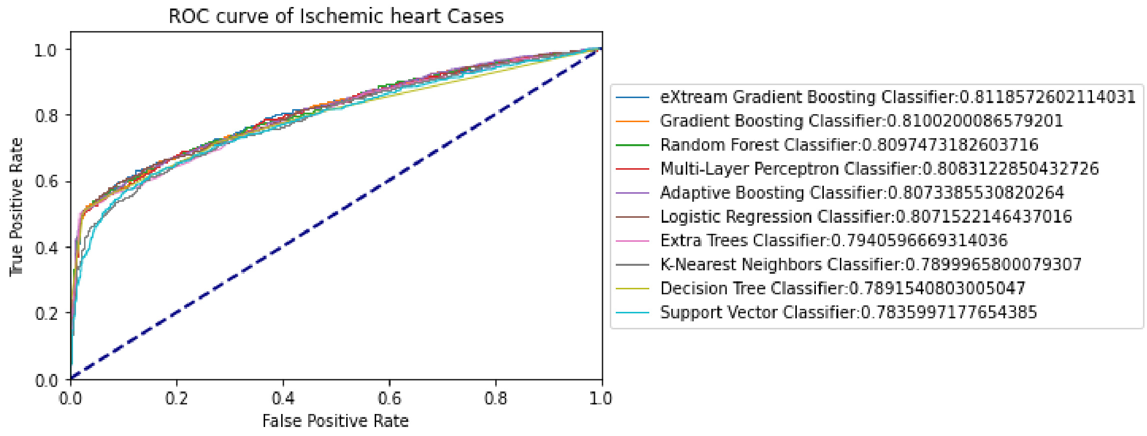

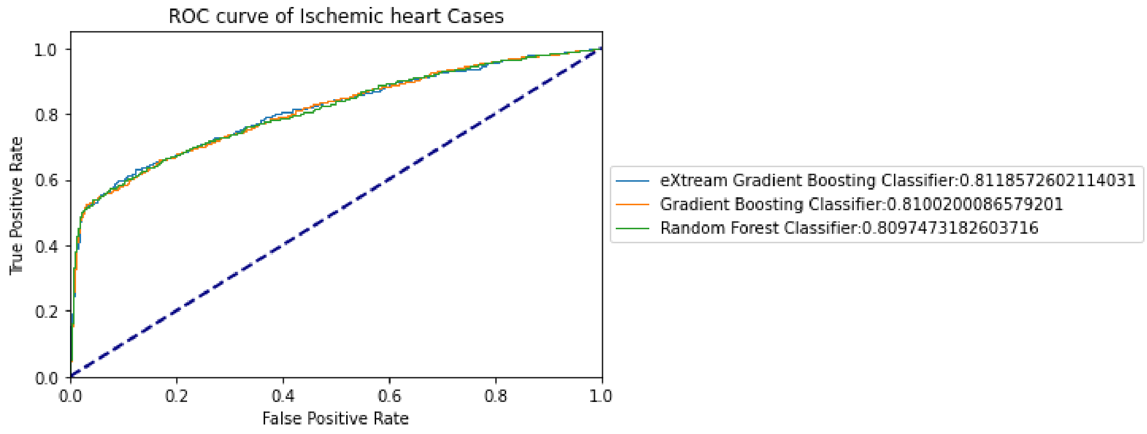

```
random state :   12436
9398
result_train_filename: data/train_test_data_ischemic heart_2013_20210519_123616.cs
v
total count : 9398, df_train count : 7518, df_test count : 1880
(7518, 38)
(1880, 38)
('Logistic Regression', LogisticRegression())
[2021-05-19 12:36:16.657021] Start parameter search for model 'Logistic Regressio
n'
[2021-05-19 12:36:17.035246] Finish parameter search for model 'Logistic Regressio
n' (time: 0 seconds)

=== train : best params ===
{'C': 0.1, 'penalty': 'l2', 'solver': 'lbfgs'}
=== train : best score : roc_auc ===
0.8046040395290845



('Random Forest', RandomForestClassifier())
[2021-05-19 12:36:17.036242] Start parameter search for model 'Random Forest'
[2021-05-19 12:36:21.474781] Finish parameter search for model 'Random Forest' (ti
me: 4 seconds)

=== train : best params ===
{'criterion': 'gini', 'max_depth': 10, 'max_features': 'auto', 'n_estimators': 10
0}
=== train : best score : roc_auc ===
0.8122014701960378



('Extreme Gradient Boosting)', XGBClassifier(base_score=None, booster=None, colsam
ple_bylevel=None,
              colsample_bynode=None, colsample_bytree=None, gamma=None,
              gpu_id=None, importance_type='gain', interaction_constraints=None,
              learning_rate=None, max_delta_step=None, max_depth=None,
              min_child_weight=None, missing=nan, monotone_constraints=None,
              n_estimators=100, n_jobs=None, num_parallel_tree=None,
              random_state=None, reg_alpha=None, reg_lambda=None,
              scale_pos_weight=None, subsample=None, tree_method=None,
              validate_parameters=None, verbosity=None))
[2021-05-19 12:36:21.475779] Start parameter search for model 'Extreme Gradient Bo
osting)'
[2021-05-19 12:36:47.181106] Finish parameter search for model 'Extreme Gradient B
oosting)' (time: 25 seconds)

=== train : best params ===
{'learning_rate': 0.05, 'max_depth': 5, 'n_estimators': 100, 'subsample': 0.7}
=== train : best score : roc_auc ===
0.8130562336918554



('k-Nearest Neighbours)', KNeighborsClassifier())
[2021-05-19 12:36:47.183100] Start parameter search for model 'k-Nearest Neighbour
s)'
[2021-05-19 12:36:55.118420] Finish parameter search for model 'k-Nearest Neighbou
rs)' (time: 7 seconds)

=== train : best params ===
{'n_neighbors': 60, 'weights': 'distance'}
=== train : best score : roc_auc ===
```

```
0.7878533365981979


('Decision Tree', DecisionTreeClassifier())
[2021-05-19 12:36:55.119413] Start parameter search for model 'Decision Tree'
[2021-05-19 12:36:55.680277] Finish parameter search for model 'Decision Tree' (ti
me: 0 seconds)

=== train : best params ===
{'criterion': 'gini', 'max_depth': 5}
=== train : best score : roc_auc ===
0.7946282160871255


('Extra Tree', ExtraTreesClassifier())
[2021-05-19 12:36:55.681275] Start parameter search for model 'Extra Tree'
[2021-05-19 12:36:58.489779] Finish parameter search for model 'Extra Tree' (time:
2 seconds)

=== train : best params ===
{'max_features': 'sqrt', 'n_estimators': 100}
=== train : best score : roc_auc ===
0.805611362581537


('Gradient Boosting', GradientBoostingClassifier())
[2021-05-19 12:36:58.490777] Start parameter search for model 'Gradient Boosting'
[2021-05-19 12:37:13.364002] Finish parameter search for model 'Gradient Boosting'
(time: 14 seconds)

=== train : best params ===
{'learning_rate': 0.075, 'max_depth': 5, 'max_features': 6, 'n_estimators': 100}
=== train : best score : roc_auc ===
0.8142229391553376


('Adaptive Boosting', AdaBoostClassifier())
[2021-05-19 12:37:13.365004] Start parameter search for model 'Adaptive Boosting'
[2021-05-19 12:37:16.907611] Finish parameter search for model 'Adaptive Boosting'
(time: 3 seconds)

=== train : best params ===
{'learning_rate': 0.2, 'n_estimators': 100}
=== train : best score : roc_auc ===
0.8047775471248745


('Suppport Vector Classfier', SVC(probability=True))
[2021-05-19 12:37:16.907611] Start parameter search for model 'Suppport Vector Cla
ssfier'
[2021-05-19 12:41:04.683390] Finish parameter search for model 'Suppport Vector Cl
assfier' (time: 227 seconds)

=== train : best params ===
{'C': 1, 'gamma': 0.1, 'kernel': 'rbf'}
=== train : best score : roc_auc ===
0.785477819163648


('Neural Network', MLPClassifier())
[2021-05-19 12:41:04.683390] Start parameter search for model 'Neural Network'
```

```
[2021-05-19 12:43:42.416672] Finish parameter search for model 'Neural Network' (t
ime: 157 seconds)

=== train : best params ===
{'activation': 'logistic', 'alpha': 0.05, 'hidden_layer_sizes': (50, 50), 'learnin
g_rate': 'adaptive'}
=== train : best score : roc_auc ===
0.8087217060749128



Confusion Matrix:
[[817  64]
 [430 569]]

Classification Report:
              precision    recall  f1-score   support

         0.0       0.66      0.93      0.77       881
         1.0       0.90      0.57      0.70       999

    accuracy                           0.74      1880
   macro avg       0.78      0.75      0.73      1880
weighted avg       0.78      0.74      0.73      1880
```

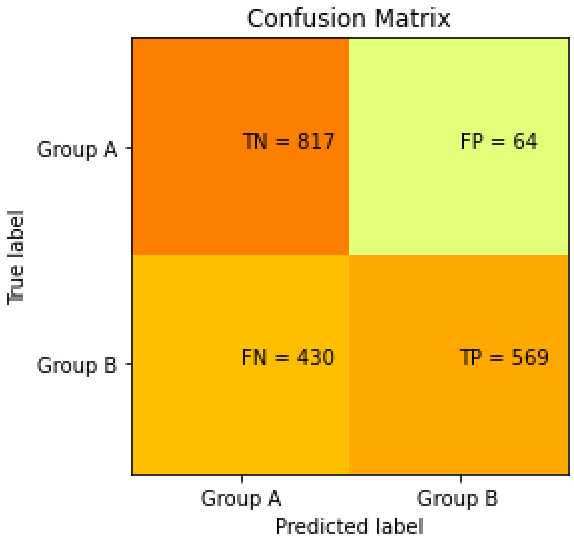


```
AUC Score : 0.7484624238313229

Accuracy Of the Model(LogisticRegression): 0.7372340425531915
```

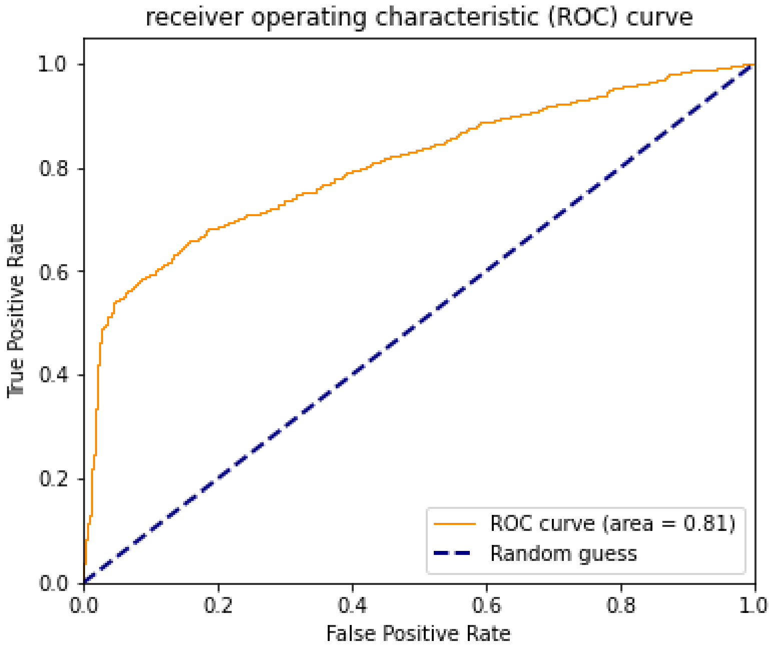


```
ROC_AUC Score of the model(LogisticRegression): 0.8052774681605555


Confusion Matrix:
[[802  79]
 [417 582]]

Classification Report:
              precision    recall  f1-score   support

         0.0       0.66      0.91      0.76       881
         1.0       0.88      0.58      0.70       999

    accuracy                           0.74      1880
   macro avg       0.77      0.75      0.73      1880
weighted avg       0.78      0.74      0.73      1880
```

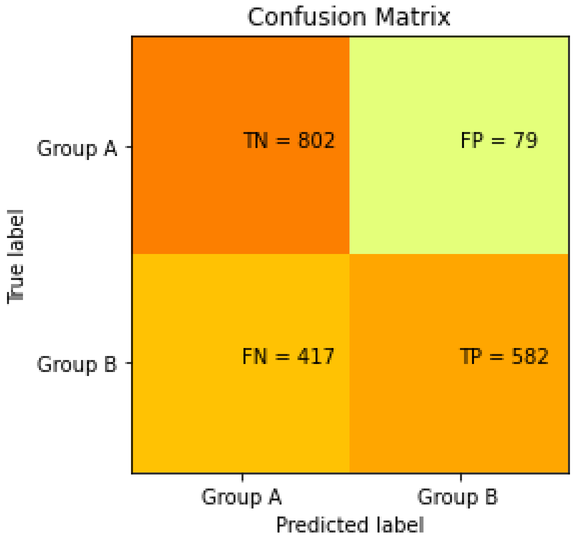


```
AUC Score : 0.7464558769893617

Accuracy Of the Model(RandomForestClassifier): 0.7361702127659574
```

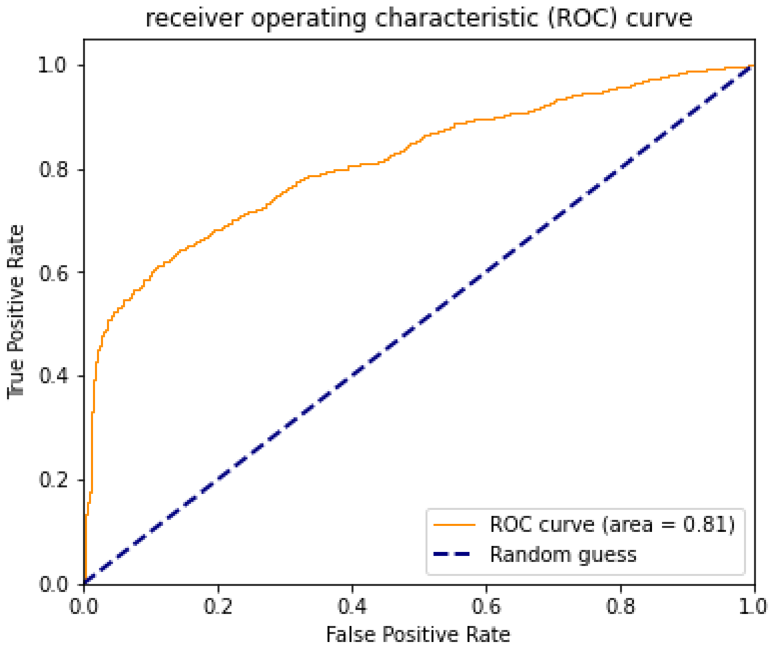

```
ROC_AUC Score of the model(RandomForestClassifier): 0.8146852868759793


Confusion Matrix:
[[803  78]
 [403 596]]

Classification Report:
              precision    recall  f1-score   support

         0.0       0.67      0.91      0.77       881
         1.0       0.88      0.60      0.71       999

    accuracy                           0.74      1880
   macro avg       0.78      0.75      0.74      1880
weighted avg       0.78      0.74      0.74      1880
```

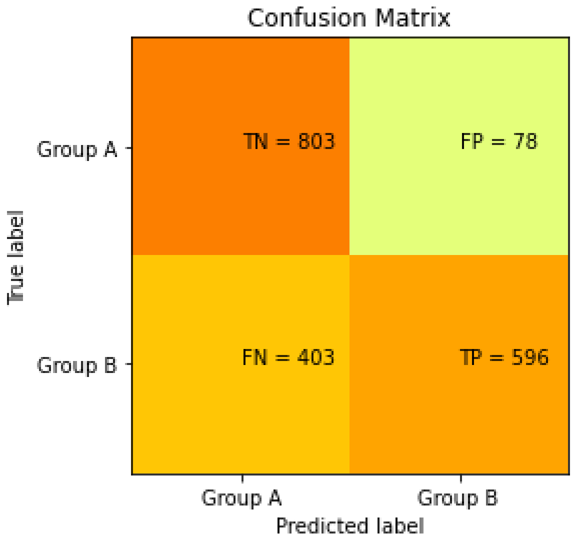


```
AUC Score : 0.7540304208862665

Accuracy Of the Model(XGBClassifier): 0.7441489361702127
```

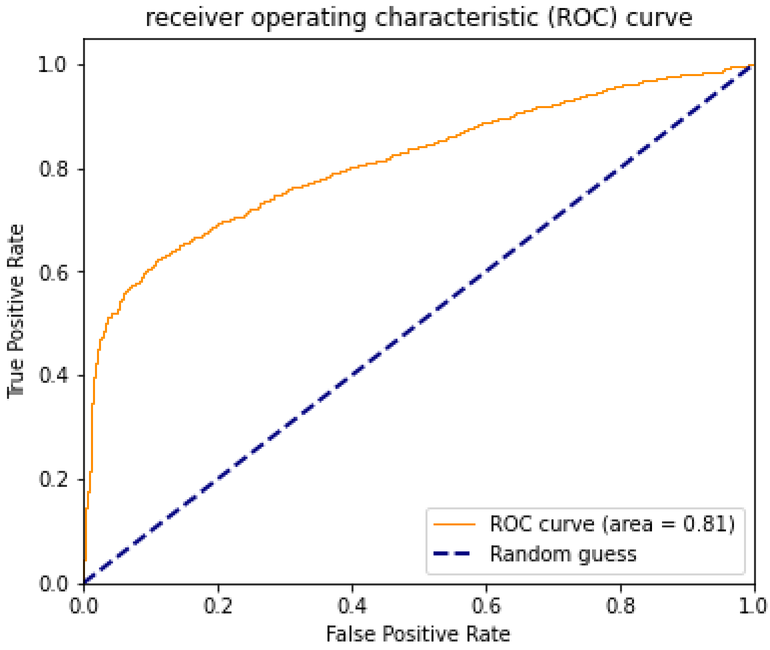

ROC_AUC Score of the model(XGBClassifier): 0.8124617239259693

Confusion Matrix:
[[800 81]
 [458 541]]

Classification Report:

	precision	recall	f1-score	support
0.0	0.64	0.91	0.75	881
1.0	0.87	0.54	0.67	999
accuracy			0.71	1880
macro avg	0.75	0.72	0.71	1880
weighted avg	0.76	0.71	0.71	1880

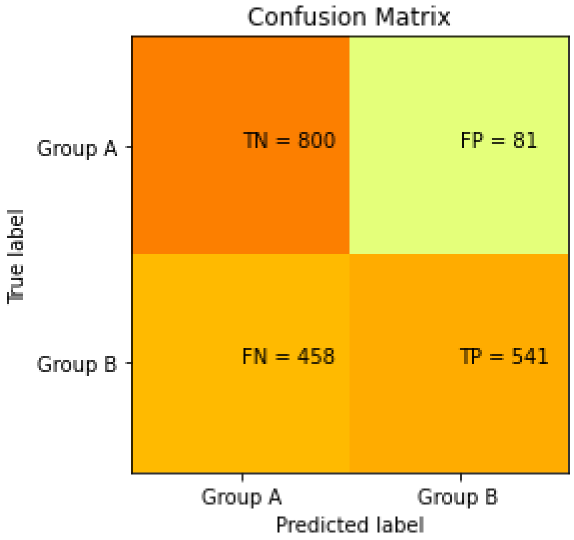

AUC Score : 0.7248002826890454

Accuracy Of the Model(KNeighborsClassifier): 0.7132978723404255

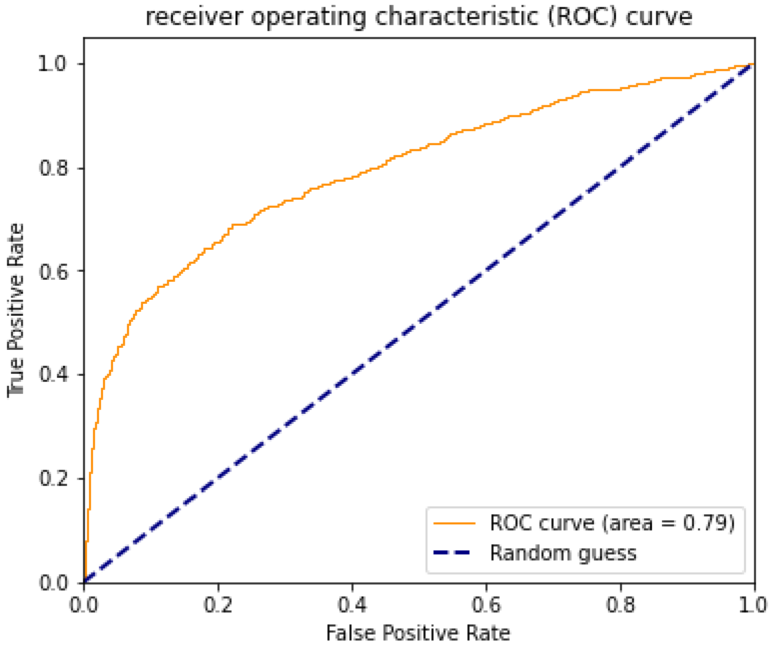

```
ROC_AUC Score of the model(KNeighborsClassifier): 0.7946368616062146


Confusion Matrix:
[[795  86]
 [407 592]]

Classification Report:
              precision    recall  f1-score   support

         0.0       0.66      0.90      0.76       881
         1.0       0.87      0.59      0.71       999

    accuracy                           0.74      1880
   macro avg       0.77      0.75      0.73      1880
weighted avg       0.77      0.74      0.73      1880
```

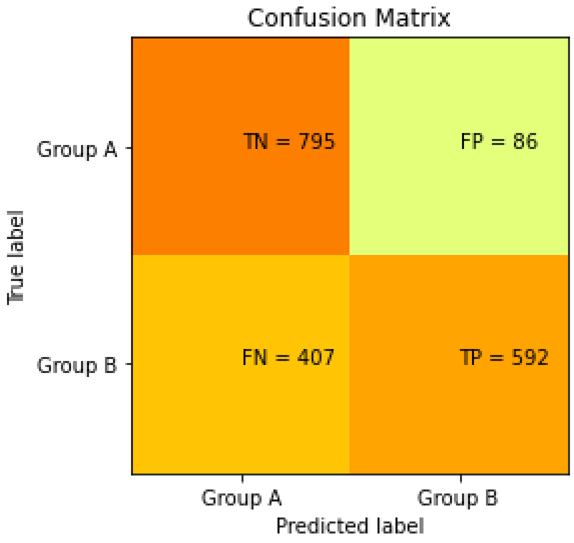


```
AUC Score : 0.7474881237650818

Accuracy Of the Model(DecisionTreeClassifier): 0.7377659574468085
```

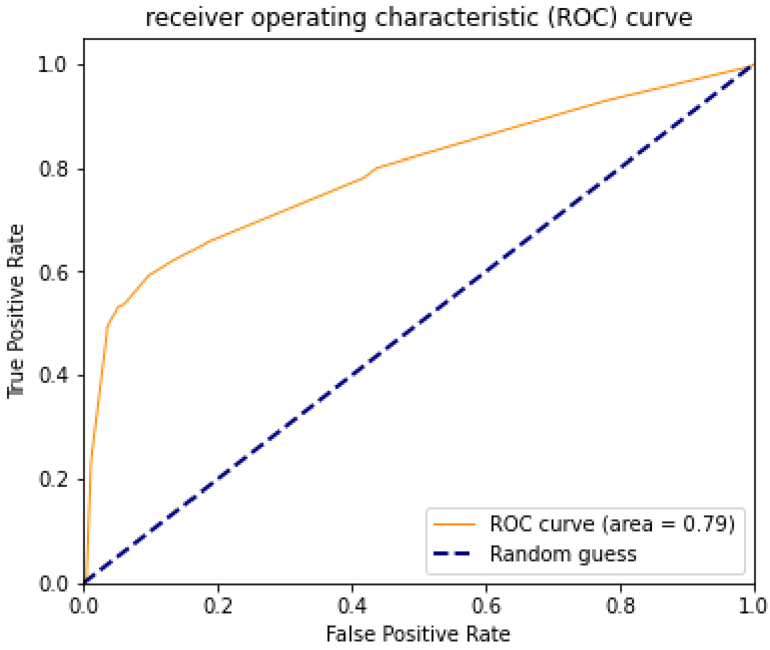

```
ROC_AUC Score of the model(DecisionTreeClassifier): 0.7891722596603414


Confusion Matrix:
[[790  91]
 [415 584]]

Classification Report:
              precision    recall  f1-score   support

         0.0       0.66      0.90      0.76       881
         1.0       0.87      0.58      0.70       999

    accuracy                           0.73      1880
   macro avg       0.76      0.74      0.73      1880
weighted avg       0.77      0.73      0.73      1880
```

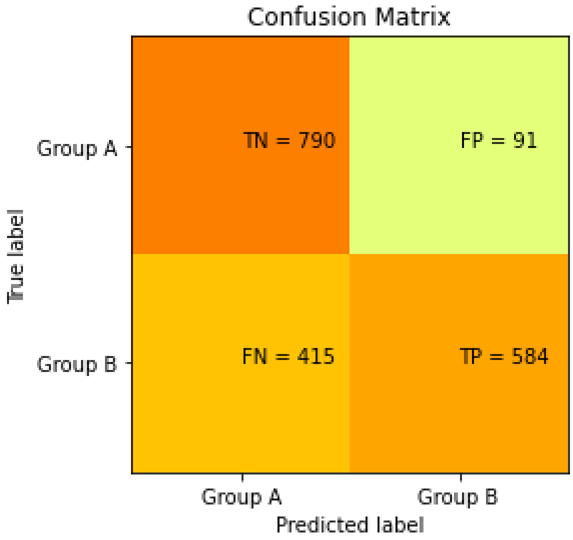


```
AUC Score : 0.7406464353115886

Accuracy Of the Model(ExtraTreesClassifier): 0.7308510638297873
```

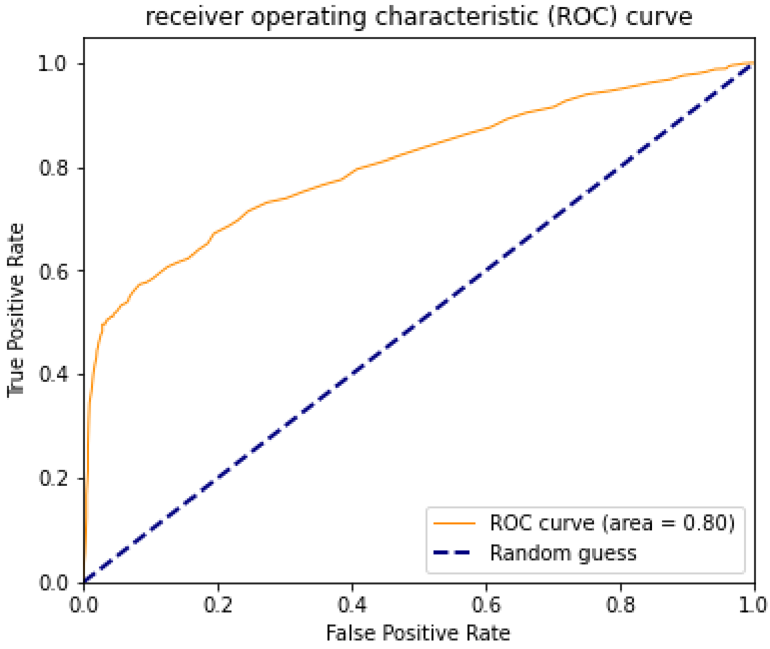

```
ROC_AUC Score of the model(ExtraTreesClassifier): 0.803543043611148


Confusion Matrix:
[[785  96]
 [397 602]]

Classification Report:
              precision    recall  f1-score   support

         0.0       0.66      0.89      0.76       881
         1.0       0.86      0.60      0.71       999

    accuracy                           0.74      1880
   macro avg       0.76      0.75      0.74      1880
weighted avg       0.77      0.74      0.73      1880
```

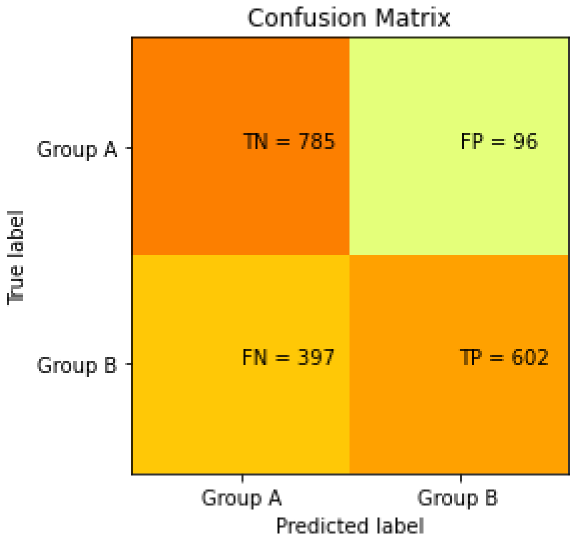


```
AUC Score : 0.7468177598711083

Accuracy Of the Model(GradientBoostingClassifier): 0.7377659574468085
```

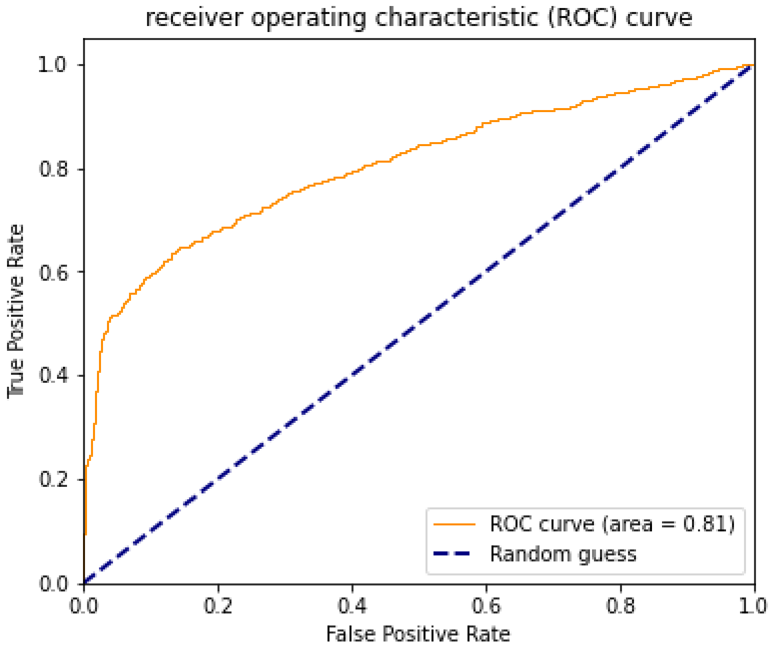

```
ROC_AUC Score of the model(GradientBoostingClassifier): 0.8058148954857242


Confusion Matrix:
[[813  68]
 [436 563]]

Classification Report:
              precision    recall  f1-score   support

         0.0       0.65      0.92      0.76       881
         1.0       0.89      0.56      0.69       999

    accuracy                           0.73      1880
   macro avg       0.77      0.74      0.73      1880
weighted avg       0.78      0.73      0.72      1880
```

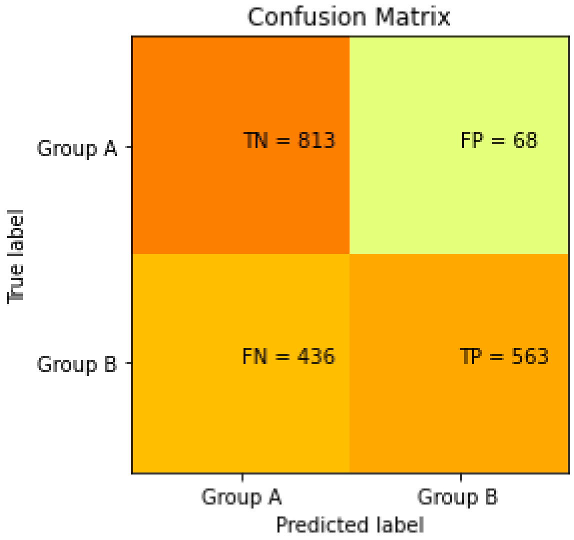


```
AUC Score : 0.7431892732687284

Accuracy Of the Model(AdaBoostClassifier): 0.7319148936170212
```

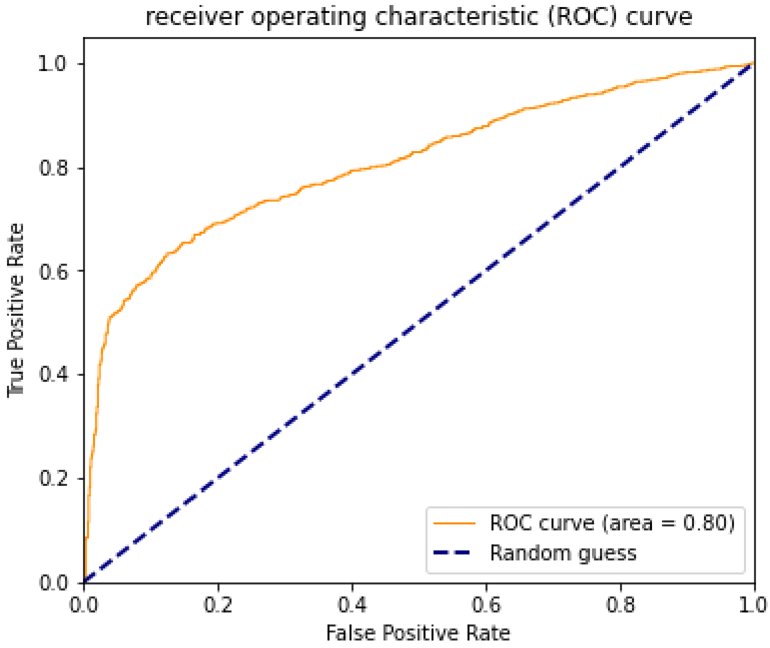

```
ROC_AUC Score of the model(AdaBoostClassifier): 0.8049479672635177


Confusion Matrix:
[[689 192]
 [342 657]]

Classification Report:
              precision    recall  f1-score   support

         0.0       0.67      0.78      0.72       881
         1.0       0.77      0.66      0.71       999

    accuracy                           0.72      1880
   macro avg       0.72      0.72      0.72      1880
weighted avg       0.72      0.72      0.72      1880
```

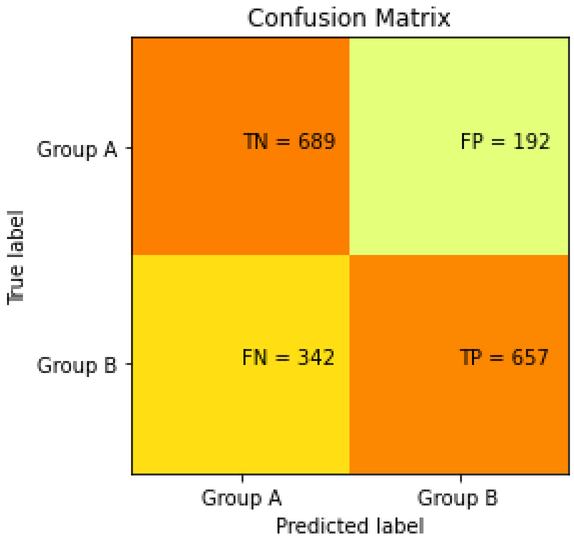


```
AUC Score : 0.7198617459684429

Accuracy Of the Model(SVC): 0.7159574468085106
```

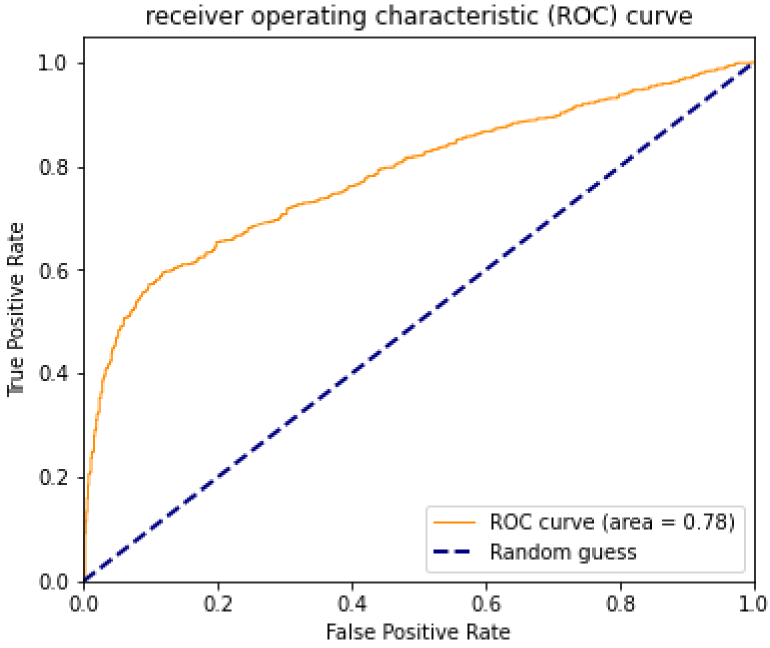

```
ROC_AUC Score of the model(SVC): 0.7823674980315162


Confusion Matrix:
[[778 103]
 [400 599]]

Classification Report:
              precision    recall  f1-score   support

         0.0       0.66      0.88      0.76       881
         1.0       0.85      0.60      0.70       999

    accuracy                           0.73      1880
   macro avg       0.76      0.74      0.73      1880
weighted avg       0.76      0.73      0.73      1880
```

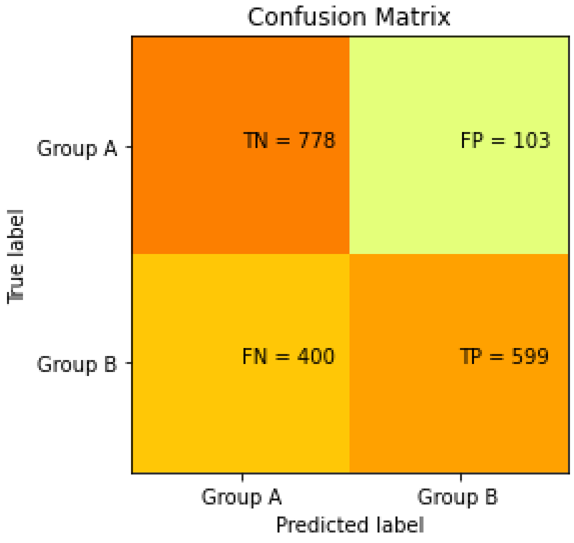


```
AUC Score : 0.7413435001403222

Accuracy Of the Model(MLPClassifier): 0.7324468085106383
```

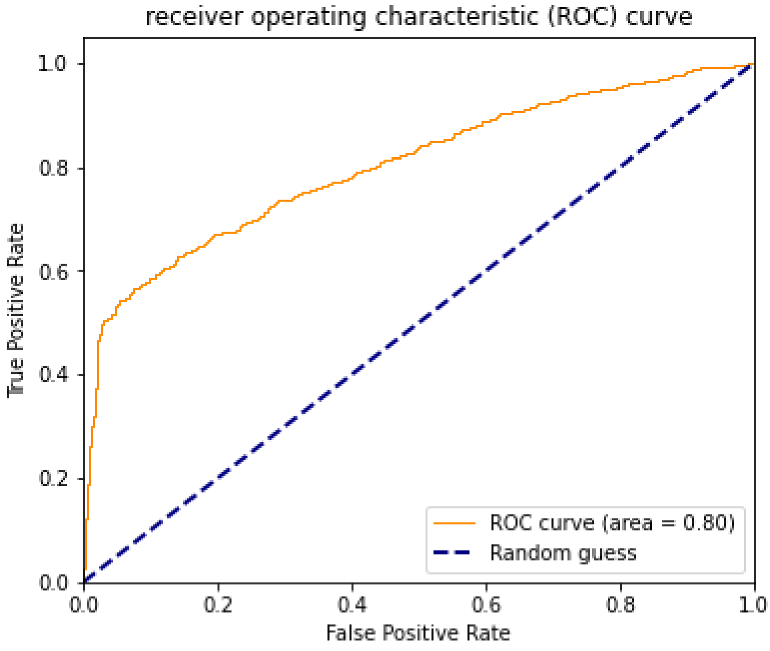

```
ROC_AUC Score of the model(MLPClassifier): 0.8046218749964493

[('XGBClassifier', 0.7441489361702127), ('DecisionTreeClassifier', 0.7377659574468085), ('GradientBoostingClassifier', 0.7377659574468085), ('LogisticRegression', 0.7372340425531915), ('RandomForestClassifier', 0.7361702127659574), ('MLPClassifier', 0.7324468085106383), ('AdaBoostClassifier', 0.7319148936170212), ('ExtraTreesClassifier', 0.7308510638297873), ('SVC', 0.7159574468085106), ('KNeighborsClassifier', 0.7132978723404255)]


sorted_total_auc:
 [('RandomForestClassifier', 0.8146852868759793), ('XGBClassifier', 0.8124617239259693), ('GradientBoostingClassifier', 0.8058148954857242), ('LogisticRegression', 0.8052774681605555), ('AdaBoostClassifier', 0.8049479672635177), ('MLPClassifier', 0.8046218749964493), ('ExtraTreesClassifier', 0.803543043611148), ('KNeighborsClassifier', 0.7946368616062146), ('DecisionTreeClassifier', 0.7891722596603414), ('SVC', 0.7823674980315162)]
```

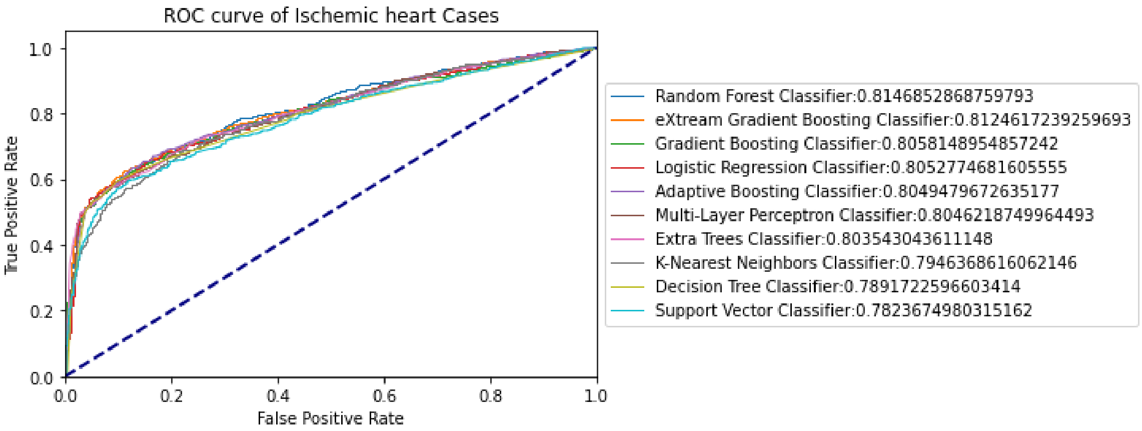

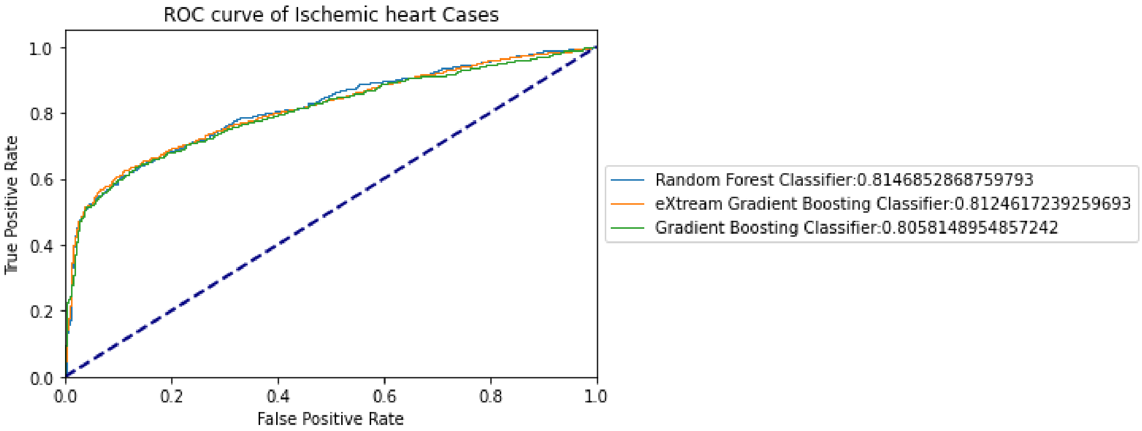

```
random state :   12450
9398
result_train_filename: data/train_test_data_ischemic heart_2013_20210519_124350.cs
v
total count : 9398, df_train count : 7518, df_test count : 1880
(7518, 38)
(1880, 38)
('Logistic Regression', LogisticRegression())
[2021-05-19 12:43:50.315624] Start parameter search for model 'Logistic Regressio
n'
[2021-05-19 12:43:50.675914] Finish parameter search for model 'Logistic Regressio
n' (time: 0 seconds)

=== train : best params ===
{'C': 0.1, 'penalty': 'l2', 'solver': 'lbfgs'}
=== train : best score : roc_auc ===
0.8060674522701057


('Random Forest', RandomForestClassifier())
[2021-05-19 12:43:50.676888] Start parameter search for model 'Random Forest'
[2021-05-19 12:43:54.995601] Finish parameter search for model 'Random Forest' (ti
me: 4 seconds)

=== train : best params ===
{'criterion': 'gini', 'max_depth': 8, 'max_features': 'auto', 'n_estimators': 70}
=== train : best score : roc_auc ===
0.8150421744339339


('Extreme Gradient Boosting)', XGBClassifier(base_score=None, booster=None, colsam
ple_bylevel=None,
              colsample_bynode=None, colsample_bytree=None, gamma=None,
              gpu_id=None, importance_type='gain', interaction_constraints=None,
              learning_rate=None, max_delta_step=None, max_depth=None,
              min_child_weight=None, missing=nan, monotone_constraints=None,
              n_estimators=100, n_jobs=None, num_parallel_tree=None,
              random_state=None, reg_alpha=None, reg_lambda=None,
              scale_pos_weight=None, subsample=None, tree_method=None,
              validate_parameters=None, verbosity=None))
[2021-05-19 12:43:54.995601] Start parameter search for model 'Extreme Gradient Bo
osting)'
[2021-05-19 12:44:22.037339] Finish parameter search for model 'Extreme Gradient B
oosting)' (time: 27 seconds)

=== train : best params ===
{'learning_rate': 0.075, 'max_depth': 5, 'n_estimators': 50, 'subsample': 0.8}
=== train : best score : roc_auc ===
0.8157033606676107


('k-Nearest Neighbours)', KNeighborsClassifier())
[2021-05-19 12:44:22.039333] Start parameter search for model 'k-Nearest Neighbour
s)'
[2021-05-19 12:44:29.931810] Finish parameter search for model 'k-Nearest Neighbou
rs)' (time: 7 seconds)

=== train : best params ===
{'n_neighbors': 60, 'weights': 'distance'}
=== train : best score : roc_auc ===
0.7899865772644967
```

```
('Decision Tree', DecisionTreeClassifier())
[2021-05-19 12:44:29.931810] Start parameter search for model 'Decision Tree'
[2021-05-19 12:44:30.517068] Finish parameter search for model 'Decision Tree' (ti
me: 0 seconds)

=== train : best params ===
{'criterion': 'entropy', 'max_depth': 5}
=== train : best score : roc_auc ===
0.7983847313136782


('Extra Tree', ExtraTreesClassifier())
[2021-05-19 12:44:30.518068] Start parameter search for model 'Extra Tree'
[2021-05-19 12:44:33.485110] Finish parameter search for model 'Extra Tree' (time:
2 seconds)

=== train : best params ===
{'max_features': 'log2', 'n_estimators': 100}
=== train : best score : roc_auc ===
0.8075078978443817


('Gradient Boosting', GradientBoostingClassifier())
[2021-05-19 12:44:33.486105] Start parameter search for model 'Gradient Boosting'
[2021-05-19 12:44:49.377083] Finish parameter search for model 'Gradient Boosting'
(time: 15 seconds)

=== train : best params ===
{'learning_rate': 0.01, 'max_depth': 8, 'max_features': 6, 'n_estimators': 100}
=== train : best score : roc_auc ===
0.8136950910949972


('Adaptive Boosting', AdaBoostClassifier())
[2021-05-19 12:44:49.377083] Start parameter search for model 'Adaptive Boosting'
[2021-05-19 12:44:52.503435] Finish parameter search for model 'Adaptive Boosting'
(time: 3 seconds)

=== train : best params ===
{'learning_rate': 0.2, 'n_estimators': 50}
=== train : best score : roc_auc ===
0.8070309311510955


('Suppport Vector Classfier', SVC(probability=True))
[2021-05-19 12:44:52.504402] Start parameter search for model 'Suppport Vector Cla
ssfier'
[2021-05-19 12:49:04.432959] Finish parameter search for model 'Suppport Vector Cl
assfier' (time: 251 seconds)

=== train : best params ===
{'C': 1, 'gamma': 0.1, 'kernel': 'rbf'}
=== train : best score : roc_auc ===
0.7827358337984918


('Neural Network', MLPClassifier())
[2021-05-19 12:49:04.432959] Start parameter search for model 'Neural Network'
[2021-05-19 12:51:46.200162] Finish parameter search for model 'Neural Network' (t
```

```
ime: 161 seconds)

=== train : best params ===
{'activation': 'logistic', 'alpha': 0.05, 'hidden_layer_sizes': (50,), 'learning_r
ate': 'adaptive'}
=== train : best score : roc_auc ===
0.8121992991903425



Confusion Matrix:
[[811  70]
 [427 572]]

Classification Report:
              precision    recall  f1-score   support

         0.0       0.66      0.92      0.77       881
         1.0       0.89      0.57      0.70       999

    accuracy                           0.74      1880
   macro avg       0.77      0.75      0.73      1880
weighted avg       0.78      0.74      0.73      1880
```

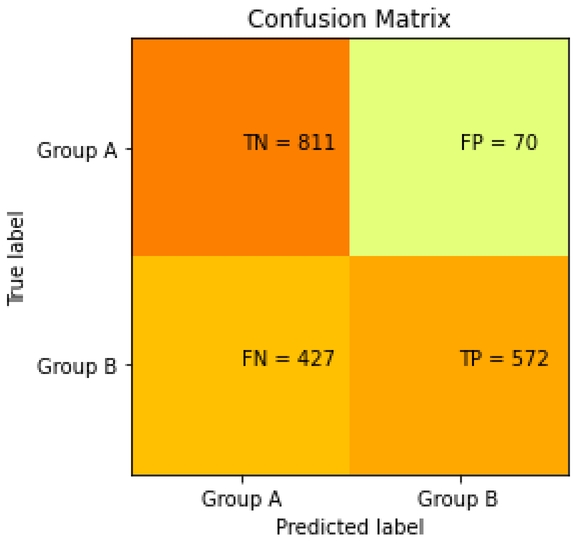


```
AUC Score : 0.7465587039934373

Accuracy Of the Model(LogisticRegression): 0.7356382978723405
```

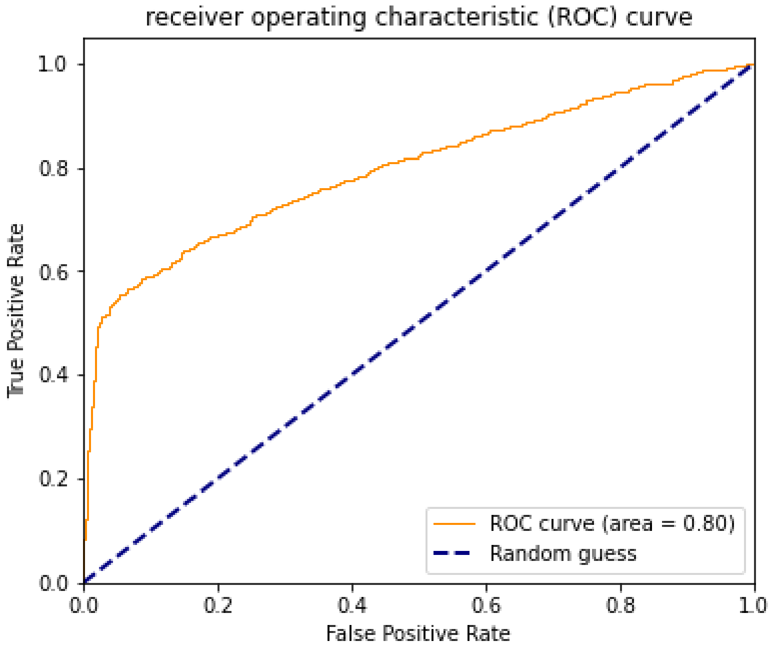


```
ROC_AUC Score of the model(LogisticRegression): 0.7988033436387579


Confusion Matrix:
[[797  84]
 [396 603]]

Classification Report:
              precision    recall  f1-score   support

         0.0       0.67      0.90      0.77       881
         1.0       0.88      0.60      0.72       999

    accuracy                           0.74      1880
   macro avg       0.77      0.75      0.74      1880
weighted avg       0.78      0.74      0.74      1880
```

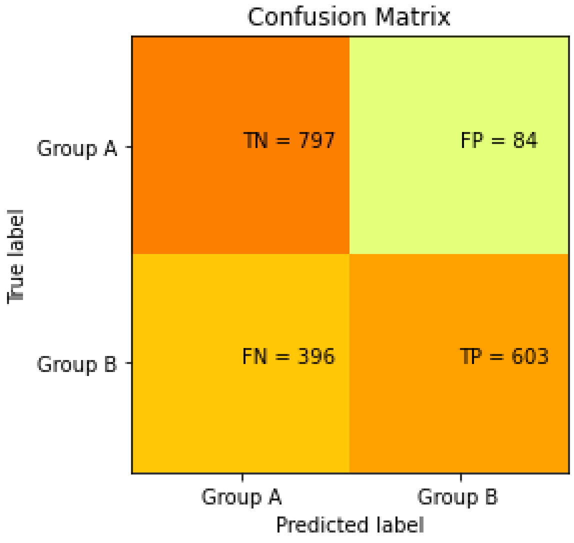

AUC Score : 0.7541287703050383

Accuracy Of the Model(RandomForestClassifier): 0.7446808510638298

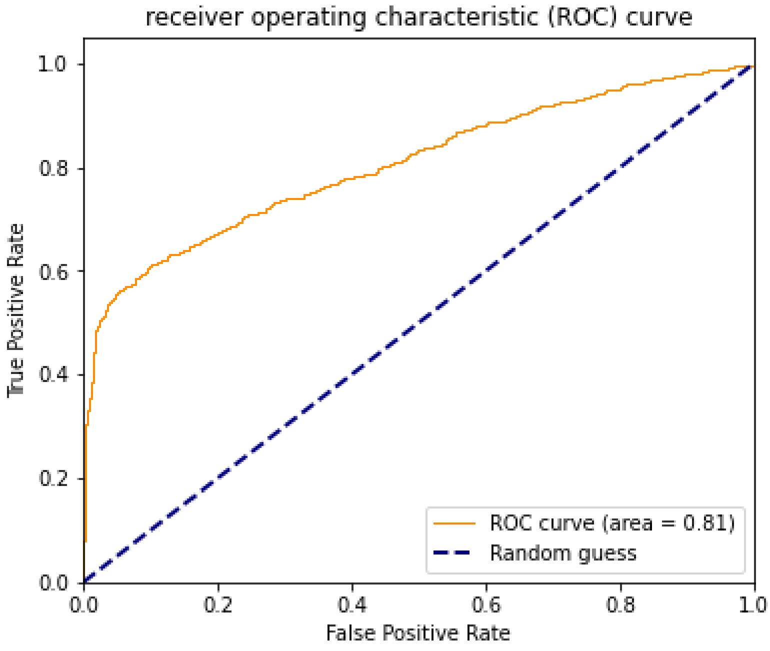

```
ROC_AUC Score of the model(RandomForestClassifier): 0.8076044262196361


Confusion Matrix:
[[800  81]
 [400 599]]

Classification Report:
              precision    recall  f1-score   support

         0.0       0.67      0.91      0.77       881
         1.0       0.88      0.60      0.71       999

    accuracy                           0.74      1880
   macro avg       0.77      0.75      0.74      1880
weighted avg       0.78      0.74      0.74      1880
```

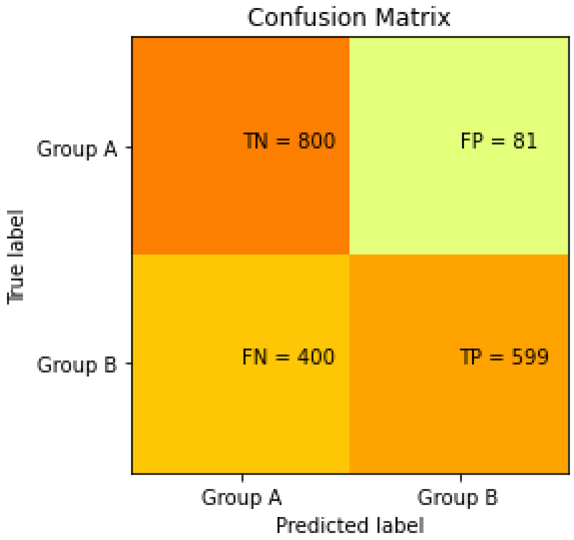


```
AUC Score : 0.7538293117180745

Accuracy Of the Model(XGBClassifier): 0.7441489361702127
```

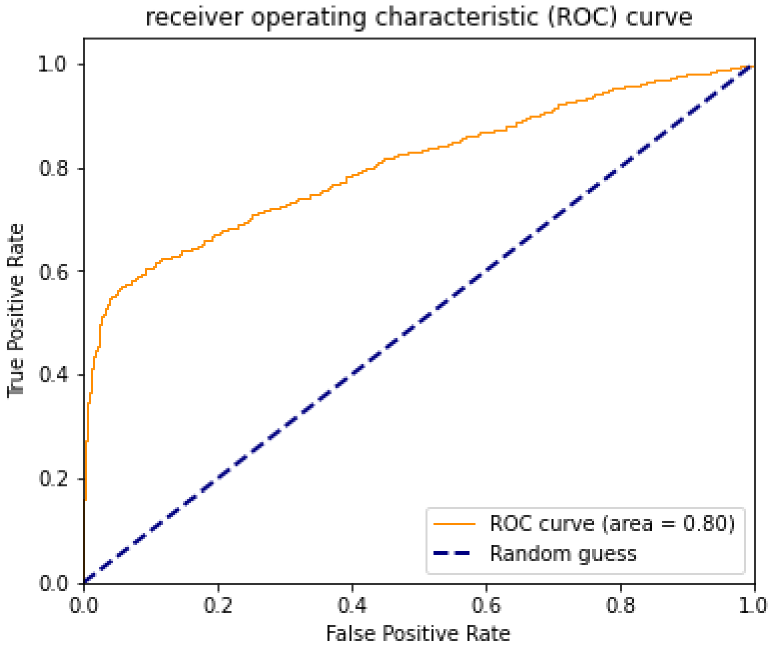

```
ROC_AUC Score of the model(XGBClassifier): 0.8049968242930785


Confusion Matrix:
[[807  74]
 [461 538]]

Classification Report:
              precision    recall  f1-score   support

         0.0       0.64      0.92      0.75       881
         1.0       0.88      0.54      0.67       999

    accuracy                           0.72      1880
   macro avg       0.76      0.73      0.71      1880
weighted avg       0.77      0.72      0.71      1880
```

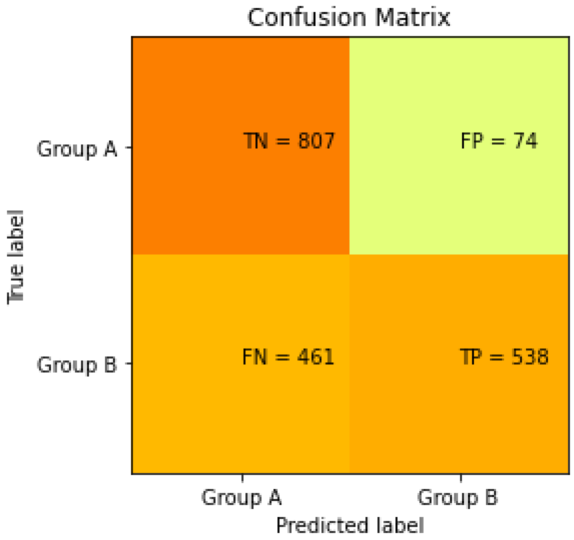


```
AUC Score : 0.7272715394168288

Accuracy Of the Model(KNeighborsClassifier): 0.7154255319148937
```

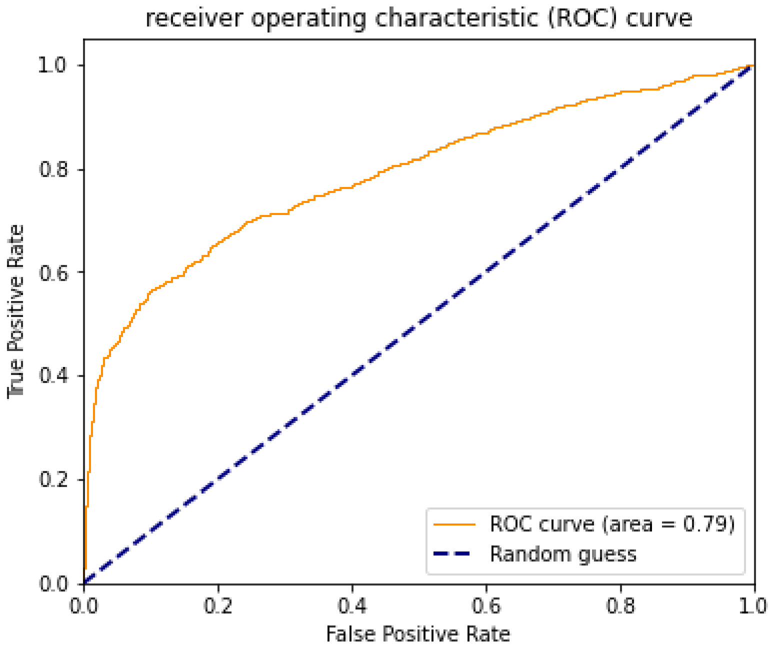

```
ROC_AUC Score of the model(KNeighborsClassifier): 0.7897363879202697


Confusion Matrix:
[[788  93]
 [410 589]]

Classification Report:
              precision    recall  f1-score   support

         0.0       0.66      0.89      0.76       881
         1.0       0.86      0.59      0.70       999

    accuracy                           0.73      1880
   macro avg       0.76      0.74      0.73      1880
weighted avg       0.77      0.73      0.73      1880
```

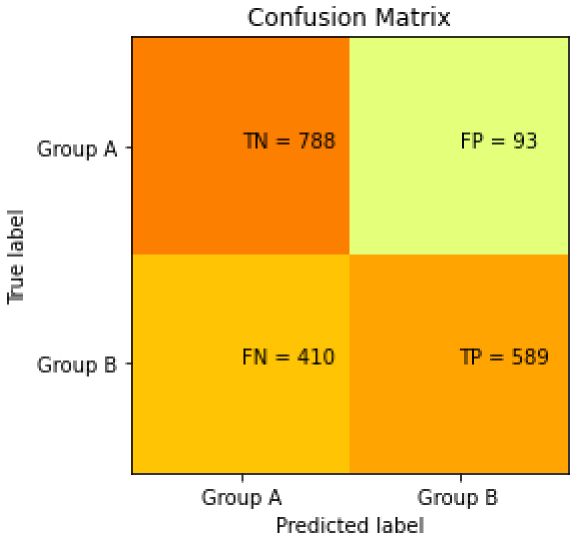


```
AUC Score : 0.7420138640342954

Accuracy Of the Model(DecisionTreeClassifier): 0.7324468085106383
```

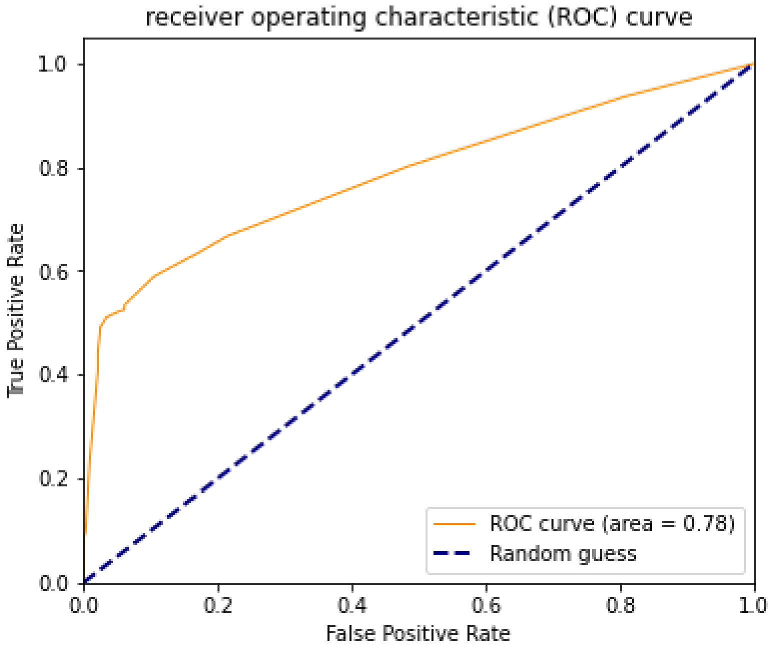

ROC_AUC Score of the model(DecisionTreeClassifier): 0.7842837161792893

```
Confusion Matrix:
[[793  88]
 [411 588]]

Classification Report:
              precision    recall  f1-score   support

         0.0       0.66      0.90      0.76       881
         1.0       0.87      0.59      0.70       999

    accuracy                           0.73      1880
   macro avg       0.76      0.74      0.73      1880
weighted avg       0.77      0.73      0.73      1880
```

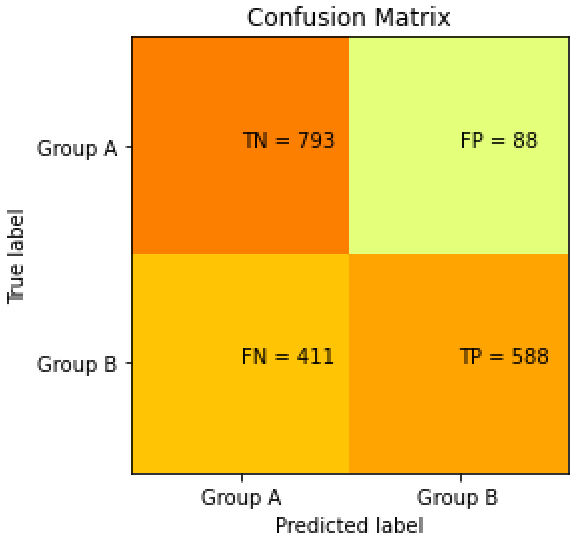

AUC Score : 0.7443510479832841

Accuracy Of the Model(ExtraTreesClassifier): 0.7345744680851064

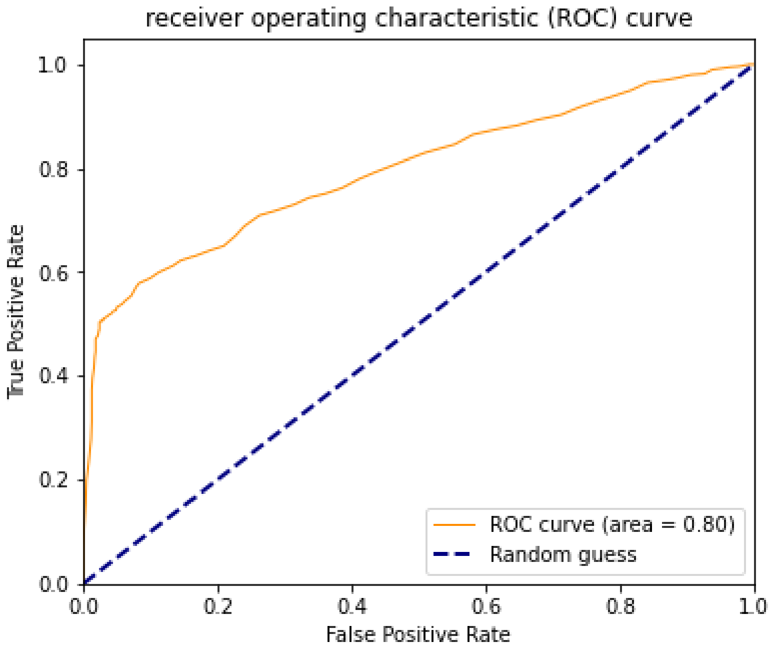

```
ROC_AUC Score of the model(ExtraTreesClassifier): 0.7960451938885537


Confusion Matrix:
[[791  90]
 [392 607]]

Classification Report:
              precision    recall  f1-score   support

         0.0       0.67      0.90      0.77       881
         1.0       0.87      0.61      0.72       999

    accuracy                           0.74      1880
   macro avg       0.77      0.75      0.74      1880
weighted avg       0.78      0.74      0.74      1880
```

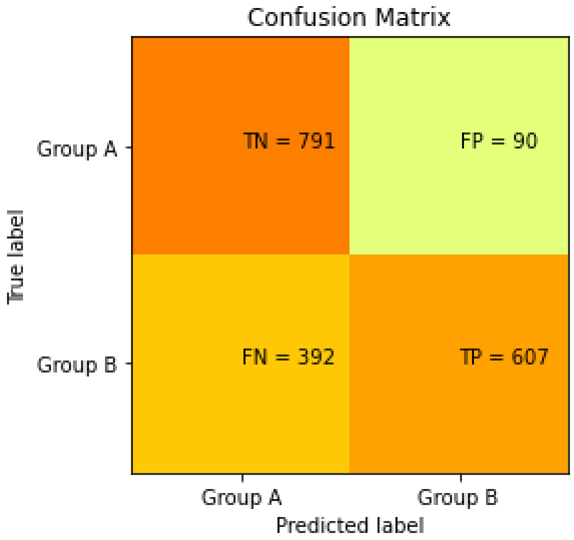


```
AUC Score : 0.7527254837129979

Accuracy Of the Model(GradientBoostingClassifier): 0.7436170212765958
```

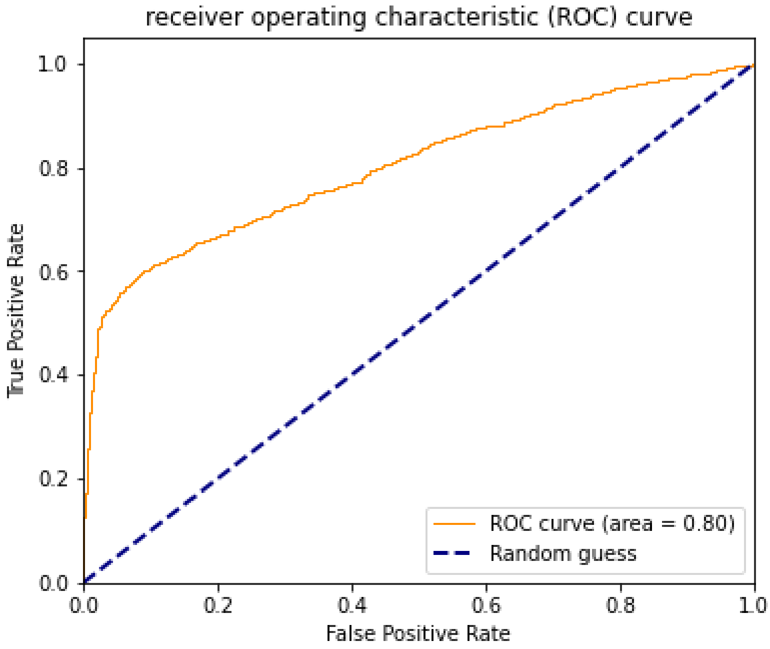

```
ROC_AUC Score of the model(GradientBoostingClassifier): 0.8033902233675219


Confusion Matrix:
[[836  45]
 [452 547]]

Classification Report:
              precision    recall  f1-score   support

         0.0       0.65      0.95      0.77       881
         1.0       0.92      0.55      0.69       999

    accuracy                           0.74      1880
   macro avg       0.79      0.75      0.73      1880
weighted avg       0.80      0.74      0.73      1880
```

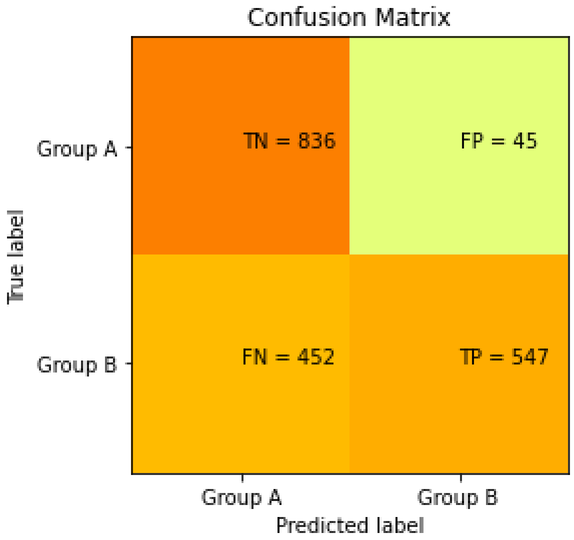


```
AUC Score : 0.7482346137283707

Accuracy Of the Model(AdaBoostClassifier): 0.7356382978723405
```

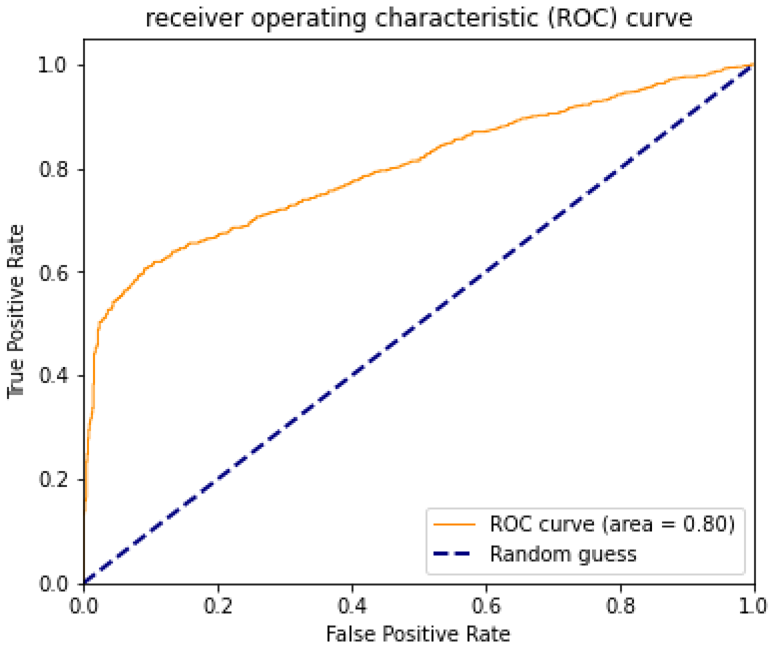

```
ROC_AUC Score of the model(AdaBoostClassifier): 0.7998020722197794


Confusion Matrix:
[[714 167]
 [347 652]]

Classification Report:
              precision    recall  f1-score   support

         0.0       0.67      0.81      0.74       881
         1.0       0.80      0.65      0.72       999

    accuracy                           0.73      1880
   macro avg       0.73      0.73      0.73      1880
weighted avg       0.74      0.73      0.73      1880
```

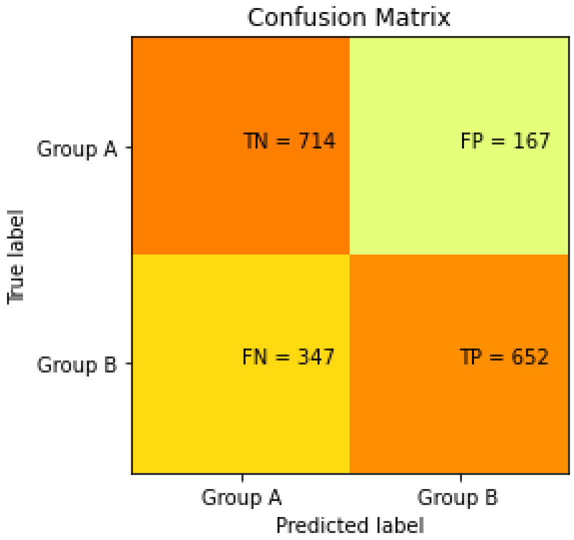


```
AUC Score : 0.7315476657133865

Accuracy Of the Model(SVC): 0.7265957446808511
```

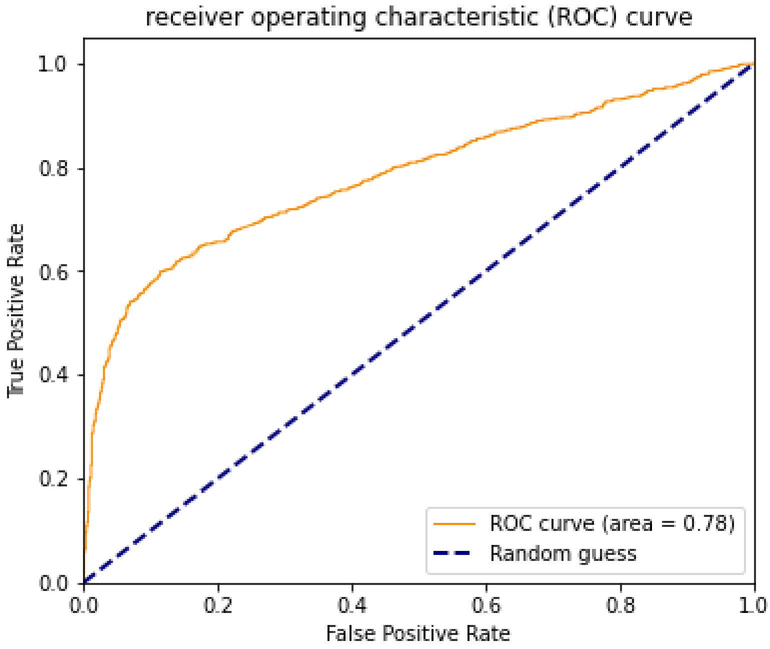

```
ROC_AUC Score of the model(SVC): 0.7824964578653568


Confusion Matrix:
[[796  85]
 [402 597]]

Classification Report:
              precision    recall  f1-score   support

         0.0       0.66      0.90      0.77       881
         1.0       0.88      0.60      0.71       999

    accuracy                           0.74      1880
   macro avg       0.77      0.75      0.74      1880
weighted avg       0.78      0.74      0.74      1880
```

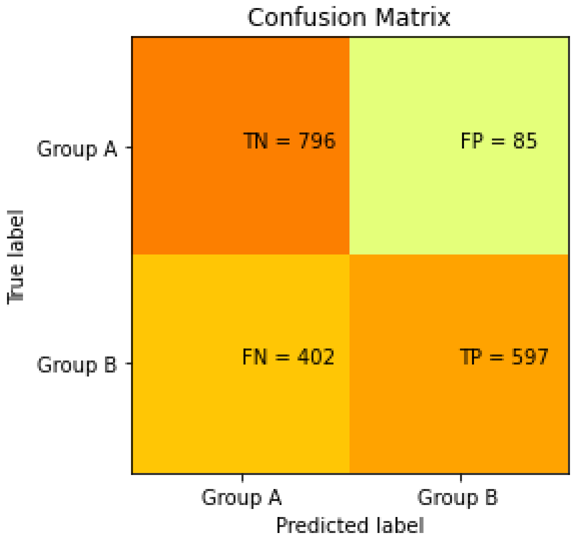


```
AUC Score : 0.7505581631574821

Accuracy Of the Model(MLPClassifier): 0.7409574468085106
```

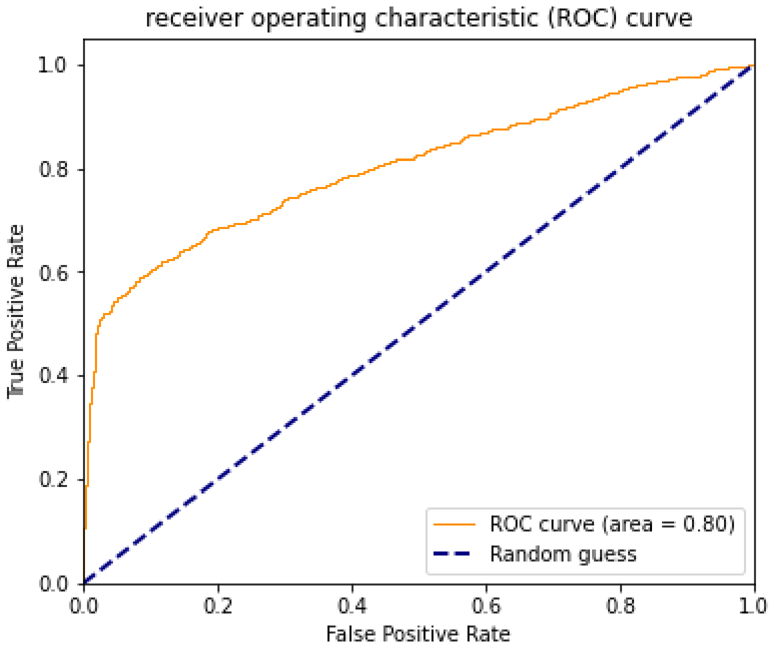

```
ROC_AUC Score of the model(MLPClassifier): 0.8046718682359999

[('RandomForestClassifier', 0.7446808510638298), ('XGBClassifier', 0.7441489361702
127), ('GradientBoostingClassifier', 0.7436170212765958), ('MLPClassifier', 0.7409
574468085106), ('LogisticRegression', 0.7356382978723405), ('AdaBoostClassifier',
0.7356382978723405), ('ExtraTreesClassifier', 0.7345744680851064), ('DecisionTreeC
lassifier', 0.7324468085106383), ('SVC', 0.7265957446808511), ('KNeighborsClassifi
er', 0.7154255319148937)]


sorted_total_auc:
 [('RandomForestClassifier', 0.8076044262196361), ('XGBClassifier', 0.804996824293
0785), ('MLPClassifier', 0.8046718682359999), ('GradientBoostingClassifier', 0.803
3902233675219), ('AdaBoostClassifier', 0.7998020722197794), ('LogisticRegression',
0.7988033436387579), ('ExtraTreesClassifier', 0.7960451938885537), ('KNeighborsCla
ssifier', 0.7897363879202697), ('DecisionTreeClassifier', 0.7842837161792893), ('S
VC', 0.7824964578653568)]
```

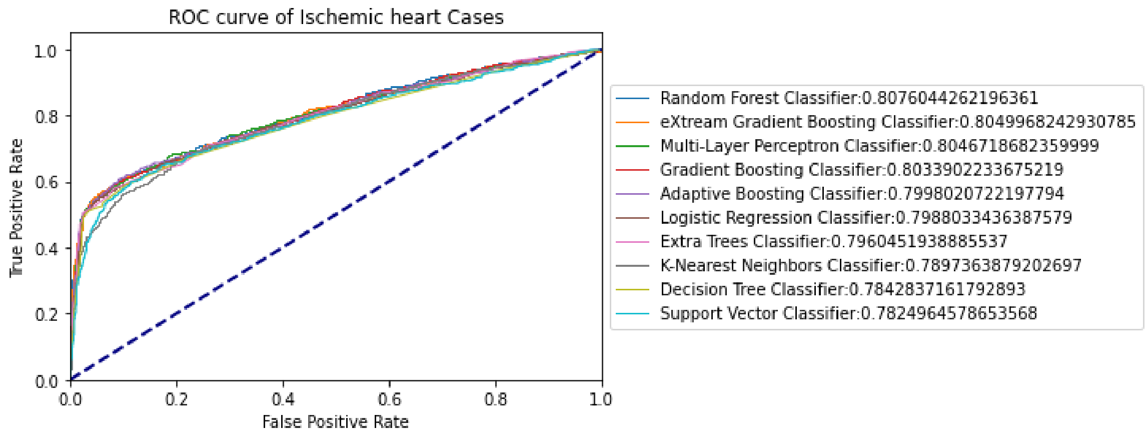

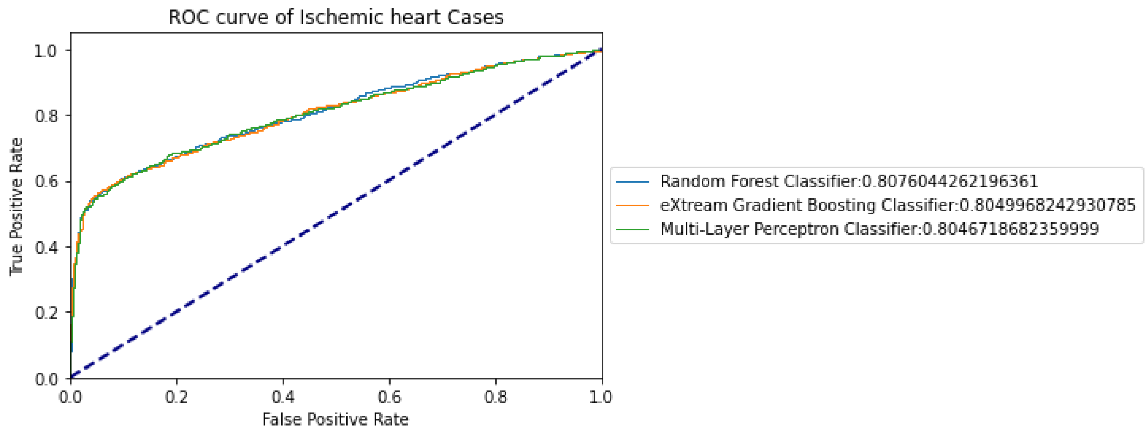

```
random state :   12465
9398
result_train_filename: data/train_test_data_ischemic heart_2013_20210519_125153.cs
v
total count : 9398, df_train count : 7518, df_test count : 1880
(7518, 38)
(1880, 38)
('Logistic Regression', LogisticRegression())
[2021-05-19 12:51:53.887082] Start parameter search for model 'Logistic Regressio
n'
[2021-05-19 12:51:54.233943] Finish parameter search for model 'Logistic Regressio
n' (time: 0 seconds)

=== train : best params ===
{'C': 0.1, 'penalty': 'l2', 'solver': 'lbfgs'}
=== train : best score : roc_auc ===
0.8038838241063713



('Random Forest', RandomForestClassifier())
[2021-05-19 12:51:54.233943] Start parameter search for model 'Random Forest'
[2021-05-19 12:51:58.787552] Finish parameter search for model 'Random Forest' (ti
me: 4 seconds)

=== train : best params ===
{'criterion': 'gini', 'max_depth': 8, 'max_features': 'auto', 'n_estimators': 90}
=== train : best score : roc_auc ===
0.8101512789745653



('Extreme Gradient Boosting)', XGBClassifier(base_score=None, booster=None, colsam
ple_bylevel=None,
              colsample_bynode=None, colsample_bytree=None, gamma=None,
              gpu_id=None, importance_type='gain', interaction_constraints=None,
              learning_rate=None, max_delta_step=None, max_depth=None,
              min_child_weight=None, missing=nan, monotone_constraints=None,
              n_estimators=100, n_jobs=None, num_parallel_tree=None,
              random_state=None, reg_alpha=None, reg_lambda=None,
              scale_pos_weight=None, subsample=None, tree_method=None,
              validate_parameters=None, verbosity=None))
[2021-05-19 12:51:58.787552] Start parameter search for model 'Extreme Gradient Bo
osting)'
[2021-05-19 12:52:25.650070] Finish parameter search for model 'Extreme Gradient B
oosting)' (time: 26 seconds)

=== train : best params ===
{'learning_rate': 0.1, 'max_depth': 2, 'n_estimators': 100, 'subsample': 0.8}
=== train : best score : roc_auc ===
0.8078543468322634



('k-Nearest Neighbours)', KNeighborsClassifier())
[2021-05-19 12:52:25.651068] Start parameter search for model 'k-Nearest Neighbour
s)'
[2021-05-19 12:52:33.589279] Finish parameter search for model 'k-Nearest Neighbou
rs)' (time: 7 seconds)

=== train : best params ===
{'n_neighbors': 60, 'weights': 'distance'}
=== train : best score : roc_auc ===
0.7919999375178606
```

```
('Decision Tree', DecisionTreeClassifier())
[2021-05-19 12:52:33.590277] Start parameter search for model 'Decision Tree'
[2021-05-19 12:52:34.155795] Finish parameter search for model 'Decision Tree' (ti
me: 0 seconds)

=== train : best params ===
{'criterion': 'entropy', 'max_depth': 4}
=== train : best score : roc_auc ===
0.7904299481618497


('Extra Tree', ExtraTreesClassifier())
[2021-05-19 12:52:34.156789] Start parameter search for model 'Extra Tree'
[2021-05-19 12:52:37.125153] Finish parameter search for model 'Extra Tree' (time:
2 seconds)

=== train : best params ===
{'max_features': 'auto', 'n_estimators': 100}
=== train : best score : roc_auc ===
0.8051035442480363


('Gradient Boosting', GradientBoostingClassifier())
[2021-05-19 12:52:37.126158] Start parameter search for model 'Gradient Boosting'
[2021-05-19 12:52:52.961261] Finish parameter search for model 'Gradient Boosting'
(time: 15 seconds)

=== train : best params ===
{'learning_rate': 0.01, 'max_depth': 8, 'max_features': 6, 'n_estimators': 100}
=== train : best score : roc_auc ===
0.8080540180767094


('Adaptive Boosting', AdaBoostClassifier())
[2021-05-19 12:52:52.961261] Start parameter search for model 'Adaptive Boosting'
[2021-05-19 12:52:56.589770] Finish parameter search for model 'Adaptive Boosting'
(time: 3 seconds)

=== train : best params ===
{'learning_rate': 0.2, 'n_estimators': 100}
=== train : best score : roc_auc ===
0.8025855309628107


('Suppport Vector Classfier', SVC(probability=True))
[2021-05-19 12:52:56.590767] Start parameter search for model 'Suppport Vector Cla
ssfier'
[2021-05-19 12:57:04.904425] Finish parameter search for model 'Suppport Vector Cl
assfier' (time: 248 seconds)

=== train : best params ===
{'C': 1, 'gamma': 0.1, 'kernel': 'rbf'}
=== train : best score : roc_auc ===
0.7864933898150988


('Neural Network', MLPClassifier())
[2021-05-19 12:57:04.905423] Start parameter search for model 'Neural Network'
[2021-05-19 12:59:44.925615] Finish parameter search for model 'Neural Network' (t
```

```
ime: 160 seconds)

=== train : best params ===
{'activation': 'logistic', 'alpha': 0.05, 'hidden_layer_sizes': (50,), 'learning_r
ate': 'adaptive'}
=== train : best score : roc_auc ===
0.8080210817722522



Confusion Matrix:
[[823  58]
 [428 571]]

Classification Report:
              precision    recall  f1-score   support

         0.0       0.66      0.93      0.77       881
         1.0       0.91      0.57      0.70       999

    accuracy                           0.74      1880
   macro avg       0.78      0.75      0.74      1880
weighted avg       0.79      0.74      0.73      1880
```

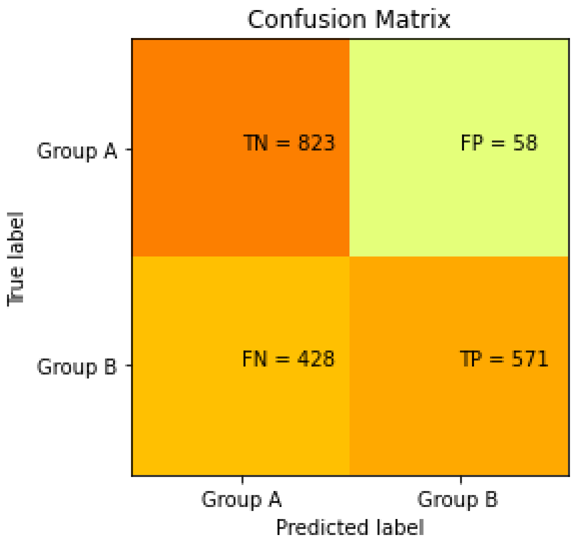


```
AUC Score : 0.7528686461717108

Accuracy Of the Model(LogisticRegression): 0.7414893617021276
```

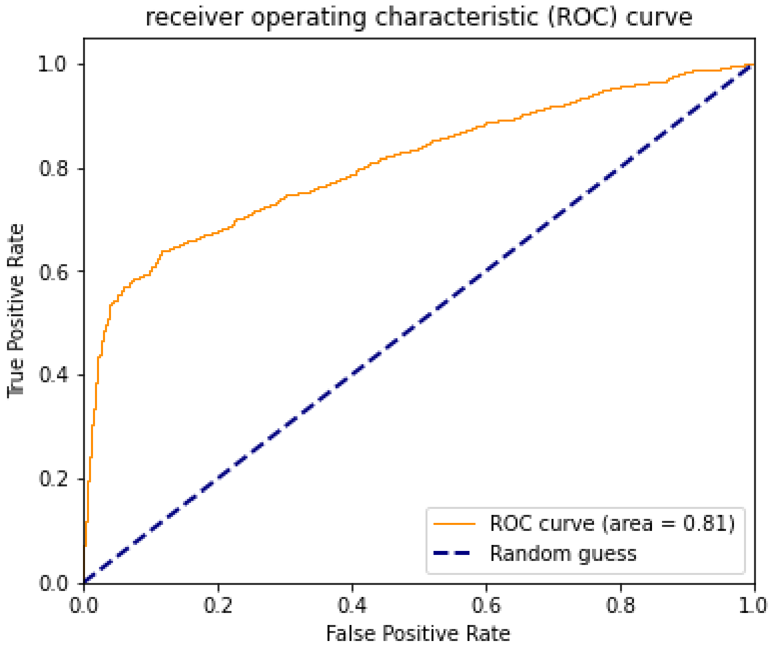


```
ROC_AUC Score of the model(LogisticRegression): 0.808473626861822


Confusion Matrix:
[[797  84]
 [401 598]]

Classification Report:
              precision    recall  f1-score   support

         0.0       0.67      0.90      0.77       881
         1.0       0.88      0.60      0.71       999

    accuracy                           0.74      1880
   macro avg       0.77      0.75      0.74      1880
weighted avg       0.78      0.74      0.74      1880
```

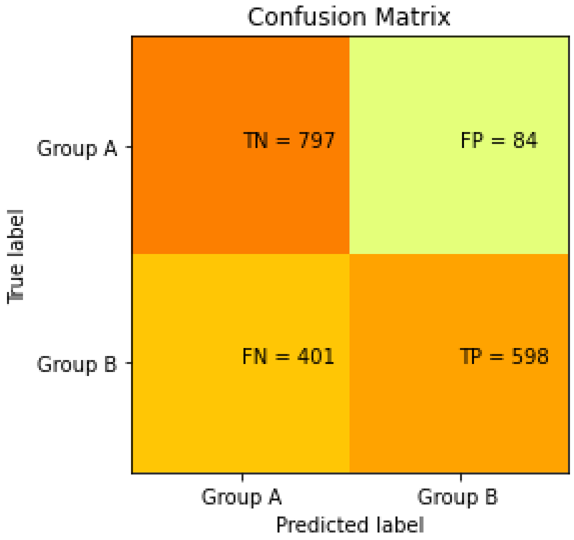

AUC Score : 0.7516262005478804

Accuracy Of the Model(RandomForestClassifier): 0.7420212765957447

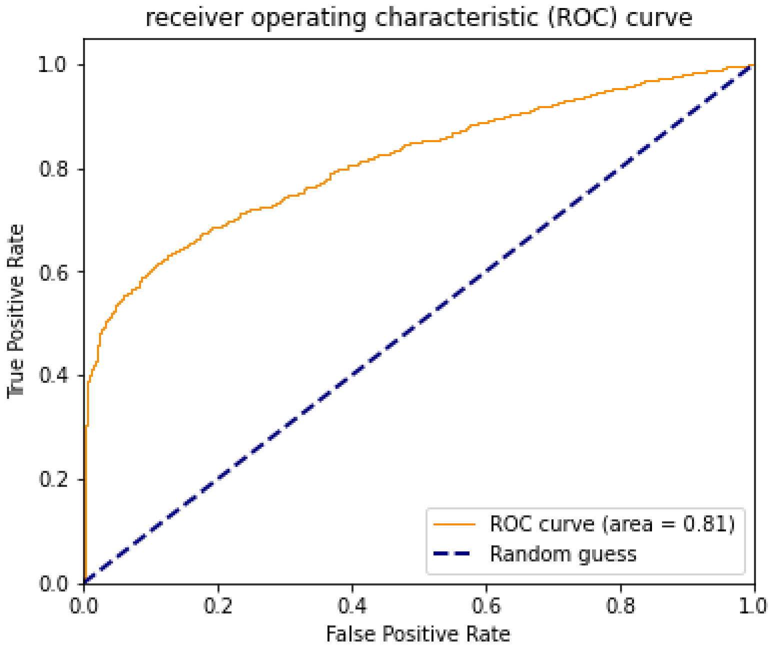

```
ROC_AUC Score of the model(RandomForestClassifier): 0.813844491483538


Confusion Matrix:
[[810  71]
 [413 586]]

Classification Report:
              precision    recall  f1-score   support

         0.0       0.66      0.92      0.77       881
         1.0       0.89      0.59      0.71       999

    accuracy                           0.74      1880
   macro avg       0.78      0.75      0.74      1880
weighted avg       0.78      0.74      0.74      1880
```

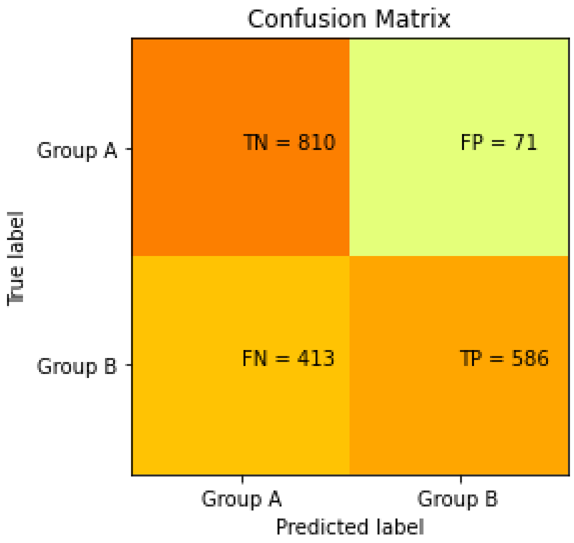


```
AUC Score : 0.7529981741105464

Accuracy Of the Model(XGBClassifier): 0.7425531914893617
```

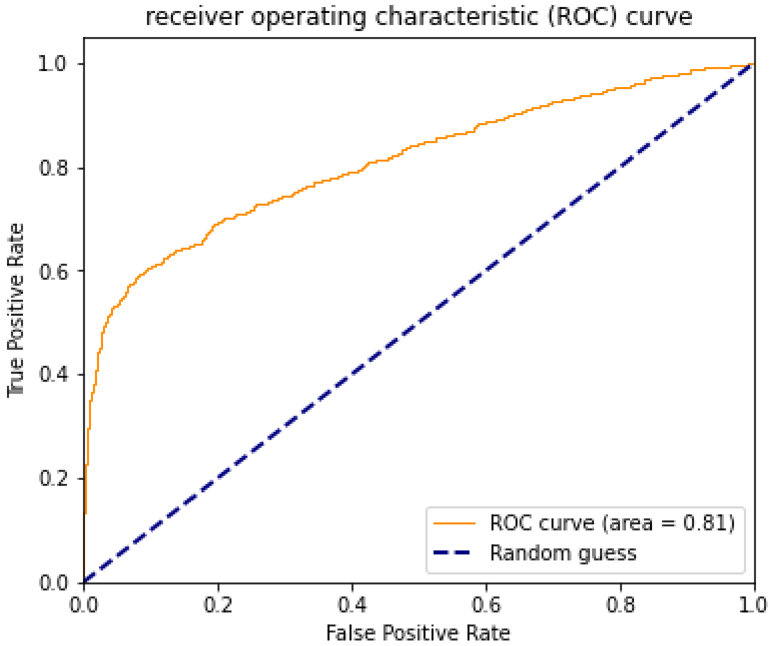

```
ROC_AUC Score of the model(XGBClassifier): 0.8119492932205759


Confusion Matrix:
[[799  82]
 [462 537]]

Classification Report:
              precision    recall  f1-score   support

         0.0       0.63      0.91      0.75       881
         1.0       0.87      0.54      0.66       999

    accuracy                           0.71      1880
   macro avg       0.75      0.72      0.70      1880
weighted avg       0.76      0.71      0.70      1880
```

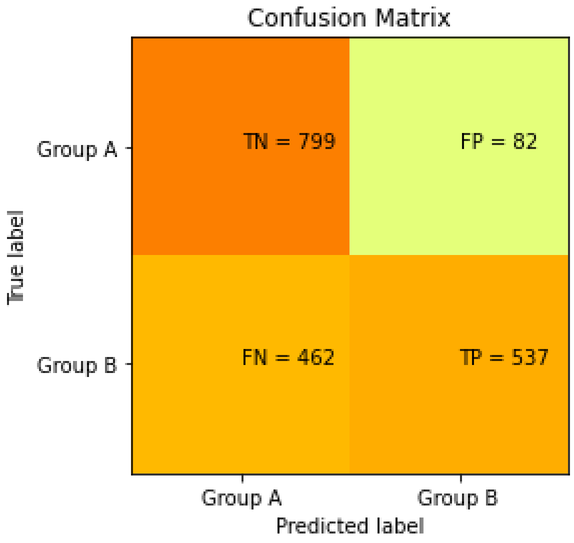


```
AUC Score : 0.7222307437971456

Accuracy Of the Model(KNeighborsClassifier): 0.7106382978723405
```

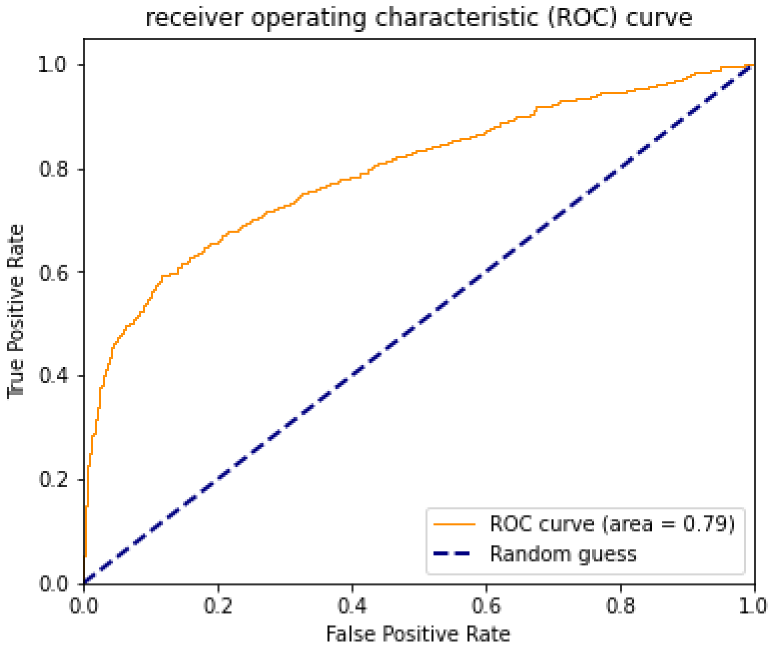

```
ROC_AUC Score of the model(KNeighborsClassifier): 0.7942721381994935


Confusion Matrix:
[[808  73]
 [428 571]]

Classification Report:
              precision    recall  f1-score   support

         0.0       0.65      0.92      0.76       881
         1.0       0.89      0.57      0.70       999

    accuracy                           0.73      1880
   macro avg       0.77      0.74      0.73      1880
weighted avg       0.78      0.73      0.73      1880
```

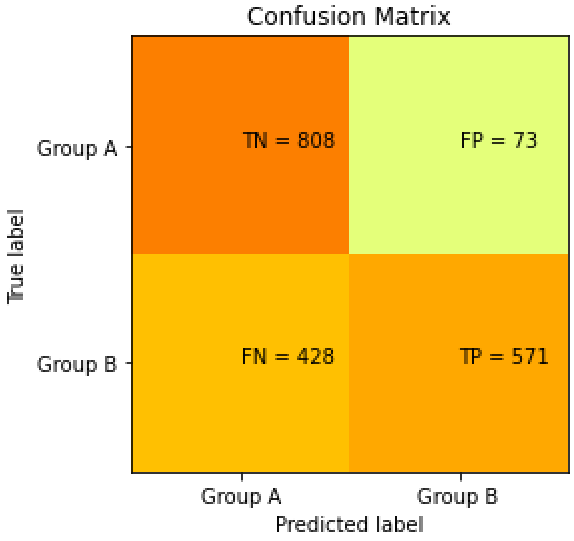


```
AUC Score : 0.7443555928232432

Accuracy Of the Model(DecisionTreeClassifier): 0.7335106382978723
```

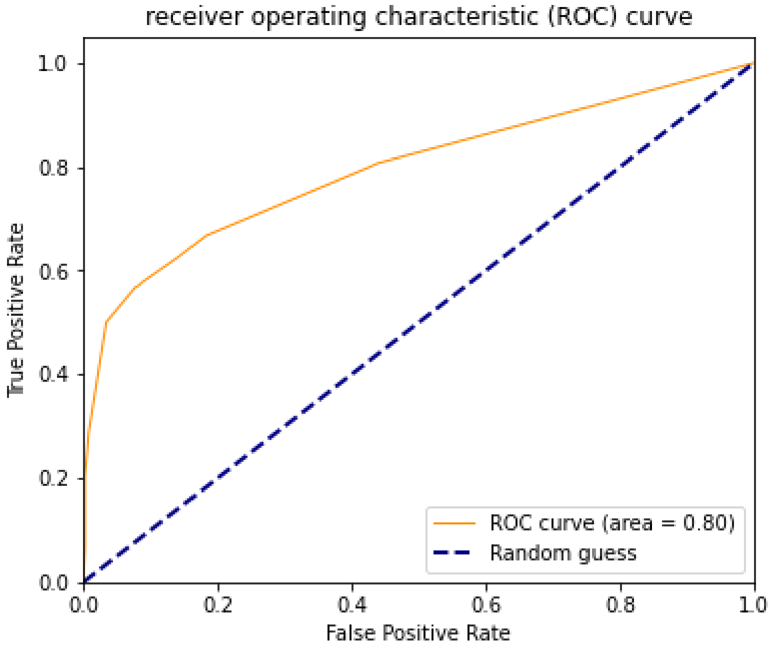

```
ROC_AUC Score of the model(DecisionTreeClassifier): 0.7956850153217916


Confusion Matrix:
[[790  91]
 [417 582]]

Classification Report:
              precision    recall  f1-score   support

         0.0       0.65      0.90      0.76       881
         1.0       0.86      0.58      0.70       999

    accuracy                           0.73      1880
   macro avg       0.76      0.74      0.73      1880
weighted avg       0.77      0.73      0.72      1880
```

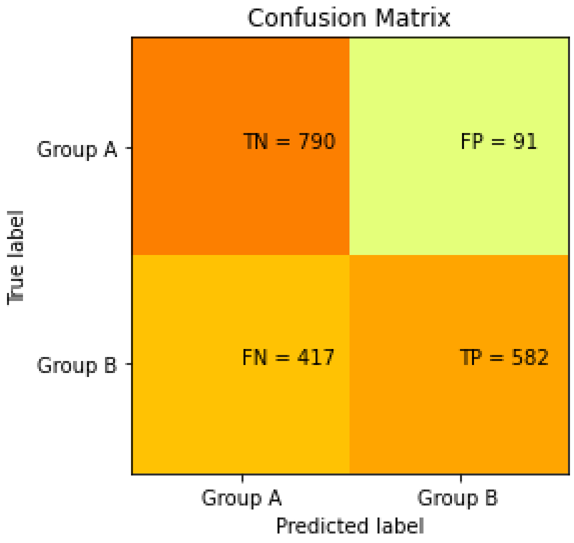


```
AUC Score : 0.7396454343105876

Accuracy Of the Model(ExtraTreesClassifier): 0.7297872340425532
```

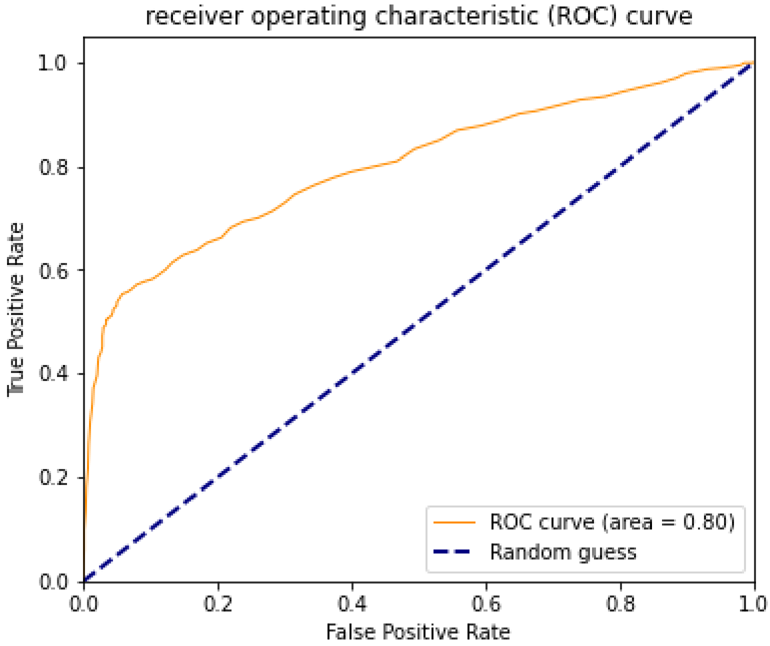

ROC_AUC Score of the model(ExtraTreesClassifier): 0.8012115407121082

Confusion Matrix:
[[802 79]
 [402 597]]

Classification Report:

	precision	recall	f1-score	support
0.0	0.67	0.91	0.77	881
1.0	0.88	0.60	0.71	999
accuracy			0.74	1880
macro avg	0.77	0.75	0.74	1880
weighted avg	0.78	0.74	0.74	1880

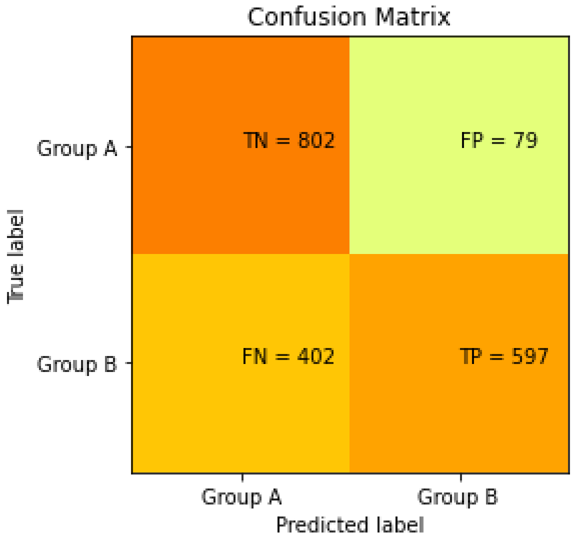

AUC Score : 0.7539633844968692

Accuracy Of the Model(GradientBoostingClassifier): 0.7441489361702127

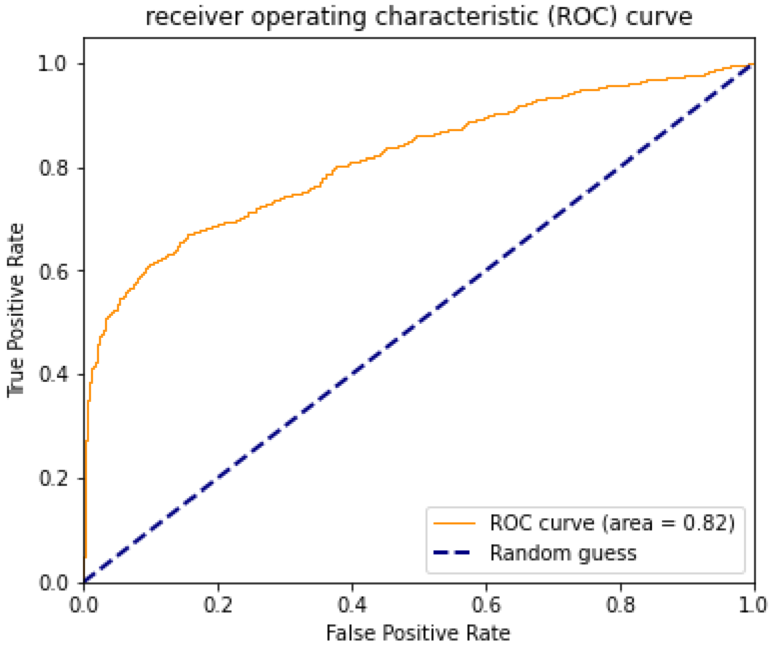

```
ROC_AUC Score of the model(GradientBoostingClassifier): 0.8171349556139568


Confusion Matrix:
[[816  65]
 [439 560]]

Classification Report:
              precision    recall  f1-score   support

         0.0       0.65      0.93      0.76       881
         1.0       0.90      0.56      0.69       999

    accuracy                           0.73      1880
   macro avg       0.77      0.74      0.73      1880
weighted avg       0.78      0.73      0.72      1880
```

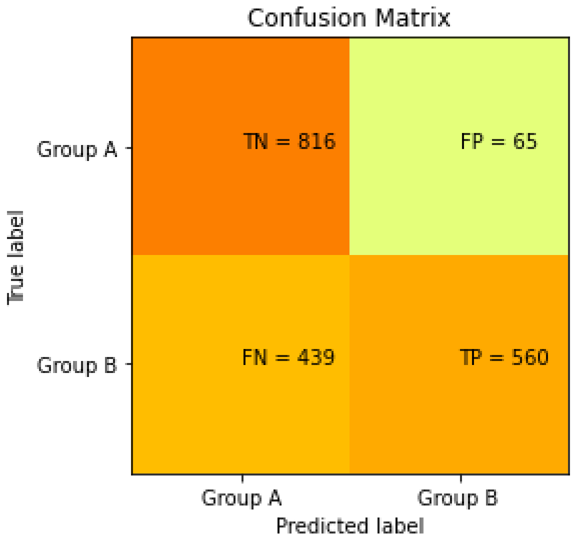


```
AUC Score : 0.7433903824369205

Accuracy Of the Model(AdaBoostClassifier): 0.7319148936170212
```

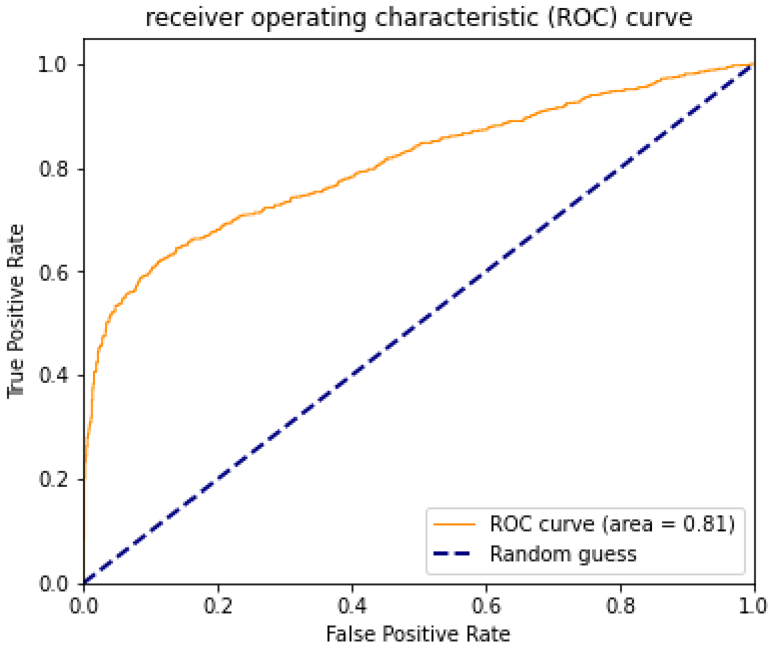

ROC_AUC Score of the model(AdaBoostClassifier): 0.8058370515805249

```
Confusion Matrix:
[[728 153]
 [348 651]]

Classification Report:
              precision    recall  f1-score   support

         0.0       0.68      0.83      0.74       881
         1.0       0.81      0.65      0.72       999

    accuracy                           0.73      1880
   macro avg       0.74      0.74      0.73      1880
weighted avg       0.75      0.73      0.73      1880
```

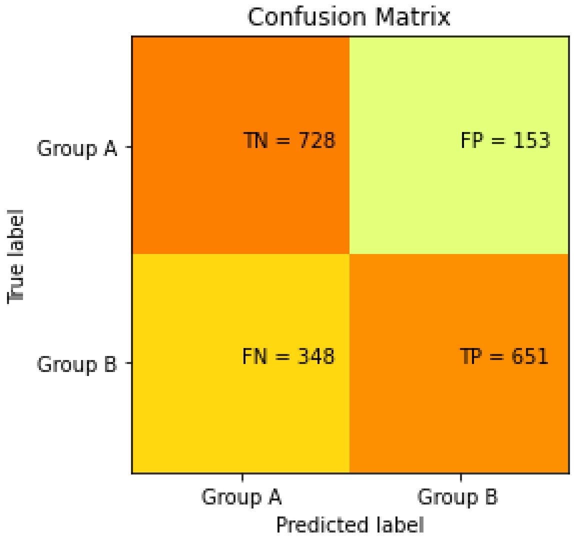

AUC Score : 0.7389926816714557

Accuracy Of the Model(SVC): 0.7335106382978723

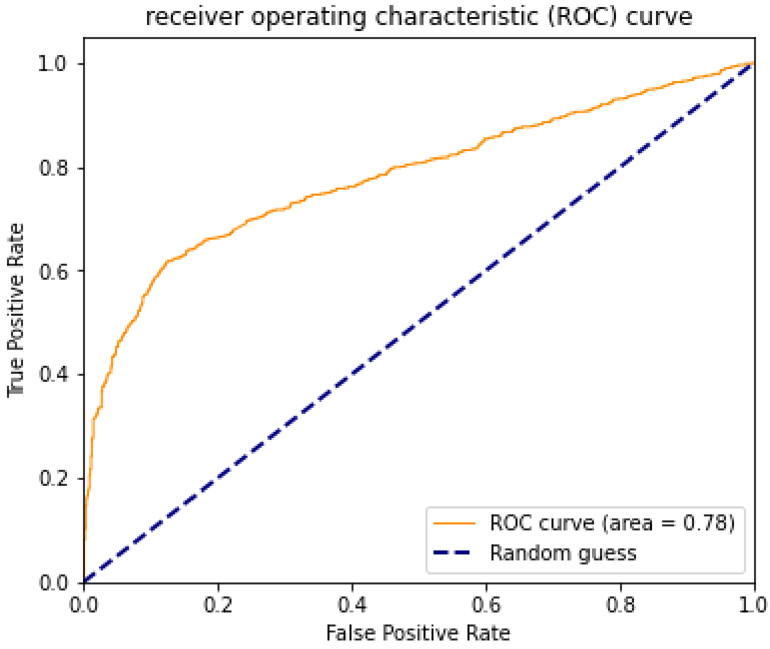

```
ROC_AUC Score of the model(SVC): 0.7808438404352138


Confusion Matrix:
[[799  82]
 [404 595]]

Classification Report:
              precision    recall  f1-score   support

         0.0       0.66      0.91      0.77       881
         1.0       0.88      0.60      0.71       999

    accuracy                           0.74      1880
   macro avg       0.77      0.75      0.74      1880
weighted avg       0.78      0.74      0.74      1880
```

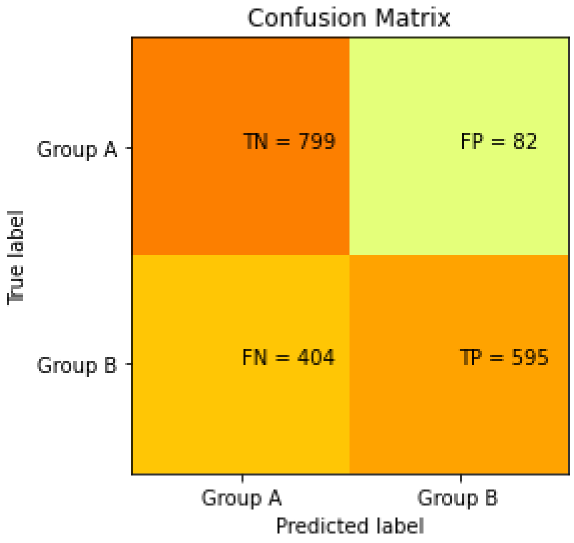


```
AUC Score : 0.7512597728261746

Accuracy Of the Model(MLPClassifier): 0.7414893617021276
```

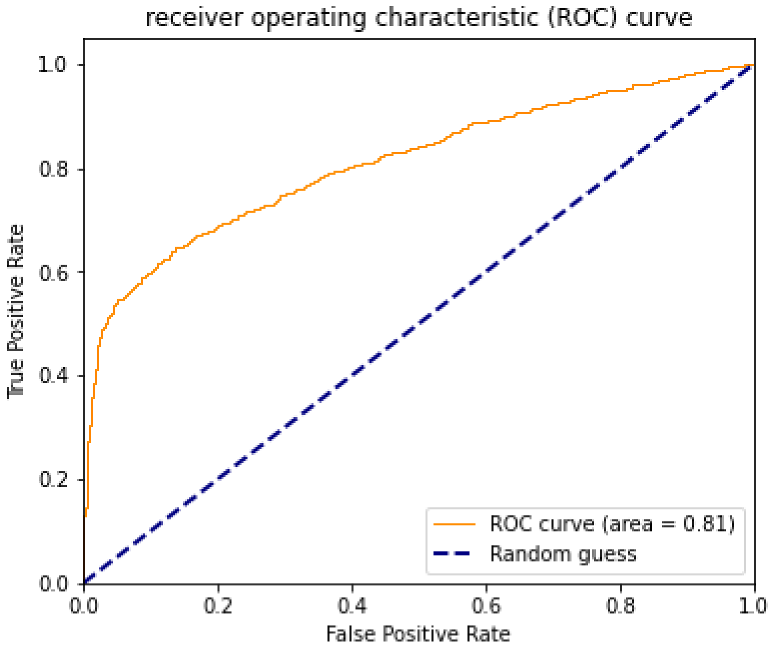

```
ROC_AUC Score of the model(MLPClassifier): 0.8124208203663368

[('GradientBoostingClassifier', 0.7441489361702127), ('XGBClassifier', 0.742553191
4893617), ('RandomForestClassifier', 0.7420212765957447), ('LogisticRegression',
0.7414893617021276), ('MLPClassifier', 0.7414893617021276), ('DecisionTreeClassifi
er', 0.7335106382978723), ('SVC', 0.7335106382978723), ('AdaBoostClassifier', 0.73
19148936170212), ('ExtraTreesClassifier', 0.7297872340425532), ('KNeighborsClassif
ier', 0.7106382978723405)]


sorted_total_auc:
 [('GradientBoostingClassifier', 0.8171349556139568), ('RandomForestClassifier',
0.813844491483538), ('MLPClassifier', 0.8124208203663368), ('XGBClassifier', 0.811
9492932205759), ('LogisticRegression', 0.808473626861822), ('AdaBoostClassifier',
0.8058370515805249), ('ExtraTreesClassifier', 0.8012115407121082), ('DecisionTreeC
lassifier', 0.7956850153217916), ('KNeighborsClassifier', 0.7942721381994935), ('S
VC', 0.7808438404352138)]
```

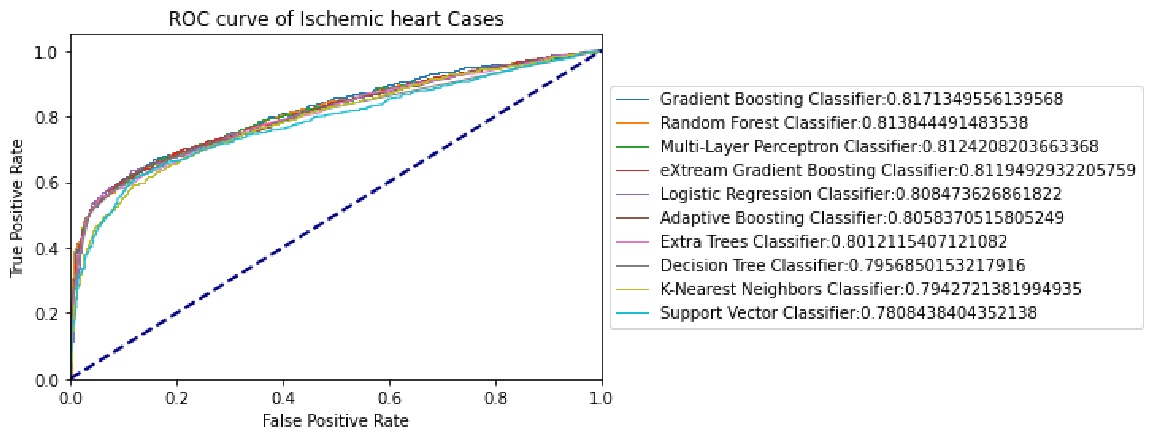

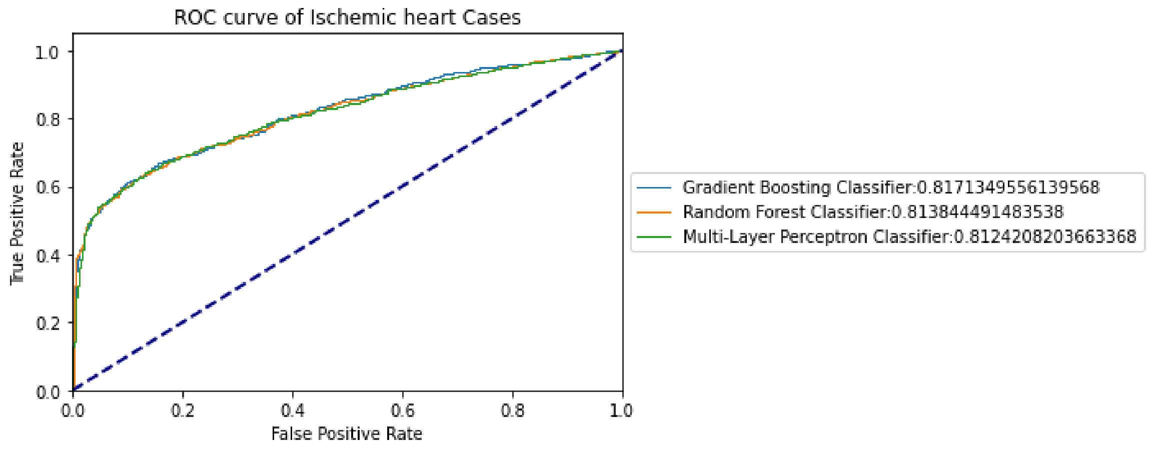

```
random state :  12481
9398
result_train_filename: data/train_test_data_ischemic heart_2013_20210519_125952.cs
v
total count : 9398, df_train count : 7518, df_test count : 1880
(7518, 38)
(1880, 38)
('Logistic Regression', LogisticRegression())
[2021-05-19 12:59:52.624296] Start parameter search for model 'Logistic Regressio
n'
[2021-05-19 12:59:52.833208] Finish parameter search for model 'Logistic Regressio
n' (time: 0 seconds)

=== train : best params ===
{'C': 0.1, 'penalty': 'l2', 'solver': 'lbfgs'}
=== train : best score : roc_auc ===
0.8051238876388



('Random Forest', RandomForestClassifier())
[2021-05-19 12:59:52.834206] Start parameter search for model 'Random Forest'
[2021-05-19 12:59:57.430028] Finish parameter search for model 'Random Forest' (ti
me: 4 seconds)

=== train : best params ===
{'criterion': 'gini', 'max_depth': 10, 'max_features': 'auto', 'n_estimators': 90}
=== train : best score : roc_auc ===
0.8133881958934903



('Extreme Gradient Boosting)', XGBClassifier(base_score=None, booster=None, colsam
ple_bylevel=None,
              colsample_bynode=None, colsample_bytree=None, gamma=None,
              gpu_id=None, importance_type='gain', interaction_constraints=None,
              learning_rate=None, max_delta_step=None, max_depth=None,
              min_child_weight=None, missing=nan, monotone_constraints=None,
              n_estimators=100, n_jobs=None, num_parallel_tree=None,
              random_state=None, reg_alpha=None, reg_lambda=None,
              scale_pos_weight=None, subsample=None, tree_method=None,
              validate_parameters=None, verbosity=None))
[2021-05-19 12:59:57.430995] Start parameter search for model 'Extreme Gradient Bo
osting)'
[2021-05-19 13:00:24.112708] Finish parameter search for model 'Extreme Gradient B
oosting)' (time: 26 seconds)

=== train : best params ===
{'learning_rate': 0.05, 'max_depth': 5, 'n_estimators': 50, 'subsample': 0.7}
=== train : best score : roc_auc ===
0.8143420180448694



('k-Nearest Neighbours)', KNeighborsClassifier())
[2021-05-19 13:00:24.114702] Start parameter search for model 'k-Nearest Neighbour
s)'
[2021-05-19 13:00:31.983407] Finish parameter search for model 'k-Nearest Neighbou
rs)' (time: 7 seconds)

=== train : best params ===
{'n_neighbors': 60, 'weights': 'distance'}
=== train : best score : roc_auc ===
0.78703275218495O8
```

```
('Decision Tree', DecisionTreeClassifier())
[2021-05-19 13:00:31.984406] Start parameter search for model 'Decision Tree'
[2021-05-19 13:00:32.565357] Finish parameter search for model 'Decision Tree' (ti
me: 0 seconds)

=== train : best params ===
{'criterion': 'entropy', 'max_depth': 4}
=== train : best score : roc_auc ===
0.7925708183951045


('Extra Tree', ExtraTreesClassifier())
[2021-05-19 13:00:32.566352] Start parameter search for model 'Extra Tree'
[2021-05-19 13:00:35.581654] Finish parameter search for model 'Extra Tree' (time:
3 seconds)

=== train : best params ===
{'max_features': 'auto', 'n_estimators': 100}
=== train : best score : roc_auc ===
0.8045432102131465


('Gradient Boosting', GradientBoostingClassifier())
[2021-05-19 13:00:35.583062] Start parameter search for model 'Gradient Boosting'
[2021-05-19 13:00:51.459566] Finish parameter search for model 'Gradient Boosting'
(time: 15 seconds)

=== train : best params ===
{'learning_rate': 0.01, 'max_depth': 8, 'max_features': 6, 'n_estimators': 100}
=== train : best score : roc_auc ===
0.813842372341889


('Adaptive Boosting', AdaBoostClassifier())
[2021-05-19 13:00:51.460564] Start parameter search for model 'Adaptive Boosting'
[2021-05-19 13:00:54.969266] Finish parameter search for model 'Adaptive Boosting'
(time: 3 seconds)

=== train : best params ===
{'learning_rate': 0.2, 'n_estimators': 100}
=== train : best score : roc_auc ===
0.8054229949163411


('Suppport Vector Classfier', SVC(probability=True))
[2021-05-19 13:00:54.969266] Start parameter search for model 'Suppport Vector Cla
ssfier'
[2021-05-19 13:05:00.466920] Finish parameter search for model 'Suppport Vector Cl
assfier' (time: 245 seconds)

=== train : best params ===
{'C': 1, 'gamma': 0.1, 'kernel': 'rbf'}
=== train : best score : roc_auc ===
0.7809701194230225


('Neural Network', MLPClassifier())
[2021-05-19 13:05:00.466920] Start parameter search for model 'Neural Network'
[2021-05-19 13:07:38.821589] Finish parameter search for model 'Neural Network' (t
```

```
ime: 158 seconds)

=== train : best params ===
{'activation': 'logistic', 'alpha': 0.05, 'hidden_layer_sizes': (50,), 'learning_r
ate': 'adaptive'}
=== train : best score : roc_auc ===
0.8088383794097895



Confusion Matrix:
[[820  61]
 [426 573]]

Classification Report:
              precision    recall  f1-score   support

         0.0       0.66      0.93      0.77       881
         1.0       0.90      0.57      0.70       999

    accuracy                           0.74      1880
   macro avg       0.78      0.75      0.74      1880
weighted avg       0.79      0.74      0.73      1880
```

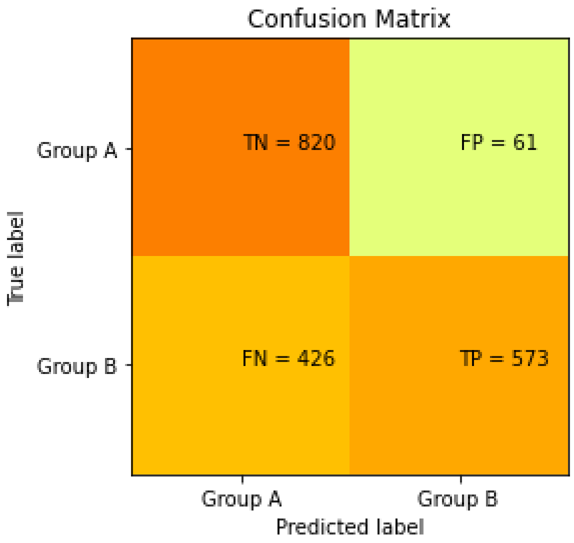


```
AUC Score : 0.7521670365030183

Accuracy Of the Model(LogisticRegression): 0.7409574468085106
```

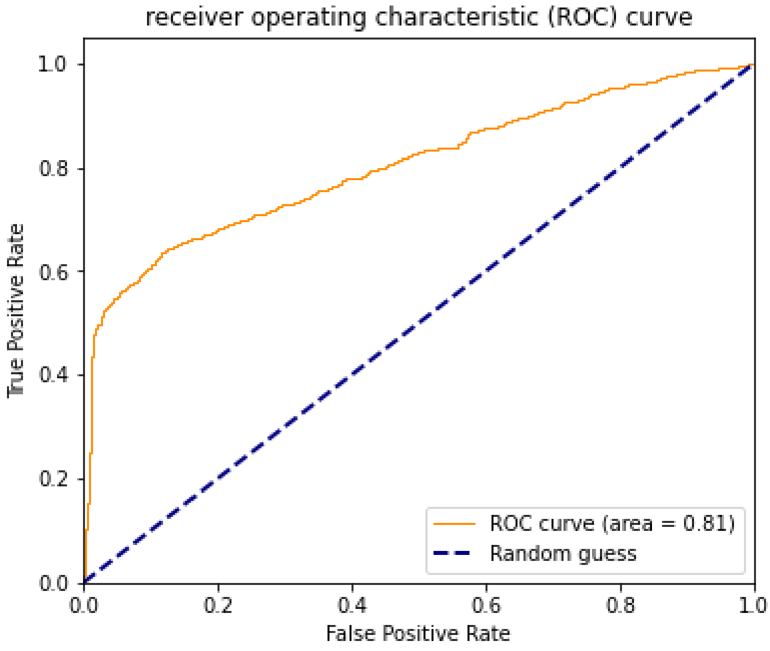

ROC_AUC Score of the model(LogisticRegression): 0.8050797676223329

```
Confusion Matrix:
[[800  81]
 [402 597]]

Classification Report:
              precision    recall  f1-score   support

         0.0       0.67      0.91      0.77       881
         1.0       0.88      0.60      0.71       999

    accuracy                           0.74      1880
   macro avg       0.77      0.75      0.74      1880
weighted avg       0.78      0.74      0.74      1880
```

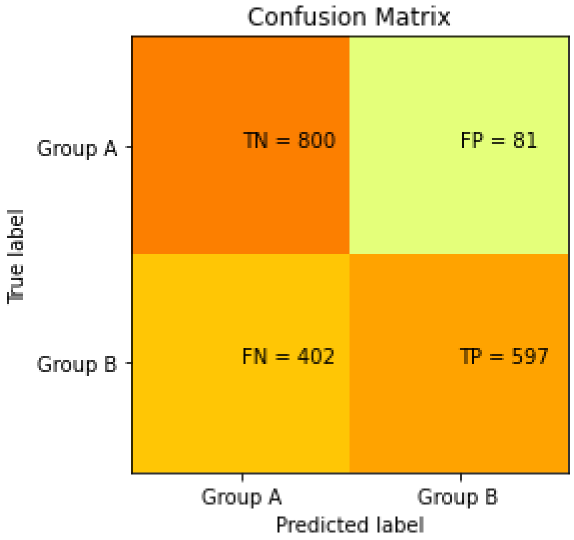


```
AUC Score : 0.7528283107170735

Accuracy Of the Model(RandomForestClassifier): 0.7430851063829788
```

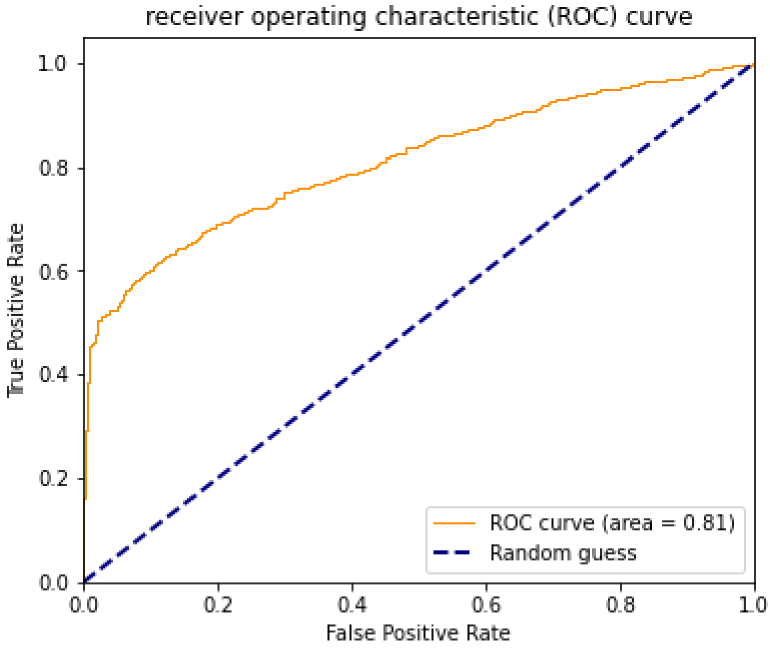

```
ROC_AUC Score of the model(RandomForestClassifier): 0.8123287873571642


Confusion Matrix:
[[823  58]
 [425 574]]

Classification Report:
              precision    recall  f1-score   support

         0.0       0.66      0.93      0.77       881
         1.0       0.91      0.57      0.70       999

    accuracy                           0.74      1880
   macro avg       0.78      0.75      0.74      1880
weighted avg       0.79      0.74      0.74      1880
```

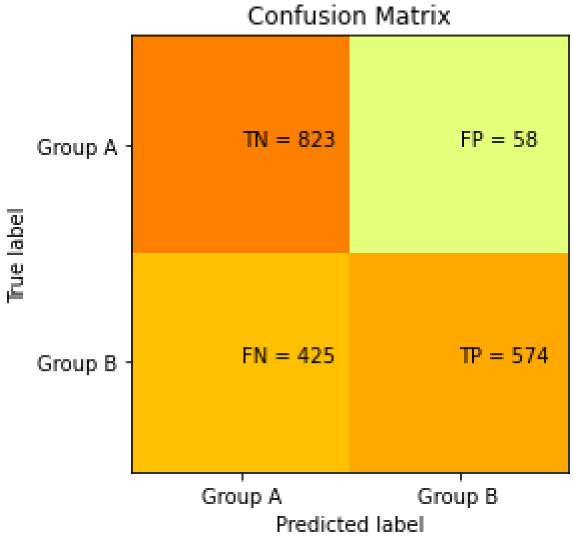


```
AUC Score : 0.7543701476732124

Accuracy Of the Model(XGBClassifier): 0.7430851063829788
```

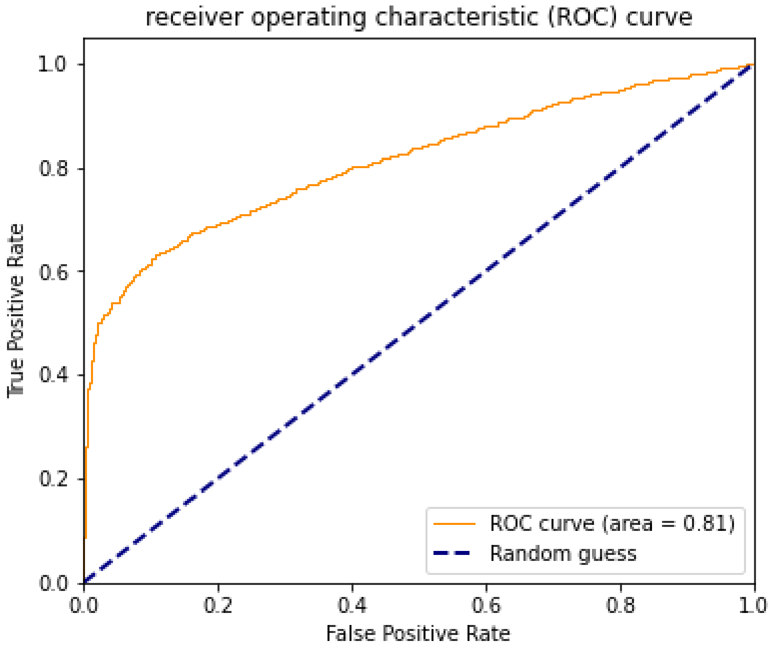

```
ROC_AUC Score of the model(XGBClassifier): 0.8133491039279916


Confusion Matrix:
[[801  80]
 [456 543]]

Classification Report:
              precision    recall  f1-score   support

         0.0       0.64      0.91      0.75       881
         1.0       0.87      0.54      0.67       999

    accuracy                           0.71      1880
   macro avg       0.75      0.73      0.71      1880
weighted avg       0.76      0.71      0.71      1880
```

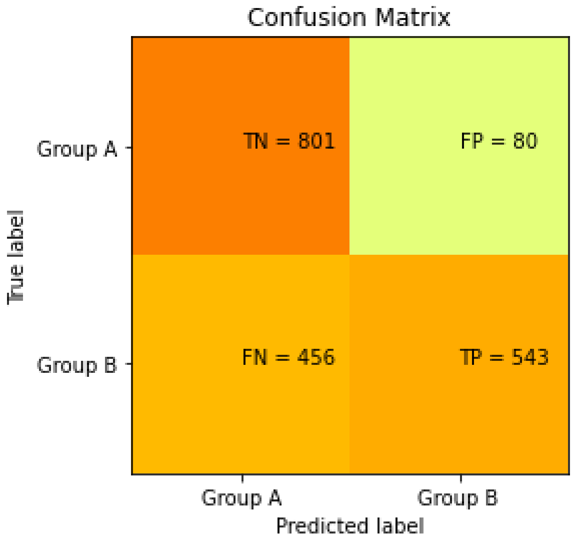


```
AUC Score : 0.7263688205799443

Accuracy Of the Model(KNeighborsClassifier): 0.7148936170212766
```

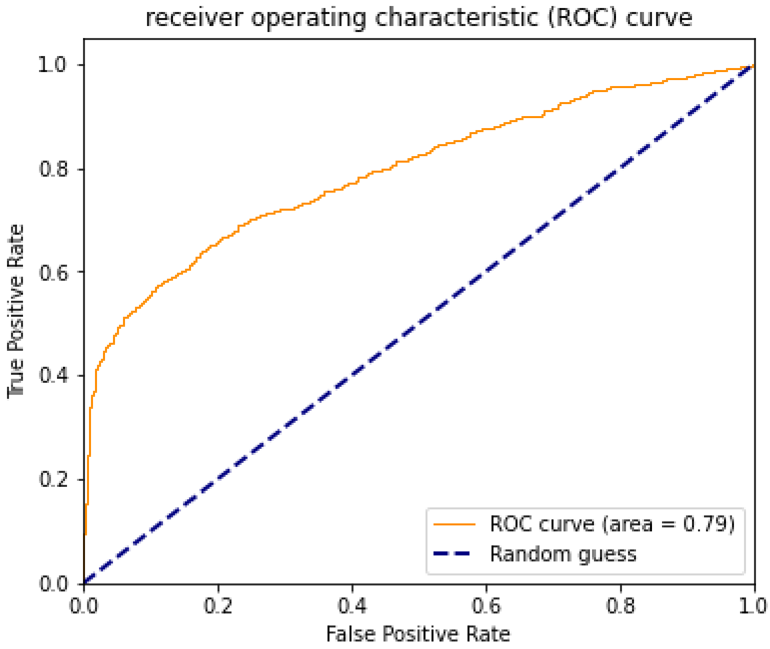

```
ROC_AUC Score of the model(KNeighborsClassifier): 0.7942119190700347


Confusion Matrix:
[[803  78]
 [424 575]]

Classification Report:
              precision    recall  f1-score   support

         0.0       0.65      0.91      0.76       881
         1.0       0.88      0.58      0.70       999

    accuracy                           0.73      1880
   macro avg       0.77      0.74      0.73      1880
weighted avg       0.77      0.73      0.73      1880
```

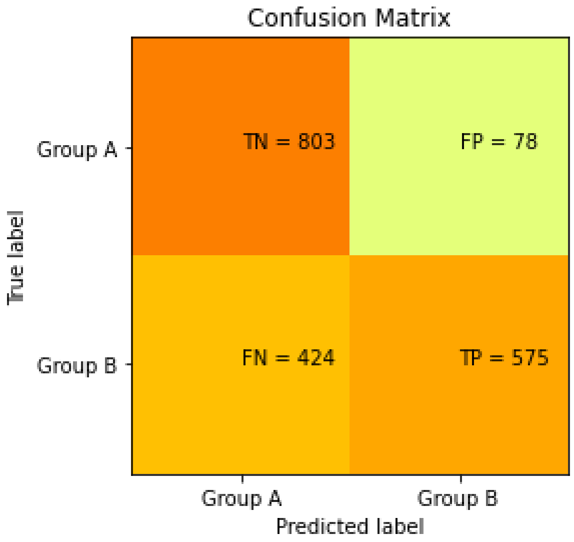


```
AUC Score : 0.7435199103757 56

Accuracy Of the Model(DecisionTreeClassifier): 0.7329787234042553
```

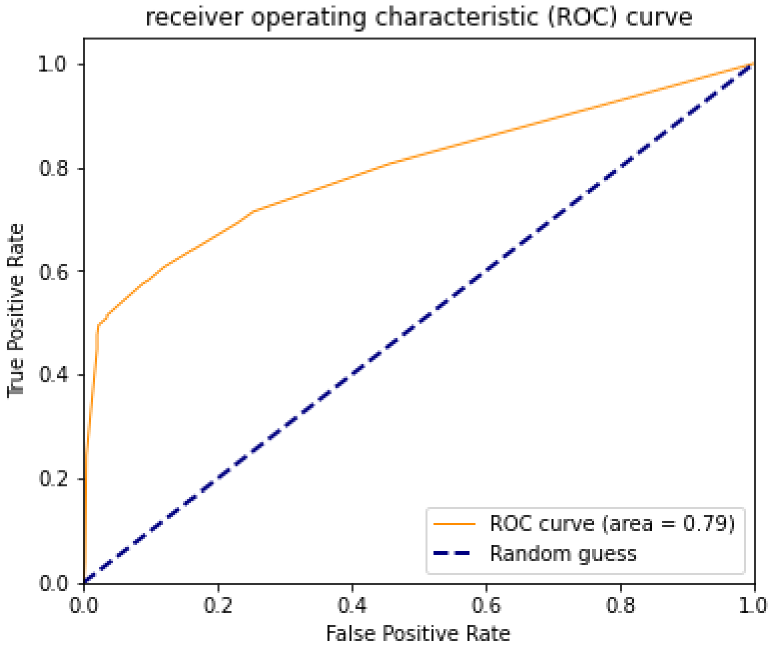

```
ROC_AUC Score of the model(DecisionTreeClassifier): 0.7944624533727825


Confusion Matrix:
[[801  80]
 [415 584]]

Classification Report:
              precision    recall  f1-score   support

         0.0       0.66      0.91      0.76       881
         1.0       0.88      0.58      0.70       999

    accuracy                           0.74      1880
   macro avg       0.77      0.75      0.73      1880
weighted avg       0.78      0.74      0.73      1880
```

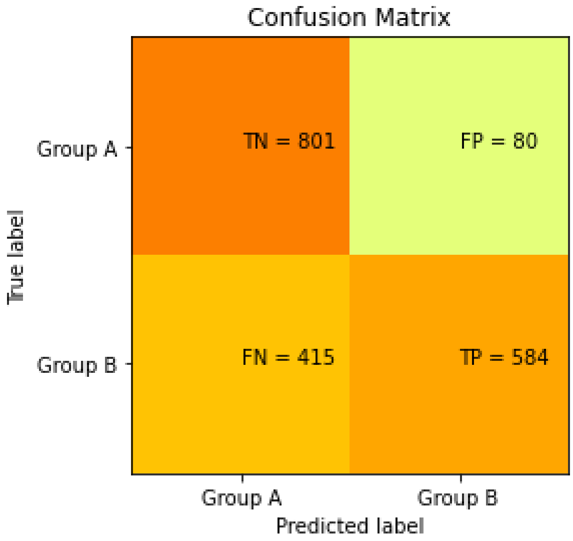


```
AUC Score : 0.7468893411004649

Accuracy Of the Model(ExtraTreesClassifier): 0.7367021276595744
```

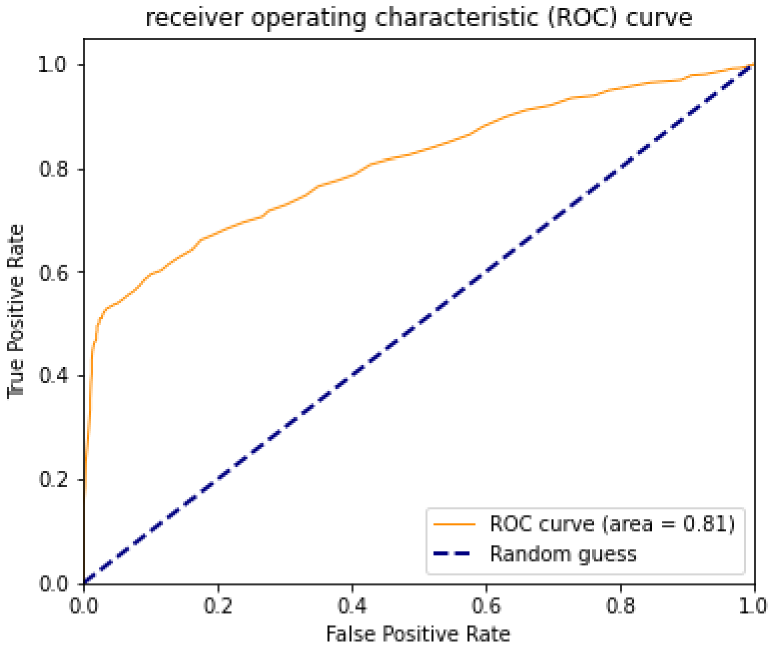

```
ROC_AUC Score of the model(ExtraTreesClassifier): 0.8061443963827619


Confusion Matrix:
[[807  74]
 [410 589]]

Classification Report:
              precision    recall  f1-score   support

         0.0       0.66      0.92      0.77       881
         1.0       0.89      0.59      0.71       999

    accuracy                           0.74      1880
   macro avg       0.78      0.75      0.74      1880
weighted avg       0.78      0.74      0.74      1880
```

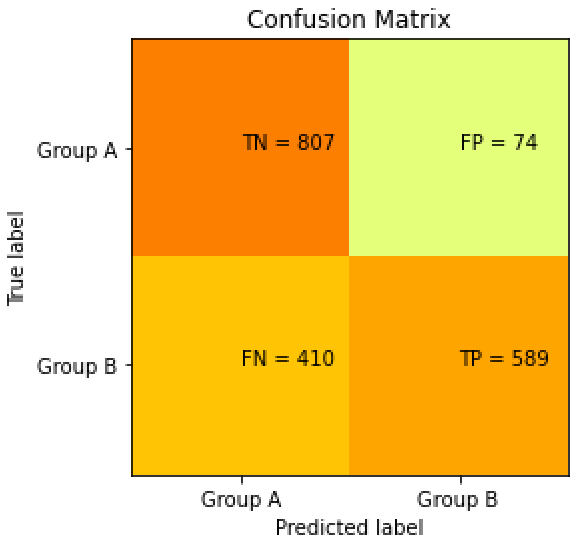


```
AUC Score : 0.7527970649423544

Accuracy Of the Model(GradientBoostingClassifier): 0.7425531914893617
```

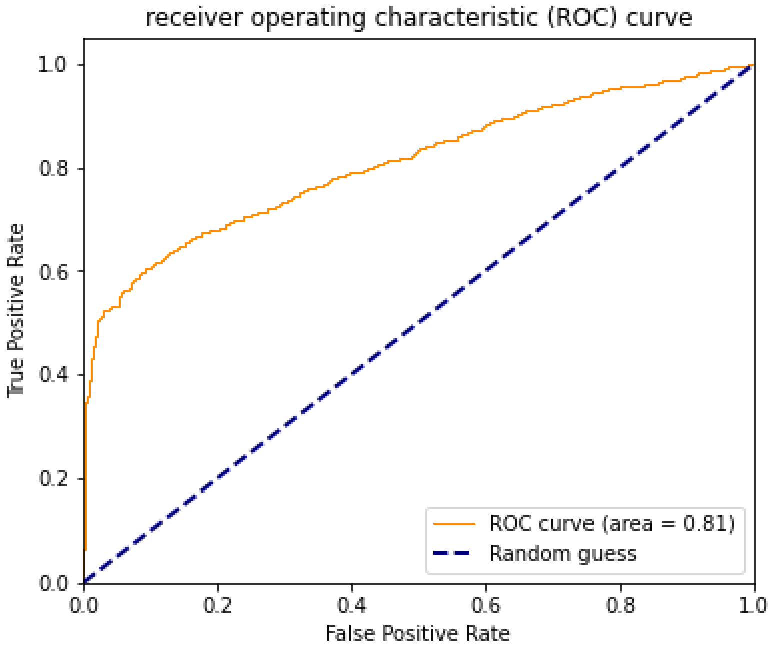

```
ROC_AUC Score of the model(GradientBoostingClassifier): 0.8099382015386556


Confusion Matrix:
[[818  63]
 [430 569]]

Classification Report:
              precision    recall  f1-score   support

         0.0       0.66      0.93      0.77       881
         1.0       0.90      0.57      0.70       999

    accuracy                           0.74      1880
   macro avg       0.78      0.75      0.73      1880
weighted avg       0.79      0.74      0.73      1880
```

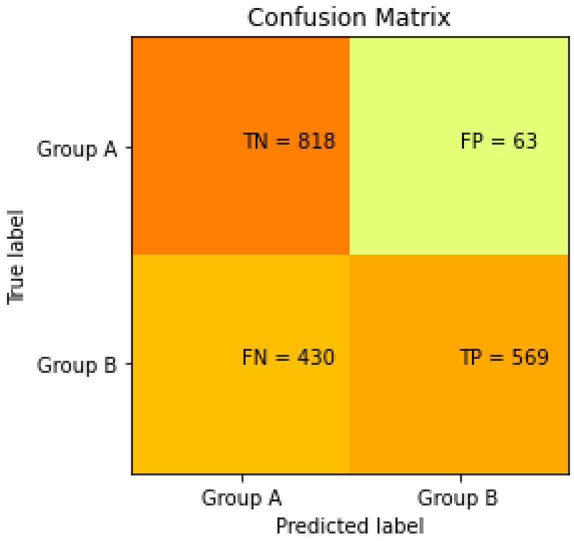


```
AUC Score : 0.7490299607212207

Accuracy Of the Model(AdaBoostClassifier): 0.7377659574468085
```

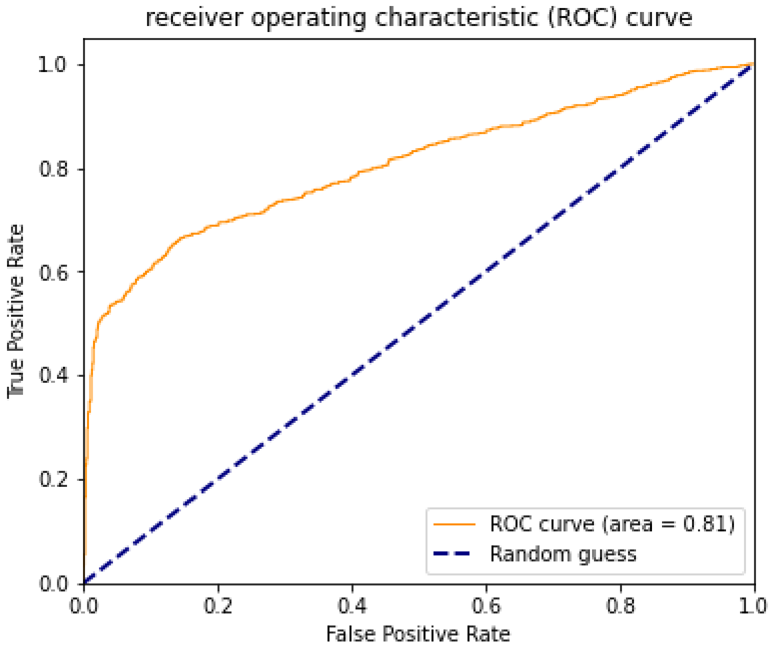

```
ROC_AUC Score of the model(AdaBoostClassifier): 0.8061074695580939


Confusion Matrix:
[[707 174]
 [343 656]]

Classification Report:
              precision    recall  f1-score   support

         0.0       0.67      0.80      0.73       881
         1.0       0.79      0.66      0.72       999

    accuracy                           0.73      1880
   macro avg       0.73      0.73      0.72      1880
weighted avg       0.74      0.72      0.72      1880
```

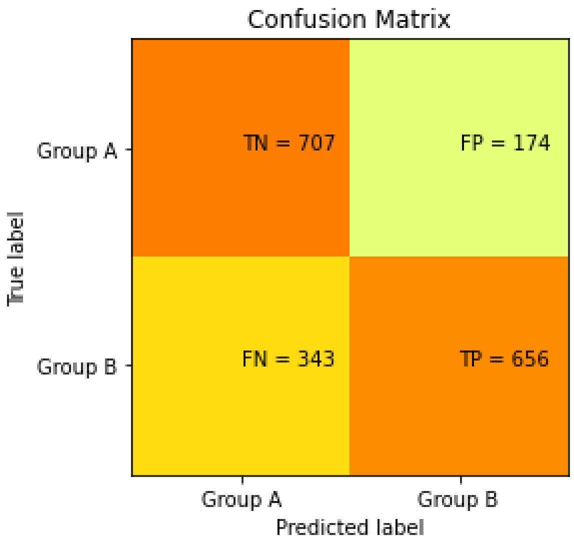


```
AUC Score : 0.7295769094861037

Accuracy Of the Model(SVC): 0.725
```

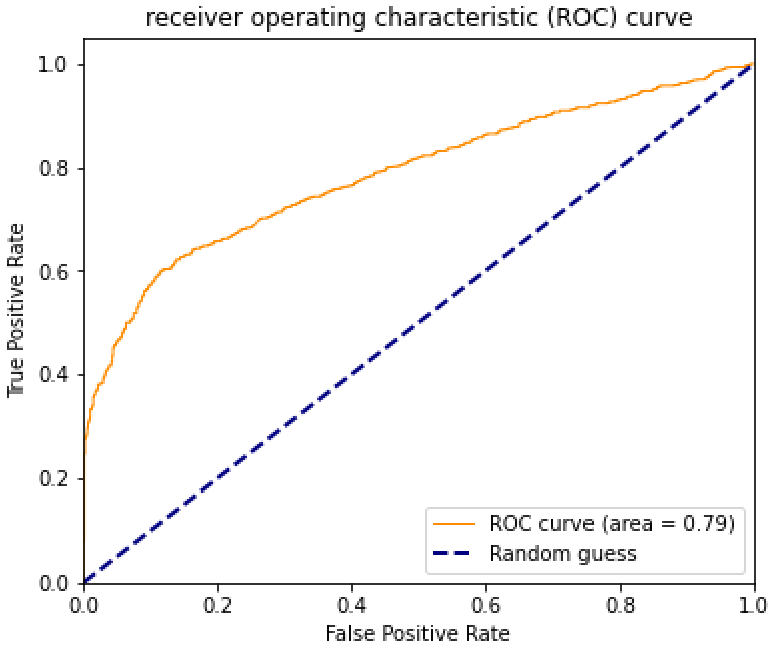

```
ROC_AUC Score of the model(SVC): 0.7865402292190034


Confusion Matrix:
[[810  71]
 [416 583]]

Classification Report:
              precision    recall  f1-score   support

         0.0       0.66      0.92      0.77       881
         1.0       0.89      0.58      0.71       999

    accuracy                           0.74      1880
   macro avg       0.78      0.75      0.74      1880
weighted avg       0.78      0.74      0.74      1880
```

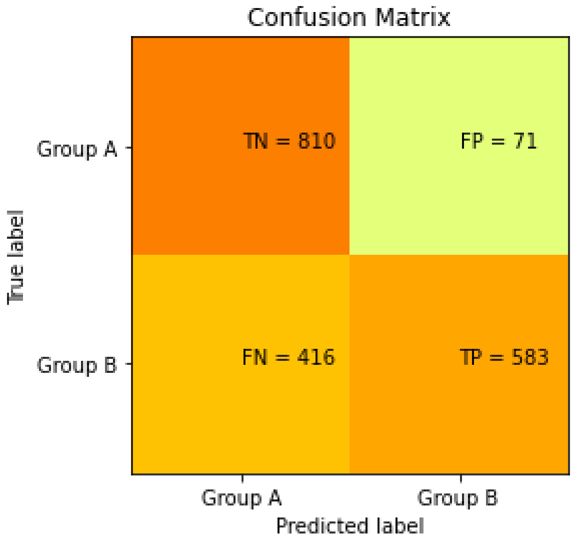


```
AUC Score : 0.7514966726090450

Accuracy Of the Model(MLPClassifier): 0.7409574468085106
```

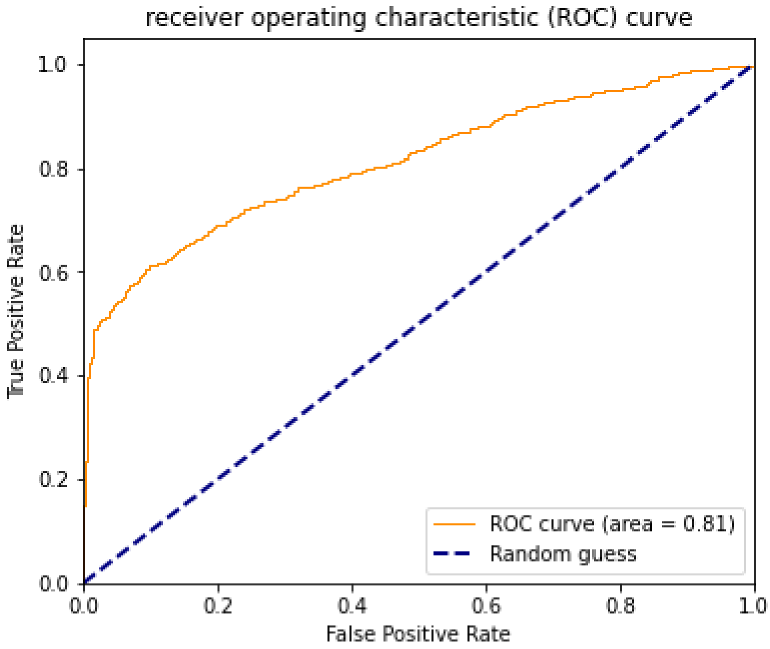

ROC_AUC Score of the model(MLPClassifier): 0.8132423001889517

[('RandomForestClassifier', 0.7430851063829788), ('XGBClassifier', 0.7430851063829788), ('GradientBoostingClassifier', 0.7425531914893617), ('LogisticRegression', 0.7409574468085106), ('MLPClassifier', 0.7409574468085106), ('AdaBoostClassifier', 0.7377659574468085), ('ExtraTreesClassifier', 0.7367021276595744), ('DecisionTreeClassifier', 0.7329787234042553), ('SVC', 0.725), ('KNeighborsClassifier', 0.7148936170212766)]

sorted_total_auc:
[('XGBClassifier', 0.8133491039279916), ('MLPClassifier', 0.8132423001889517), ('RandomForestClassifier', 0.8123287873571642), ('GradientBoostingClassifier', 0.8099382015386556), ('ExtraTreesClassifier', 0.8061443963827619), ('AdaBoostClassifier', 0.8061074695580939), ('LogisticRegression', 0.8050797676223329), ('DecisionTreeClassifier', 0.7944624533727825), ('KNeighborsClassifier', 0.7942119190700347), ('SVC', 0.7865402292190034)]

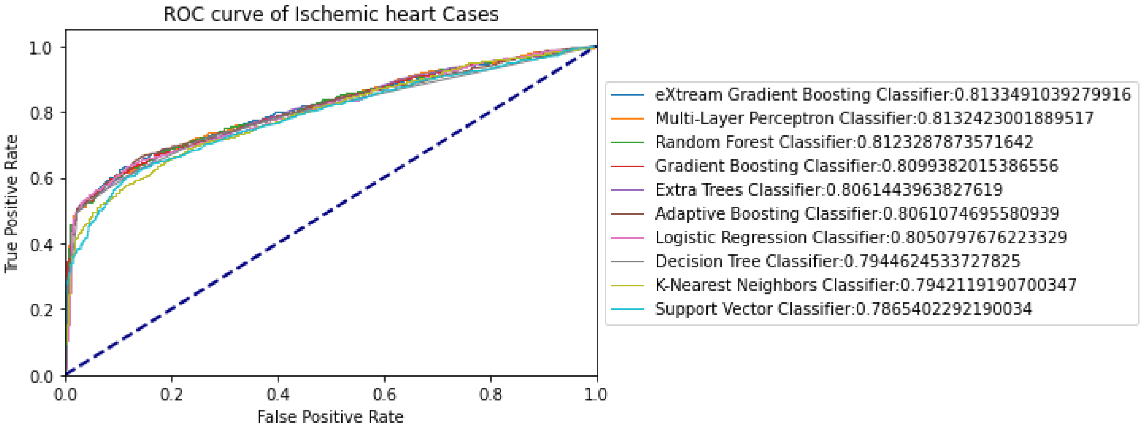

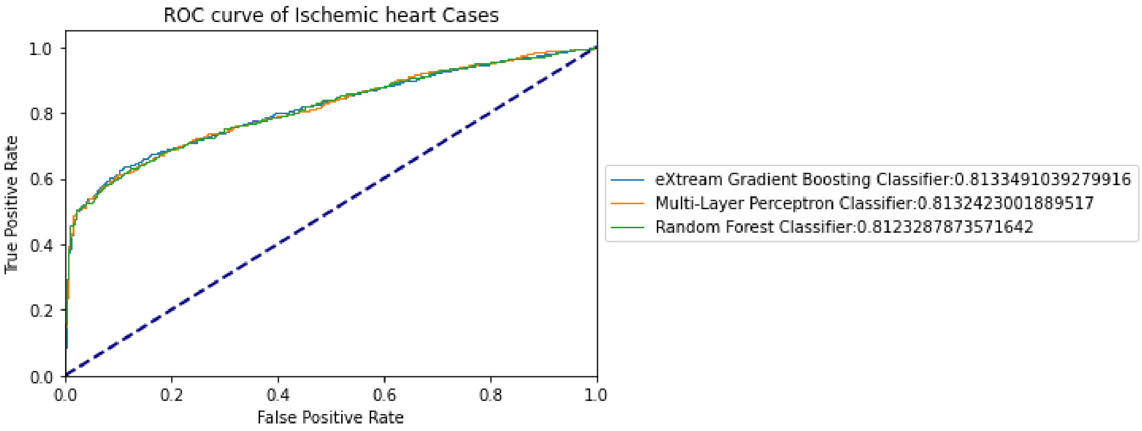

```
random state :   12498
9398
result_train_filename: data/train_test_data_ischemic heart_2013_20210519_130746.cs
v
total count : 9398, df_train count : 7518, df_test count : 1880
(7518, 38)
(1880, 38)
('Logistic Regression', LogisticRegression())
[2021-05-19 13:07:46.546406] Start parameter search for model 'Logistic Regressio
n'
[2021-05-19 13:07:46.874056] Finish parameter search for model 'Logistic Regressio
n' (time: 0 seconds)

=== train : best params ===
{'C': 10, 'penalty': 'l2', 'solver': 'lbfgs'}
=== train : best score : roc_auc ===
0.8043540274181171



('Random Forest', RandomForestClassifier())
[2021-05-19 13:07:46.875058] Start parameter search for model 'Random Forest'
[2021-05-19 13:07:51.702570] Finish parameter search for model 'Random Forest' (ti
me: 4 seconds)

=== train : best params ===
{'criterion': 'gini', 'max_depth': 8, 'max_features': 'auto', 'n_estimators': 100}
=== train : best score : roc_auc ===
0.8110016963848317



('Extreme Gradient Boosting)', XGBClassifier(base_score=None, booster=None, colsam
ple_bylevel=None,
              colsample_bynode=None, colsample_bytree=None, gamma=None,
              gpu_id=None, importance_type='gain', interaction_constraints=None,
              learning_rate=None, max_delta_step=None, max_depth=None,
              min_child_weight=None, missing=nan, monotone_constraints=None,
              n_estimators=100, n_jobs=None, num_parallel_tree=None,
              random_state=None, reg_alpha=None, reg_lambda=None,
              scale_pos_weight=None, subsample=None, tree_method=None,
              validate_parameters=None, verbosity=None))
[2021-05-19 13:07:51.703568] Start parameter search for model 'Extreme Gradient Bo
osting)'
[2021-05-19 13:08:19.485504] Finish parameter search for model 'Extreme Gradient B
oosting)' (time: 27 seconds)

=== train : best params ===
{'learning_rate': 0.05, 'max_depth': 5, 'n_estimators': 50, 'subsample': 0.8}
=== train : best score : roc_auc ===
0.8112565579968283



('k-Nearest Neighbours)', KNeighborsClassifier())
[2021-05-19 13:08:19.487498] Start parameter search for model 'k-Nearest Neighbour
s)'
[2021-05-19 13:08:27.445708] Finish parameter search for model 'k-Nearest Neighbou
rs)' (time: 7 seconds)

=== train : best params ===
{'n_neighbors': 60, 'weights': 'distance'}
=== train : best score : roc_auc ===
0.7879973030072982
```

```
('Decision Tree', DecisionTreeClassifier())
[2021-05-19 13:08:27.445708] Start parameter search for model 'Decision Tree'
[2021-05-19 13:08:28.041553] Finish parameter search for model 'Decision Tree' (ti
me: 0 seconds)

=== train : best params ===
{'criterion': 'gini', 'max_depth': 4}
=== train : best score : roc_auc ===
0.7920169080397645


('Extra Tree', ExtraTreesClassifier())
[2021-05-19 13:08:28.041553] Start parameter search for model 'Extra Tree'
[2021-05-19 13:08:31.068029] Finish parameter search for model 'Extra Tree' (time:
3 seconds)

=== train : best params ===
{'max_features': 'log2', 'n_estimators': 100}
=== train : best score : roc_auc ===
0.8076640485007524


('Gradient Boosting', GradientBoostingClassifier())
[2021-05-19 13:08:31.069027] Start parameter search for model 'Gradient Boosting'
[2021-05-19 13:08:46.058231] Finish parameter search for model 'Gradient Boosting'
(time: 14 seconds)

=== train : best params ===
{'learning_rate': 0.075, 'max_depth': 5, 'max_features': 4, 'n_estimators': 50}
=== train : best score : roc_auc ===
0.8115822503665012


('Adaptive Boosting', AdaBoostClassifier())
[2021-05-19 13:08:46.058231] Start parameter search for model 'Adaptive Boosting'
[2021-05-19 13:08:49.273142] Finish parameter search for model 'Adaptive Boosting'
(time: 3 seconds)

=== train : best params ===
{'learning_rate': 0.2, 'n_estimators': 50}
=== train : best score : roc_auc ===
0.8018674325303042


('Suppport Vector Classfier', SVC(probability=True))
[2021-05-19 13:08:49.273142] Start parameter search for model 'Suppport Vector Cla
ssfier'
[2021-05-19 13:12:56.163868] Finish parameter search for model 'Suppport Vector Cl
assfier' (time: 246 seconds)

=== train : best params ===
{'C': 1, 'gamma': 0.1, 'kernel': 'rbf'}
=== train : best score : roc_auc ===
0.7815847004882777


('Neural Network', MLPClassifier())
[2021-05-19 13:12:56.164865] Start parameter search for model 'Neural Network'
[2021-05-19 13:15:17.916434] Finish parameter search for model 'Neural Network' (t
```

```
ime: 141 seconds)

=== train : best params ===
{'activation': 'logistic', 'alpha': 0.05, 'hidden_layer_sizes': (50,), 'learning_r
ate': 'adaptive'}
=== train : best score : roc_auc ===
0.8090053336913698



Confusion Matrix:
[[821  60]
 [426 573]]

Classification Report:
              precision    recall  f1-score   support

         0.0       0.66      0.93      0.77       881
         1.0       0.91      0.57      0.70       999

    accuracy                           0.74      1880
   macro avg       0.78      0.75      0.74      1880
weighted avg       0.79      0.74      0.73      1880
```

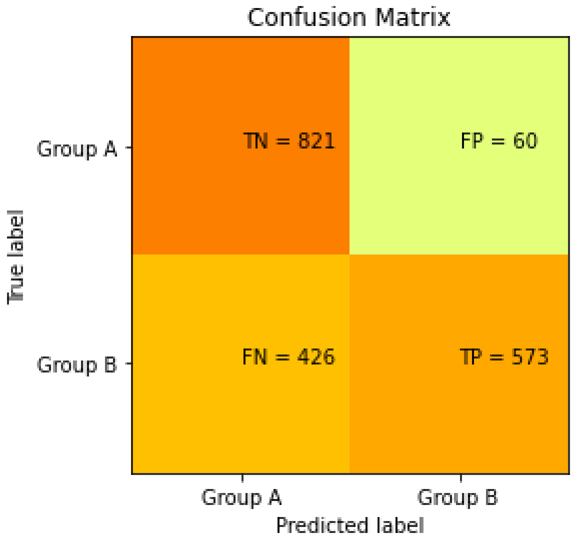


```
AUC Score : 0.7527345733929162

Accuracy Of the Model(LogisticRegression): 0.7414893617021276
```

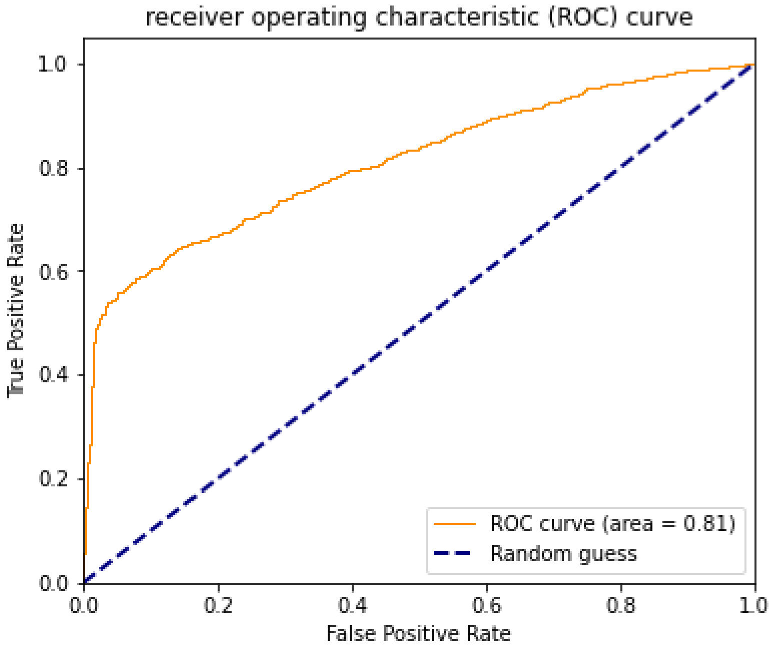


```
ROC_AUC Score of the model(LogisticRegression): 0.8118822568311785


Confusion Matrix:
[[798  83]
 [400 599]]

Classification Report:
              precision    recall  f1-score   support

         0.0       0.67      0.91      0.77       881
         1.0       0.88      0.60      0.71       999

    accuracy                           0.74      1880
   macro avg       0.77      0.75      0.74      1880
weighted avg       0.78      0.74      0.74      1880
```

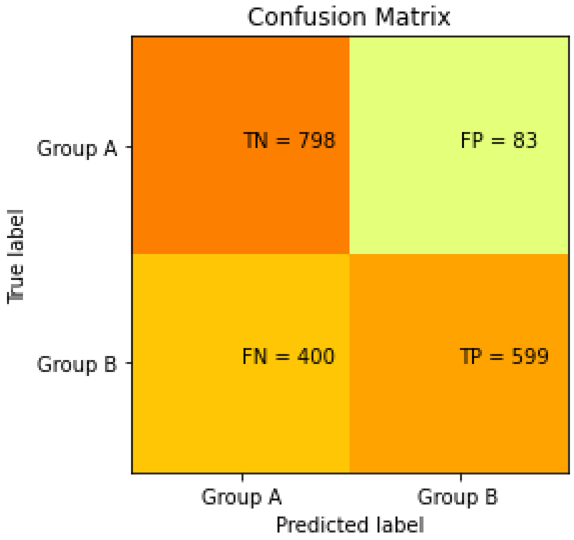


```
AUC Score : 0.7526942379382788

Accuracy Of the Model(RandomForestClassifier): 0.7430851063829788
```

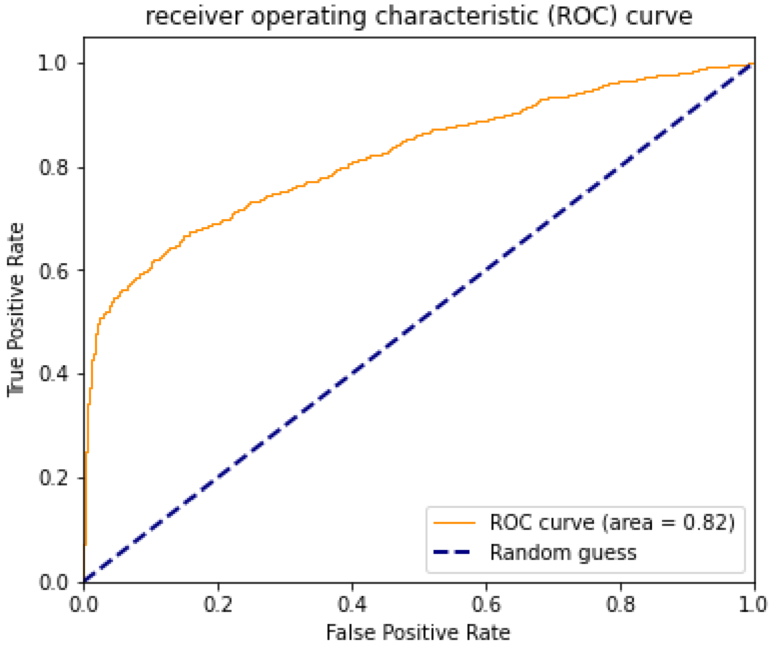

```
ROC_AUC Score of the model(RandomForestClassifier): 0.82093103318983


Confusion Matrix:
[[822  59]
 [418 581]]

Classification Report:
              precision    recall  f1-score   support

         0.0       0.66      0.93      0.78       881
         1.0       0.91      0.58      0.71       999

    accuracy                           0.75      1880
   macro avg       0.79      0.76      0.74      1880
weighted avg       0.79      0.75      0.74      1880
```

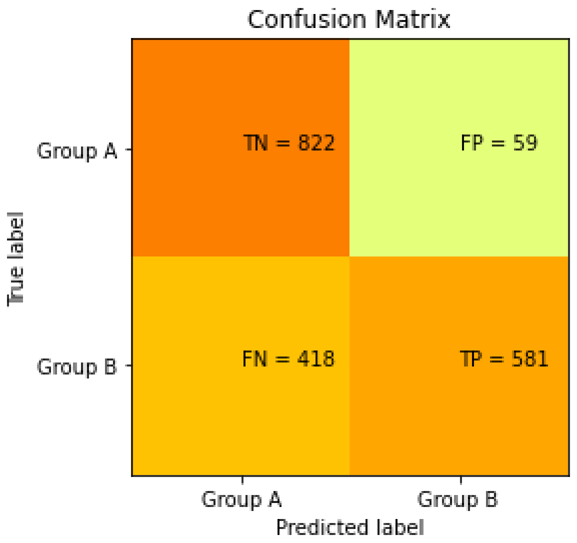


```
AUC Score : 0.7573061142868179

Accuracy Of the Model(XGBClassifier): 0.7462765957446809
```

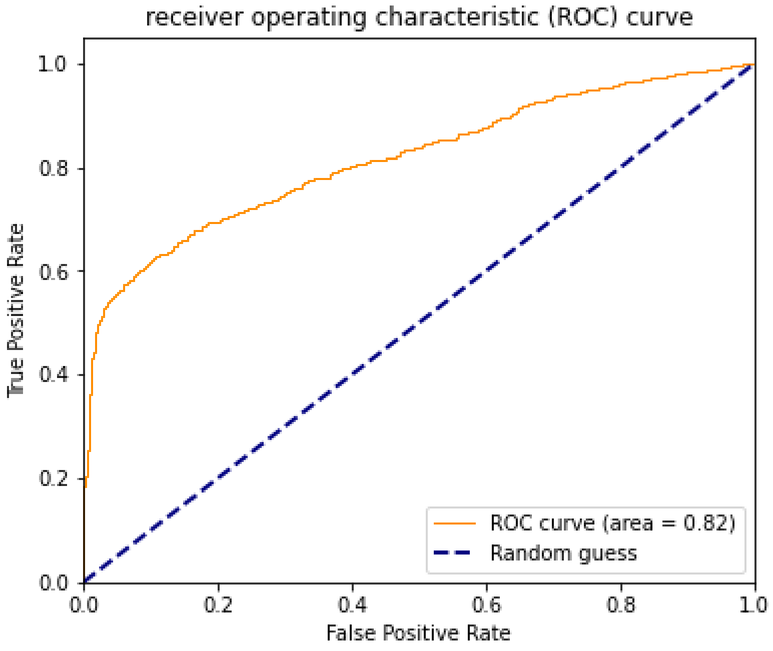

```
ROC_AUC Score of the model(XGBClassifier): 0.8177280572286247


Confusion Matrix:
[[801  80]
 [455 544]]

Classification Report:
              precision    recall  f1-score   support

         0.0       0.64      0.91      0.75       881
         1.0       0.87      0.54      0.67       999

    accuracy                           0.72      1880
   macro avg       0.75      0.73      0.71      1880
weighted avg       0.76      0.72      0.71      1880
```

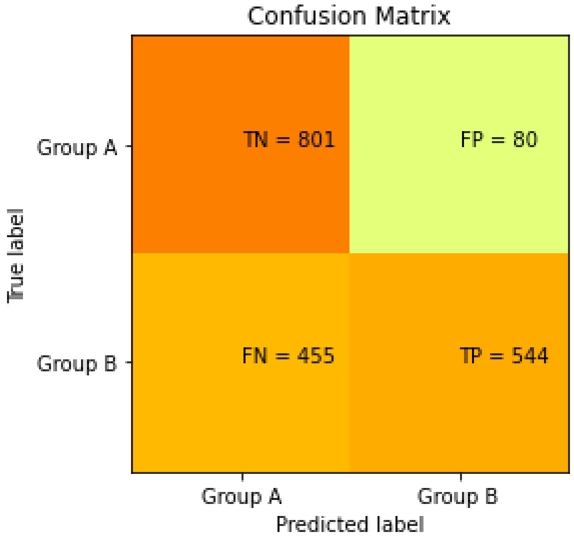


```
AUC Score : 0.7268693210804448

Accuracy Of the Model(KNeighborsClassifier): 0.7154255319148937
```

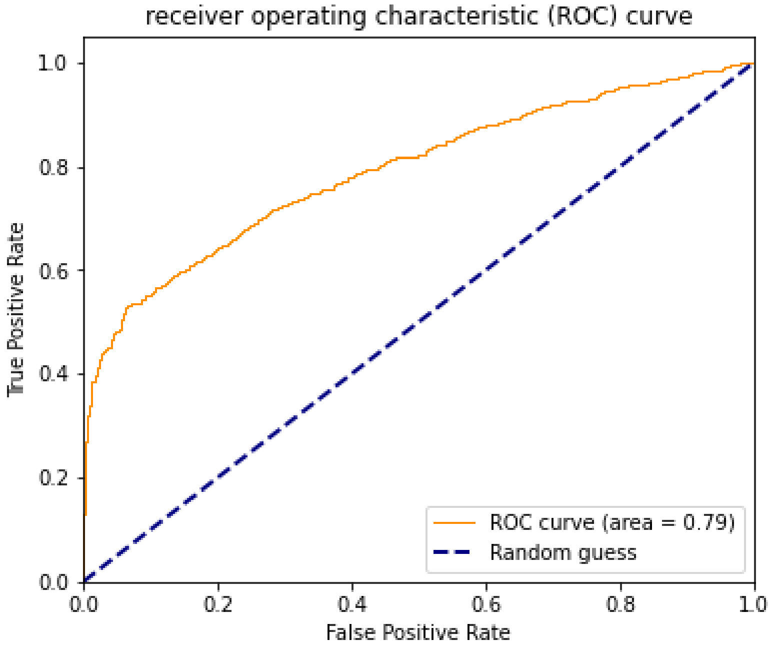

```
ROC_AUC Score of the model(KNeighborsClassifier): 0.793639269235183


Confusion Matrix:
[[844  37]
 [476 523]]

Classification Report:
              precision    recall  f1-score   support

         0.0       0.64      0.96      0.77       881
         1.0       0.93      0.52      0.67       999

    accuracy                           0.73      1880
   macro avg       0.79      0.74      0.72      1880
weighted avg       0.80      0.73      0.72      1880
```

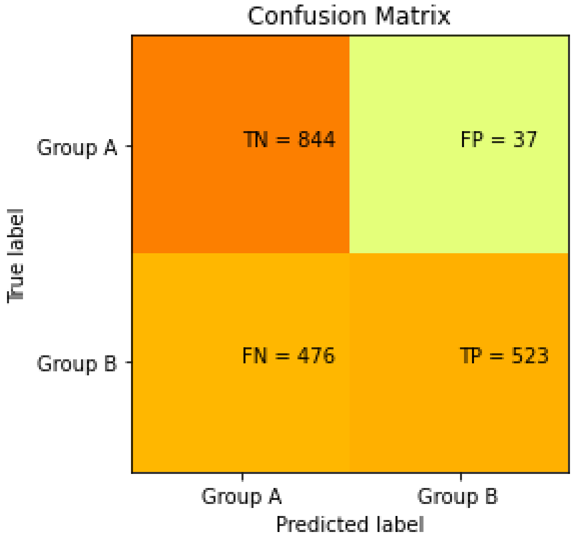


```
AUC Score : 0.7407628968355415

Accuracy Of the Model(DecisionTreeClassifier): 0.7271276595744681
```

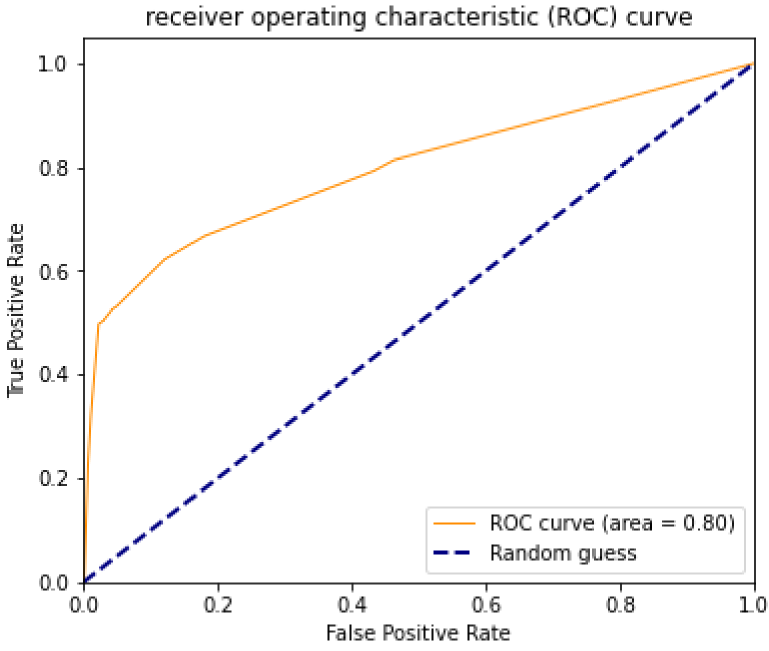

```
ROC_AUC Score of the model(DecisionTreeClassifier): 0.795487882888564


Confusion Matrix:
[[796  85]
 [411 588]]

Classification Report:
              precision    recall  f1-score   support

         0.0       0.66      0.90      0.76       881
         1.0       0.87      0.59      0.70       999

    accuracy                           0.74      1880
   macro avg       0.77      0.75      0.73      1880
weighted avg       0.77      0.74      0.73      1880
```

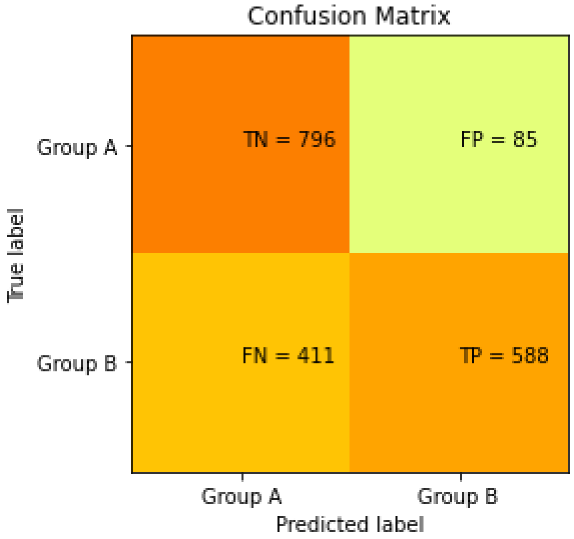


```
AUC Score : 0.7460536586529776

Accuracy Of the Model(ExtraTreesClassifier): 0.7361702127659574
```

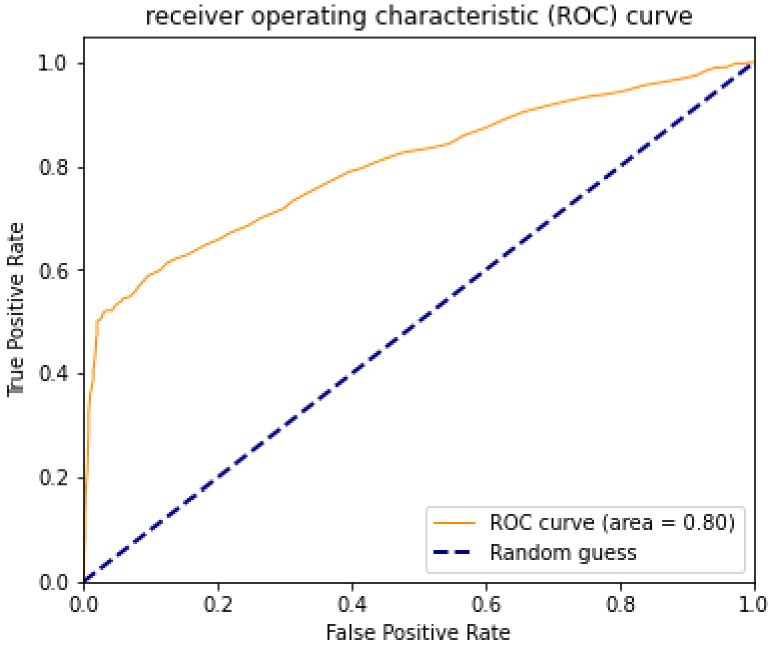

```
ROC_AUC Score of the model(ExtraTreesClassifier): 0.8013353876009949


Confusion Matrix:
[[784  97]
 [389 610]]

Classification Report:
              precision    recall  f1-score   support

         0.0       0.67      0.89      0.76       881
         1.0       0.86      0.61      0.72       999

    accuracy                           0.74      1880
   macro avg       0.77      0.75      0.74      1880
weighted avg       0.77      0.74      0.74      1880
```

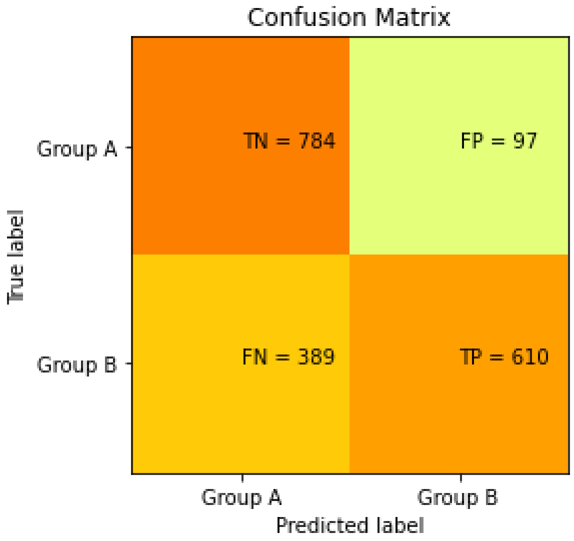


```
AUC Score : 0.7502542269852145

Accuracy Of the Model(GradientBoostingClassifier): 0.7414893617021276
```

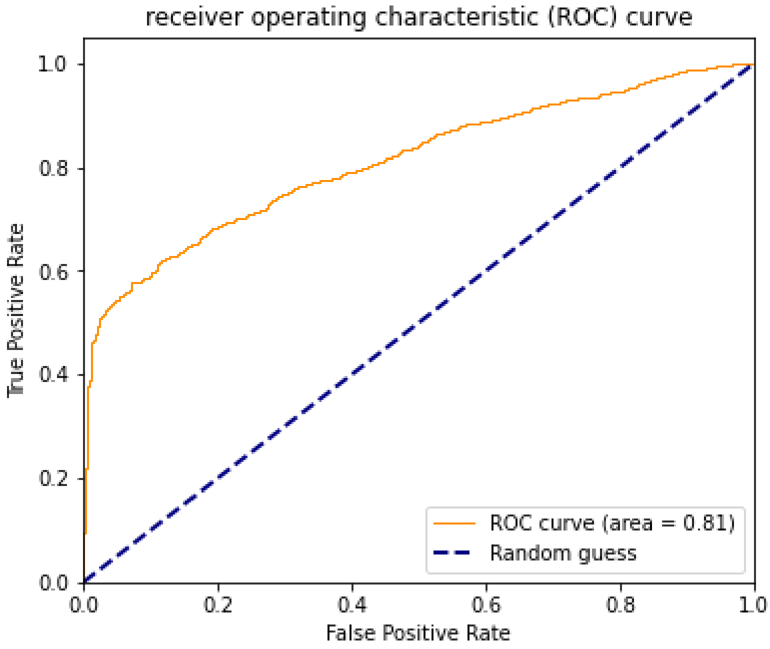

```
ROC_AUC Score of the model(GradientBoostingClassifier): 0.8129434769616382


Confusion Matrix:
[[838  43]
 [459 540]]

Classification Report:
              precision    recall  f1-score   support

         0.0       0.65      0.95      0.77       881
         1.0       0.93      0.54      0.68       999

    accuracy                           0.73      1880
   macro avg       0.79      0.75      0.73      1880
weighted avg       0.79      0.73      0.72      1880
```

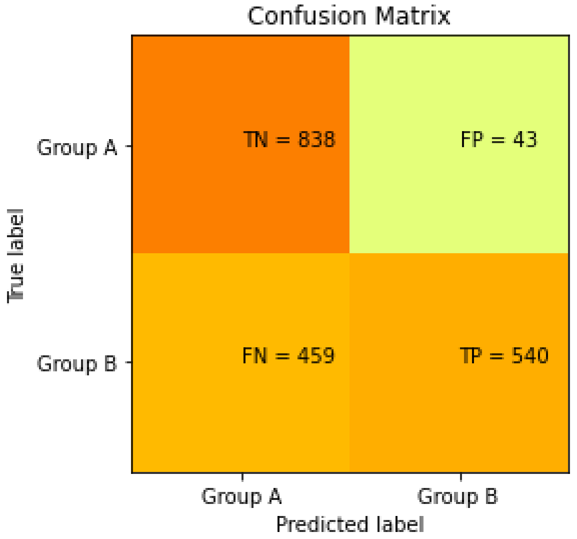


```
AUC Score : 0.7458661840046631

Accuracy Of the Model(AdaBoostClassifier): 0.7329787234042553
```

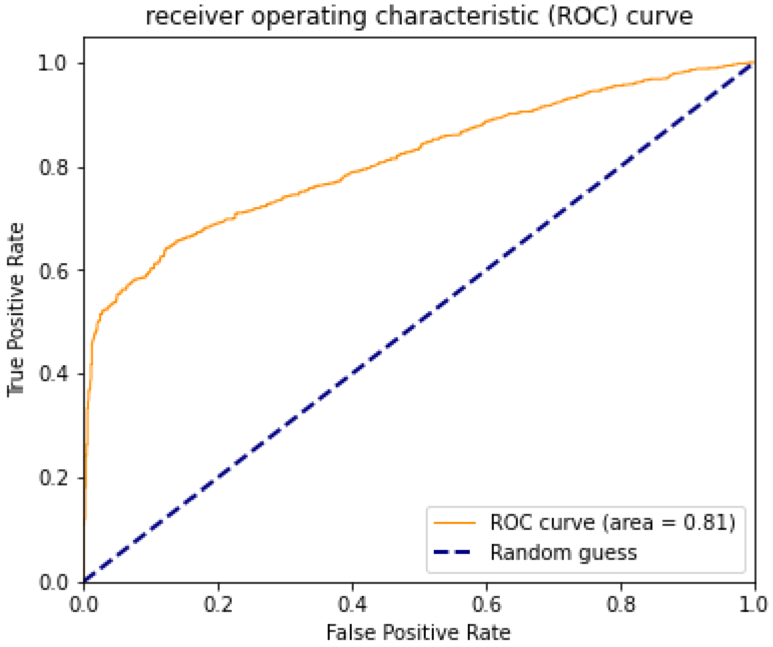

```
ROC_AUC Score of the model(AdaBoostClassifier): 0.8122816346425882


Confusion Matrix:
[[694 187]
 [346 653]]

Classification Report:
              precision    recall  f1-score   support

         0.0       0.67      0.79      0.72       881
         1.0       0.78      0.65      0.71       999

    accuracy                           0.72      1880
   macro avg       0.72      0.72      0.72      1880
weighted avg       0.73      0.72      0.72      1880
```

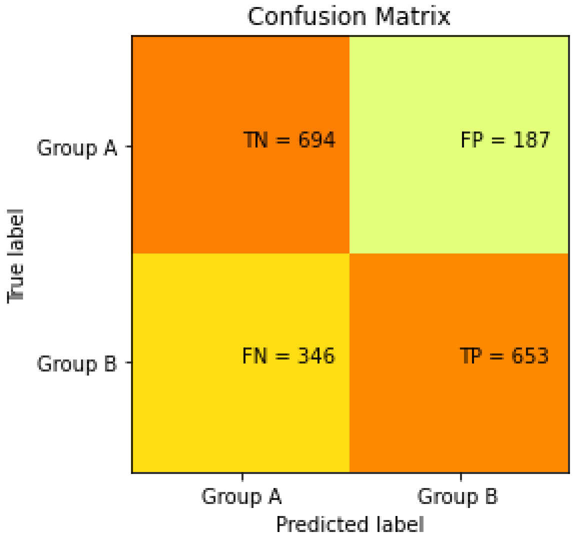


```
AUC Score : 0.7206974284159302

Accuracy Of the Model(SVC): 0.7164893617021276
```

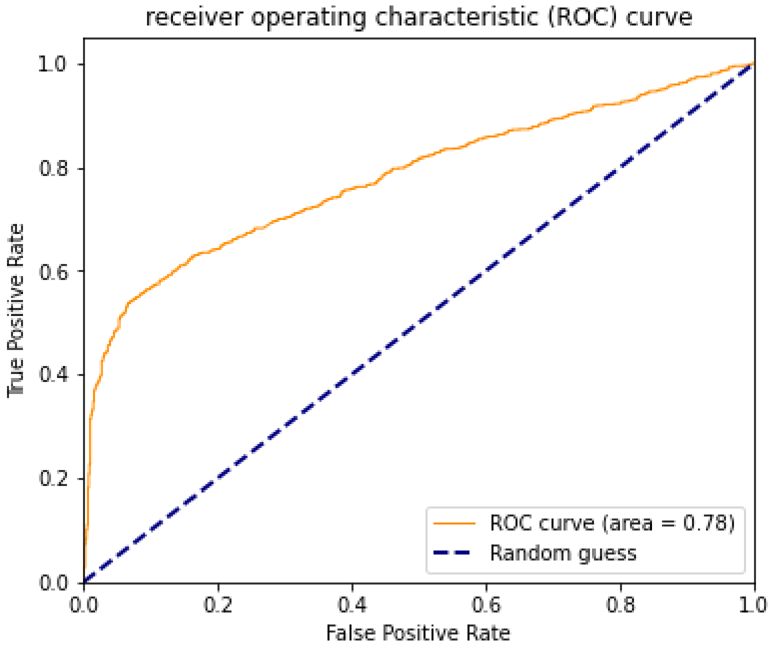

```
ROC_AUC Score of the model(SVC): 0.7792031532099637


Confusion Matrix:
[[787  94]
 [394 605]]

Classification Report:
              precision    recall  f1-score   support

         0.0       0.67      0.89      0.76       881
         1.0       0.87      0.61      0.71       999

    accuracy                           0.74      1880
   macro avg       0.77      0.75      0.74      1880
weighted avg       0.77      0.74      0.74      1880
```

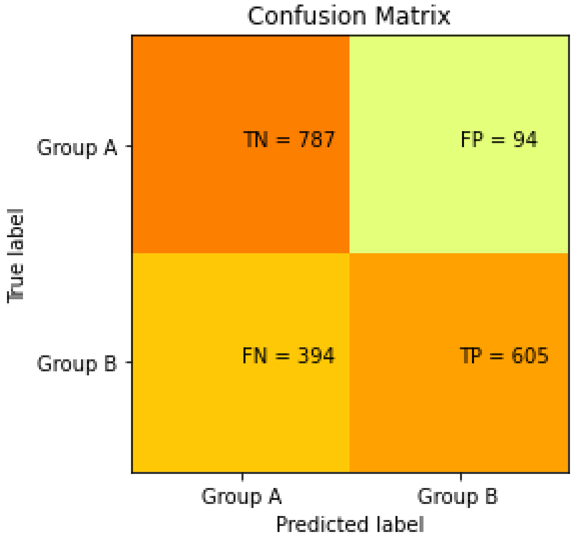


```
AUC Score : 0.7494543351524056

Accuracy Of the Model(MLPClassifier): 0.7404255319148936
```

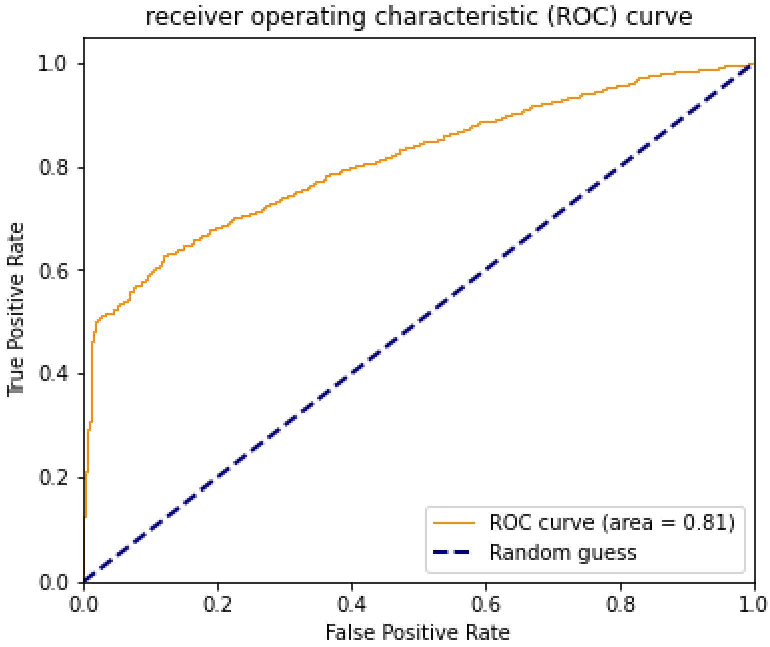

```
ROC_AUC Score of the model(MLPClassifier): 0.812642381314345

[('XGBClassifier', 0.7462765957446809), ('RandomForestClassifier', 0.7430851063829
788), ('LogisticRegression', 0.7414893617021276), ('GradientBoostingClassifier',
0.7414893617021276), ('MLPClassifier', 0.7404255319148936), ('ExtraTreesClassifie
r', 0.7361702127659574), ('AdaBoostClassifier', 0.7329787234042553), ('DecisionTre
eClassifier', 0.7271276595744681), ('SVC', 0.7164893617021276), ('KNeighborsClassi
fier', 0.7154255319148937)]


sorted_total_auc:
 [('RandomForestClassifier', 0.82093103318983), ('XGBClassifier', 0.81772805722862
47), ('GradientBoostingClassifier', 0.8129434769616382), ('MLPClassifier', 0.81264
2381314345), ('AdaBoostClassifier', 0.8122816346425882), ('LogisticRegression', 0.
8118822568311785), ('ExtraTreesClassifier', 0.8013353876009949), ('DecisionTreeCla
ssifier', 0.795487882888564), ('KNeighborsClassifier', 0.793639269235183), ('SVC',
0.7792031532099637)]
```

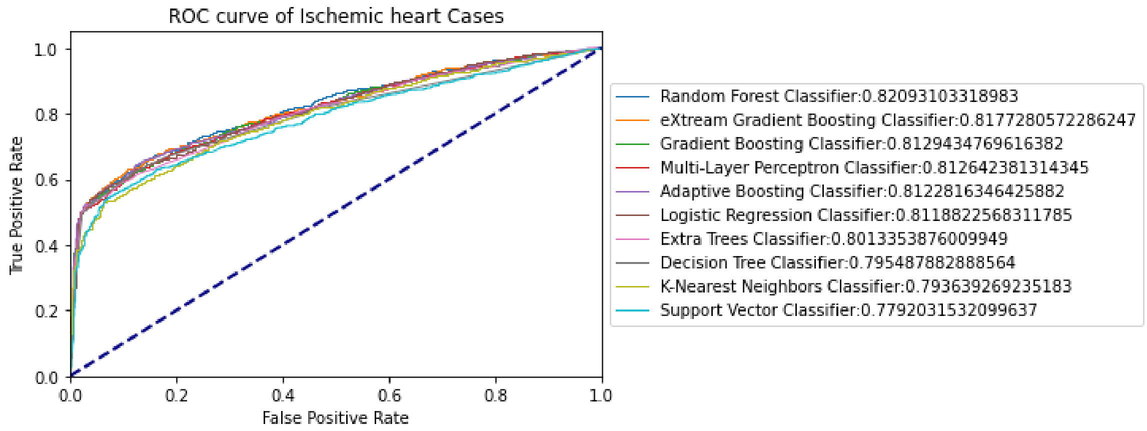

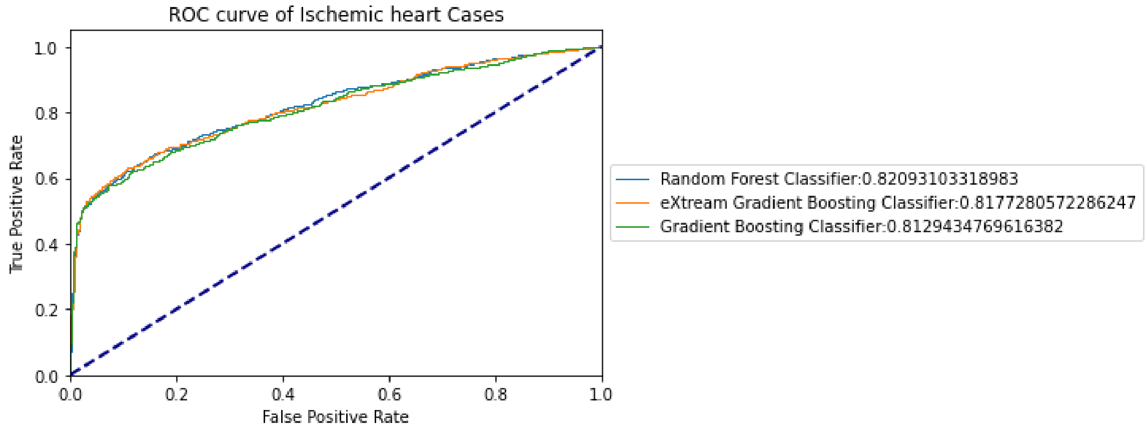

```
random state :   12516
9398
result_train_filename: data/train_test_data_ischemic heart_2013_20210519_131525.cs
v
total count : 9398, df_train count : 7518, df_test count : 1880
(7518, 38)
(1880, 38)
('Logistic Regression', LogisticRegression())
[2021-05-19 13:15:25.612171] Start parameter search for model 'Logistic Regressio
n'
[2021-05-19 13:15:25.931069] Finish parameter search for model 'Logistic Regressio
n' (time: 0 seconds)

=== train : best params ===
{'C': 10, 'penalty': 'l2', 'solver': 'lbfgs'}
=== train : best score : roc_auc ===
0.8046019349782306


('Random Forest', RandomForestClassifier())
[2021-05-19 13:15:25.932066] Start parameter search for model 'Random Forest'
[2021-05-19 13:15:30.127016] Finish parameter search for model 'Random Forest' (ti
me: 4 seconds)

=== train : best params ===
{'criterion': 'gini', 'max_depth': 8, 'max_features': 'auto', 'n_estimators': 100}
=== train : best score : roc_auc ===
0.8125642112380329


('Extreme Gradient Boosting)', XGBClassifier(base_score=None, booster=None, colsam
ple_bylevel=None,
              colsample_bynode=None, colsample_bytree=None, gamma=None,
              gpu_id=None, importance_type='gain', interaction_constraints=None,
              learning_rate=None, max_delta_step=None, max_depth=None,
              min_child_weight=None, missing=nan, monotone_constraints=None,
              n_estimators=100, n_jobs=None, num_parallel_tree=None,
              random_state=None, reg_alpha=None, reg_lambda=None,
              scale_pos_weight=None, subsample=None, tree_method=None,
              validate_parameters=None, verbosity=None))
[2021-05-19 13:15:30.127016] Start parameter search for model 'Extreme Gradient Bo
osting)'
[2021-05-19 13:15:56.475650] Finish parameter search for model 'Extreme Gradient B
oosting)' (time: 26 seconds)

=== train : best params ===
{'learning_rate': 0.05, 'max_depth': 5, 'n_estimators': 100, 'subsample': 0.8}
=== train : best score : roc_auc ===
0.812412789592903


('k-Nearest Neighbours)', KNeighborsClassifier())
[2021-05-19 13:15:56.477645] Start parameter search for model 'k-Nearest Neighbour
s)'
[2021-05-19 13:16:04.525401] Finish parameter search for model 'k-Nearest Neighbou
rs)' (time: 8 seconds)

=== train : best params ===
{'n_neighbors': 60, 'weights': 'distance'}
=== train : best score : roc_auc ===
0.7850879706612408
```

```
('Decision Tree', DecisionTreeClassifier())
[2021-05-19 13:16:04.526398] Start parameter search for model 'Decision Tree'
[2021-05-19 13:16:05.171805] Finish parameter search for model 'Decision Tree' (ti
me: 0 seconds)

=== train : best params ===
{'criterion': 'gini', 'max_depth': 5}
=== train : best score : roc_auc ===
0.7977610383513557


('Extra Tree', ExtraTreesClassifier())
[2021-05-19 13:16:05.172802] Start parameter search for model 'Extra Tree'
[2021-05-19 13:16:07.901625] Finish parameter search for model 'Extra Tree' (time:
2 seconds)

=== train : best params ===
{'max_features': 'log2', 'n_estimators': 50}
=== train : best score : roc_auc ===
0.8020127978114313


('Gradient Boosting', GradientBoostingClassifier())
[2021-05-19 13:16:07.901625] Start parameter search for model 'Gradient Boosting'
[2021-05-19 13:16:24.646802] Finish parameter search for model 'Gradient Boosting'
(time: 16 seconds)

=== train : best params ===
{'learning_rate': 0.01, 'max_depth': 8, 'max_features': 6, 'n_estimators': 100}
=== train : best score : roc_auc ===
0.8131285977196611


('Adaptive Boosting', AdaBoostClassifier())
[2021-05-19 13:16:24.646802] Start parameter search for model 'Adaptive Boosting'
[2021-05-19 13:16:28.391112] Finish parameter search for model 'Adaptive Boosting'
(time: 3 seconds)

=== train : best params ===
{'learning_rate': 0.2, 'n_estimators': 100}
=== train : best score : roc_auc ===
0.8060528675886811


('Suppport Vector Classfier', SVC(probability=True))
[2021-05-19 13:16:28.391112] Start parameter search for model 'Suppport Vector Cla
ssfier'
[2021-05-19 13:20:20.863520] Finish parameter search for model 'Suppport Vector Cl
assfier' (time: 232 seconds)

=== train : best params ===
{'C': 1, 'gamma': 0.1, 'kernel': 'rbf'}
=== train : best score : roc_auc ===
0.7829891120369372


('Neural Network', MLPClassifier())
[2021-05-19 13:20:20.863520] Start parameter search for model 'Neural Network'
[2021-05-19 13:22:35.757135] Finish parameter search for model 'Neural Network' (t
```

```
ime: 134 seconds)

=== train : best params ===
{'activation': 'logistic', 'alpha': 0.05, 'hidden_layer_sizes': (50,), 'learning_r
ate': 'constant'}
=== train : best score : roc_auc ===
0.808642253437358



Confusion Matrix:
[[814  67]
 [429 570]]

Classification Report:
              precision    recall  f1-score   support

         0.0       0.65      0.92      0.77       881
         1.0       0.89      0.57      0.70       999

    accuracy                           0.74      1880
   macro avg       0.77      0.75      0.73      1880
weighted avg       0.78      0.74      0.73      1880
```

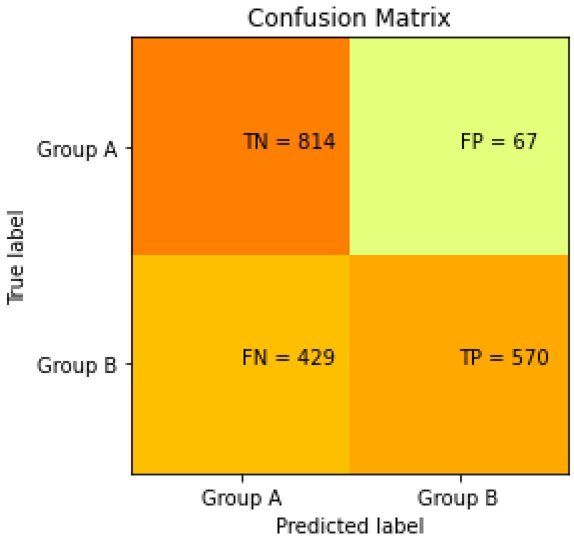


```
AUC Score : 0.7472603136621299

Accuracy Of the Model(LogisticRegression): 0.7361702127659574
```

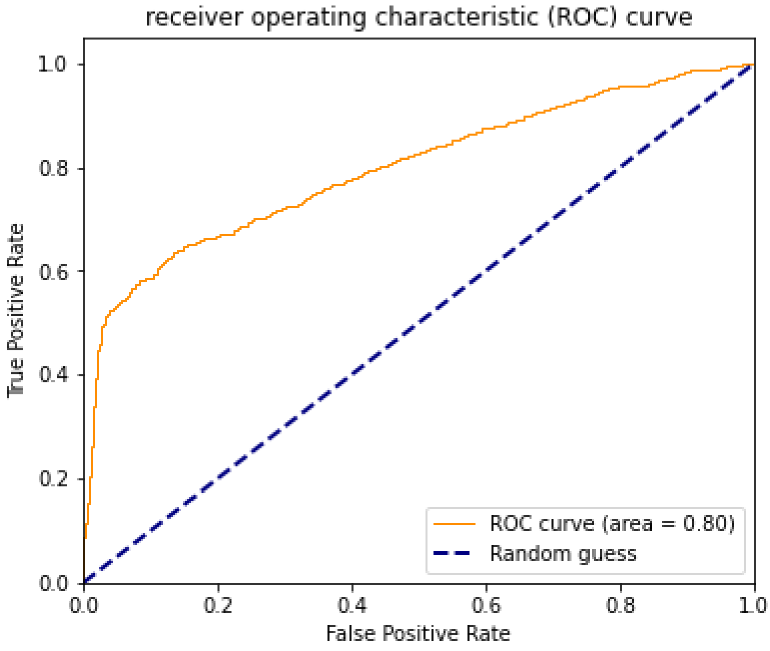


```
ROC_AUC Score of the model(LogisticRegression): 0.8003985824644168


Confusion Matrix:
[[775 106]
 [400 599]]

Classification Report:
              precision    recall  f1-score   support

         0.0       0.66      0.88      0.75       881
         1.0       0.85      0.60      0.70       999

    accuracy                           0.73      1880
   macro avg       0.75      0.74      0.73      1880
weighted avg       0.76      0.73      0.73      1880
```

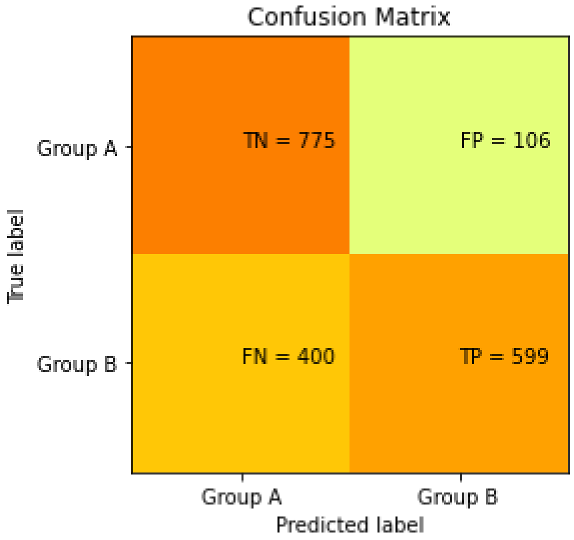


```
AUC Score : 0.7396408894706283

Accuracy Of the Model(RandomForestClassifier): 0.7308510638297873
```

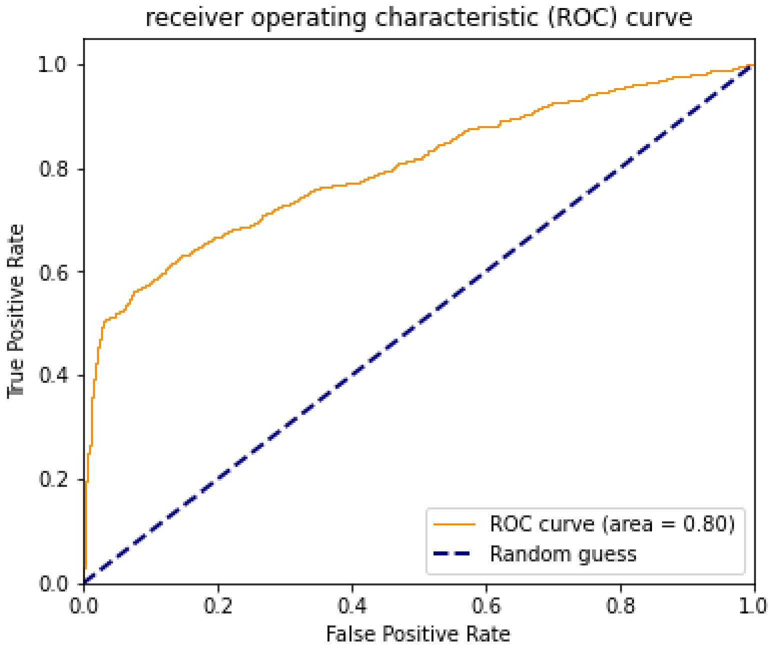

```
ROC_AUC Score of the model(RandomForestClassifier): 0.8005781036428029


Confusion Matrix:
[[772 109]
 [390 609]]

Classification Report:
              precision    recall  f1-score   support

         0.0       0.66      0.88      0.76       881
         1.0       0.85      0.61      0.71       999

    accuracy                           0.73      1880
   macro avg       0.76      0.74      0.73      1880
weighted avg       0.76      0.73      0.73      1880
```

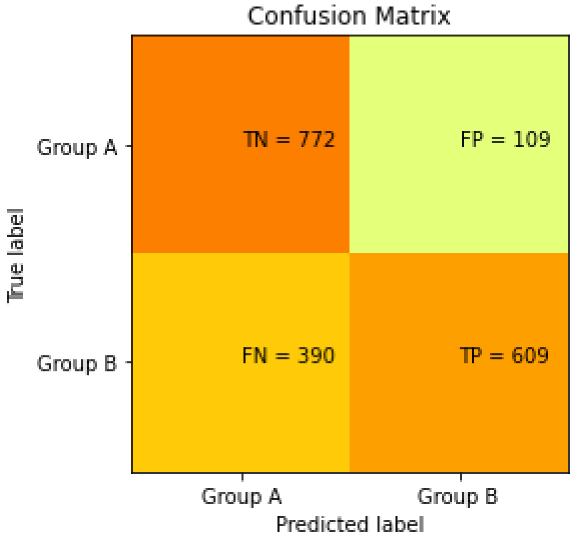


```
AUC Score : 0.742943283805594

Accuracy Of the Model(XGBClassifier): 0.7345744680851064
```

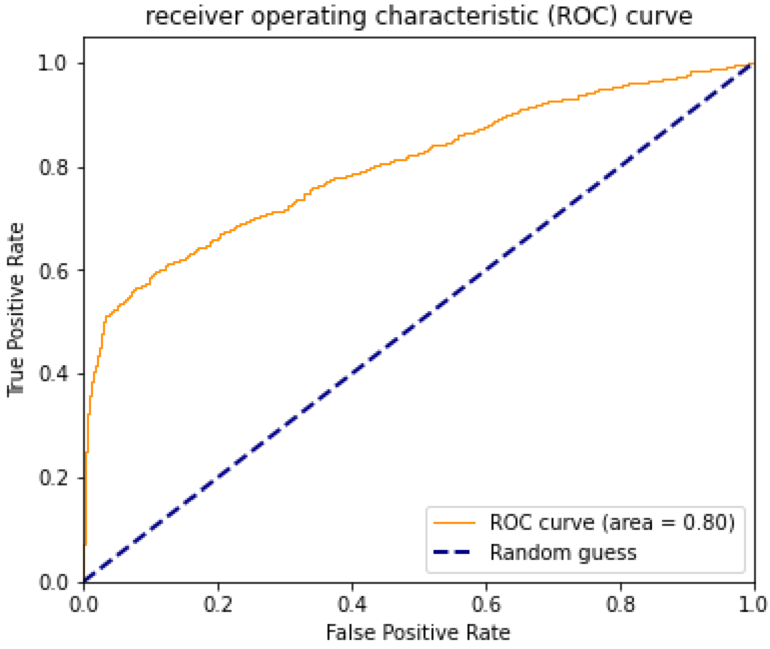

```
ROC_AUC Score of the model(XGBClassifier): 0.8030039119709949


Confusion Matrix:
[[791  90]
 [462 537]]

Classification Report:
              precision    recall  f1-score   support

         0.0       0.63      0.90      0.74       881
         1.0       0.86      0.54      0.66       999

    accuracy                           0.71      1880
   macro avg       0.74      0.72      0.70      1880
weighted avg       0.75      0.71      0.70      1880
```

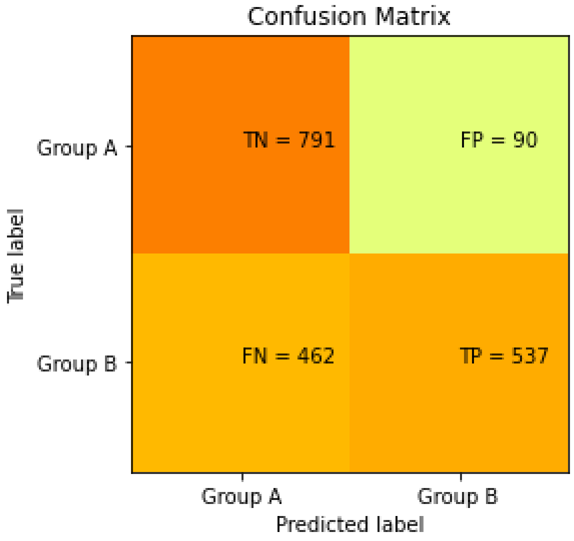


```
AUC Score : 0.7176904486779627

Accuracy Of the Model(KNeighborsClassifier): 0.7063829787234043
```

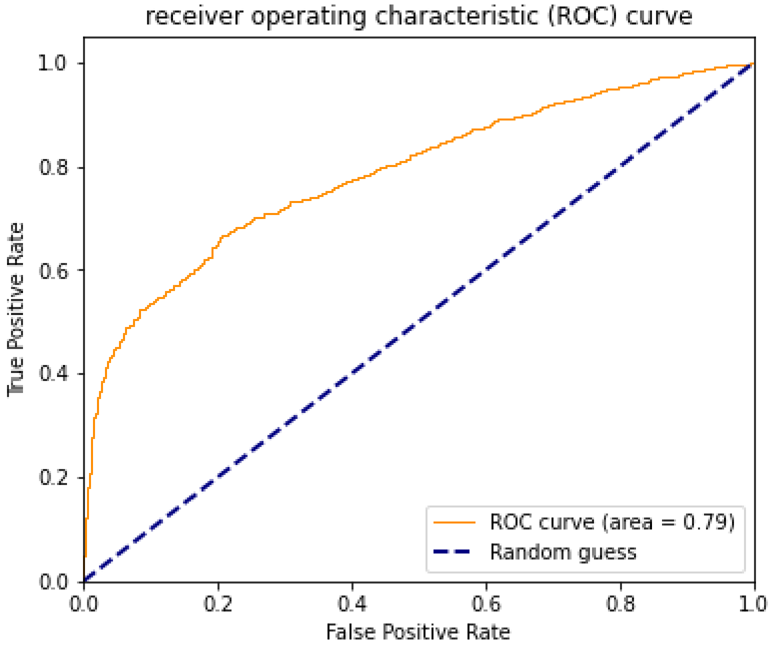

```
ROC_AUC Score of the model(KNeighborsClassifier): 0.788533141541087


Confusion Matrix:
[[789  92]
 [413 586]]

Classification Report:
              precision    recall  f1-score   support

         0.0       0.66      0.90      0.76       881
         1.0       0.86      0.59      0.70       999

    accuracy                           0.73      1880
   macro avg       0.76      0.74      0.73      1880
weighted avg       0.77      0.73      0.73      1880
```

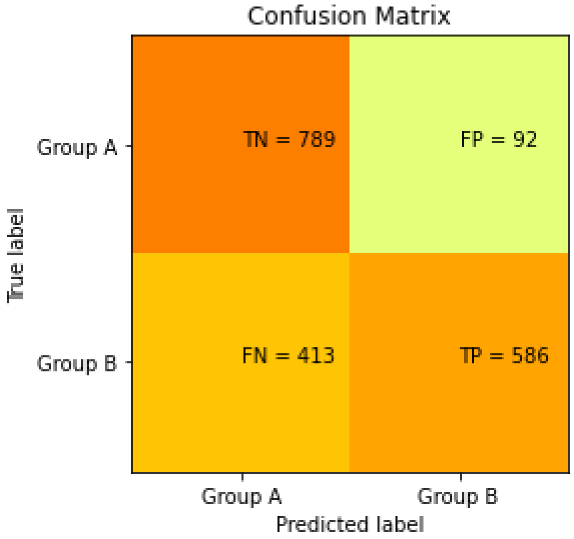


```
AUC Score : 0.7410798994226916

Accuracy Of the Model(DecisionTreeClassifier): 0.7313829787234043
```

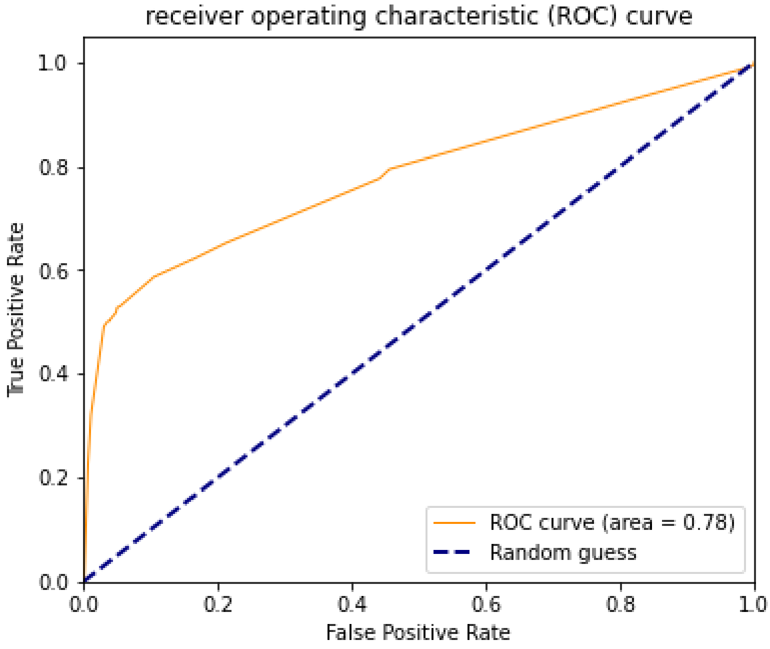

```
ROC_AUC Score of the model(DecisionTreeClassifier): 0.7785396065759289


Confusion Matrix:
[[775 106]
 [416 583]]

Classification Report:
              precision    recall  f1-score   support

         0.0       0.65      0.88      0.75       881
         1.0       0.85      0.58      0.69       999

    accuracy                           0.72      1880
   macro avg       0.75      0.73      0.72      1880
weighted avg       0.75      0.72      0.72      1880
```

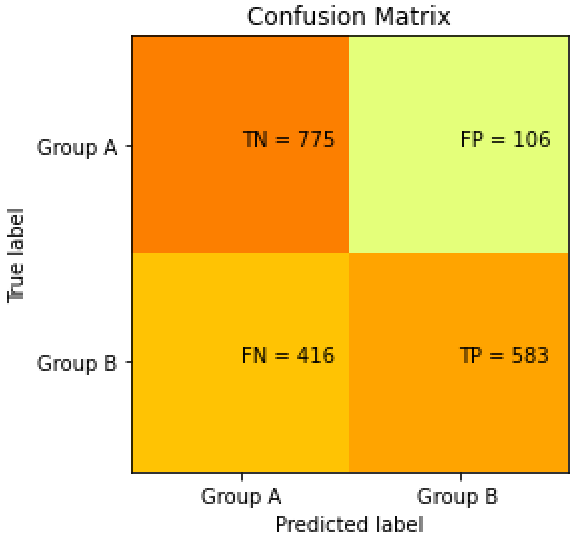


```
AUC Score : 0.7316328814626203

Accuracy Of the Model(ExtraTreesClassifier): 0.7223404255319149
```

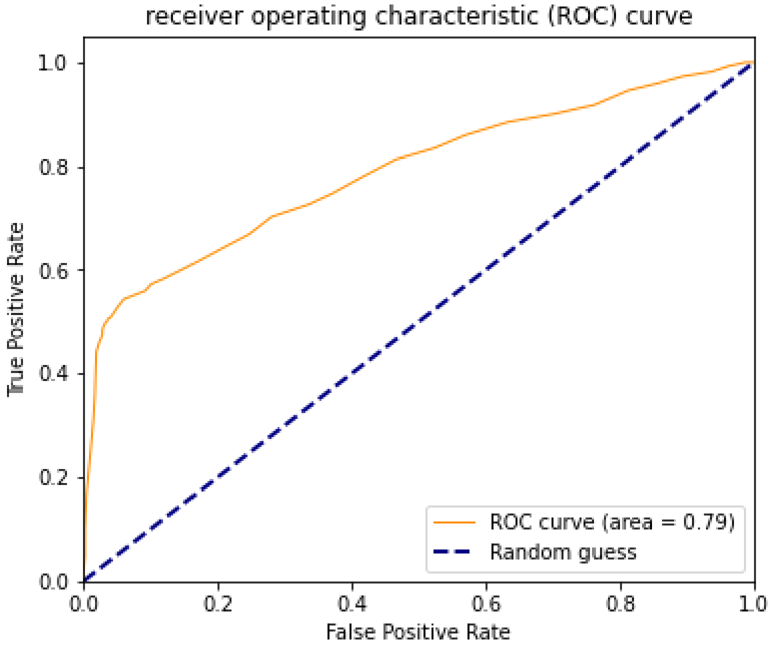

```
ROC_AUC Score of the model(ExtraTreesClassifier): 0.7885689321557654


Confusion Matrix:
[[780 101]
 [407 592]]

Classification Report:
              precision    recall  f1-score   support

         0.0       0.66      0.89      0.75       881
         1.0       0.85      0.59      0.70       999

    accuracy                           0.73      1880
   macro avg       0.76      0.74      0.73      1880
weighted avg       0.76      0.73      0.73      1880
```

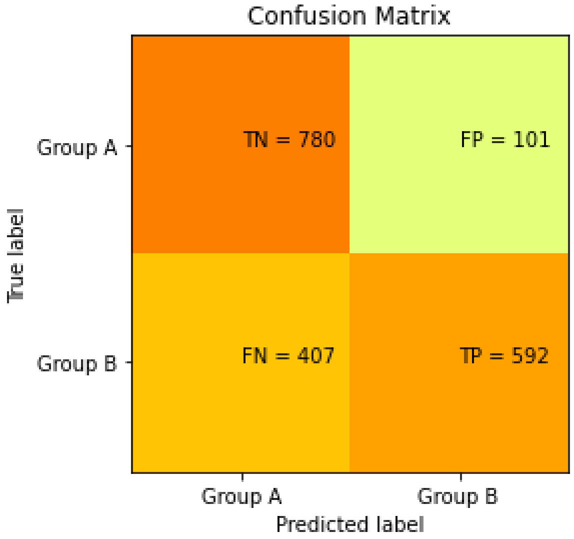


```
AUC Score : 0.7389750704166141

Accuracy Of the Model(GradientBoostingClassifier): 0.7297872340425532
```

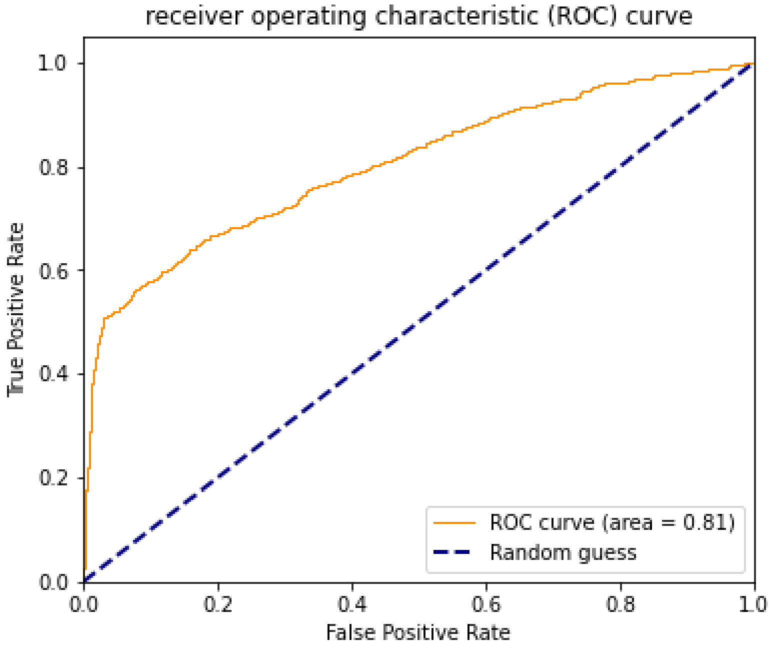

```
ROC_AUC Score of the model(GradientBoostingClassifier): 0.8050150036529151


Confusion Matrix:
[[801  80]
 [430 569]]

Classification Report:
              precision    recall  f1-score   support

         0.0       0.65      0.91      0.76       881
         1.0       0.88      0.57      0.69       999

    accuracy                           0.73      1880
   macro avg       0.76      0.74      0.72      1880
weighted avg       0.77      0.73      0.72      1880
```

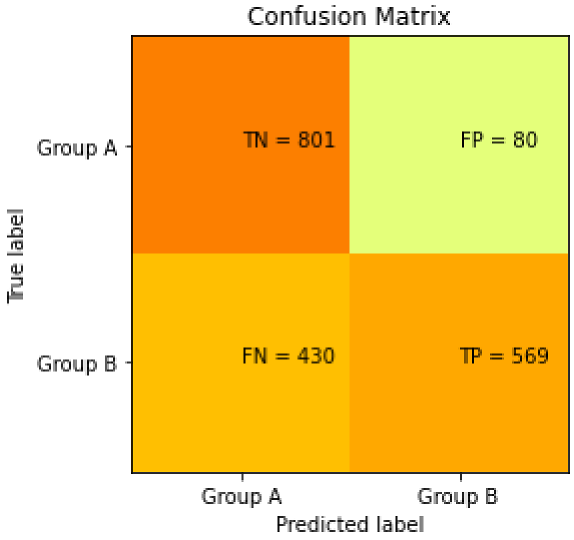


```
AUC Score : 0.7393818335929574

Accuracy Of the Model(AdaBoostClassifier): 0.7287234042553191
```

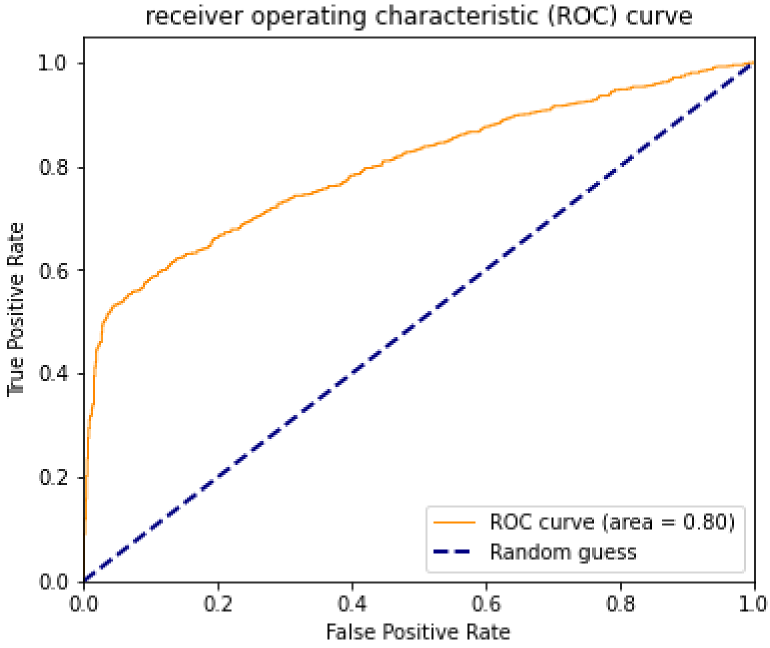

```
ROC_AUC Score of the model(AdaBoostClassifier): 0.799005020911945


Confusion Matrix:
[[689 192]
 [350 649]]

Classification Report:
              precision    recall  f1-score   support

         0.0       0.66      0.78      0.72       881
         1.0       0.77      0.65      0.71       999

    accuracy                           0.71      1880
   macro avg       0.72      0.72      0.71      1880
weighted avg       0.72      0.71      0.71      1880
```

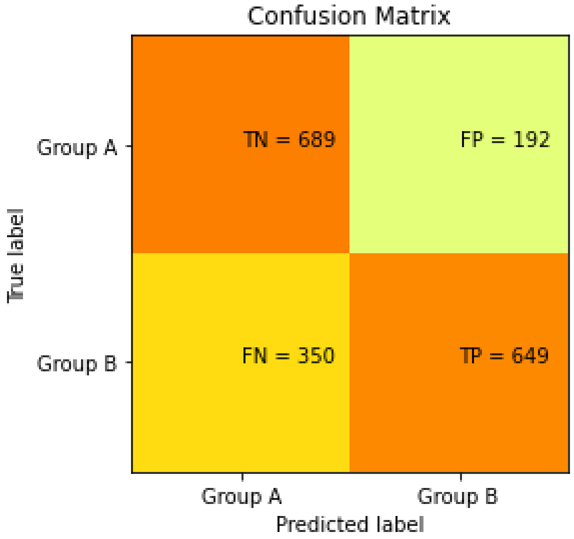


```
AUC Score : 0.7158577419644439

Accuracy Of the Model(SVC): 0.7117021276595744
```

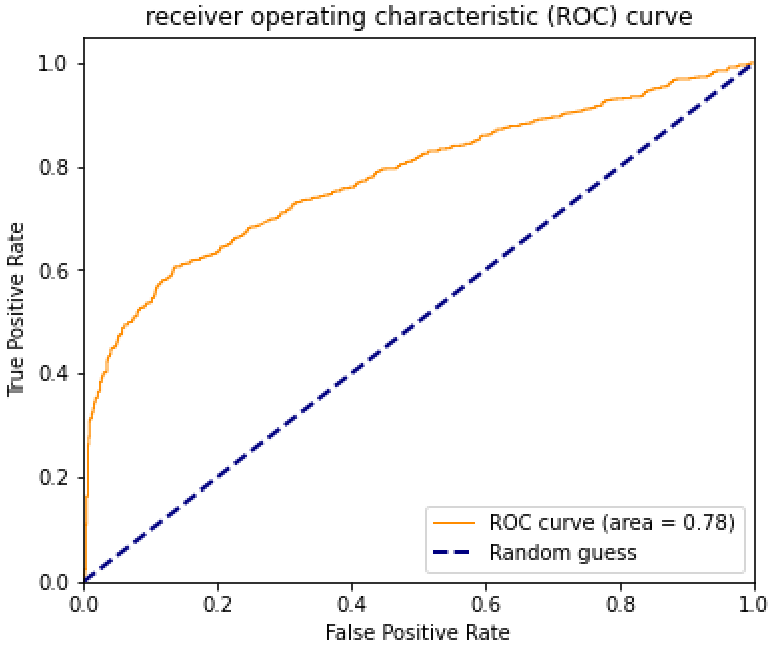

```
ROC_AUC Score of the model(SVC): 0.7797161520203517


Confusion Matrix:
[[789  92]
 [407 592]]

Classification Report:
              precision    recall  f1-score   support

         0.0       0.66      0.90      0.76       881
         1.0       0.87      0.59      0.70       999

    accuracy                           0.73      1880
   macro avg       0.76      0.74      0.73      1880
weighted avg       0.77      0.73      0.73      1880
```

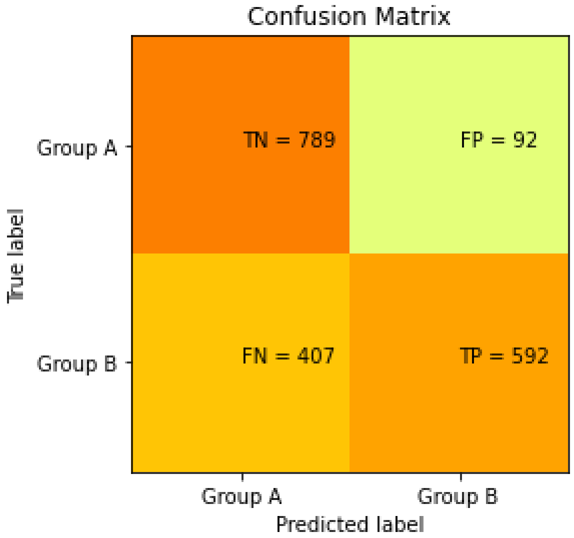


```
AUC Score : 0.7440829024256947

Accuracy Of the Model(MLPClassifier): 0.7345744680851064
```

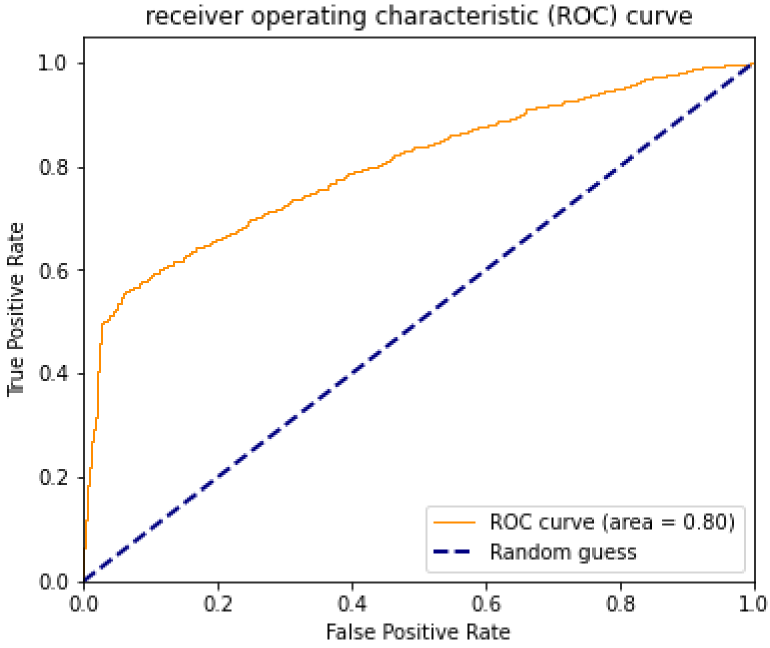

```
ROC_AUC Score of the model(MLPClassifier): 0.800730355781434

[('LogisticRegression', 0.7361702127659574), ('XGBClassifier', 0.734574468085106
4), ('MLPClassifier', 0.7345744680851064), ('DecisionTreeClassifier', 0.7313829787
234043), ('RandomForestClassifier', 0.7308510638297873), ('GradientBoostingClassif
ier', 0.7297872340425532), ('AdaBoostClassifier', 0.7287234042553191), ('ExtraTree
sClassifier', 0.7223404255319149), ('SVC', 0.7117021276595744), ('KNeighborsClassi
fier', 0.7063829787234043)]


sorted_total_auc:
 [('GradientBoostingClassifier', 0.8050150036529151), ('XGBClassifier', 0.80300391
19709949), ('MLPClassifier', 0.800730355781434), ('RandomForestClassifier', 0.8005
781036428029), ('LogisticRegression', 0.8003985824644168), ('AdaBoostClassifier',
0.799005020911945), ('ExtraTreesClassifier', 0.7885689321557654), ('KNeighborsClas
sifier', 0.788533141541087), ('SVC', 0.7797161520203517), ('DecisionTreeClassifie
r', 0.7785396065759289)]
```

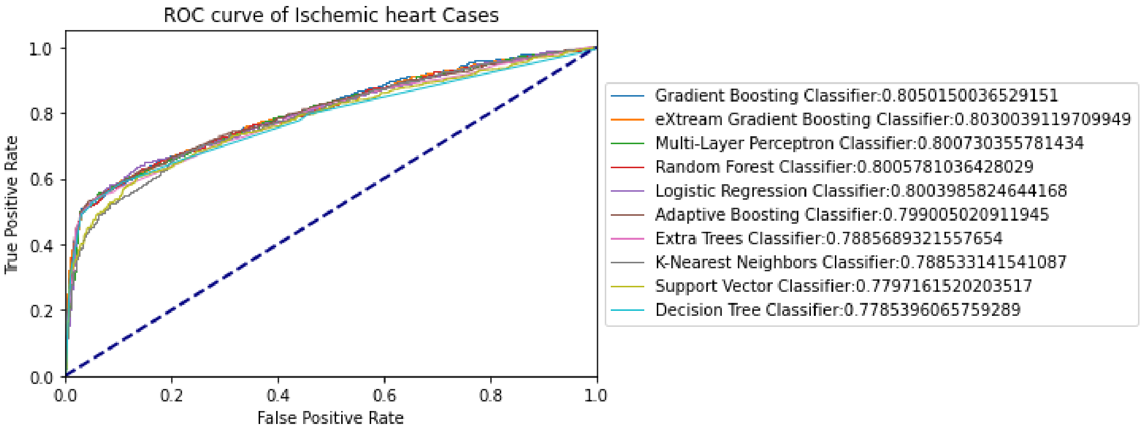

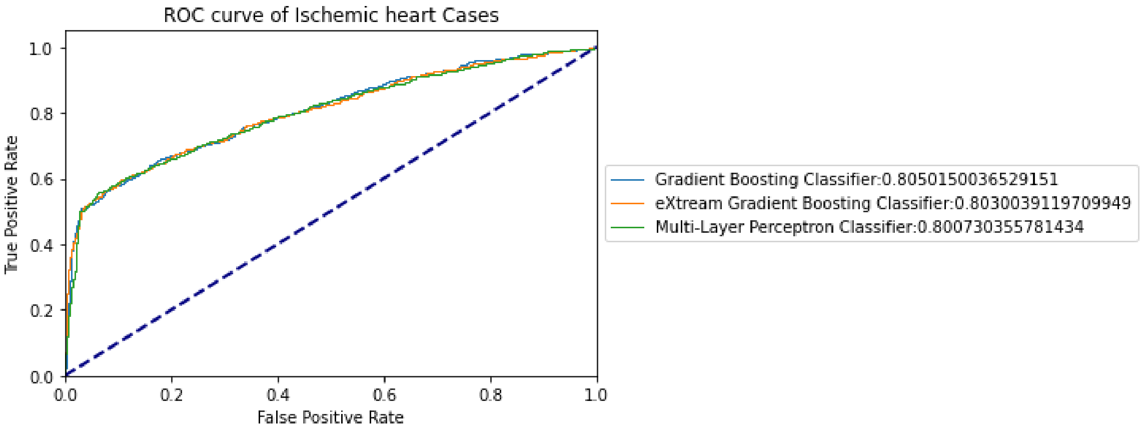

```
random state :   12535
9398
result_train_filename: data/train_test_data_ischemic heart_2013_20210519_132243.cs
v
total count : 9398, df_train count : 7518, df_test count : 1880
(7518, 38)
(1880, 38)
('Logistic Regression', LogisticRegression())
[2021-05-19 13:22:43.518628] Start parameter search for model 'Logistic Regressio
n'
[2021-05-19 13:22:43.876205] Finish parameter search for model 'Logistic Regressio
n' (time: 0 seconds)

=== train : best params ===
{'C': 0.1, 'penalty': 'l2', 'solver': 'lbfgs'}
=== train : best score : roc_auc ===
0.8074030190058131


('Random Forest', RandomForestClassifier())
[2021-05-19 13:22:43.877203] Start parameter search for model 'Random Forest'
[2021-05-19 13:22:48.503183] Finish parameter search for model 'Random Forest' (ti
me: 4 seconds)

=== train : best params ===
{'criterion': 'gini', 'max_depth': 10, 'max_features': 'auto', 'n_estimators': 10
0}
=== train : best score : roc_auc ===
0.8112816917489334


('Extreme Gradient Boosting)', XGBClassifier(base_score=None, booster=None, colsam
ple_bylevel=None,
              colsample_bynode=None, colsample_bytree=None, gamma=None,
              gpu_id=None, importance_type='gain', interaction_constraints=None,
              learning_rate=None, max_delta_step=None, max_depth=None,
              min_child_weight=None, missing=nan, monotone_constraints=None,
              n_estimators=100, n_jobs=None, num_parallel_tree=None,
              random_state=None, reg_alpha=None, reg_lambda=None,
              scale_pos_weight=None, subsample=None, tree_method=None,
              validate_parameters=None, verbosity=None))
[2021-05-19 13:22:48.504185] Start parameter search for model 'Extreme Gradient Bo
osting)'
[2021-05-19 13:23:16.269133] Finish parameter search for model 'Extreme Gradient B
oosting)' (time: 27 seconds)

=== train : best params ===
{'learning_rate': 0.05, 'max_depth': 5, 'n_estimators': 100, 'subsample': 0.8}
=== train : best score : roc_auc ===
0.8152647188115463


('k-Nearest Neighbours)', KNeighborsClassifier())
[2021-05-19 13:23:16.271128] Start parameter search for model 'k-Nearest Neighbour
s)'
[2021-05-19 13:23:24.340071] Finish parameter search for model 'k-Nearest Neighbou
rs)' (time: 8 seconds)

=== train : best params ===
{'n_neighbors': 60, 'weights': 'distance'}
=== train : best score : roc_auc ===
```

```
0.7878138123250489


('Decision Tree', DecisionTreeClassifier())
[2021-05-19 13:23:24.341034] Start parameter search for model 'Decision Tree'
[2021-05-19 13:23:24.970508] Finish parameter search for model 'Decision Tree' (ti
me: 0 seconds)

=== train : best params ===
{'criterion': 'entropy', 'max_depth': 5}
=== train : best score : roc_auc ===
0.7925381778010454


('Extra Tree', ExtraTreesClassifier())
[2021-05-19 13:23:24.970508] Start parameter search for model 'Extra Tree'
[2021-05-19 13:23:28.189306] Finish parameter search for model 'Extra Tree' (time:
3 seconds)

=== train : best params ===
{'max_features': 'auto', 'n_estimators': 100}
=== train : best score : roc_auc ===
0.8038804914116403


('Gradient Boosting', GradientBoostingClassifier())
[2021-05-19 13:23:28.190300] Start parameter search for model 'Gradient Boosting'
[2021-05-19 13:23:45.329123] Finish parameter search for model 'Gradient Boosting'
(time: 17 seconds)

=== train : best params ===
{'learning_rate': 0.01, 'max_depth': 8, 'max_features': 6, 'n_estimators': 100}
=== train : best score : roc_auc ===
0.8150516062537725


('Adaptive Boosting', AdaBoostClassifier())
[2021-05-19 13:23:45.329123] Start parameter search for model 'Adaptive Boosting'
[2021-05-19 13:23:49.116461] Finish parameter search for model 'Adaptive Boosting'
(time: 3 seconds)

=== train : best params ===
{'learning_rate': 0.2, 'n_estimators': 100}
=== train : best score : roc_auc ===
0.807298283429382


('Suppport Vector Classfier', SVC(probability=True))
[2021-05-19 13:23:49.116461] Start parameter search for model 'Suppport Vector Cla
ssfier'
[2021-05-19 13:29:04.982326] Finish parameter search for model 'Suppport Vector Cl
assfier' (time: 315 seconds)

=== train : best params ===
{'C': 1, 'gamma': 0.1, 'kernel': 'rbf'}
=== train : best score : roc_auc ===
0.7831419743168977


('Neural Network', MLPClassifier())
[2021-05-19 13:29:04.983292] Start parameter search for model 'Neural Network'
```

```
[2021-05-19 13:31:16.287397] Finish parameter search for model 'Neural Network' (t
ime: 131 seconds)

=== train : best params ===
{'activation': 'logistic', 'alpha': 0.05, 'hidden_layer_sizes': (50,), 'learning_r
ate': 'adaptive'}
=== train : best score : roc_auc ===
0.811732431776429



Confusion Matrix:
[[822  59]
 [425 574]]

Classification Report:
              precision    recall  f1-score   support

         0.0       0.66      0.93      0.77       881
         1.0       0.91      0.57      0.70       999

    accuracy                           0.74      1880
   macro avg       0.78      0.75      0.74      1880
weighted avg       0.79      0.74      0.74      1880
```

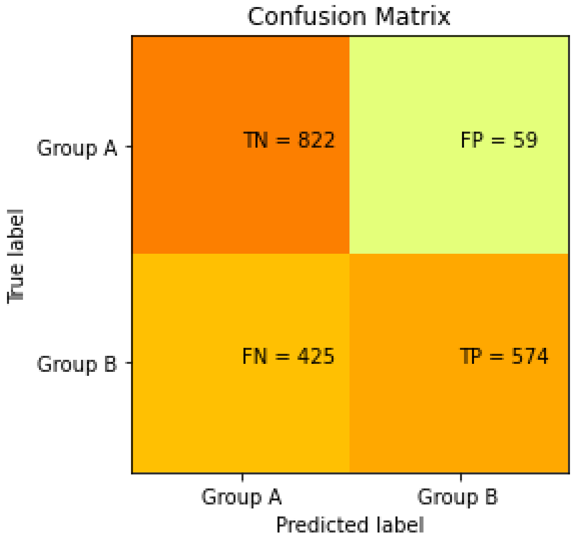


```
AUC Score : 0.7538026107833147

Accuracy Of the Model(LogisticRegression): 0.7425531914893617
```

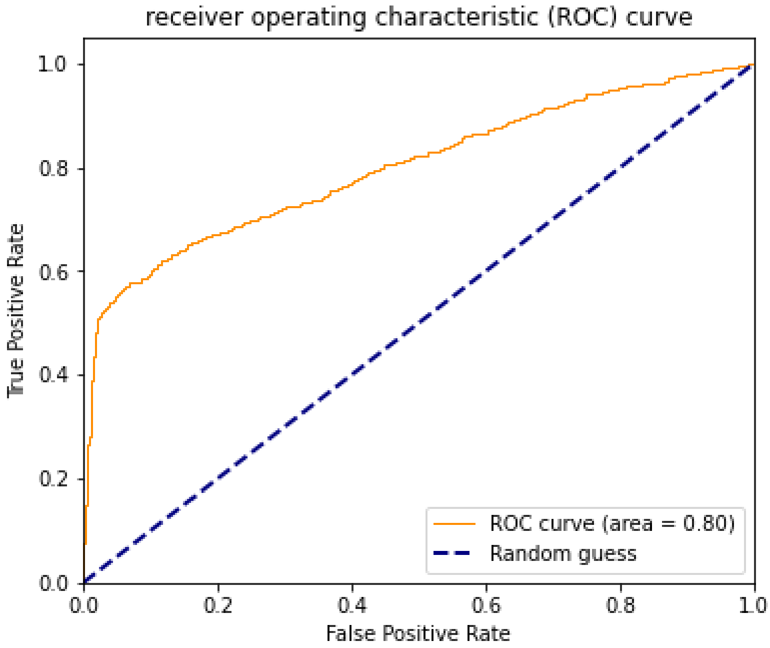


```
ROC_AUC Score of the model(LogisticRegression): 0.8015097958344269


Confusion Matrix:
[[804  77]
 [398 601]]

Classification Report:
              precision    recall  f1-score   support

         0.0       0.67      0.91      0.77       881
         1.0       0.89      0.60      0.72       999

    accuracy                           0.75      1880
   macro avg       0.78      0.76      0.74      1880
weighted avg       0.78      0.75      0.74      1880
```

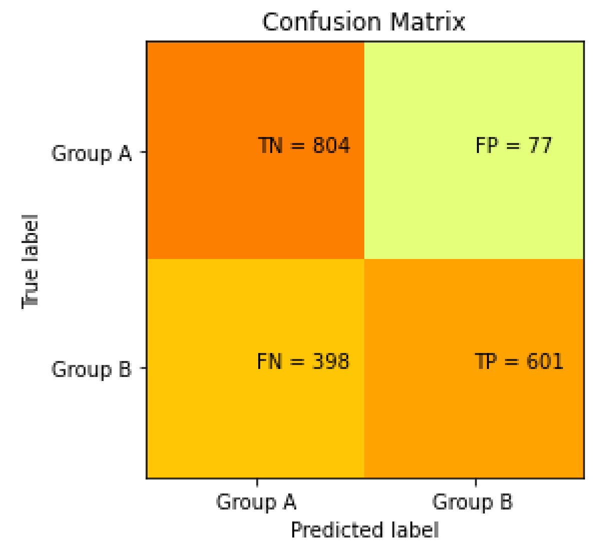

AUC Score : 0.7571004602786667

Accuracy Of the Model(RandomForestClassifier): 0.7473404255319149

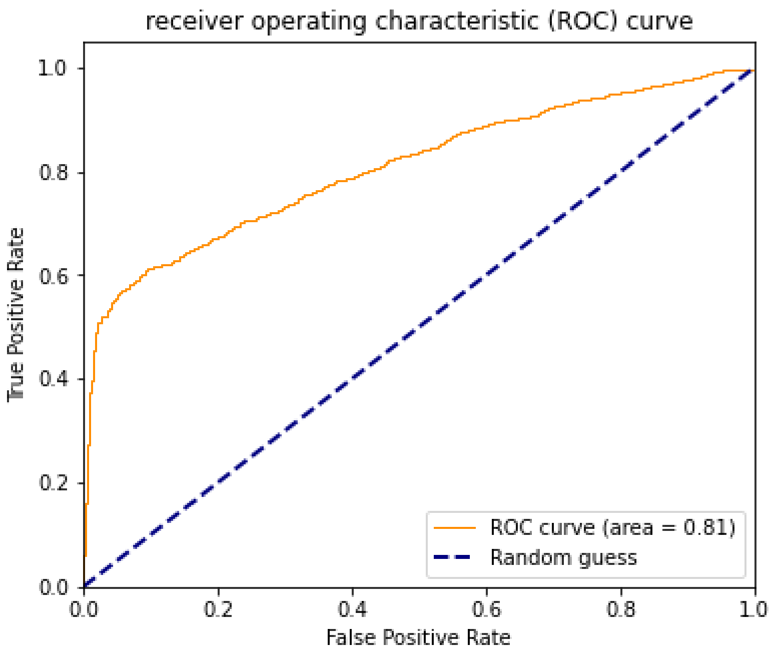

```
ROC_AUC Score of the model(RandomForestClassifier): 0.8100643208475218


Confusion Matrix:
[[810  71]
 [404 595]]

Classification Report:
              precision    recall  f1-score   support

         0.0       0.67      0.92      0.77       881
         1.0       0.89      0.60      0.71       999

    accuracy                           0.75      1880
   macro avg       0.78      0.76      0.74      1880
weighted avg       0.79      0.75      0.74      1880
```

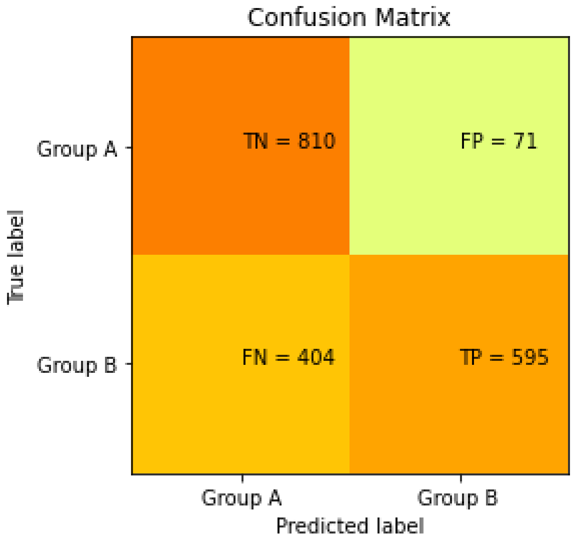


```
AUC Score : 0.757502678615051

Accuracy Of the Model(XGBClassifier): 0.7473404255319149
```

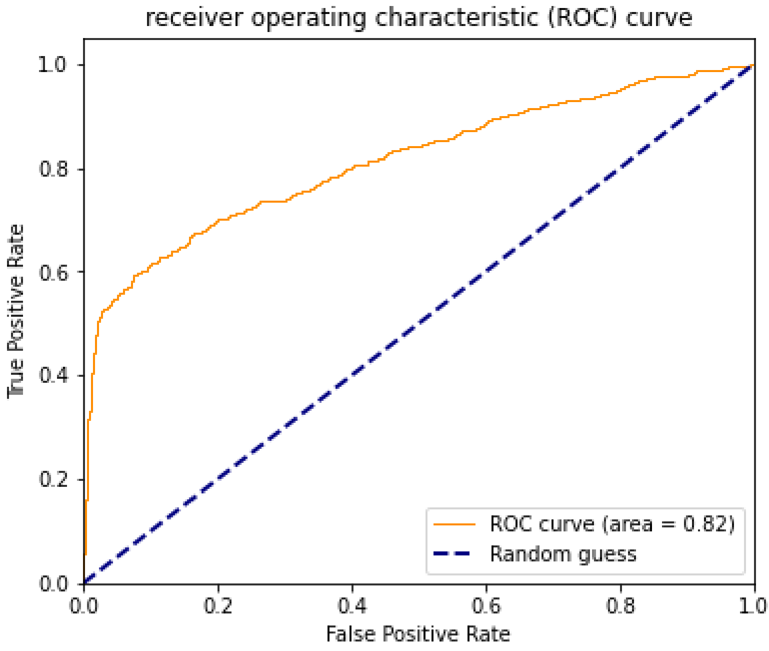

```
ROC_AUC Score of the model(XGBClassifier): 0.8151125018321386


Confusion Matrix:
[[797  84]
 [459 540]]

Classification Report:
              precision    recall  f1-score   support

         0.0       0.63      0.90      0.75       881
         1.0       0.87      0.54      0.67       999

    accuracy                           0.71      1880
   macro avg       0.75      0.72      0.71      1880
weighted avg       0.76      0.71      0.70      1880
```

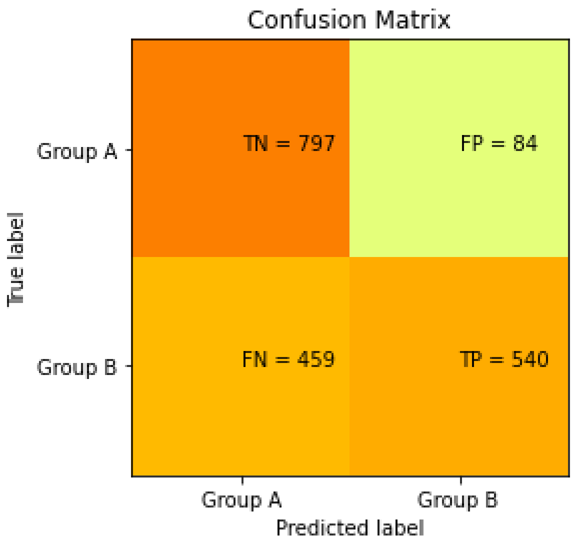


```
AUC Score : 0.7225971715188514

Accuracy Of the Model(KNeighborsClassifier): 0.7111702127659575
```

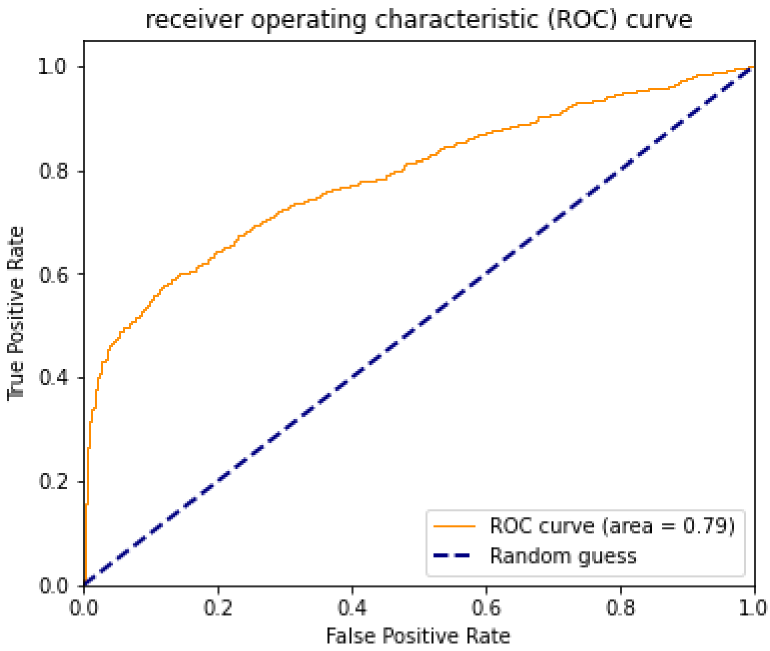

ROC_AUC Score of the model(KNeighborsClassifier): 0.7879888969559798

```
Confusion Matrix:
[[791  90]
 [407 592]]

Classification Report:
              precision    recall  f1-score   support

         0.0       0.66      0.90      0.76       881
         1.0       0.87      0.59      0.70       999

    accuracy                           0.74      1880
   macro avg       0.76      0.75      0.73      1880
weighted avg       0.77      0.74      0.73      1880
```

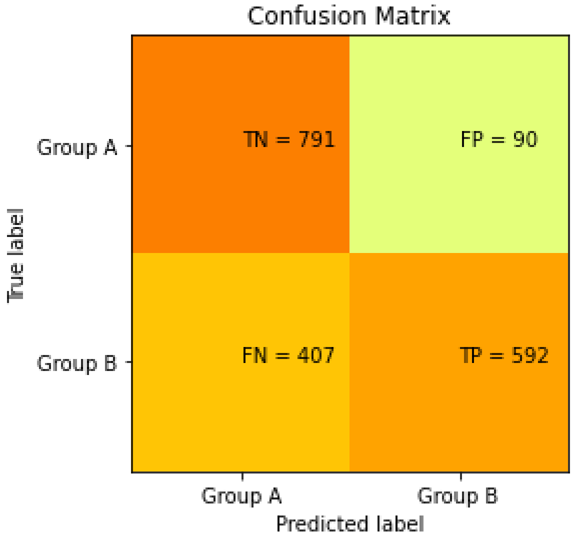

AUC Score : 0.7452179762054904

Accuracy Of the Model(DecisionTreeClassifier): 0.7356382978723405

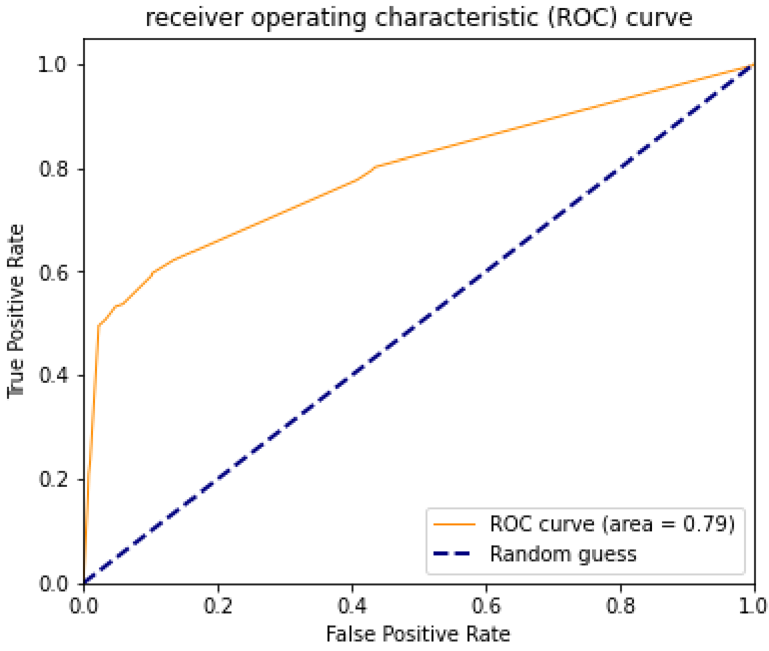

ROC_AUC Score of the model(DecisionTreeClassifier): 0.7903550542597081

```
Confusion Matrix:
[[793  88]
 [412 587]]

Classification Report:
              precision    recall  f1-score   support

         0.0       0.66      0.90      0.76       881
         1.0       0.87      0.59      0.70       999

    accuracy                           0.73      1880
   macro avg       0.76      0.74      0.73      1880
weighted avg       0.77      0.73      0.73      1880
```

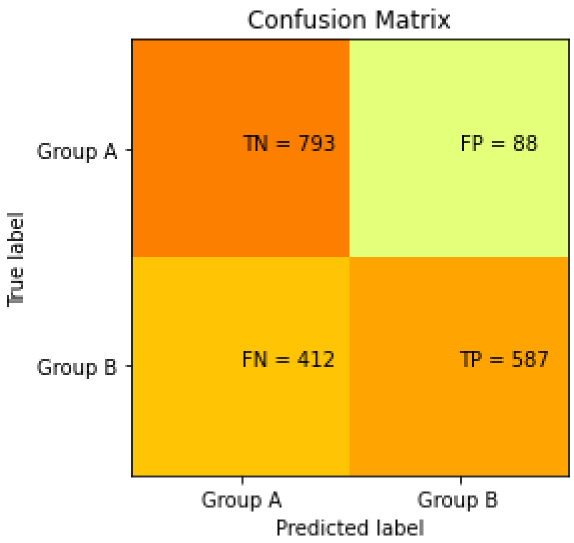

AUC Score : 0.7438505474827836

Accuracy Of the Model(ExtraTreesClassifier): 0.7340425531914894

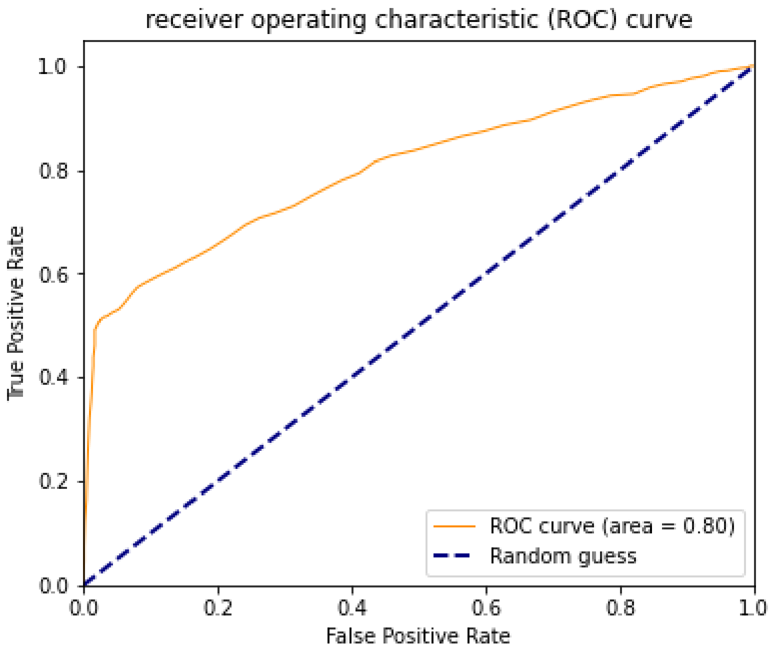

```
ROC_AUC Score of the model(ExtraTreesClassifier): 0.8018097552717303


Confusion Matrix:
[[810  71]
 [407 592]]

Classification Report:
              precision    recall  f1-score   support

         0.0       0.67      0.92      0.77       881
         1.0       0.89      0.59      0.71       999

    accuracy                           0.75      1880
   macro avg       0.78      0.76      0.74      1880
weighted avg       0.79      0.75      0.74      1880
```

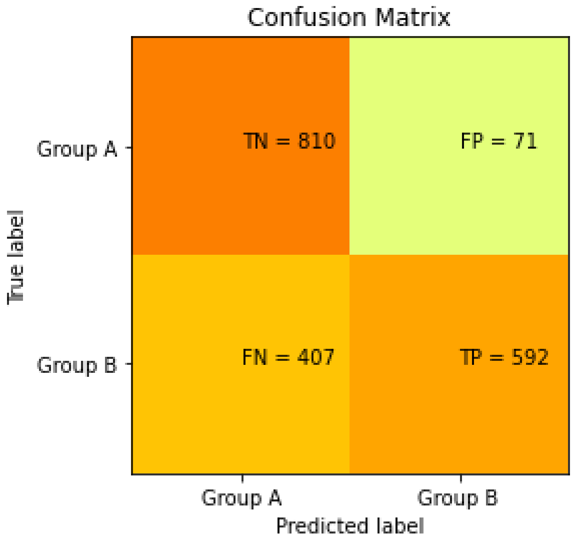


```
AUC Score : 0.7560011771135494

Accuracy Of the Model(GradientBoostingClassifier): 0.7457446808510638
```

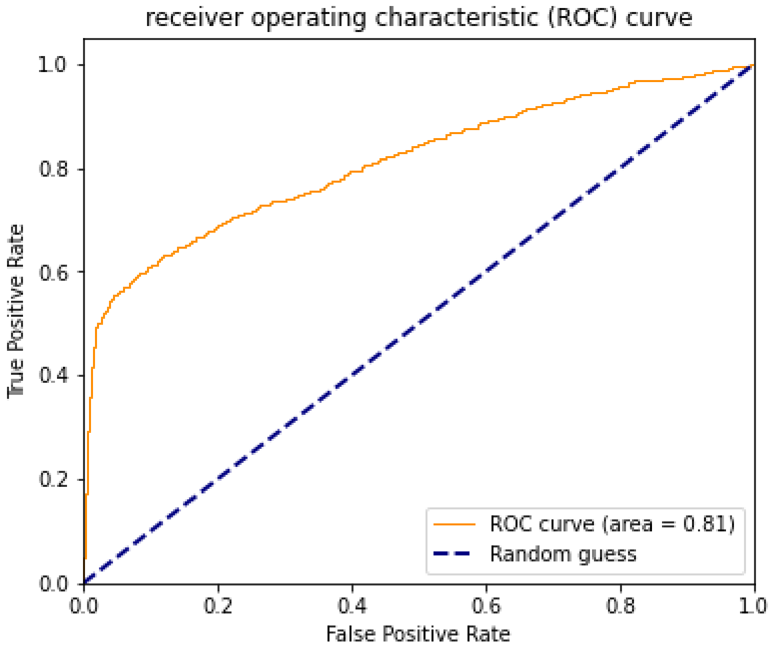

```
ROC_AUC Score of the model(GradientBoostingClassifier): 0.8133650108678485


Confusion Matrix:
[[827  54]
 [427 572]]

Classification Report:
              precision    recall  f1-score   support

         0.0       0.66      0.94      0.77       881
         1.0       0.91      0.57      0.70       999

    accuracy                           0.74      1880
   macro avg       0.79      0.76      0.74      1880
weighted avg       0.79      0.74      0.74      1880
```

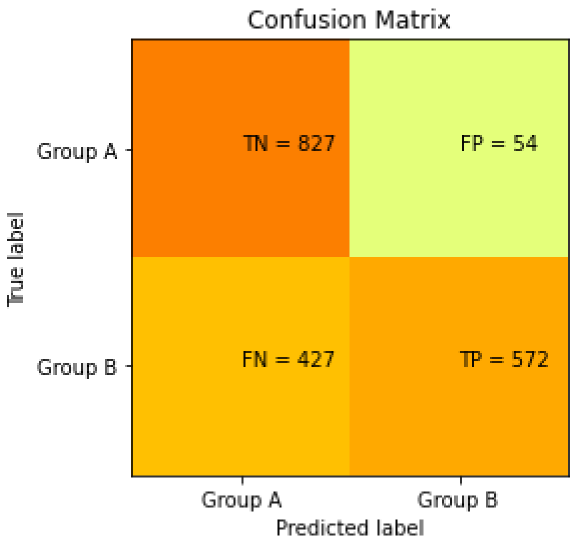


```
AUC Score : 0.7556392942318029

Accuracy Of the Model(AdaBoostClassifier): 0.7441489361702127
```

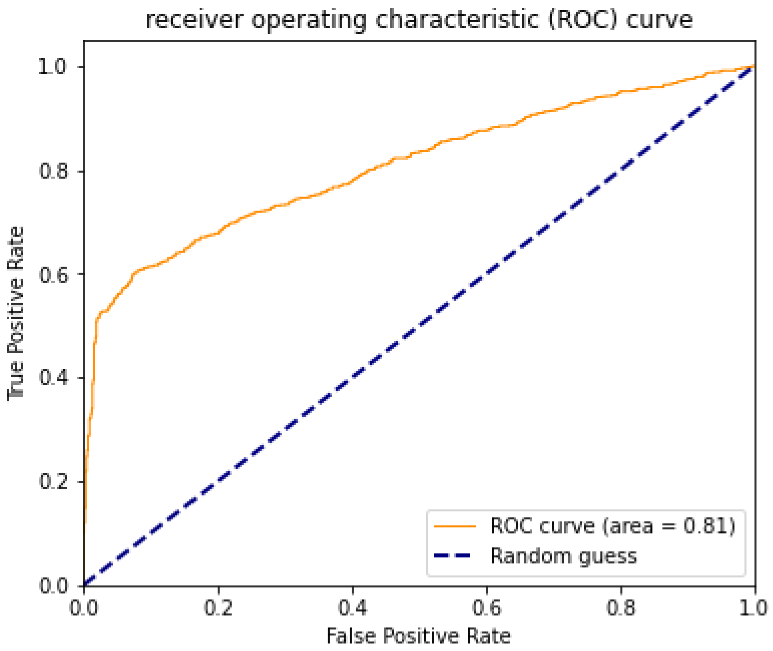

```
ROC_AUC Score of the model(AdaBoostClassifier): 0.8073232142471642


Confusion Matrix:
[[707 174]
 [347 652]]

Classification Report:
              precision    recall  f1-score   support

         0.0       0.67      0.80      0.73       881
         1.0       0.79      0.65      0.71       999

    accuracy                           0.72      1880
   macro avg       0.73      0.73      0.72      1880
weighted avg       0.73      0.72      0.72      1880
```

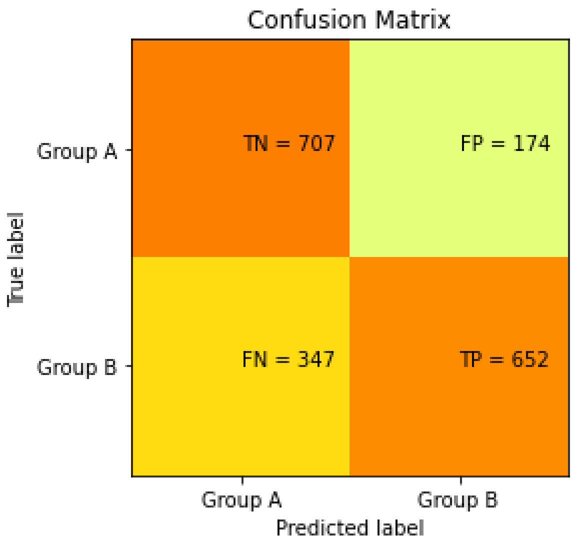


```
AUC Score : 0.7275749074841016

Accuracy Of the Model(SVC): 0.722872340425532
```

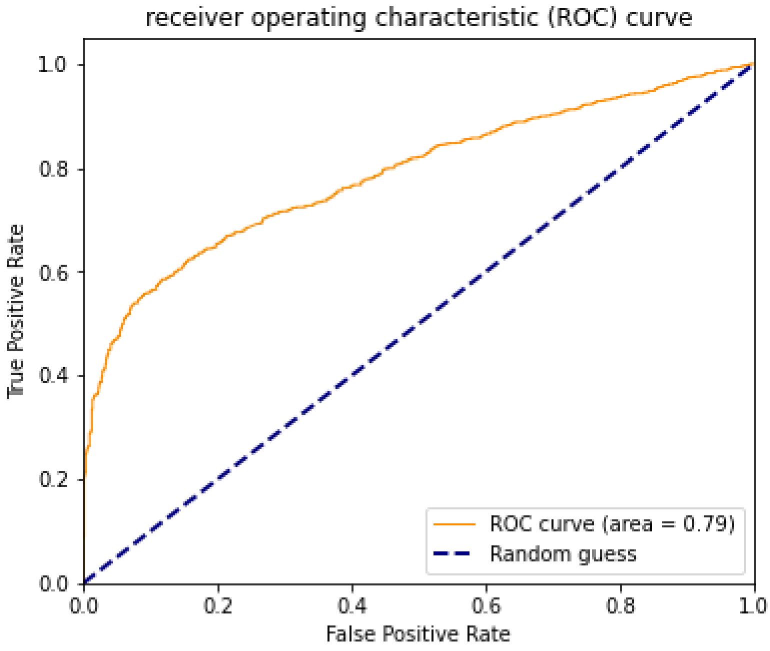

```
ROC_AUC Score of the model(SVC): 0.7869651717551832


Confusion Matrix:
[[802  79]
 [421 578]]

Classification Report:
              precision    recall  f1-score   support

         0.0       0.66      0.91      0.76       881
         1.0       0.88      0.58      0.70       999

    accuracy                           0.73      1880
   macro avg       0.77      0.74      0.73      1880
weighted avg       0.77      0.73      0.73      1880
```

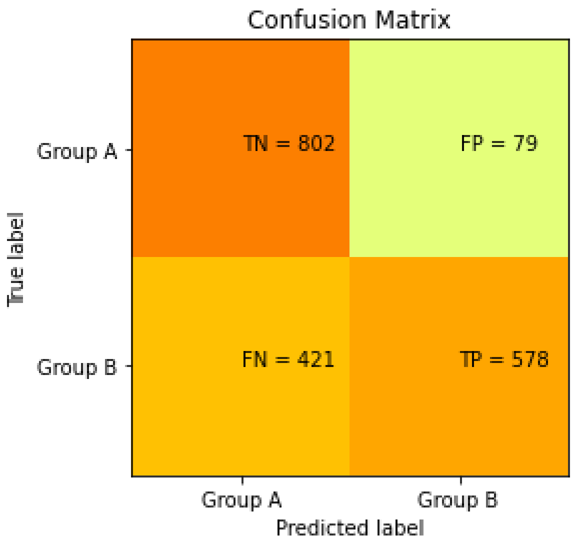


```
AUC Score : 0.7444538749873597

Accuracy Of the Model(MLPClassifier): 0.7340425531914894
```

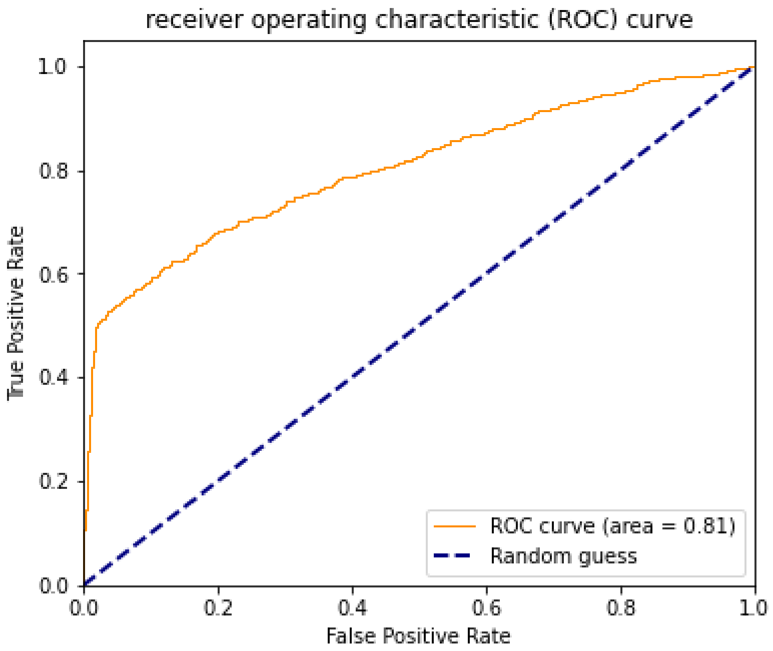

```
ROC_AUC Score of the model(MLPClassifier): 0.8053797270596363

[('RandomForestClassifier', 0.7473404255319149), ('XGBClassifier', 0.7473404255319149), ('GradientBoostingClassifier', 0.7457446808510638), ('AdaBoostClassifier', 0.7441489361702127), ('LogisticRegression', 0.7425531914893617), ('DecisionTreeClassifier', 0.7356382978723405), ('ExtraTreesClassifier', 0.7340425531914894), ('MLPClassifier', 0.7340425531914894), ('SVC', 0.722872340425532), ('KNeighborsClassifier', 0.7111702127659575)]


sorted_total_auc:
 [('XGBClassifier', 0.8151125018321386), ('GradientBoostingClassifier', 0.8133650108678485), ('RandomForestClassifier', 0.8100643208475218), ('AdaBoostClassifier', 0.8073232142471642), ('MLPClassifier', 0.8053797270596363), ('ExtraTreesClassifier', 0.8018097552717303), ('LogisticRegression', 0.8015097958344269), ('DecisionTreeClassifier', 0.7903550542597081), ('KNeighborsClassifier', 0.7879888969559798), ('SVC', 0.7869651717551832)]
```

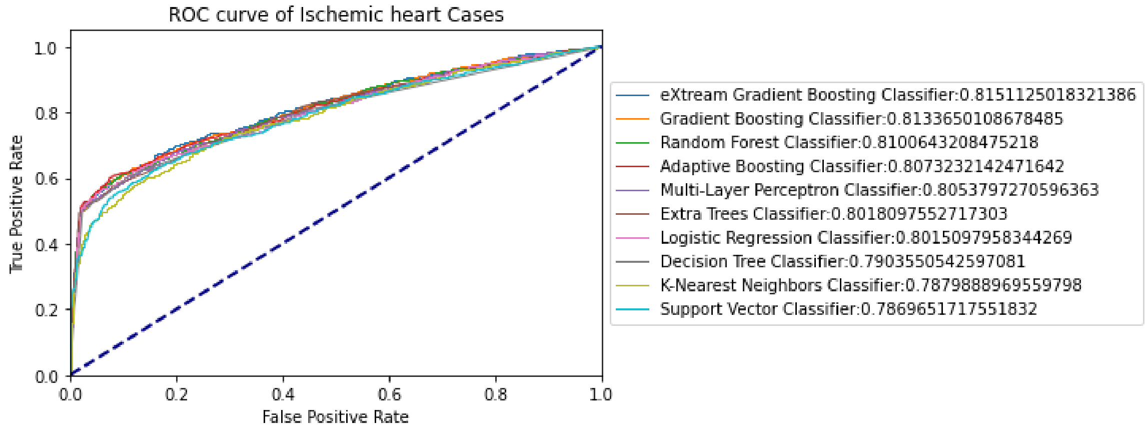

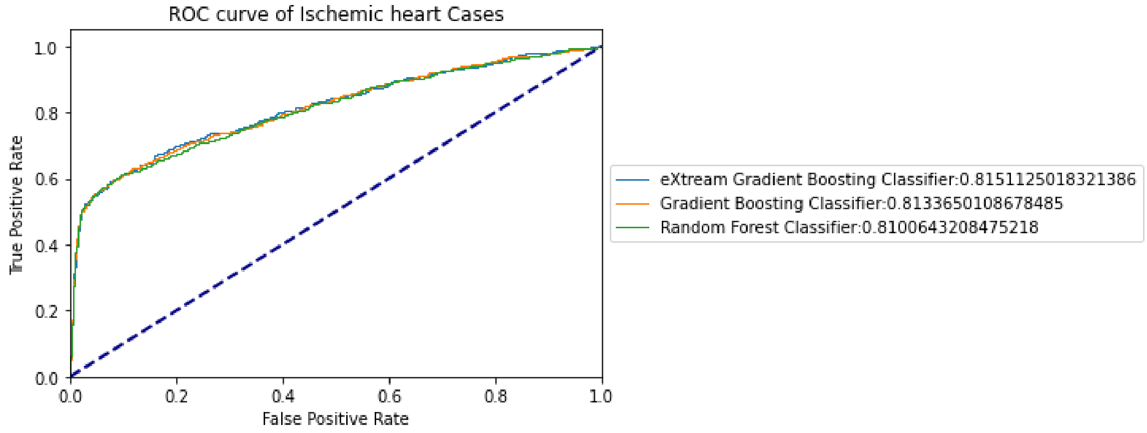

```
random state :   12555
9398
result_train_filename: data/train_test_data_ischemic heart_2013_20210519_133123.cs
v
total count : 9398, df_train count : 7518, df_test count : 1880
(7518, 38)
(1880, 38)
('Logistic Regression', LogisticRegression())
[2021-05-19 13:31:24.072816] Start parameter search for model 'Logistic Regressio
n'
[2021-05-19 13:31:24.401034] Finish parameter search for model 'Logistic Regressio
n' (time: 0 seconds)

=== train : best params ===
{'C': 1, 'penalty': 'l2', 'solver': 'lbfgs'}
=== train : best score : roc_auc ===
0.8055629097646825


('Random Forest', RandomForestClassifier())
[2021-05-19 13:31:24.401034] Start parameter search for model 'Random Forest'
[2021-05-19 13:31:29.351373] Finish parameter search for model 'Random Forest' (ti
me: 4 seconds)

=== train : best params ===
{'criterion': 'gini', 'max_depth': 10, 'max_features': 'auto', 'n_estimators': 10
0}
=== train : best score : roc_auc ===
0.8107683574285331


('Extreme Gradient Boosting)', XGBClassifier(base_score=None, booster=None, colsam
ple_bylevel=None,
              colsample_bynode=None, colsample_bytree=None, gamma=None,
              gpu_id=None, importance_type='gain', interaction_constraints=None,
              learning_rate=None, max_delta_step=None, max_depth=None,
              min_child_weight=None, missing=nan, monotone_constraints=None,
              n_estimators=100, n_jobs=None, num_parallel_tree=None,
              random_state=None, reg_alpha=None, reg_lambda=None,
              scale_pos_weight=None, subsample=None, tree_method=None,
              validate_parameters=None, verbosity=None))
[2021-05-19 13:31:29.351373] Start parameter search for model 'Extreme Gradient Bo
osting)'
[2021-05-19 13:31:59.810487] Finish parameter search for model 'Extreme Gradient B
oosting)' (time: 30 seconds)

=== train : best params ===
{'learning_rate': 0.1, 'max_depth': 2, 'n_estimators': 100, 'subsample': 0.8}
=== train : best score : roc_auc ===
0.8103190633595132


('k-Nearest Neighbours)', KNeighborsClassifier())
[2021-05-19 13:31:59.812481] Start parameter search for model 'k-Nearest Neighbour
s)'
[2021-05-19 13:32:08.354340] Finish parameter search for model 'k-Nearest Neighbou
rs)' (time: 8 seconds)

=== train : best params ===
{'n_neighbors': 60, 'weights': 'distance'}
=== train : best score : roc_auc ===
```

```
0.788206564922438


('Decision Tree', DecisionTreeClassifier())
[2021-05-19 13:32:08.355337] Start parameter search for model 'Decision Tree'
[2021-05-19 13:32:09.023342] Finish parameter search for model 'Decision Tree' (ti
me: 0 seconds)

=== train : best params ===
{'criterion': 'entropy', 'max_depth': 4}
=== train : best score : roc_auc ===
0.7902629423278188


('Extra Tree', ExtraTreesClassifier())
[2021-05-19 13:32:09.024337] Start parameter search for model 'Extra Tree'
[2021-05-19 13:32:12.503104] Finish parameter search for model 'Extra Tree' (time:
3 seconds)

=== train : best params ===
{'max_features': 'log2', 'n_estimators': 100}
=== train : best score : roc_auc ===
0.8007262830673634


('Gradient Boosting', GradientBoostingClassifier())
[2021-05-19 13:32:12.503104] Start parameter search for model 'Gradient Boosting'
[2021-05-19 13:32:31.388745] Finish parameter search for model 'Gradient Boosting'
(time: 18 seconds)

=== train : best params ===
{'learning_rate': 0.075, 'max_depth': 5, 'max_features': 6, 'n_estimators': 50}
=== train : best score : roc_auc ===
0.8104699354229447


('Adaptive Boosting', AdaBoostClassifier())
[2021-05-19 13:32:31.388745] Start parameter search for model 'Adaptive Boosting'
[2021-05-19 13:32:35.083964] Finish parameter search for model 'Adaptive Boosting'
(time: 3 seconds)

=== train : best params ===
{'learning_rate': 0.2, 'n_estimators': 50}
=== train : best score : roc_auc ===
0.8044366980495044


('Suppport Vector Classfier', SVC(probability=True))
[2021-05-19 13:32:35.084962] Start parameter search for model 'Suppport Vector Cla
ssfier'
[2021-05-19 13:36:30.863342] Finish parameter search for model 'Suppport Vector Cl
assfier' (time: 235 seconds)

=== train : best params ===
{'C': 1, 'gamma': 0.1, 'kernel': 'rbf'}
=== train : best score : roc_auc ===
0.7838822570054371


('Neural Network', MLPClassifier())
[2021-05-19 13:36:30.864339] Start parameter search for model 'Neural Network'
```

```
[2021-05-19 13:38:44.369565] Finish parameter search for model 'Neural Network' (t
ime: 133 seconds)

=== train : best params ===
{'activation': 'logistic', 'alpha': 0.05, 'hidden_layer_sizes': (50,), 'learning_r
ate': 'adaptive'}
=== train : best score : roc_auc ===
0.812601293461918



Confusion Matrix:
[[824  57]
 [429 570]]

Classification Report:
              precision    recall  f1-score   support

         0.0       0.66      0.94      0.77       881
         1.0       0.91      0.57      0.70       999

    accuracy                           0.74      1880
   macro avg       0.78      0.75      0.74      1880
weighted avg       0.79      0.74      0.73      1880
```

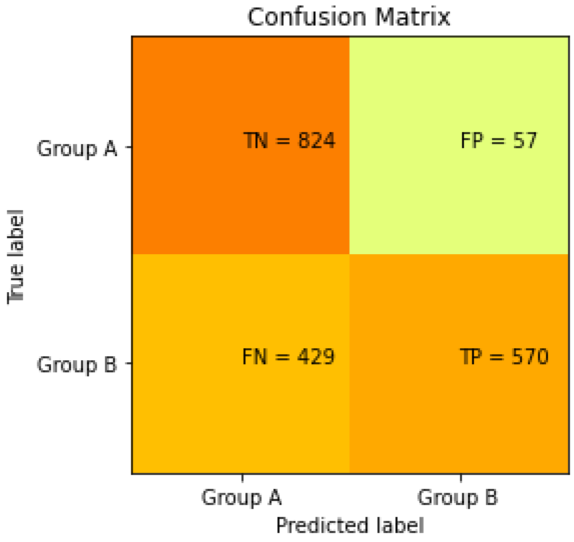


```
AUC Score : 0.7529356825611082

Accuracy Of the Model(LogisticRegression): 0.7414893617021276
```

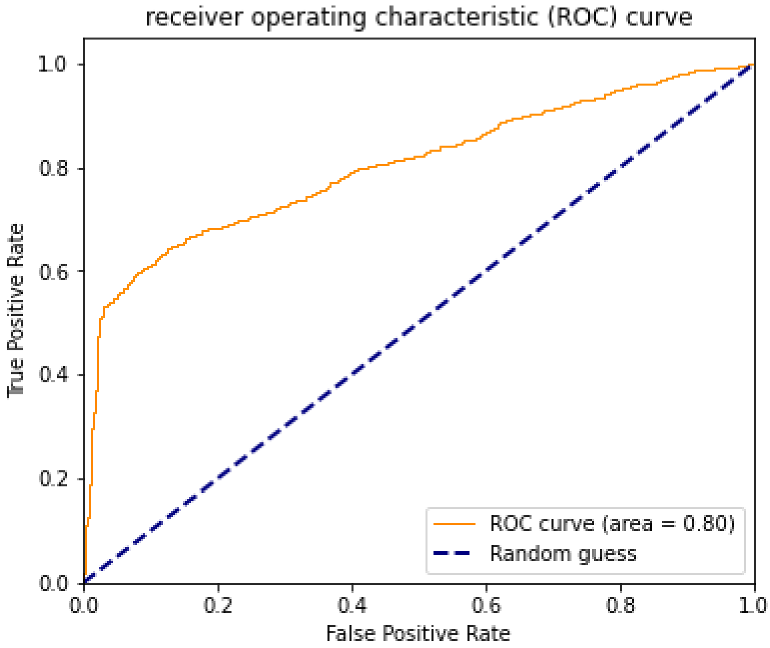


```
ROC_AUC Score of the model(LogisticRegression): 0.8041321684908518


Confusion Matrix:
[[804  77]
 [404 595]]

Classification Report:
              precision    recall  f1-score   support

         0.0       0.67      0.91      0.77       881
         1.0       0.89      0.60      0.71       999

    accuracy                           0.74      1880
   macro avg       0.78      0.75      0.74      1880
weighted avg       0.78      0.74      0.74      1880
```

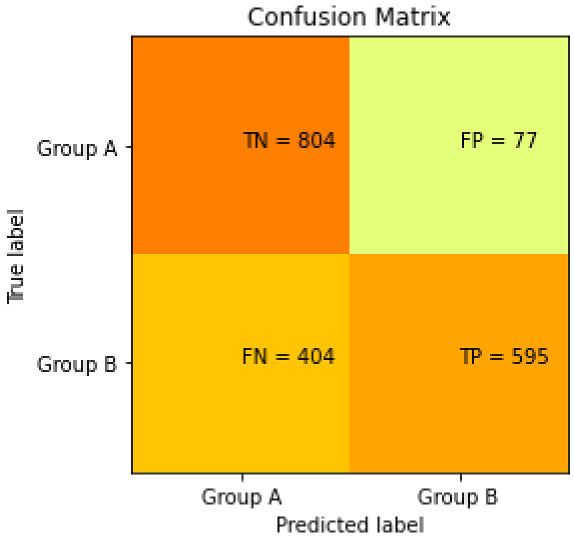


```
AUC Score : 0.7540974572756638

Accuracy Of the Model(RandomForestClassifier): 0.7441489361702127
```

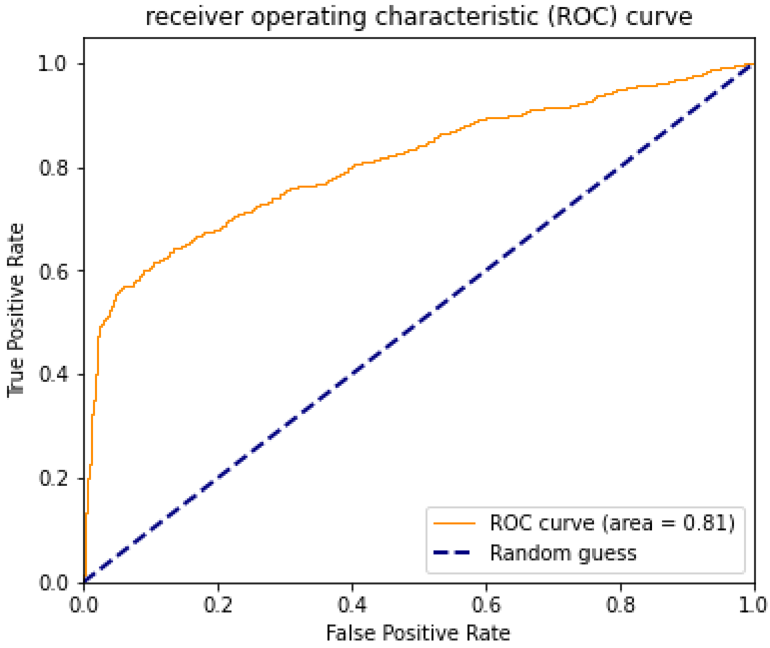

```
ROC_AUC Score of the model(RandomForestClassifier): 0.8099529722685228


Confusion Matrix:
[[820  61]
 [412 587]]

Classification Report:
              precision    recall  f1-score   support

         0.0       0.67      0.93      0.78       881
         1.0       0.91      0.59      0.71       999

    accuracy                           0.75      1880
   macro avg       0.79      0.76      0.74      1880
weighted avg       0.79      0.75      0.74      1880
```

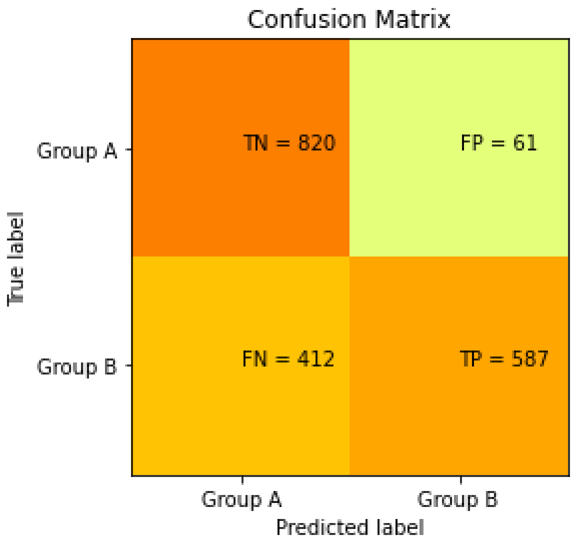


```
AUC Score : 0.7591740435100253

Accuracy Of the Model(XGBClassifier): 0.7484042553191489
```

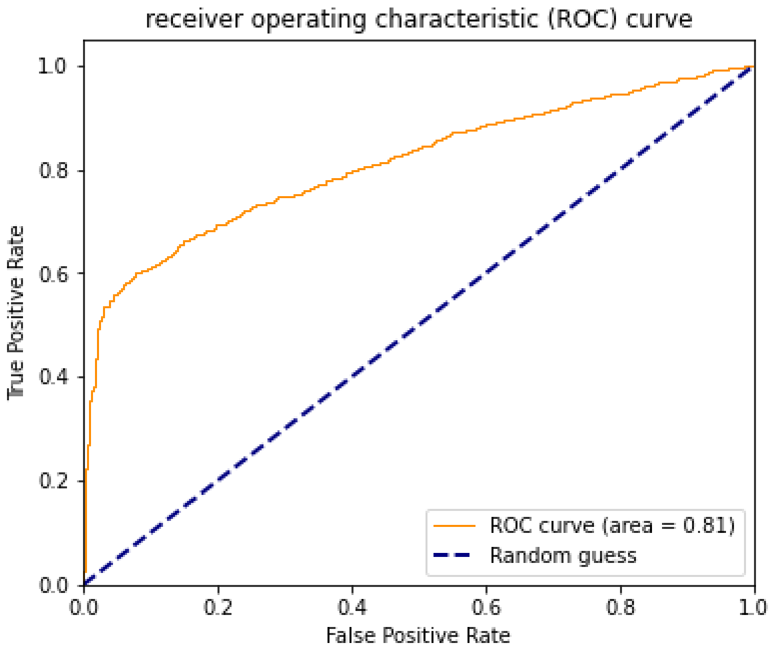

```
ROC_AUC Score of the model(XGBClassifier): 0.8132604795487883


Confusion Matrix:
[[800  81]
 [456 543]]

Classification Report:
              precision    recall  f1-score   support

         0.0       0.64      0.91      0.75       881
         1.0       0.87      0.54      0.67       999

    accuracy                           0.71      1880
   macro avg       0.75      0.73      0.71      1880
weighted avg       0.76      0.71      0.71      1880
```

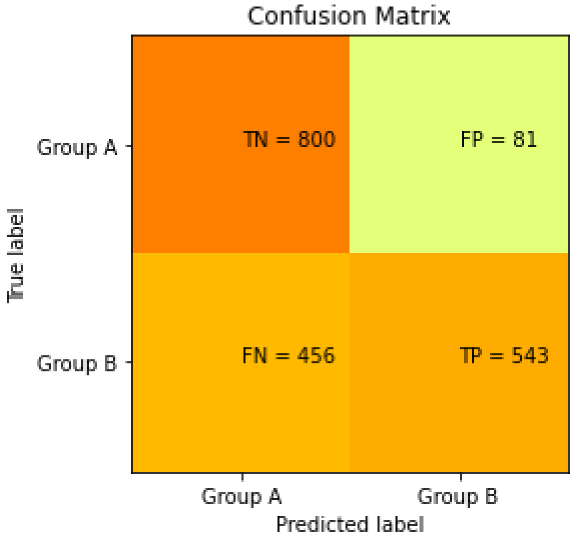


```
AUC Score : 0.7258012836900465

Accuracy Of the Model(KNeighborsClassifier): 0.7143617021276596
```

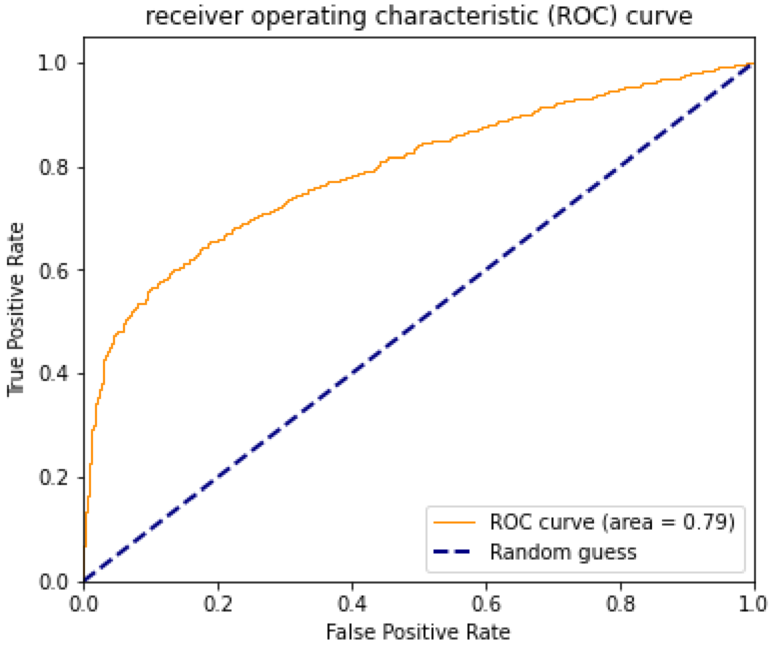

```
ROC_AUC Score of the model(KNeighborsClassifier): 0.7947743430149787


Confusion Matrix:
[[849  32]
 [486 513]]

Classification Report:
              precision    recall  f1-score   support

         0.0       0.64      0.96      0.77       881
         1.0       0.94      0.51      0.66       999

    accuracy                           0.72      1880
   macro avg       0.79      0.74      0.72      1880
weighted avg       0.80      0.72      0.71      1880
```

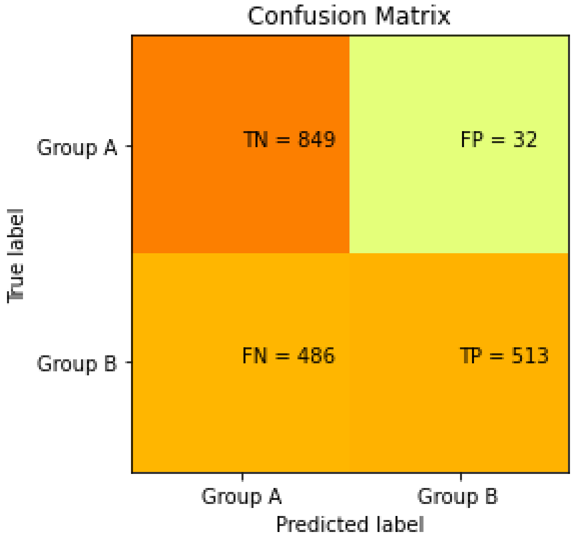


```
AUC Score : 0.7385955762800258

Accuracy Of the Model(DecisionTreeClassifier): 0.7244680851063829
```

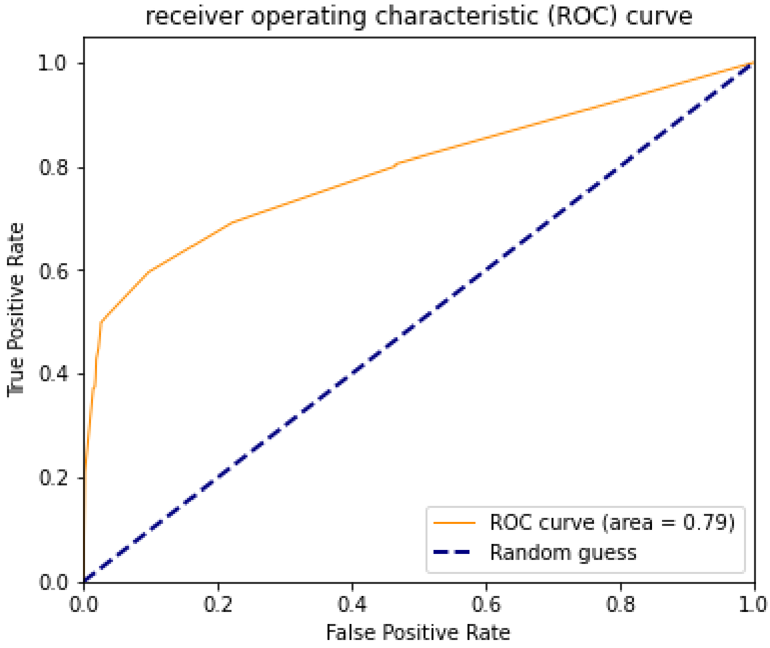

ROC_AUC Score of the model(DecisionTreeClassifier): 0.7922065084380635

Confusion Matrix:
[[806 75]
 [431 568]]

Classification Report:

	precision	recall	f1-score	support
0.0	0.65	0.91	0.76	881
1.0	0.88	0.57	0.69	999
accuracy			0.73	1880
macro avg	0.77	0.74	0.73	1880
weighted avg	0.77	0.73	0.72	1880

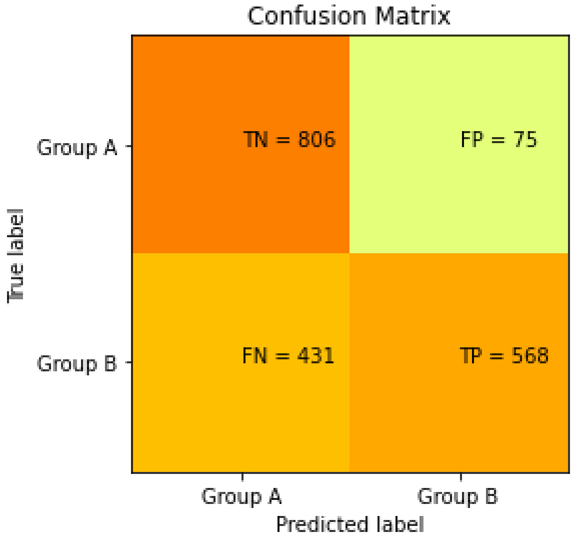

AUC Score : 0.7417190175419461

Accuracy Of the Model(ExtraTreesClassifier): 0.7308510638297873

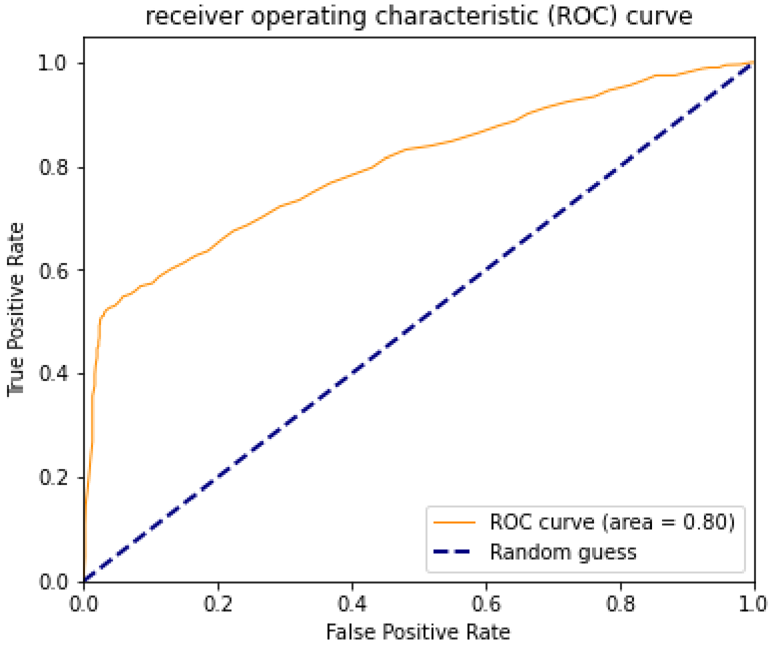

ROC_AUC Score of the model(ExtraTreesClassifier): 0.7988379980434464

Confusion Matrix:
[[808 73]
 [413 586]]

Classification Report:

	precision	recall	f1-score	support
0.0	0.66	0.92	0.77	881
1.0	0.89	0.59	0.71	999
accuracy			0.74	1880
macro avg	0.78	0.75	0.74	1880
weighted avg	0.78	0.74	0.74	1880

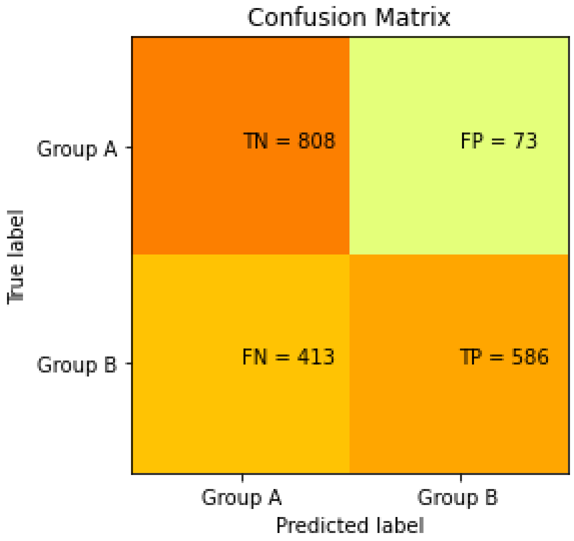

AUC Score : 0.7518631003307507

Accuracy Of the Model(GradientBoostingClassifier): 0.7414893617021276

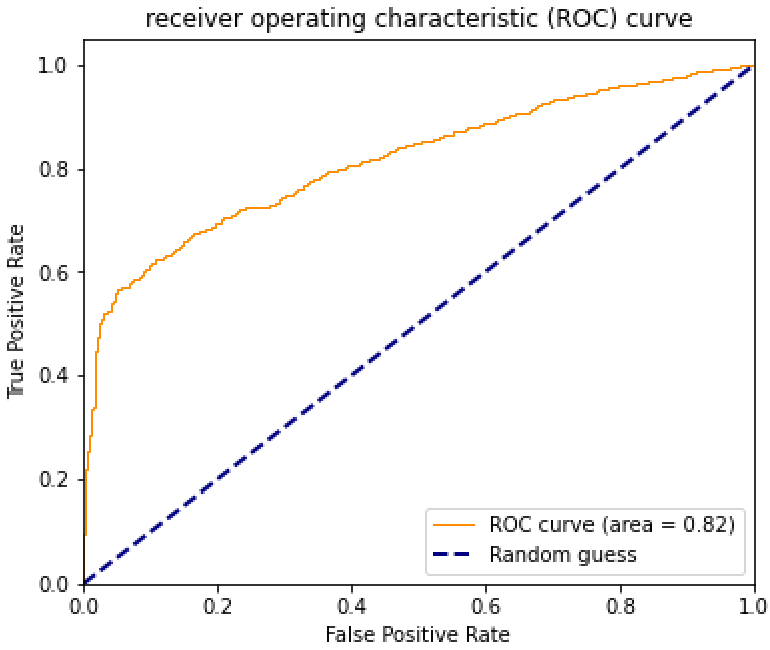

```
ROC_AUC Score of the model(GradientBoostingClassifier): 0.8168656738463775


Confusion Matrix:
[[840  41]
 [451 548]]

Classification Report:
              precision    recall  f1-score   support

         0.0       0.65      0.95      0.77       881
         1.0       0.93      0.55      0.69       999

    accuracy                           0.74      1880
   macro avg       0.79      0.75      0.73      1880
weighted avg       0.80      0.74      0.73      1880
```

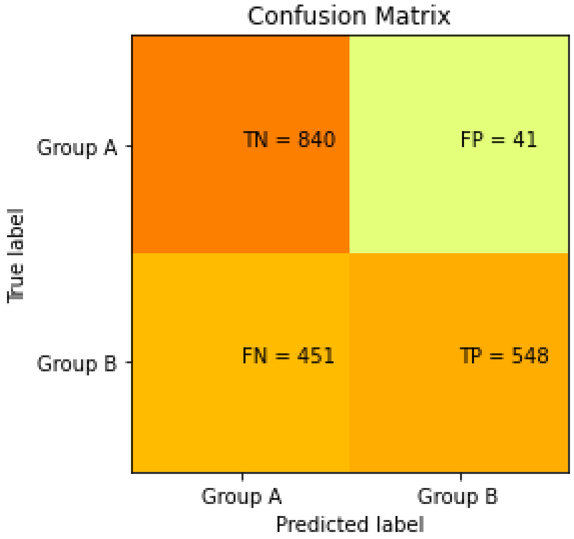


```
AUC Score : 0.7510052617884627

Accuracy Of the Model(AdaBoostClassifier): 0.7382978723404255
```

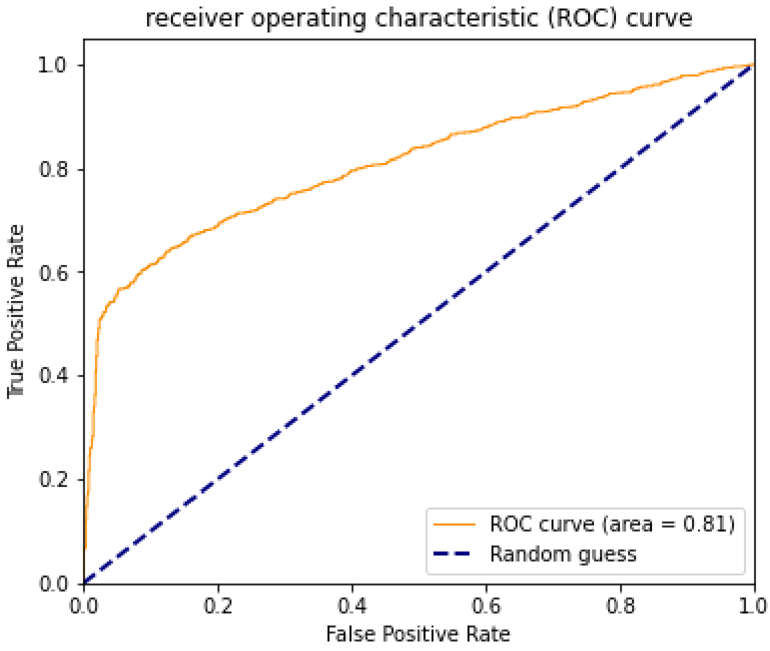

ROC_AUC Score of the model(AdaBoostClassifier): 0.8087957423939263

```
Confusion Matrix:
[[712 169]
 [349 650]]

Classification Report:
              precision    recall  f1-score   support

         0.0       0.67      0.81      0.73       881
         1.0       0.79      0.65      0.72       999

    accuracy                           0.72      1880
   macro avg       0.73      0.73      0.72      1880
weighted avg       0.74      0.72      0.72      1880
```

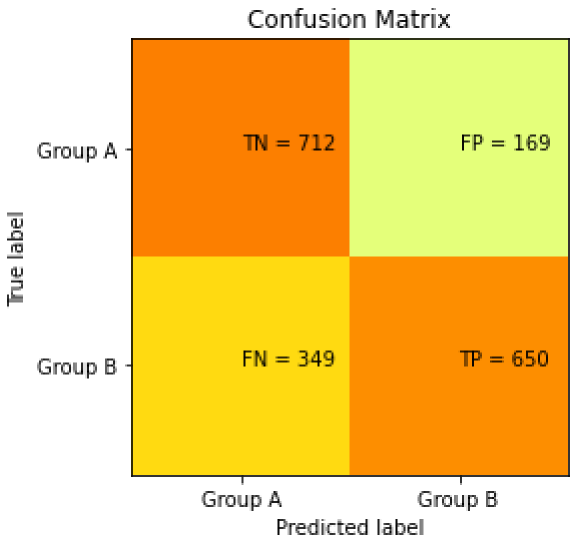

AUC Score : 0.7294115909325898

Accuracy Of the Model(SVC): 0.7244680851063829

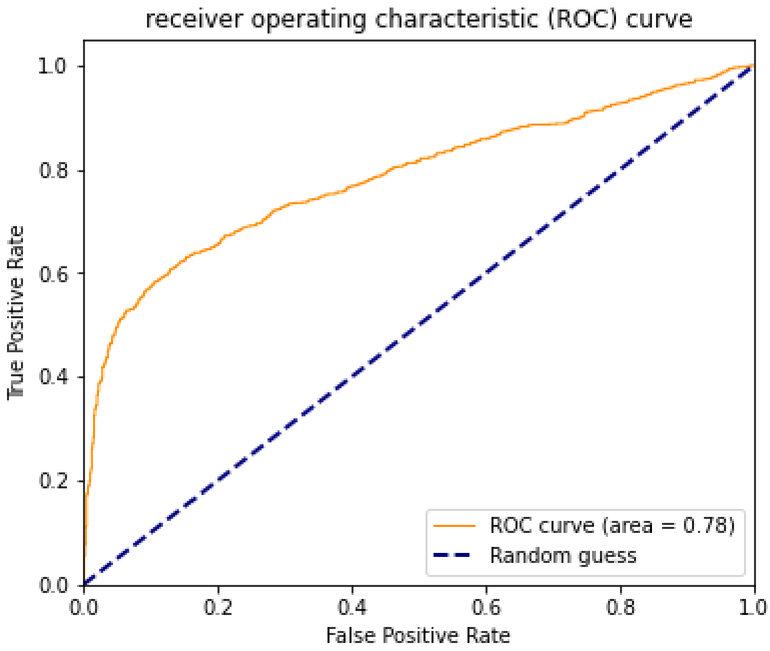

```
ROC_AUC Score of the model(SVC): 0.7844564200977368


Confusion Matrix:
[[795  86]
 [401 598]]

Classification Report:
              precision    recall  f1-score   support

         0.0       0.66      0.90      0.77       881
         1.0       0.87      0.60      0.71       999

    accuracy                           0.74      1880
   macro avg       0.77      0.75      0.74      1880
weighted avg       0.78      0.74      0.74      1880
```

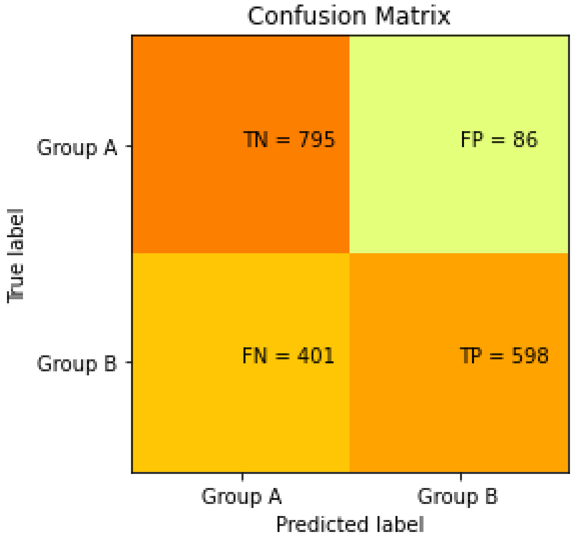


```
AUC Score : 0.7504911267680848

Accuracy Of the Model(MLPClassifier): 0.7409574468085106
```

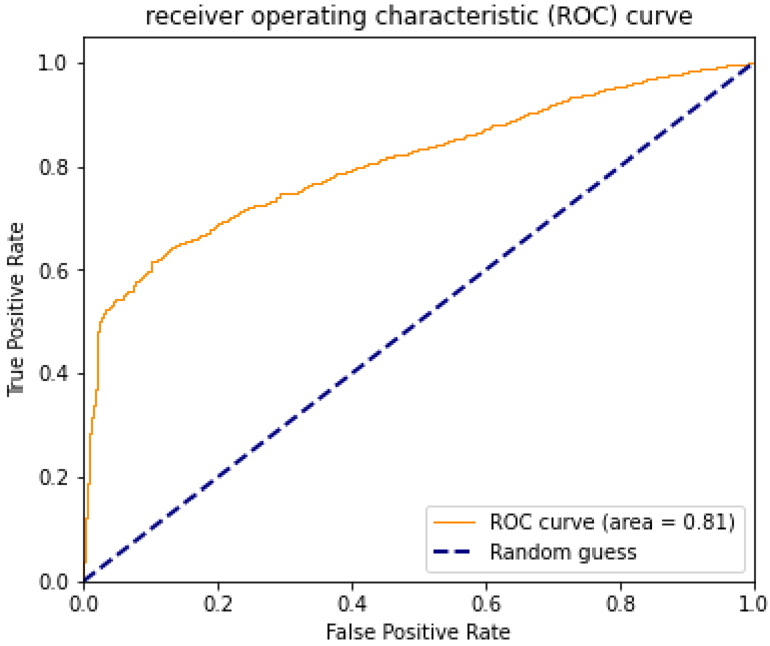

```
ROC_AUC Score of the model(MLPClassifier): 0.8083225109331806

[('XGBClassifier', 0.7484042553191489), ('RandomForestClassifier', 0.7441489361702
127), ('LogisticRegression', 0.7414893617021276), ('GradientBoostingClassifier',
0.7414893617021276), ('MLPClassifier', 0.7409574468085106), ('AdaBoostClassifier',
0.7382978723404255), ('ExtraTreesClassifier', 0.7308510638297873), ('DecisionTreeC
lassifier', 0.7244680851063829), ('SVC', 0.7244680851063829), ('KNeighborsClassifi
er', 0.7143617021276596)]


sorted_total_auc:
 [('GradientBoostingClassifier', 0.8168656738463775), ('XGBClassifier', 0.81326047
95487883), ('RandomForestClassifier', 0.8099529722685228), ('AdaBoostClassifier',
0.8087957423939263), ('MLPClassifier', 0.8083225109331806), ('LogisticRegression',
0.8041321684908518), ('ExtraTreesClassifier', 0.7988379980434464), ('KNeighborsCla
ssifier', 0.7947743430149787), ('DecisionTreeClassifier', 0.7922065084380635), ('S
VC', 0.7844564200977368)]
```

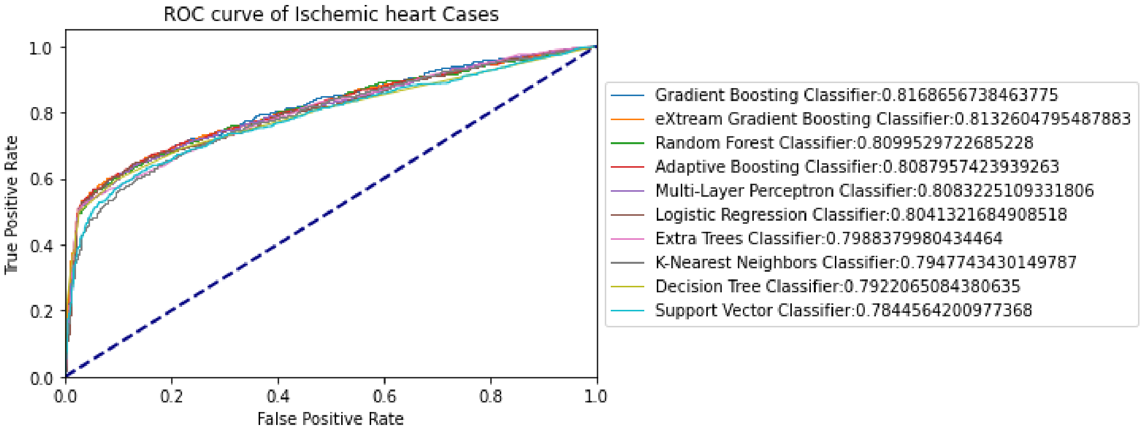

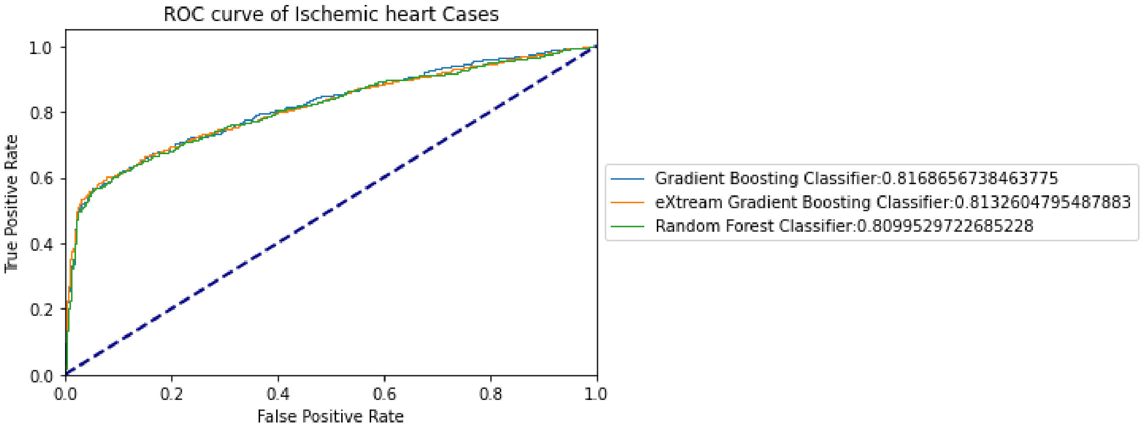

```
random state :   12576
9398
result_train_filename: data/train_test_data_ischemic heart_2013_20210519_133851.cs
v
total count : 9398, df_train count : 7518, df_test count : 1880
(7518, 38)
(1880, 38)
('Logistic Regression', LogisticRegression())
[2021-05-19 13:38:52.112474] Start parameter search for model 'Logistic Regressio
n'
[2021-05-19 13:38:52.462704] Finish parameter search for model 'Logistic Regressio
n' (time: 0 seconds)

=== train : best params ===
{'C': 0.1, 'penalty': 'l2', 'solver': 'lbfgs'}
=== train : best score : roc_auc ===
0.8051741660065046



('Random Forest', RandomForestClassifier())
[2021-05-19 13:38:52.463701] Start parameter search for model 'Random Forest'
[2021-05-19 13:38:57.322075] Finish parameter search for model 'Random Forest' (ti
me: 4 seconds)

=== train : best params ===
{'criterion': 'gini', 'max_depth': 10, 'max_features': 'auto', 'n_estimators': 70}
=== train : best score : roc_auc ===
0.8157239667772952



('Extreme Gradient Boosting)', XGBClassifier(base_score=None, booster=None, colsam
ple_bylevel=None,
              colsample_bynode=None, colsample_bytree=None, gamma=None,
              gpu_id=None, importance_type='gain', interaction_constraints=None,
              learning_rate=None, max_delta_step=None, max_depth=None,
              min_child_weight=None, missing=nan, monotone_constraints=None,
              n_estimators=100, n_jobs=None, num_parallel_tree=None,
              random_state=None, reg_alpha=None, reg_lambda=None,
              scale_pos_weight=None, subsample=None, tree_method=None,
              validate_parameters=None, verbosity=None))
[2021-05-19 13:38:57.323072] Start parameter search for model 'Extreme Gradient Bo
osting)'
[2021-05-19 13:39:27.590835] Finish parameter search for model 'Extreme Gradient B
oosting)' (time: 30 seconds)

=== train : best params ===
{'learning_rate': 0.075, 'max_depth': 5, 'n_estimators': 40, 'subsample': 0.8}
=== train : best score : roc_auc ===
0.8125388334608867



('k-Nearest Neighbours)', KNeighborsClassifier())
[2021-05-19 13:39:27.592829] Start parameter search for model 'k-Nearest Neighbour
s)'
[2021-05-19 13:39:35.784469] Finish parameter search for model 'k-Nearest Neighbou
rs)' (time: 8 seconds)

=== train : best params ===
{'n_neighbors': 60, 'weights': 'distance'}
=== train : best score : roc_auc ===
0.7906454244430796
```

```
('Decision Tree', DecisionTreeClassifier())
[2021-05-19 13:39:35.784469] Start parameter search for model 'Decision Tree'
[2021-05-19 13:39:36.426924] Finish parameter search for model 'Decision Tree' (ti
me: 0 seconds)

=== train : best params ===
{'criterion': 'entropy', 'max_depth': 4}
=== train : best score : roc_auc ===
0.795137029848138


('Extra Tree', ExtraTreesClassifier())
[2021-05-19 13:39:36.426924] Start parameter search for model 'Extra Tree'
[2021-05-19 13:39:39.675694] Finish parameter search for model 'Extra Tree' (time:
3 seconds)

=== train : best params ===
{'max_features': 'log2', 'n_estimators': 100}
=== train : best score : roc_auc ===
0.8057027933845206


('Gradient Boosting', GradientBoostingClassifier())
[2021-05-19 13:39:39.675694] Start parameter search for model 'Gradient Boosting'
[2021-05-19 13:39:57.258242] Finish parameter search for model 'Gradient Boosting'
(time: 17 seconds)

=== train : best params ===
{'learning_rate': 0.01, 'max_depth': 8, 'max_features': 6, 'n_estimators': 100}
=== train : best score : roc_auc ===
0.8128903524965534


('Adaptive Boosting', AdaBoostClassifier())
[2021-05-19 13:39:57.258242] Start parameter search for model 'Adaptive Boosting'
[2021-05-19 13:40:00.673405] Finish parameter search for model 'Adaptive Boosting'
(time: 3 seconds)

=== train : best params ===
{'learning_rate': 0.2, 'n_estimators': 50}
=== train : best score : roc_auc ===
0.8044340824925467


('Suppport Vector Classfier', SVC(probability=True))
[2021-05-19 13:40:00.674418] Start parameter search for model 'Suppport Vector Cla
ssfier'
[2021-05-19 13:43:44.654015] Finish parameter search for model 'Suppport Vector Cl
assfier' (time: 223 seconds)

=== train : best params ===
{'C': 1, 'gamma': 0.1, 'kernel': 'rbf'}
=== train : best score : roc_auc ===
0.7867223196461255


('Neural Network', MLPClassifier())
[2021-05-19 13:43:44.655012] Start parameter search for model 'Neural Network'
[2021-05-19 13:46:26.990245] Finish parameter search for model 'Neural Network' (t
```

```
ime: 162 seconds)

=== train : best params ===
{'activation': 'logistic', 'alpha': 0.05, 'hidden_layer_sizes': (50,), 'learning_r
ate': 'constant'}
=== train : best score : roc_auc ===
0.8108889636966605



Confusion Matrix:
[[806  75]
 [426 573]]

Classification Report:
              precision    recall  f1-score   support

         0.0       0.65      0.91      0.76       881
         1.0       0.88      0.57      0.70       999

    accuracy                           0.73      1880
   macro avg       0.77      0.74      0.73      1880
weighted avg       0.78      0.73      0.73      1880
```

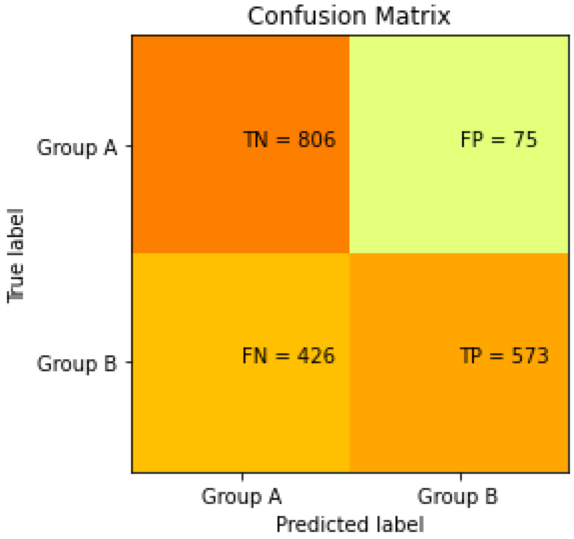


```
AUC Score : 0.7442215200444486

Accuracy Of the Model(LogisticRegression): 0.7335106382978723
```

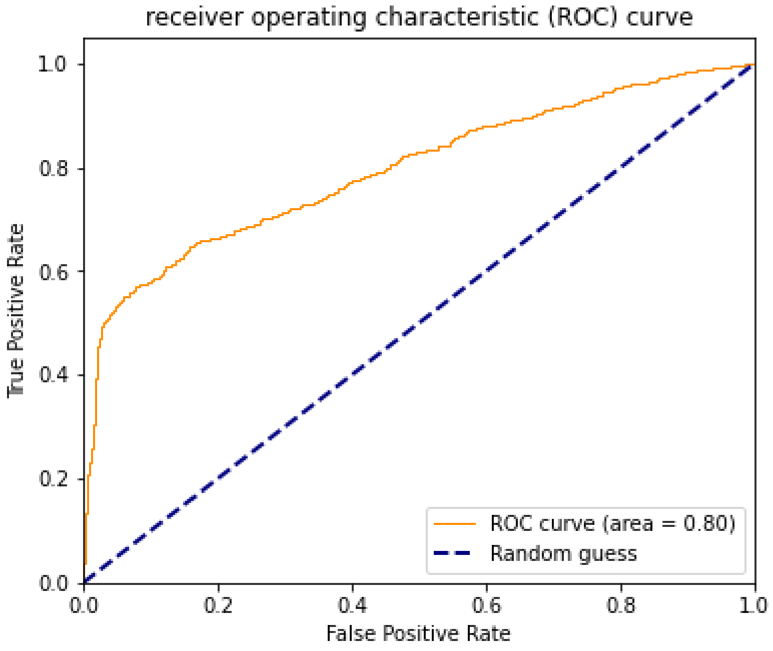


```
ROC_AUC Score of the model(LogisticRegression): 0.7974012605113626


Confusion Matrix:
[[788  93]
 [408 591]]

Classification Report:
              precision    recall  f1-score   support

         0.0       0.66      0.89      0.76       881
         1.0       0.86      0.59      0.70       999

    accuracy                           0.73      1880
   macro avg       0.76      0.74      0.73      1880
weighted avg       0.77      0.73      0.73      1880
```

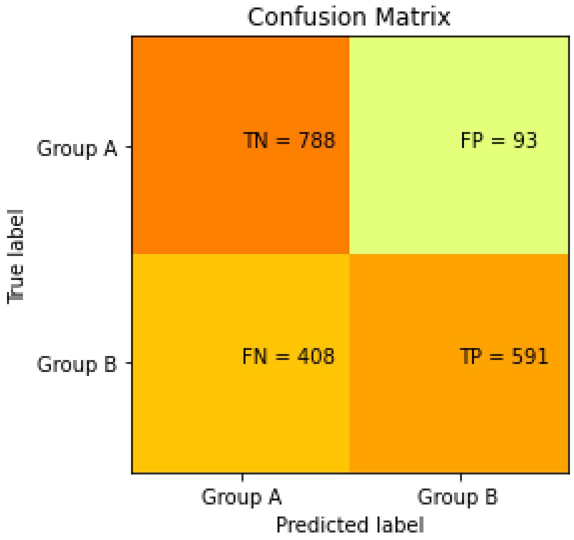

AUC Score : 0.7430148650352962

Accuracy Of the Model(RandomForestClassifier): 0.7335106382978723

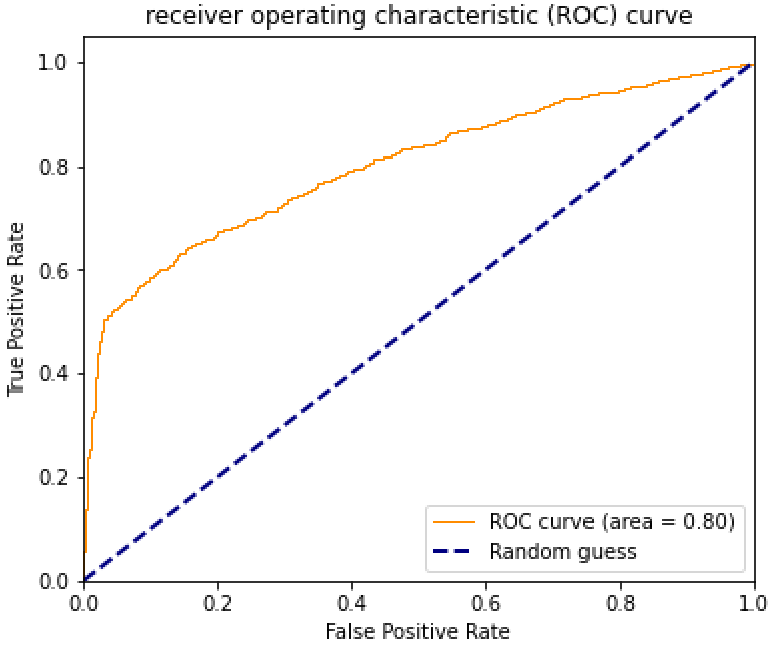

```
ROC_AUC Score of the model(RandomForestClassifier): 0.8021722062584717


Confusion Matrix:
[[805  76]
 [406 593]]

Classification Report:
              precision    recall  f1-score   support

         0.0       0.66      0.91      0.77       881
         1.0       0.89      0.59      0.71       999

    accuracy                           0.74      1880
   macro avg       0.78      0.75      0.74      1880
weighted avg       0.78      0.74      0.74      1880
```

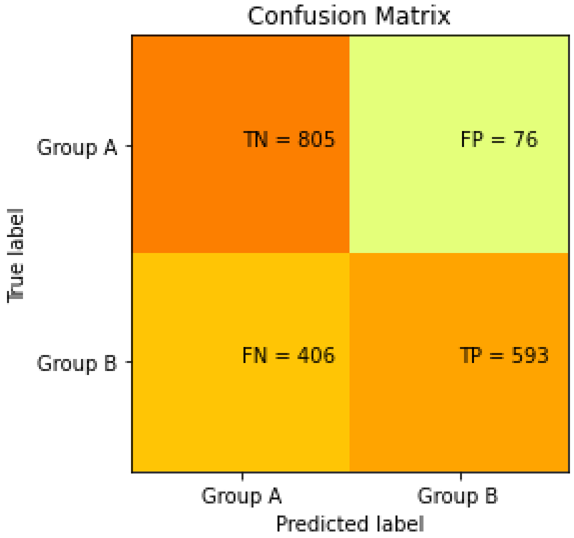


```
AUC Score : 0.7536639931645608

Accuracy Of the Model(XGBClassifier): 0.7436170212765958
```

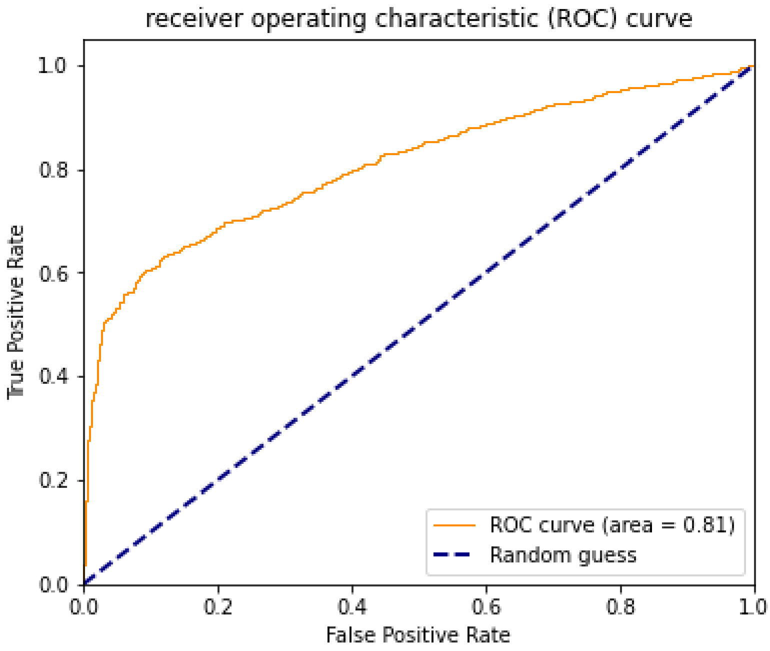

ROC_AUC Score of the model(XGBClassifier): 0.8091326286558976

```
Confusion Matrix:
[[799  82]
 [464 535]]

Classification Report:
              precision    recall  f1-score   support

         0.0       0.63      0.91      0.75       881
         1.0       0.87      0.54      0.66       999

    accuracy                           0.71      1880
   macro avg       0.75      0.72      0.70      1880
weighted avg       0.76      0.71      0.70      1880
```

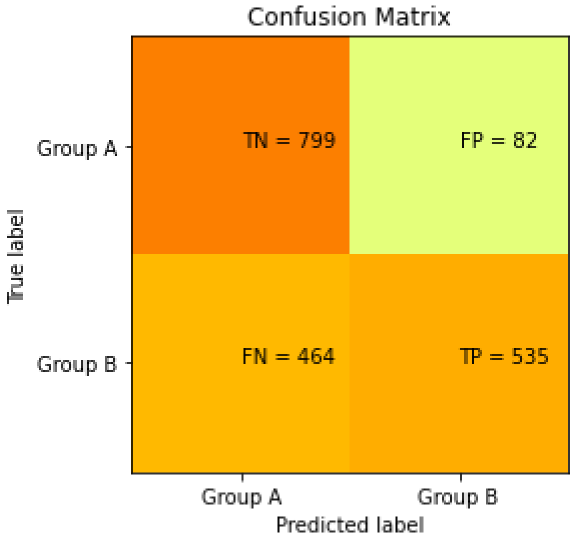

AUC Score : 0.7212297427961446

Accuracy Of the Model(KNeighborsClassifier): 0.7095744680851064

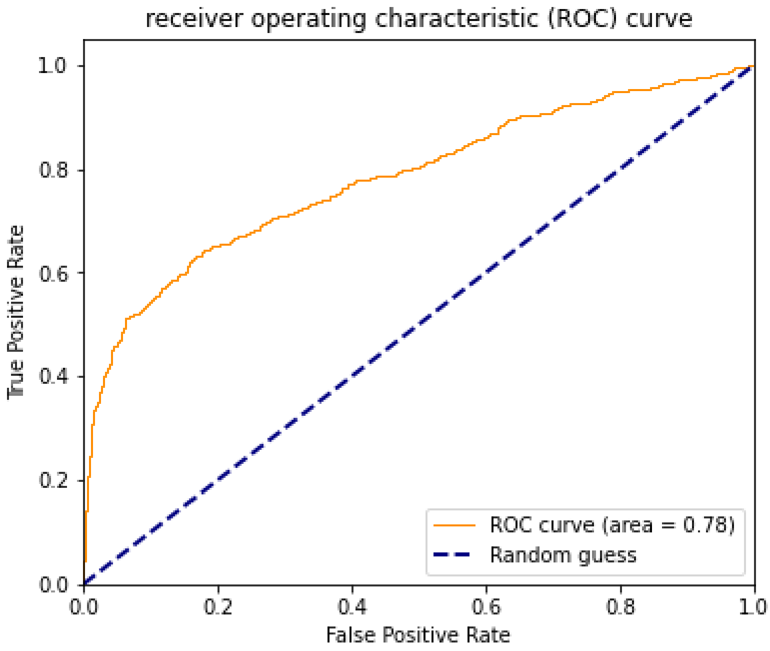

```
ROC_AUC Score of the model(KNeighborsClassifier): 0.7842973506991668


Confusion Matrix:
[[806  75]
 [428 571]]

Classification Report:
              precision    recall  f1-score   support

         0.0       0.65      0.91      0.76       881
         1.0       0.88      0.57      0.69       999

    accuracy                           0.73      1880
   macro avg       0.77      0.74      0.73      1880
weighted avg       0.78      0.73      0.73      1880
```

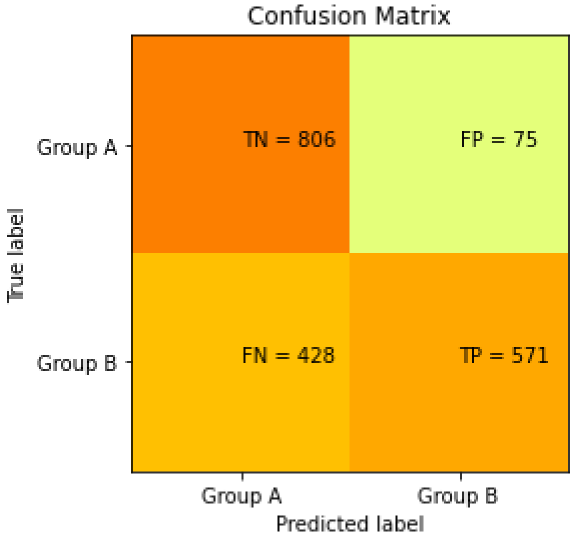


```
AUC Score : 0.7432205190434475

Accuracy Of the Model(DecisionTreeClassifier): 0.7324468085106383
```

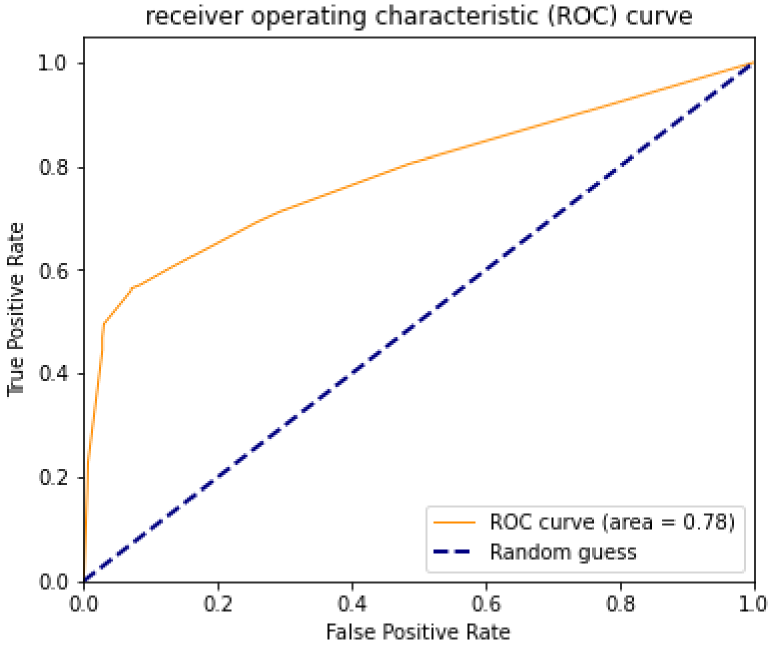

```
ROC_AUC Score of the model(DecisionTreeClassifier): 0.7825845141395652


Confusion Matrix:
[[770 111]
 [420 579]]

Classification Report:
              precision    recall  f1-score   support

         0.0       0.65      0.87      0.74       881
         1.0       0.84      0.58      0.69       999

    accuracy                           0.72      1880
   macro avg       0.74      0.73      0.71      1880
weighted avg       0.75      0.72      0.71      1880
```

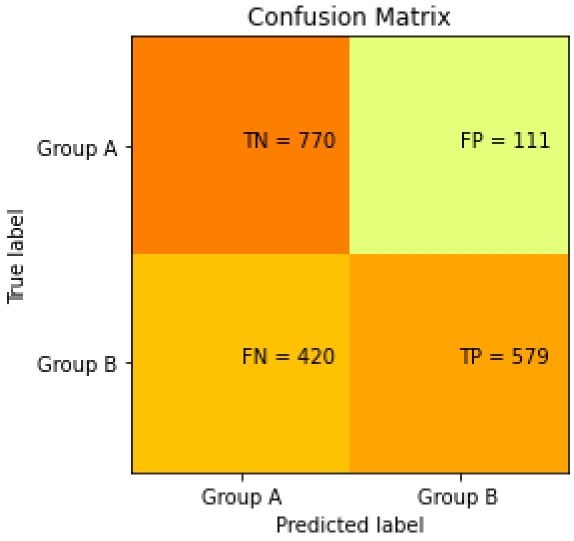


```
AUC Score : 0.7267931950111293

Accuracy Of the Model(ExtraTreesClassifier): 0.7175531914893617
```

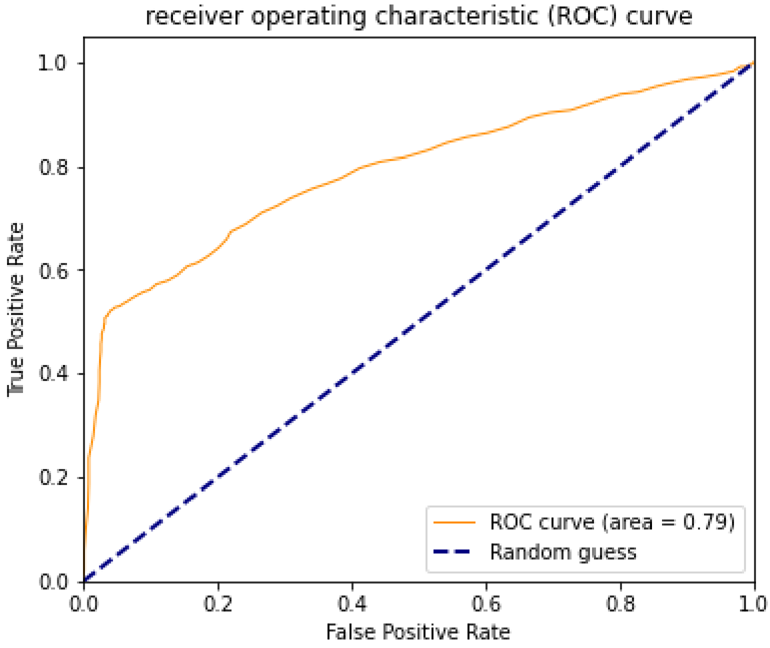

ROC_AUC Score of the model(ExtraTreesClassifier): 0.7905510504829459

Confusion Matrix:
[[801 80]
 [403 596]]

Classification Report:

	precision	recall	f1-score	support
0.0	0.67	0.91	0.77	881
1.0	0.88	0.60	0.71	999
accuracy			0.74	1880
macro avg	0.77	0.75	0.74	1880
weighted avg	0.78	0.74	0.74	1880

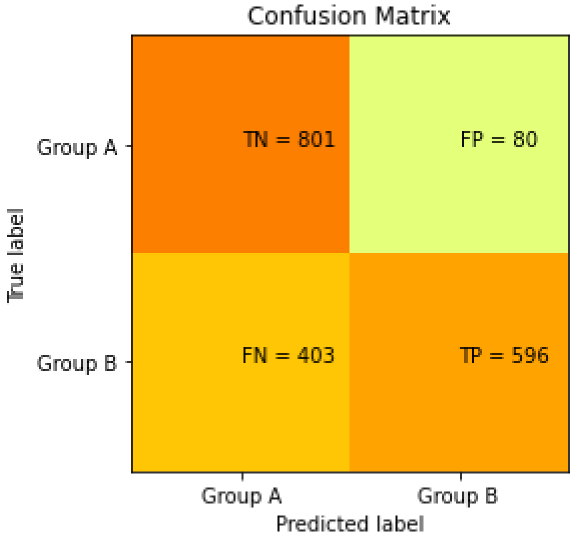

AUC Score : 0.7528953471064708

Accuracy Of the Model(GradientBoostingClassifier): 0.7430851063829788

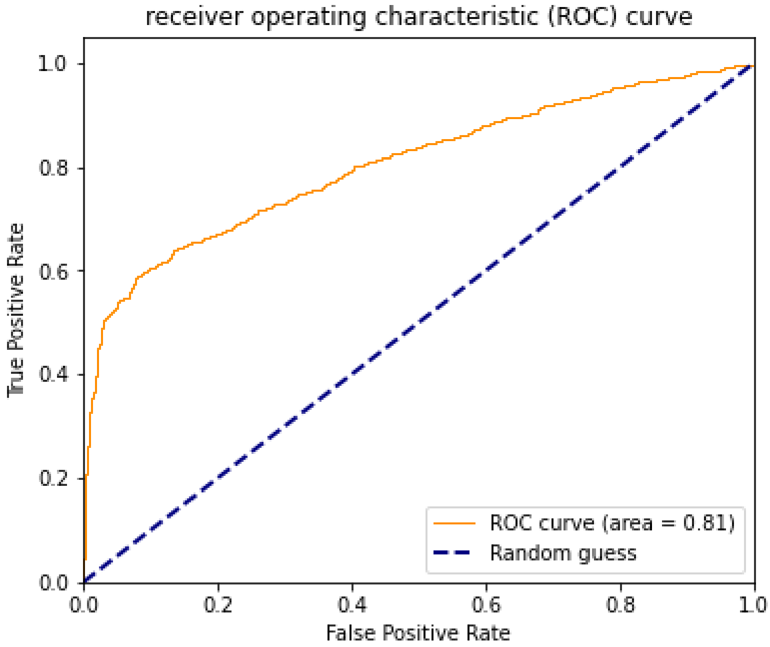

ROC_AUC Score of the model(GradientBoostingClassifier): 0.8064568541299528

Confusion Matrix:
[[823 58]
 [451 548]]

Classification Report:

	precision	recall	f1-score	support
0.0	0.65	0.93	0.76	881
1.0	0.90	0.55	0.68	999
accuracy			0.73	1880
macro avg	0.78	0.74	0.72	1880
weighted avg	0.78	0.73	0.72	1880

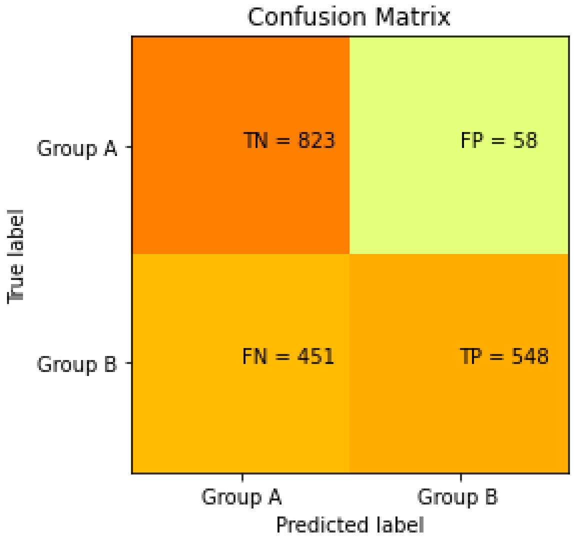

AUC Score : 0.7413571346601994

Accuracy Of the Model(AdaBoostClassifier): 0.7292553191489362

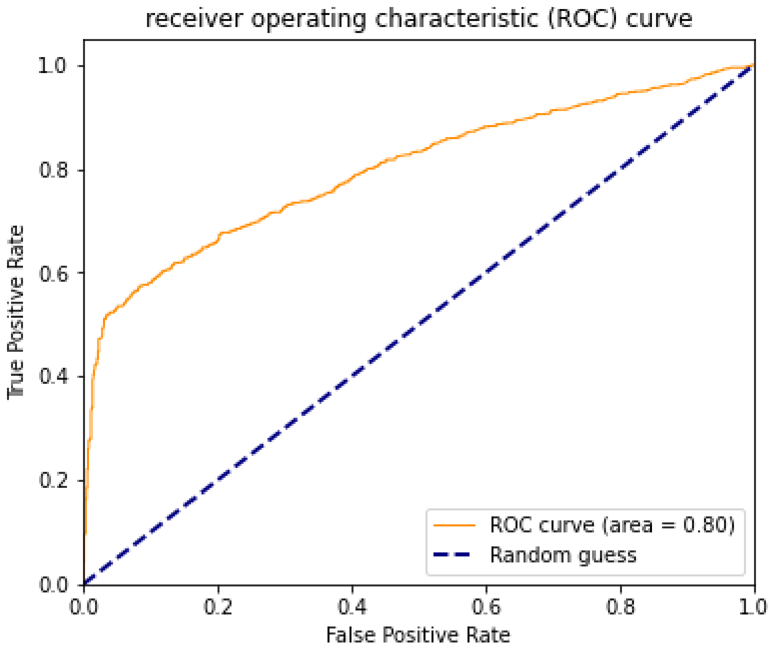

ROC_AUC Score of the model(AdaBoostClassifier): 0.7986090517305047

Confusion Matrix:
[[668 213]
 [348 651]]

Classification Report:

	precision	recall	f1-score	support
0.0	0.66	0.76	0.70	881
1.0	0.75	0.65	0.70	999
accuracy			0.70	1880
macro avg	0.71	0.70	0.70	1880
weighted avg	0.71	0.70	0.70	1880

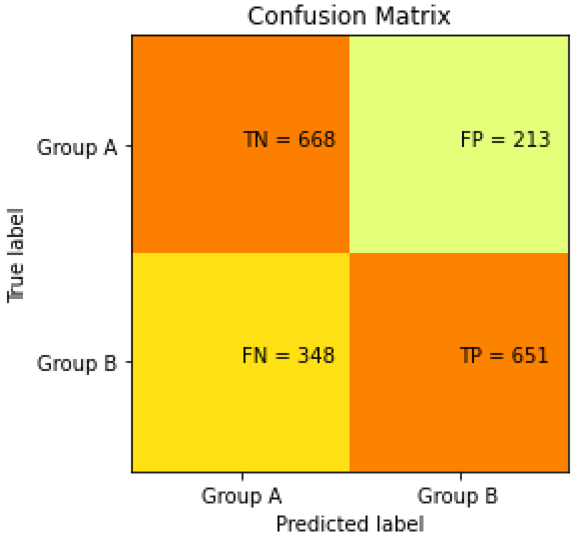

AUC Score : 0.704940468277585

Accuracy Of the Model(SVC): 0.7015957446808511

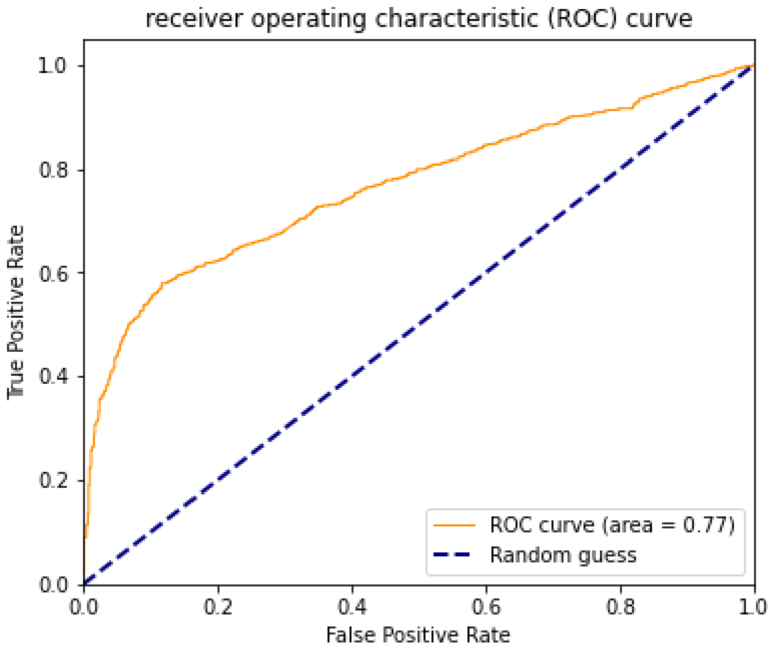

```
ROC_AUC Score of the model(SVC): 0.7656544171867667


Confusion Matrix:
[[799  82]
 [426 573]]

Classification Report:
              precision    recall  f1-score   support

         0.0       0.65      0.91      0.76       881
         1.0       0.87      0.57      0.69       999

    accuracy                           0.73      1880
   macro avg       0.76      0.74      0.73      1880
weighted avg       0.77      0.73      0.72      1880
```

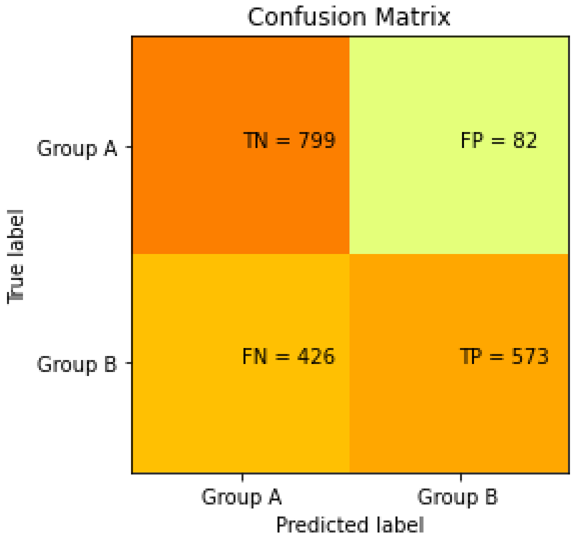


```
AUC Score : 0.7402487618151635

Accuracy Of the Model(MLPClassifier): 0.7297872340425532
```

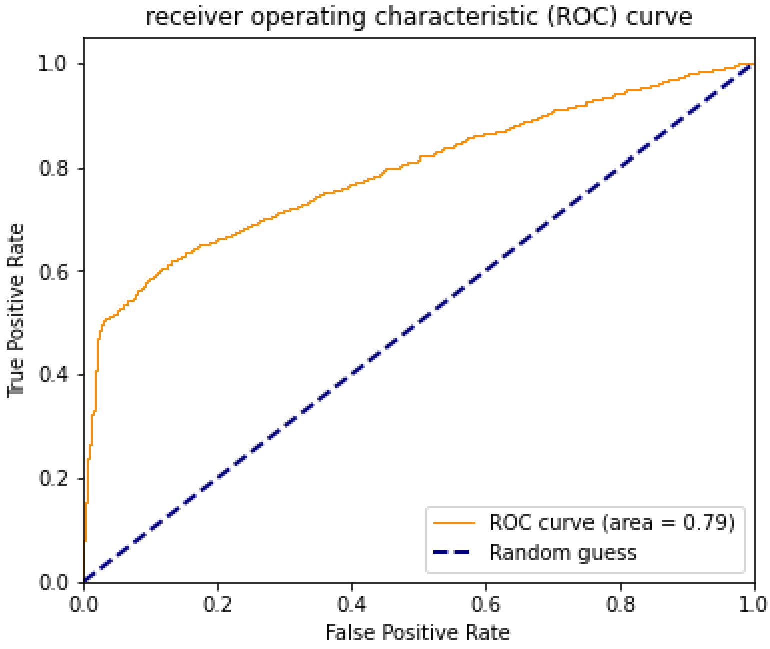

```
ROC_AUC Score of the model(MLPClassifier): 0.7924894247255201

[('XGBClassifier', 0.7436170212765958), ('GradientBoostingClassifier', 0.743085106
3829788), ('LogisticRegression', 0.7335106382978723), ('RandomForestClassifier',
0.7335106382978723), ('DecisionTreeClassifier', 0.7324468085106383), ('MLPClassifi
er', 0.7297872340425532), ('AdaBoostClassifier', 0.7292553191489362), ('ExtraTrees
Classifier', 0.7175531914893617), ('KNeighborsClassifier', 0.7095744680851064),
('SVC', 0.7015957446808511)]


sorted_total_auc:
 [('XGBClassifier', 0.8091326286558976), ('GradientBoostingClassifier', 0.80645685
41299528), ('RandomForestClassifier', 0.8021722062584717), ('AdaBoostClassifier',
0.7986090517305047), ('LogisticRegression', 0.7974012605113626), ('MLPClassifier',
0.7924894247255201), ('ExtraTreesClassifier', 0.7905510504829459), ('KNeighborsCla
ssifier', 0.7842973506991668), ('DecisionTreeClassifier', 0.7825845141395652), ('S
VC', 0.7656544171867667)]
```

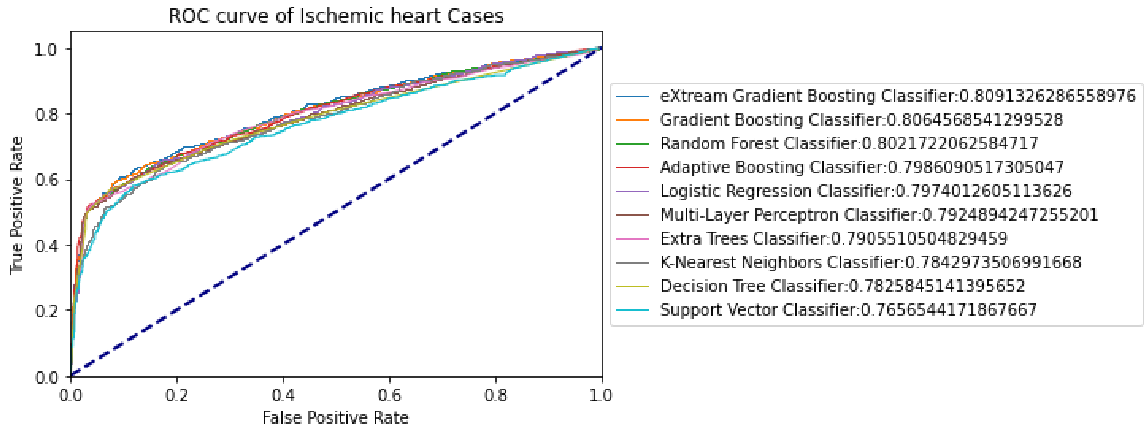

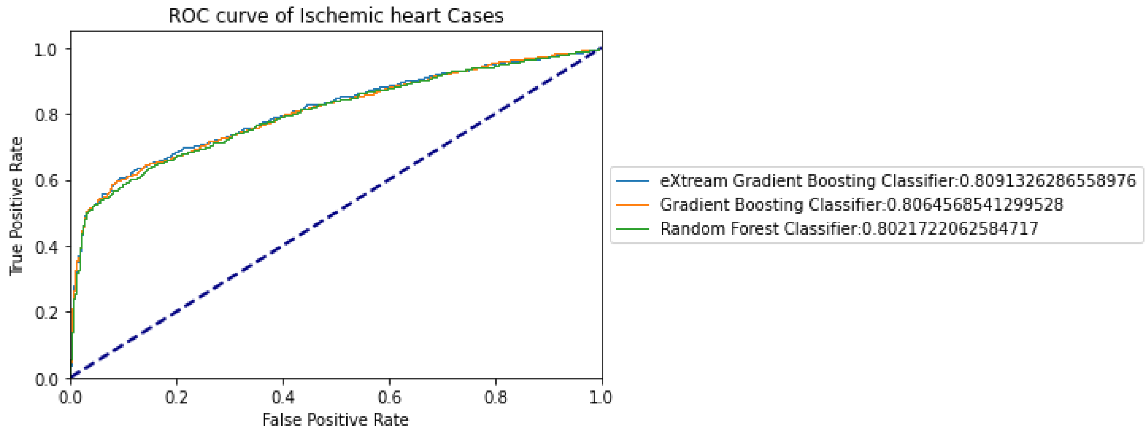

```
random state :   12598
9398
result_train_filename: data/train_test_data_ischemic heart_2013_20210519_134634.cs
v
total count : 9398, df_train count : 7518, df_test count : 1880
(7518, 38)
(1880, 38)
('Logistic Regression', LogisticRegression())
[2021-05-19 13:46:34.760398] Start parameter search for model 'Logistic Regressio
n'
[2021-05-19 13:46:35.116577] Finish parameter search for model 'Logistic Regressio
n' (time: 0 seconds)

=== train : best params ===
{'C': 0.1, 'penalty': 'l2', 'solver': 'lbfgs'}
=== train : best score : roc_auc ===
0.8029013935974797


('Random Forest', RandomForestClassifier())
[2021-05-19 13:46:35.117574] Start parameter search for model 'Random Forest'
[2021-05-19 13:46:39.598472] Finish parameter search for model 'Random Forest' (ti
me: 4 seconds)

=== train : best params ===
{'criterion': 'gini', 'max_depth': 10, 'max_features': 'auto', 'n_estimators': 70}
=== train : best score : roc_auc ===
0.8092183611489896


('Extreme Gradient Boosting)', XGBClassifier(base_score=None, booster=None, colsam
ple_bylevel=None,
              colsample_bynode=None, colsample_bytree=None, gamma=None,
              gpu_id=None, importance_type='gain', interaction_constraints=None,
              learning_rate=None, max_delta_step=None, max_depth=None,
              min_child_weight=None, missing=nan, monotone_constraints=None,
              n_estimators=100, n_jobs=None, num_parallel_tree=None,
              random_state=None, reg_alpha=None, reg_lambda=None,
              scale_pos_weight=None, subsample=None, tree_method=None,
              validate_parameters=None, verbosity=None))
[2021-05-19 13:46:39.598472] Start parameter search for model 'Extreme Gradient Bo
osting)'
[2021-05-19 13:47:05.808431] Finish parameter search for model 'Extreme Gradient B
oosting)' (time: 26 seconds)

=== train : best params ===
{'learning_rate': 0.1, 'max_depth': 2, 'n_estimators': 100, 'subsample': 0.7}
=== train : best score : roc_auc ===
0.8089943492680547


('k-Nearest Neighbours)', KNeighborsClassifier())
[2021-05-19 13:47:05.810426] Start parameter search for model 'k-Nearest Neighbour
s)'
[2021-05-19 13:47:13.680833] Finish parameter search for model 'k-Nearest Neighbou
rs)' (time: 7 seconds)

=== train : best params ===
{'n_neighbors': 60, 'weights': 'distance'}
=== train : best score : roc_auc ===
0.784167167472757
```

```
('Decision Tree', DecisionTreeClassifier())
[2021-05-19 13:47:13.680833] Start parameter search for model 'Decision Tree'
[2021-05-19 13:47:14.242384] Finish parameter search for model 'Decision Tree' (ti
me: 0 seconds)

=== train : best params ===
{'criterion': 'entropy', 'max_depth': 4}
=== train : best score : roc_auc ===
0.7881107381415684


('Extra Tree', ExtraTreesClassifier())
[2021-05-19 13:47:14.242384] Start parameter search for model 'Extra Tree'
[2021-05-19 13:47:17.132321] Finish parameter search for model 'Extra Tree' (time:
2 seconds)

=== train : best params ===
{'max_features': 'log2', 'n_estimators': 100}
=== train : best score : roc_auc ===
0.803061164466104


('Gradient Boosting', GradientBoostingClassifier())
[2021-05-19 13:47:17.132321] Start parameter search for model 'Gradient Boosting'
[2021-05-19 13:47:31.828012] Finish parameter search for model 'Gradient Boosting'
(time: 14 seconds)

=== train : best params ===
{'learning_rate': 0.075, 'max_depth': 5, 'max_features': 6, 'n_estimators': 50}
=== train : best score : roc_auc ===
0.8098086741347161


('Adaptive Boosting', AdaBoostClassifier())
[2021-05-19 13:47:31.829011] Start parameter search for model 'Adaptive Boosting'
[2021-05-19 13:47:35.412266] Finish parameter search for model 'Adaptive Boosting'
(time: 3 seconds)

=== train : best params ===
{'learning_rate': 0.2, 'n_estimators': 100}
=== train : best score : roc_auc ===
0.8035170308563717


('Suppport Vector Classfier', SVC(probability=True))
[2021-05-19 13:47:35.413260] Start parameter search for model 'Suppport Vector Cla
ssfier'
[2021-05-19 13:51:27.440735] Finish parameter search for model 'Suppport Vector Cl
assfier' (time: 232 seconds)

=== train : best params ===
{'C': 1, 'gamma': 0.1, 'kernel': 'rbf'}
=== train : best score : roc_auc ===
0.780239122948789


('Neural Network', MLPClassifier())
[2021-05-19 13:51:27.441683] Start parameter search for model 'Neural Network'
[2021-05-19 13:54:16.401555] Finish parameter search for model 'Neural Network' (t
```

```
ime: 168 seconds)

=== train : best params ===
{'activation': 'logistic', 'alpha': 0.05, 'hidden_layer_sizes': (50,), 'learning_r
ate': 'adaptive'}
=== train : best score : roc_auc ===
0.8060735975518541



Confusion Matrix:
[[814  67]
 [429 570]]

Classification Report:
              precision    recall  f1-score   support

         0.0       0.65      0.92      0.77       881
         1.0       0.89      0.57      0.70       999

    accuracy                           0.74      1880
   macro avg       0.77      0.75      0.73      1880
weighted avg       0.78      0.74      0.73      1880
```

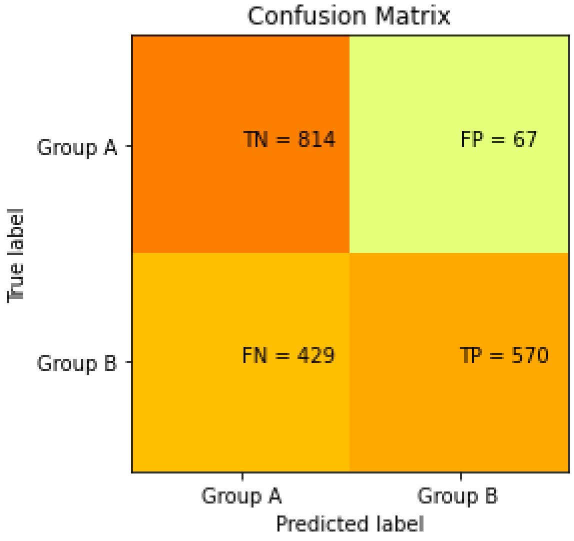


```
AUC Score : 0.7472603136621299

Accuracy Of the Model(LogisticRegression): 0.7361702127659574
```

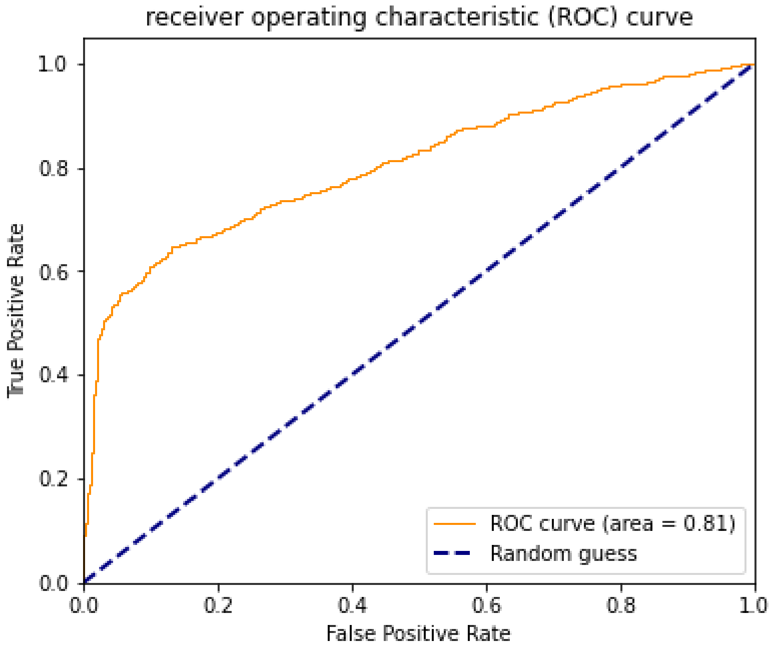


```
ROC_AUC Score of the model(LogisticRegression): 0.8064704886498304


Confusion Matrix:
[[804  77]
 [401 598]]

Classification Report:
              precision    recall  f1-score   support

         0.0       0.67      0.91      0.77       881
         1.0       0.89      0.60      0.71       999

    accuracy                           0.75      1880
   macro avg       0.78      0.76      0.74      1880
weighted avg       0.78      0.75      0.74      1880
```

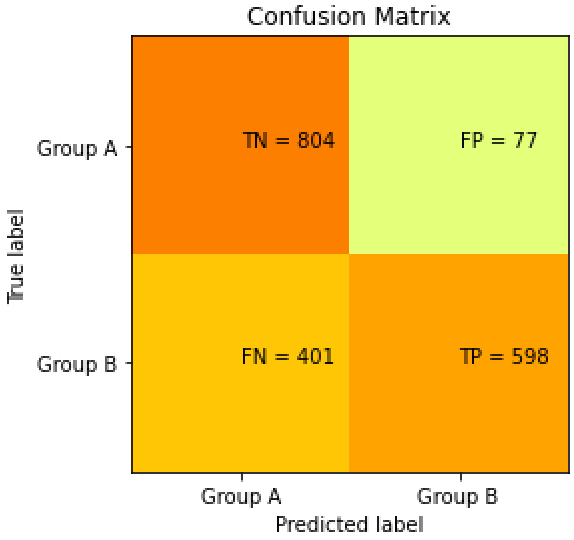


```
AUC Score : 0.7555989587771654

Accuracy Of the Model(RandomForestClassifier): 0.7457446808510638
```

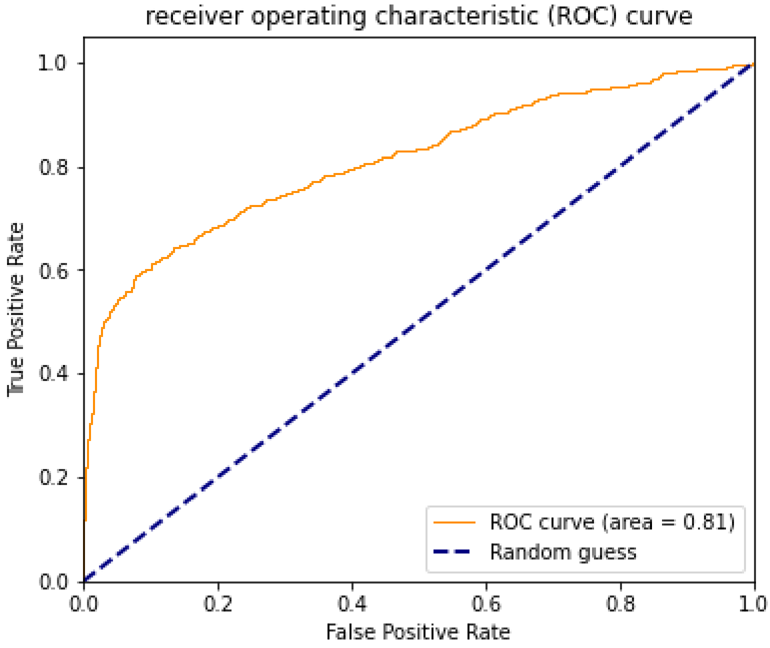

```
ROC_AUC Score of the model(RandomForestClassifier): 0.8137365515345084


Confusion Matrix:
[[810  71]
 [408 591]]

Classification Report:
              precision    recall  f1-score   support

         0.0       0.67      0.92      0.77       881
         1.0       0.89      0.59      0.71       999

    accuracy                           0.75      1880
   macro avg       0.78      0.76      0.74      1880
weighted avg       0.79      0.75      0.74      1880
```

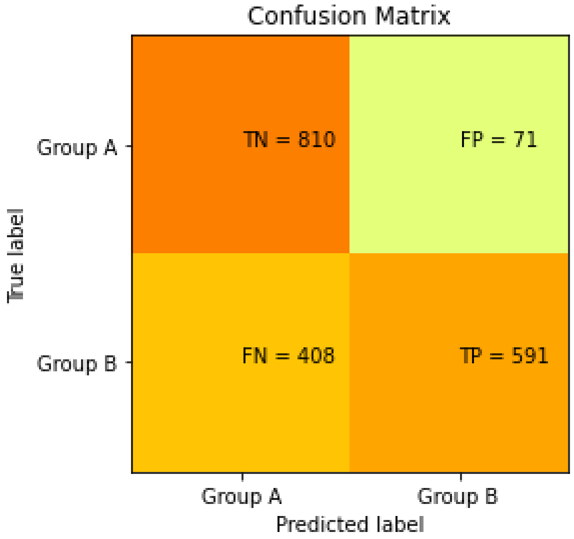


```
AUC Score : 0.7555006766113049

Accuracy Of the Model(XGBClassifier): 0.7452127659574468
```

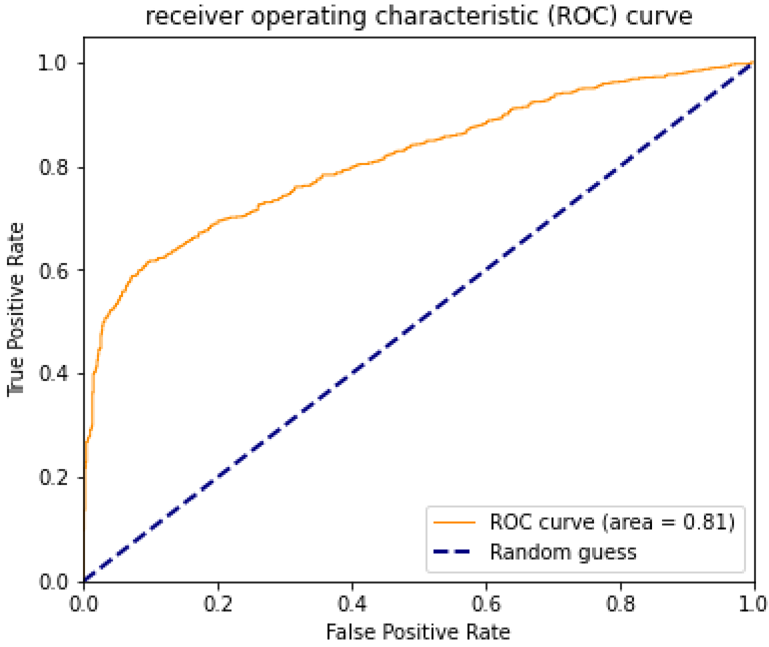

```
ROC_AUC Score of the model(XGBClassifier): 0.8138780096782368


Confusion Matrix:
[[809  72]
 [461 538]]

Classification Report:
              precision    recall  f1-score   support

         0.0       0.64      0.92      0.75       881
         1.0       0.88      0.54      0.67       999

    accuracy                           0.72      1880
   macro avg       0.76      0.73      0.71      1880
weighted avg       0.77      0.72      0.71      1880
```

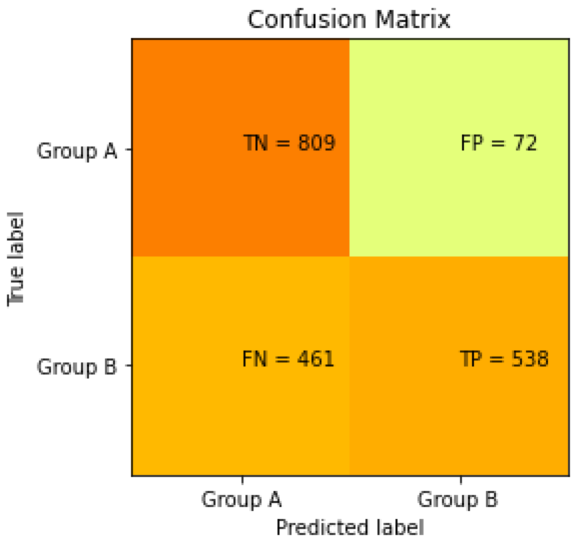


```
AUC Score : 0.7284066131966246

Accuracy Of the Model(KNeighborsClassifier): 0.7164893617021276
```

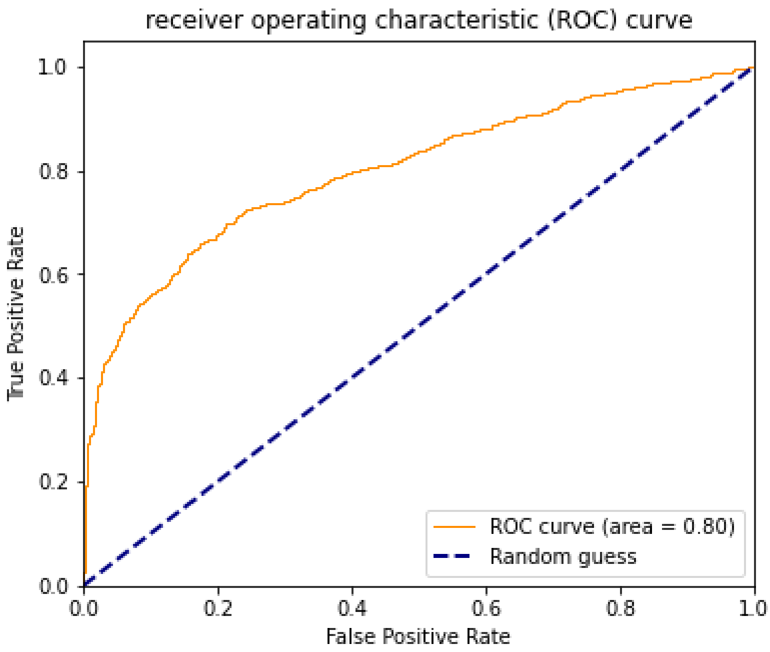

```
ROC_AUC Score of the model(KNeighborsClassifier): 0.8016961342727517


Confusion Matrix:
[[769 112]
 [392 607]]

Classification Report:
              precision    recall  f1-score   support

         0.0       0.66      0.87      0.75       881
         1.0       0.84      0.61      0.71       999

    accuracy                           0.73      1880
   macro avg       0.75      0.74      0.73      1880
weighted avg       0.76      0.73      0.73      1880
```

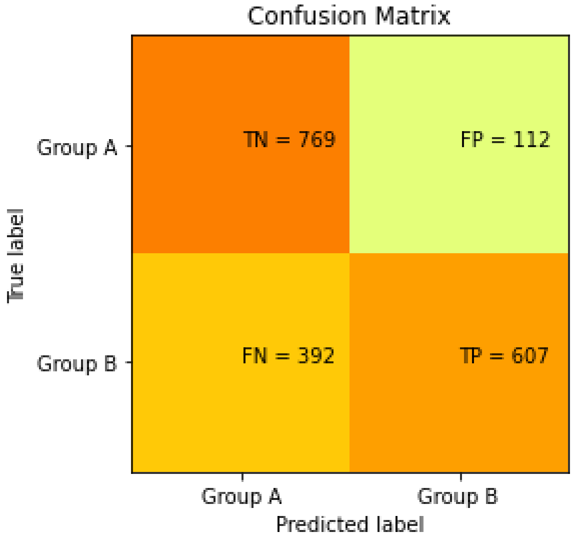


```
AUC Score : 0.7402396721352453

Accuracy Of the Model(DecisionTreeClassifier): 0.7319148936170212
```

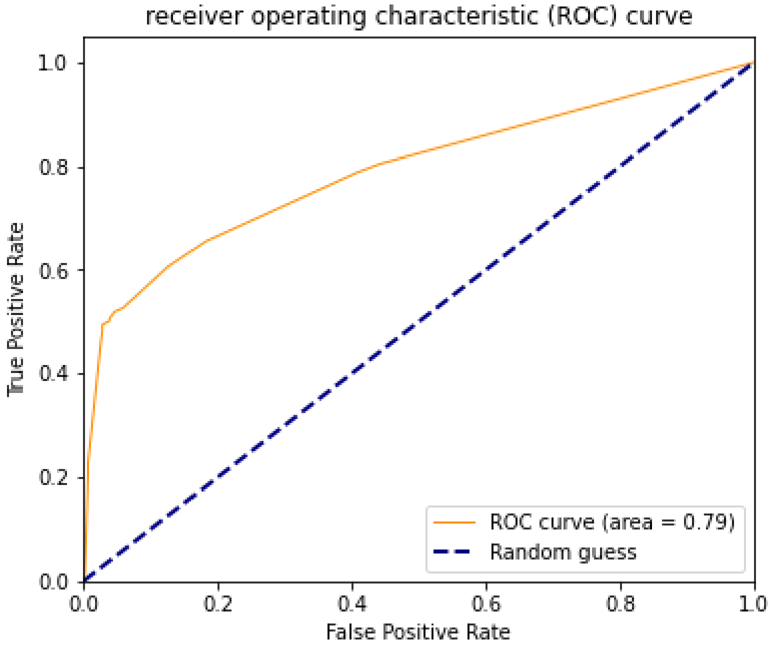

ROC_AUC Score of the model(DecisionTreeClassifier): 0.7908152193055713

Confusion Matrix:
[[809 72]
 [414 585]]

Classification Report:

	precision	recall	f1-score	support
0.0	0.66	0.92	0.77	881
1.0	0.89	0.59	0.71	999
accuracy			0.74	1880
macro avg	0.78	0.75	0.74	1880
weighted avg	0.78	0.74	0.74	1880

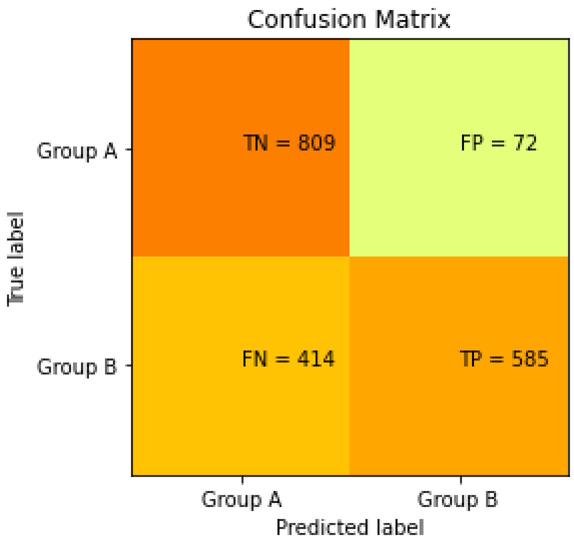

AUC Score : 0.7519301367201481

Accuracy Of the Model(ExtraTreesClassifier): 0.7414893617021276

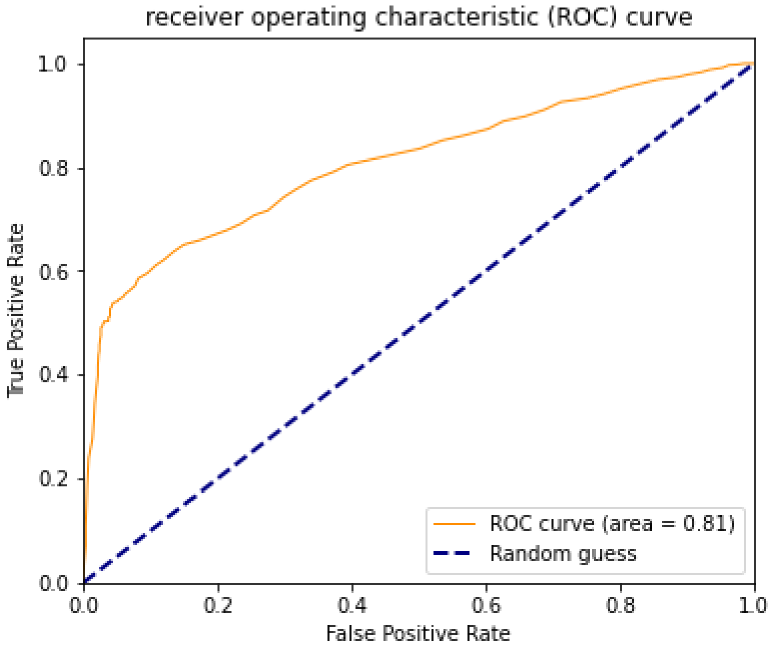

```
ROC_AUC Score of the model(ExtraTreesClassifier): 0.8070704075244369


Confusion Matrix:
[[799  82]
 [412 587]]

Classification Report:
              precision    recall  f1-score   support

         0.0       0.66      0.91      0.76       881
         1.0       0.88      0.59      0.70       999

    accuracy                           0.74      1880
   macro avg       0.77      0.75      0.73      1880
weighted avg       0.78      0.74      0.73      1880
```

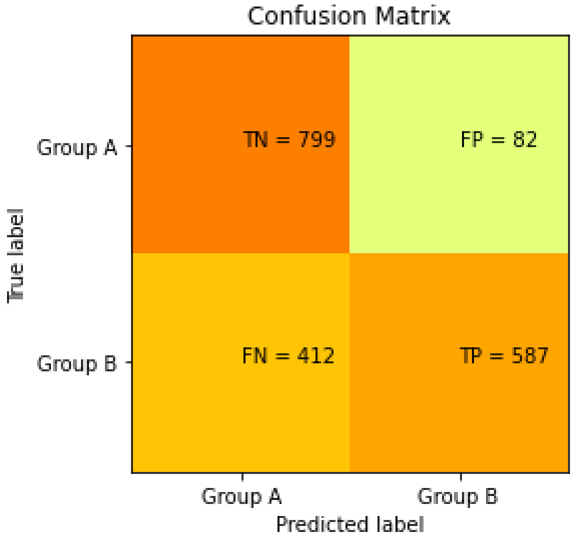


```
AUC Score : 0.74725576882217O5

Accuracy Of the Model(GradientBoostingClassifier): 0.7372340425531915
```

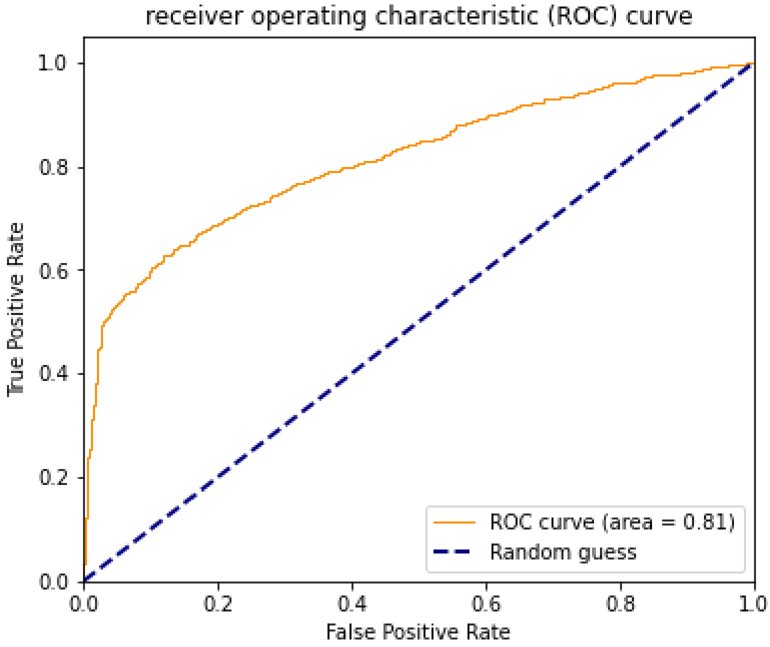

ROC_AUC Score of the model(GradientBoostingClassifier): 0.8145943900767965

Confusion Matrix:
[[816 65]
 [424 575]]

Classification Report:

	precision	recall	f1-score	support
0.0	0.66	0.93	0.77	881
1.0	0.90	0.58	0.70	999
accuracy			0.74	1880
macro avg	0.78	0.75	0.74	1880
weighted avg	0.79	0.74	0.73	1880

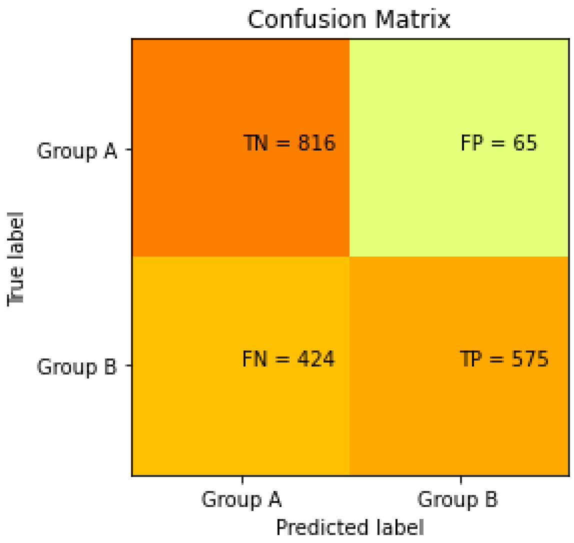

AUC Score : 0.7508978899444428

Accuracy Of the Model(AdaBoostClassifier): 0.7398936170212767

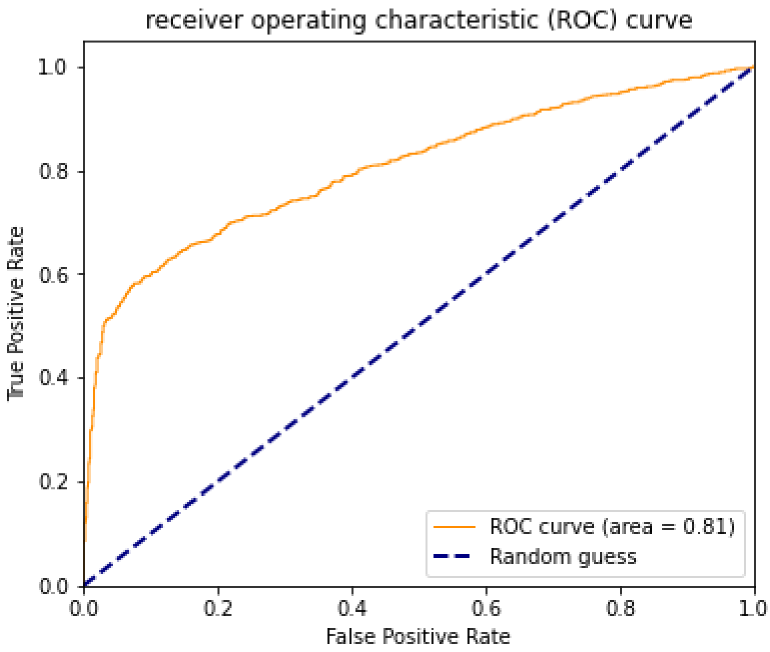

```
ROC_AUC Score of the model(AdaBoostClassifier): 0.8058609119903104


Confusion Matrix:
[[721 160]
 [345 654]]

Classification Report:
              precision    recall  f1-score   support

         0.0       0.68      0.82      0.74       881
         1.0       0.80      0.65      0.72       999

    accuracy                           0.73      1880
   macro avg       0.74      0.74      0.73      1880
weighted avg       0.74      0.73      0.73      1880
```

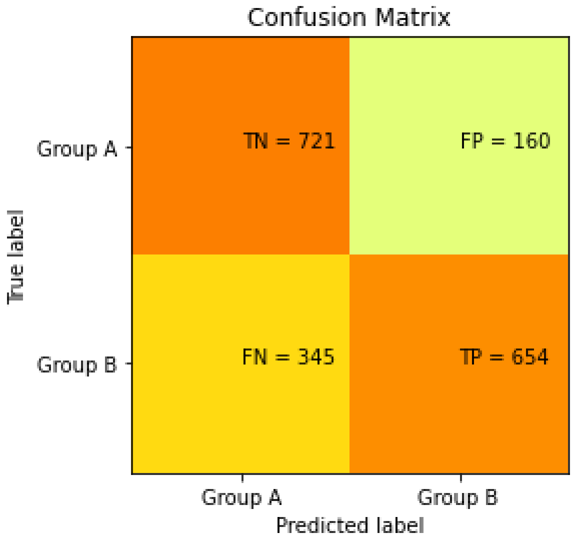


```
AUC Score : 0.7365214249436725

Accuracy Of the Model(SVC): 0.7313829787234043
```

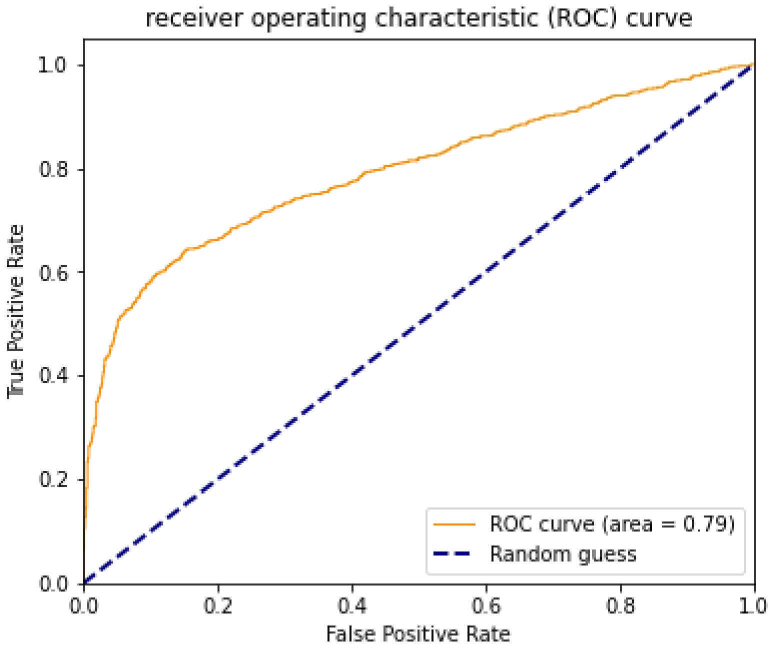

```
ROC_AUC Score of the model(SVC): 0.7912430023667255


Confusion Matrix:
[[808  73]
 [403 596]]

Classification Report:
              precision    recall  f1-score   support

         0.0       0.67      0.92      0.77       881
         1.0       0.89      0.60      0.71       999

    accuracy                           0.75      1880
   macro avg       0.78      0.76      0.74      1880
weighted avg       0.79      0.75      0.74      1880
```

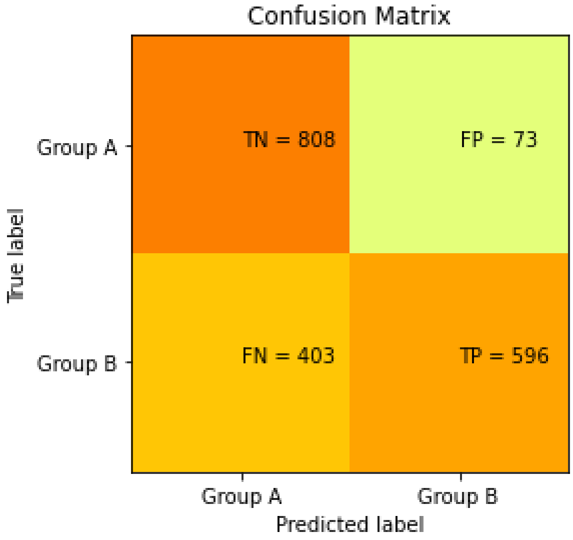


```
AUC Score : 0.7568681053357557

Accuracy Of the Model(MLPClassifier): 0.7468085106382979
```

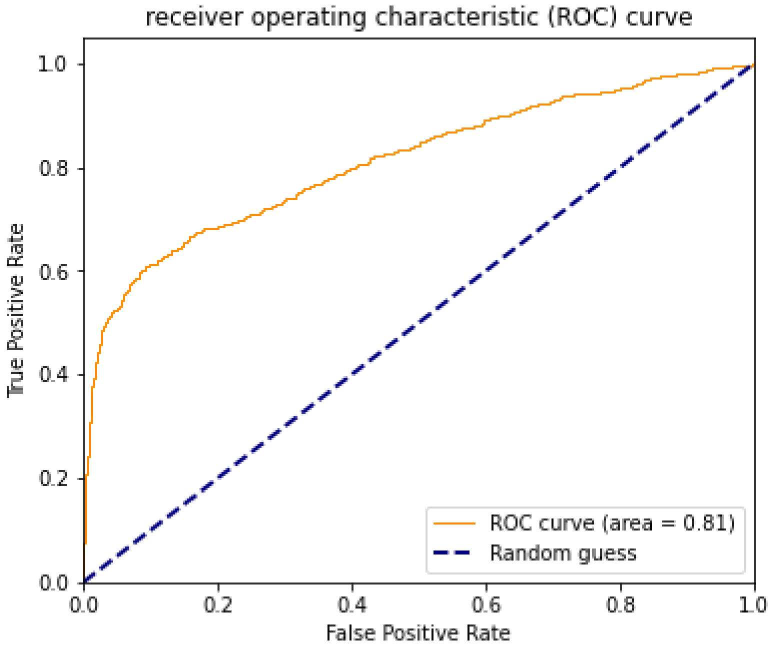

```
ROC_AUC Score of the model(MLPClassifier): 0.8127355505335074

[('MLPClassifier', 0.7468085106382979), ('RandomForestClassifier', 0.7457446808510638), ('XGBClassifier', 0.7452127659574468), ('ExtraTreesClassifier', 0.7414893617021276), ('AdaBoostClassifier', 0.7398936170212767), ('GradientBoostingClassifier', 0.7372340425531915), ('LogisticRegression', 0.7361702127659574), ('DecisionTreeClassifier', 0.7319148936170212), ('SVC', 0.7313829787234043), ('KNeighborsClassifier', 0.7164893617021276)]


sorted_total_auc:
 [('GradientBoostingClassifier', 0.8145943900767965), ('XGBClassifier', 0.8138780096782368), ('RandomForestClassifier', 0.8137365515345084), ('MLPClassifier', 0.8127355505335074), ('ExtraTreesClassifier', 0.8070704075244369), ('LogisticRegression', 0.8064704886498304), ('AdaBoostClassifier', 0.8058609119903104), ('KNeighborsClassifier', 0.8016961342727517), ('SVC', 0.7912430023667255), ('DecisionTreeClassifier', 0.7908152193055713)]
```

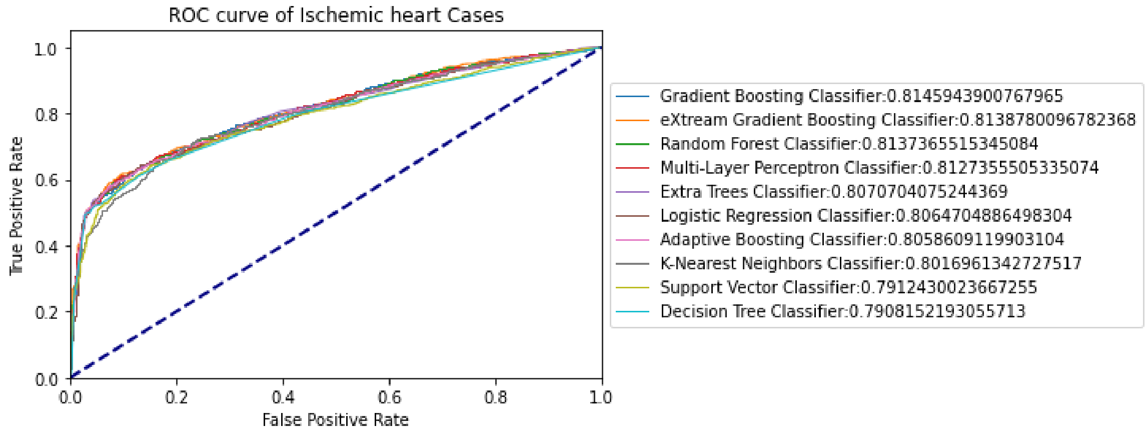

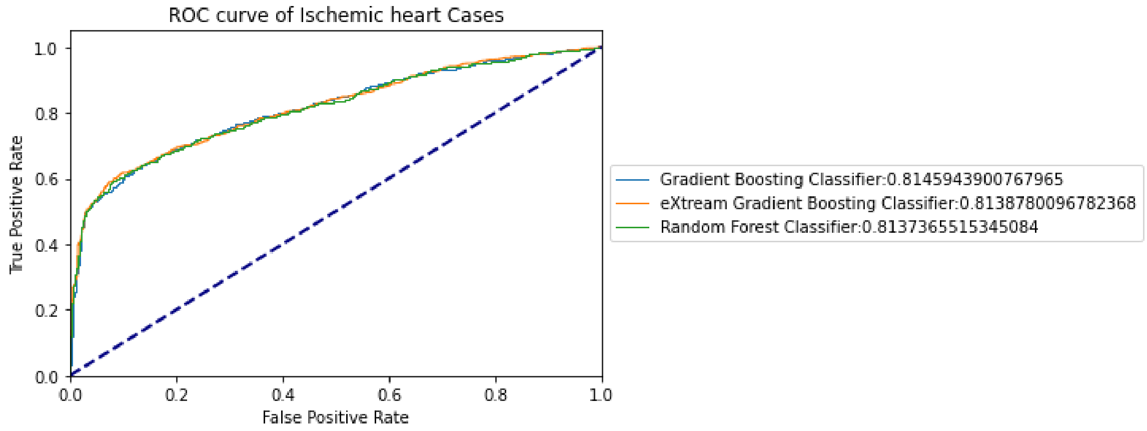

```
random state :   12621
9398
result_train_filename: data/train_test_data_ischemic heart_2013_20210519_135423.cs
v
total count : 9398, df_train count : 7518, df_test count : 1880
(7518, 38)
(1880, 38)
('Logistic Regression', LogisticRegression())
[2021-05-19 13:54:24.243180] Start parameter search for model 'Logistic Regressio
n'
[2021-05-19 13:54:24.576809] Finish parameter search for model 'Logistic Regressio
n' (time: 0 seconds)

=== train : best params ===
{'C': 1000.0, 'penalty': 'l2', 'solver': 'lbfgs'}
=== train : best score : roc_auc ===
0.8046944721079841



('Random Forest', RandomForestClassifier())
[2021-05-19 13:54:24.576809] Start parameter search for model 'Random Forest'
[2021-05-19 13:54:29.731684] Finish parameter search for model 'Random Forest' (ti
me: 5 seconds)

=== train : best params ===
{'criterion': 'gini', 'max_depth': 10, 'max_features': 'auto', 'n_estimators': 10
0}
=== train : best score : roc_auc ===
0.8102336093101323



('Extreme Gradient Boosting)', XGBClassifier(base_score=None, booster=None, colsam
ple_bylevel=None,
              colsample_bynode=None, colsample_bytree=None, gamma=None,
              gpu_id=None, importance_type='gain', interaction_constraints=None,
              learning_rate=None, max_delta_step=None, max_depth=None,
              min_child_weight=None, missing=nan, monotone_constraints=None,
              n_estimators=100, n_jobs=None, num_parallel_tree=None,
              random_state=None, reg_alpha=None, reg_lambda=None,
              scale_pos_weight=None, subsample=None, tree_method=None,
              validate_parameters=None, verbosity=None))
[2021-05-19 13:54:29.732672] Start parameter search for model 'Extreme Gradient Bo
osting)'
[2021-05-19 13:54:58.135549] Finish parameter search for model 'Extreme Gradient B
oosting)' (time: 28 seconds)

=== train : best params ===
{'learning_rate': 0.05, 'max_depth': 5, 'n_estimators': 40, 'subsample': 0.8}
=== train : best score : roc_auc ===
0.8085825545966199



('k-Nearest Neighbours)', KNeighborsClassifier())
[2021-05-19 13:54:58.137543] Start parameter search for model 'k-Nearest Neighbour
s)'
[2021-05-19 13:55:06.113821] Finish parameter search for model 'k-Nearest Neighbou
rs)' (time: 7 seconds)

=== train : best params ===
{'n_neighbors': 60, 'weights': 'distance'}
=== train : best score : roc_auc ===
```

```
0.7870776808191899


('Decision Tree', DecisionTreeClassifier())
[2021-05-19 13:55:06.113821] Start parameter search for model 'Decision Tree'
[2021-05-19 13:55:06.715665] Finish parameter search for model 'Decision Tree' (ti
me: 0 seconds)

=== train : best params ===
{'criterion': 'entropy', 'max_depth': 4}
=== train : best score : roc_auc ===
0.7913782880979873


('Extra Tree', ExtraTreesClassifier())
[2021-05-19 13:55:06.716660] Start parameter search for model 'Extra Tree'
[2021-05-19 13:55:09.763977] Finish parameter search for model 'Extra Tree' (time:
3 seconds)

=== train : best params ===
{'max_features': 'auto', 'n_estimators': 100}
=== train : best score : roc_auc ===
0.8018528283852275


('Gradient Boosting', GradientBoostingClassifier())
[2021-05-19 13:55:09.763977] Start parameter search for model 'Gradient Boosting'
[2021-05-19 13:55:25.783604] Finish parameter search for model 'Gradient Boosting'
(time: 16 seconds)

=== train : best params ===
{'learning_rate': 0.01, 'max_depth': 8, 'max_features': 6, 'n_estimators': 100}
=== train : best score : roc_auc ===
0.8109909729181783


('Adaptive Boosting', AdaBoostClassifier())
[2021-05-19 13:55:25.784595] Start parameter search for model 'Adaptive Boosting'
[2021-05-19 13:55:29.299285] Finish parameter search for model 'Adaptive Boosting'
(time: 3 seconds)

=== train : best params ===
{'learning_rate': 0.2, 'n_estimators': 100}
=== train : best score : roc_auc ===
0.8008606260961179


('Suppport Vector Classfier', SVC(probability=True))
[2021-05-19 13:55:29.299285] Start parameter search for model 'Suppport Vector Cla
ssfier'
[2021-05-19 13:59:54.376271] Finish parameter search for model 'Suppport Vector Cl
assfier' (time: 265 seconds)

=== train : best params ===
{'C': 1, 'gamma': 0.1, 'kernel': 'rbf'}
=== train : best score : roc_auc ===
0.7844445240112576


('Neural Network', MLPClassifier())
[2021-05-19 13:59:54.376271] Start parameter search for model 'Neural Network'
```

```
[2021-05-19 14:02:35.293632] Finish parameter search for model 'Neural Network' (t
ime: 160 seconds)

=== train : best params ===
{'activation': 'logistic', 'alpha': 0.05, 'hidden_layer_sizes': (50,), 'learning_r
ate': 'adaptive'}
=== train : best score : roc_auc ===
0.8097213166536619



Confusion Matrix:
[[811  70]
 [426 573]]

Classification Report:
              precision    recall  f1-score   support

         0.0       0.66      0.92      0.77       881
         1.0       0.89      0.57      0.70       999

    accuracy                           0.74      1880
   macro avg       0.77      0.75      0.73      1880
weighted avg       0.78      0.74      0.73      1880
```

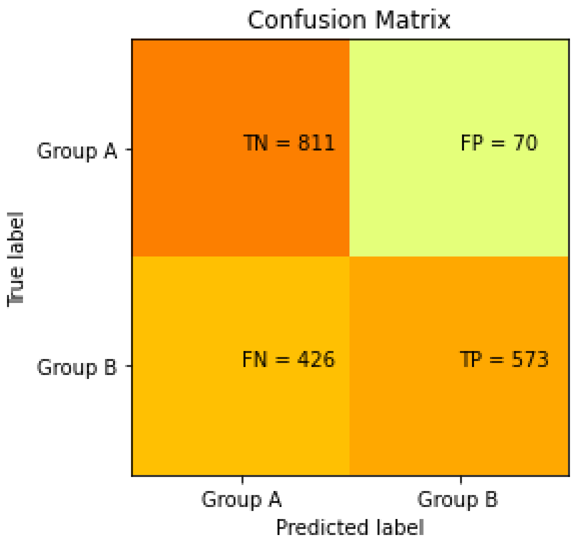


```
AUC Score : 0.7470592044939378

Accuracy Of the Model(LogisticRegression): 0.7361702127659574
```

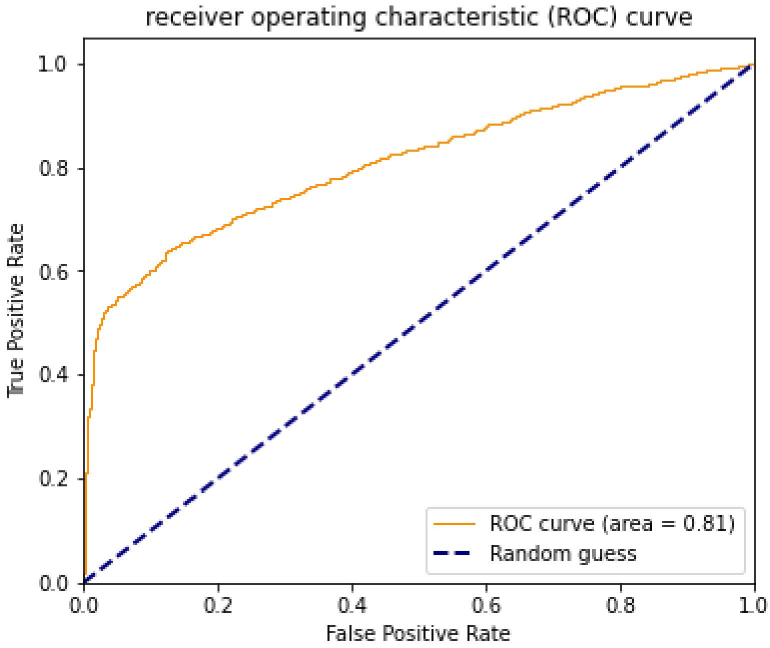

ROC_AUC Score of the model(LogisticRegression): 0.8099529722685227

Confusion Matrix:
[[799 82]
 [403 596]]

Classification Report:

	precision	recall	f1-score	support
0.0	0.66	0.91	0.77	881
1.0	0.88	0.60	0.71	999
accuracy			0.74	1880
macro avg	0.77	0.75	0.74	1880
weighted avg	0.78	0.74	0.74	1880

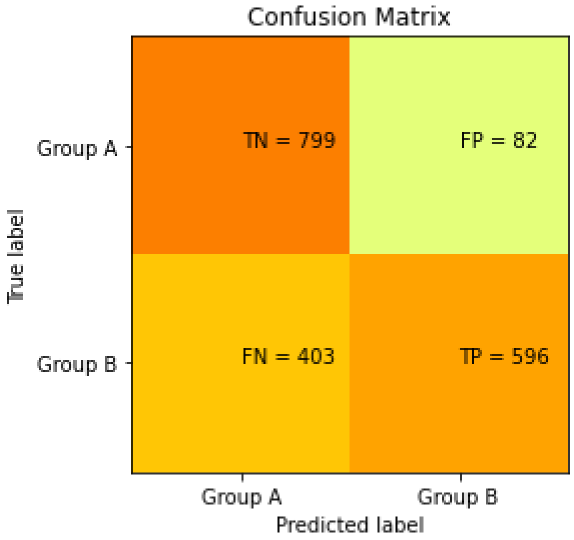


```
AUC Score : 0.7517602733266749

Accuracy Of the Model(RandomForestClassifier): 0.7420212765957447
```

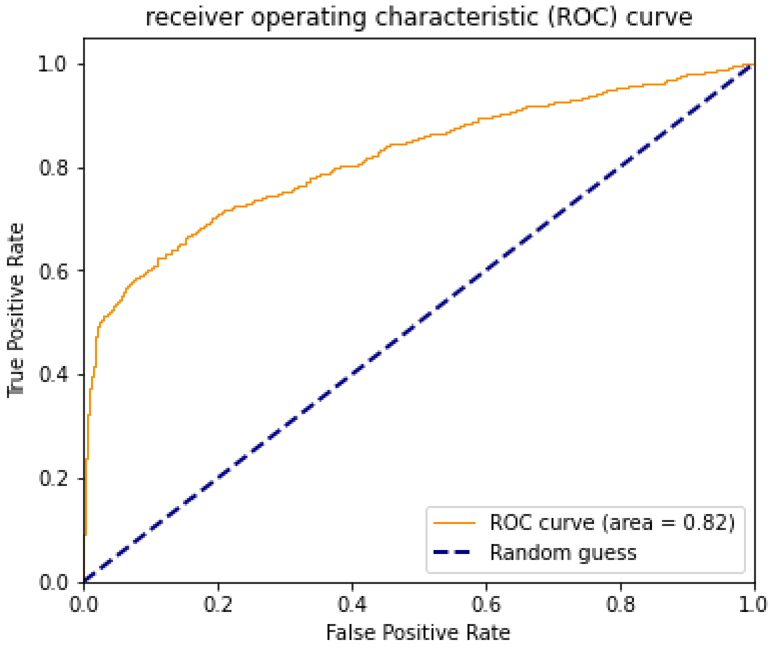

```
ROC_AUC Score of the model(RandomForestClassifier): 0.8187188323397177


Confusion Matrix:
[[816  65]
 [425 574]]

Classification Report:
              precision    recall  f1-score   support

         0.0       0.66      0.93      0.77       881
         1.0       0.90      0.57      0.70       999

    accuracy                           0.74      1880
   macro avg       0.78      0.75      0.73      1880
weighted avg       0.79      0.74      0.73      1880
```

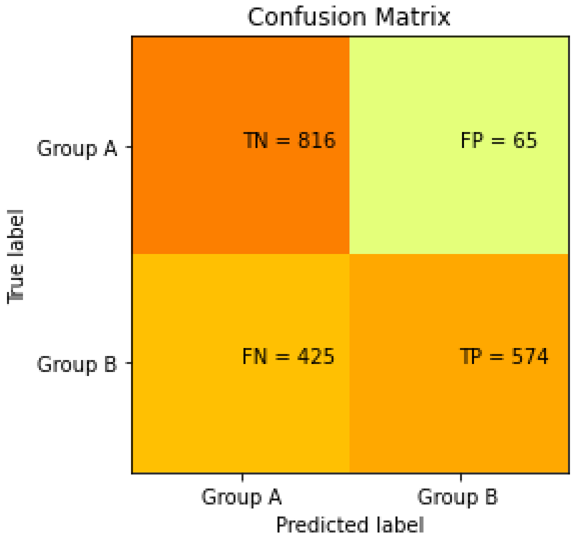


```
AUC Score : 0.7503973894439275

Accuracy Of the Model(XGBClassifier): 0.7393617021276596
```

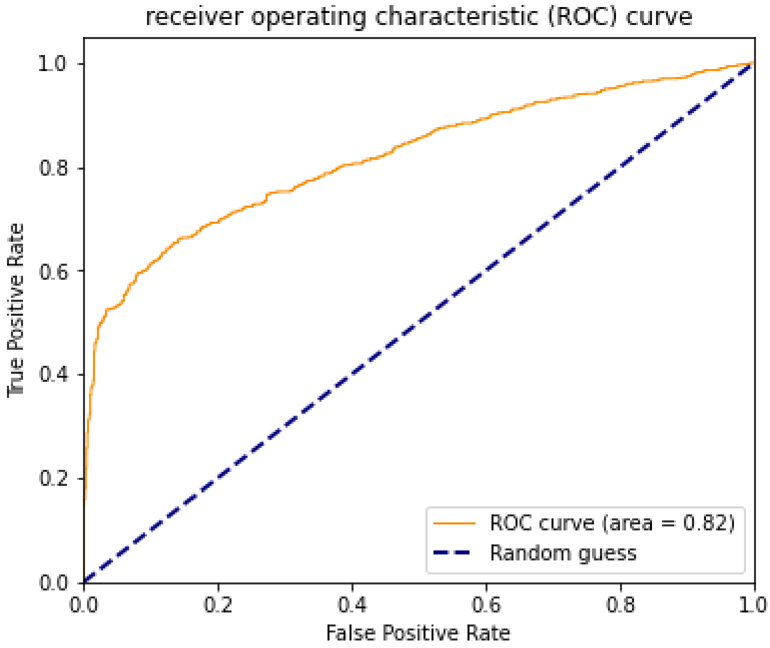

ROC_AUC Score of the model(XGBClassifier): 0.8184188729024142

Confusion Matrix:
[[810 71]
 [454 545]]

Classification Report:

	precision	recall	f1-score	support
0.0	0.64	0.92	0.76	881
1.0	0.88	0.55	0.67	999
accuracy			0.72	1880
macro avg	0.76	0.73	0.72	1880
weighted avg	0.77	0.72	0.71	1880

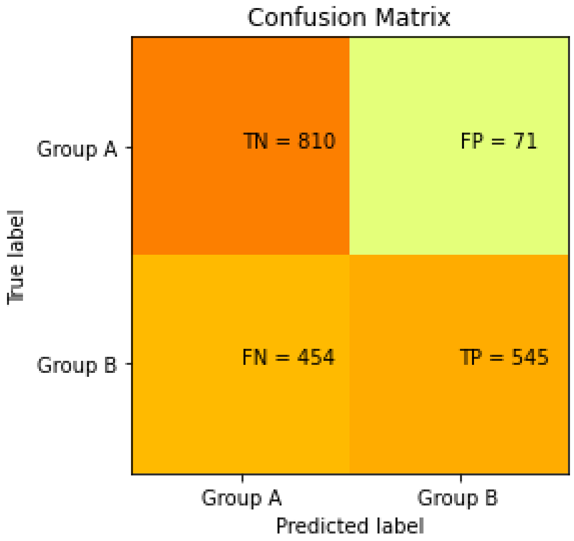

AUC Score : 0.7324776535900259

Accuracy Of the Model(KNeighborsClassifier): 0.7207446808510638

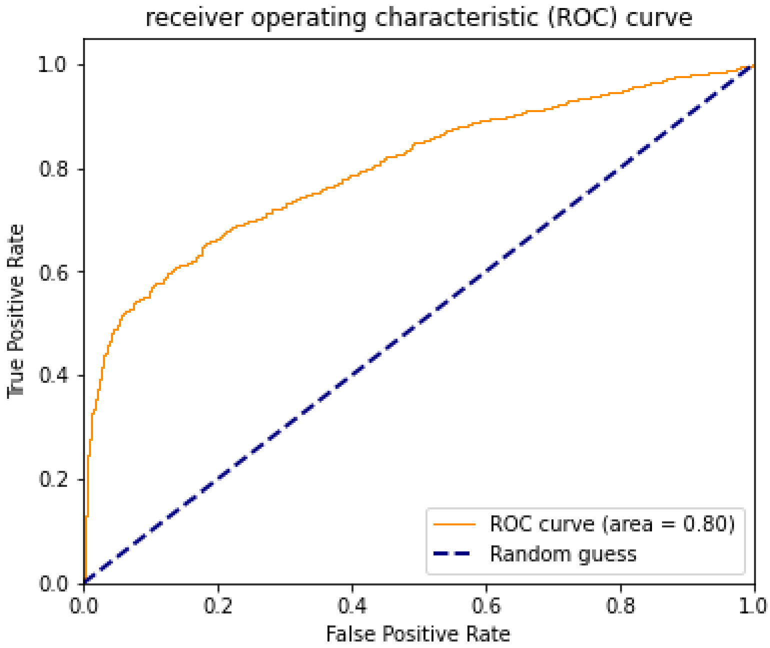

```
ROC_AUC Score of the model(KNeighborsClassifier): 0.7997645772901164


Confusion Matrix:
[[794  87]
 [409 590]]

Classification Report:
              precision    recall  f1-score   support

         0.0       0.66      0.90      0.76       881
         1.0       0.87      0.59      0.70       999

    accuracy                           0.74      1880
   macro avg       0.77      0.75      0.73      1880
weighted avg       0.77      0.74      0.73      1880
```

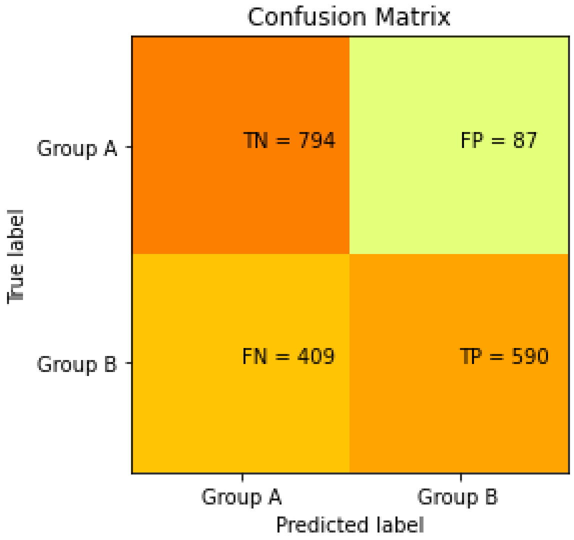


```
AUC Score : 0.7459195858741829

Accuracy Of the Model(DecisionTreeClassifier): 0.7361702127659574
```

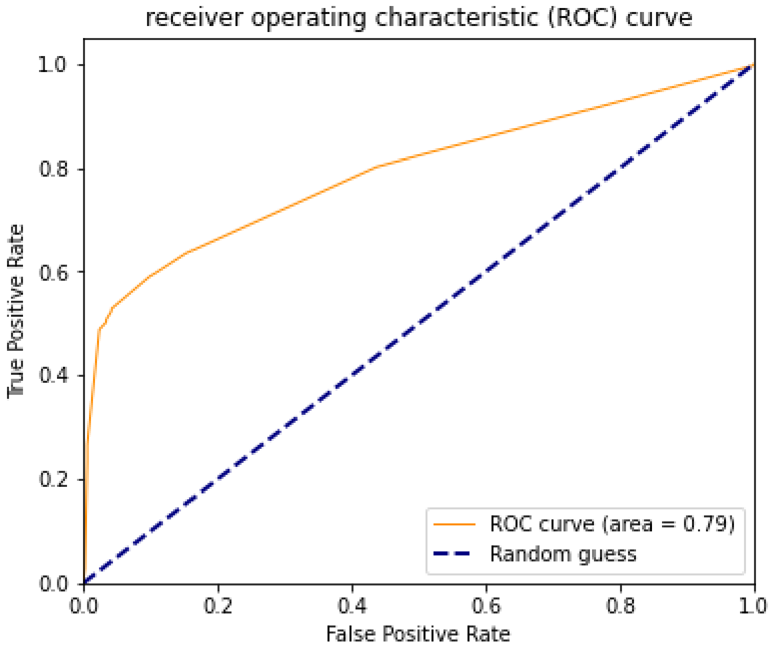

ROC_AUC Score of the model(DecisionTreeClassifier): 0.7920497114594731

```
Confusion Matrix:
[[797  84]
 [414 585]]

Classification Report:
              precision    recall  f1-score   support

         0.0       0.66      0.90      0.76       881
         1.0       0.87      0.59      0.70       999

    accuracy                           0.74      1880
   macro avg       0.77      0.75      0.73      1880
weighted avg       0.77      0.74      0.73      1880
```

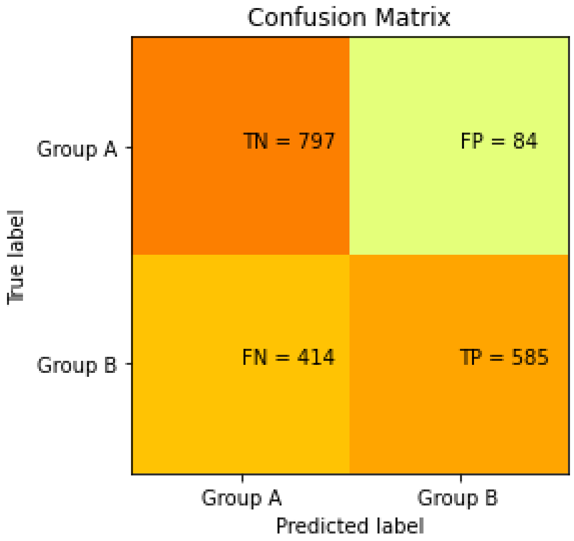

AUC Score : 0.7451196940413740

Accuracy Of the Model(ExtraTreesClassifier): 0.7351063829787234

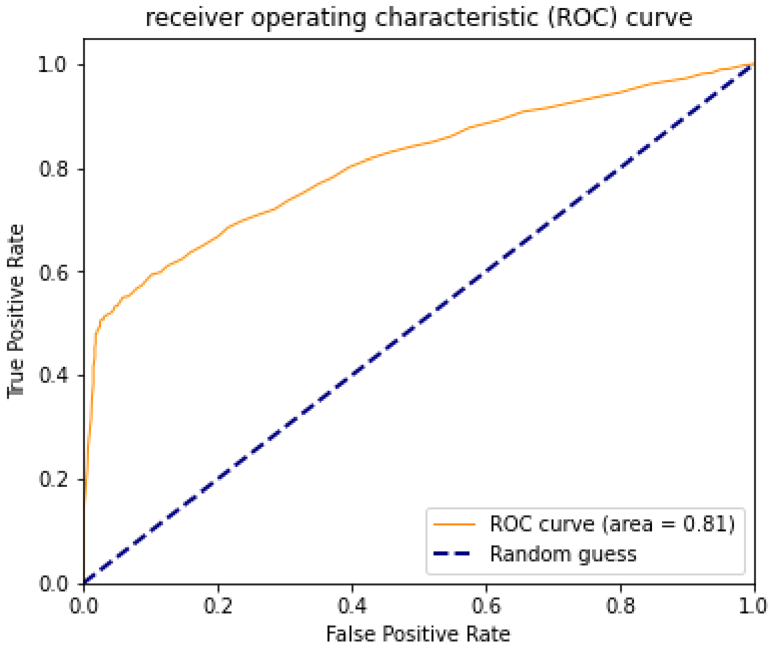

ROC_AUC Score of the model(ExtraTreesClassifier): 0.80694883305553

Confusion Matrix:
[[806 75]
 [416 583]]

Classification Report:

	precision	recall	f1-score	support
0.0	0.66	0.91	0.77	881
1.0	0.89	0.58	0.70	999
accuracy			0.74	1880
macro avg	0.77	0.75	0.74	1880
weighted avg	0.78	0.74	0.73	1880

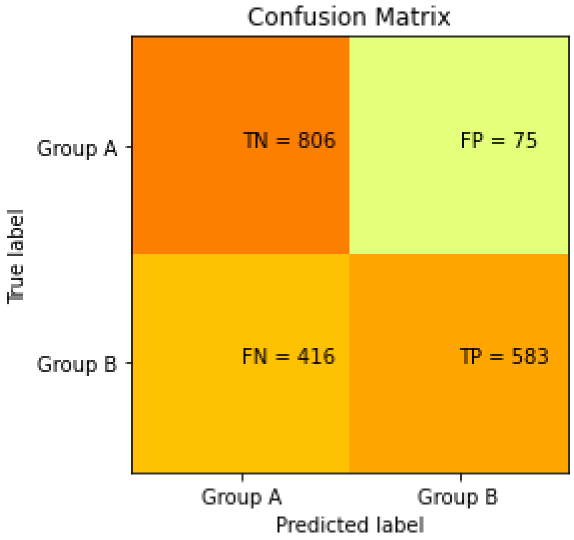

AUC Score : 0.7492265250494536

Accuracy Of the Model(GradientBoostingClassifier): 0.7388297872340426

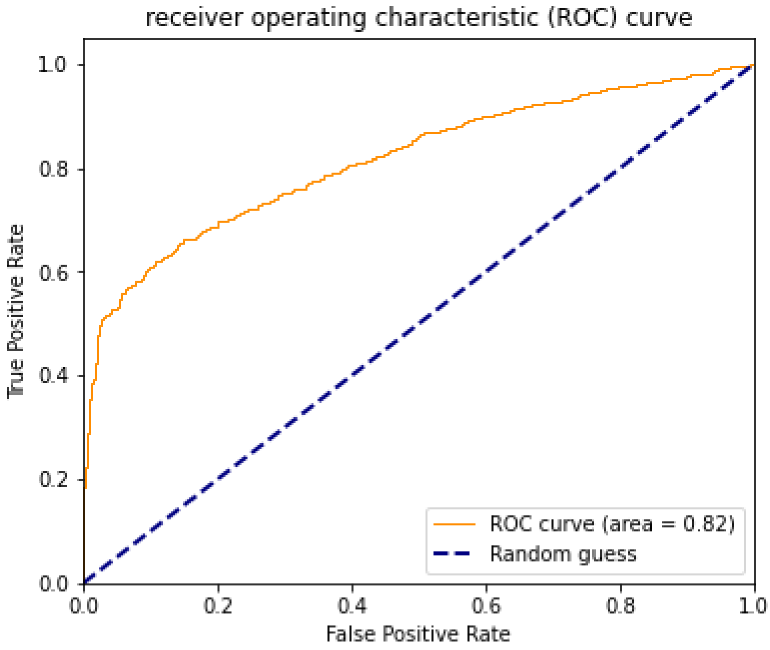

ROC_AUC Score of the model(GradientBoostingClassifier): 0.8176451138993704

```
Confusion Matrix:
[[816  65]
 [434 565]]

Classification Report:
              precision    recall  f1-score   support

         0.0       0.65      0.93      0.77       881
         1.0       0.90      0.57      0.69       999

    accuracy                           0.73      1880
   macro avg       0.77      0.75      0.73      1880
weighted avg       0.78      0.73      0.73      1880
```

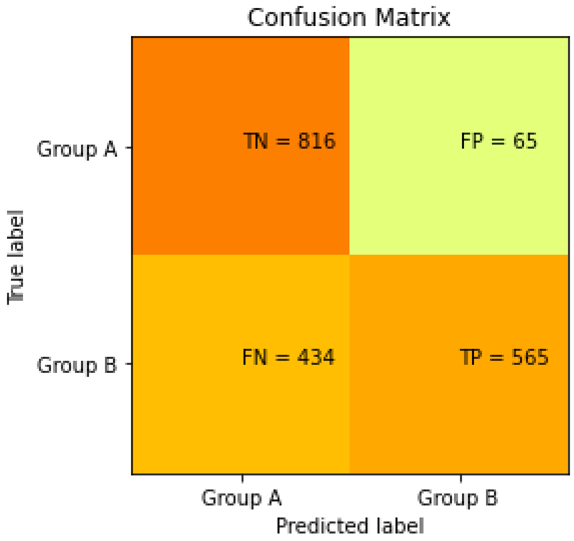

AUC Score : 0.7458928849394023

Accuracy Of the Model(AdaBoostClassifier): 0.7345744680851064

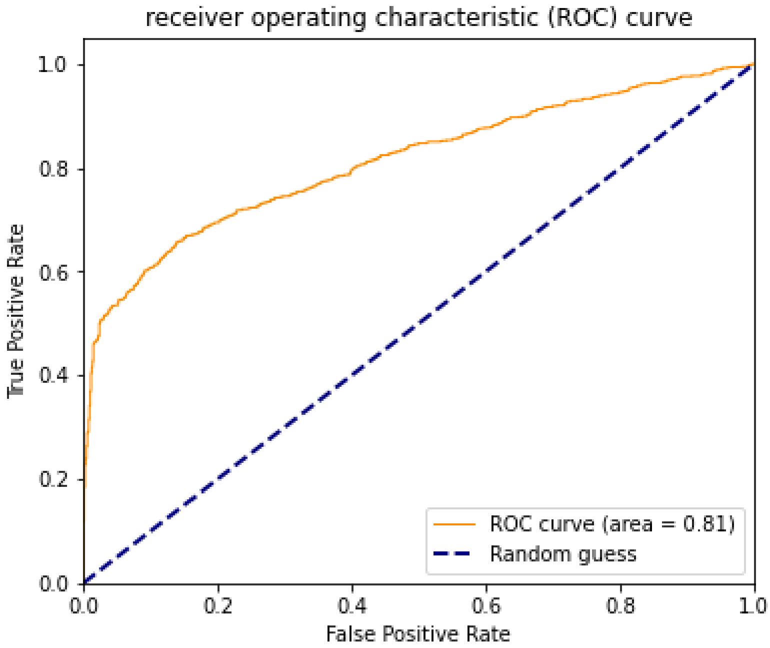

```
ROC_AUC Score of the model(AdaBoostClassifier): 0.8118822568311785


Confusion Matrix:
[[713 168]
 [346 653]]

Classification Report:
              precision    recall  f1-score   support

         0.0       0.67      0.81      0.74       881
         1.0       0.80      0.65      0.72       999

    accuracy                           0.73      1880
   macro avg       0.73      0.73      0.73      1880
weighted avg       0.74      0.73      0.73      1880
```

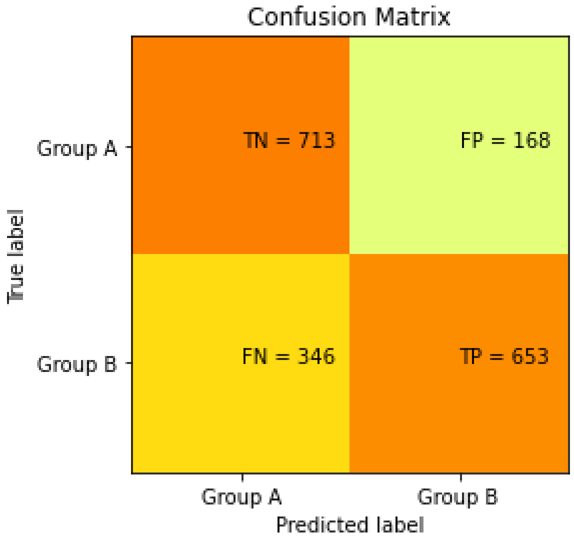


```
AUC Score : 0.7314806293239892

Accuracy Of the Model(SVC): 0.7265957446808511
```

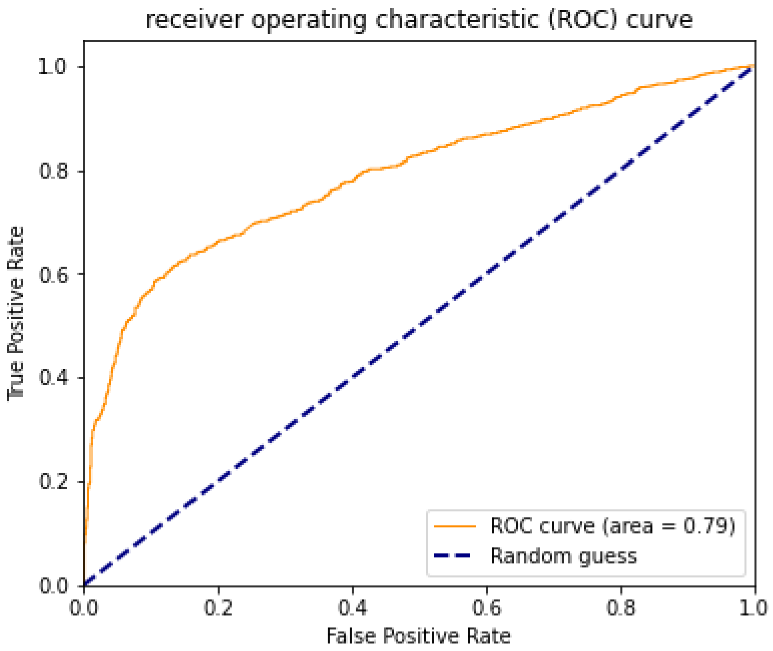

```
ROC_AUC Score of the model(SVC): 0.7878718673270317


Confusion Matrix:
[[784  97]
 [392 607]]

Classification Report:
              precision    recall  f1-score   support

         0.0       0.67      0.89      0.76       881
         1.0       0.86      0.61      0.71       999

    accuracy                           0.74      1880
   macro avg       0.76      0.75      0.74      1880
weighted avg       0.77      0.74      0.74      1880
```

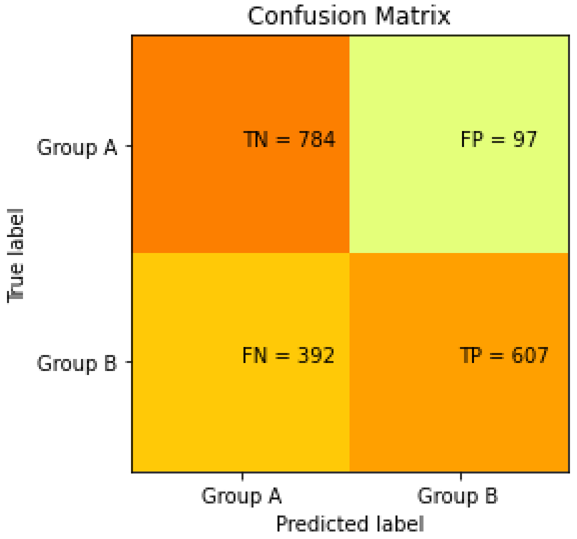


```
AUC Score : 0.7487527254837 13

Accuracy Of the Model(MLPClassifier): 0.7398936170212767
```

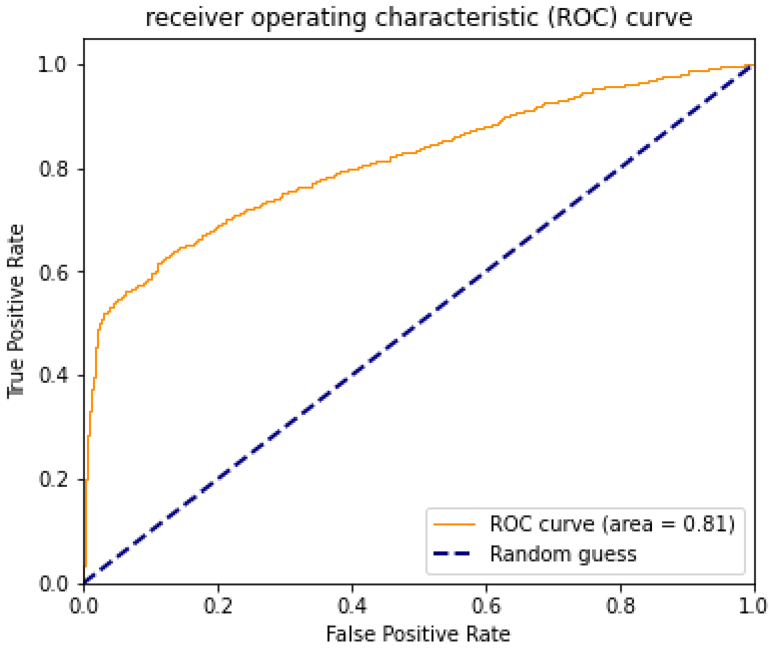

```
ROC_AUC Score of the model(MLPClassifier): 0.8131741275895645

[('RandomForestClassifier', 0.7420212765957447), ('MLPClassifier', 0.7398936170212
767), ('XGBClassifier', 0.7393617021276596), ('GradientBoostingClassifier', 0.7388
297872340426), ('LogisticRegression', 0.7361702127659574), ('DecisionTreeClassifie
r', 0.7361702127659574), ('ExtraTreesClassifier', 0.7351063829787234), ('AdaBoostC
lassifier', 0.7345744680851064), ('SVC', 0.7265957446808511), ('KNeighborsClassifi
er', 0.7207446808510638)]


sorted_total_auc:
 [('RandomForestClassifier', 0.8187188323397177), ('XGBClassifier', 0.818418872902
4142), ('GradientBoostingClassifier', 0.8176451138993704), ('MLPClassifier', 0.813
1741275895645), ('AdaBoostClassifier', 0.8118822568311785), ('LogisticRegression',
0.8099529722685227), ('ExtraTreesClassifier', 0.80694883305553), ('KNeighborsClass
ifier', 0.7997645772901164), ('DecisionTreeClassifier', 0.7920497114594731), ('SV
C', 0.7878718673270317)]
```

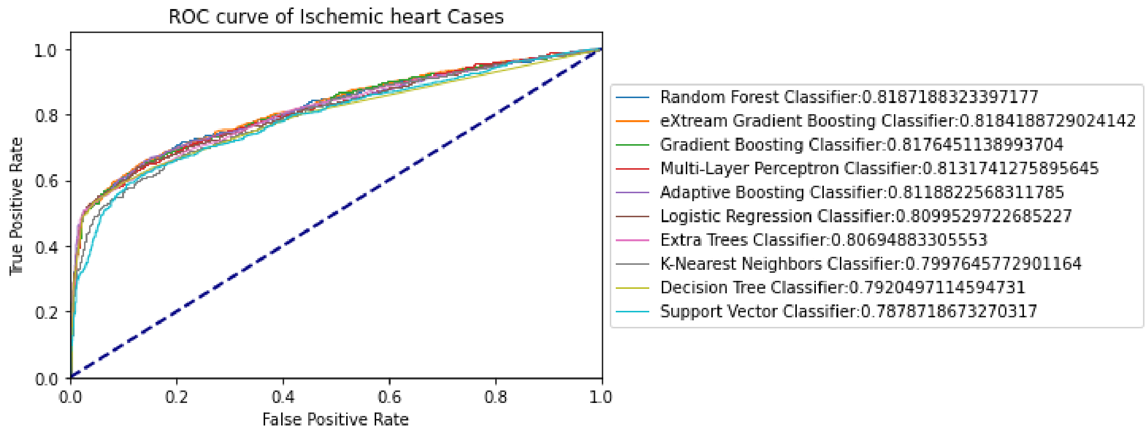

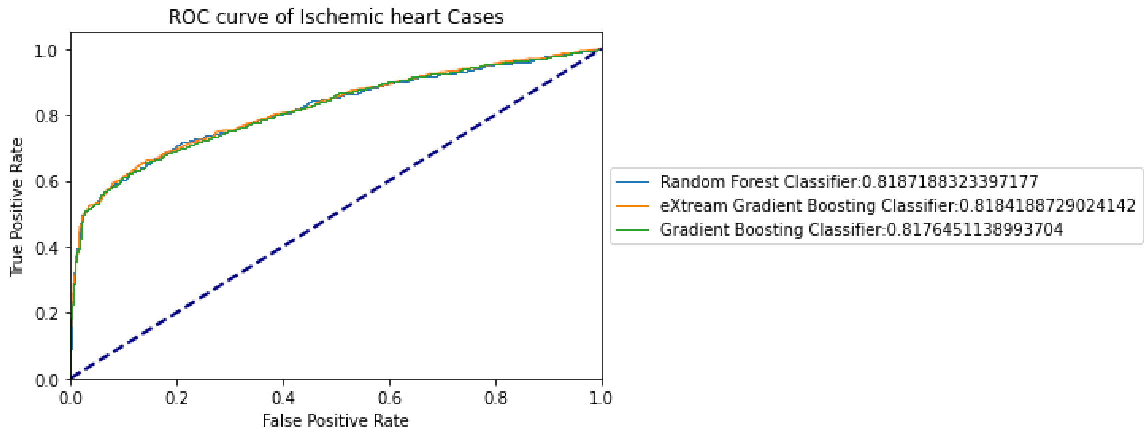

```
random state :   12645
9398
result_train_filename: data/train_test_data_ischemic heart_2013_20210519_140242.cs
v
total count : 9398, df_train count : 7518, df_test count : 1880
(7518, 38)
(1880, 38)
('Logistic Regression', LogisticRegression())
[2021-05-19 14:02:42.982841] Start parameter search for model 'Logistic Regressio
n'
[2021-05-19 14:02:43.341747] Finish parameter search for model 'Logistic Regressio
n' (time: 0 seconds)

=== train : best params ===
{'C': 0.1, 'penalty': 'l2', 'solver': 'lbfgs'}
=== train : best score : roc_auc ===
0.805773688211963



('Random Forest', RandomForestClassifier())
[2021-05-19 14:02:43.342742] Start parameter search for model 'Random Forest'
[2021-05-19 14:02:47.751735] Finish parameter search for model 'Random Forest' (ti
me: 4 seconds)

=== train : best params ===
{'criterion': 'gini', 'max_depth': 10, 'max_features': 'auto', 'n_estimators': 70}
=== train : best score : roc_auc ===
0.8093153272627039



('Extreme Gradient Boosting)', XGBClassifier(base_score=None, booster=None, colsam
ple_bylevel=None,
              colsample_bynode=None, colsample_bytree=None, gamma=None,
              gpu_id=None, importance_type='gain', interaction_constraints=None,
              learning_rate=None, max_delta_step=None, max_depth=None,
              min_child_weight=None, missing=nan, monotone_constraints=None,
              n_estimators=100, n_jobs=None, num_parallel_tree=None,
              random_state=None, reg_alpha=None, reg_lambda=None,
              scale_pos_weight=None, subsample=None, tree_method=None,
              validate_parameters=None, verbosity=None))
[2021-05-19 14:02:47.751735] Start parameter search for model 'Extreme Gradient Bo
osting)'
[2021-05-19 14:03:14.019711] Finish parameter search for model 'Extreme Gradient B
oosting)' (time: 26 seconds)

=== train : best params ===
{'learning_rate': 0.05, 'max_depth': 5, 'n_estimators': 100, 'subsample': 0.8}
=== train : best score : roc_auc ===
0.812445528913273



('k-Nearest Neighbours)', KNeighborsClassifier())
[2021-05-19 14:03:14.021706] Start parameter search for model 'k-Nearest Neighbour
s)'
[2021-05-19 14:03:21.960531] Finish parameter search for model 'k-Nearest Neighbou
rs)' (time: 7 seconds)

=== train : best params ===
{'n_neighbors': 60, 'weights': 'distance'}
=== train : best score : roc_auc ===
0.7894956319777701
```

```
('Decision Tree', DecisionTreeClassifier())
[2021-05-19 14:03:21.960531] Start parameter search for model 'Decision Tree'
[2021-05-19 14:03:22.541495] Finish parameter search for model 'Decision Tree' (ti
me: 0 seconds)

=== train : best params ===
{'criterion': 'entropy', 'max_depth': 5}
=== train : best score : roc_auc ===
0.7935564088248475


('Extra Tree', ExtraTreesClassifier())
[2021-05-19 14:03:22.542493] Start parameter search for model 'Extra Tree'
[2021-05-19 14:03:25.522396] Finish parameter search for model 'Extra Tree' (time:
2 seconds)

=== train : best params ===
{'max_features': 'sqrt', 'n_estimators': 100}
=== train : best score : roc_auc ===
0.8039663820984322


('Gradient Boosting', GradientBoostingClassifier())
[2021-05-19 14:03:25.522396] Start parameter search for model 'Gradient Boosting'
[2021-05-19 14:03:40.484343] Finish parameter search for model 'Gradient Boosting'
(time: 14 seconds)

=== train : best params ===
{'learning_rate': 0.075, 'max_depth': 5, 'max_features': 4, 'n_estimators': 100}
=== train : best score : roc_auc ===
0.8126106754611193


('Adaptive Boosting', AdaBoostClassifier())
[2021-05-19 14:03:40.485341] Start parameter search for model 'Adaptive Boosting'
[2021-05-19 14:03:43.690517] Finish parameter search for model 'Adaptive Boosting'
(time: 3 seconds)

=== train : best params ===
{'learning_rate': 0.2, 'n_estimators': 50}
=== train : best score : roc_auc ===
0.805025007873173


('Suppport Vector Classfier', SVC(probability=True))
[2021-05-19 14:03:43.690517] Start parameter search for model 'Suppport Vector Cla
ssfier'
[2021-05-19 14:08:34.088793] Finish parameter search for model 'Suppport Vector Cl
assfier' (time: 290 seconds)

=== train : best params ===
{'C': 1, 'gamma': 0.1, 'kernel': 'rbf'}
=== train : best score : roc_auc ===
0.7880820193522942


('Neural Network', MLPClassifier())
[2021-05-19 14:08:34.088793] Start parameter search for model 'Neural Network'
[2021-05-19 14:11:09.365324] Finish parameter search for model 'Neural Network' (t
```

```
ime: 155 seconds)

=== train : best params ===
{'activation': 'logistic', 'alpha': 0.05, 'hidden_layer_sizes': (50,), 'learning_r
ate': 'constant'}
=== train : best score : roc_auc ===
0.8115712305813292



Confusion Matrix:
[[813  68]
 [422 577]]

Classification Report:
              precision    recall  f1-score   support

         0.0       0.66      0.92      0.77       881
         1.0       0.89      0.58      0.70       999

    accuracy                           0.74      1880
   macro avg       0.78      0.75      0.74      1880
weighted avg       0.78      0.74      0.73      1880
```

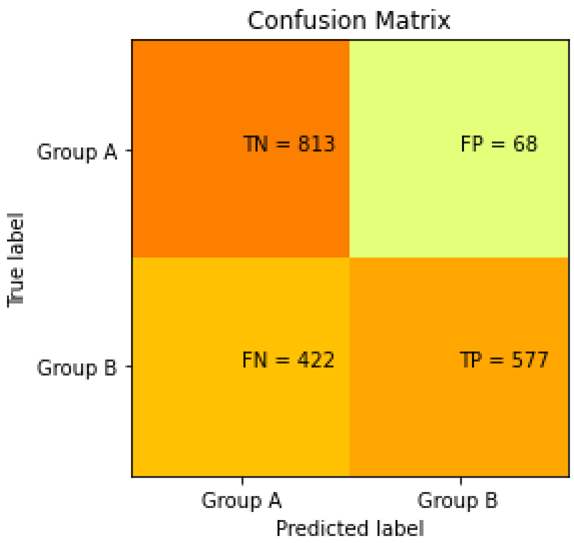


```
AUC Score : 0.7501962802757355

Accuracy Of the Model(LogisticRegression): 0.7393617021276596
```

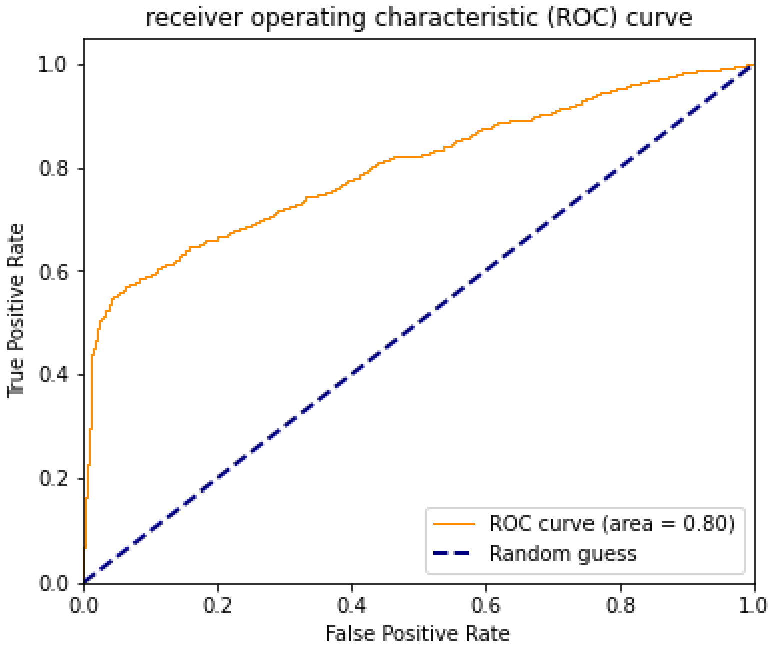


```
ROC_AUC Score of the model(LogisticRegression): 0.8014461680749989


Confusion Matrix:
[[802  79]
 [409 590]]

Classification Report:
              precision    recall  f1-score   support

         0.0       0.66      0.91      0.77       881
         1.0       0.88      0.59      0.71       999

    accuracy                           0.74      1880
   macro avg       0.77      0.75      0.74      1880
weighted avg       0.78      0.74      0.74      1880
```

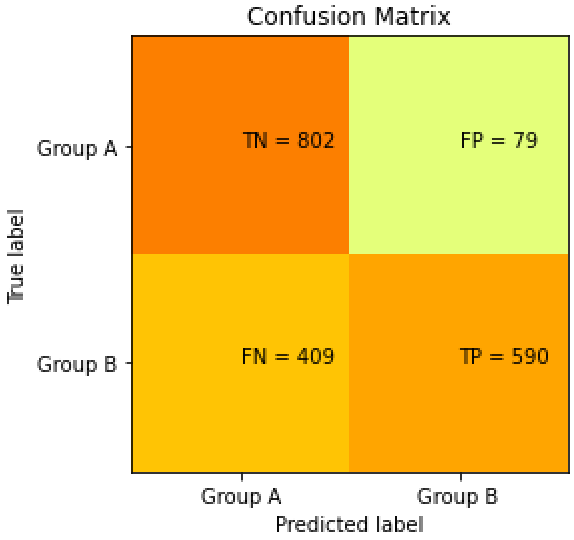

AUC Score : 0.7504598809933657

Accuracy Of the Model(RandomForestClassifier): 0.7404255319148936

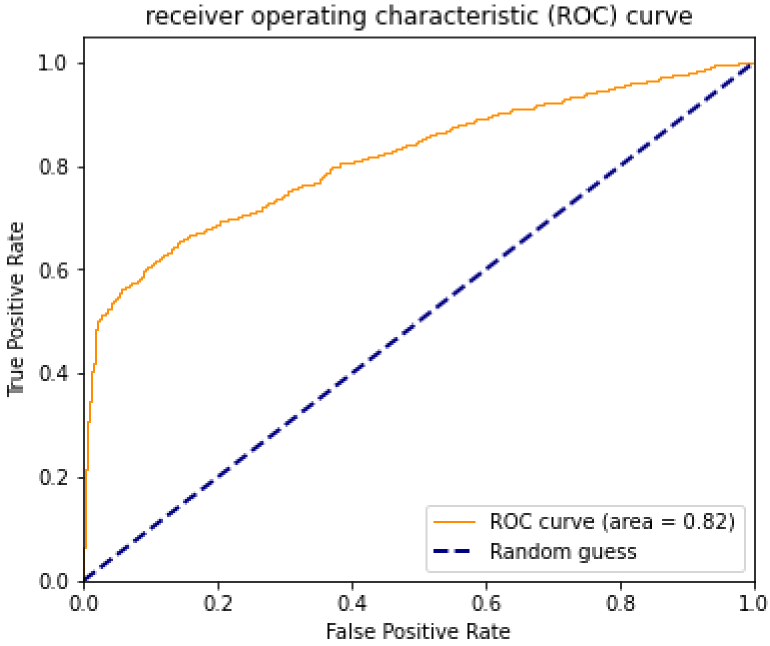

```
ROC_AUC Score of the model(RandomForestClassifier): 0.8159657955344675


Confusion Matrix:
[[795  86]
 [403 596]]

Classification Report:
              precision    recall  f1-score   support

         0.0       0.66      0.90      0.76       881
         1.0       0.87      0.60      0.71       999

    accuracy                           0.74      1880
   macro avg       0.77      0.75      0.74      1880
weighted avg       0.78      0.74      0.74      1880
```

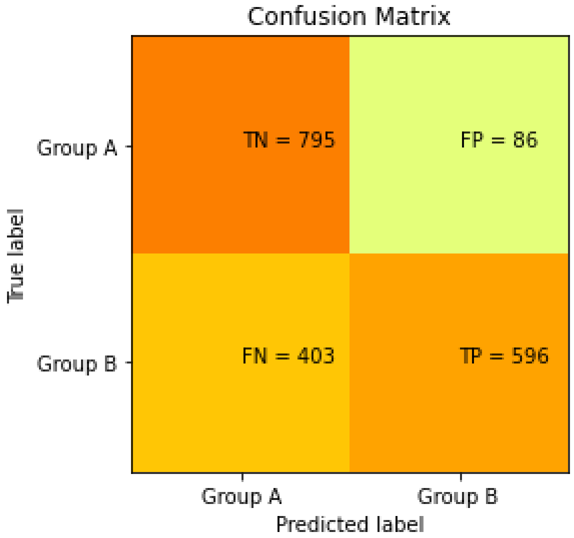


```
AUC Score : 0.7494901257670838

Accuracy Of the Model(XGBClassifier): 0.7398936170212767
```

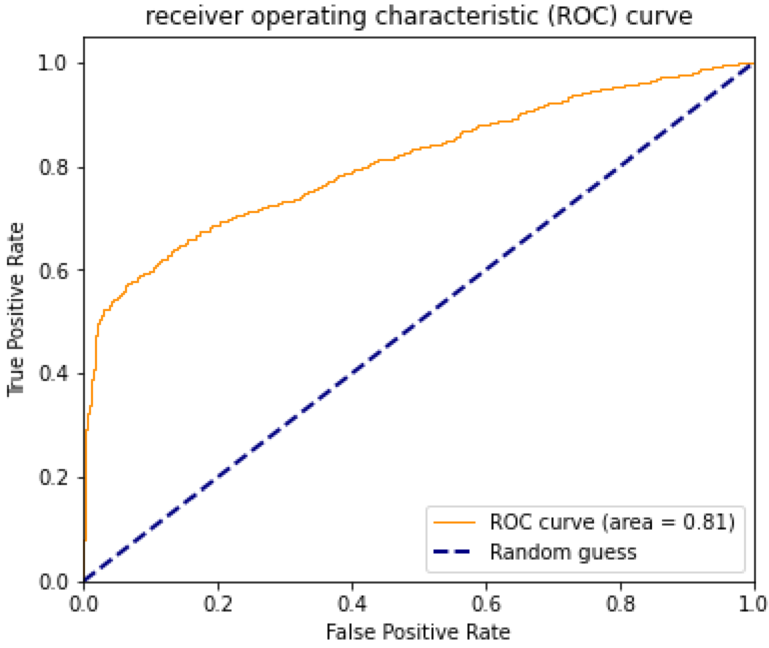

```
ROC_AUC Score of the model(XGBClassifier): 0.8101518090167352


Confusion Matrix:
[[797  84]
 [467 532]]

Classification Report:
              precision    recall  f1-score   support

         0.0       0.63      0.90      0.74       881
         1.0       0.86      0.53      0.66       999

    accuracy                           0.71      1880
   macro avg       0.75      0.72      0.70      1880
weighted avg       0.75      0.71      0.70      1880
```

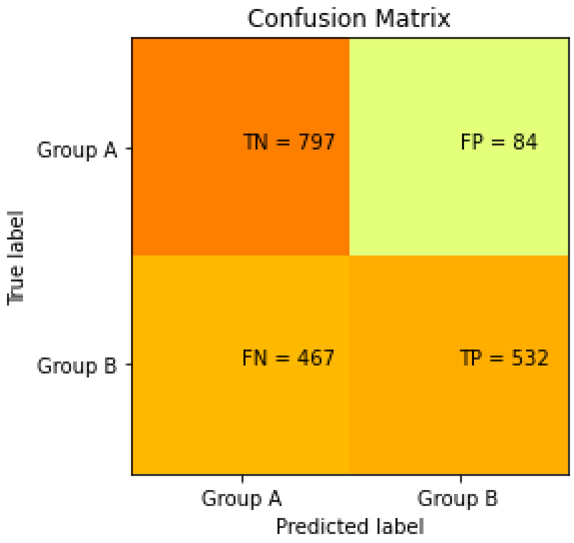


```
AUC Score : 0.7185931675148475

Accuracy Of the Model(KNeighborsClassifier): 0.7069148936170213
```

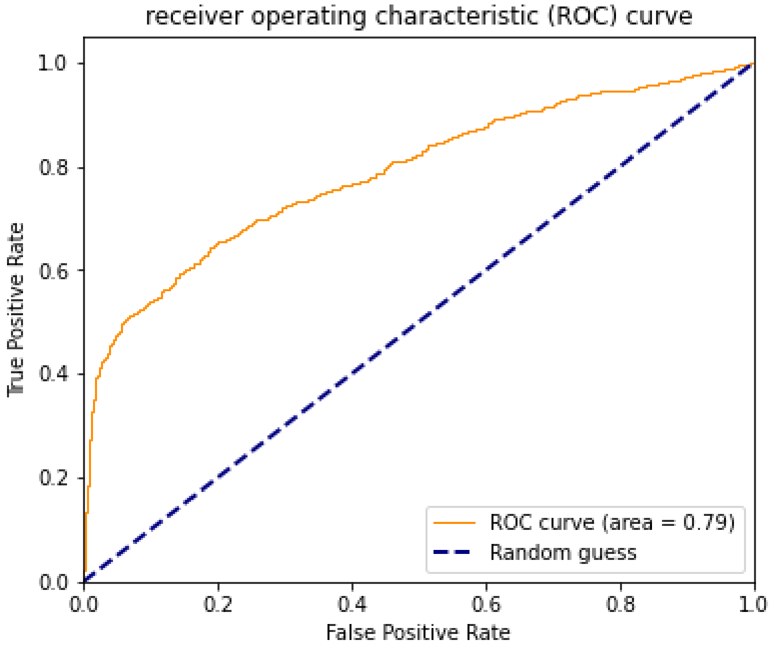

```
ROC_AUC Score of the model(KNeighborsClassifier): 0.7891376052556529


Confusion Matrix:
[[823  58]
 [442 557]]

Classification Report:
              precision    recall  f1-score   support

         0.0       0.65      0.93      0.77       881
         1.0       0.91      0.56      0.69       999

    accuracy                           0.73      1880
   macro avg       0.78      0.75      0.73      1880
weighted avg       0.79      0.73      0.73      1880
```

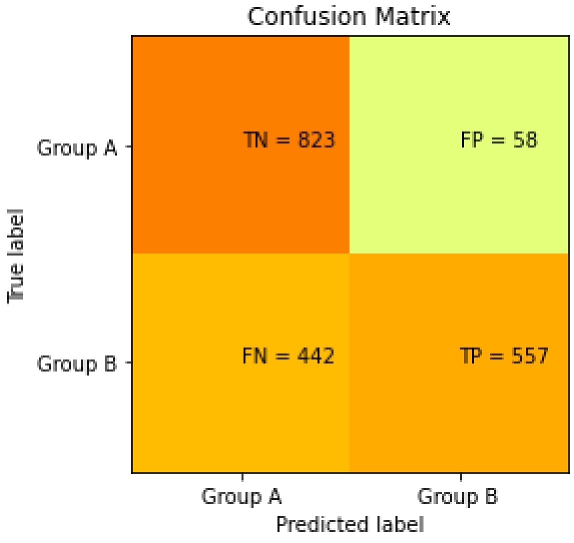


```
AUC Score : 0.7458616391647038

Accuracy Of the Model(DecisionTreeClassifier): 0.7340425531914894
```

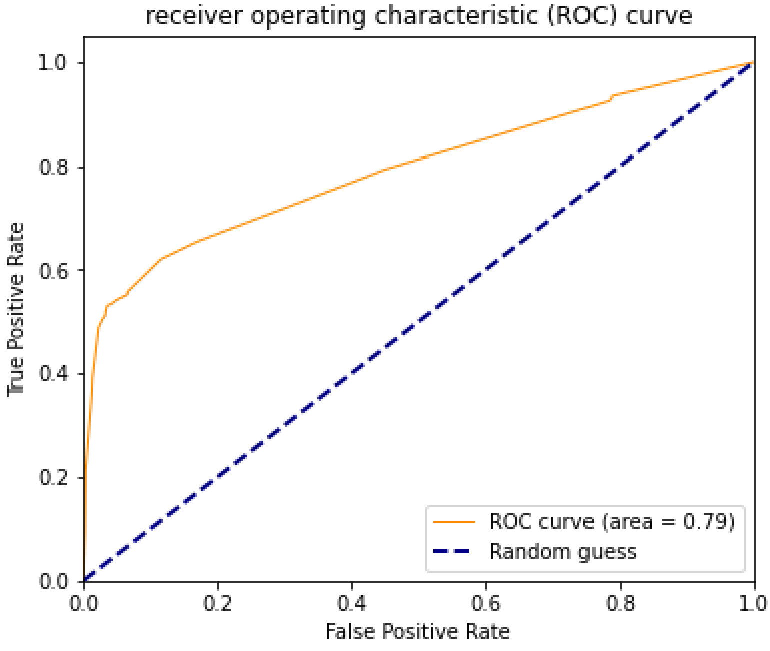

```
ROC_AUC Score of the model(DecisionTreeClassifier): 0.7924047770812811


Confusion Matrix:
[[785  96]
 [419 580]]

Classification Report:
              precision    recall  f1-score   support

         0.0       0.65      0.89      0.75       881
         1.0       0.86      0.58      0.69       999

    accuracy                           0.73      1880
   macro avg       0.75      0.74      0.72      1880
weighted avg       0.76      0.73      0.72      1880
```

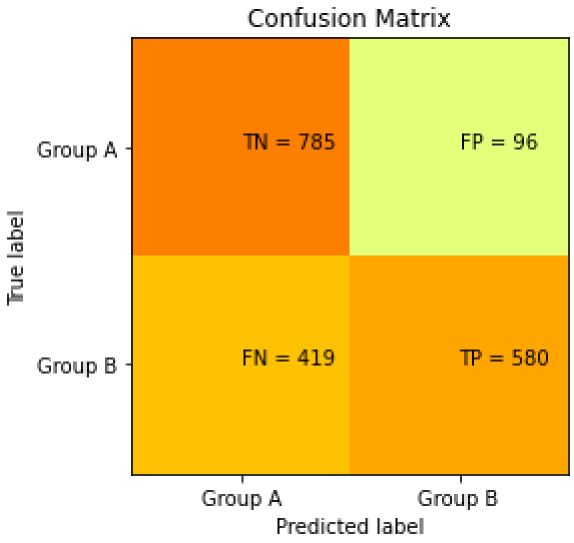


```
AUC Score : 0.7358067488600973

Accuracy Of the Model(ExtraTreesClassifier): 0.726063829787234
```

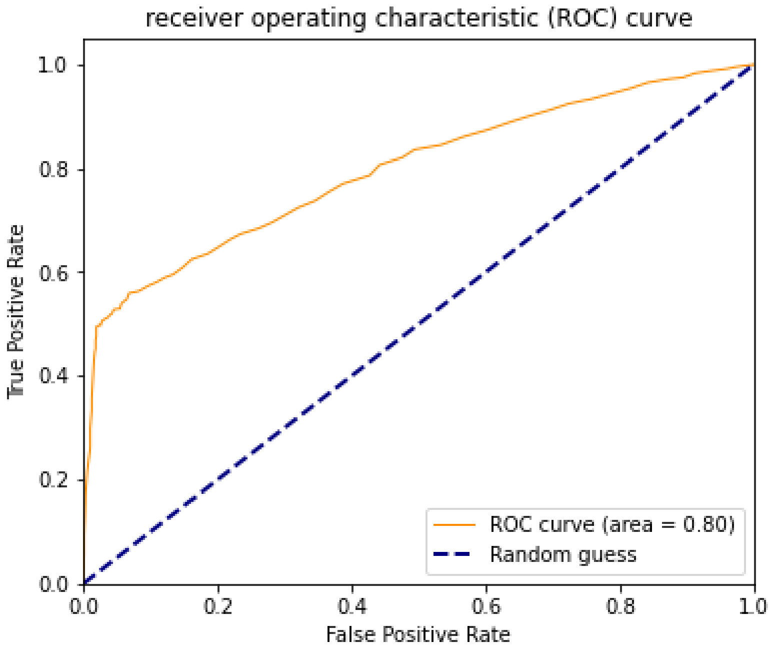

```
ROC_AUC Score of the model(ExtraTreesClassifier): 0.7965911427886456


Confusion Matrix:
[[799  82]
 [395 604]]

Classification Report:
              precision    recall  f1-score   support

         0.0       0.67      0.91      0.77       881
         1.0       0.88      0.60      0.72       999

    accuracy                           0.75      1880
   macro avg       0.77      0.76      0.74      1880
weighted avg       0.78      0.75      0.74      1880
```

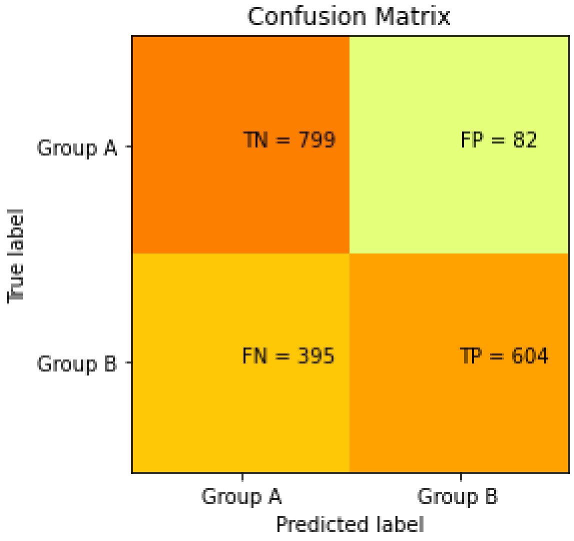


```
AUC Score : 0.7557642773306792

Accuracy Of the Model(GradientBoostingClassifier): 0.7462765957446809
```

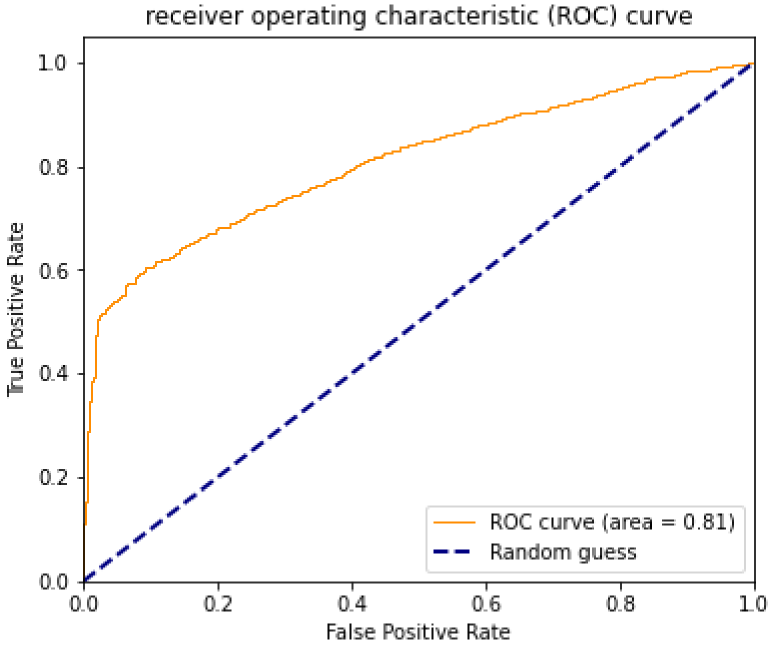

```
ROC_AUC Score of the model(GradientBoostingClassifier): 0.8099257032287679


Confusion Matrix:
[[827  54]
 [449 550]]

Classification Report:
              precision    recall  f1-score   support

         0.0       0.65      0.94      0.77       881
         1.0       0.91      0.55      0.69       999

    accuracy                           0.73      1880
   macro avg       0.78      0.74      0.73      1880
weighted avg       0.79      0.73      0.72      1880
```

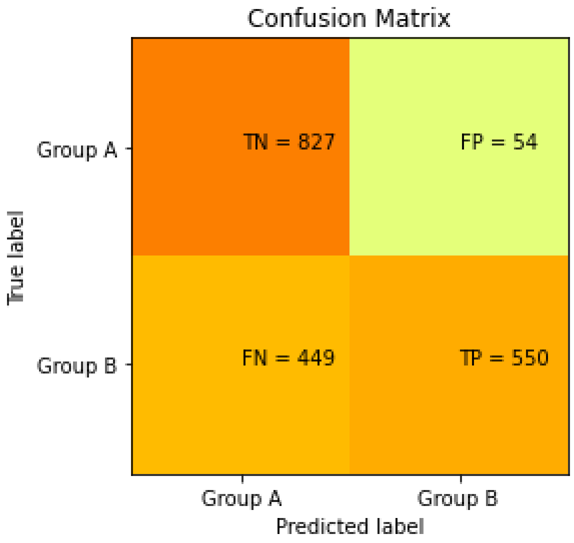


```
AUC Score : 0.7446282832207918

Accuracy Of the Model(AdaBoostClassifier): 0.7324468085106383
```

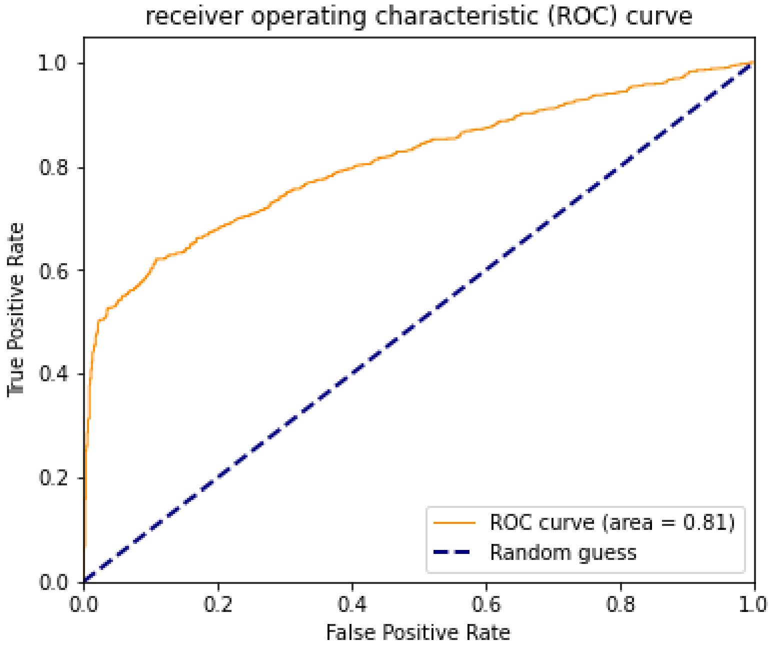

```
ROC_AUC Score of the model(AdaBoostClassifier): 0.8077856517130071


Confusion Matrix:
[[703 178]
 [352 647]]

Classification Report:
              precision    recall  f1-score   support

         0.0       0.67      0.80      0.73       881
         1.0       0.78      0.65      0.71       999

    accuracy                           0.72      1880
   macro avg       0.73      0.72      0.72      1880
weighted avg       0.73      0.72      0.72      1880
```

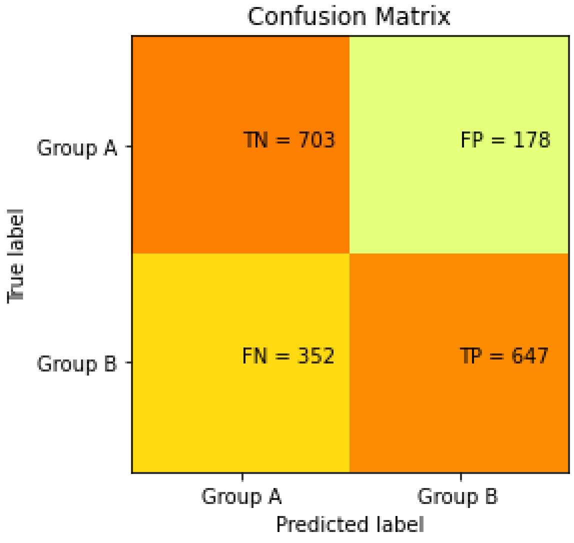


```
AUC Score : 0.7228022574220077

Accuracy Of the Model(SVC): 0.7180851063829787
```

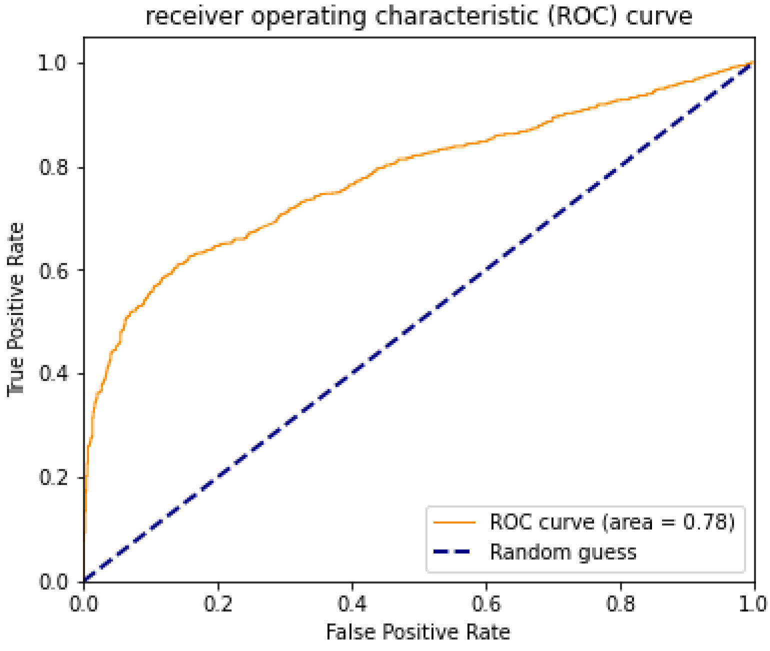

```
ROC_AUC Score of the model(SVC): 0.7786458422099738


Confusion Matrix:
[[798  83]
 [413 586]]

Classification Report:
              precision    recall  f1-score   support

         0.0       0.66      0.91      0.76       881
         1.0       0.88      0.59      0.70       999

    accuracy                           0.74      1880
   macro avg       0.77      0.75      0.73      1880
weighted avg       0.77      0.74      0.73      1880
```

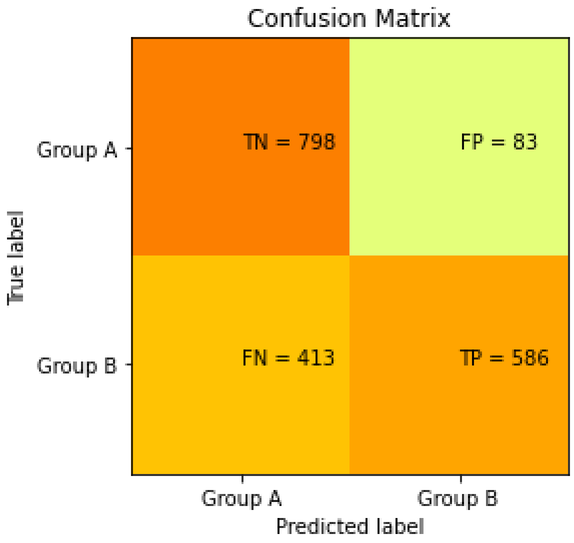


```
AUC Score : 0.7461877314317722

Accuracy Of the Model(MLPClassifier): 0.7361702127659574
```

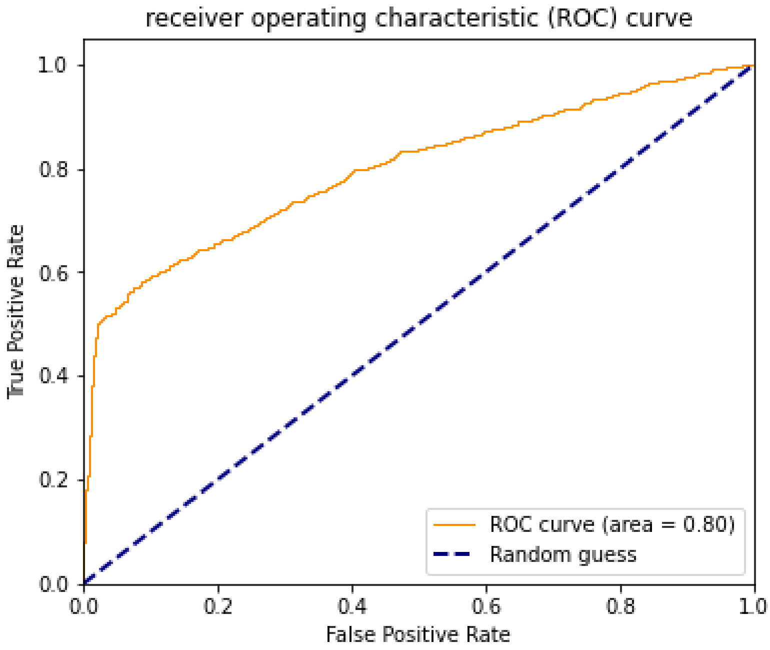

```
ROC_AUC Score of the model(MLPClassifier): 0.7996259596713626

[('GradientBoostingClassifier', 0.7462765957446809), ('RandomForestClassifier', 0.7404255319148936), ('XGBClassifier', 0.7398936170212767), ('LogisticRegression', 0.7393617021276596), ('MLPClassifier', 0.7361702127659574), ('DecisionTreeClassifier', 0.7340425531914894), ('AdaBoostClassifier', 0.7324468085106383), ('ExtraTreesClassifier', 0.726063829787234), ('SVC', 0.7180851063829787), ('KNeighborsClassifier', 0.7069148936170213)]


sorted_total_auc:
 [('RandomForestClassifier', 0.8159657955344675), ('XGBClassifier', 0.8101518090167352), ('GradientBoostingClassifier', 0.8099257032287679), ('AdaBoostClassifier', 0.8077856517130071), ('LogisticRegression', 0.8014461680749989), ('MLPClassifier', 0.7996259596713626), ('ExtraTreesClassifier', 0.7965911427886456), ('DecisionTreeClassifier', 0.7924047770812811), ('KNeighborsClassifier', 0.7891376052556529), ('SVC', 0.7786458422099738)]
```

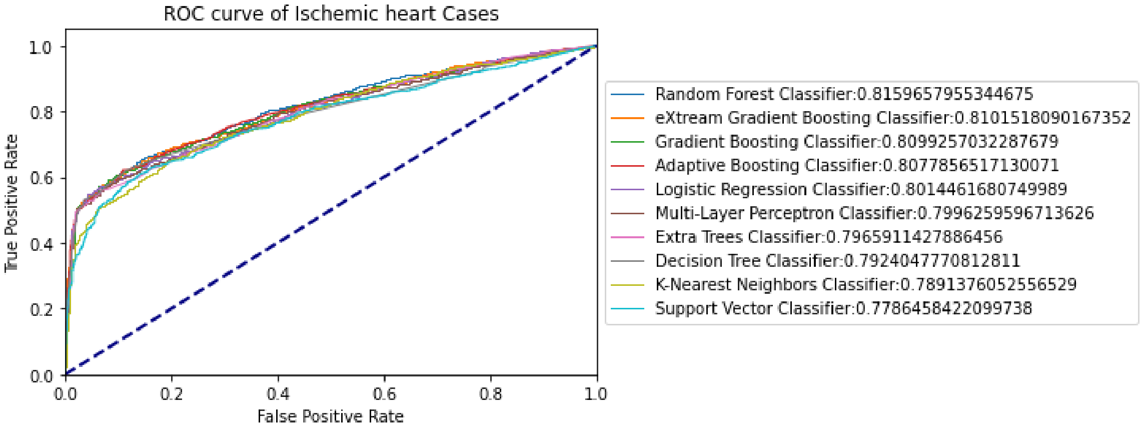

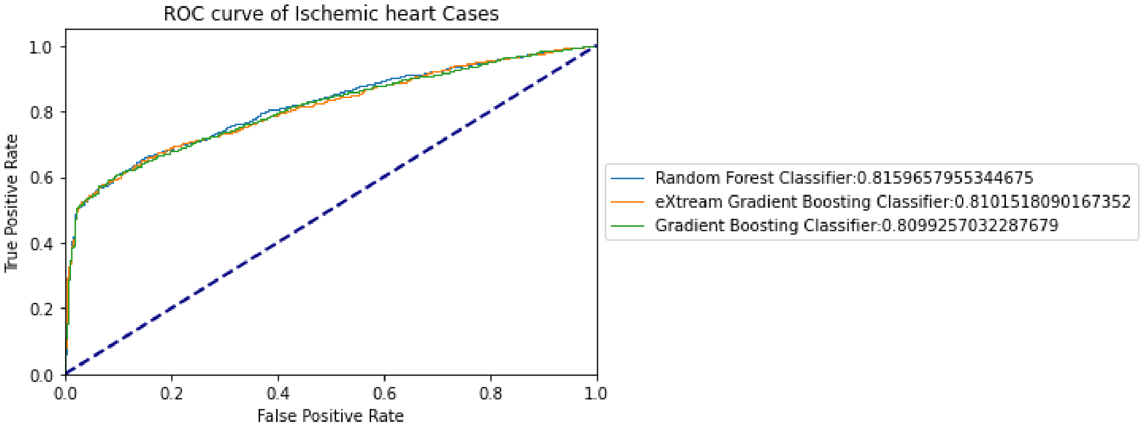

```
random state :   12670
9398
result_train_filename: data/train_test_data_ischemic heart_2013_20210519_141116.cs
v
total count : 9398, df_train count : 7518, df_test count : 1880
(7518, 38)
(1880, 38)
('Logistic Regression', LogisticRegression())
[2021-05-19 14:11:17.085487] Start parameter search for model 'Logistic Regressio
n'
[2021-05-19 14:11:17.448530] Finish parameter search for model 'Logistic Regressio
n' (time: 0 seconds)

=== train : best params ===
{'C': 10, 'penalty': 'l2', 'solver': 'lbfgs'}
=== train : best score : roc_auc ===
0.8035620049731276



('Random Forest', RandomForestClassifier())
[2021-05-19 14:11:17.449527] Start parameter search for model 'Random Forest'
[2021-05-19 14:11:21.816571] Finish parameter search for model 'Random Forest' (ti
me: 4 seconds)

=== train : best params ===
{'criterion': 'gini', 'max_depth': 10, 'max_features': 'auto', 'n_estimators': 90}
=== train : best score : roc_auc ===
0.8090394646729827



('Extreme Gradient Boosting)', XGBClassifier(base_score=None, booster=None, colsam
ple_bylevel=None,
              colsample_bynode=None, colsample_bytree=None, gamma=None,
              gpu_id=None, importance_type='gain', interaction_constraints=None,
              learning_rate=None, max_delta_step=None, max_depth=None,
              min_child_weight=None, missing=nan, monotone_constraints=None,
              n_estimators=100, n_jobs=None, num_parallel_tree=None,
              random_state=None, reg_alpha=None, reg_lambda=None,
              scale_pos_weight=None, subsample=None, tree_method=None,
              validate_parameters=None, verbosity=None))
[2021-05-19 14:11:21.817572] Start parameter search for model 'Extreme Gradient Bo
osting)'
[2021-05-19 14:11:48.178488] Finish parameter search for model 'Extreme Gradient B
oosting)' (time: 26 seconds)

=== train : best params ===
{'learning_rate': 0.05, 'max_depth': 5, 'n_estimators': 50, 'subsample': 0.7}
=== train : best score : roc_auc ===
0.8101591587087882



('k-Nearest Neighbours)', KNeighborsClassifier())
[2021-05-19 14:11:48.180482] Start parameter search for model 'k-Nearest Neighbour
s)'
[2021-05-19 14:11:56.108633] Finish parameter search for model 'k-Nearest Neighbou
rs)' (time: 7 seconds)

=== train : best params ===
{'n_neighbors': 60, 'weights': 'distance'}
=== train : best score : roc_auc ===
0.7865088931149012
```

```
('Decision Tree', DecisionTreeClassifier())
[2021-05-19 14:11:56.108633] Start parameter search for model 'Decision Tree'
[2021-05-19 14:11:56.679986] Finish parameter search for model 'Decision Tree' (ti
me: 0 seconds)

=== train : best params ===
{'criterion': 'entropy', 'max_depth': 4}
=== train : best score : roc_auc ===
0.7918624250094402


('Extra Tree', ExtraTreesClassifier())
[2021-05-19 14:11:56.680990] Start parameter search for model 'Extra Tree'
[2021-05-19 14:11:59.779464] Finish parameter search for model 'Extra Tree' (time:
3 seconds)

=== train : best params ===
{'max_features': 'sqrt', 'n_estimators': 100}
=== train : best score : roc_auc ===
0.8016311991042495


('Gradient Boosting', GradientBoostingClassifier())
[2021-05-19 14:11:59.779464] Start parameter search for model 'Gradient Boosting'
[2021-05-19 14:12:16.892680] Finish parameter search for model 'Gradient Boosting'
(time: 17 seconds)

=== train : best params ===
{'learning_rate': 0.01, 'max_depth': 8, 'max_features': 6, 'n_estimators': 100}
=== train : best score : roc_auc ===
0.8112247368721188


('Adaptive Boosting', AdaBoostClassifier())
[2021-05-19 14:12:16.892680] Start parameter search for model 'Adaptive Boosting'
[2021-05-19 14:12:20.560287] Finish parameter search for model 'Adaptive Boosting'
(time: 3 seconds)

=== train : best params ===
{'learning_rate': 0.2, 'n_estimators': 100}
=== train : best score : roc_auc ===
0.803247729930106


('Suppport Vector Classfier', SVC(probability=True))
[2021-05-19 14:12:20.561283] Start parameter search for model 'Suppport Vector Cla
ssfier'
[2021-05-19 14:16:39.303753] Finish parameter search for model 'Suppport Vector Cl
assfier' (time: 258 seconds)

=== train : best params ===
{'C': 1, 'gamma': 0.1, 'kernel': 'rbf'}
=== train : best score : roc_auc ===
0.7813259153480696


('Neural Network', MLPClassifier())
[2021-05-19 14:16:39.304763] Start parameter search for model 'Neural Network'
[2021-05-19 14:19:08.677211] Finish parameter search for model 'Neural Network' (t
```

```
ime: 149 seconds)

=== train : best params ===
{'activation': 'logistic', 'alpha': 0.05, 'hidden_layer_sizes': (50,), 'learning_r
ate': 'adaptive'}
=== train : best score : roc_auc ===
0.8080850234891009



Confusion Matrix:
[[820  61]
 [426 573]]

Classification Report:
              precision    recall  f1-score   support

         0.0       0.66      0.93      0.77       881
         1.0       0.90      0.57      0.70       999

    accuracy                           0.74      1880
   macro avg       0.78      0.75      0.74      1880
weighted avg       0.79      0.74      0.73      1880
```

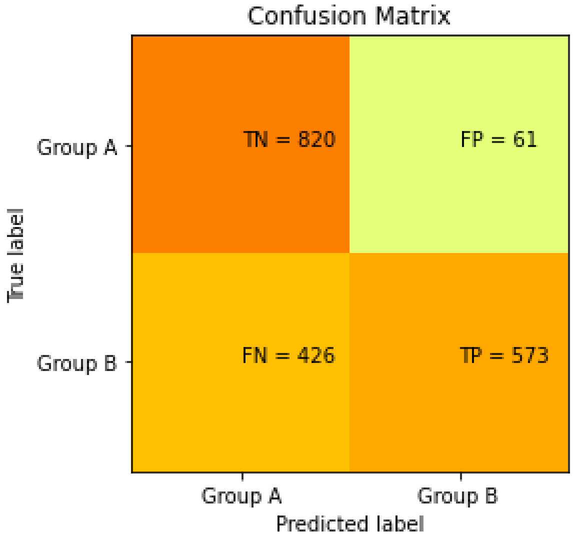


```
AUC Score : 0.7521670365030183

Accuracy Of the Model(LogisticRegression): 0.7409574468085106
```

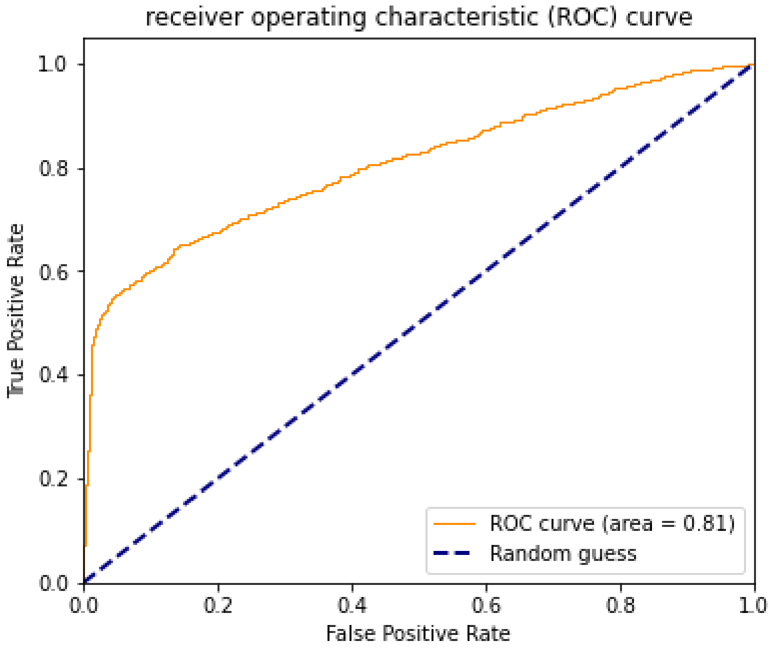


```
ROC_AUC Score of the model(LogisticRegression): 0.8078100802277873


Confusion Matrix:
[[806  75]
 [407 592]]

Classification Report:
              precision    recall  f1-score   support

         0.0       0.66      0.91      0.77       881
         1.0       0.89      0.59      0.71       999

    accuracy                           0.74      1880
   macro avg       0.78      0.75      0.74      1880
weighted avg       0.78      0.74      0.74      1880
```

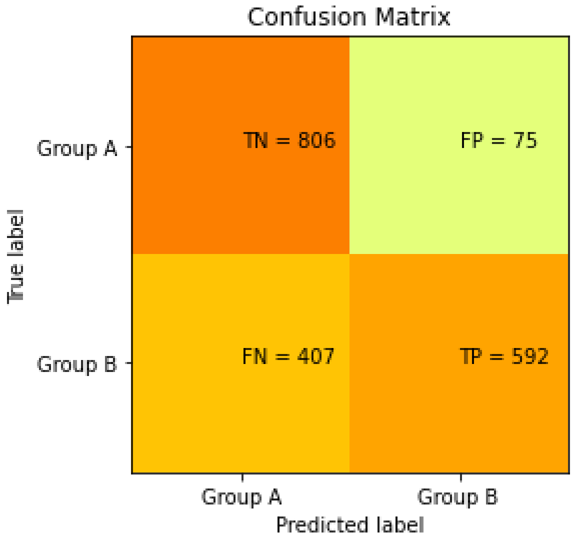

AUC Score : 0.7537310295539558

Accuracy Of the Model(RandomForestClassifier): 0.7436170212765958

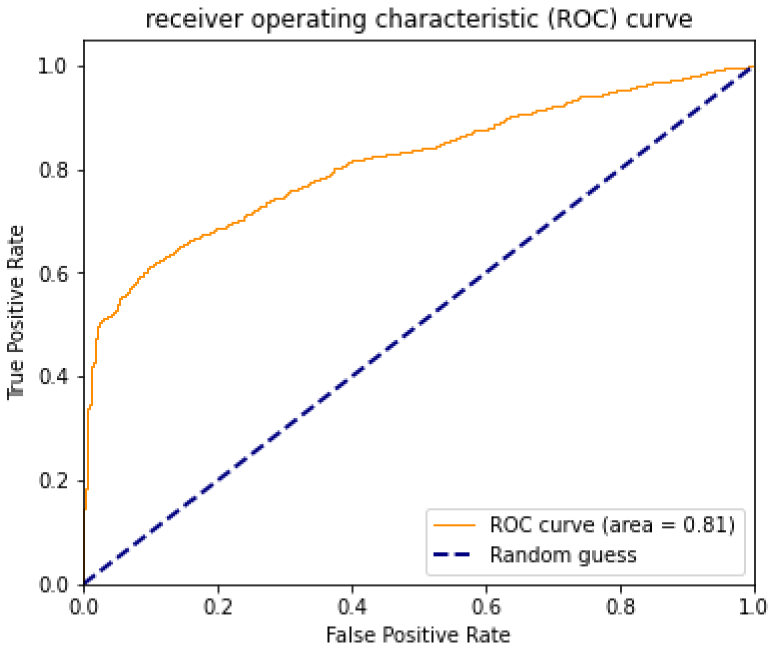

ROC_AUC Score of the model(RandomForestClassifier): 0.8145114467475421

```
Confusion Matrix:
[[820  61]
 [415 584]]

Classification Report:
              precision    recall  f1-score   support

         0.0       0.66      0.93      0.78       881
         1.0       0.91      0.58      0.71       999

    accuracy                           0.75      1880
   macro avg       0.78      0.76      0.74      1880
weighted avg       0.79      0.75      0.74      1880
```

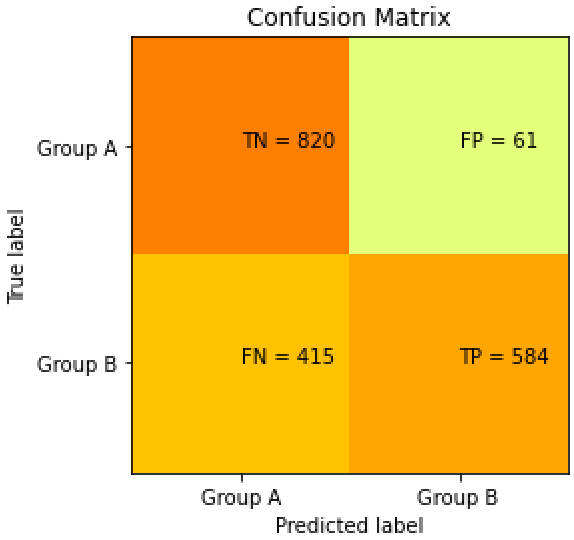

AUC Score : 0.7576725420085239

Accuracy Of the Model(XGBClassifier): 0.7468085106382979

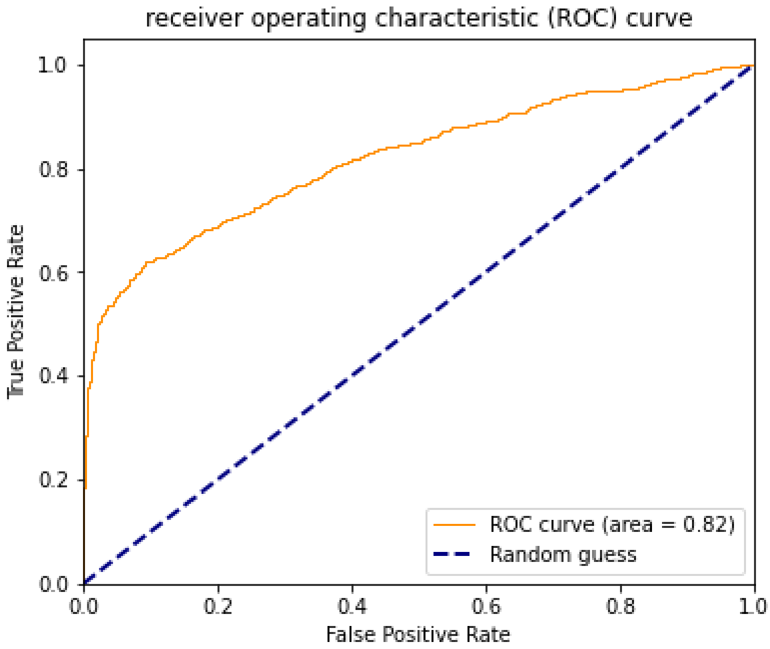

ROC_AUC Score of the model(XGBClassifier): 0.8209764815894214

```
Confusion Matrix:
[[794  87]
 [464 535]]

Classification Report:
              precision    recall  f1-score   support

         0.0       0.63      0.90      0.74       881
         1.0       0.86      0.54      0.66       999

    accuracy                           0.71      1880
   macro avg       0.75      0.72      0.70      1880
weighted avg       0.75      0.71      0.70      1880
```

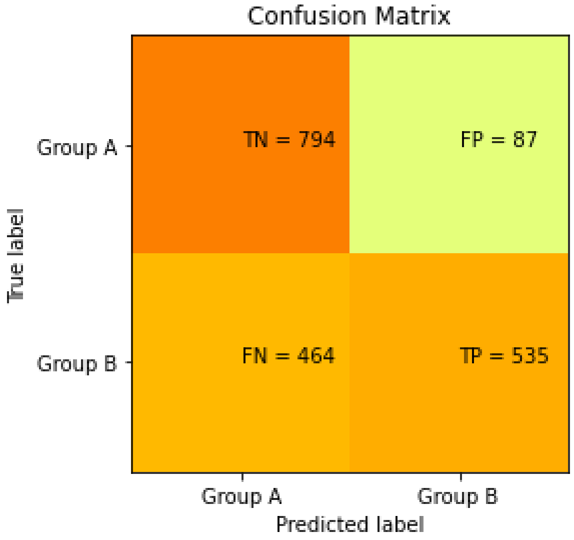

AUC Score : 0.7183920583466554

Accuracy Of the Model(KNeighborsClassifier): 0.7069148936170213

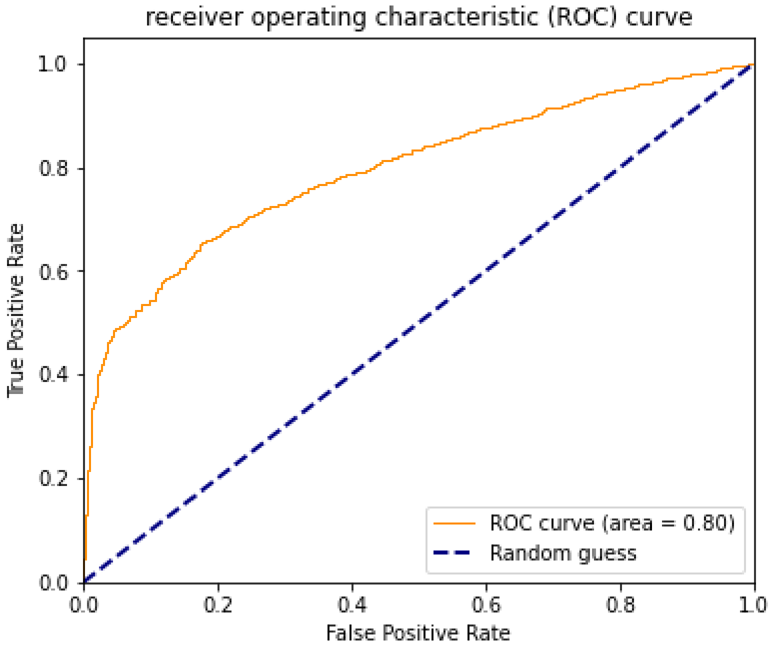

```
ROC_AUC Score of the model(KNeighborsClassifier): 0.7967093086275834


Confusion Matrix:
[[829  52]
 [460 539]]

Classification Report:
              precision    recall  f1-score   support

         0.0       0.64      0.94      0.76       881
         1.0       0.91      0.54      0.68       999

    accuracy                           0.73      1880
   macro avg       0.78      0.74      0.72      1880
weighted avg       0.79      0.73      0.72      1880
```

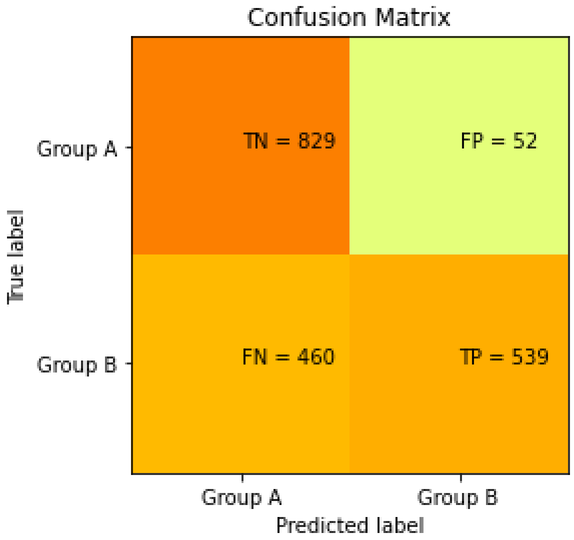


```
AUC Score : 0.7402578514950082

Accuracy Of the Model(DecisionTreeClassifier): 0.7276595744680852
```

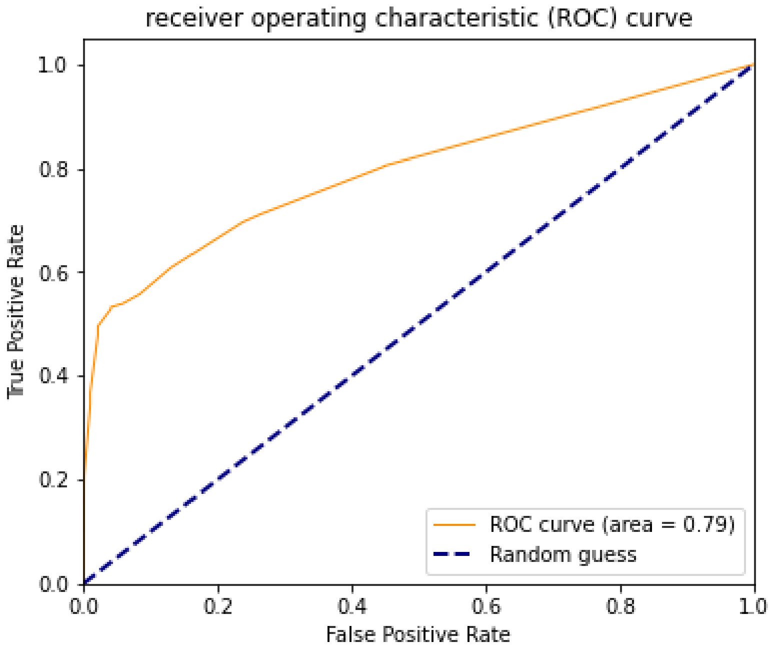

```
ROC_AUC Score of the model(DecisionTreeClassifier): 0.7938909397479205


Confusion Matrix:
[[792  89]
 [405 594]]

Classification Report:
              precision    recall  f1-score   support

         0.0       0.66      0.90      0.76       881
         1.0       0.87      0.59      0.71       999

    accuracy                           0.74      1880
   macro avg       0.77      0.75      0.73      1880
weighted avg       0.77      0.74      0.73      1880
```

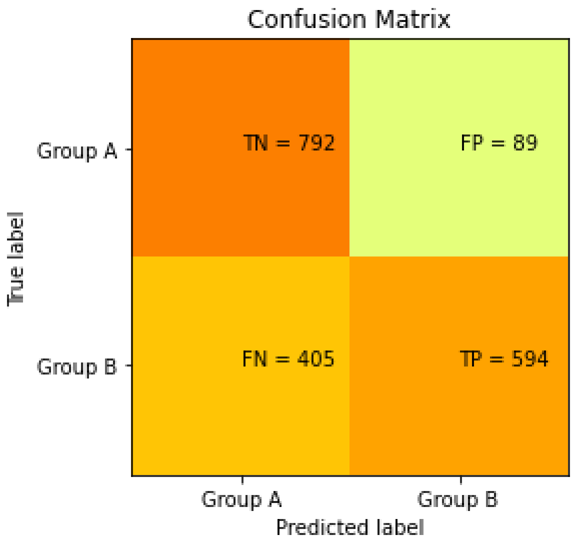


```
AUC Score : 0.7467865140963893

Accuracy Of the Model(ExtraTreesClassifier): 0.7372340425531915
```

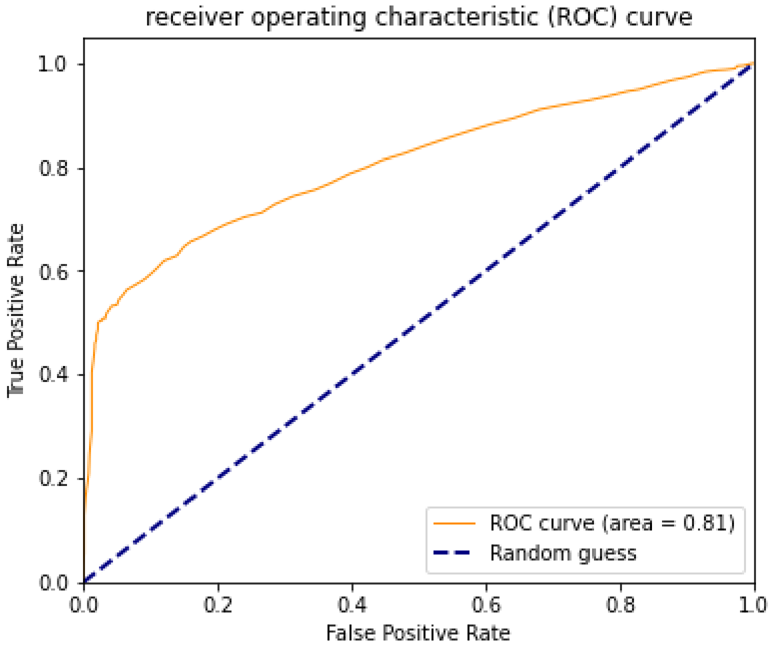

```
ROC_AUC Score of the model(ExtraTreesClassifier): 0.8055234576233441


Confusion Matrix:
[[805  76]
 [405 594]]

Classification Report:
              precision    recall  f1-score   support

         0.0       0.67      0.91      0.77       881
         1.0       0.89      0.59      0.71       999

    accuracy                           0.74      1880
   macro avg       0.78      0.75      0.74      1880
weighted avg       0.78      0.74      0.74      1880
```

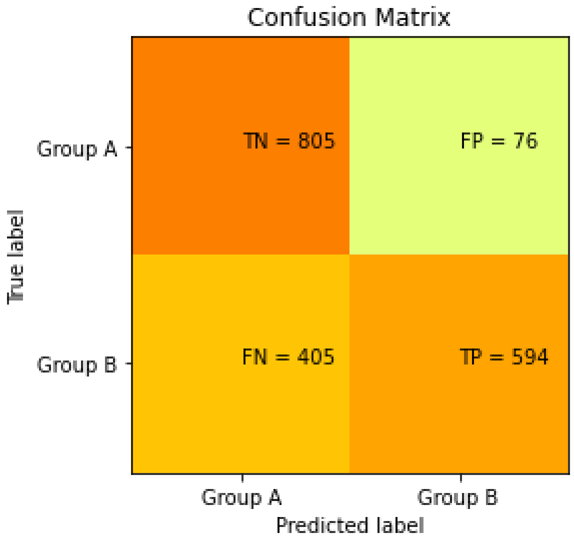


```
AUC Score : 0.7541644936650612

Accuracy Of the Model(GradientBoostingClassifier): 0.7441489361702127
```

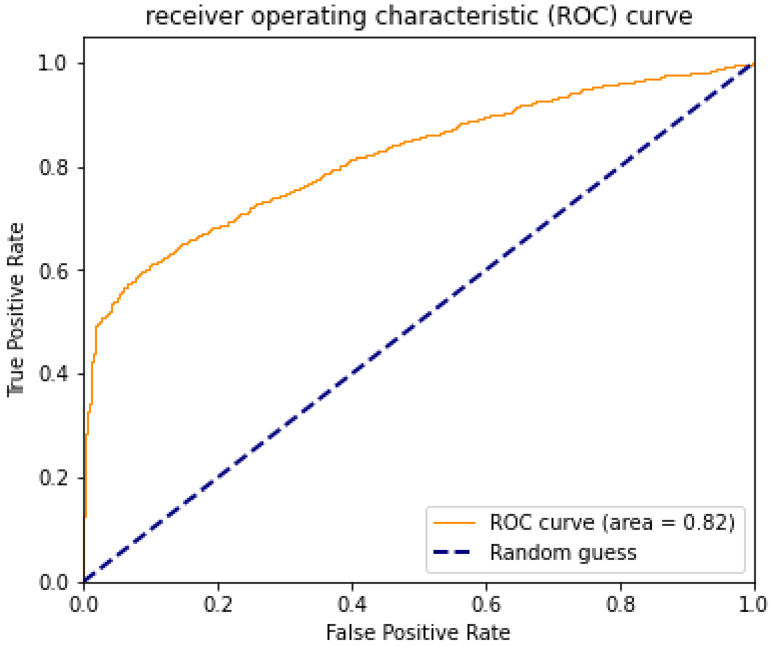

```
ROC_AUC Score of the model(GradientBoostingClassifier): 0.8188222274487882


Confusion Matrix:
[[818  63]
 [437 562]]

Classification Report:
              precision    recall  f1-score   support

         0.0       0.65      0.93      0.77       881
         1.0       0.90      0.56      0.69       999

    accuracy                           0.73      1880
   macro avg       0.78      0.75      0.73      1880
weighted avg       0.78      0.73      0.73      1880
```

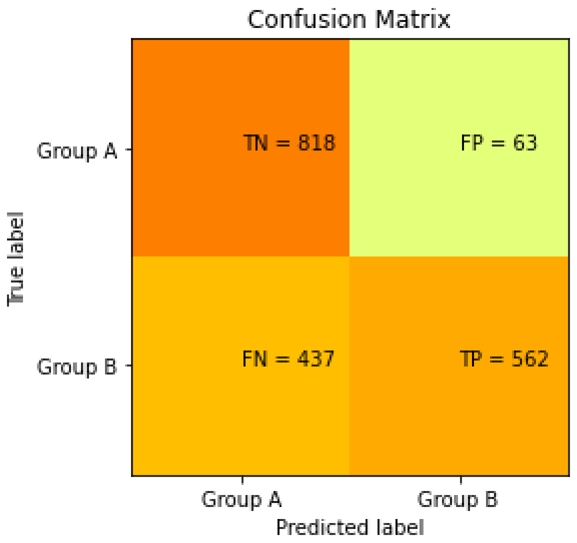


```
AUC Score : 0.7455264572177172

Accuracy Of the Model(AdaBoostClassifier): 0.7340425531914894
```

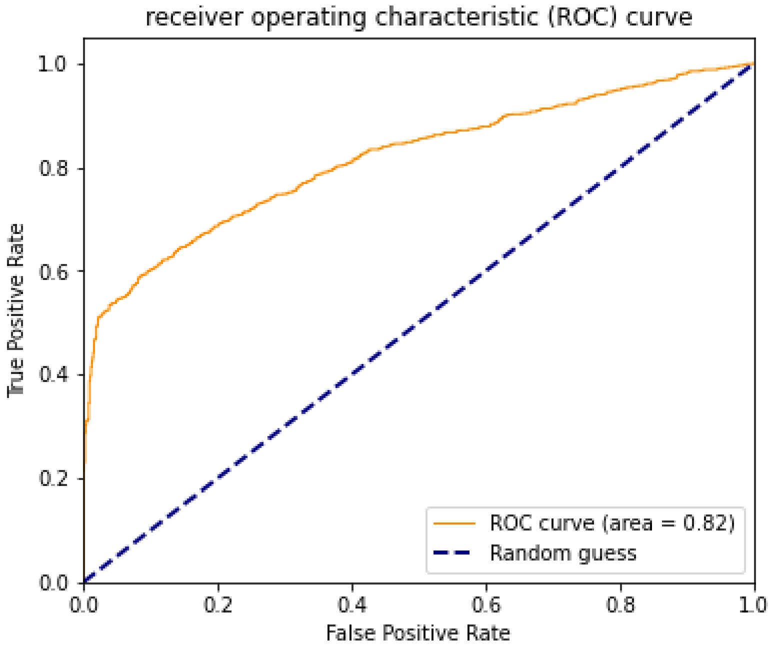

ROC_AUC Score of the model(AdaBoostClassifier): 0.8154527967240794

Confusion Matrix:
[[712 169]
 [358 641]]

Classification Report:

	precision	recall	f1-score	support
0.0	0.67	0.81	0.73	881
1.0	0.79	0.64	0.71	999
accuracy			0.72	1880
macro avg	0.73	0.72	0.72	1880
weighted avg	0.73	0.72	0.72	1880

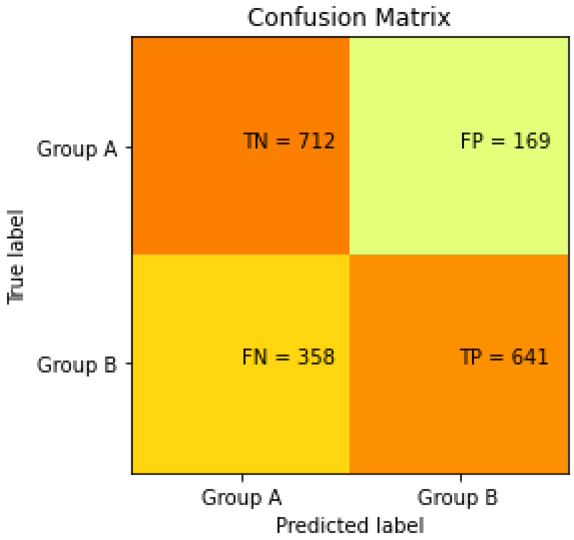

AUC Score : 0.7249070864280852

Accuracy Of the Model(SVC): 0.7196808510638298

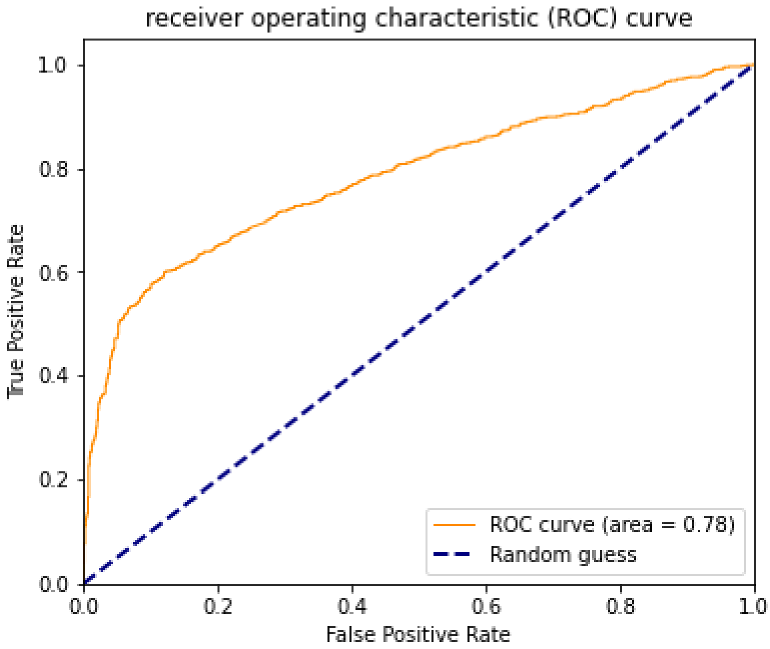

```
ROC_AUC Score of the model(SVC): 0.7838871788928543


Confusion Matrix:
[[793  88]
 [402 597]]

Classification Report:
              precision    recall  f1-score   support

         0.0       0.66      0.90      0.76       881
         1.0       0.87      0.60      0.71       999

    accuracy                           0.74      1880
   macro avg       0.77      0.75      0.74      1880
weighted avg       0.77      0.74      0.73      1880
```

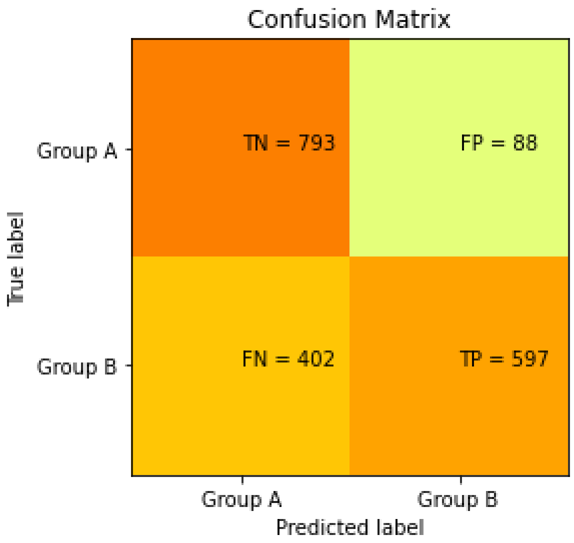


```
AUC Score : 0.7488555524877886

Accuracy Of the Model(MLPClassifier): 0.7393617021276596
```

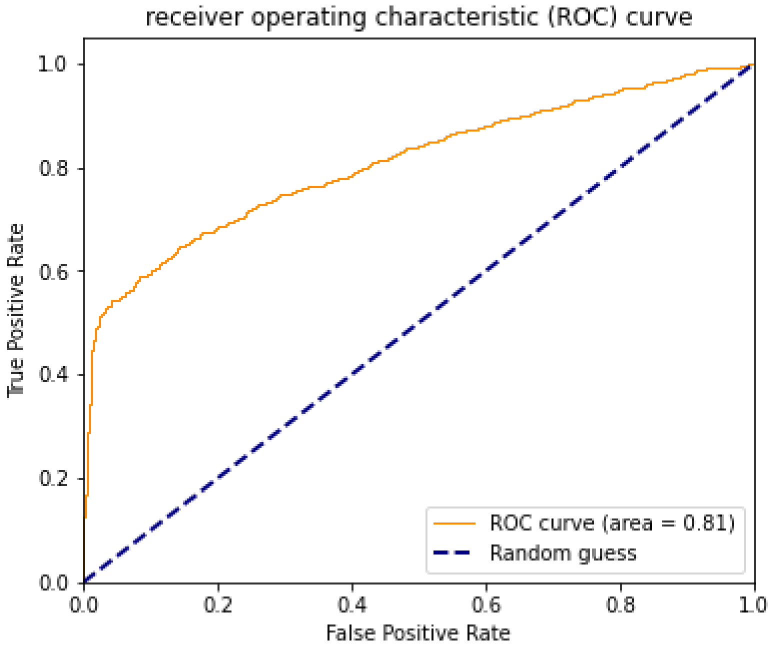

```
ROC_AUC Score of the model(MLPClassifier): 0.8098745737792276

[('XGBClassifier', 0.7468085106382979), ('GradientBoostingClassifier', 0.744148936
1702127), ('RandomForestClassifier', 0.7436170212765958), ('LogisticRegression',
0.7409574468085106), ('MLPClassifier', 0.7393617021276596), ('ExtraTreesClassifie
r', 0.7372340425531915), ('AdaBoostClassifier', 0.7340425531914894), ('DecisionTre
eClassifier', 0.7276595744680852), ('SVC', 0.7196808510638298), ('KNeighborsClassi
fier', 0.7069148936170213)]


sorted_total_auc:
 [('XGBClassifier', 0.8209764815894214), ('GradientBoostingClassifier', 0.81882222
74487882), ('AdaBoostClassifier', 0.8154527967240794), ('RandomForestClassifier',
0.8145114467475421), ('MLPClassifier', 0.8098745737792276), ('LogisticRegression',
0.8078100802277873), ('ExtraTreesClassifier', 0.8055234576233441), ('KNeighborsCla
ssifier', 0.7967093086275834), ('DecisionTreeClassifier', 0.7938909397479205), ('S
VC', 0.7838871788928543)]
```

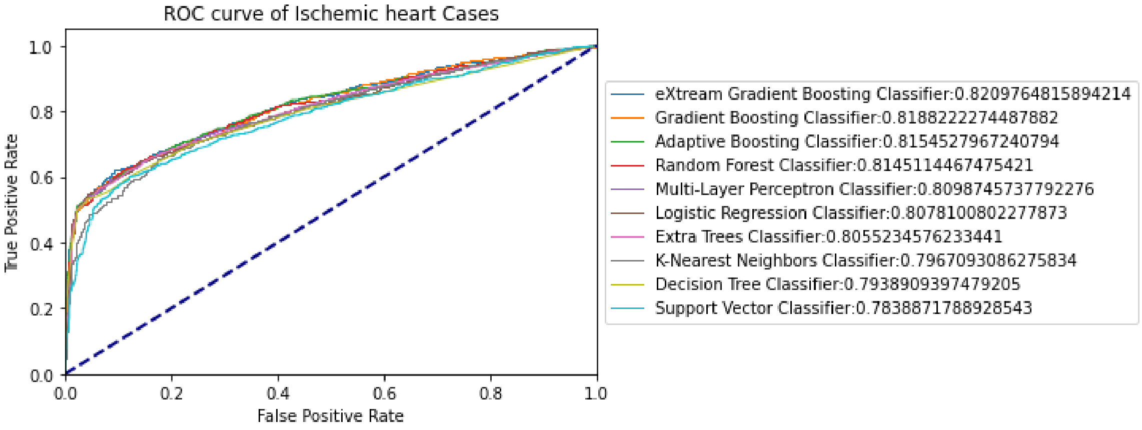

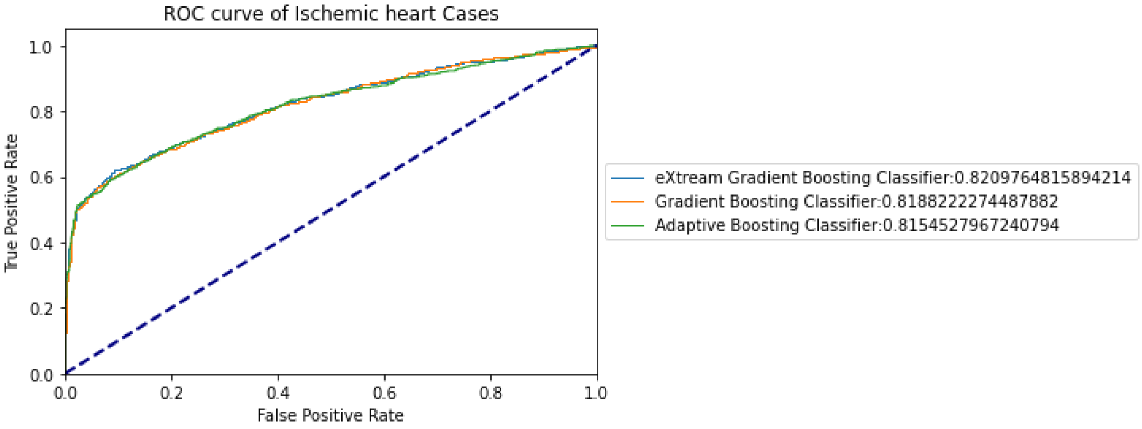

```
random state :   12696
9398
result_train_filename: data/train_test_data_ischemic heart_2013_20210519_141916.cs
v
total count : 9398, df_train count : 7518, df_test count : 1880
(7518, 38)
(1880, 38)
('Logistic Regression', LogisticRegression())
[2021-05-19 14:19:16.545386] Start parameter search for model 'Logistic Regressio
n'
[2021-05-19 14:19:16.870129] Finish parameter search for model 'Logistic Regressio
n' (time: 0 seconds)

=== train : best params ===
{'C': 100.0, 'penalty': 'l2', 'solver': 'lbfgs'}
=== train : best score : roc_auc ===
0.8062443948074851



('Random Forest', RandomForestClassifier())
[2021-05-19 14:19:16.871127] Start parameter search for model 'Random Forest'
[2021-05-19 14:19:21.269910] Finish parameter search for model 'Random Forest' (ti
me: 4 seconds)

=== train : best params ===
{'criterion': 'gini', 'max_depth': 10, 'max_features': 'auto', 'n_estimators': 10
0}
=== train : best score : roc_auc ===
0.8147837714600087



('Extreme Gradient Boosting)', XGBClassifier(base_score=None, booster=None, colsam
ple_bylevel=None,
              colsample_bynode=None, colsample_bytree=None, gamma=None,
              gpu_id=None, importance_type='gain', interaction_constraints=None,
              learning_rate=None, max_delta_step=None, max_depth=None,
              min_child_weight=None, missing=nan, monotone_constraints=None,
              n_estimators=100, n_jobs=None, num_parallel_tree=None,
              random_state=None, reg_alpha=None, reg_lambda=None,
              scale_pos_weight=None, subsample=None, tree_method=None,
              validate_parameters=None, verbosity=None))
[2021-05-19 14:19:21.270910] Start parameter search for model 'Extreme Gradient Bo
osting)'
[2021-05-19 14:19:47.444928] Finish parameter search for model 'Extreme Gradient B
oosting)' (time: 26 seconds)

=== train : best params ===
{'learning_rate': 0.05, 'max_depth': 5, 'n_estimators': 100, 'subsample': 0.8}
=== train : best score : roc_auc ===
0.8170679142570325



('k-Nearest Neighbours)', KNeighborsClassifier())
[2021-05-19 14:19:47.445925] Start parameter search for model 'k-Nearest Neighbour
s)'
[2021-05-19 14:19:55.371888] Finish parameter search for model 'k-Nearest Neighbou
rs)' (time: 7 seconds)

=== train : best params ===
{'n_neighbors': 60, 'weights': 'distance'}
=== train : best score : roc_auc ===
```

```
0.789432849616146


('Decision Tree', DecisionTreeClassifier())
[2021-05-19 14:19:55.372880] Start parameter search for model 'Decision Tree'
[2021-05-19 14:19:55.940363] Finish parameter search for model 'Decision Tree' (ti
me: 0 seconds)

=== train : best params ===
{'criterion': 'entropy', 'max_depth': 4}
=== train : best score : roc_auc ===
0.7928443889321923


('Extra Tree', ExtraTreesClassifier())
[2021-05-19 14:19:55.941392] Start parameter search for model 'Extra Tree'
[2021-05-19 14:19:58.908290] Finish parameter search for model 'Extra Tree' (time:
2 seconds)

=== train : best params ===
{'max_features': 'auto', 'n_estimators': 100}
=== train : best score : roc_auc ===
0.8099516660887991


('Gradient Boosting', GradientBoostingClassifier())
[2021-05-19 14:19:58.909288] Start parameter search for model 'Gradient Boosting'
[2021-05-19 14:20:14.567631] Finish parameter search for model 'Gradient Boosting'
(time: 15 seconds)

=== train : best params ===
{'learning_rate': 0.075, 'max_depth': 5, 'max_features': 4, 'n_estimators': 100}
=== train : best score : roc_auc ===
0.8168875463848753


('Adaptive Boosting', AdaBoostClassifier())
[2021-05-19 14:20:14.568597] Start parameter search for model 'Adaptive Boosting'
[2021-05-19 14:20:18.251798] Finish parameter search for model 'Adaptive Boosting'
(time: 3 seconds)

=== train : best params ===
{'learning_rate': 0.2, 'n_estimators': 100}
=== train : best score : roc_auc ===
0.8086528476178676


('Suppport Vector Classfier', SVC(probability=True))
[2021-05-19 14:20:18.251798] Start parameter search for model 'Suppport Vector Cla
ssfier'
[2021-05-19 14:24:12.586118] Finish parameter search for model 'Suppport Vector Cl
assfier' (time: 234 seconds)

=== train : best params ===
{'C': 1, 'gamma': 0.1, 'kernel': 'rbf'}
=== train : best score : roc_auc ===
0.7900376208187514


('Neural Network', MLPClassifier())
[2021-05-19 14:24:12.586118] Start parameter search for model 'Neural Network'
```

```
[2021-05-19 14:26:27.144543] Finish parameter search for model 'Neural Network' (t
ime: 134 seconds)

=== train : best params ===
{'activation': 'logistic', 'alpha': 0.05, 'hidden_layer_sizes': (50,), 'learning_r
ate': 'adaptive'}
=== train : best score : roc_auc ===
0.812313364273908



Confusion Matrix:
[[813  68]
 [429 570]]

Classification Report:
              precision    recall  f1-score   support

         0.0       0.65      0.92      0.77       881
         1.0       0.89      0.57      0.70       999

    accuracy                           0.74      1880
   macro avg       0.77      0.75      0.73      1880
weighted avg       0.78      0.74      0.73      1880
```

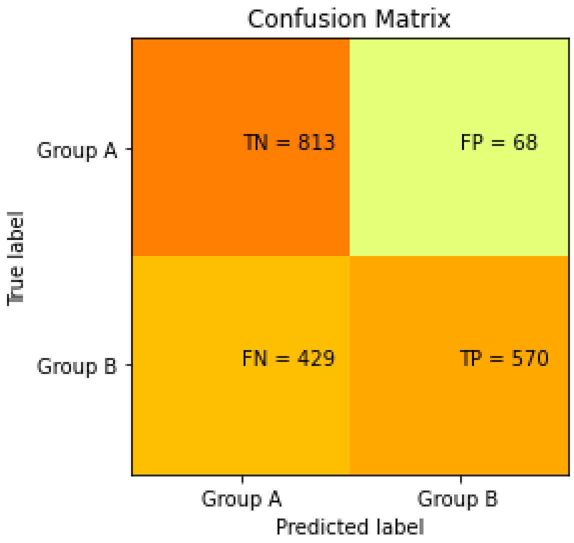


```
AUC Score : 0.7466927767722319

Accuracy Of the Model(LogisticRegression): 0.7356382978723405
```

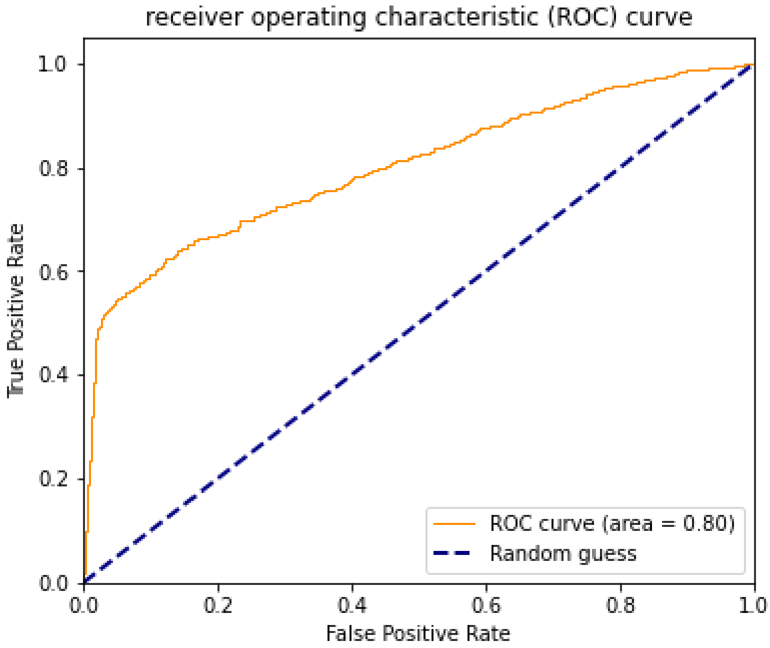


```
ROC_AUC Score of the model(LogisticRegression): 0.8026516868741613


Confusion Matrix:
[[789  92]
 [406 593]]

Classification Report:
              precision    recall  f1-score   support

         0.0       0.66      0.90      0.76       881
         1.0       0.87      0.59      0.70       999

    accuracy                           0.74      1880
   macro avg       0.76      0.74      0.73      1880
weighted avg       0.77      0.74      0.73      1880
```

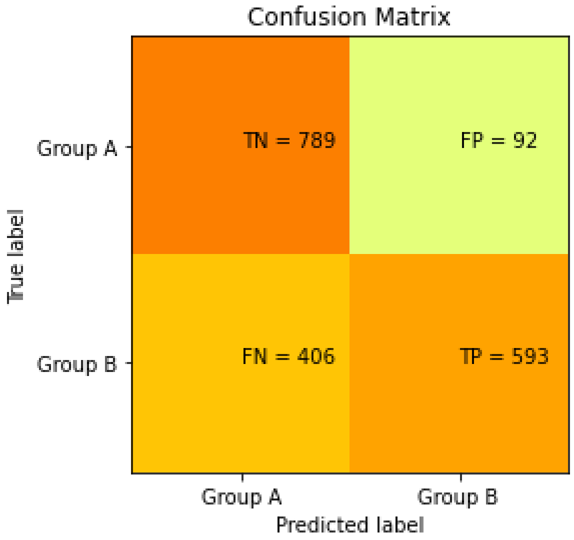


```
AUC Score : 0.7445834029261953

Accuracy Of the Model(RandomForestClassifier): 0.7351063829787234
```

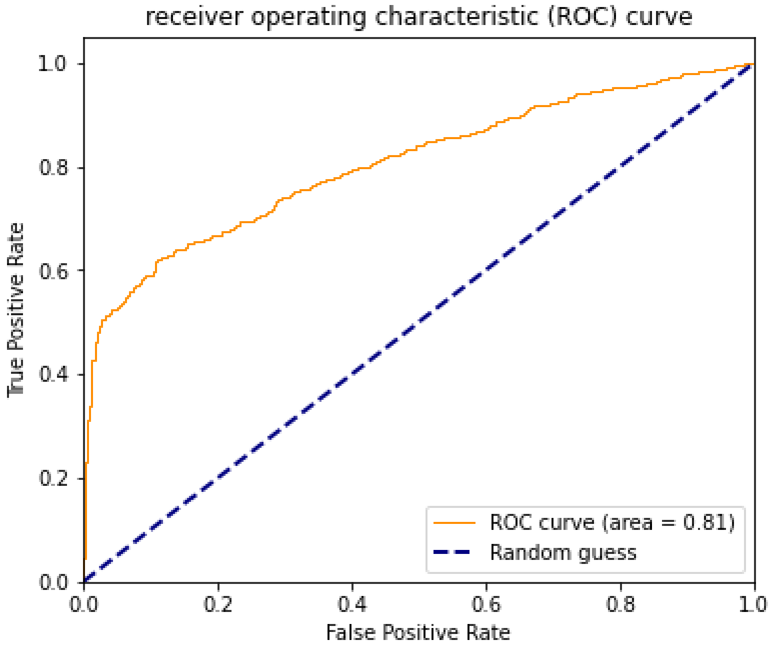

```
ROC_AUC Score of the model(RandomForestClassifier): 0.8075214828903818


Confusion Matrix:
[[788  93]
 [403 596]]

Classification Report:
              precision    recall  f1-score   support

         0.0       0.66      0.89      0.76       881
         1.0       0.87      0.60      0.71       999

    accuracy                           0.74      1880
   macro avg       0.76      0.75      0.73      1880
weighted avg       0.77      0.74      0.73      1880
```

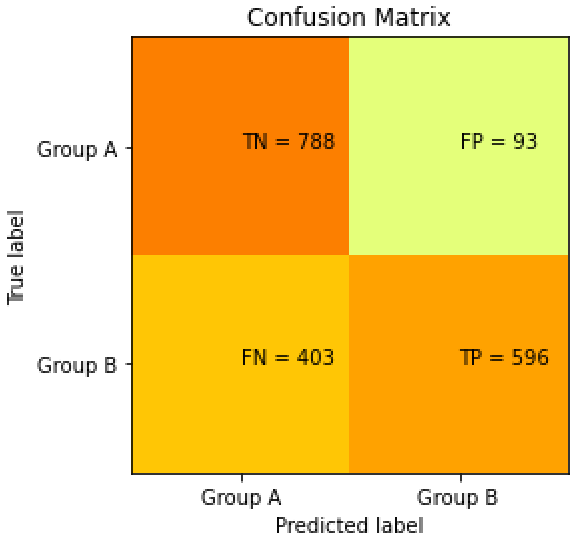


```
AUC Score : 0.7455173675377988

Accuracy Of the Model(XGBClassifier): 0.7361702127659574
```

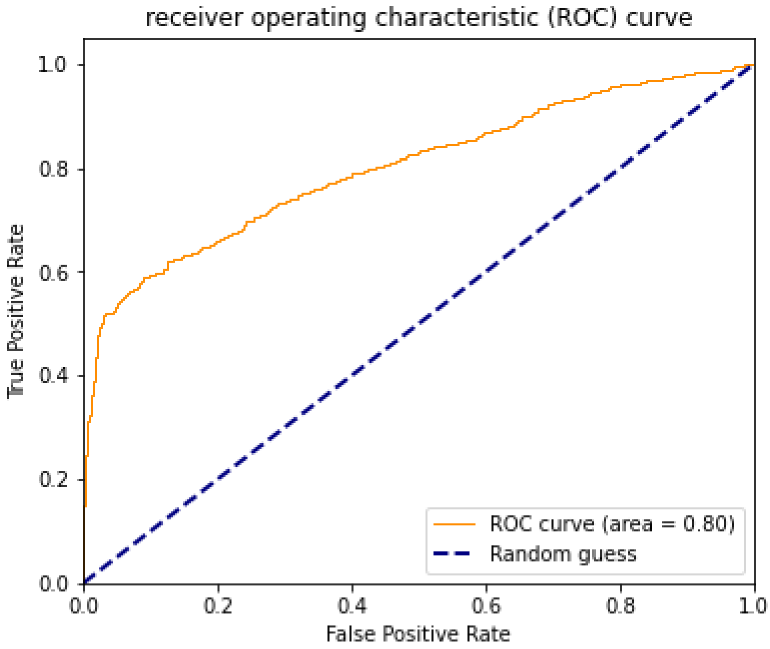

ROC_AUC Score of the model(XGBClassifier): 0.8034447614470317

Confusion Matrix:
[[785 96]
 [449 550]]

Classification Report:

	precision	recall	f1-score	support
0.0	0.64	0.89	0.74	881
1.0	0.85	0.55	0.67	999
accuracy			0.71	1880
macro avg	0.74	0.72	0.71	1880
weighted avg	0.75	0.71	0.70	1880

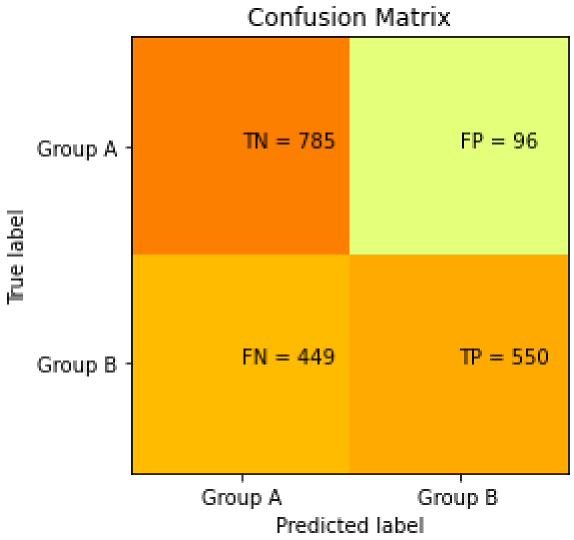

AUC Score : 0.7207917338450823

Accuracy Of the Model(KNeighborsClassifier): 0.7101063829787234

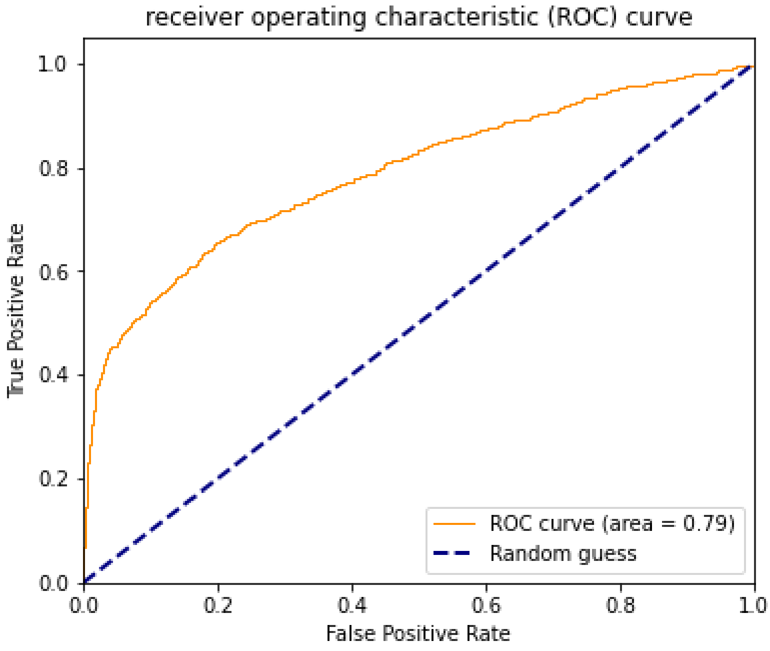

```
ROC_AUC Score of the model(KNeighborsClassifier): 0.7889830806970421


Confusion Matrix:
[[817  64]
 [450 549]]

Classification Report:
              precision    recall  f1-score   support

         0.0       0.64      0.93      0.76       881
         1.0       0.90      0.55      0.68       999

    accuracy                           0.73      1880
   macro avg       0.77      0.74      0.72      1880
weighted avg       0.78      0.73      0.72      1880
```

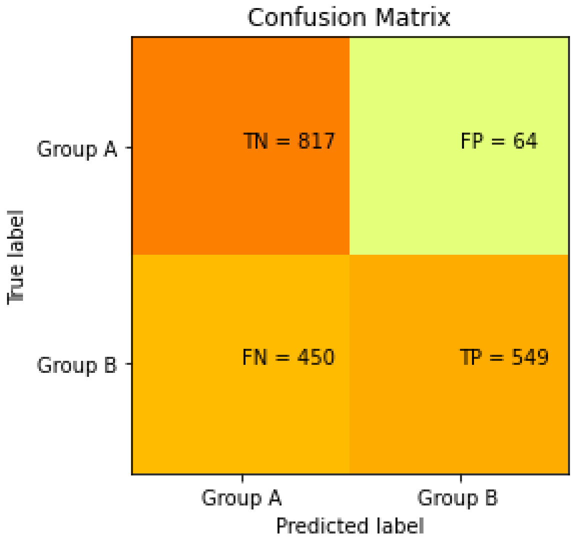


```
AUC Score : 0.7384524138213129

Accuracy Of the Model(DecisionTreeClassifier): 0.7265957446808511
```

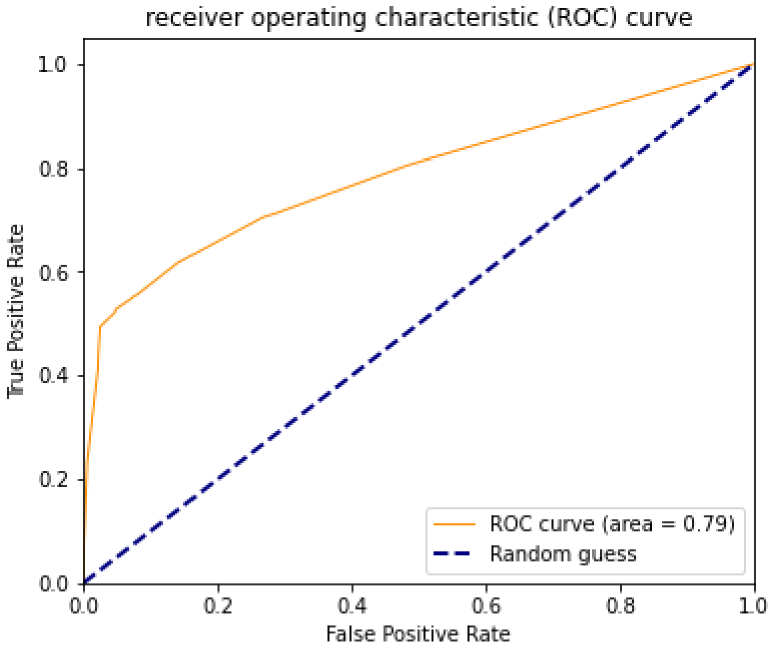

ROC_AUC Score of the model(DecisionTreeClassifier): 0.785244381725653

Confusion Matrix:
[[780 101]
 [415 584]]

Classification Report:

	precision	recall	f1-score	support
0.0	0.65	0.89	0.75	881
1.0	0.85	0.58	0.69	999
accuracy			0.73	1880
macro avg	0.75	0.73	0.72	1880
weighted avg	0.76	0.73	0.72	1880

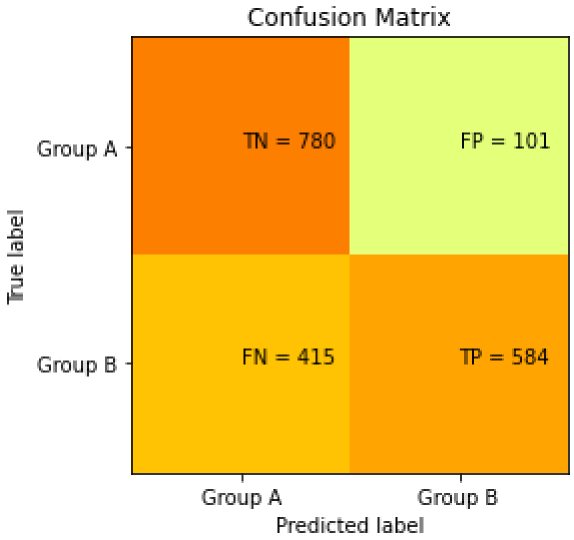

AUC Score : 0.7349710664126101

Accuracy Of the Model(ExtraTreesClassifier): 0.725531914893617

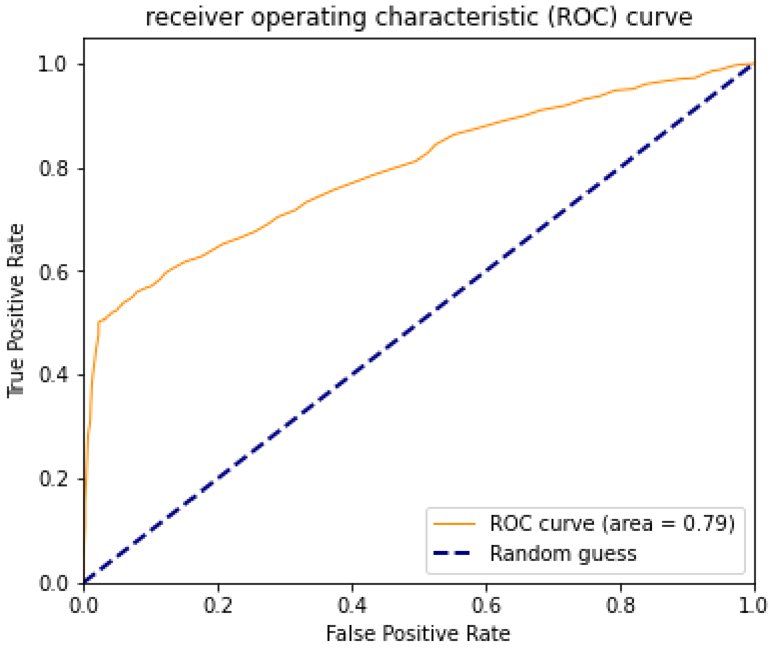

```
ROC_AUC Score of the model(ExtraTreesClassifier): 0.794544260492047


Confusion Matrix:
[[781 100]
 [399 600]]

Classification Report:
              precision    recall  f1-score   support

         0.0       0.66      0.89      0.76       881
         1.0       0.86      0.60      0.71       999

    accuracy                           0.73      1880
   macro avg       0.76      0.74      0.73      1880
weighted avg       0.77      0.73      0.73      1880
```

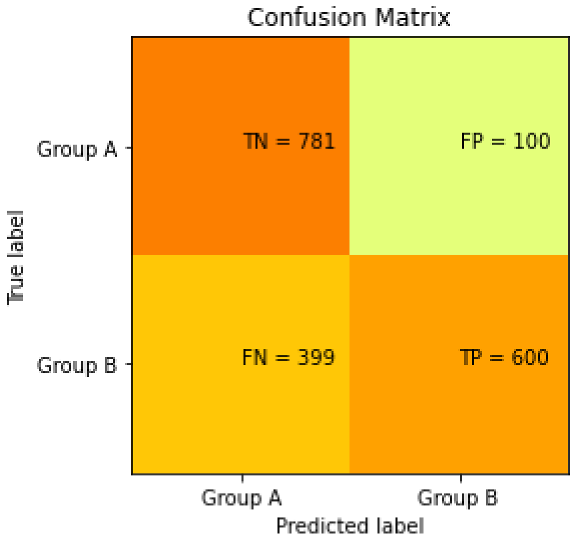


```
AUC Score : 0.7435466113105159

Accuracy Of the Model(GradientBoostingClassifier): 0.7345744680851064
```

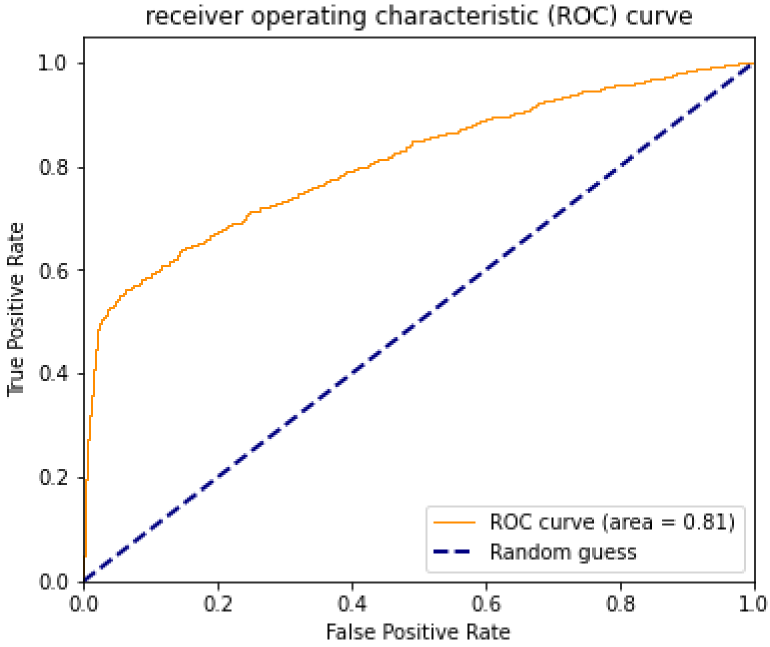

ROC_AUC Score of the model(GradientBoostingClassifier): 0.8098791186191868

Confusion Matrix:
[[813 68]
 [427 572]]

Classification Report:

	precision	recall	f1-score	support
0.0	0.66	0.92	0.77	881
1.0	0.89	0.57	0.70	999
accuracy			0.74	1880
macro avg	0.77	0.75	0.73	1880
weighted avg	0.78	0.74	0.73	1880

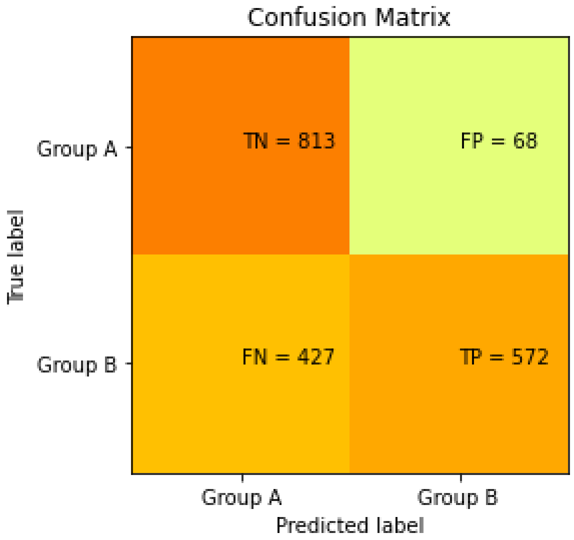

AUC Score : 0.7476937777732329

Accuracy Of the Model(AdaBoostClassifier): 0.7367021276595744

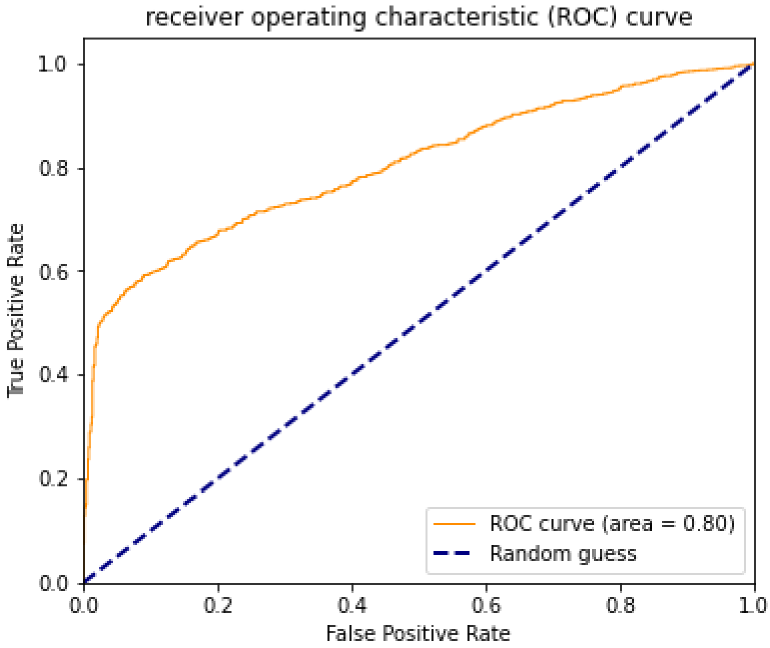

ROC_AUC Score of the model(AdaBoostClassifier): 0.802743151778339

Confusion Matrix:
[[683 198]
 [348 651]]

Classification Report:

	precision	recall	f1-score	support
0.0	0.66	0.78	0.71	881
1.0	0.77	0.65	0.70	999
accuracy			0.71	1880
macro avg	0.71	0.71	0.71	1880
weighted avg	0.72	0.71	0.71	1880

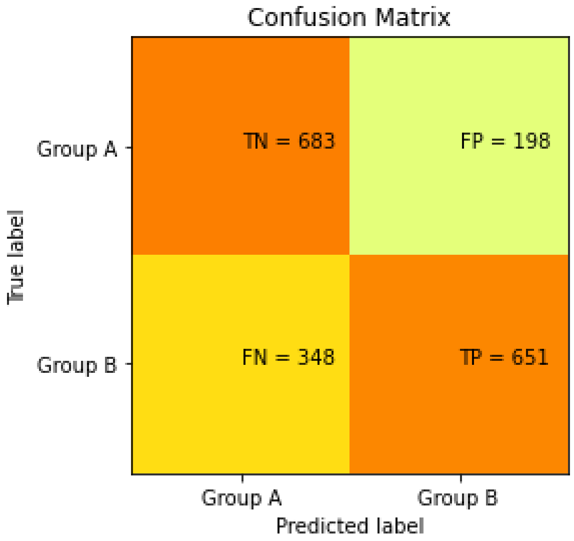

AUC Score : 0.7134535216260528

Accuracy Of the Model(SVC): 0.7095744680851064

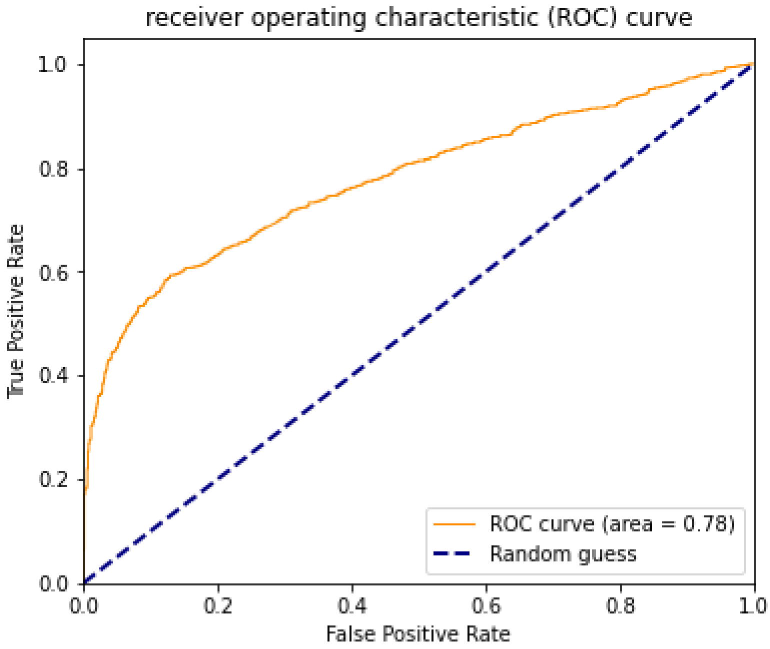

ROC_AUC Score of the model(SVC): 0.7773766956513835

Confusion Matrix:
[[774 107]
 [391 608]]

Classification Report:

	precision	recall	f1-score	support
0.0	0.66	0.88	0.76	881
1.0	0.85	0.61	0.71	999
accuracy			0.74	1880
macro avg	0.76	0.74	0.73	1880
weighted avg	0.76	0.74	0.73	1880

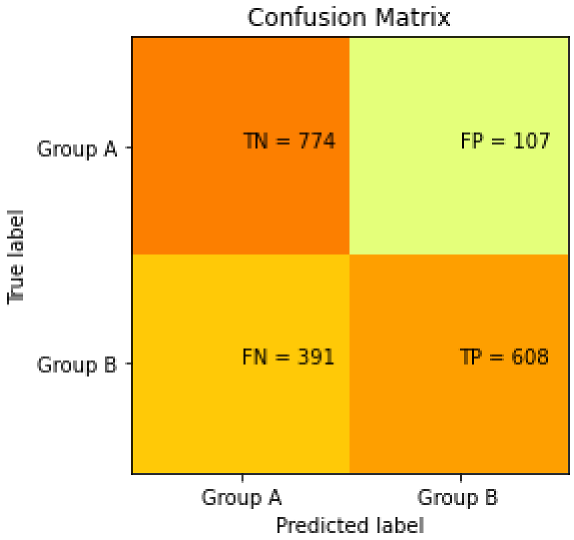

AUC Score : 0.7435778570852352

Accuracy Of the Model(MLPClassifier): 0.7351063829787234

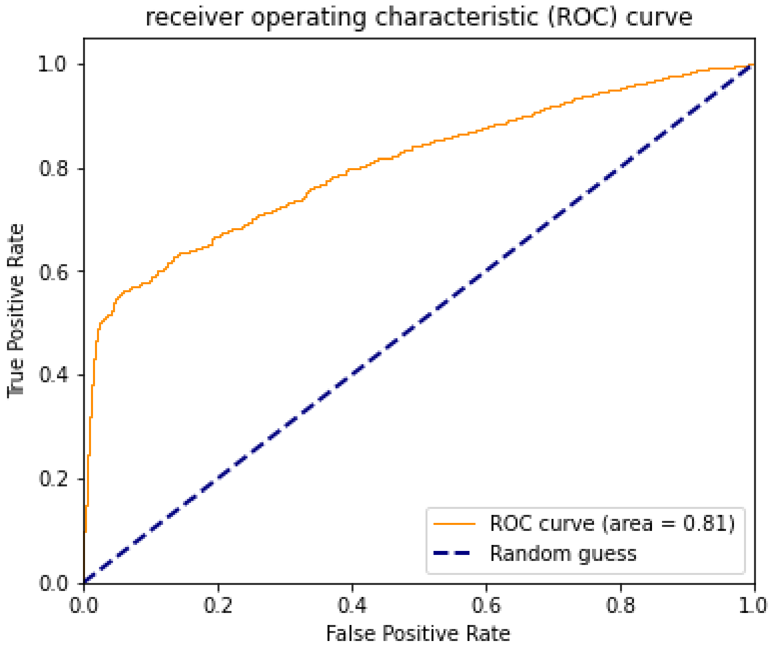

```
ROC_AUC Score of the model(MLPClassifier): 0.8062648346416792

[('AdaBoostClassifier', 0.7367021276595744), ('XGBClassifier', 0.736170212765957
4), ('LogisticRegression', 0.7356382978723405), ('RandomForestClassifier', 0.73510
63829787234), ('MLPClassifier', 0.7351063829787234), ('GradientBoostingClassifie
r', 0.7345744680851064), ('DecisionTreeClassifier', 0.7265957446808511), ('ExtraTr
eesClassifier', 0.725531914893617), ('KNeighborsClassifier', 0.7101063829787234),
('SVC', 0.7095744680851064)]


sorted_total_auc:
 [('GradientBoostingClassifier', 0.8098791186191868), ('RandomForestClassifier',
0.8075214828903818), ('MLPClassifier', 0.8062648346416792), ('XGBClassifier', 0.80
34447614470317), ('AdaBoostClassifier', 0.802743151778339), ('LogisticRegression',
0.8026516868741613), ('ExtraTreesClassifier', 0.794544260492047), ('KNeighborsClas
sifier', 0.7889830806970421), ('DecisionTreeClassifier', 0.785244381725653), ('SV
C', 0.7773766956513835)]
```

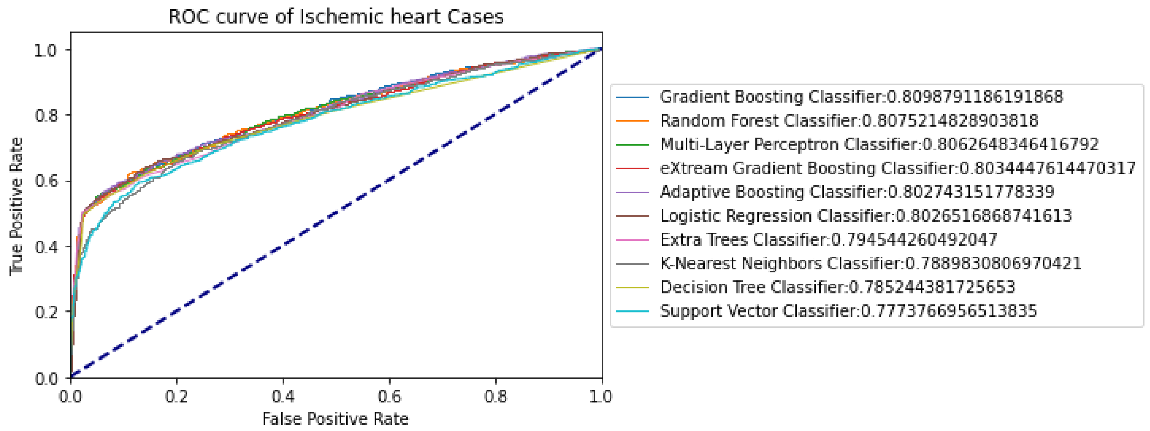

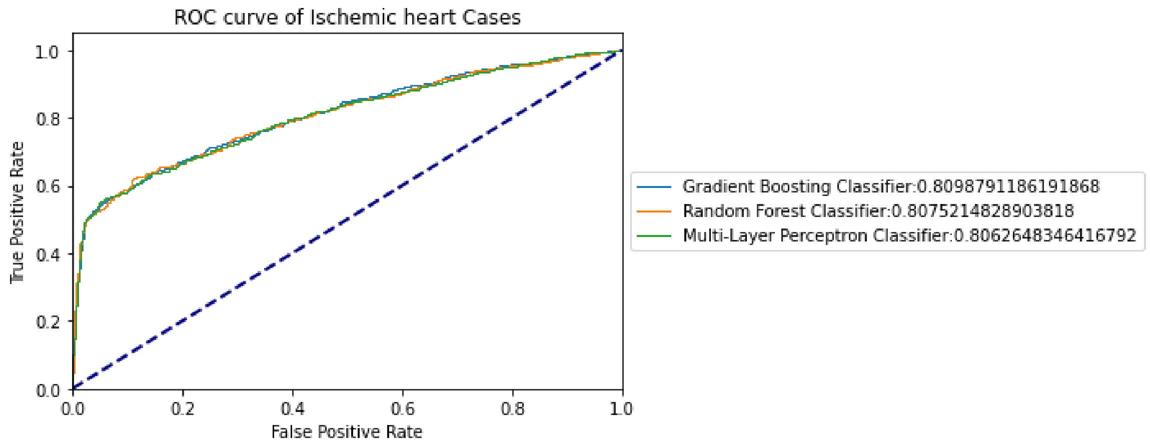

```
random state :   12723
9398
result_train_filename: data/train_test_data_ischemic heart_2013_20210519_142634.cs
v
total count : 9398, df_train count : 7518, df_test count : 1880
(7518, 38)
(1880, 38)
('Logistic Regression', LogisticRegression())
[2021-05-19 14:26:34.969412] Start parameter search for model 'Logistic Regressio
n'
[2021-05-19 14:26:35.350025] Finish parameter search for model 'Logistic Regressio
n' (time: 0 seconds)

=== train : best params ===
{'C': 1000.0, 'penalty': 'l2', 'solver': 'lbfgs'}
=== train : best score : roc_auc ===
0.8055931147549704


('Random Forest', RandomForestClassifier())
[2021-05-19 14:26:35.352017] Start parameter search for model 'Random Forest'
[2021-05-19 14:26:40.301772] Finish parameter search for model 'Random Forest' (ti
me: 4 seconds)

=== train : best params ===
{'criterion': 'gini', 'max_depth': 10, 'max_features': 'auto', 'n_estimators': 10
0}
=== train : best score : roc_auc ===
0.811609130017641


('Extreme Gradient Boosting)', XGBClassifier(base_score=None, booster=None, colsam
ple_bylevel=None,
              colsample_bynode=None, colsample_bytree=None, gamma=None,
              gpu_id=None, importance_type='gain', interaction_constraints=None,
              learning_rate=None, max_delta_step=None, max_depth=None,
              min_child_weight=None, missing=nan, monotone_constraints=None,
              n_estimators=100, n_jobs=None, num_parallel_tree=None,
              random_state=None, reg_alpha=None, reg_lambda=None,
              scale_pos_weight=None, subsample=None, tree_method=None,
              validate_parameters=None, verbosity=None))
[2021-05-19 14:26:40.302770] Start parameter search for model 'Extreme Gradient Bo
osting)'
[2021-05-19 14:27:09.072555] Finish parameter search for model 'Extreme Gradient B
oosting)' (time: 28 seconds)

=== train : best params ===
{'learning_rate': 0.075, 'max_depth': 5, 'n_estimators': 40, 'subsample': 0.8}
=== train : best score : roc_auc ===
0.8101958900409867


('k-Nearest Neighbours)', KNeighborsClassifier())
[2021-05-19 14:27:09.074549] Start parameter search for model 'k-Nearest Neighbour
s)'
[2021-05-19 14:27:17.301190] Finish parameter search for model 'k-Nearest Neighbou
rs)' (time: 8 seconds)

=== train : best params ===
{'n_neighbors': 60, 'weights': 'distance'}
=== train : best score : roc_auc ===
```

```
0.7874758408042137


('Decision Tree', DecisionTreeClassifier())
[2021-05-19 14:27:17.302188] Start parameter search for model 'Decision Tree'
[2021-05-19 14:27:17.933049] Finish parameter search for model 'Decision Tree' (ti
me: 0 seconds)

=== train : best params ===
{'criterion': 'gini', 'max_depth': 4}
=== train : best score : roc_auc ===
0.7909312950686012


('Extra Tree', ExtraTreesClassifier())
[2021-05-19 14:27:17.933049] Start parameter search for model 'Extra Tree'
[2021-05-19 14:27:21.119318] Finish parameter search for model 'Extra Tree' (time:
3 seconds)

=== train : best params ===
{'max_features': 'sqrt', 'n_estimators': 100}
=== train : best score : roc_auc ===
0.803093309962263


('Gradient Boosting', GradientBoostingClassifier())
[2021-05-19 14:27:21.119318] Start parameter search for model 'Gradient Boosting'
[2021-05-19 14:27:37.320719] Finish parameter search for model 'Gradient Boosting'
(time: 16 seconds)

=== train : best params ===
{'learning_rate': 0.075, 'max_depth': 5, 'max_features': 4, 'n_estimators': 100}
=== train : best score : roc_auc ===
0.8113319729237578


('Adaptive Boosting', AdaBoostClassifier())
[2021-05-19 14:27:37.320719] Start parameter search for model 'Adaptive Boosting'
[2021-05-19 14:27:40.658153] Finish parameter search for model 'Adaptive Boosting'
(time: 3 seconds)

=== train : best params ===
{'learning_rate': 0.2, 'n_estimators': 50}
=== train : best score : roc_auc ===
0.8048197012551368


('Suppport Vector Classfier', SVC(probability=True))
[2021-05-19 14:27:40.659153] Start parameter search for model 'Suppport Vector Cla
ssfier'
[2021-05-19 14:31:50.117616] Finish parameter search for model 'Suppport Vector Cl
assfier' (time: 249 seconds)

=== train : best params ===
{'C': 1, 'gamma': 0.1, 'kernel': 'rbf'}
=== train : best score : roc_auc ===
0.7831904063114021


('Neural Network', MLPClassifier())
[2021-05-19 14:31:50.117616] Start parameter search for model 'Neural Network'
```

```
[2021-05-19 14:34:23.028373] Finish parameter search for model 'Neural Network' (t
ime: 152 seconds)

=== train : best params ===
{'activation': 'logistic', 'alpha': 0.05, 'hidden_layer_sizes': (50,), 'learning_r
ate': 'constant'}
=== train : best score : roc_auc ===
0.8099697330083



Confusion Matrix:
[[804  77]
 [425 574]]

Classification Report:
              precision    recall  f1-score   support

         0.0       0.65      0.91      0.76       881
         1.0       0.88      0.57      0.70       999

    accuracy                           0.73      1880
   macro avg       0.77      0.74      0.73      1880
weighted avg       0.78      0.73      0.73      1880
```

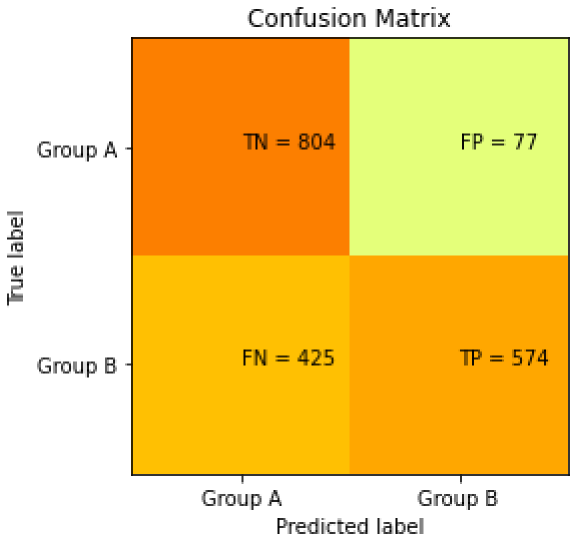


```
AUC Score : 0.7435869467651535

Accuracy Of the Model(LogisticRegression): 0.7329787234042553
```

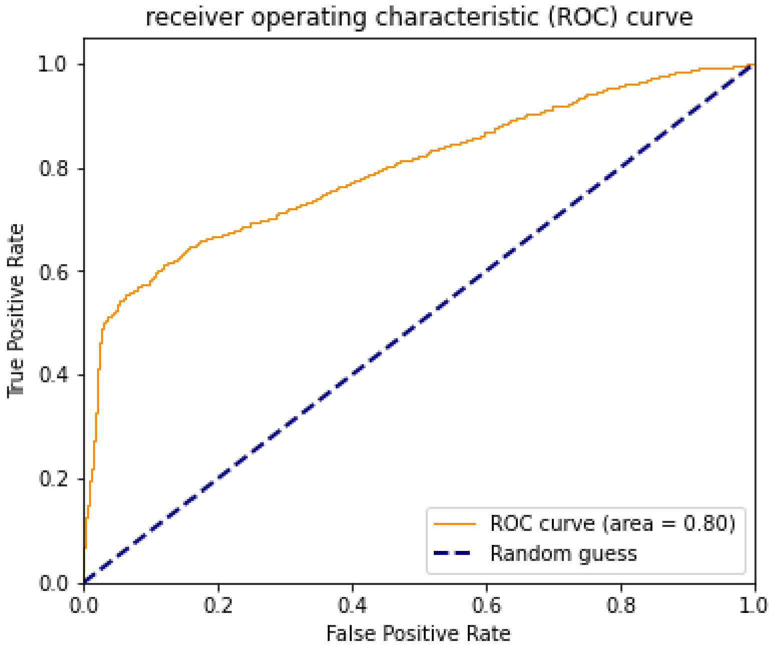


```
ROC_AUC Score of the model(LogisticRegression): 0.7972240117529563


Confusion Matrix:
[[785  96]
 [401 598]]

Classification Report:
              precision    recall  f1-score   support

         0.0       0.66      0.89      0.76       881
         1.0       0.86      0.60      0.71       999

    accuracy                           0.74      1880
   macro avg       0.76      0.74      0.73      1880
weighted avg       0.77      0.74      0.73      1880
```

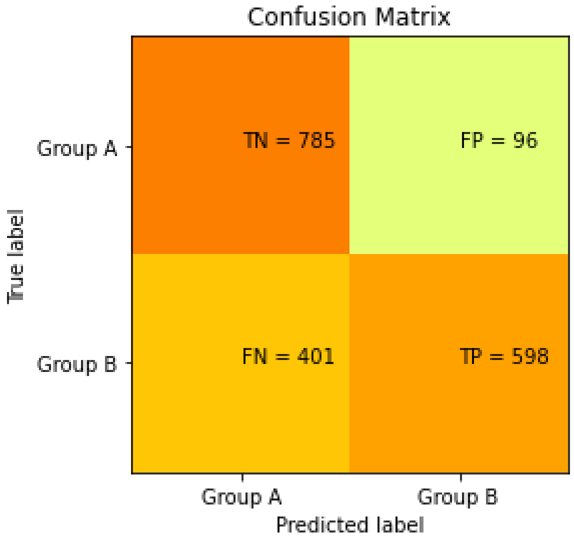

AUC Score : 0.7448157578691064

Accuracy Of the Model(RandomForestClassifier): 0.7356382978723405

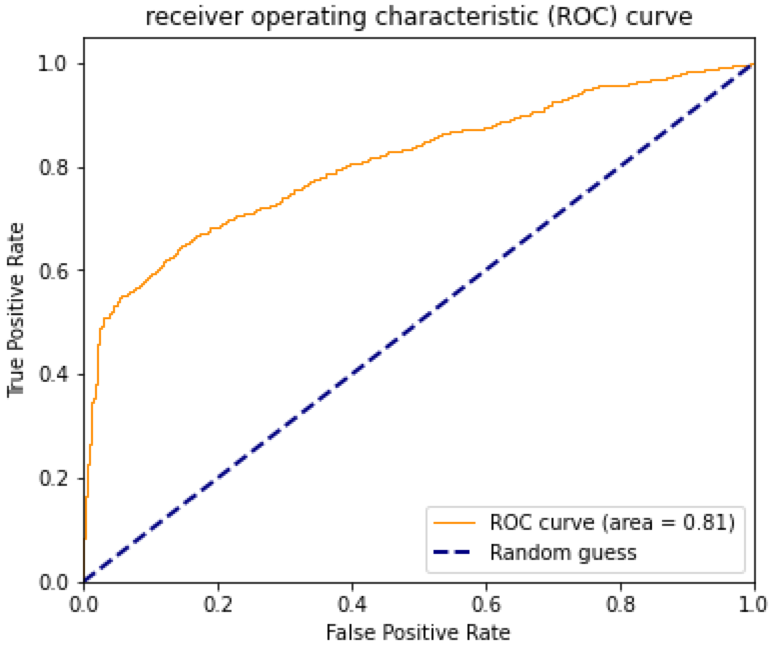

```
ROC_AUC Score of the model(RandomForestClassifier): 0.8105881136528128


Confusion Matrix:
[[803  78]
 [406 593]]

Classification Report:
              precision    recall  f1-score   support

         0.0       0.66      0.91      0.77       881
         1.0       0.88      0.59      0.71       999

    accuracy                           0.74      1880
   macro avg       0.77      0.75      0.74      1880
weighted avg       0.78      0.74      0.74      1880
```

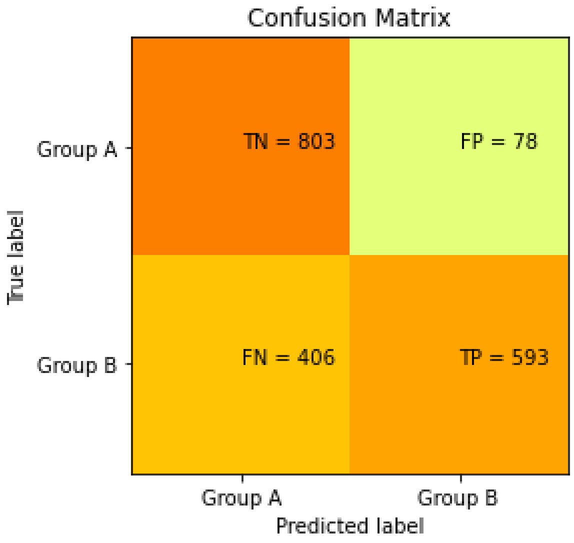


```
AUC Score : 0.7525289193847651

Accuracy Of the Model(XGBClassifier): 0.7425531914893617
```

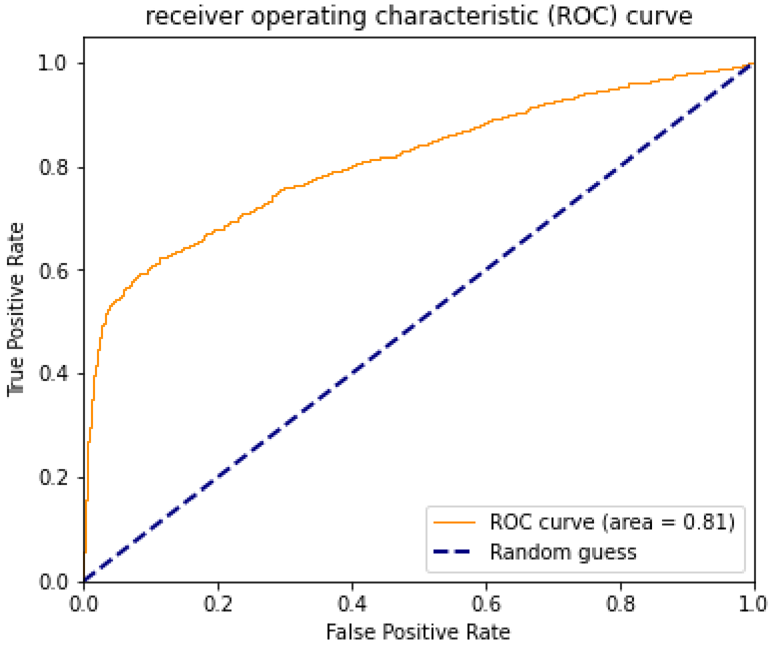

```
ROC_AUC Score of the model(XGBClassifier): 0.8113221053062143


Confusion Matrix:
[[787  94]
 [468 531]]

Classification Report:
              precision    recall  f1-score   support

         0.0       0.63      0.89      0.74       881
         1.0       0.85      0.53      0.65       999

    accuracy                           0.70      1880
   macro avg       0.74      0.71      0.70      1880
weighted avg       0.75      0.70      0.69      1880
```

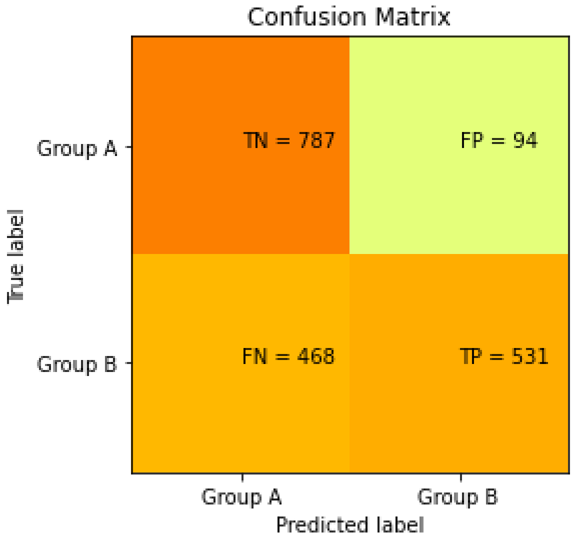


```
AUC Score : 0.7124172981153685

Accuracy Of the Model(KNeighborsClassifier): 0.701063829787234
```

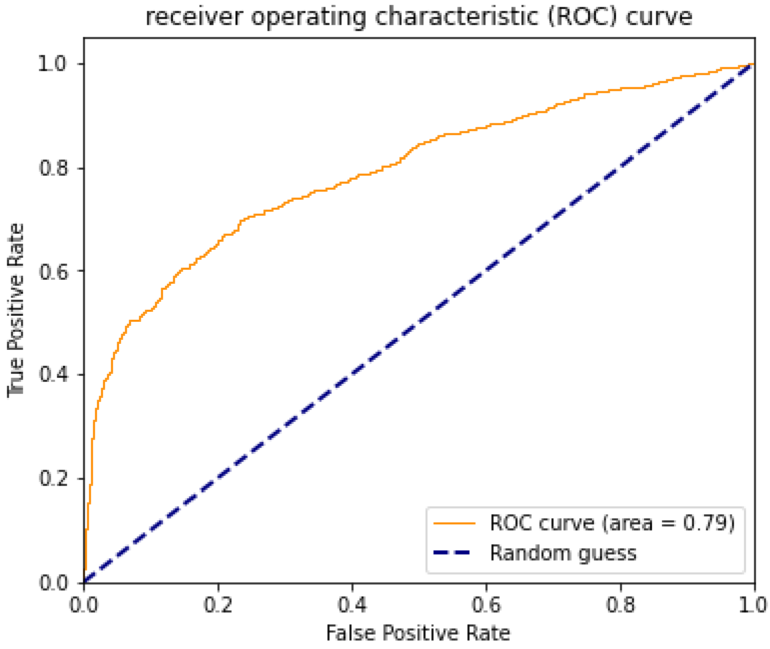

ROC_AUC Score of the model(KNeighborsClassifier): 0.7919201835206375

```
Confusion Matrix:
[[787  94]
 [410 589]]

Classification Report:
              precision    recall  f1-score   support

         0.0       0.66      0.89      0.76       881
         1.0       0.86      0.59      0.70       999

    accuracy                           0.73      1880
   macro avg       0.76      0.74      0.73      1880
weighted avg       0.77      0.73      0.73      1880
```

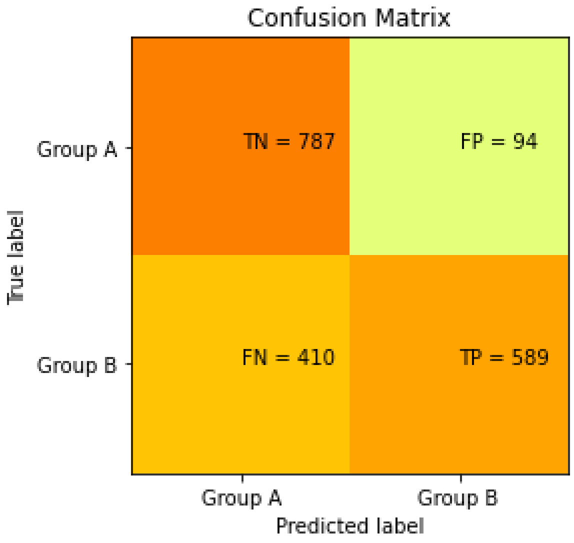

AUC Score : 0.7414463271443976

Accuracy Of the Model(DecisionTreeClassifier): 0.7319148936170212

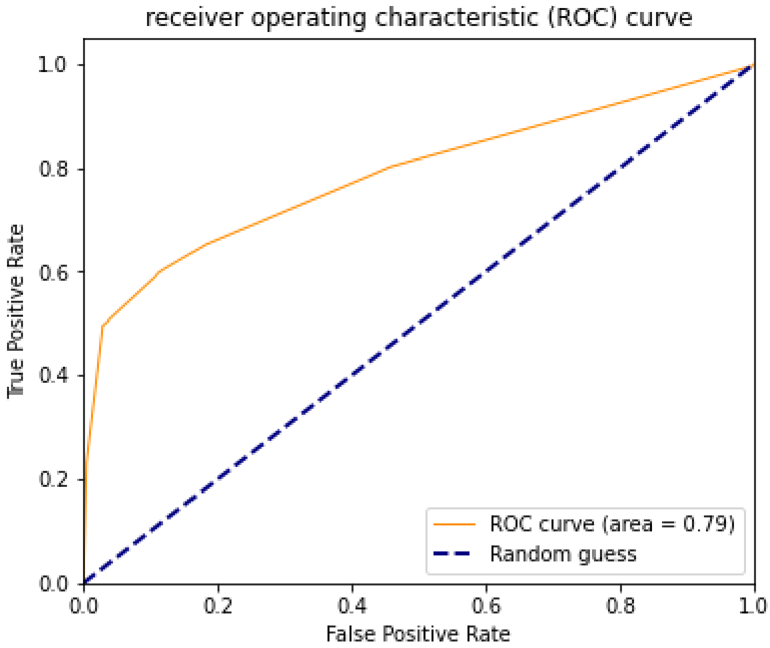

ROC_AUC Score of the model(DecisionTreeClassifier): 0.7867527004870931

```
Confusion Matrix:
[[787  94]
 [413 586]]

Classification Report:
              precision    recall  f1-score   support

         0.0       0.66      0.89      0.76       881
         1.0       0.86      0.59      0.70       999

    accuracy                           0.73      1880
   macro avg       0.76      0.74      0.73      1880
weighted avg       0.77      0.73      0.73      1880
```

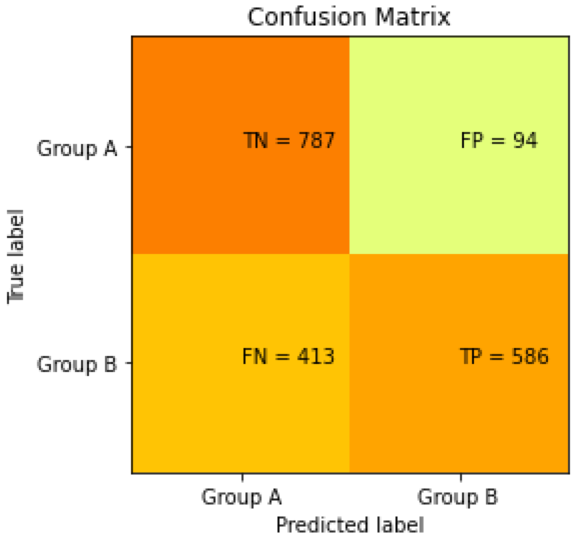

AUC Score : 0.7399448256428 96

Accuracy Of the Model(ExtraTreesClassifier): 0.7303191489361702

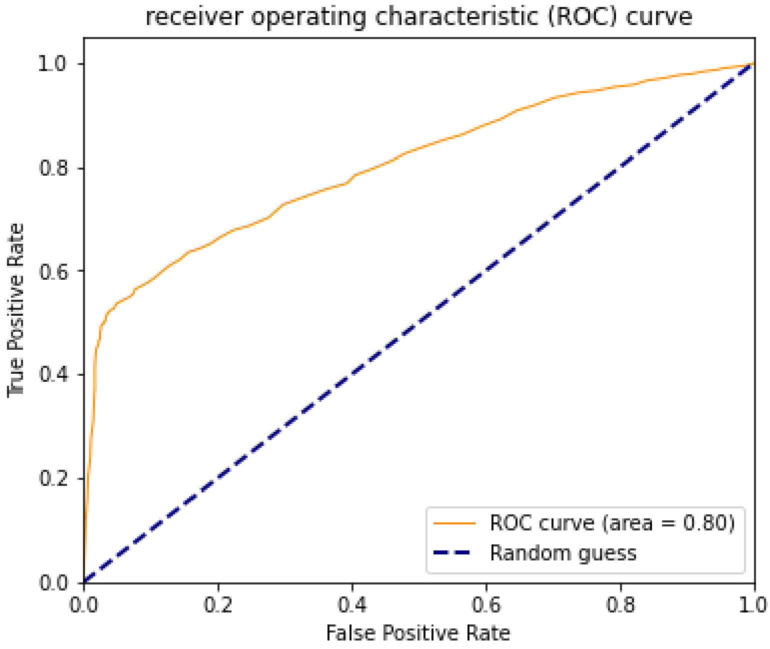

```
ROC_AUC Score of the model(ExtraTreesClassifier): 0.8018728149261632


Confusion Matrix:
[[790  91]
 [386 613]]

Classification Report:
              precision    recall  f1-score   support

         0.0       0.67      0.90      0.77       881
         1.0       0.87      0.61      0.72       999

    accuracy                           0.75      1880
   macro avg       0.77      0.76      0.74      1880
weighted avg       0.78      0.75      0.74      1880
```

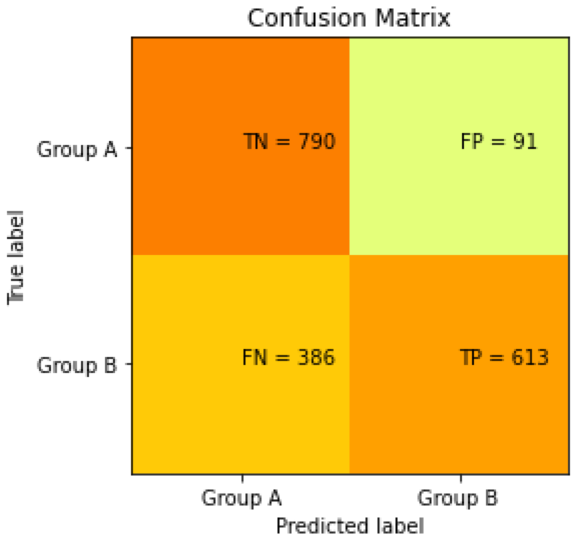


```
AUC Score : 0.7551609498261O3

Accuracy Of the Model(GradientBoostingClassifier): 0.7462765957446809
```

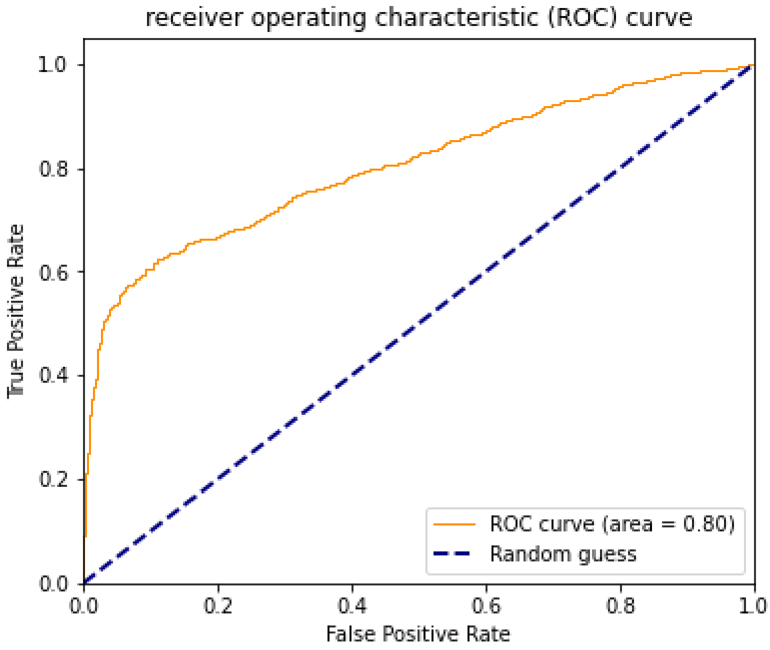

```
ROC_AUC Score of the model(GradientBoostingClassifier): 0.8048309376345699


Confusion Matrix:
[[828  53]
 [455 544]]

Classification Report:
              precision    recall  f1-score   support

         0.0       0.65      0.94      0.77       881
         1.0       0.91      0.54      0.68       999

    accuracy                           0.73      1880
   macro avg       0.78      0.74      0.72      1880
weighted avg       0.79      0.73      0.72      1880
```

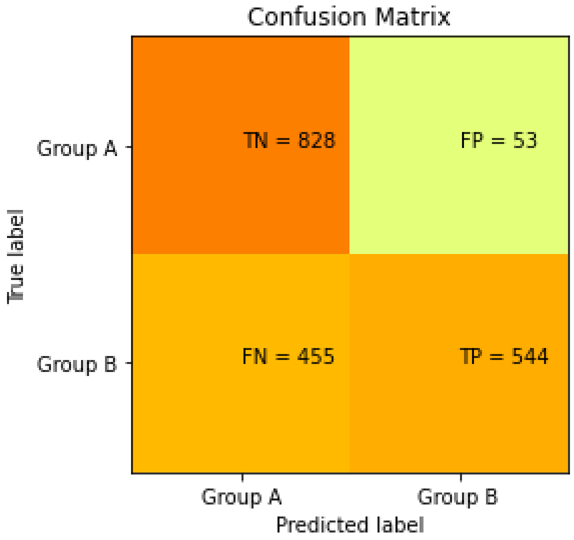


```
AUC Score : 0.7421928171076866

Accuracy Of the Model(AdaBoostClassifier): 0.7297872340425532
```

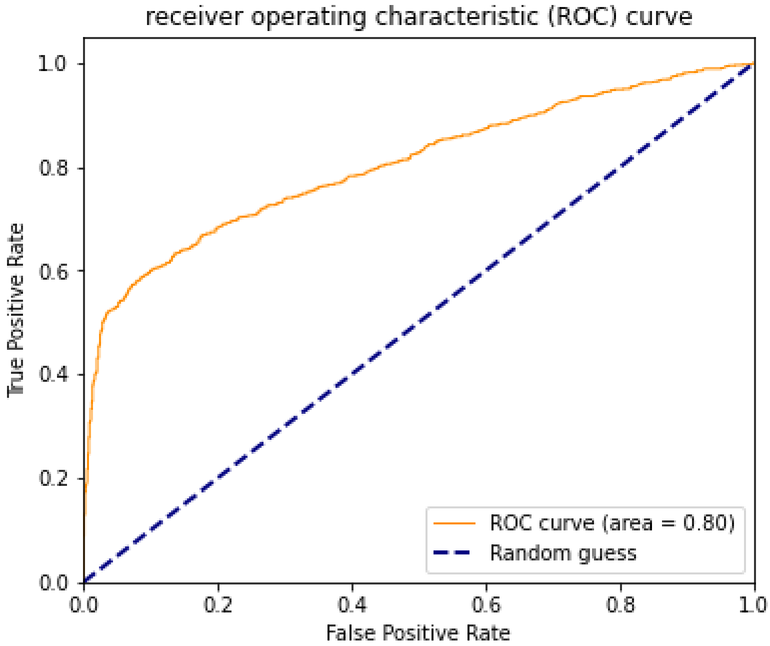

```
ROC_AUC Score of the model(AdaBoostClassifier): 0.8037526743542635


Confusion Matrix:
[[719 162]
 [344 655]]

Classification Report:
              precision    recall  f1-score   support

         0.0       0.68      0.82      0.74       881
         1.0       0.80      0.66      0.72       999

    accuracy                           0.73      1880
   macro avg       0.74      0.74      0.73      1880
weighted avg       0.74      0.73      0.73      1880
```

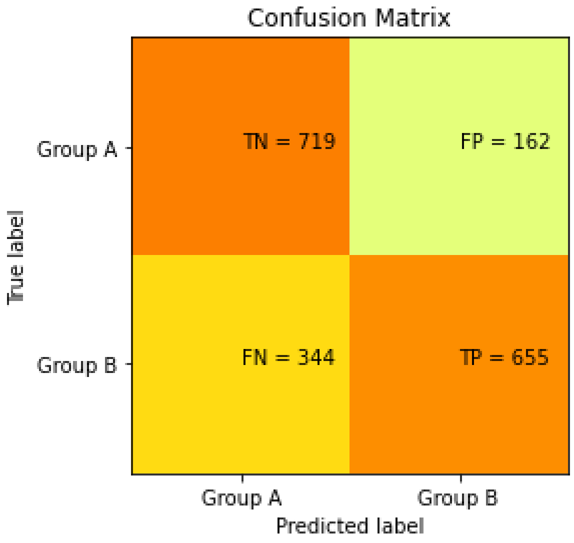


```
AUC Score : 0.7358868516643772

Accuracy Of the Model(SVC): 0.7308510638297873
```

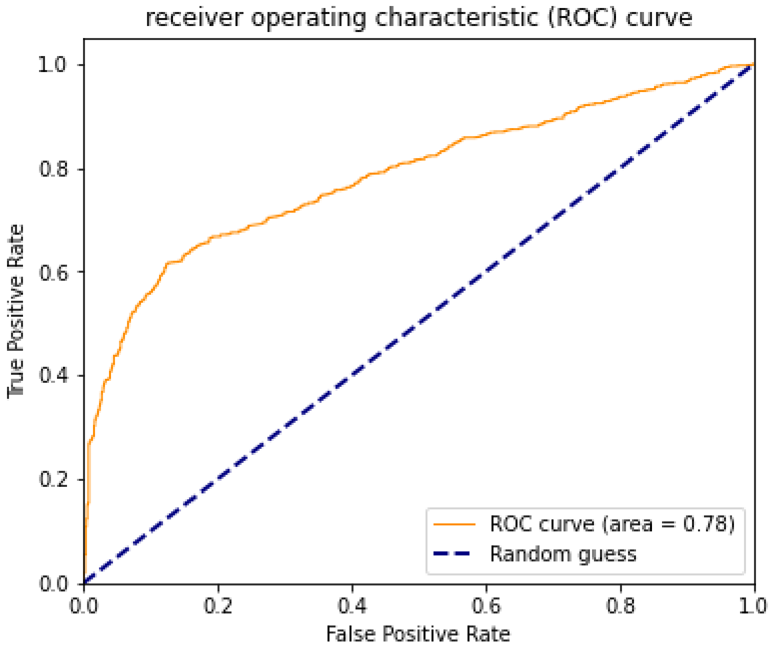

```
ROC_AUC Score of the model(SVC): 0.7837281094942843


Confusion Matrix:
[[788  93]
 [403 596]]

Classification Report:
              precision    recall  f1-score   support

         0.0       0.66      0.89      0.76       881
         1.0       0.87      0.60      0.71       999

    accuracy                           0.74      1880
   macro avg       0.76      0.75      0.73      1880
weighted avg       0.77      0.74      0.73      1880
```

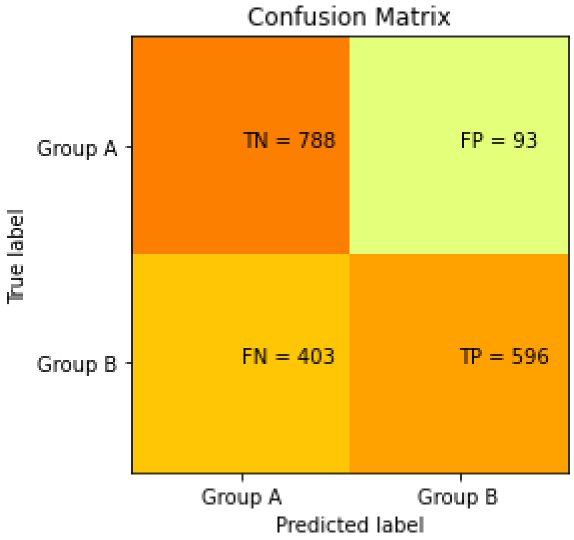


```
AUC Score : 0.7455173675377988

Accuracy Of the Model(MLPClassifier): 0.7361702127659574
```

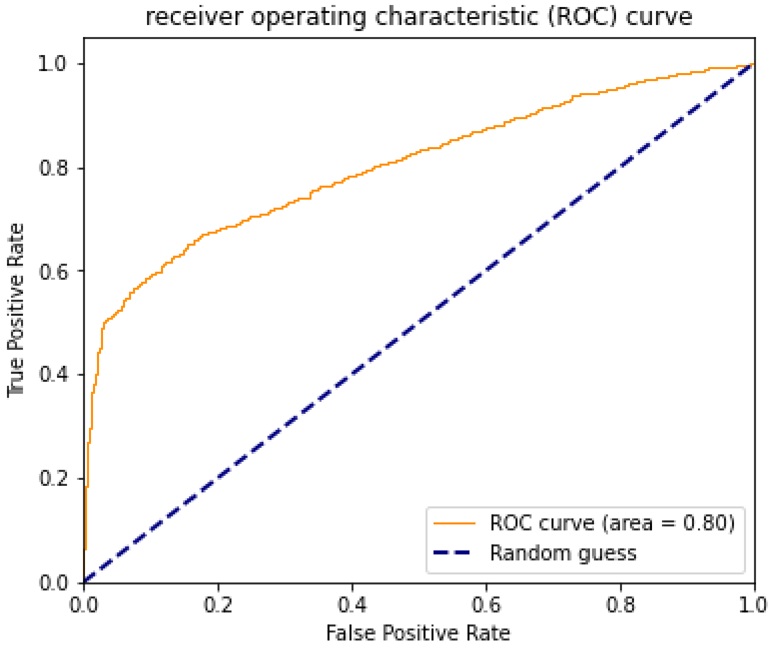

```
ROC_AUC Score of the model(MLPClassifier): 0.8036356447253156

[('GradientBoostingClassifier', 0.7462765957446809), ('XGBClassifier', 0.742553191
4893617), ('MLPClassifier', 0.7361702127659574), ('RandomForestClassifier', 0.7356
382978723405), ('LogisticRegression', 0.7329787234042553), ('DecisionTreeClassifie
r', 0.7319148936170212), ('SVC', 0.7308510638297873), ('ExtraTreesClassifier', 0.7
303191489361702), ('AdaBoostClassifier', 0.7297872340425532), ('KNeighborsClassifi
er', 0.701063829787234)]


sorted_total_auc:
 [('XGBClassifier', 0.8113221053062143), ('RandomForestClassifier', 0.810588113652
8128), ('GradientBoostingClassifier', 0.8048309376345699), ('AdaBoostClassifier',
0.8037526743542635), ('MLPClassifier', 0.8036356447253156), ('ExtraTreesClassifie
r', 0.8018728149261632), ('LogisticRegression', 0.7972240117529563), ('KNeighborsC
lassifier', 0.7919201835206375), ('DecisionTreeClassifier', 0.7867527004870931),
('SVC', 0.7837281094942843)]
```

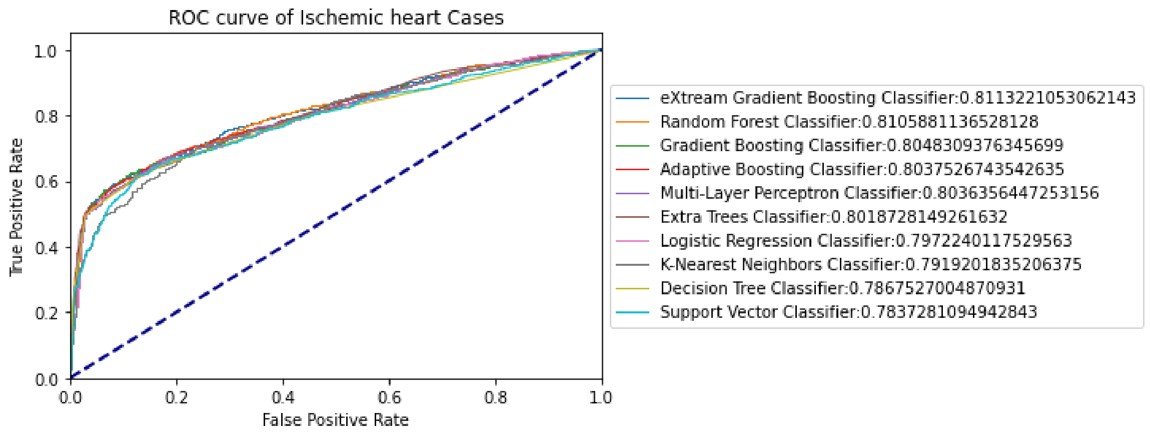

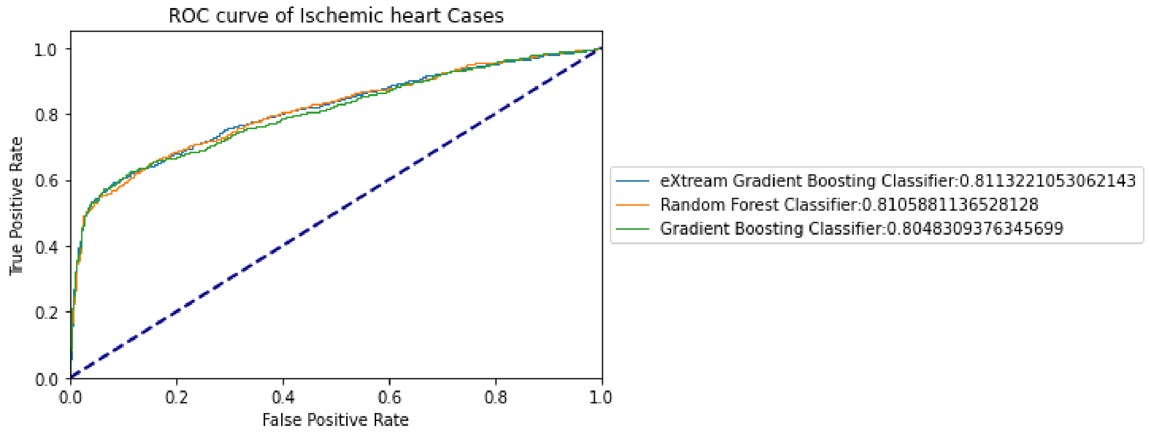

```
random state :   12751
9398
result_train_filename: data/train_test_data_ischemic heart_2013_20210519_143430.cs
v
total count : 9398, df_train count : 7518, df_test count : 1880
(7518, 38)
(1880, 38)
('Logistic Regression', LogisticRegression())
[2021-05-19 14:34:30.733232] Start parameter search for model 'Logistic Regressio
n'
[2021-05-19 14:34:31.075319] Finish parameter search for model 'Logistic Regressio
n' (time: 0 seconds)

=== train : best params ===
{'C': 0.1, 'penalty': 'l2', 'solver': 'lbfgs'}
=== train : best score : roc_auc ===
0.8038223935965394



('Random Forest', RandomForestClassifier())
[2021-05-19 14:34:31.075319] Start parameter search for model 'Random Forest'
[2021-05-19 14:34:35.661243] Finish parameter search for model 'Random Forest' (ti
me: 4 seconds)

=== train : best params ===
{'criterion': 'gini', 'max_depth': 8, 'max_features': 'auto', 'n_estimators': 100}
=== train : best score : roc_auc ===
0.8102827506402015



('Extreme Gradient Boosting)', XGBClassifier(base_score=None, booster=None, colsam
ple_bylevel=None,
              colsample_bynode=None, colsample_bytree=None, gamma=None,
              gpu_id=None, importance_type='gain', interaction_constraints=None,
              learning_rate=None, max_delta_step=None, max_depth=None,
              min_child_weight=None, missing=nan, monotone_constraints=None,
              n_estimators=100, n_jobs=None, num_parallel_tree=None,
              random_state=None, reg_alpha=None, reg_lambda=None,
              scale_pos_weight=None, subsample=None, tree_method=None,
              validate_parameters=None, verbosity=None))
[2021-05-19 14:34:35.662245] Start parameter search for model 'Extreme Gradient Bo
osting)'
[2021-05-19 14:35:02.893665] Finish parameter search for model 'Extreme Gradient B
oosting)' (time: 27 seconds)

=== train : best params ===
{'learning_rate': 0.05, 'max_depth': 5, 'n_estimators': 100, 'subsample': 0.8}
=== train : best score : roc_auc ===
0.8108491484231791



('k-Nearest Neighbours)', KNeighborsClassifier())
[2021-05-19 14:35:02.894662] Start parameter search for model 'k-Nearest Neighbour
s)'
[2021-05-19 14:35:10.824104] Finish parameter search for model 'k-Nearest Neighbou
rs)' (time: 7 seconds)

=== train : best params ===
{'n_neighbors': 60, 'weights': 'distance'}
=== train : best score : roc_auc ===
0.7910183383201808
```

```
('Decision Tree', DecisionTreeClassifier())
[2021-05-19 14:35:10.825098] Start parameter search for model 'Decision Tree'
[2021-05-19 14:35:11.372983] Finish parameter search for model 'Decision Tree' (ti
me: 0 seconds)

=== train : best params ===
{'criterion': 'entropy', 'max_depth': 4}
=== train : best score : roc_auc ===
0.7894724458858016


('Extra Tree', ExtraTreesClassifier())
[2021-05-19 14:35:11.372983] Start parameter search for model 'Extra Tree'
[2021-05-19 14:35:14.377217] Finish parameter search for model 'Extra Tree' (time:
3 seconds)

=== train : best params ===
{'max_features': 'log2', 'n_estimators': 100}
=== train : best score : roc_auc ===
0.8056712602381164


('Gradient Boosting', GradientBoostingClassifier())
[2021-05-19 14:35:14.377217] Start parameter search for model 'Gradient Boosting'
[2021-05-19 14:35:29.508505] Finish parameter search for model 'Gradient Boosting'
(time: 15 seconds)

=== train : best params ===
{'learning_rate': 0.075, 'max_depth': 8, 'max_features': 4, 'n_estimators': 50}
=== train : best score : roc_auc ===
0.8119947620794763


('Adaptive Boosting', AdaBoostClassifier())
[2021-05-19 14:35:29.508505] Start parameter search for model 'Adaptive Boosting'
[2021-05-19 14:35:33.105346] Finish parameter search for model 'Adaptive Boosting'
(time: 3 seconds)

=== train : best params ===
{'learning_rate': 0.2, 'n_estimators': 100}
=== train : best score : roc_auc ===
0.8026157538507344


('Suppport Vector Classfier', SVC(probability=True))
[2021-05-19 14:35:33.105346] Start parameter search for model 'Suppport Vector Cla
ssfier'
[2021-05-19 14:39:53.798304] Finish parameter search for model 'Suppport Vector Cl
assfier' (time: 260 seconds)

=== train : best params ===
{'C': 1, 'gamma': 0.1, 'kernel': 'rbf'}
=== train : best score : roc_auc ===
0.7880356692784343


('Neural Network', MLPClassifier())
[2021-05-19 14:39:53.799301] Start parameter search for model 'Neural Network'
[2021-05-19 14:42:33.308394] Finish parameter search for model 'Neural Network' (t
```

```
ime: 159 seconds)

=== train : best params ===
{'activation': 'logistic', 'alpha': 0.05, 'hidden_layer_sizes': (50,), 'learning_r
ate': 'adaptive'}
=== train : best score : roc_auc ===
0.8080470436432559



Confusion Matrix:
[[813  68]
 [432 567]]

Classification Report:
              precision    recall  f1-score   support

         0.0       0.65      0.92      0.76       881
         1.0       0.89      0.57      0.69       999

    accuracy                           0.73      1880
   macro avg       0.77      0.75      0.73      1880
weighted avg       0.78      0.73      0.73      1880
```

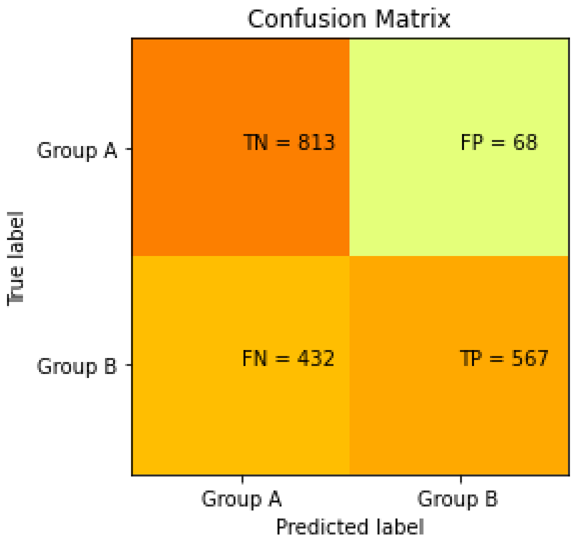


```
AUC Score : 0.7451912752707304

Accuracy Of the Model(LogisticRegression): 0.7340425531914894
```

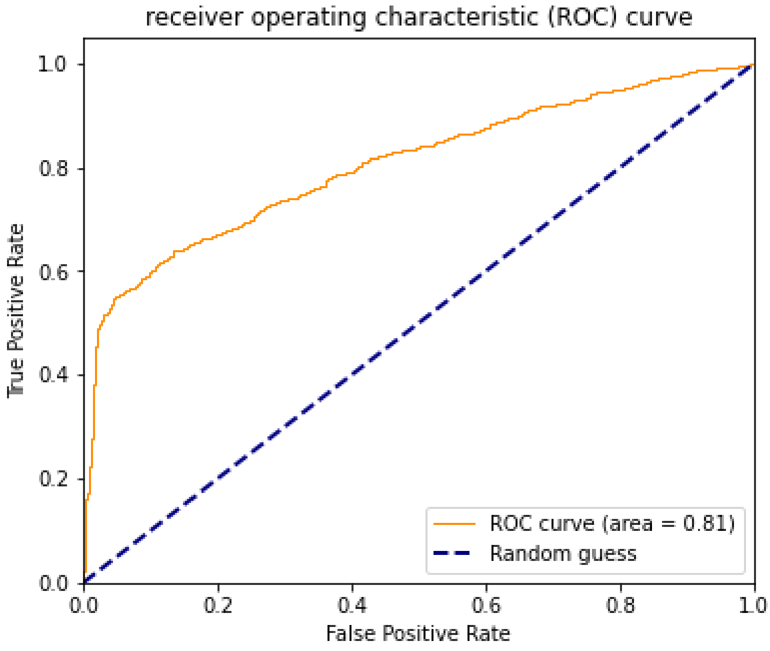


```
ROC_AUC Score of the model(LogisticRegression): 0.8072022078832521


Confusion Matrix:
[[793  88]
 [403 596]]

Classification Report:
              precision    recall  f1-score   support

         0.0       0.66      0.90      0.76       881
         1.0       0.87      0.60      0.71       999

    accuracy                           0.74      1880
   macro avg       0.77      0.75      0.74      1880
weighted avg       0.77      0.74      0.73      1880
```

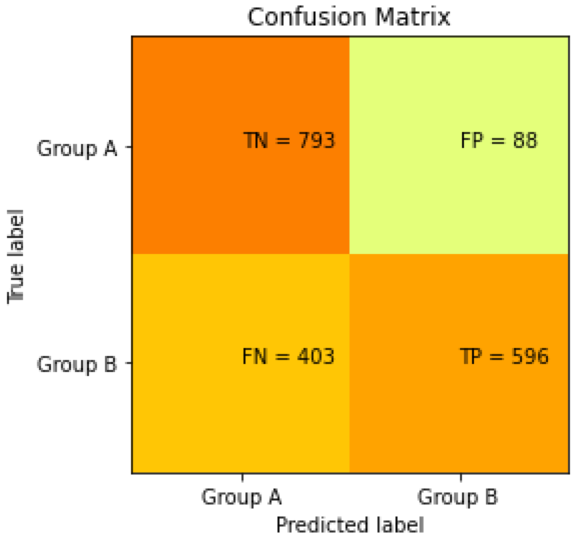


```
AUC Score : 0.748355051987288

Accuracy Of the Model(RandomForestClassifier): 0.7388297872340426
```

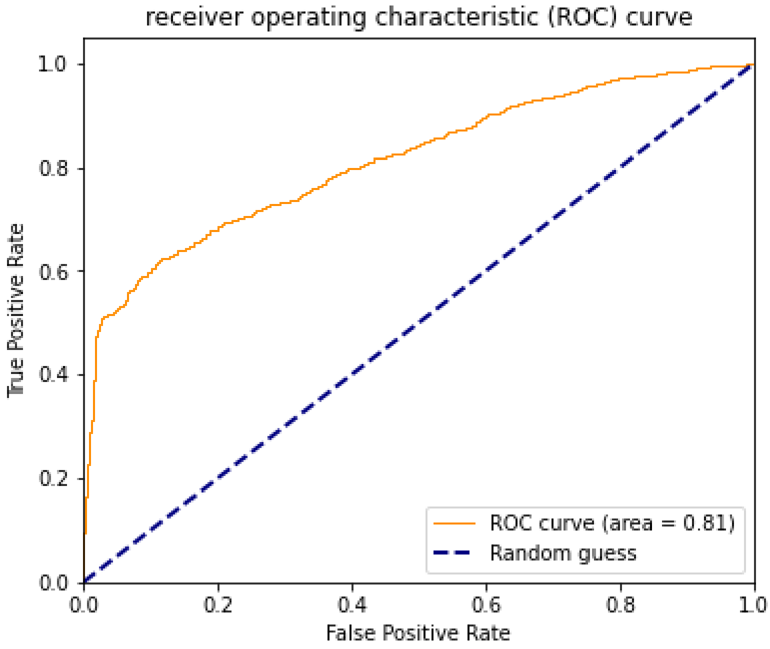

```
ROC_AUC Score of the model(RandomForestClassifier): 0.8145807555569189


Confusion Matrix:
[[796  85]
 [400 599]]

Classification Report:
              precision    recall  f1-score   support

         0.0       0.67      0.90      0.77       881
         1.0       0.88      0.60      0.71       999

    accuracy                           0.74      1880
   macro avg       0.77      0.75      0.74      1880
weighted avg       0.78      0.74      0.74      1880
```

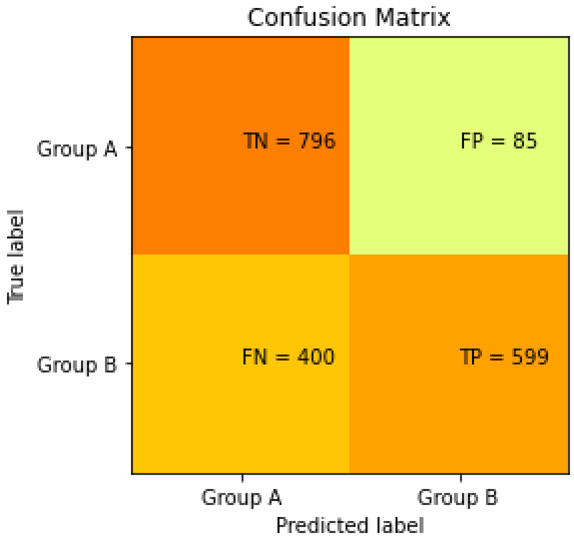


```
AUC Score : 0.7515591641584832

Accuracy Of the Model(XGBClassifier): 0.7420212765957447
```

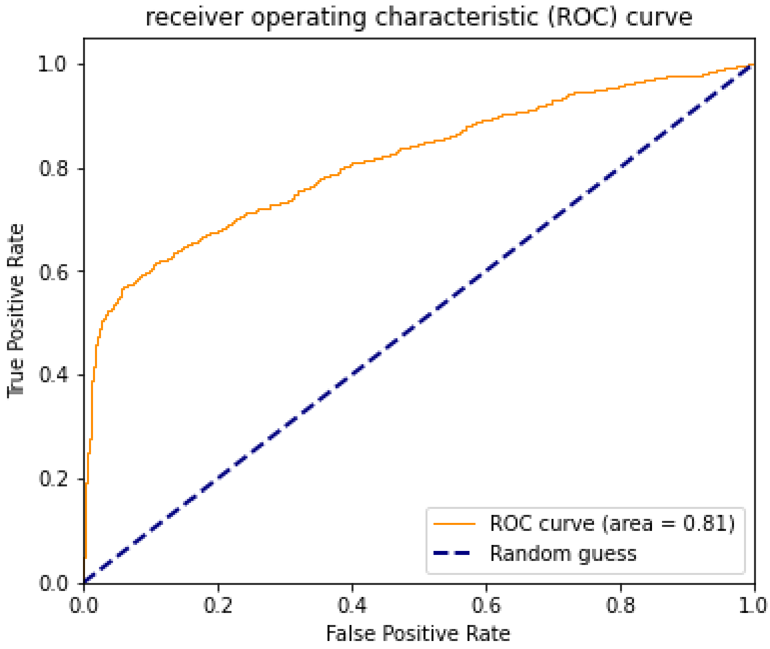

```
ROC_AUC Score of the model(XGBClassifier): 0.8131252705600038


Confusion Matrix:
[[796  85]
 [461 538]]

Classification Report:
              precision    recall  f1-score   support

         0.0       0.63      0.90      0.74       881
         1.0       0.86      0.54      0.66       999

    accuracy                           0.71      1880
   macro avg       0.75      0.72      0.70      1880
weighted avg       0.76      0.71      0.70      1880
```

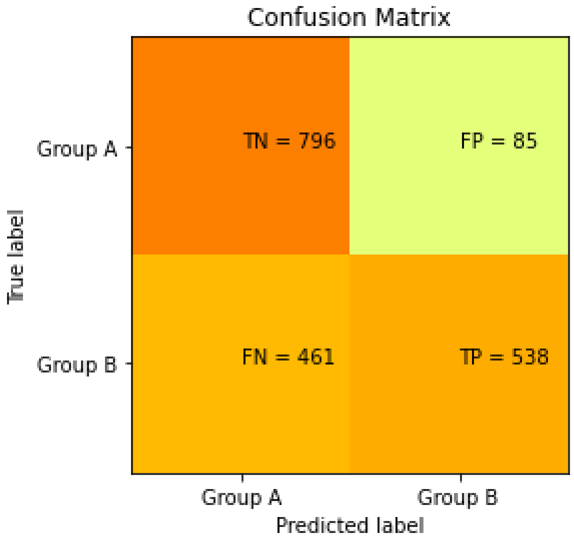


```
AUC Score : 0.7210286336279526

Accuracy Of the Model(KNeighborsClassifier): 0.7095744680851064
```

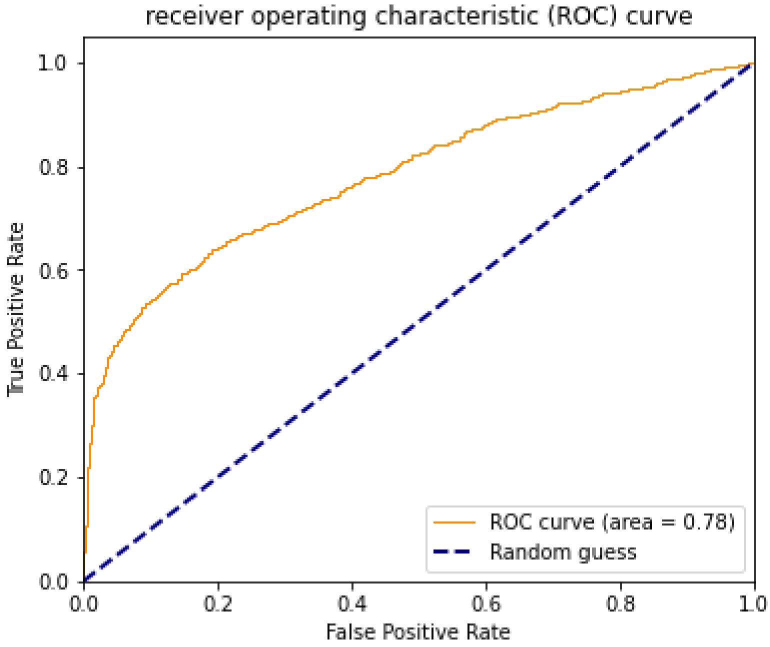

ROC_AUC Score of the model(KNeighborsClassifier): 0.7838451391232322

Confusion Matrix:
[[809 72]
 [437 562]]

Classification Report:

	precision	recall	f1-score	support
0.0	0.65	0.92	0.76	881
1.0	0.89	0.56	0.69	999
accuracy			0.73	1880
macro avg	0.77	0.74	0.72	1880
weighted avg	0.78	0.73	0.72	1880

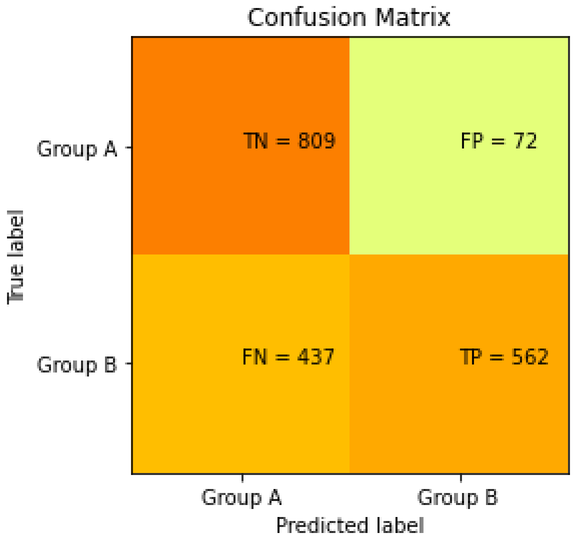

AUC Score : 0.7404186252086365

Accuracy Of the Model(DecisionTreeClassifier): 0.7292553191489362

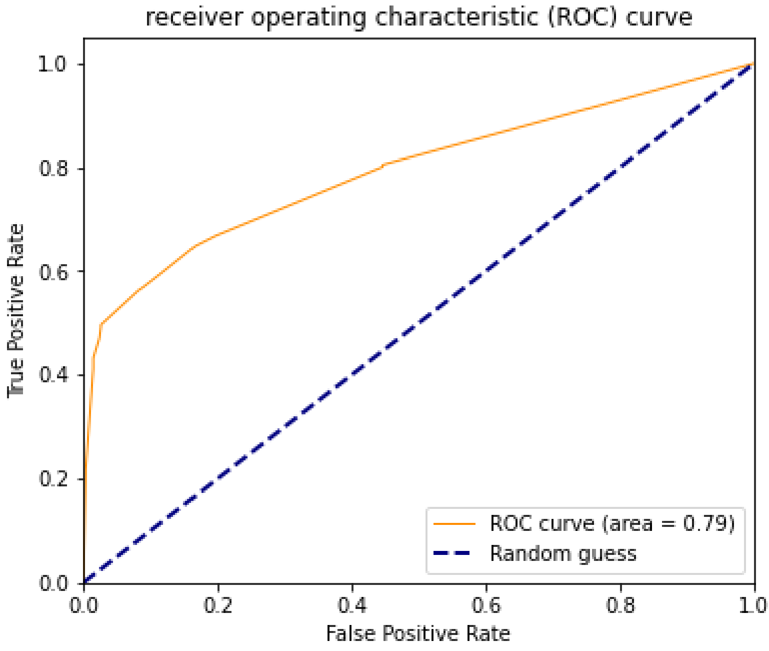

```
ROC_AUC Score of the model(DecisionTreeClassifier): 0.7927518892331606


Confusion Matrix:
[[788  93]
 [420 579]]

Classification Report:
              precision    recall  f1-score   support

         0.0       0.65      0.89      0.75       881
         1.0       0.86      0.58      0.69       999

    accuracy                           0.73      1880
   macro avg       0.76      0.74      0.72      1880
weighted avg       0.76      0.73      0.72      1880
```

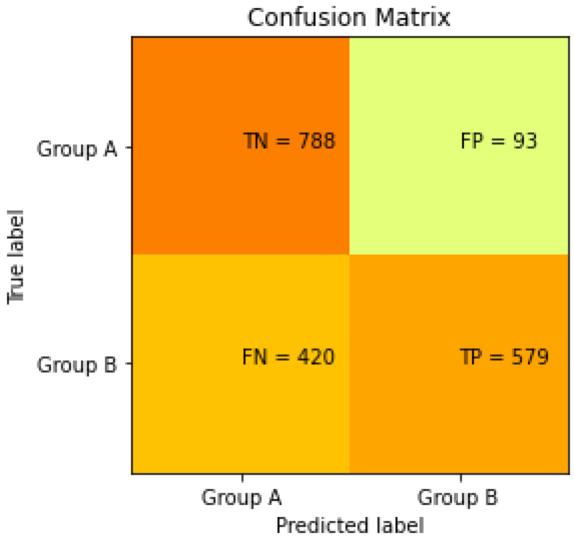


```
AUC Score : 0.7370088590292905

Accuracy Of the Model(ExtraTreesClassifier): 0.7271276595744681
```

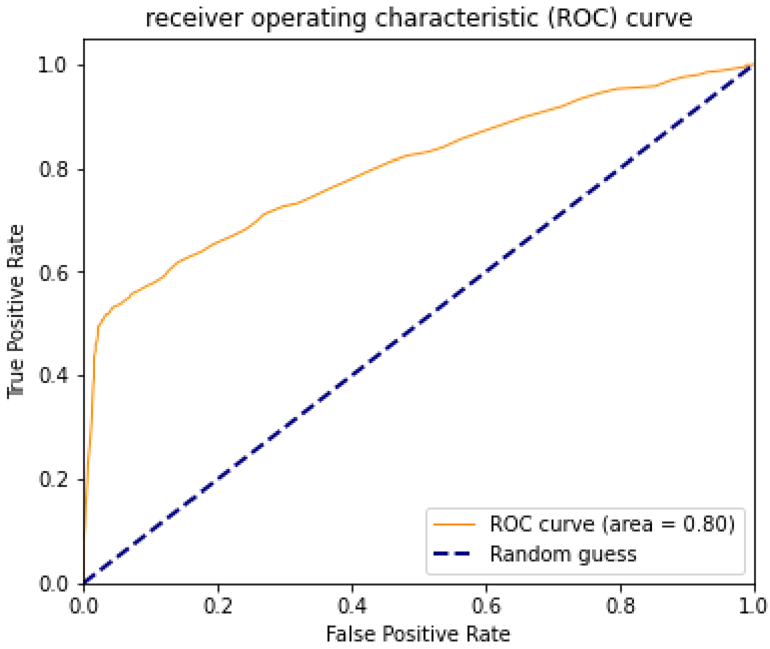

ROC_AUC Score of the model(ExtraTreesClassifier): 0.7988931042279511

Confusion Matrix:
[[778 103]
 [391 608]]

Classification Report:

	precision	recall	f1-score	support
0.0	0.67	0.88	0.76	881
1.0	0.86	0.61	0.71	999
accuracy			0.74	1880
macro avg	0.76	0.75	0.74	1880
weighted avg	0.77	0.74	0.73	1880

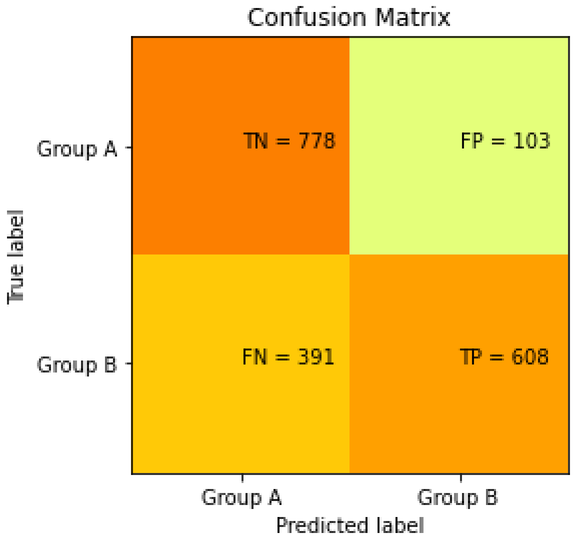

AUC Score : 0.7458480046448265

Accuracy Of the Model(GradientBoostingClassifier): 0.7372340425531915

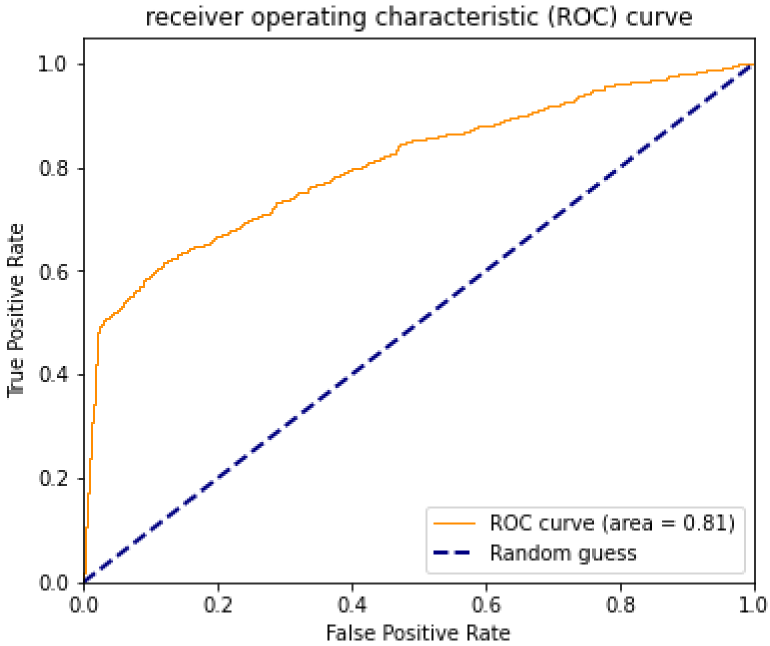

```
ROC_AUC Score of the model(GradientBoostingClassifier): 0.80652502672934


Confusion Matrix:
[[818  63]
 [429 570]]

Classification Report:
              precision    recall  f1-score   support

         0.0       0.66      0.93      0.77       881
         1.0       0.90      0.57      0.70       999

    accuracy                           0.74      1880
   macro avg       0.78      0.75      0.73      1880
weighted avg       0.79      0.74      0.73      1880
```

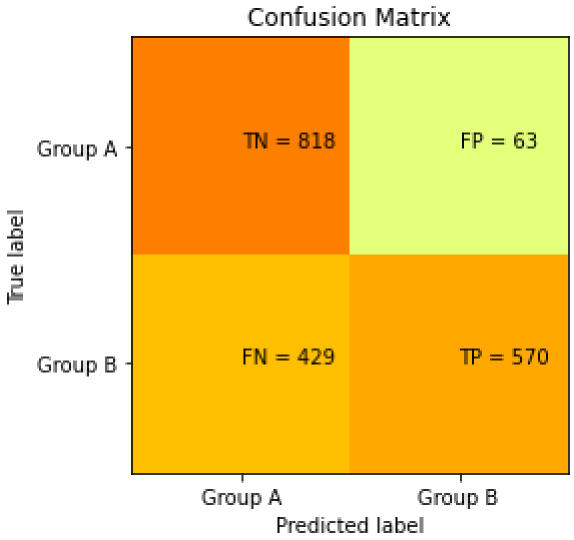


```
AUC Score : 0.7495304612217212

Accuracy Of the Model(AdaBoostClassifier): 0.7382978723404255
```

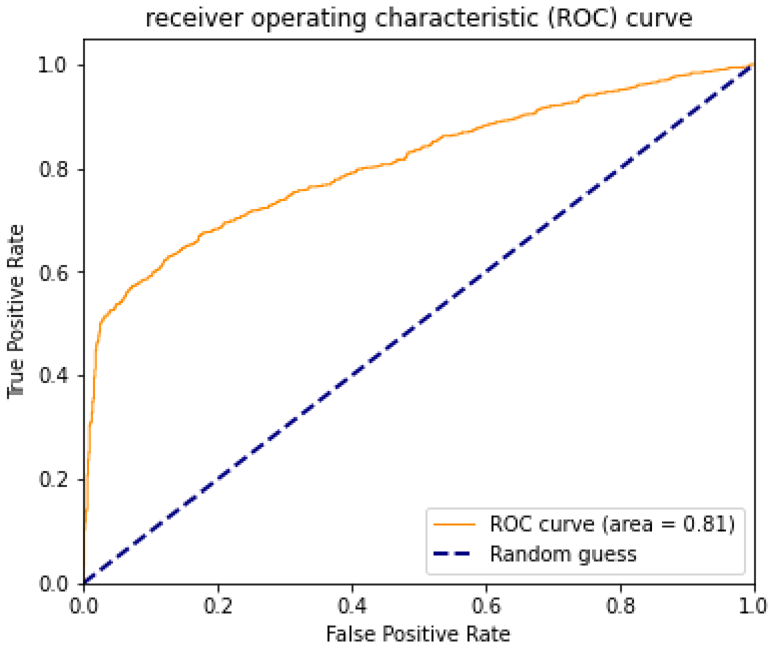

ROC_AUC Score of the model(AdaBoostClassifier): 0.8078413260025066

```
Confusion Matrix:
[[703 178]
 [346 653]]

Classification Report:
              precision    recall  f1-score   support

         0.0       0.67      0.80      0.73       881
         1.0       0.79      0.65      0.71       999

    accuracy                           0.72      1880
   macro avg       0.73      0.73      0.72      1880
weighted avg       0.73      0.72      0.72      1880
```

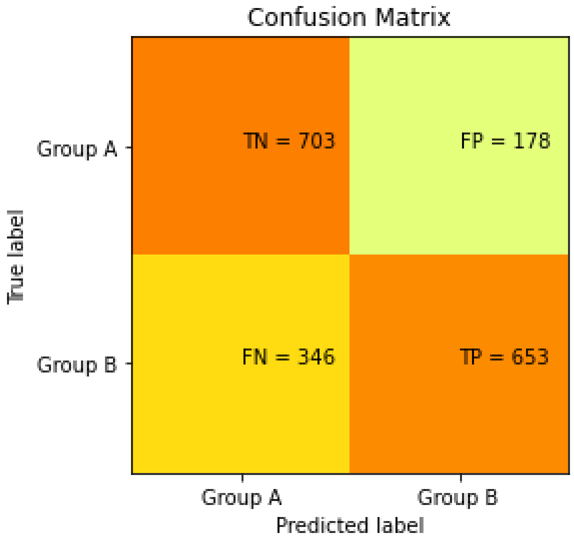

AUC Score : 0.7258052604250107

Accuracy Of the Model(SVC): 0.7212765957446808

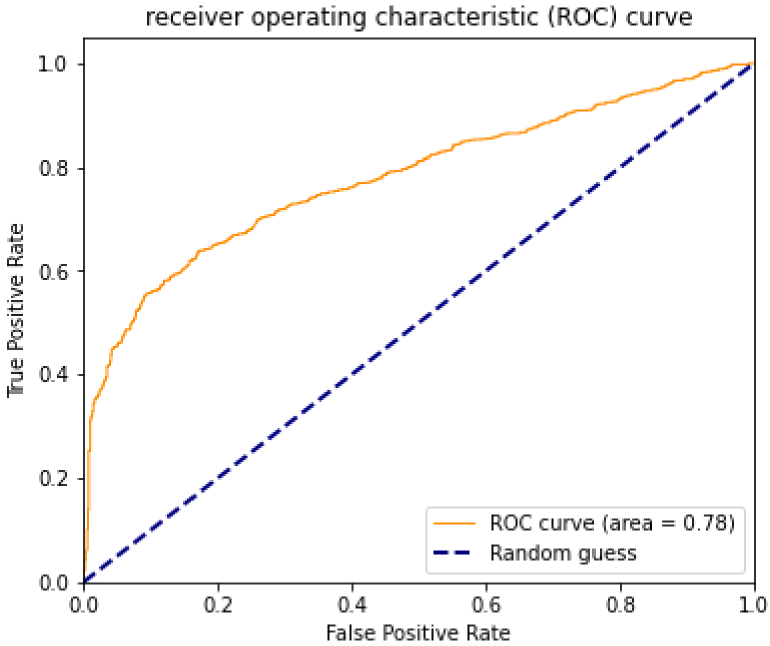

```
ROC_AUC Score of the model(SVC): 0.7796593415208625


Confusion Matrix:
[[794  87]
 [405 594]]

Classification Report:
              precision    recall  f1-score   support

         0.0       0.66      0.90      0.76       881
         1.0       0.87      0.59      0.71       999

    accuracy                           0.74      1880
   macro avg       0.77      0.75      0.74      1880
weighted avg       0.77      0.74      0.73      1880
```

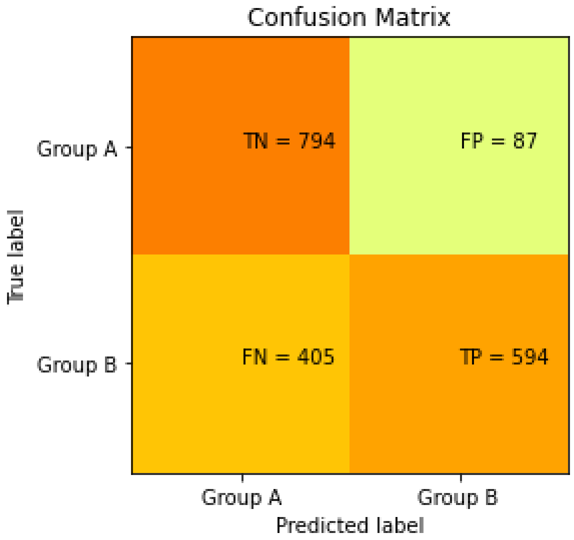


```
AUC Score : 0.747921587876185

Accuracy Of the Model(MLPClassifier): 0.7382978723404255
```

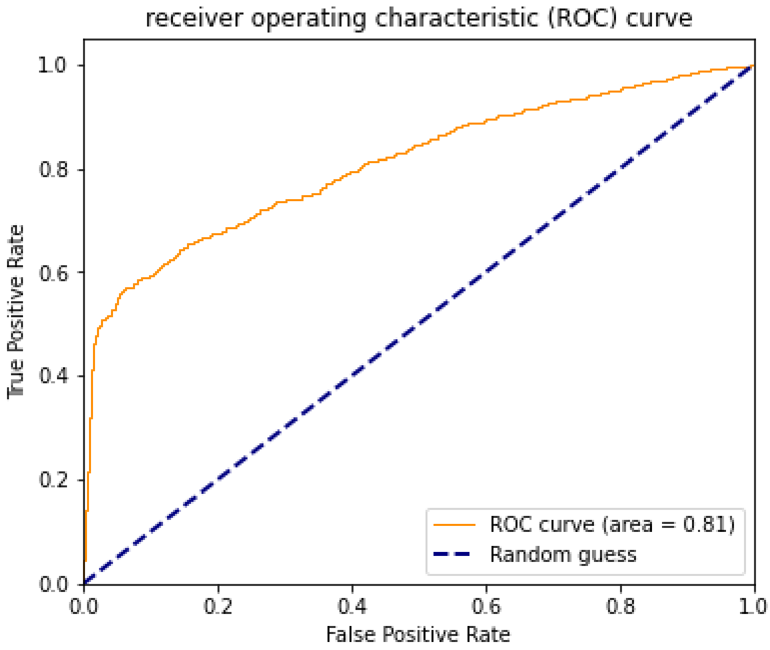

```
ROC_AUC Score of the model(MLPClassifier): 0.8114130021053971

[('XGBClassifier', 0.7420212765957447), ('RandomForestClassifier', 0.7388297872340426), ('AdaBoostClassifier', 0.7382978723404255), ('MLPClassifier', 0.7382978723404255), ('GradientBoostingClassifier', 0.7372340425531915), ('LogisticRegression', 0.7340425531914894), ('DecisionTreeClassifier', 0.7292553191489362), ('ExtraTreesClassifier', 0.7271276595744681), ('SVC', 0.7212765957446808), ('KNeighborsClassifier', 0.7095744680851064)]


sorted_total_auc:
 [('RandomForestClassifier', 0.8145807555569189), ('XGBClassifier', 0.8131252705600038), ('MLPClassifier', 0.8114130021053971), ('AdaBoostClassifier', 0.8078413260025066), ('LogisticRegression', 0.8072022078832521), ('GradientBoostingClassifier', 0.80652502672934), ('ExtraTreesClassifier', 0.7988931042279511), ('DecisionTreeClassifier', 0.7927518892331606), ('KNeighborsClassifier', 0.7838451391232322), ('SVC', 0.7796593415208625)]
```

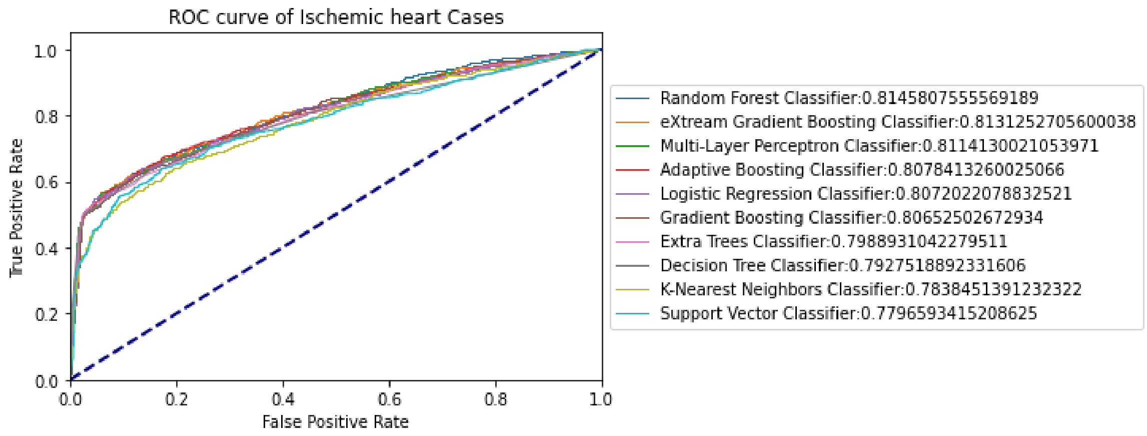

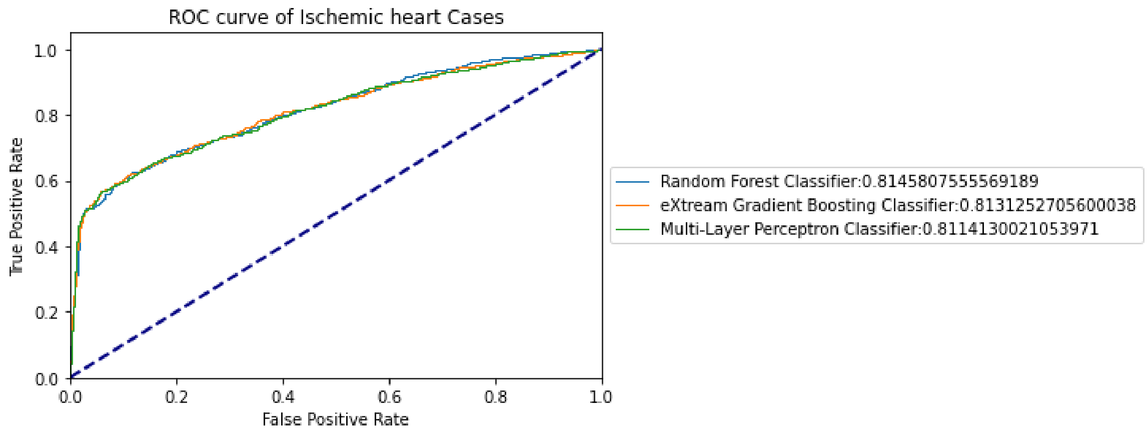

```
random state :   12780
9398
result_train_filename: data/train_test_data_ischemic heart_2013_20210519_144240.cs
v
total count : 9398, df_train count : 7518, df_test count : 1880
(7518, 38)
(1880, 38)
('Logistic Regression', LogisticRegression())
[2021-05-19 14:42:41.064195] Start parameter search for model 'Logistic Regressio
n'
[2021-05-19 14:42:41.408775] Finish parameter search for model 'Logistic Regressio
n' (time: 0 seconds)

=== train : best params ===
{'C': 0.1, 'penalty': 'l2', 'solver': 'lbfgs'}
=== train : best score : roc_auc ===
0.8023041359029972


('Random Forest', RandomForestClassifier())
[2021-05-19 14:42:41.409771] Start parameter search for model 'Random Forest'
[2021-05-19 14:42:45.745494] Finish parameter search for model 'Random Forest' (ti
me: 4 seconds)

=== train : best params ===
{'criterion': 'gini', 'max_depth': 10, 'max_features': 'auto', 'n_estimators': 70}
=== train : best score : roc_auc ===
0.8102693807748985


('Extreme Gradient Boosting)', XGBClassifier(base_score=None, booster=None, colsam
ple_bylevel=None,
              colsample_bynode=None, colsample_bytree=None, gamma=None,
              gpu_id=None, importance_type='gain', interaction_constraints=None,
              learning_rate=None, max_delta_step=None, max_depth=None,
              min_child_weight=None, missing=nan, monotone_constraints=None,
              n_estimators=100, n_jobs=None, num_parallel_tree=None,
              random_state=None, reg_alpha=None, reg_lambda=None,
              scale_pos_weight=None, subsample=None, tree_method=None,
              validate_parameters=None, verbosity=None))
[2021-05-19 14:42:45.746491] Start parameter search for model 'Extreme Gradient Bo
osting)'
[2021-05-19 14:43:12.461653] Finish parameter search for model 'Extreme Gradient B
oosting)' (time: 26 seconds)

=== train : best params ===
{'learning_rate': 0.01, 'max_depth': 8, 'n_estimators': 100, 'subsample': 0.7}
=== train : best score : roc_auc ===
0.8116776442662951


('k-Nearest Neighbours)', KNeighborsClassifier())
[2021-05-19 14:43:12.463648] Start parameter search for model 'k-Nearest Neighbour
s)'
[2021-05-19 14:43:20.301333] Finish parameter search for model 'k-Nearest Neighbou
rs)' (time: 7 seconds)

=== train : best params ===
{'n_neighbors': 60, 'weights': 'distance'}
=== train : best score : roc_auc ===
0.7894267054748989
```

```
('Decision Tree', DecisionTreeClassifier())
[2021-05-19 14:43:20.301333] Start parameter search for model 'Decision Tree'
[2021-05-19 14:43:20.853468] Finish parameter search for model 'Decision Tree' (ti
me: 0 seconds)

=== train : best params ===
{'criterion': 'gini', 'max_depth': 5}
=== train : best score : roc_auc ===
0.7938205284343101


('Extra Tree', ExtraTreesClassifier())
[2021-05-19 14:43:20.854466] Start parameter search for model 'Extra Tree'
[2021-05-19 14:43:23.791928] Finish parameter search for model 'Extra Tree' (time:
2 seconds)

=== train : best params ===
{'max_features': 'auto', 'n_estimators': 100}
=== train : best score : roc_auc ===
0.8017474459031166


('Gradient Boosting', GradientBoostingClassifier())
[2021-05-19 14:43:23.791928] Start parameter search for model 'Gradient Boosting'
[2021-05-19 14:43:38.838879] Finish parameter search for model 'Gradient Boosting'
(time: 15 seconds)

=== train : best params ===
{'learning_rate': 0.075, 'max_depth': 5, 'max_features': 6, 'n_estimators': 50}
=== train : best score : roc_auc ===
0.8106567618610252


('Adaptive Boosting', AdaBoostClassifier())
[2021-05-19 14:43:38.838879] Start parameter search for model 'Adaptive Boosting'
[2021-05-19 14:43:42.084436] Finish parameter search for model 'Adaptive Boosting'
(time: 3 seconds)

=== train : best params ===
{'learning_rate': 0.2, 'n_estimators': 50}
=== train : best score : roc_auc ===
0.8030499001253933


('Suppport Vector Classfier', SVC(probability=True))
[2021-05-19 14:43:42.084436] Start parameter search for model 'Suppport Vector Cla
ssfier'
[2021-05-19 14:48:03.395945] Finish parameter search for model 'Suppport Vector Cl
assfier' (time: 261 seconds)

=== train : best params ===
{'C': 1, 'gamma': 0.1, 'kernel': 'rbf'}
=== train : best score : roc_auc ===
0.7834934002851566


('Neural Network', MLPClassifier())
[2021-05-19 14:48:03.395945] Start parameter search for model 'Neural Network'
[2021-05-19 14:50:44.098169] Finish parameter search for model 'Neural Network' (t
```

```
ime: 160 seconds)

=== train : best params ===
{'activation': 'logistic', 'alpha': 0.05, 'hidden_layer_sizes': (50,), 'learning_r
ate': 'constant'}
=== train : best score : roc_auc ===
0.8076497406699592



Confusion Matrix:
[[821  60]
 [426 573]]

Classification Report:
              precision    recall  f1-score   support

         0.0       0.66      0.93      0.77       881
         1.0       0.91      0.57      0.70       999

    accuracy                           0.74      1880
   macro avg       0.78      0.75      0.74      1880
weighted avg       0.79      0.74      0.73      1880
```

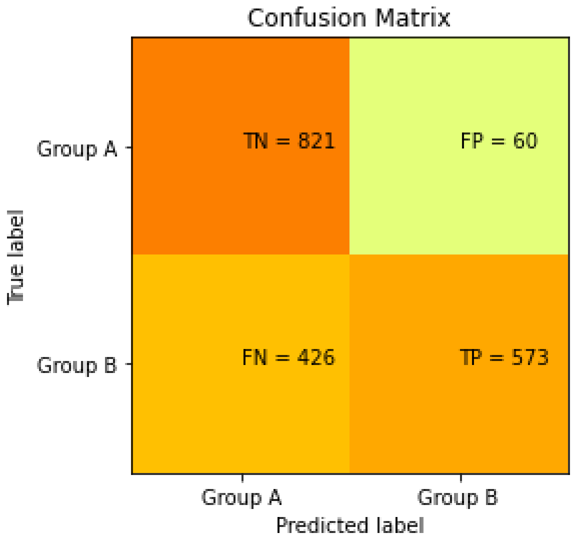


```
AUC Score : 0.7527345733929162

Accuracy Of the Model(LogisticRegression): 0.7414893617021276
```

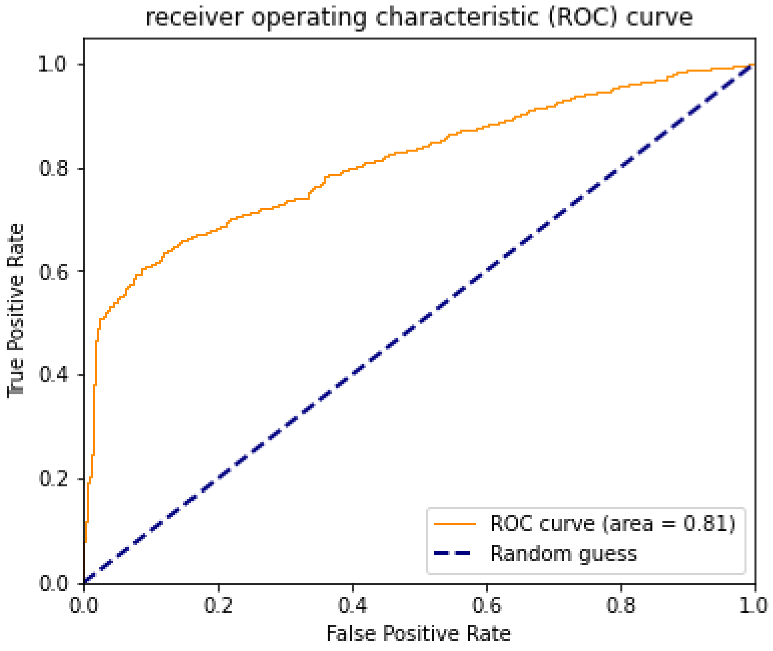


```
ROC_AUC Score of the model(LogisticRegression): 0.811255068916817


Confusion Matrix:
[[800  81]
 [404 595]]

Classification Report:
              precision    recall  f1-score   support

         0.0       0.66      0.91      0.77       881
         1.0       0.88      0.60      0.71       999

    accuracy                           0.74      1880
   macro avg       0.77      0.75      0.74      1880
weighted avg       0.78      0.74      0.74      1880
```

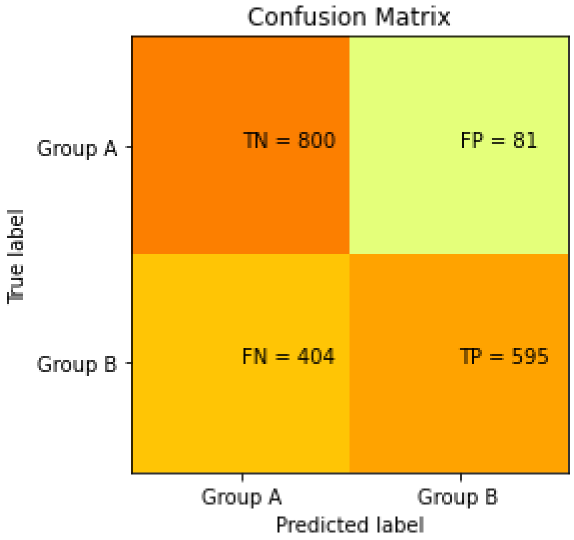

AUC Score : 0.7518273097160725

Accuracy Of the Model(RandomForestClassifier): 0.7420212765957447

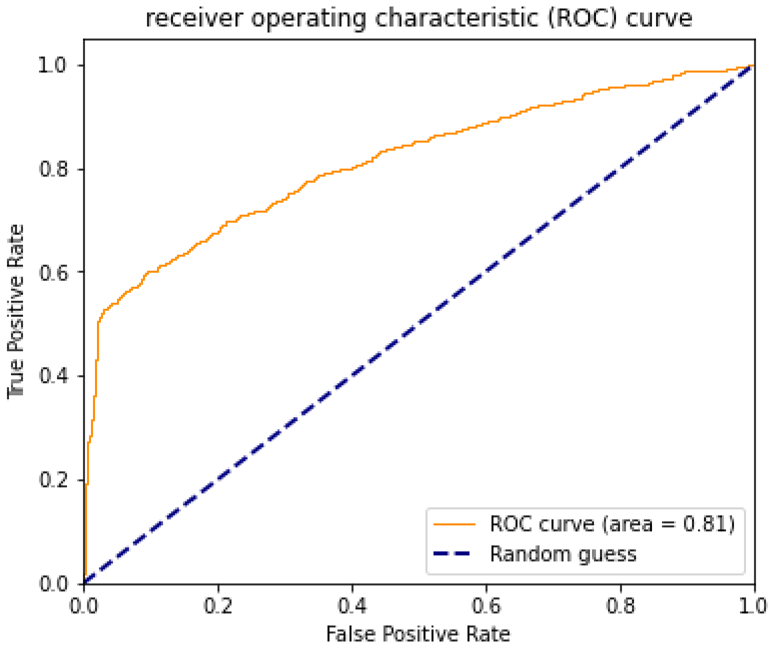

```
ROC_AUC Score of the model(RandomForestClassifier): 0.8141262715610049


Confusion Matrix:
[[800  81]
 [408 591]]

Classification Report:
              precision    recall  f1-score   support

         0.0       0.66      0.91      0.77       881
         1.0       0.88      0.59      0.71       999

    accuracy                           0.74      1880
   macro avg       0.77      0.75      0.74      1880
weighted avg       0.78      0.74      0.73      1880
```

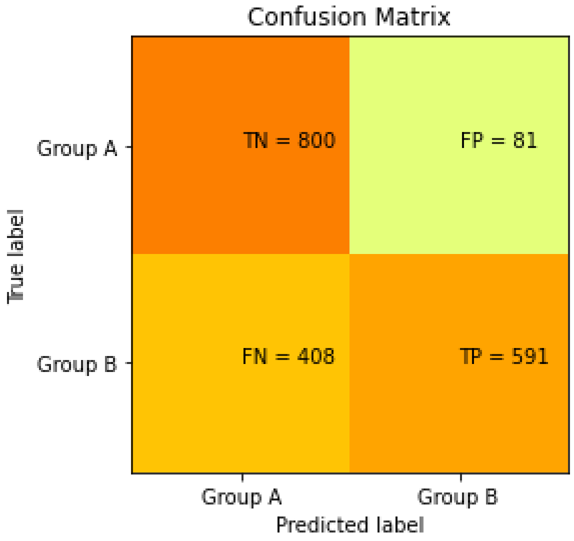


```
AUC Score : 0.7498253077140704

Accuracy Of the Model(XGBClassifier): 0.7398936170212767
```

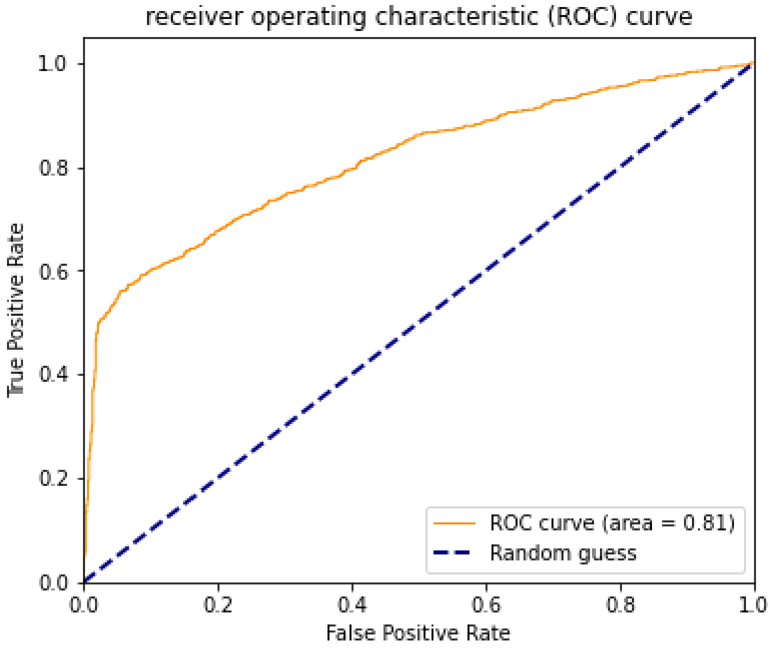

```
ROC_AUC Score of the model(XGBClassifier): 0.8117231874326085


Confusion Matrix:
[[798  83]
 [462 537]]

Classification Report:
              precision    recall  f1-score   support

         0.0       0.63      0.91      0.75       881
         1.0       0.87      0.54      0.66       999

    accuracy                           0.71      1880
   macro avg       0.75      0.72      0.70      1880
weighted avg       0.76      0.71      0.70      1880
```

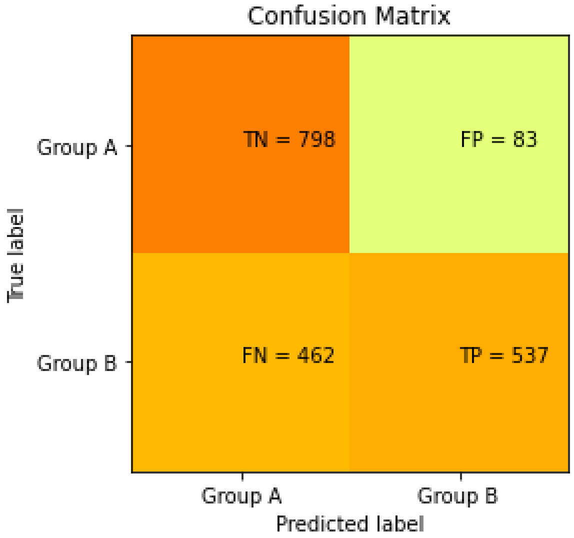


```
AUC Score : 0.7216632069072477

Accuracy Of the Model(KNeighborsClassifier): 0.7101063829787234
```

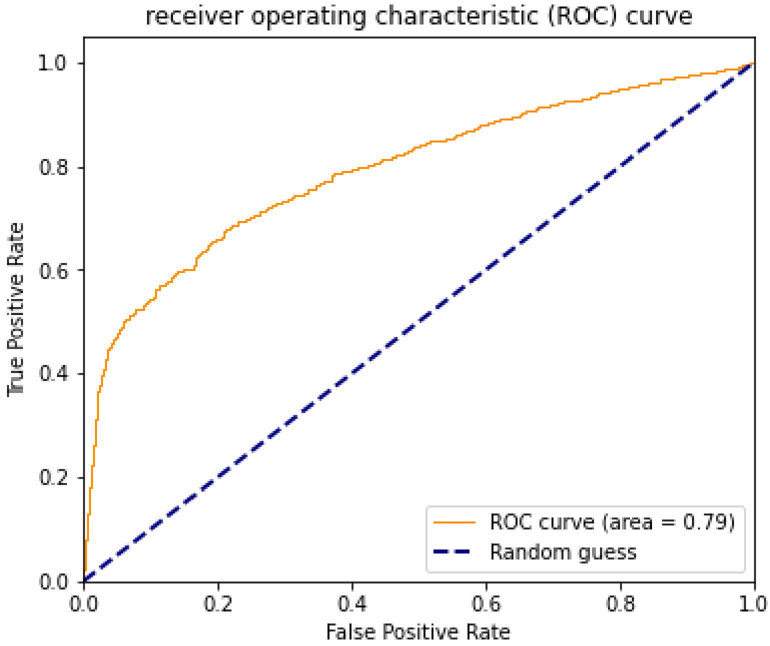

```
ROC_AUC Score of the model(KNeighborsClassifier): 0.7943687160486252


Confusion Matrix:
[[768 113]
 [382 617]]

Classification Report:
              precision    recall  f1-score   support

         0.0       0.67      0.87      0.76       881
         1.0       0.85      0.62      0.71       999

    accuracy                           0.74      1880
   macro avg       0.76      0.74      0.73      1880
weighted avg       0.76      0.74      0.73      1880
```

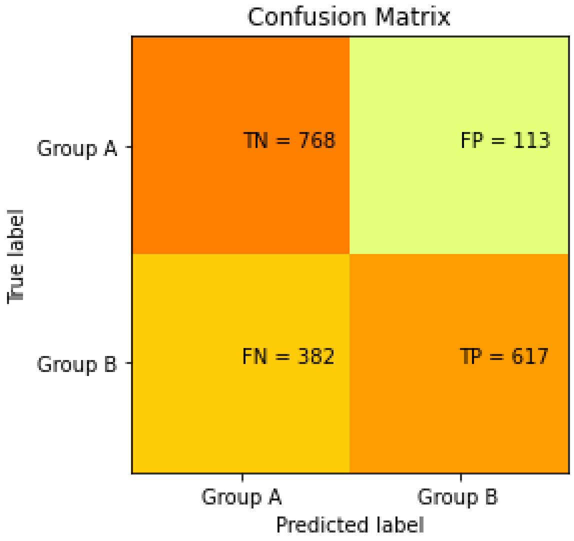


```
AUC Score : 0.7446771402503525

Accuracy Of the Model(DecisionTreeClassifier): 0.7367021276595744
```

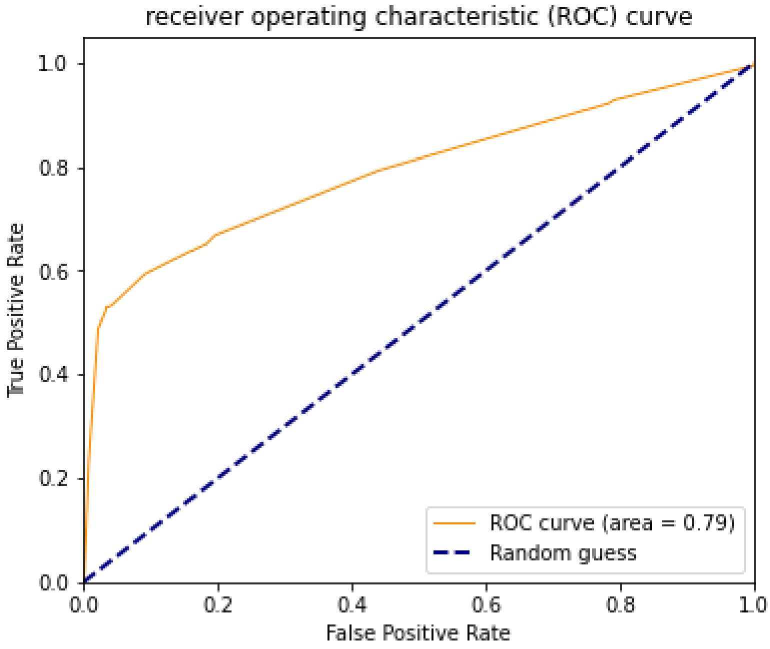

```
ROC_AUC Score of the model(DecisionTreeClassifier): 0.7904612898937531


Confusion Matrix:
[[799  82]
 [409 590]]

Classification Report:
              precision    recall  f1-score   support

         0.0       0.66      0.91      0.76       881
         1.0       0.88      0.59      0.71       999

    accuracy                           0.74      1880
   macro avg       0.77      0.75      0.74      1880
weighted avg       0.78      0.74      0.73      1880
```

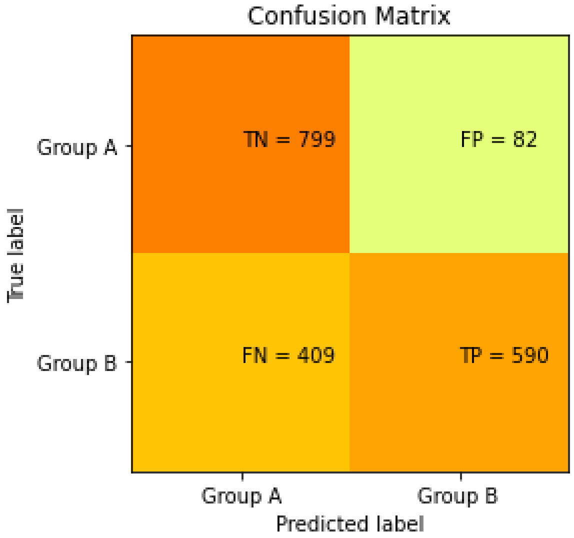


```
AUC Score : 0.7487572703236721

Accuracy Of the Model(ExtraTreesClassifier): 0.7388297872340426
```

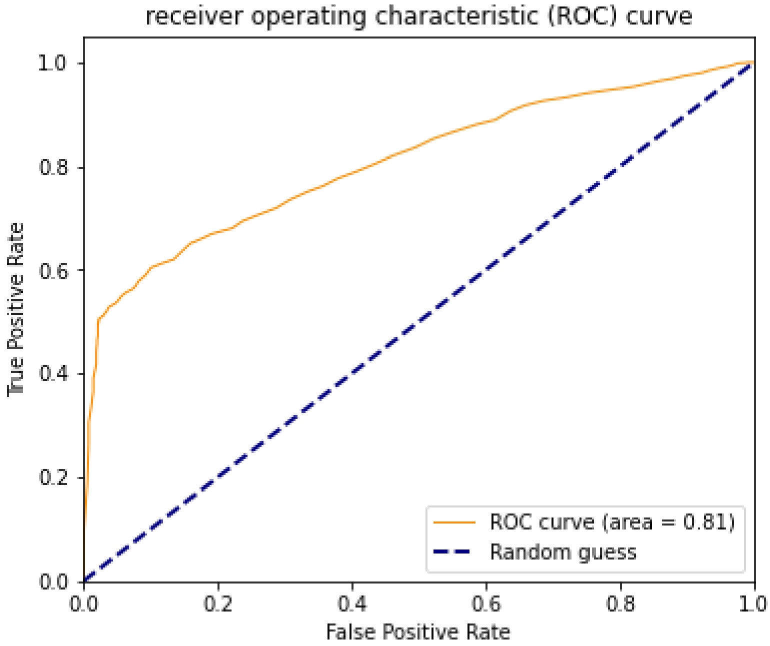

```
ROC_AUC Score of the model(ExtraTreesClassifier): 0.8072039121982368


Confusion Matrix:
[[798  83]
 [395 604]]

Classification Report:
              precision    recall  f1-score   support

         0.0       0.67      0.91      0.77       881
         1.0       0.88      0.60      0.72       999

    accuracy                           0.75      1880
   macro avg       0.77      0.76      0.74      1880
weighted avg       0.78      0.75      0.74      1880
```

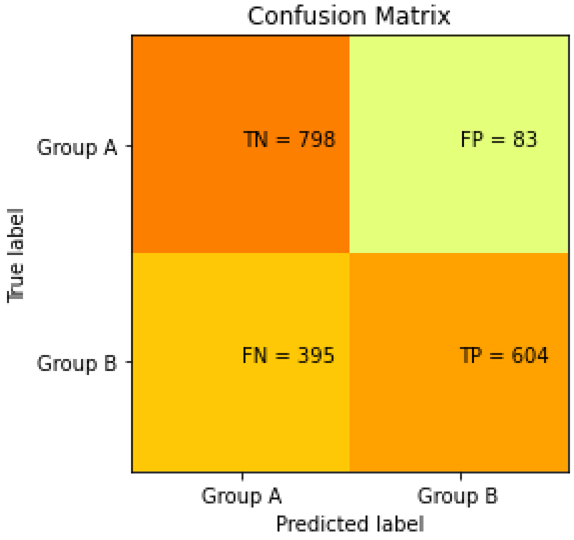


```
AUC Score : 0.7551967404407813

Accuracy Of the Model(GradientBoostingClassifier): 0.7457446808510638
```

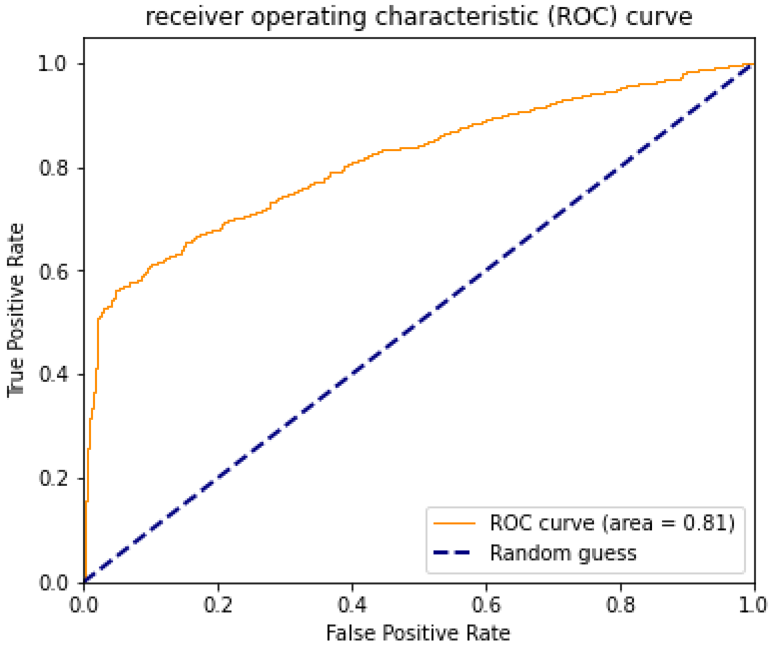

```
ROC_AUC Score of the model(GradientBoostingClassifier): 0.8129707460013931


Confusion Matrix:
[[839  42]
 [446 553]]

Classification Report:
              precision    recall  f1-score   support

         0.0       0.65      0.95      0.77       881
         1.0       0.93      0.55      0.69       999

    accuracy                           0.74      1880
   macro avg       0.79      0.75      0.73      1880
weighted avg       0.80      0.74      0.73      1880
```

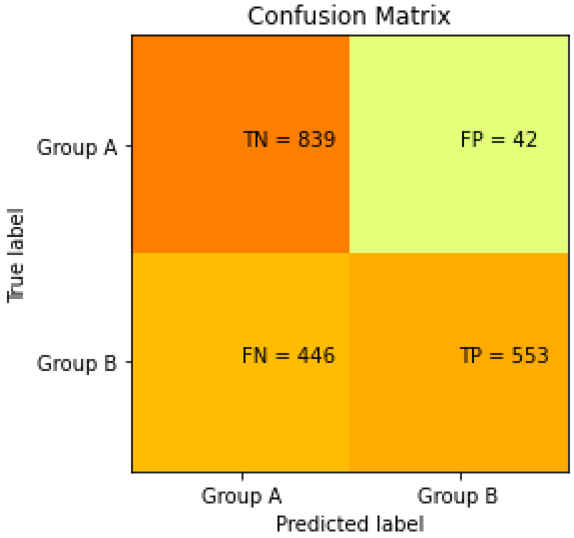


```
AUC Score : 0.7529402274010674

Accuracy Of the Model(AdaBoostClassifier): 0.7404255319148936
```

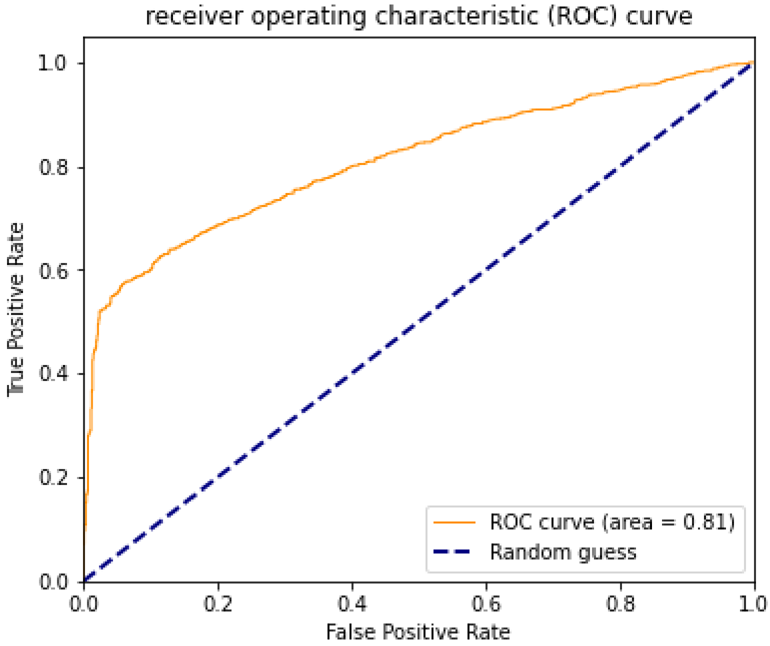

ROC_AUC Score of the model(AdaBoostClassifier): 0.8107477511563776

```
Confusion Matrix:
[[728 153]
 [347 652]]

Classification Report:
              precision    recall  f1-score   support

         0.0       0.68      0.83      0.74       881
         1.0       0.81      0.65      0.72       999

    accuracy                           0.73      1880
   macro avg       0.74      0.74      0.73      1880
weighted avg       0.75      0.73      0.73      1880
```

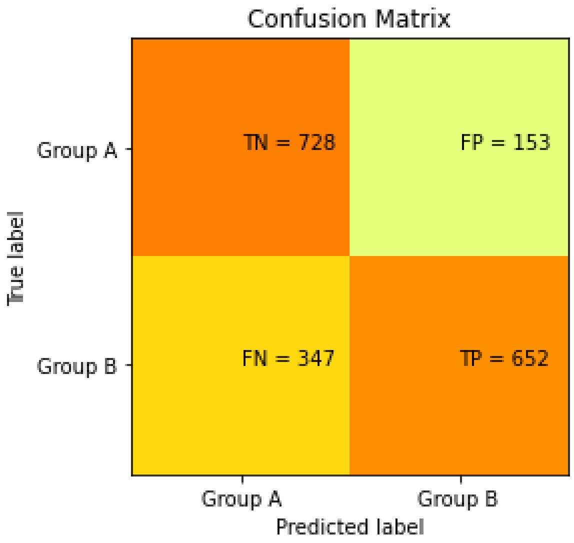

AUC Score : 0.7394931821719563

Accuracy Of the Model(SVC): 0.7340425531914894

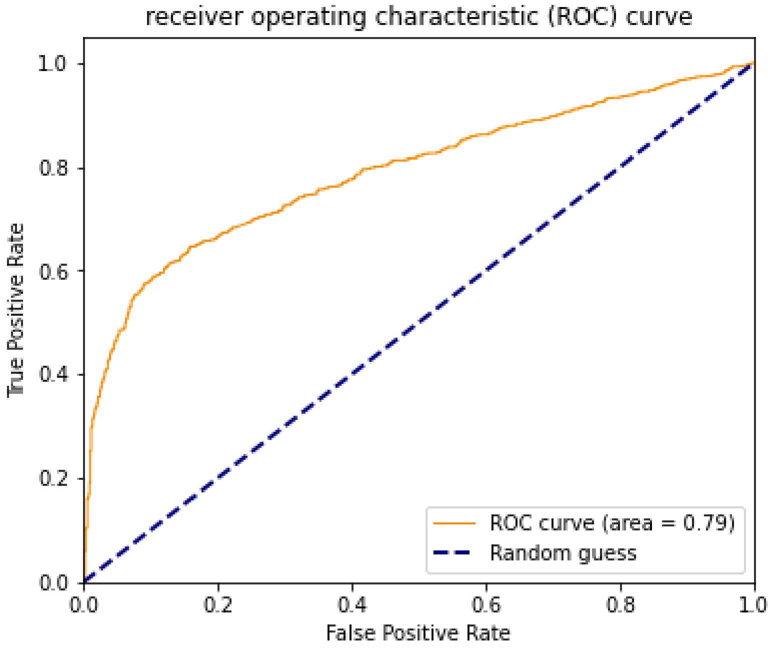

ROC_AUC Score of the model(SVC): 0.7883218064829869

```
Confusion Matrix:
[[808  73]
 [402 597]]

Classification Report:
              precision    recall  f1-score   support

         0.0       0.67      0.92      0.77       881
         1.0       0.89      0.60      0.72       999

    accuracy                           0.75      1880
   macro avg       0.78      0.76      0.74      1880
weighted avg       0.79      0.75      0.74      1880
```

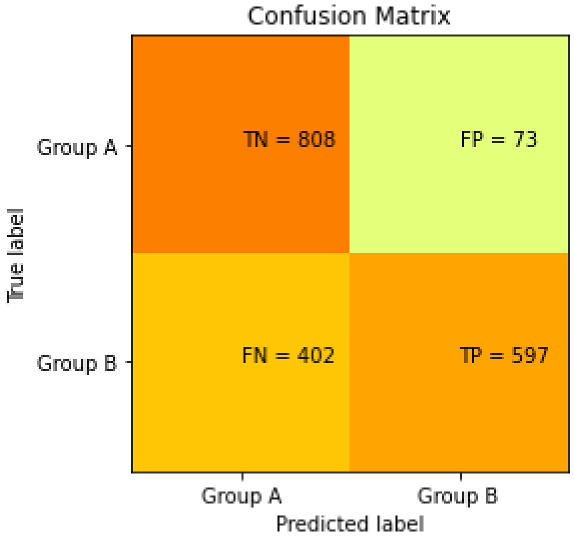

AUC Score : 0.7573686058362562

Accuracy Of the Model(MLPClassifier): 0.7473404255319149

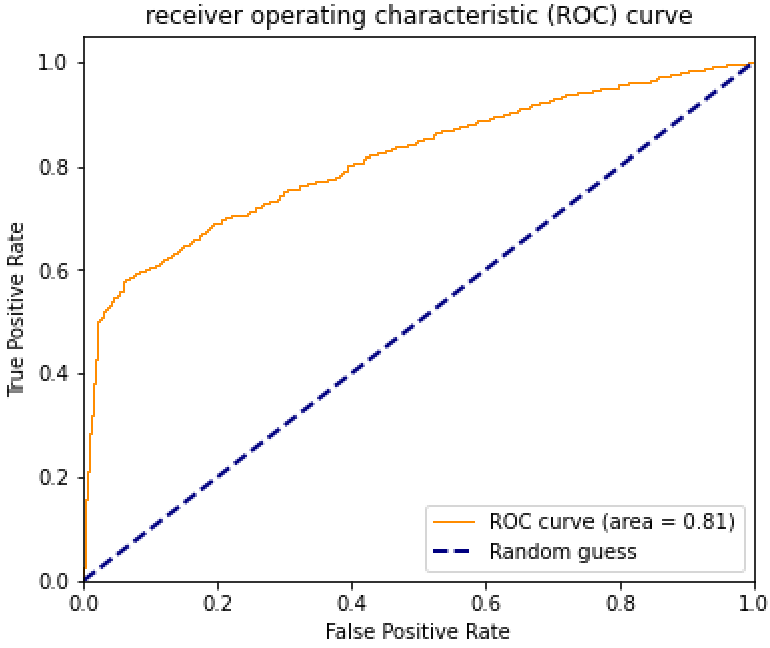

```
ROC_AUC Score of the model(MLPClassifier): 0.8140103781420466

[('MLPClassifier', 0.7473404255319149), ('GradientBoostingClassifier', 0.745744680
8510638), ('RandomForestClassifier', 0.7420212765957447), ('LogisticRegression',
0.7414893617021276), ('AdaBoostClassifier', 0.7404255319148936), ('XGBClassifier',
0.7398936170212767), ('ExtraTreesClassifier', 0.7388297872340426), ('DecisionTreeC
lassifier', 0.7367021276595744), ('SVC', 0.7340425531914894), ('KNeighborsClassifi
er', 0.7101063829787234)]


sorted_total_auc:
 [('RandomForestClassifier', 0.8141262715610049), ('MLPClassifier', 0.814010378142
0466), ('GradientBoostingClassifier', 0.8129707460013931), ('XGBClassifier', 0.811
7231874326085), ('LogisticRegression', 0.811255068916817), ('AdaBoostClassifier',
0.8107477511563776), ('ExtraTreesClassifier', 0.8072039121982368), ('KNeighborsCla
ssifier', 0.7943687160486252), ('DecisionTreeClassifier', 0.7904612898937531), ('S
VC', 0.7883218064829869)]
```

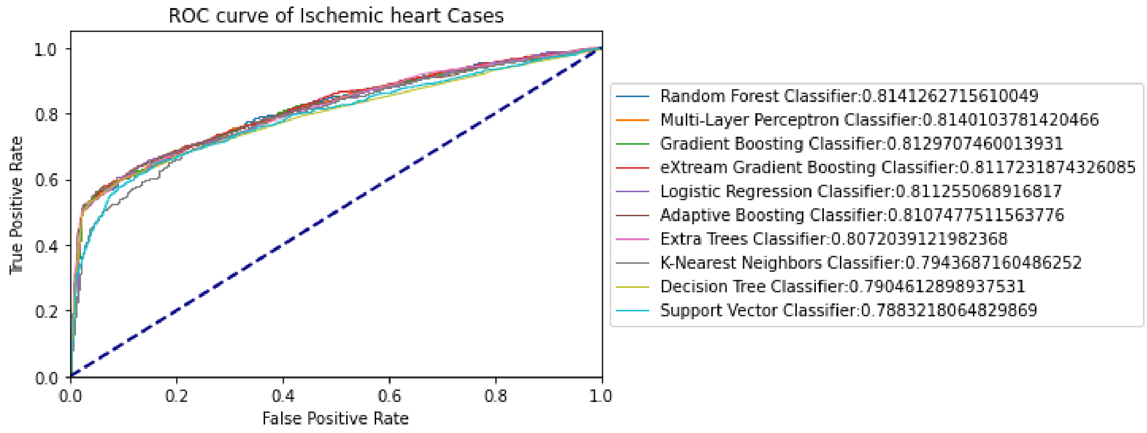

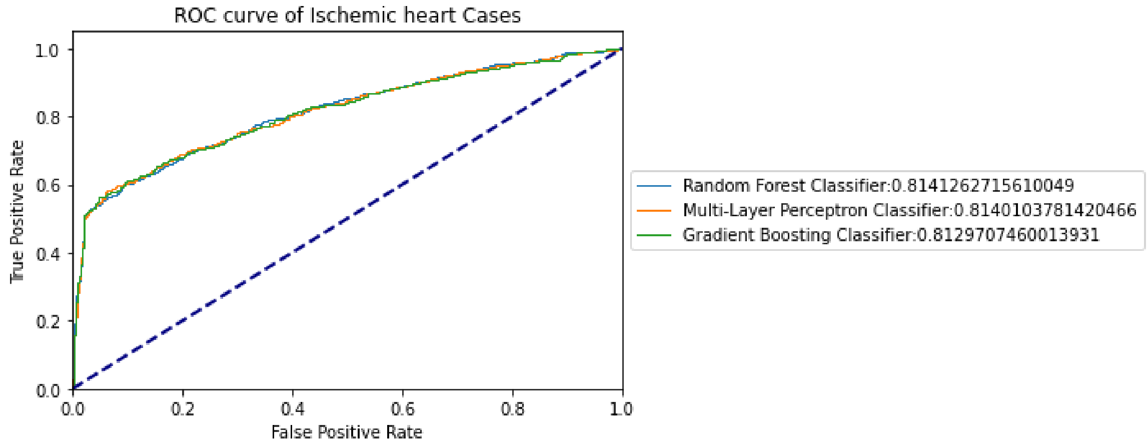

In [14]:

```
total_roc_aucs = total_roc_aucs.append(total_roc_auc, ignore_index=True)
total_roc_aucs
```

Out[14]:

	result_train_filename	RandomState	LogisticRegression	RandomForestClassifier	XGBClas
**0**	NaN	NaN	0.807767	0.818490	0.81
**1**	NaN	NaN	0.805630	0.810861	0.81
**2**	NaN	NaN	0.807484	0.817645	0.81
**3**	NaN	NaN	0.800775	0.805327	0.80
**4**	NaN	NaN	0.792836	0.802230	0.80
**5**	NaN	NaN	0.813980	0.816039	0.81
**6**	NaN	NaN	0.801266	0.813375	0.81
**7**	NaN	NaN	0.807361	0.810320	0.8[illegible]
**8**	NaN	NaN	0.804276	0.807234	0.8[illegible]
**9**	NaN	NaN	0.813435	0.817022	0.82
**10**	NaN	NaN	0.803254	0.809262	0.80
**11**	NaN	NaN	0.802989	0.806138	0.8[illegible]
**12**	NaN	NaN	0.807152	0.809747	0.8[illegible]
**13**	NaN	NaN	0.805277	0.814685	0.81
**14**	NaN	NaN	0.798803	0.807604	0.80
**15**	NaN	NaN	0.808474	0.813844	0.8[illegible]
**16**	NaN	NaN	0.805080	0.812329	0.81
**17**	NaN	NaN	0.811882	0.820931	0.81
**18**	NaN	NaN	0.800399	0.800578	0.80
**19**	NaN	NaN	0.801510	0.810064	0.8[illegible]
**20**	NaN	NaN	0.804132	0.809953	0.81
**21**	NaN	NaN	0.797401	0.802172	0.80
**22**	NaN	NaN	0.806470	0.813737	0.81
**23**	NaN	NaN	0.809953	0.818719	0.81
**24**	NaN	NaN	0.801446	0.815966	0.81
**25**	NaN	NaN	0.807810	0.814511	0.82
**26**	NaN	NaN	0.802652	0.807521	0.80
**27**	NaN	NaN	0.797224	0.810588	0.8[illegible]
**28**	NaN	NaN	0.807202	0.814581	0.81
**29**	NaN	NaN	0.811255	0.814126	0.8[illegible]
**30**	NaN	NaN	0.811255	0.814126	0.8[illegible]

In [15]:

```
total_roc_auc_file_name = 'total_roc_auc_' + str(kfold_split_number) + '_fold_test_result.xls'
print('total_roc_auc_file_name:', total_roc_auc_file_name)

total_roc_aucs.to_excel(total_roc_auc_file_name)
```

```
total_roc_auc_file_name: total_roc_auc_4_fold_test_result.xls
```

In [16]:

```
total_roc_aucs = pd.read_excel(total_roc_auc_file_name, index_col = 0)
```

In [17]:

```
total_roc_aucs
```

Out[17]:

	result_train_filename	RandomState	LogisticRegression	RandomForestClassifier	XGBClas
**0**	NaN	NaN	0.807767	0.818490	0.81
**1**	NaN	NaN	0.805630	0.810861	0.81
**2**	NaN	NaN	0.807484	0.817645	0.81
**3**	NaN	NaN	0.800775	0.805327	0.80
**4**	NaN	NaN	0.792836	0.802230	0.80
**5**	NaN	NaN	0.813980	0.816039	0.81
**6**	NaN	NaN	0.801266	0.813375	0.81
**7**	NaN	NaN	0.807361	0.810320	0.81
**8**	NaN	NaN	0.804276	0.807234	0.81
**9**	NaN	NaN	0.813435	0.817022	0.82
**10**	NaN	NaN	0.803254	0.809262	0.80
**11**	NaN	NaN	0.802989	0.806138	0.81
**12**	NaN	NaN	0.807152	0.809747	0.81
**13**	NaN	NaN	0.805277	0.814685	0.81
**14**	NaN	NaN	0.798803	0.807604	0.80
**15**	NaN	NaN	0.808474	0.813844	0.81
**16**	NaN	NaN	0.805080	0.812329	0.81
**17**	NaN	NaN	0.811882	0.820931	0.81
**18**	NaN	NaN	0.800399	0.800578	0.80
**19**	NaN	NaN	0.801510	0.810064	0.81
**20**	NaN	NaN	0.804132	0.809953	0.81
**21**	NaN	NaN	0.797401	0.802172	0.80
**22**	NaN	NaN	0.806470	0.813737	0.81
**23**	NaN	NaN	0.809953	0.818719	0.81
**24**	NaN	NaN	0.801446	0.815966	0.81
**25**	NaN	NaN	0.807810	0.814511	0.82
**26**	NaN	NaN	0.802652	0.807521	0.80
**27**	NaN	NaN	0.797224	0.810588	0.81
**28**	NaN	NaN	0.807202	0.814581	0.81
**29**	NaN	NaN	0.811255	0.814126	0.81
**30**	NaN	NaN	0.811255	0.814126	0.81

In [18]:

```
total_roc_aucs = total_roc_aucs.T
```

In [19]:

```
total_roc_aucs
```

Out[19]:

	0	1	2	3	4	5	
**result_train_filename**	NaN	NaN	NaN	NaN	NaN	NaN	[illegible]
**RandomState**	NaN	NaN	NaN	NaN	NaN	NaN	[illegible]
**LogisticRegression**	0.807767	0.805630	0.807484	0.800775	0.792836	0.813980	0.801
**RandomForestClassifier**	0.818490	0.810861	0.817645	0.805327	0.802230	0.816039	0.813
**XGBClassifier**	0.816900	0.816575	0.815168	0.805248	0.804874	0.812641	0.810
**KNeighborsClassifier**	0.797554	0.795950	0.801087	0.789633	0.779439	0.793897	0.787
**DecisionTreeClassifier**	0.791957	0.793250	0.793870	0.782085	0.780864	0.797154	0.788
**ExtraTreesClassifier**	0.811702	0.812073	0.806342	0.803125	0.793734	0.797301	0.804
**GradientBoostingClassifier**	0.817230	0.816970	0.818088	0.806002	0.802355	0.813804	0.810
**AdaBoostClassifier**	0.812444	0.810001	0.810170	0.797815	0.798456	0.801192	0.806
**SVC**	0.784843	0.785309	0.792830	0.775093	0.773914	0.779814	0.781
**MLPClassifier**	0.813147	0.813489	0.817989	0.803439	0.800705	0.812130	0.807

12 rows × 31 columns

In [20]:

```
total_roc_aucs.describe()
```

Out[20]:

	0	1	2	3	4	5	6	
**count**	10.000000	10.000000	10.000000	10.000000	10.000000	10.000000	10.000000	10.00000[illegible]
**mean**	0.807203	0.806011	0.808067	0.796854	0.792941	0.803795	0.801377	0.8001[illegible]
**std**	0.011695	0.010835	0.009610	0.010897	0.011050	0.011868	0.011142	0.0131[illegible]
**min**	0.784843	0.785309	0.792830	0.775093	0.773914	0.779814	0.781836	0.7754[illegible]
**25%**	0.800107	0.798370	0.802401	0.791678	0.783857	0.797191	0.791956	0.7933[illegible]
**50%**	0.812073	0.810431	0.808827	0.801950	0.796095	0.806661	0.805736	0.8053[illegible]
**75%**	0.815962	0.813135	0.817026	0.804796	0.801849	0.813513	0.809914	0.8098[illegible]
**max**	0.818490	0.816970	0.818088	0.806002	0.804874	0.816039	0.813375	0.8118[illegible]

8 rows × 31 columns

In [21]:

```
total_roc_aucs.describe().T.sort_values('mean', ascending=False)
```

Out[21]:

	count	mean	std	min	25%	50%	75%	max
**9**	10.0	0.809713	0.011510	0.789577	0.800036	0.813235	0.817402	0.822130
**2**	10.0	0.808067	0.009610	0.792830	0.802401	0.808827	0.817026	0.818088
**23**	10.0	0.807643	0.011002	0.787872	0.801561	0.810918	0.816527	0.818719
**0**	10.0	0.807203	0.011695	0.784843	0.800107	0.812073	0.815962	0.818490
**25**	10.0	0.806746	0.011951	0.783887	0.798913	0.808842	0.815217	0.820976
**1**	10.0	0.806011	0.010835	0.785309	0.798370	0.810431	0.813135	0.816970
**22**	10.0	0.805810	0.008876	0.790815	0.802737	0.806770	0.813486	0.814594
**17**	10.0	0.805807	0.013037	0.779203	0.796950	0.812082	0.812868	0.820931
**29**	10.0	0.805519	0.010275	0.788322	0.797578	0.811001	0.812659	0.814126
**30**	10.0	0.805519	0.010275	0.788322	0.797578	0.811001	0.812659	0.814126
**15**	10.0	0.804167	0.011227	0.780844	0.797067	0.807155	0.812303	0.817135
**16**	10.0	0.804140	0.009304	0.786540	0.797117	0.806126	0.811731	0.813349
**5**	10.0	0.803795	0.011868	0.779814	0.797191	0.806661	0.813513	0.816039
**20**	10.0	0.803160	0.010297	0.784456	0.795790	0.806227	0.809664	0.816866
**8**	10.0	0.802482	0.008966	0.785886	0.796025	0.805202	0.810014	0.811556
**19**	10.0	0.801987	0.010350	0.786965	0.793144	0.803595	0.809379	0.815113
**13**	10.0	0.801753	0.010095	0.782367	0.796863	0.804785	0.805681	0.814685
**28**	10.0	0.801584	0.012358	0.779659	0.794287	0.806864	0.810520	0.814581
**6**	10.0	0.801377	0.011142	0.781836	0.791956	0.805736	0.809914	0.813375
**10**	10.0	0.801236	0.010412	0.782143	0.798262	0.803369	0.808567	0.813298
**12**	10.0	0.801124	0.010643	0.783600	0.791012	0.807245	0.809389	0.811857
**7**	10.0	0.800189	0.013125	0.775494	0.793331	0.805334	0.809822	0.811885
**24**	10.0	0.800168	0.011364	0.778646	0.793451	0.800536	0.809391	0.815966
**27**	10.0	0.799563	0.009462	0.783728	0.793246	0.802754	0.804561	0.811322
**26**	10.0	0.797865	0.010827	0.777377	0.790373	0.802697	0.805560	0.809879
**11**	10.0	0.797675	0.012153	0.773210	0.789477	0.802571	0.806245	0.810811
**14**	10.0	0.797183	0.008898	0.782496	0.791314	0.799303	0.804351	0.807604
**3**	10.0	0.796854	0.010897	0.775093	0.791678	0.801950	0.804796	0.806002
**18**	10.0	0.794409	0.009762	0.778540	0.788542	0.799702	0.800692	0.805015
**4**	10.0	0.792941	0.011050	0.773914	0.783857	0.796095	0.801849	0.804874
**21**	10.0	0.792935	0.012972	0.765654	0.785861	0.794945	0.801281	0.809133

In [22]:

```
statistic_file_name = 'statistics_' + str(kfold_split_number) + '_fold_test_result.xls'
total_roc_aucs.describe().T.sort_values('mean', ascending=False).to_excel(statistic_file_name)
```

In [ ]:

```

```
