# Supplementary material for: Machine Learning-Based Cardiovascular Disease Prediction Model: A Cohort Study on the Korean National Health Insurance Service Health Screening Database
Source: Diagnostics (Basel). 2021 May 25;11(6):943. doi: 10.3390/diagnostics11060943 (PMC8229422; doi:10.3390/diagnostics11060943)
Supplement: Supplementary file 1 [file diagnostics-11-00943-s001.zip › Table S1.pdf]

Table S1. Factors used in ML and DL algorithm-based CVD prediction model

No.	Factor	Data Variables
1	Previous Cardiovascular Disease	HCHK_HDISE_PMH_YN
2	Total Cholesterol	TOT_CHOLE
3	High Density Lipoprotein	HDL_CHOLE
4	Body Mass Index	BMI
5	Waist-to-height ratio	WHTR
6	Fasting Blood Sugar	BLDS
7	Triglyceride	TRIGLYCERIDE
8	Hemoglobin	HMG
9	SGPT	SGPT_ALT
10	γGTP	GAMMA_GTP
11	Sex	SEX
12	Age Group	AGE_GROUP
13	Systolic Blood Pressure	BP_HIGH
14	Diastolic Blood Pressure	BP_LWST
15	Low Density Lipoprotein	LDL_CHOLE
16	Urine Protein	OLIG_PROTE_CD
17	Creatinine	CREATININE
18	SGOT	SGOT_AST
19	Past History of Hypertension	HCHK_HPRTS_PMH_YN
20	Family History of Cardiovascular disease	FMLY_HDISE_PATIEN_YN
21	More than 30 min Walking per a week	WLK30_WEK_FREQ_ID
22	Past History of Stroke	HCHK_APOP_PMH_YN
23	Past History of Diabetes	HCHK_DIABML_PMH_YN

24	Past History of Hyperlipidemia	HCHK_HPLPDM_PMH_YN
25	Past History of Pulmonary Tuberculosis	HCHK_PHSS_PMH_YN
26	Other Past Histories	HCHK_ETCDSE_PMH_YN
27	Family History of Stroke	FMLY_APOP_PATIEN_YN
28	Family History of Hypertension	FMLY_HPRTS_PATIEN_YN
29	Family History of Diabetes	FMLY_DIABML_PATIEN_YN
30	Family History of Cancer	FMLY_CANCER_PATIEN_YN
31	Past Smoking Period	PAST_SMK_TERM_RSPS_CD
32	Past Daily Smoking	PAST_DSQTY_RSPS_CD
33	Current Smoking Period	CUR_SMK_TERM_RSPS_CD
34	Current Daily Smoking	CUR_DSQTY_RSPS_CD
35	Drinking Habit	DRNK_HABIT_RSPS_CD
36	Intake Volume in one Drinking Session	TM1_DRKQTY_RSPS_CD
37	Vigorous Exercise more than 20 min per a week	MOV20_WEK_FREQ_ID
38	Moderate Exercise more than 30 min per a week	MOV30_WEK_FREQ_ID
